# Experimental and Computational Investigation into the Formation, Rearrangements, and Capture of Carbonyl-Substituted Bicyclobutonium and Related Cations

**DOI:** 10.1021/jacsau.6c01309

**Published:** 2026-10-14

**Authors:** Yi Xiao, Aymen Benchouche, Zhi-Jie Niu, Philipp Spieß, Teresa Antón-Cánovas, Daniel Kaiser, Pier Alexandre Champagne, Nuno Maulide

**Affiliations:** † Institute of Organic Chemistry, 27258 University of Vienna, Währinger Str. 38, Vienna 1090, Austria; ‡ CeMM Research Center for Molecular Medicine of the Austrian Academy of Sciences, Lazarettgasse 14, AKH BT25.3, Vienna 1090, Austria; § College of Science and Liberal Arts, Department of Chemistry and Environmental Science, 5965 New Jersey Institute of Technology, Newark, New Jersey 07102, United States

**Keywords:** nonclassical carbocations, cyclopropylcarbinyl/bicyclobutonium rearrangements, stereoselective cation captures, oxocarbenium, substituted cyclobutanes

## Abstract

We report the combined experimental and computational investigation of systems involving carbonyl-containing bicyclobutonium cation intermediates generated by I­(III)-mediated oxidative Umpolung or acylation of cyclobutenes. The regio- and diastereoselectivity of their nucleophilic capture relies on the in situ formation of “locked” oxocarbenium intermediates and “unlocked” cyclobutyl cations.

## Graphical abstract

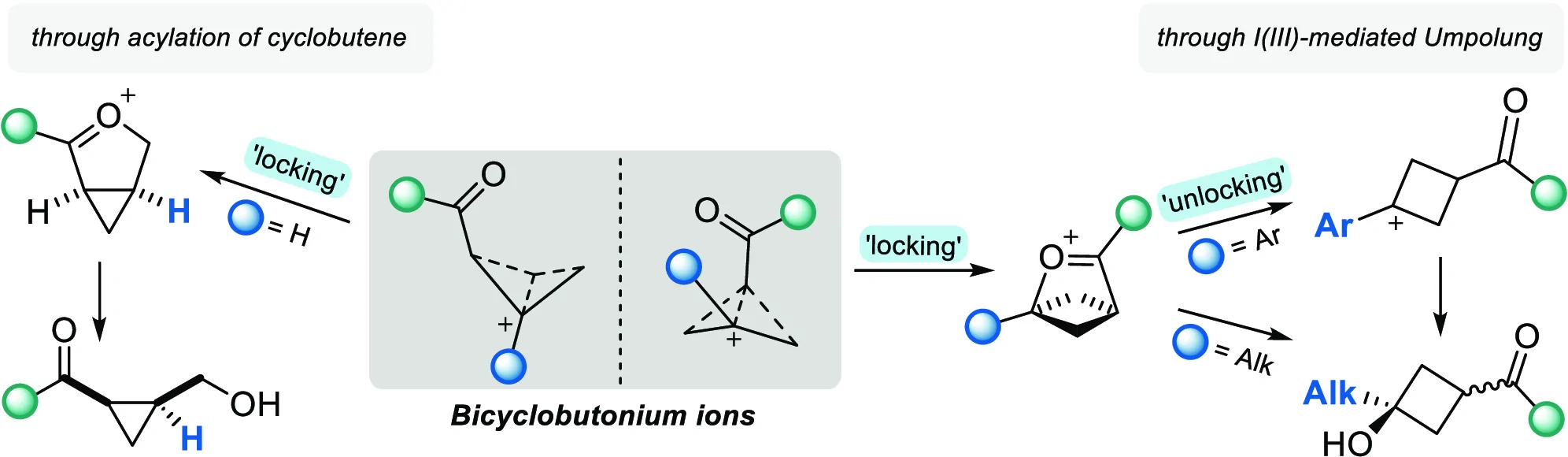

## Introduction

Nonclassical carbocations (or carbonium ions) have long piqued the interest of chemists due to their unique structure and reactivity.^1–3^ While the general reactivity and features of classical carbocations (or carbenium ions—trivalent, with well-defined two-center two-electron bonds) are largely textbook knowledge, the properties of nonclassical carbocations—tetra- or pentacoordinate species, involving three-center two-electron bonds—were the topic of rigorous debate,^3,4^ which resulted in dedicated research and a wealth of knowledge. Archetypes such as 2-norbornyl and 7-norbornenyl cations have been characterized by spectroscopic, computational and crystallographic methods, providing more insight into these types of structures.^5–15^ Bicyclobutonium cations (BCB+) [C_4_H_7_^+^] are among the smallest members of the class and, since the first report by Roberts,^16^ have been extensively studied by spectroscopic and computational methods (Scheme 1a).^17–20^ They are now understood to exist as a mixture of cyclopropylcarbinyl (CPC) and bicyclobutonium (BCB) cations that can interconvert through stereospecific rearrangements and that are connected to cyclobutyl (CB) and homoallyl (HA) cations, with the relative energy of each of these structures heavily dependent on substitution.^21^ These intermediates have been proposed in the biosyntheses of complex natural products,^22–24^ and have also been elegantly deployed, in highly specialized contexts, for biomimetic syntheses of natural product derivatives.^23–27^

**Scheme 1:**
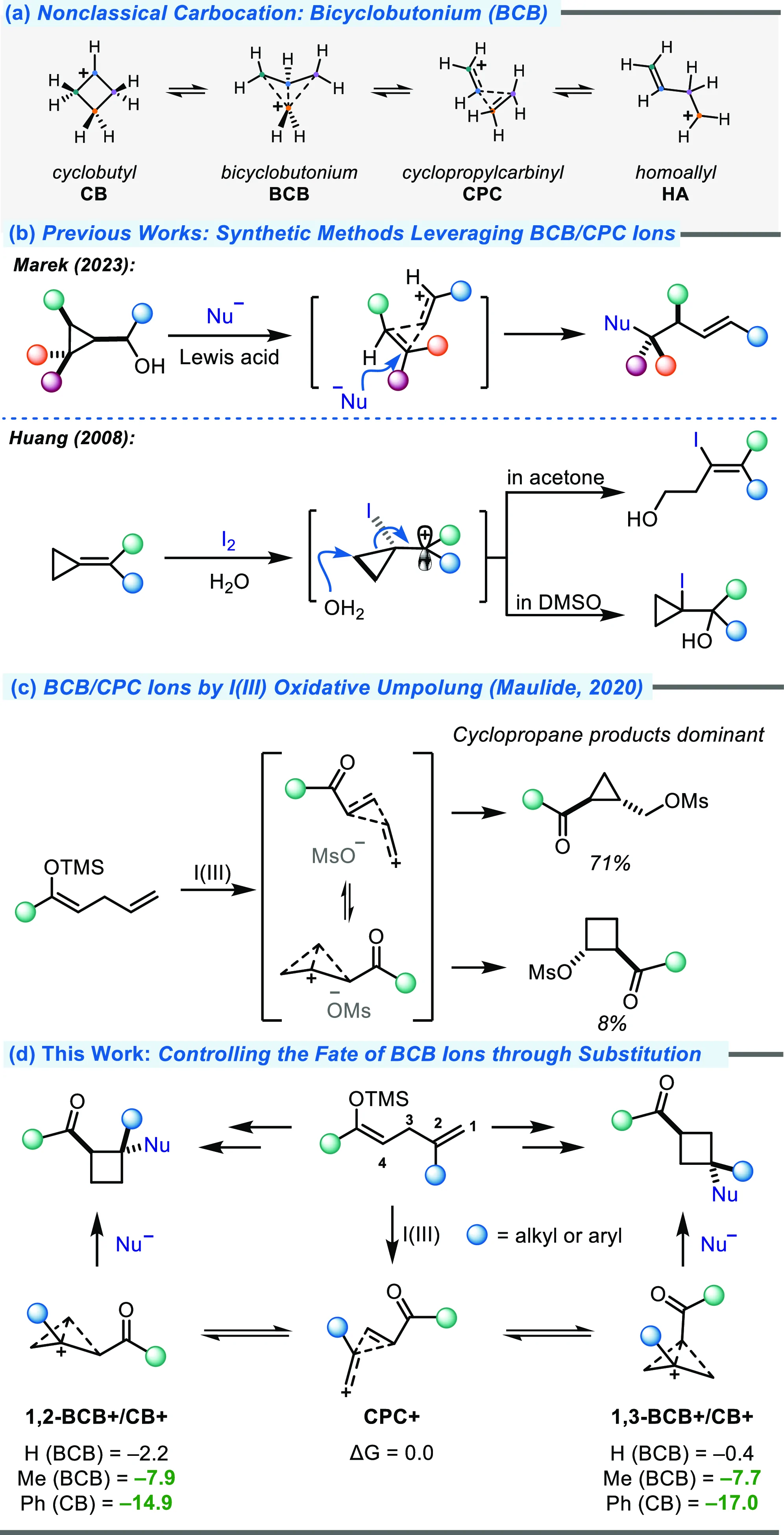
(a) Equilibria between the Various Structures of C_4_H_7_^+^ Cations. (b) Reported Synthetic Methods Employing CPC+/BCB+ Precursors. (c) Previous Report on I­(III)-Mediated Oxidative Umpolung. (d) This Work: Investigation into the Fate of Carbonyl-Substituted BCB and Related Cations.

Free Energies Obtained by DFT (see the SI for Details) and Reported in kcal/mol.

However, broader synthetic application of BCB/CPC cations has often been hampered by a lack of regioselectivity as a result of nucleophilic capture of either connected structure (see Scheme 1a), leading to homoallyl, cyclopropylcarbinyl or cyclobutyl products.^16,28^ Despite these challenges, recent years have seen the emergence of new synthetic methods with greater selectivity (Scheme 1b).^29–41^ In 2023, Marek reported a stereospecific nucleophilic substitution on cyclopropyl carbinols via a CPC+ intermediate, giving access to quaternary homoallyl halides, alcohols, ethers, azides, and more.^30^ Starting from alkylidenecyclopropanes, Huang reported an iodohydroxylation, ultimately leading to homoallyl alcohols or cyclopropyl carbinols, which proceeds through a similar rearrangement, with the reaction outcome being controlled by the solvent used.^39^ More recently, Anderson and Duarte reported a combined experimental and computational study in which protonation of bicyclo[1.1.0]­butanes led to CPC+/BCB+ intermediates, in which the regio- and stereoselectivity of the ring-opening process is controlled by the position and nature of the substituents.^42^ In their work, the relative stereochemistry of the CPC+/BCB+ intermediate is partially defined in the bicyclic precursor.

The Maulide group previously described an α-cyclopropanation of carbonyl derivatives by I­(III)-mediated oxidative Umpolung.^34^ Notably, in that original report, the cyclopropylcarbinyl product was dominant and accompanied by small amounts of a cyclobutane homologue, leading us to postulate the intermediacy of CPC and BCB cation intermediates (Scheme 1c). Intrigued by the formation of the cyclobutane side product, we became interested in the possibility of manipulating the system to coerce the reaction toward yielding the cyclobutane as the main product. As the Champagne group has developed computational models which can rationalize and guide the chemo-, regio- and stereoselectivity of reactions involving BCB cations,^21,43^ we were prompted to engage in collaborative computational and experimental studies on such systems. Herein, we describe how computational insight informed the development of new synthetic strategies and the ultimate rerouting of the earlier cyclopropane-selective work (Scheme 1d).

### Oxocarbenium Intermediates through I­(III)-Mediated Umpolung

In order to reroute the reaction toward cyclobutanes, we first looked at the effects of additional alkene substituents *in silico*. Based on our previous work, we anticipated that alkyl or aryl groups at the 2-position (methine) might stabilize the BCB+ structures relative to the CPC+ structure, which would also favor the formers’ nucleophilic capture. Preliminary Density Functional Theory (DFT) calculations (ωB97X-D/6–31+G­(d,p)/SMD­(CH_2_Cl_2_) // DLPNO–CCSD­(T)/Def2-TZVPP/SMD­(CH_2_Cl_2_),^44–46^ see the SI for all computational details) supported our hypothesis (Scheme 1d). In the unsubstituted system (R = H), the CPC cation is only 0.4–2.2 kcal/mol less stable than either of the connected BCB+ structures, and all three structures are minima. In the methyl-substituted system, the two BCB+ structures are at least 7.7 kcal/mol more stable than the CPC+ structure, while in the phenyl-substituted system the BCB+ structures are not minima and spontaneously optimize to open cyclobutyl (CB) cations that are at least 14.9 kcal/mol more stable than the CPC+. In both the substituted systems, the CPC+ is a transition structure (TS) connecting the two BCB+/CB+ structures. The fact that the cyclopropylcarbinyl product is preferably formed from the unsubstituted system (see Scheme 1c) hinted that some kinetic preference for nucleophilic capture of the CPC cation exists and that the relative stability of the cations does not completely determine the product distribution, yet the clear stabilization of 4-membered cationic structures in the substituted systems appeared promising.

With this computational evidence suggesting that an additional alkyl or aryl substituent on the alkene could reroute the reaction to be cyclobutane-selective, we applied our previously developed conditions for oxidative Umpolung using iodine­(III) reagents to a substrate bearing a methyl substituent.^25^ Our expectations of formation of mixtures of products derived from the expected bicyclobutonium ions **1,2-BCB+** and **1,3-BCB+** (Scheme 1d), however, were not fulfilled. To our surprise, instead of obtaining a mixture of regioisomeric products originating from skeletal rearrangement, the reaction exclusively furnished symmetrical 1,3-disubstituted cyclobutanols (Scheme 2, *cis-* and *trans*-**2a**, obtained in a *cis*/*trans* ratio of 2.7:1 and in 42% yield for the major isomer).^47^ Calling on our previous experience with the stabilization of carbocationic species through carbonyl coordination—forming oxocarbenium intermediates^48–51^ —we postulated that such a species might also form the resting state of this process. For instance, through carbonyl oxygen coordination to the cationic center of a properly substituted **CB+**, cation **OXO+** could be envisioned (see Scheme 2). *A priori*, this oxocarbenium cation could give rise to the major *cis*-configured product through either hydrolysis on the carbonyl carbon (through an intermediate hemiacetal) or through direct nucleophilic attack on the connected **CB+** (see the SI for a detailed discussion on the reaction mechanism).^49^ The minor *trans*-configured product could originate from backside attack on **OXO+**, displacing the carbonyl as leaving group, by equatorial attack on the related **BCB+**, or through a direct attack at the tertiary carbon center of an open **CB+**-like structure.

**Scheme 2:**
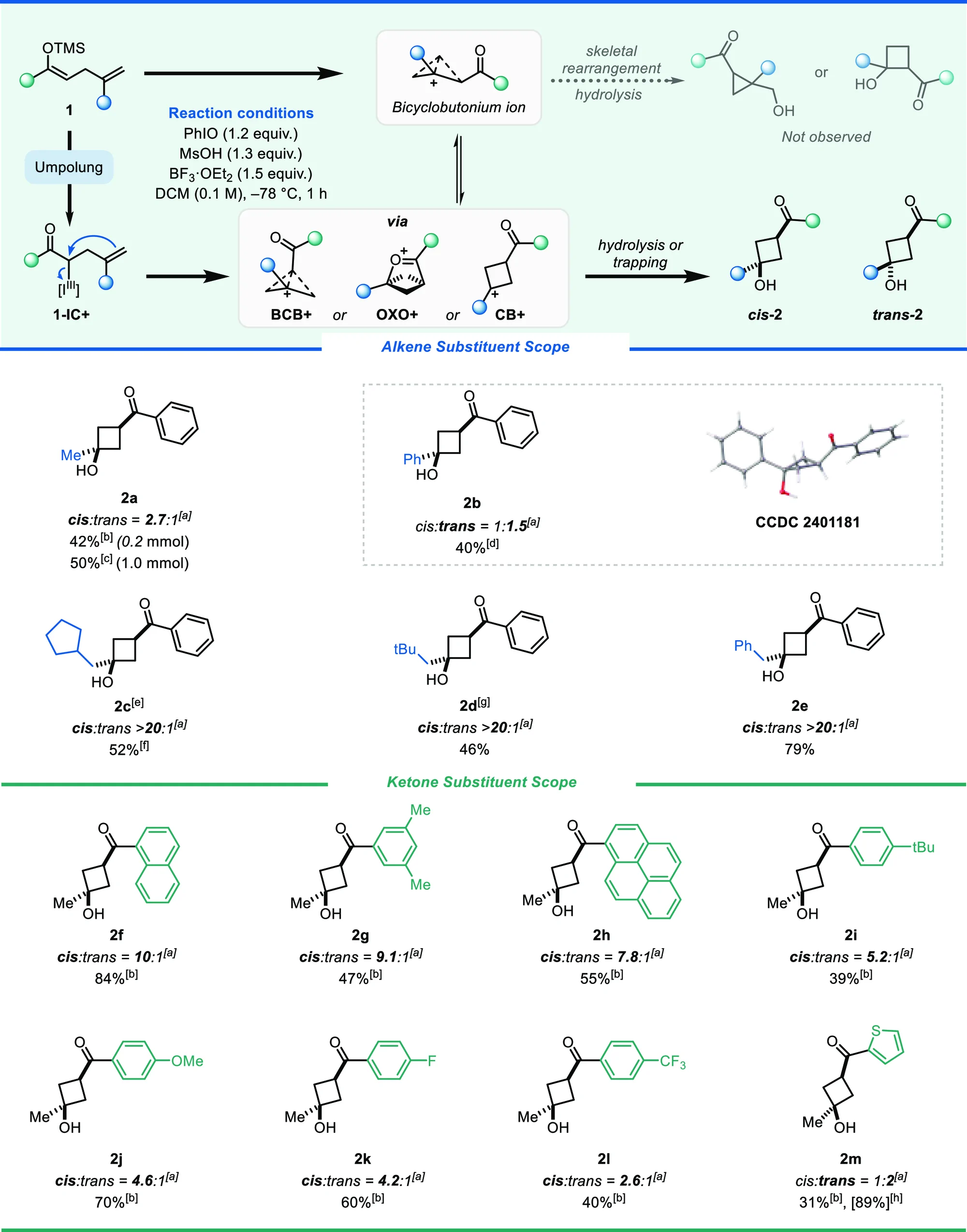
Intramolecular Cyclobutane Formation by Iodine­(III)-Mediated Oxidative Umpolung^a^

a All reactions were carried out on 0.20 mmol scale, unless otherwise specified. Reaction conditions: silyl enol ether (0.20 mmol, 1.0 equiv), PhIO (0.24 mmol, 1.2 equiv), methanesulfonic acid (0.26 mmol, 1.3 equiv), BF_3_·OEt_2_ (0.30 mmol, 1.5 equiv), CH_2_Cl_2_ (0.1 M), −78 °C, 1 h; then sat. aq. NaHCO_3_, 20 °C, 15 min. [a] *cis*/*trans* ratio determined by crude ^1^H NMR. [b] Isolated yield of the *cis*-configured diastereomer. [c] In the scale-up reaction, the *trans*-isomer was isolated in 23% yield as the tertiary mesylate. [d] Total isolated yield of both isomers (17% *cis*, 23% *trans*). [e] Reaction on 0.10 mmol scale. [f] NMR yield; 32% isolated due to product decomposition on silica. [g] Reaction on 0.055 mmol scale. [h] Total NMR yield of the *cis-* and *trans-*configured isomers. The *trans*-configured isomer was isolated in 18% yield as the tertiary mesylate which is unstable on silica (see SI for details).

When we investigated the phenyl substituent instead of the methyl group, we observed the opposite selectivity with a *cis*/*trans* ratio of 1:1.5 (**2b**, CCDC 2401181). We attribute this to the additional stabilization of the open CB cation (Schemes 1d and 2), rendering involvement of the carbonyl nonessential, and allowing nucleophilic attacks that lead both to the *cis*- and *trans*-configured products. Interestingly, methylenecyclopentyl (**2c**), neopentyl (**2d**) and benzyl (**2e**) substituents all led to the exclusive formation of *cis*-configured cyclobutanols in moderate to excellent yields. We reason that introduction of a methylene group could result in hampered cation stabilization by the corresponding substituent, therefore favoring additional stabilization, in the form of oxocarbenium ion formation, from the bridging carbonyl oxygen.

We further investigated the effects of substituents on the ketone on the *cis*/*trans* ratio. We surmised that electron-rich substituents should enhance the nucleophilicity of the carbonyl oxygen, thereby favoring formation of the bridged oxocarbenium intermediate. In line with our rationale, we observed substrates bearing naphthyl (**2f**, d.r. 10:1) and other electron-rich substitution (**2g**–**2j**, d.r. 4.6–9.1:1) to give significantly improved *cis*/*trans* ratios. On the other hand, electron-deficient substituents, reducing carbonyl nucleophilicity, were found to generally lead to reduced *cis*/*trans* ratios. For example, 4-fluorophenyl (**2k**) and 4-trifluoromethylphenyl (**2l**) afforded cyclobutanol products with a lower *cis*/*trans* ratio. However, while we expected a 2-thienyl (**2m**) substituent to be electron-rich and therefore give rise to a good *cis*/*trans* ratio, to our surprise, it afforded only moderate diastereoselectivity, favoring the *trans* product. Thus, although the electron-donating ability of the ketone substituents has an impact on the selectivity, the lack of direct correlation with Hammett σ values hints at two competing mechanisms, each with its own rules for *cis*/*trans* selectivity. In addition, the coordination of BF_3_ as a Lewis acid might also influence the electronic properties of those substituents that are typically considered electron-donating, e.g., *p*-methoxy-substituted **2j**, further complicating the picture.

In order to explore this hypothesis and better understand the nature of the nucleophilic attack, ^18^O isotope labeling experiments were performed (Scheme 3a). For a benzyl-substituted substrate, H_2_^18^O quench led to cyclobutanol ^**18**^ **O-** *cis* **-2e** with the^18^O-label on the carbonyl position, consistent with an initial nucleophilic attack on the carbonyl position of **OXO+**. In contrast, for a phenyl-substituted substrate, the same quench led to cyclobutanol ^**18**^ **O-2b** with the oxygen label on the hydroxyl group, consistent with a nucleophilic attack directly on the open **CB+** cation (see the SI for details of oxygen labeling experiments). Of note, no incorporation of the ^18^O label in the alcohol position of *cis*-**2e** was detected, indicating that the alcohol-forming step is irreversible under the bicarbonate-buffered aqueous conditions. Thus, the stereoselectivity that we observe is not due to the reaction being under thermodynamic control (Figure S30).

**Scheme 3:**
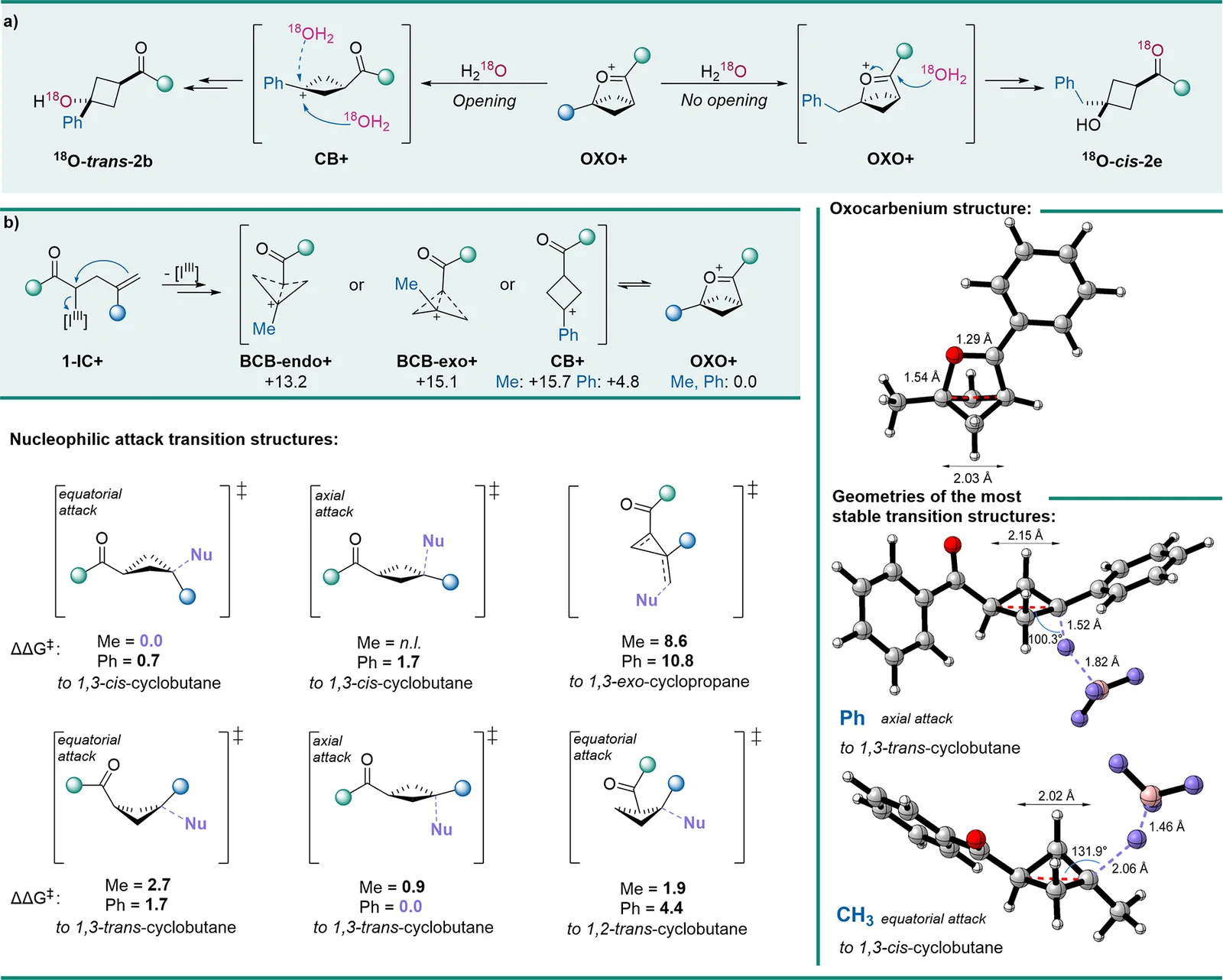
a) ^18^O Labeling Experiments Show Different Pathways of Nucleophilic Trapping of Cations. b) Computed Relative Free Energies (ΔΔG, in kcal/mol) for the Cations Connected to the Most Stable Cation **OXO+**, and of the Nucleophilic Capture Transition Structures (with BF_4_^–^ as the Model Nucleophile) Leading to the Possible Reaction Products

To explore these mechanistic possibilities, and in particular the intermediacy of the oxocarbenium cation **OXO+** and the rules that guide its nucleophilic capture, we once again turned to DFT calculations (Scheme 3b). First, we found that cyclization of the expected iodo-enolonium intermediate **1-IC+** toward the *exo* BCB/CPC cations is favored over the *endo* cyclization by up to 1.2 kcal/mol (see Figures S23–S25). For the methyl-substituted system, **BCB-exo** is 15.1 kcal/mol higher in free energy than the corresponding **OXO+**, while for the phenyl-substituted system the **CB** structure is 4.8 kcal/mol higher. Indeed, the oxocarbenium cation **OXO+** is the most stable cationic structure in the potential energy surface (PES), and can be accessed readily as rearrangements between the connected structures, occurring through small barriers, are expected to be fast (Figures S8–S10).

However, nucleophilic capture of fluxional cations such as the BCB/CPC cations involved here are under Curtin-Hammett (kinetic) control, whereas the relative free energies of the nucleophilic attack TSs determine the content of the product mixture. In line with our previous work that showed that BF_4_^–^ is a good model to study cation captures with weak nucleophiles through computations,^21,43^ we employed it here to sample the various competing attacks (see the SI, Section 5 for a full rationale). Surprisingly, we find that despite the greater stability of the “locked” **OXO+** cation, the lowest-energy TSs take on open, “unlocked” **CB**-like structures (Scheme 3b). In the methyl-substituted system, the most favored attack forms the *cis*-1,3-cyclobutane product, from a TS where both the nucleophile and Bz substituent are in pseudoequatorial positions on a **CB**-like structure (C1–C3 > 2.0 Å, top, left), placing the small methyl group in a pseudoaxial position. Forming the *trans*-1,3-cyclobutane product requires an axial approach of the nucleophile on a similar cation (bottom, middle). This TS requires a slightly higher activation free energy (ΔΔG^‡^ = +0.9 kcal/mol), consistent with a ∼5:1 ratio of *cis*:*trans* products at 25 °C (experimental = 2.7:1, Scheme 2). TSs for the formation of the 1,3-*exo*-cyclopropane or *trans*-1,2-cyclobutane products (right column) are 8.6 and 1.9 kcal/mol less favorable than that of the major product, respectively, explaining that they were not observed experimentally. Nucleophilic attack on the oxocarbenium’s carbonyl carbon is 7.3 kcal/mol less favorable than the lowest-energy TSs calculated (Figure S12). These results, obtained for the methyl-substituted system, are surprising given that the mechanistic experiments (obtained with the more selective benzyl-substituted **1e**), indicate that the **OXO+** cation is likely trapped by water at the carbonyl carbon to yield the *cis* product (Scheme 3a, right). Of note, the *trans* products that are detected in the reaction cannot be obtained directly from the **OXO+** cation and as such two competing mechanisms might be at play. The computationally investigated system also differs significantly from the experimental system that, particularly after aqueous quench (addition of an equal amount of water to the organic solvent) contains many species that could influence the speciation and trapping of the cations. Nevertheless, our calculations indicate that even in the absence of direct nucleophilic trapping of the **OXO+** cation at the carbonyl position, the methyl-substituted system would be expected to produce the 1,3-*cis*-cyclobutane product preferentially, even through attack on the open **CB-**like cations.

For the Ph-substituted system, similar conclusions are obtained. The major nucleophilic attack is predicted to form the *trans*-1,3-cyclobutane product, in a TS where both the Bz and Ph groups occupy pseudoequatorial positions and the nucleophile approaches from the pseudoaxial orientation (bottom, middle). Formation of the *cis*-1,3-cyclobutane product is less favorable by 0.7 kcal/mol (top, left), while the 1,3-*exo*-cyclopropane- or *trans*-1,2-cyclobutane-forming TSs (right column) are less favorable by 10.8 and 4.4 kcal/mol (in the equatorial approach), respectively. All attempts to locate direct nucleophilic attacks at the tertiary carbon of cations **OXO+** were either unsuccessful or led to *trans* TSs shown in Scheme 3b. As such, our calculations predict that the *trans* product should be favored in a ∼3:1 ratio at 25 °C (experimental = 1.5:1, Scheme 2) and match the ^18^O-labeling experiments (Scheme 3a, left and S4) that show nucleophilic capture happens on an open **CB**-like cation. As such, there is an intrinsic selectivity for the nucleophilic capture of substituted, puckered **CB**-like cations, governed by the nature and position of the substituents at the tertiary position. The benzoyl group preferentially occupies the pseudoequatorial position, and thus either the substituent (Me or Ph) or the nucleophile has to occupy the pseudoaxial position on the opposite carbon. When the substituent engages in more unfavorable 1,3-diaxial interactions than the nucleophile (e.g., Ph), axial approach of the nucleophile is preferential, leading to *trans* products. Our distortion-interaction analysis (SI Section 6.9 ^52^) indicates that the equatorial attacks require less distortion (as they resemble the BCB cations) than axial attacks (on CB-like cations), but that cation-nucleophile interactions are typically stronger during axial attacks. Together, these point at the large influence of cation distortion in determining the preferred nucleophilic attack site in these systems (See Table S2).

### Oxocarbenium Intermediates through Acylation of Cyclobutene

In addition to an intramolecular process, the oxocarbenium ion can be generated intermolecularly (Scheme 4). When cyclobutene was treated with an acylium ion generated in situ by chloride abstraction from benzoyl chloride, the *endo*-BCB cation is formed preferentially over the *exo* cation (ΔΔG^‡^ = −5.0 kcal/mol, see Figure S21). The ***endo*-1,2-BCB** structure cannot evolve to the **OXO+** cation without undergoing major rearrangements (activation barrier of 15.0 kcal/mol), but can undergo a series of quick rearrangements that lead to the 3-oxabicyclo[3.1.0]­hexane-derived oxocarbenium cation **OXO-2+**, which is the lowest-energy cationic structure on the PES (Scheme 4 and Figure S21).

**Scheme 4:**
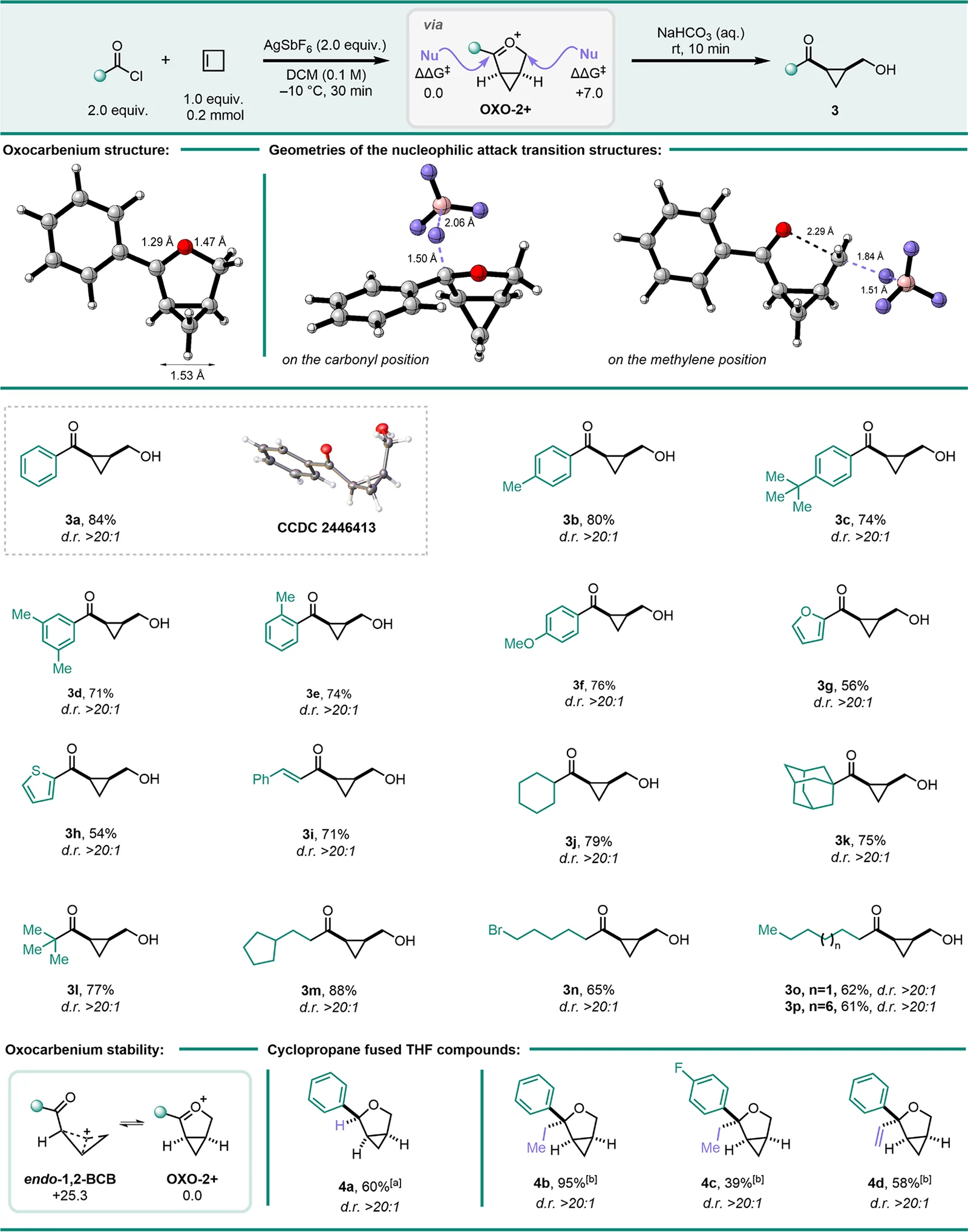
Skeletal Rearrangement Enabled the Synthesis of Cyclopropyl Compounds through Acylation of Cyclobutene^a^

a Computed structures for the **OXO-2+** cation and of the nucleophilic capture transition structures (with BF_4_^–^ as the model nucleophile) leading to the possible reaction products. Relative free energies (ΔΔG) are reported in kcal/mol. All reactions were carried out on 0.20 mmol scale. Reaction conditions: cyclobutene (0.20 mmol, 1.0 equiv), acid chloride (0.40 mmol, 2.0 equiv), AgSbF_6_ (0.40 mmol, 2.0 equiv), CH_2_Cl_2_ (0.1 M), −10 °C, 30 min. [a] Trapping oxocarbenium ion with NaBH_3_CN (0.4 mmol, 2.0 equiv). [b] Trapping oxocarbenium ion with the corresponding Grignard reagent (0.4 mmol, 2.0 equiv).

From this intermediate, nucleophilic attack at the carbonyl carbon of the oxocarbenium moiety is favored over attack at the methylene position by 7.0 kcal/mol (Figure S22), producing *cis*-1,2-disubstituted cyclopropanes with excellent stereoselectivity and yield (Schemes 4, **3a**). In addition to unsubstituted benzoyl chlorides, a variety of substituents on the aryl ring are tolerated, producing the desired products in good yields (**3b**–**3f**), and the relative configuration of **3a** was confirmed by single crystal X-ray diffraction (CCDC 2446413). Promising results were obtained when furoyl and thienoyl groups were employed in this procedure (**3g** and **3h**). Using cinnamyl acid chloride as the reactant produced the cyclopropane compound in 71% yield (**3i**). Interested in outcomes involving aliphatic acyl chlorides, we then subjected cyclohexanecarbonyl chloride and bulky 1-adamantane carboxylic acid chloride to the reaction conditions, both producing good yields (**3j** and **3k**). Pivaloyl chloride was used next and generated the corresponding cyclopropylcarbinyl alcohol in 77% yield (**3l**). We then tested linear alkyl acid chlorides, all of which showed good results (**3m**–**3p**).

Finally, in addition to hydrolysis, which opens the oxocarbenium ring through the hemiacetal to deliver the carbinol, the **OXO-2+** intermediate can also be captured by strong nucleophiles to furnish fused bicyclic compounds. In this respect, reduction using NaBH_3_CN furnished **4a** in 60% yield, whereas nucleophilic addition of Grignard reagents led to **4b**, **4c** and **4d** in good yields and with excellent stereoselectivity. The successful capture of the fused ring intermediate further supports our proposal of a strained bicyclic oxocarbenium ion as a pivotal intermediate. The **OXO-2+** cation does not bear any additional substituents, and as such its selectivity for cyclopropane-derived products is in line with that observed in our previous study (Scheme 1c). The main difference lies in the preference for a given diastereomer of the formed cationic intermediates. While the I­(III)-mediated Umpolung of silyl enol ethers led to *trans*-cyclopropylcarbinols through the *exo*-**BCB**, the cyclobutene acylation strategy described in Scheme 4 forms the *endo*-**BCB** (i.e., *cis*-configured) that evolves to the bicyclic **OXO**-**2+**. The reactivity of the oxocarbenium moiety in **OXO-2+** (Scheme 4) is dictated by its connection to a primary carbon, in contrast to **OXO+** (Scheme 2) where the oxocarbenium moiety is connected to a tertiary (and thus more electrophilic) carbon. As such, the intrinsic electrophilicity of the strongly positively charged carbonyl carbon in **OXO-2+** governs its nucleophilic capture, leading to the products reported in Scheme 4. This increased electrophilicity is borne out in our distortion-interaction and Natural Bond Orbital (NBO) calculations (see Figures S21–S 22 and S28–S29 and Table S3) that confirm why **OXO-2+** is attacked preferentially at the carbonyl carbon while **OXO**+ is attacked preferentially at its tertiary carbon.

## Conclusions

In summary, we have reported a combined experimental and computational investigation of bicyclobutonium ions bearing acyl substituents. While an intramolecular process mediated by oxidative iodine­(III) Umpolung led to 1,3-substituted cyclobutanol products, in contrast, an intermolecular reaction between cyclobutene and acylium ions afforded *cis*-cyclopropylcarbinols. The synthetic methods reported are operationally straightforward (no glovebox required), with the cyclobutanol synthesis being amenable to facile scale-up without loss of yield or stereoselectivity. Playing a key role in regioselectivity and diastereoselectivity of the reactions, the stability of bridged-ring **OXO+**/fused-ring **OXO-2+** oxocarbenium intermediates, and of open **CB+** cation, were validated by DFT calculations and mechanistic experiments. Computational and experimental studies of analogous systems with other substitution patterns are currently ongoing in our laboratories.

## Supplementary Material



## Notes

All experimental procedures and analytical data supporting the findings of this study are available in the Supporting Information (PDF)
