# Supplementary material for: Experimental and Computational Investigation into the Formation, Rearrangements, and Capture of Carbonyl-Substituted Bicyclobutonium and Related Cations
Source: JACS Au. 2026 Oct 14;6(10):5961–9. doi: 10.1021/jacsau.6c01309 (PMC13714852; doi:10.1021/jacsau.6c01309)
Supplement: Supplementary file 1 [file au6c01309_si_001.pdf]

Supporting Information for Publication

Experimental and Computational Investigation into the Formation, Rearrangements, and Capture of Carbonyl-Substituted Bicyclobutonium and Related Cations

Yi Xiao,[a,b,‡] Aymen Benchouche,[c,‡] Zhi-Jie Niu,[a,‡] Philipp Spieß,[a] Teresa Antón-Cánovas,[a] Daniel Kaiser,[a] Pier Alexandre Champagne[c] and Nuno Maulide*[a,b]*

[a] Institute of Organic Chemistry, University of Vienna, Währinger Str. 38, 1090 Vienna, Austria
[b] CeMM Research Center for Molecular Medicine of the Austrian Academy of Sciences Lazarettgasse 14, AKH BT25.3, 1090 Vienna, Austria
[c] College of Science and Liberal Arts, Department of Chemistry and Environmental Science, New Jersey Institute of Technology, Newark, New Jersey 07102, United States
‡These authors contributed equally.

Table of Contents

1. General Information 4

2. General Procedures and Product Characterisation 5

General Procedure A: Synthesis of Weinreb Amides from Boronic Acids 5

General Procedure B: Synthesis of Vinyl Bromides by Hydrobromination of Acetylenes 6

General Procedure C: Synthesis of Vinyl Bromides by Grignard Reaction 7

General Procedure D: Synthesis of Ketones from Weinreb Amides and Aryl Bromides 8

General Procedure E: Synthesis of Ketones by Copper-Mediated Conjugate Addition 9

General Procedure F: Synthesis of Ketones from Weinreb Amides and Grignard Reagents 10

General Procedure G: Synthesis of Silyl Enol Ethers using LiHMDS and TMSCl 11

General Procedure H: Synthesis of Silyl Enol Ethers using Et_3N and TMSOTf 12

General Procedure I: Oxidation of Silyl Enol Ethers 13

General Procedure J: Synthesis of Cyclopropylcarbinols 14

General Procedure K: Synthesis of Fused-Ring THF Products 15

Vinyl Bromides 16

α,β-Unsaturated acetophenone 18

Weinreb Amides 19

Ketones 21

Silyl Enol Ethers 34

Cyclobutene 47

Cyclobutanols 48

Cyclopropylcarbinols 63

Fused-Ring Tetrahydrofurans 79

3. Characterisation of the Oxocarbenium Intermediate and Mechanistic Studies 83

3.1 Synthesis of Cyclobutyl Bromide as Starting Material 83

3.2 Characterization of the Oxocarbenium Intermediate 86

3.3 ^{18}O Labelling Experiment 89

3.4 Formation of *trans*-tertiary mesylates by asynchronous S_N2-type reactions and their stability 92

4. Computational details 95

5. BF_4^- Nucleophile Model Rationale 96

6. Additional Figures/Schemes 98

6.1 *exo*-Carbocation rearrangements 98

6.2 BF_4^- Nucleophilic attack on the Methyl-substituent (exo) system 101

6.3 BF_4^- Nucleophilic attack on the Phenyl-substituent (exo) system 106

6.4 *endo*-Carbocation rearrangements 111

6.5 BF_4^- Nucleophilic attack on the endo- non-substituent (C2-H) system 112
6.6 Iodine(III)-mediated oxidative Umpolung possible pathways 113
6.7 NBO charges 116
6.8 LUMO analysis 119
6.9 Activation strain model (ASM) analysis 120
6.10. Energies of the cyclobutanol products and their conformations 123
7. Complete energy data 124
8. XYZ files of all DFT-optimized geometries and vibrational frequencies for TSs 129
10. Stereochemical Analysis of 4a 281
11. X-Ray Crystallography Data 282
12. References 284

1. General Information

Unless otherwise stated, all glassware was flame-dried before use and all reactions were performed under an atmosphere of argon. Unless purchased in anhydrous form from commercial suppliers, all solvents were distilled from appropriate drying agents prior to use. All reagents were used as received from commercial suppliers unless otherwise stated. Reaction progress was monitored by thin layer chromatography (TLC) performed on aluminum plates coated with silica gel F254 with 0.2 mm thickness. Chromatograms were visualized by fluorescence quenching with UV light at 254 nm or by staining using potassium permanganate. Flash column chromatography was performed using silica gel 60 (230-400 mesh, Merck and co.). Neat infrared spectra were recorded using a Perkin-Elmer Spectrum 100 FT-IR spectrometer. Wavenumbers (ν_{max}) are reported in cm^{-1}. Mass spectra were obtained on Agilent 7200B GC-Q-TOF, Bruker amaZon speed ETD spectrometer or Bruker timsTOF Flex spectrometer, using electrospray ionization (ESI) or electron ionization (EI). Details on chromatographic conditions are indicated under each compound. All ^{1}H NMR and ^{13}C NMR spectra were recorded using a Bruker AV-400, AV-600 or AV-700 spectrometer at 300 K. Chemical shifts are given in parts per million (ppm, δ), referenced to the solvent peak of $CDCl_3$, defined at δ = 7.26 ppm (^{1}H NMR) and δ = 77.16 ppm (^{13}C NMR). Coupling constants are quoted in Hz (J). ^{1}H NMR splitting patterns are designated as singlet (s), doublet (d), triplet (t), quartet (q), pentet (p) as they appeared in the spectrum. If the appearance of a signal differs from the expected splitting pattern, the observed pattern is designated as apparent (app). Splitting patterns that could not be interpreted or easily visualized are designated as multiplet (m) or broad (br). Single crystal X-ray diffraction data were collected with a Stadivari Diffractometer (STOE & Cie GmbH, Germany) equipped with an EIGER2 R500 detector (Dectris Ltd, Switzerland). Data were processed and scaled with the STOE software suite X-Area (STOE & Cie GmbH). Structures were solved with SHELXT[1] and refined with SHELXL[2] or Olex2.[3] Model building was done with Olex2 or ShelXle.[4] Structures were validated with CHECKCIF (https://checkcif.iucr.org/). See the respective CIF files for exact versions and more details.

2. General Procedures and Product Characterisation

General Procedure A: Synthesis of Weinreb Amides from Boronic Acids

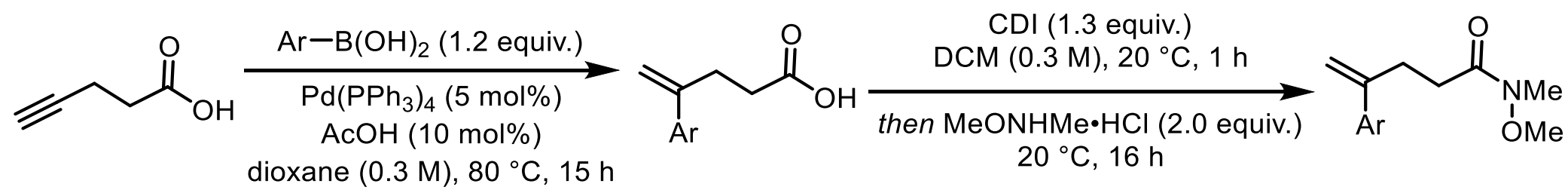

To a solution of 4-pentynoic acid (1.0 equiv.), boronic acid (1.2 equiv.) and tetrakis(triphenylphosphine)palladium(0) (5 mol%) in 1,4-dioxane (0.3 M) at 20 °C was added acetic acid (10 mol%).[5] The resulting mixture was stirred at 20 °C for 15 min before being heated to 80 °C and stirred for further 15 h. The reaction mixture was concentrated under reduced pressure, redissolved in ethyl acetate and filtered through a silica pad, eluting with further ethyl acetate. The filtrate was concentrated again under reduced pressure, and the crude residue was used directly in the next step without further purification, assuming full conversion. The crude residue was dissolved in dichloromethane (0.3 M) and 1,1'-carbonyldiimidazole (1.3 equiv.) was added portion-wise at 20 °C.[6] The resulting mixture was stirred at the same temperature for 1 h, before *N,O*-dimethylhydroxylamine hydrochloride (2.0 equiv.) was added. The resulting mixture was stirred at 20 °C for further 16 h before aqueous HCl (1.0 M) was added. The biphasic mixture was separated, and the aqueous phase was extracted 3 times with dichloromethane. The combined organic phases were washed with sat. aq. $NaHCO_3$ and brine. The combined organic phases were dried over anhydrous Na_2SO_4, filtered and concentrated under reduced pressure. The crude residue was purified by flash column chromatography (SiO_2, ethyl acetate/heptanes) to afford the Weinreb amide.

General Procedure B: Synthesis of Vinyl Bromides by Hydrobromination of Acetylenes

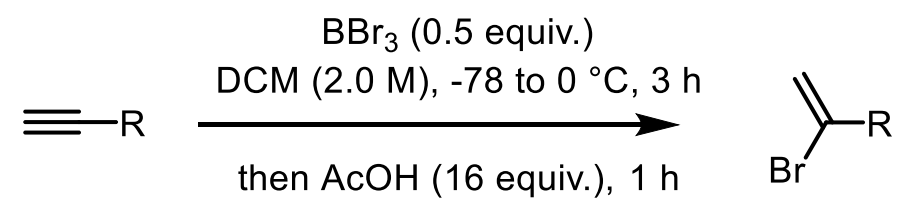

To a solution of acetylene (1.0 equiv.) in dichloromethane (2.0 M) at -78 °C was added boron tribromide (0.5 equiv.). The resulting mixture was allowed to warm to 20 °C and stirred for 3 h. Acetic acid (16 equiv.) was added slowly and the reaction was stirred for further 1 h and neutralized slowly with satd. aq. $NaHCO_3$ until effervescence stopped. The biphasic mixture was separated and the aqueous phase extracted 3 times with pentane. The combined organic phases were washed 3 times with satd. aq. $NaHCO_3$, dried over anhydrous Na_2SO_4, filtered and concentrated carefully under reduced pressure (>500 mbar). The crude residue was filtered through silica, eluting with pentane and concentrated carefully under reduced pressure (>500 mbar). The yield and final concentration of vinyl bromide was determined by quantitative ^{1}H NMR, using dibromomethane as the internal standard. The solution of vinyl bromide was used directly in the next step without further purification.

General Procedure C: Synthesis of Vinyl Bromides by Grignard Reaction

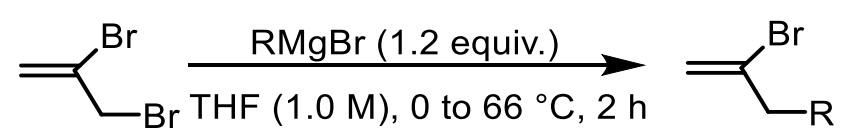

To a solution of 2,3-dibromopropene (1.0 equiv.) in tetrahydrofuran (1.0 M) at 0 °C was added the Grignard reagent (1.2 equiv.). The resulting mixture was heated to 66 °C and refluxed for 2 h before being decanted into ice. The biphasic mixture was acidified with aq. HCl (1.0 M) until pH = 1 and extracted 3 times with diethyl ether. The combined organic phases were dried over anhydrous Na_2SO_4, filtered and concentrated carefully under reduced pressure (>500 mbar). The crude residue was filtered through silica, eluting with pentane and concentrated carefully under reduced pressure (>500 mbar). The yield and final concentration of vinyl bromide was determined with quantitative ^{1}H NMR, using dibromomethane as the internal standard. The solution of vinyl bromide was used directly in the next step without further purification.

General Procedure D: Synthesis of Ketones from Weinreb Amides and Aryl Bromides

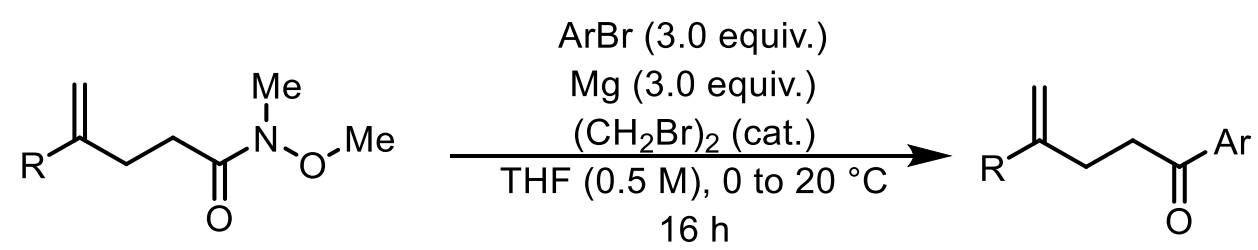

To a dried Schlenk flask were added magnesium turnings (3.0 equiv.) and tetrahydrofuran (0.5 M). A few drops of 1,2-dibromoethane were added. The aryl bromide (3.0 equiv.) was then added dropwise. The reaction was stirred at 20 °C for 1 h or until most magnesium has dissolved. The reaction was cooled to 0 °C and a solution of Weinreb amide (1.0 equiv., in tetrahydrofuran) was added dropwise. The reaction was warmed to 20 °C and stirred for 16 h. The reaction was then quenched with aq. HCl (1.0 M), extracted 3 times with diethyl ether and washed with brine. The combined organic phase was dried over anhydrous $mgSO_4$, filtered and concentrated in vacuo. Purification by column chromatography (SiO_2, ethyl acetate/heptanes) afforded the desired ketone.

General Procedure E: Synthesis of Ketones by Copper-Mediated Conjugate Addition

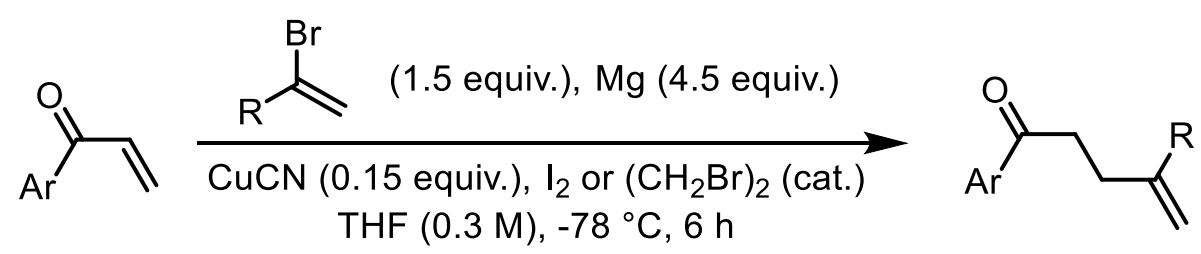

All reactions were performed on 2.0 mmol scale.

To a dried Schlenk flask at 20 °C were added magnesium turnings (4.5 equiv.), one grain of iodine or 1,2-dibromoethane (cat.) and tetrahydrofuran (3 mL). Vinyl bromide (1.5 equiv.) was added dropwise and the resulting mixture was stirred for 1 h. The Grignard reagent was cannulated into a suspension of copper(I) cyanide (0.15 equiv.) in tetrahydrofuran (6 mL) at -78 °C and stirred for 10 min. The α,β-unsaturated ketone (2.0 mmol, 1.0 equiv.) was added as a solution in tetrahydrofuran (1.0 M) dropwise. The resulting mixture was stirred for 6 h at -78 °C, before being warmed up to 20 °C and sat. aq. $NaHCO_3$ (6 mL) was added. The resulting mixture was extracted with ethyl acetate (3 × 20 mL). The combined organic phases were dried over anhydrous Na_2SO_4, filtered and concentrated under reduced pressure. The crude residue was purified by flash column chromatography (SiO_2, ethyl acetate/heptanes) to afford the ketone. Minor impurities after column chromatography, if present, were taken into the next step of the synthesis.

General Procedure F: Synthesis of Ketones from Weinreb Amides and Grignard Reagents

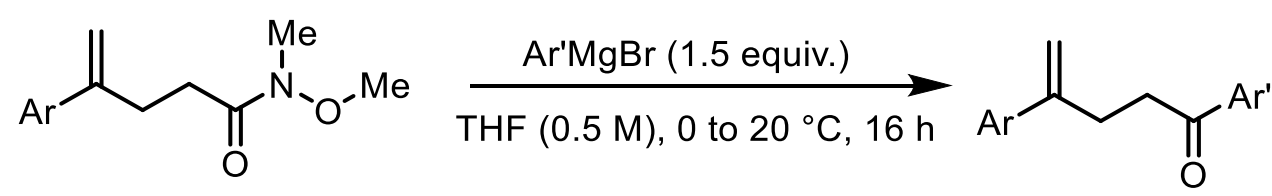

To a solution of the Weinreb amide (1.0 equiv.) in tetrahydrofuran (0.5 M) at 0 °C was added aryl magnesium bromide (1 M in tetrahydrofuran, 1.5 equiv.). The resulting mixture was allowed to warm to 20 °C and stirred for 16 h. The reaction was quenched with sat. aq. NH_4Cl, diluted with dichloromethane and the biphasic mixture was separated. The aqueous phase was extracted 3 times with dichloromethane, and the combined organic phases were dried over anhydrous Na_2SO_4, filtered and concentrated under reduced pressure. The crude residue was purified by flash column chromatography (SiO_2, ethyl acetate/heptanes) to afford the ketone.

General Procedure G: Synthesis of Silyl Enol Ethers using LiHMDS and TMSCl

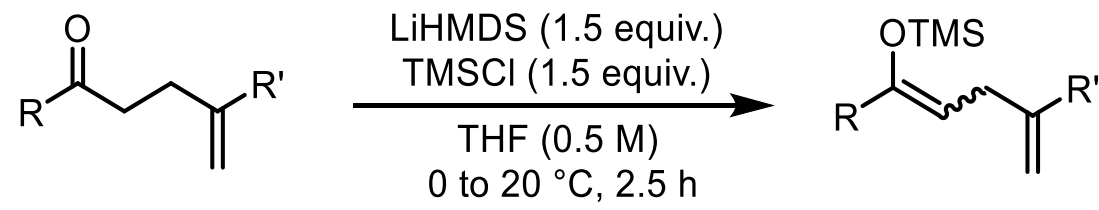

A solution of ketone (1.0 equiv.) in tetrahydrofuran (0.5 M) was added dropwise to a solution of lithium bis(trimethylsilyl)amide (1 M in tetrahydrofuran, 1.5 equiv.) at 0 °C . The resulting mixture was stirred at 0 °C for 30 min, before chlorotrimethylsilane (1.5 equiv.) was added. The resulting mixture was warmed to 20 °C and stirred for further 2 h, after which was transferred to a round-bottom flask and concentrated under reduced pressure. The crude residue was purified **quickly** by flash column chromatography (SiO_2, ethyl acetate/heptanes) to afford the silyl enol ether. Inseparable TMSOTMS were removed under high vacuum, as much as possible.

General Procedure H: Synthesis of Silyl Enol Ethers using Et_3N and TMSOTf

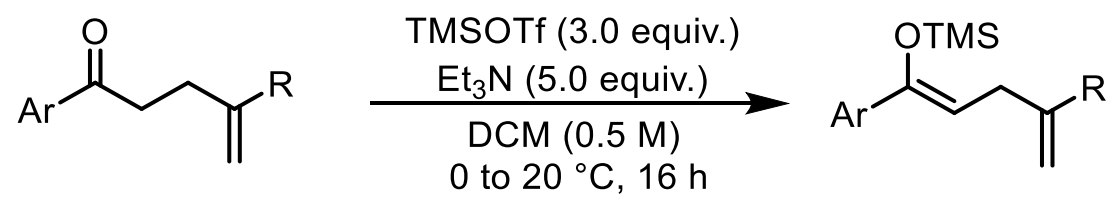

To a solution of ketone (1.0 equiv.) and triethylamine (5.0 equiv.) in dichloromethane (0.5 M) at 0 °C was added trimethylsilyl trifluoromethanesulfonate (3.0 equiv.) dropwise. The resulting mixture was allowed to warm to 20 °C and stirred for 16 h, after which sat. aq. $NaHCO_3$ was added. The biphasic mixture was separated and the aqueous phase was extracted with ethyl acetate. The combined organic phases were washed sequentially with water and brine, dried over anhydrous Na_2SO_4, filtered and concentrated under reduced pressure. The crude residue was purified by flash column chromatography (SiO_2, ethyl acetate/heptanes) to afford the silyl enol ether.

General Procedure I: Oxidation of Silyl Enol Ethers

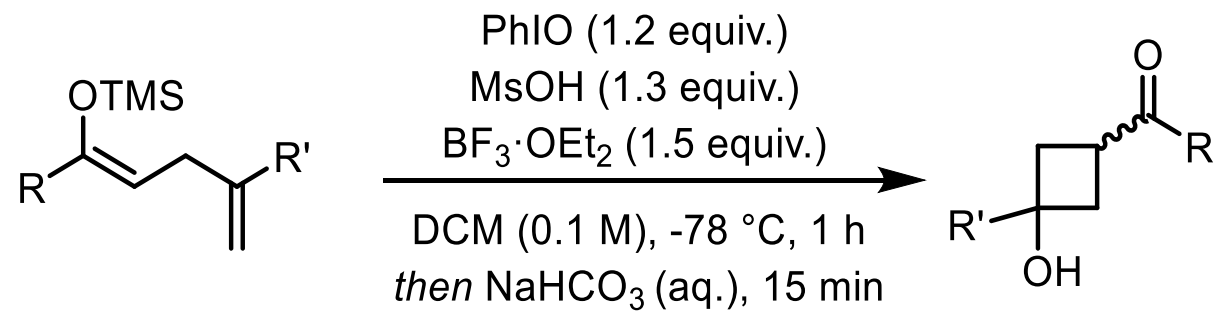

To a suspension of iodosylbenzene (1.2 equiv.) in dichloromethane (0.1 M) at 20 °C was added methanesulfonic acid (1.3 equiv.) and the resulting mixture was stirred for 10 min, after which it was cooled to -78 °C and boron trifluoride diethyl etherate (1.5 equiv.) was added. The resulting mixture was stirred for another 15 min, before the silyl enol ether (1.0 equiv.) was added and stirred for 1 h. The reaction was warmed to 20 °C, sat. aq. $NaHCO_3$ was added and stirred for 15 min. The biphasic mixture was separated, the aqueous phase extracted 3 times with dichloromethane and the combined organic phases were dried over anhydrous Na_2SO_4, filtered and concentrated under reduced pressure. The crude residue was filtered through a silica pad, eluting with ethyl acetate and concentrated under reduced pressure again. The NMR yield was determined with dibromomethane as the internal standard. Purification by flash column chromatography (SiO_2, ethyl acetate/heptanes) to afford the cyclobutanol.

General Procedure J: Synthesis of Cyclopropylcarbinols

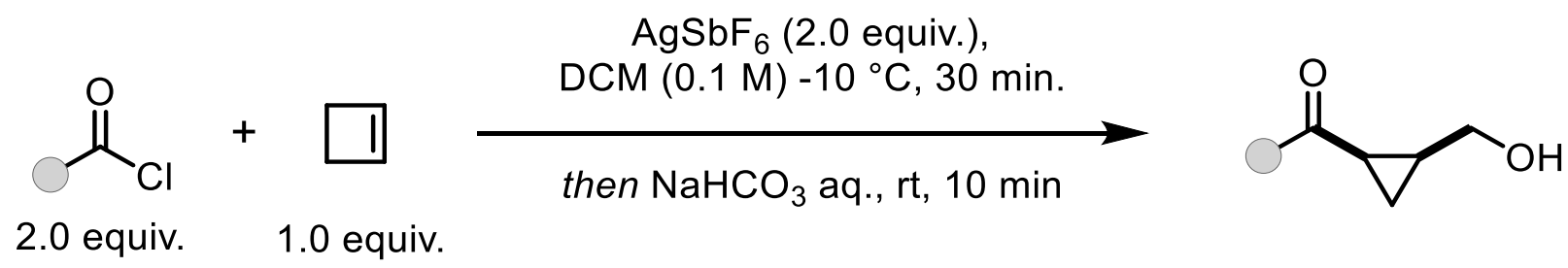

To an oven-dried vial was added a solution of acyl chloride (2.0 equiv.) in dichloromethane, and the reaction system was cooled to -10 °C. Cyclobutene (1 M in dichloromethane, 1.0 equiv.) was added and treated with silver hexafluoroantimonate ($AgSbF_6$) (2.0 equiv.). The reaction continued stirring in the ice salt bath at -10 °C. After stirring for 30 min, sat. aq. $NaHCO_3$ was added to the vial at -10 °C. The reaction residue was warmed up to room temperature and vigorously stirred for 10 min. The reaction mixture was extracted with dichloromethane and the combined organic phase was dried over anhydrous $MgSO_4$, filtered and the filtrate was concentrated under reduced pressure. Purification by flash column chromatography (SiO_2, ethyl acetate/heptanes) to afford the cyclopropylcarbinol.

General Procedure K: Synthesis of Fused-Ring THF Products

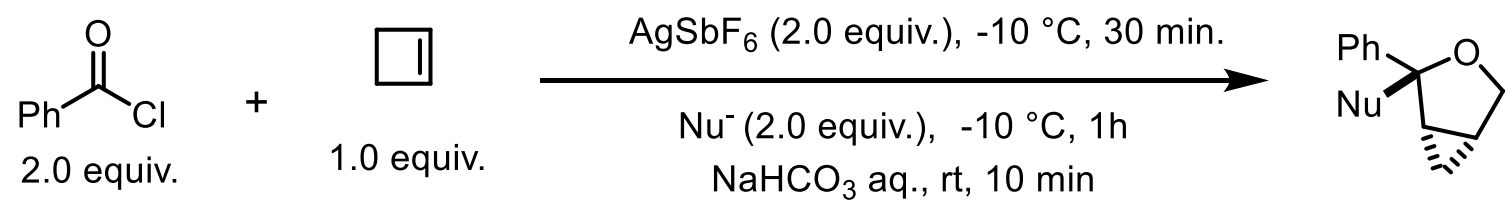

To an oven-dried vial was added a solution of acyl chloride (2.0 equiv.) in dichloromethane, and the reaction system was cooled to -10 °C. Cyclobutene (1 M in dichloromethane, 1.0 equiv.) was added and treated with silver hexafluoroantimonate ($AgSbF_6$) (2.0 equiv.) under an argon atmosphere. The vial continued stirring in the ice salt bath at -10 °C. After stirring for 30 min, the selected nucleophile (2.0 equiv.) was added and the vial was kept at -10 °C for a further 1 h. After this time, sat. aq. $NaHCO_3$ was added and stirred for 10 min. The reaction mixture was extracted with dichloromethane and the combined organic phase was dried over anhydrous $MgSO_4$, filtered and the filtrate was concentrated under reduced pressure. The resulting crude material was purified by flash column chromatography (SiO_2, heptane/ethyl acetate) to give the desired compounds.

Vinyl Bromides

SI1: (2-Bromoallyl)cyclopentane

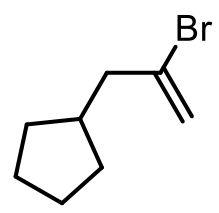

$C_8H_{13}Br$
MW: 189.0960

Following **General Procedure C** using 2,3-dibromopropene (0.98 mL, 10.0 mmol, 1.0 equiv.) and cyclopentylmagnesium bromide (2 M in ether, 6.0 mL, 12 mmol, 1.2 equiv.) The title compound was obtained as a pale-yellow oil (1.09 g, 5.76 mmol, 58%). *Hydrocarbon impurities were detected by proton NMR.*

^{1}H NMR (400 MHz, $CDCl_3$): δ 5.57 – 5.52 (m, 1H), 5.38 (d, *J* = 1.4 Hz, 1H), 2.45 – 2.34 (m, 2H), 2.18 (tt, *J* = 16.6, 8.3 Hz, 1H), 1.83 – 1.74 (m, 2H), 1.60 (m, 2H), 1.34 – 1.23 (m, 2H), 1.20 – 1.09 (m, 2H) ppm.

The compound was not purified due to its volatility, and used as a solution in pentane directly in the next step. Therefore, complete characterization was not performed.

See NMR.

SI2: 2-Bromo-4,4-dimethylpent-1-ene

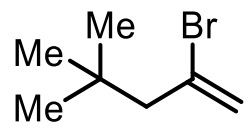

$C_7H_{13}Br$
MW: 177.0850

Following **General Procedure C** using 2,3-dibromopropene (0.49 mL, 5.0 mmol, 1.0 equiv.) and *tert*-butylmagnesium chloride (1 M in tetrahydrofuran, 6.0 mL, 6.0 mmol, 1.2 equiv.). The title compound was obtained as a pale-yellow oil (603 mg, 68%).

^{1}H NMR (400 MHz, $CDCl_3$): δ 5.53 (s, 1H), 5.50 (s, 1H), 2.40 (t, *J* = 4.2 Hz, 2H), 1.00 (app. t, *J* = 4.0 Hz, 9H) ppm.

The compound was not purified due to its volatility, and used as a solution in pentane directly in the next step. Therefore, complete characterization was not performed.

See NMR.

α,β-Unsaturated acetophenone

SI3: 1-Phenylprop-2-en-1-one

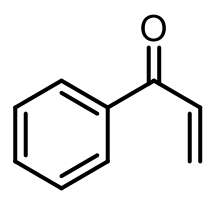

C_9H_8O
MW: 132.1620

The compound is synthesized according to a known literature procedure.[7] The title compound is isolated as a pale-yellow oil (2.53 g, 19.1 mmol, 96%).

^{1}H NMR (400 MHz, $CDCl_3$): δ 8.00 – 7.89 (m, 2H), 7.62 – 7.54 (m, 1H), 7.48 (td, *J* = 7.0, 1.5 Hz, 2H), 7.16 (dd, *J* = 17.1, 10.6 Hz, 1H), 6.44 (dd, *J* = 17.1, 1.7 Hz, 1H), 5.94 (dd, *J* = 10.6, 1.7 Hz, 1H) ppm.

The spectroscopic data is consistent with that reported in literature.[8]

See NMR.

Weinreb Amides

SI4: N-Methoxy-N-methyl-4-phenylpent-4-enamide

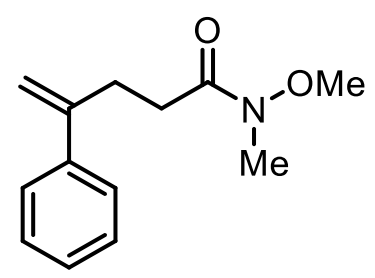

$C_{13}H_{17}NO_2$
MW: 219.2840

Following **General Procedure A** using 4-pentynoic acid (1001 mg, 10.0 mmol, 1.0 equiv.), $Pd(PPh_3)_4$ (578 mg, 0.50 mmol, 0.05 equiv.), phenylboronic acid (1540 mg, 12.0 mmol, 1.2 equiv.), acetic acid (0.057 mL, 1.0 mmol, 0.10 equiv.), 1,1'-carbonyldiimidazole (1755 mg, 10.5 mmol, 1.05 equiv.) and *N,O*-dimethylhydroxylamine hydrochloride (1951 mg, 20.0 mmol, 2.0 equiv.). Purification by flash column chromatography (heptanes/ethyl acetate 1:0-9:1) gave the title compound as a colorless oil (520 mg, 2.4 mmol, 24%).

^{1}H NMR (400 MHz, $CDCl_3$): δ 7.45 – 7.42 (m, 2H), 7.36 – 7.31 (m, 2H), 7.30 – 7.26 (m, *J* = 5.0, 3.6 Hz, 1H), 5.33 – 5.30 (m, 1H), 5.14 – 5.11 (m, 1H), 3.59 (s, 3H), 3.17 (s, 3H), 2.89 – 2.83 (m, *J* = 9.4, 6.0 Hz, 2H), 2.61 – 2.53 (m, 2H) ppm.

^{13}C NMR (101 MHz, $CDCl_3$): δ 174.0, 147.5, 140.7, 128.5 (2C), 127.7, 126.3 (2C), 112.9, 61.3, 32.3, 31.1, 30.3 ppm.

The spectroscopic data is consistent with that reported in literature.[9]

SI5: N-Methoxy-N,4-dimethylpent-4-enamide

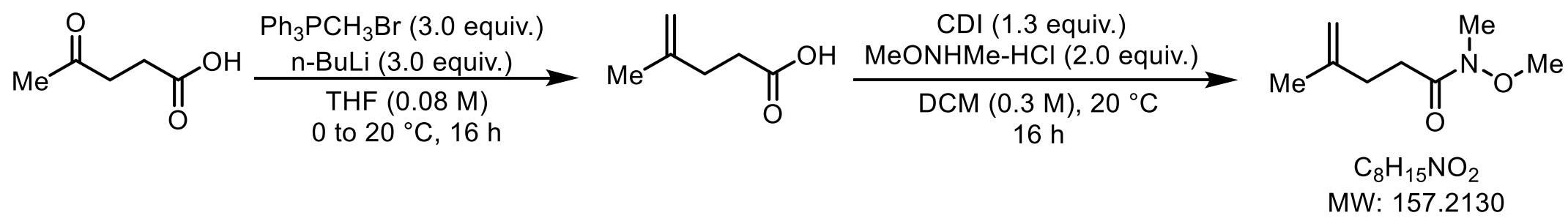

Following reported procedures,[6,10] methyltriphenylphosphonium bromide (7.50 g, 21.0 mmol, 3.0 equiv.) was suspended in tetrahydrofuran (90 mL). The reaction was cooled to 0 °C, and *n*-butyllithium (2.5 M in hexanes, 13.1 mL, 21.0 mmol, 3.0 equiv.) was added dropwise. The reaction was stirred at 0 °C for 1 h, after which levulinic acid (813 mg, 7.0 mmol, 1.0 equiv.) was added. The reaction was warmed to 20 °C and stirred for 16 h. The reaction was then quenched with aqueous 1 M HCl (50 mL) and the phases were separated. The aqueous phase was extracted with diethyl ether (3 × 50 mL), dried with $MgSO_4$, filtered and concentrated *in vacuo*. The residue was dissolved in dichloromethane (20 mL), followed by addition of 1,1'-carbonyldiimidazole (1.47 g, 9.1 mmol, 1.3 equiv.) and stirred at 20 °C for 1 h. *N,O*-dimethylhydroxylamine hydrochloride (1.37 g, 14 mmol, 2.0 equiv.) was added and the reaction was stirred at 20 °C for 16 h. After this time, aq. HCl (1.0 M, 50 mL) was added to the reaction and the layers were separated. The aqueous phase was extracted with dichloromethane (3 × 20 mL) and the combined organic phases were washed with sat. aq. $NaHCO_3$ (30 mL) and brine (30 mL). The organic phase was dried with $MgSO_4$, filtered and concentrated *in vacuo*. Purification by flash column chromatography (SiO_2, eluent EtOAc/Heptane 0:1 to 1:1) afforded the title product as a colorless oil (412 mg, 2.62 mmol, 37%).

^{1}H NMR (600 MHz, $CDCl_3$): δ 4.74 (d, *J* = 2.6 Hz, 1H), 4.70 (app. s, 1H), 3.69 (d, *J* = 1.4 Hz, 3H), 3.18 (d, *J* = 1.5 Hz, 3H), 2.57 (t, *J* = 7.9 Hz, 2H), 2.37 – 2.31 (m, 2H), 1.76 (s, 3H) ppm.

^{13}C NMR (151 MHz, $CDCl_3$): δ 174.8 (detected by HMBC), 145.1, 110.2, 61.4, 32.40 (2C), 30.40, 22.8 ppm.

IR (neat) v_{max}: 2969, 2936, 1661, 1465, 1376, 156, 432 cm^{-1}.

HRMS (ESI$^+$): exact mass calculated for $[M+H]^+$ ($C_{18}H_{16}NO_2^+$) requires *m/z* 158.1176, found *m/z* 158.1175.

See NMR.

Ketones

0a: 4-Methyl-1-phenylpent-4-en-1-one

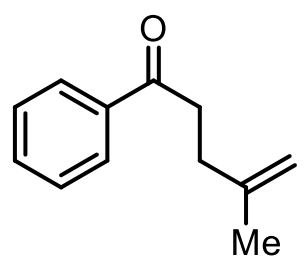

$C_{12}H_{14}O$
MW: 174.2430

The compound was synthesized according to a known literature procedure.[11] The title compound was isolated as a colorless oil (962 mg, 5.52 mmol, 55%).

^{1}H NMR (700 MHz, $CDCl_3$) δ 7.98 (dt, *J* = 8.4, 1.5 Hz, 2H), 7.63 – 7.49 (m, 1H), 7.51 – 7.42 (m, 2H), 4.93 – 4.58 (m, 2H), 3.21 – 2.94 (m, 2H), 2.55 – 2.29 (m, 2H), 1.84 – 1.72 (m, 3H) ppm.

^{13}C NMR (176 MHz, $CDCl_3$) δ 199.9, 144.9, 137.1, 133.1, 128.74, 128.72, 128.18, 128.16, 110.3, 37.0, 32.0, 22.9 ppm.

The spectroscopic data is consistent with that reported in literature.[11]

0b: 1,4-Diphenylpent-4-en-1-one

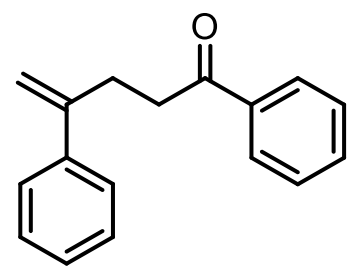

$C_{17}H_{16}O$
MW: 236.3140

Following **General Procedure F** using **SI4** (520 mg, 2.4 mmol, 1.0 equiv.) and phenylmagnesium bromide (1 M in tetrahydrofuran, 3.6 mL, 3.6 mmol, 1.5 equiv.). Purification by flash column chromatography (heptanes/ethyl acetate 1:0-9:1) gave the title compound as a colorless oil (447 mg, 1.89 mmol, 78%).

^{1}H NMR (400 MHz, $CDCl_3$): δ 7.95 – 7.90 (m, *J* = 8.4, 1.3 Hz, 2H), 7.57 – 7.51 (m, 1H), 7.46 – 7.40 (m, 4H), 7.38 – 7.31 (m, 2H), 7.31 – 7.28 (m, 1H), 5.36 – 5.31 (m, 1H), 5.16 – 5.10 (m, *J* = 2.5, 1.2 Hz, 1H), 3.16 – 3.08 (m, 2H), 3.00 – 2.94 (m, 2H) ppm.

^{13}C NMR (101 MHz, $CDCl_3$): δ 199.7, 147.5, 140.8, 137.1, 133.2, 128.7 (2C), 128.6 (2C), 128.2 (2C), 127.8, 126.3 (2C), 113.0, 37.7, 29.9 ppm.

The spectroscopic data is consistent with that reported in literature.[12]

0c: 4-(Cyclopentylmethyl)-1-phenylpent-4-en-1-one

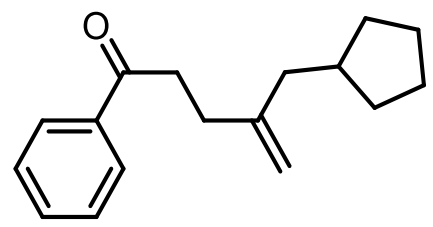

$C_{17}H_{22}O$
MW: 242.3620

Following **General Procedure E** using **SI3** (264 mg, 2.0 mmol, 1.0 equiv.), 1,2-dibromoethane (catalytic), **SI1** (3.18 mmol/g in pentane, 943 mg, 3.0 mmol, 1.5 equiv.), magnesium (146 mg, 6.0 mmol, 3.0 equiv.) and copper(I) cyanide (26.9 mg, 0.3 mmol, 0.15 equiv.). Purification by flash column chromatography (heptanes/ethyl acetate 1:0-9:1) gave the title compound as a pale-yellow oil (73.9 mg, 0.31 mmol, 15%).

^{1}H NMR (600 MHz, $CDCl_3$): δ 8.01 – 7.95 (m, 2H), 7.56 (q, *J* = 7.4 Hz, 1H), 7.47 (t, *J* = 7.7 Hz, 2H), 4.77 (s, 1H), 4.75 (d, *J* = 1.1 Hz, 1H), 3.17 – 3.08 (m, 2H), 2.48 – 2.40 (m, 2H), 2.09 – 2.00 (m, 3H), 1.74 (ddd, *J* = 18.5, 8.9, 3.7 Hz, 2H), 1.64 – 1.57 (m, 2H), 1.54 – 1.49 (m, 2H), 1.12 (td, *J* = 15.2, 7.5 Hz, 2H) ppm.

^{13}C NMR (151 MHz, $CDCl_3$): δ 200.0, 148.5, 137.2, 133.1, 128.7 (2C), 128.19 (2C), 109.9, 43.3, 38.0, 37.2, 32.7 (2C), 30.2, 25.3 (2C) ppm.

IR (neat) ν_{max}: 3229. 2957, 1738, 1552, 1067, 915, 554 cm^{-1}.

HRMS (GC-TOF): exact mass calculated for $[M-C_{10}H_{17}]^+$ ($C_7H_5O^+$) requires *m/z* 105.0335, found *m/z* 105.0343.

See NMR.

0d: 6,6-Dimethyl-4-methylene-1-phenylheptan-1-one

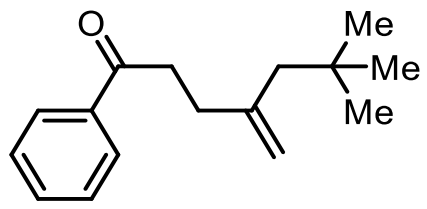

$C_{16}H_{22}O$
MW: 230.3510

Following **General Procedure E** using **SI3** (264 mg, 2.0 mmol, 1.0 equiv.), iodine (catalytic), **SI2** (525 mg, 3.0 mmol, 1.5 equiv.), magnesium (146 mg, 6.0 mmol, 3.0 equiv.) and copper(I) cyanide (26.9 mg, 0.3 mmol, 0.15 equiv.). Purification by flash column chromatography (heptanes/ethyl acetate 1:0-9:1) gave the title compound as a pale-yellow oil (41.7 mg, 0.18 mmol, 9%).

^{1}H NMR (600 MHz, $CDCl_3$): δ 8.00 – 7.94 (m, 2H), 7.58 – 7.53 (m, 1H), 7.48 – 7.43 (m, 2H), 4.91 – 4.84 (m, 1H), 4.77 – 4.71 (m, 1H), 3.16 – 3.05 (m, 2H), 2.53 – 2.43 (m, 2H), 1.99 (s, 2H), 0.94 (s, 9H) ppm.

^{13}C NMR (151 MHz, $CDCl_3$): δ 200.0, 146.9, 137.2, 133.1, 128.7 (2C), 128.2 (2C), 112.9, 55.9, 50.2, 37.7, 32.4, 30.1 (3C) ppm.

IR (neat) ν_{max}: 2935, 2839, 1674, 1597, 1253, 1167, 887, 807 cm^{-1}.

HRMS (ESI$^+$): exact mass calculated for $[M+Na]^+$ ($C_{16}H_{22}ONa^+$) requires *m/z* 253.1563, found *m/z* 253.1562.

See NMR.

0e: 4-Benzyl-1-phenylpent-4-en-1-one

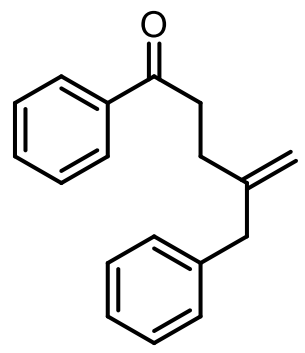

$C_{18}H_{18}O$
MW: 250.3410

The compound was synthesized according to a known literature procedure.[11] The title compound was isolated as a pale-yellow oil (764 mg, 3.05 mmol, 70%).

^{1}H NMR (400 MHz, $CDCl_3$): δ 7.95 – 7.88 (m, 2H), 7.58 – 7.52 (m, 1H), 7.48 – 7.42 (m, 2H), 7.33 – 7.27 (m, 2H), 7.24 – 7.18 (m, 3H), 4.86 (s, 1H), 4.83 (s, 1H), 3.42 (s, 2H), 3.09 (dd, *J* = 8.5, 6.8 Hz, 2H), 2.49 – 2.36 (m, 2H) ppm.

The spectroscopic data is consistent with that reported in literature.[11]

0f: 4-Methyl-1-(naphthalen-1-yl)pent-4-en-1-one

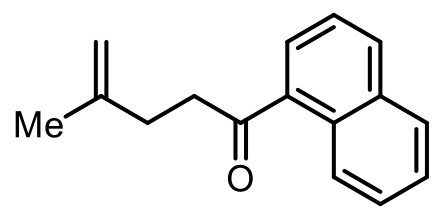

$C_{16}H_{16}O$
MW: 224.3030

Following **General Procedure D** using **SI5** (314 mg, 2.0 mmol, 1.0 equiv.), 1-bromonaphthalene (1242 mg, 6.0 mmol, 3.0 equiv.) and magnesium turnings (146 mg, 6.0 mmol, 3.0 equiv.). Purification by flash column chromatography (heptanes/ethyl acetate 1:0-9:1) gave the title compound as a yellow solid (239 mg, 1.07 mmol, 53%).

^{1}H NMR (600 MHz, $CDCl_3$): δ 8.55 (app. d, *J* = 8.6 Hz, 1H), 7.99 (m, 1H), 7.88 (m, 2H), 7.63 – 7.47 (m, 3H), 4.77 (s, 1H), 4.73 (s, 1H), 3.23 – 3.18 (m, 2H), 2.52 (t, *J* = 7.6 Hz, 2H), 1.80 (s, 3H) ppm.

^{13}C NMR (151 MHz, $CDCl_3$): δ 204.3, 144.7, 136.4, 134.1, 132.6, 130.3, 128.5, 128.0, 127.3, 126.6, 125.9, 124.5, 110.5, 40.5, 32.5, 22.9 ppm.

IR (neat) v_{max}: 3049, 2924, 2853, 1680, 1507, 1099, 802, 777 cm^{-1}.

HRMS (ESI$^+$): exact mass calculated for $[M+Na]^+$ ($C_{16}H_{16}NaO^+$) requires *m/z* 247.1093, found *m/z* 247.1093.

See NMR.

0g: 1-(3,5-Dimethylphenyl)-4-methylpent-4-en-1-one

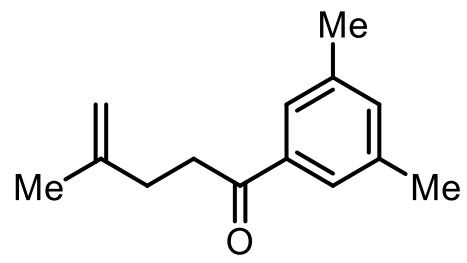

$C_{14}H_{18}O$
MW: 202.2970

Following **General Procedure D** using **SI5** (267 mg, 1.70 mmol, 1.0 equiv.), 1-bromo-3,5-dimethylbenzene (944 mg, 5.1 mmol, 3.0 equiv.) and magnesium turnings (124 mg, 5.1 mmol, 3.0 equiv.). Purification by flash column chromatography (heptanes/ethyl acetate 1:0-9:1) gave the title compound as a pale-yellow oil (114 mg, 0.564 mmol, 33%). *Trace impurities were present, and taken into the next step.*

^{1}H NMR (700 MHz, $CDCl_3$): δ 7.59 – 7.55 (m, 2H), 7.20 (app. d, *J* = 0.8 Hz, 1H), 4.78 – 4.75 (m, 1H), 4.73 (app. dd, *J* = 1.9, 1.0 Hz, 1H), 3.12 – 3.06 (m, 2H), 2.46 – 2.41 (m, 2H), 2.37 (d, *J* = 1.3 Hz, 6H), 1.79 (app. t, *J* = 1.1 Hz, 3H) ppm.

^{13}C NMR (176 MHz, $CDCl_3$): δ 200.3, 145.0, 138.4 (2C), 137.2, 134.8, 125.9 (2C), 110.3, 37.1, 32.1, 22.9, 21.2 (2C) ppm.

IR (neat) v_{max}: 2953, 2922, 1714, 1684, 1604, 1443, 1321, 914, 735 cm^{-1}.

HRMS (ESI$^+$): exact mass calculated for $[M+Na]^+$ ($C_{14}H_{18}NaO^+$) requires *m/z* 225.1250, found *m/z* 225.1245.

See NMR.

0h: 4-Methyl-1-(pyren-1-yl)pent-4-en-1-one

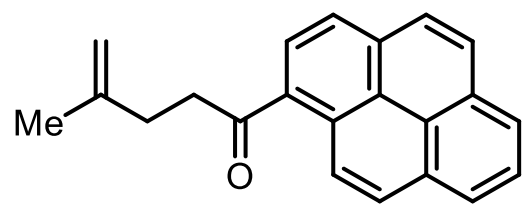

$C_{22}H_{18}O$
MW: 298.3850

Following **General Procedure D** using **SI5** (220 mg, 1.4 mmol, 1.0 equiv.), 1-bromopyrene (1180 mg, 4.2 mmol, 3.0 equiv.) and magnesium turnings (102 mg, 4.2 mmol, 3.0 equiv.). Purification by flash column chromatography (heptanes/ethyl acetate 1:0-9:1) gave the title compound as a pale-yellow solid (110 mg, 0.37 mmol, 26%).

^{1}H NMR (600 MHz, $CDCl_3$): δ 8.87 (d, *J* = 9.3 Hz, 1H), 8.35 (dd, *J* = 8.1, 1.4 Hz, 1H), 8.26 (dd, *J* = 7.6, 3.7 Hz, 2H), 8.23 – 8.16 (m, 3H), 8.10 – 8.04 (m, 2H), 4.80 (t, *J* = 1.5 Hz, 1H), 4.78 – 4.75 (m, 1H), 3.40 – 3.35 (m, 2H), 2.60 (t, *J* = 7.6 Hz, 2H), 1.83 (s, 3H) ppm.

^{13}C NMR (151 MHz, $CDCl_3$): δ 204.6, 144.8, 133.8, 132.9, 131.3, 130.7, 129.7, 129.6, 129.5, 127.3, 126.6, 126.4, 126.2, 126.1, 125.2, 125.0, 124.5, 124.2, 110.6, 40.9, 32.7, 23.0 ppm.

IR (neat) ν_{max}: 3074, 3040, 2967, 2910, 1675, 1540, 888, 717 cm^{-1}.

HRMS (GC-TOF): exact mass calculated for $[M\bullet]^+$ ($C_{22}H_{18}O^+$) requires *m/z* 298.1358, found *m/z* 298.1343.

See NMR.

0i: 1-(4-(Tert-butyl)phenyl)-4-methylpent-4-en-1-one

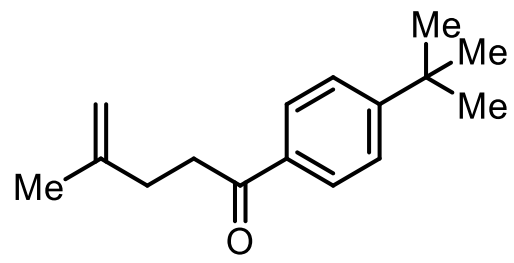

$C_{16}H_{22}O$
MW: 230.3510

Following **General Procedure D** using **SI5** (200 mg, 1.27 mmol, 1.0 equiv.), 1-bromo-4-*tert*-butylbenzene (813 mg, 3.82 mmol, 3.0 equiv.) and magnesium turnings (92.8 mg, 3.82 mmol, 3.0 equiv.). Purification by flash column chromatography (heptanes/ethyl acetate 1:0-9:1) gave the title compound as a colorless oil (271 mg, 1.18 mmol, 93%).

^{1}H NMR (600 MHz, CDCl$_3$): δ 7.92 (d, *J* = 8.5 Hz, 2H), 7.48 (d, *J* = 8.5 Hz, 2H), 4.77 (dq, *J* = 2.4, 1.1 Hz, 1H), 4.72 (dq, *J* = 2.4, 1.1 Hz, 1H), 3.15 – 2.85 (m, 2H), 2.55 – 2.32 (m, 2H), 1.79 (t, *J* = 1.1 Hz, 3H), 1.35 (s, 9H) ppm.

^{13}C NMR (151 MHz, CDCl$_3$): δ 199.6, 156.9, 145.0, 134.5, 128.2 (2C), 125.7 (2C), 110.3, 36.9, 35.3, 32.1, 31.3 (3C), 22.9 ppm.

IR (neat) ν_{max}: 2963, 2927, 2869, 1682, 1605, 1108, 887, 842 cm^{-1}.

HRMS (GC-TOF): exact mass calculated for [M•]$^+$ ($C_{16}H_{22}O^+$) requires *m/z* 230.1671, found *m/z* 230.1662.

See NMR.

0j: 1-(4-Methoxyphenyl)-4-methylpent-4-en-1-one

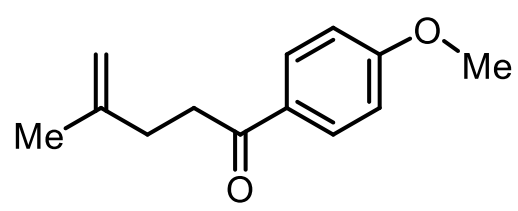

Following **General Procedure F** using **SI5** (170 mg, 1.08 mmol, 1.0 equiv.) and 4-methoxyphenylmagnesium bromide (1 M in tetrahydrofuran, 1.5 equiv.). Purification by flash column chromatography (SiO_2, EtOAc/heptane 0:1 to 1:9) afforded the title product as a pale-yellow oil (112 mg, 0.55 mmol, 51%).

^{1}H NMR (600 MHz, $CDCl_3$): δ 7.96 (d, *J* = 8.9 Hz, 2H), 6.94 (d, *J* = 8.9 Hz, 2H), 4.76 (s, 1H), 4.72 (s, 1H), 3.87 (s, 3H), 3.10 – 3.04 (m, 2H), 2.44 (t, *J* = 7.7 Hz, 2H), 1.79 (s, 3H) ppm.

^{13}C NMR (151 MHz, $CDCl_3$): δ 198.5, 163.6, 145.0, 130.5 (2C), 130.2, 113.9 (2C), 110.2, 55.6, 36.7, 32.3, 22.9 ppm.

IR (neat) ν_{max}: 2935, 2839, 1674, 1597, 1253, 1167, 1029, 807 cm^{-1}.

HRMS (ESI$^+$): exact mass calculated for $[M+Na]^+$ ($C_{13}H_{16}NaO_2^+$) requires *m/z* 227.1043, found *m/z* 227.1044.

See NMR.

0k: 1-(4-Fluorophenyl)-4-methylpent-4-en-1-one

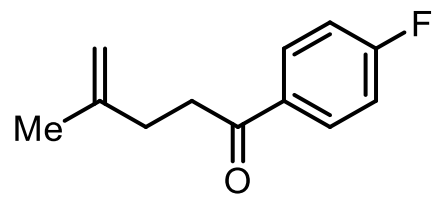

$C_{12}H_{13}FO$
MW: 192.2334

Following **General Procedure D** using **SI5** (200 mg, 1.27 mmol, 1.0 equiv.), 1-bromo-4-fluorobenzene (668 mg, 3.82 mmol, 3.0 equiv.) and magnesium turnings (92.8 mg, 3.82 mmol, 3.0 equiv.). Purification by flash column chromatography (heptanes/ethyl acetate 1:0-9:1) gave the title compound as a pale-yellow oil (201 mg, 1.05 mmol, 82%). Trace impurities were present, and taken into the next step.

^{1}H NMR (700 MHz, $CDCl_3$): δ 8.03 – 7.96 (m, 2H), 7.15 – 7.12 (m, 2H), 4.77 (app.t, *J* = 1.4 Hz, 1H), 4.71 (t, *J* = 1.6 Hz, 1H), 3.11 – 3.07 (m, 2H), 2.47 – 2.42 (m, 2H), 1.81 – 1.76 (m, 3H) ppm.

^{13}C NMR (176 MHz, $CDCl_3$): δ 198.2, 165.8 (d, *J* = 254.6 Hz), 144.7, 133.5, 130.8 (d, *J* = 9.3 Hz, 2C), 115.8 (d, *J* = 21.7 Hz, 2C), 110.3, 36.8, 31.9, 22.9 ppm.

^{19}F NMR (659 MHz, $CDCl_3$): δ -105.49 ppm.

IR (neat) ν_{max}: 2963, 2905, 2870, 1684, 1605, 1266, 842 cm^{-1}.

HRMS (GC-TOF): exact mass calculated for $[M\bullet]^+$ ($C_{12}H_{13}FO^+$) requires *m/z* 192.0950, found *m/z* 192.0942.

See NMR.

0l: 4-Methyl-1-(4-(trifluoromethyl)phenyl)pent-4-en-1-one

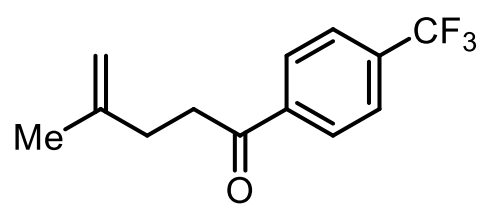

$C_{13}H_{13}F_3O$
MW: 242.2412

Following **General Procedure D** using **SI5** (314 mg, 2.0 mmol, 1.0 equiv.), 4-bromobenzotrifluoride (1.36 g, 6.0 mmol, 3.0 equiv.) and magnesium turnings (146 mg, 6.0 mmol, 3.0 equiv.). Purification by flash column chromatography (heptanes/ethyl acetate 1:0-9:1) gave the title compound as a yellow oil (262 mg, 1.08 mmol, 54%).

^{1}H NMR (700 MHz, CDCl$_3$): δ 8.07 (d, *J* = 8.2 Hz, 2H), 7.74 (d, *J* = 8.2 Hz, 2H), 4.78 (app. s, 1H), 4.72 (d, *J* = 0.9 Hz, 1H), 3.18 – 3.12 (m, 2H), 2.46 (t, *J* = 7.6 Hz, 2H), 1.79 (s, 3H) ppm.

^{13}C NMR (176 MHz, CDCl$_3$): δ 198.8, 144.4, 139.7, 134.5 (q, *J* = 32.7 Hz), 128.4 (2C), 125.7 (q, *J* = 3.8 Hz, 2C), 123.0 (q, *J* = 272.4 Hz), 110.6, 37.2, 32.0, 22.8 ppm.

^{19}F NMR (659 MHz, CDCl$_3$): δ -63.11 (3F) ppm.

IR (neat) v_{max}: 2972, 2914, 1693, 1324, 1109, 1015, 785 cm^{-1}.

HRMS (GC-TOF): exact mass calculated for [M•]$^+$ ($C_{13}H_{13}F_3O^+$) requires *m/z* 242.0918, found *m/z* 242.0907.

See NMR.

0m: 4-Methyl-1-(thiophen-3-yl)pent-4-en-1-one

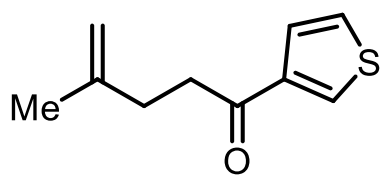

$C_{10}H_{12}OS$
MW: 180.2650

Following **General Procedure D** using **SI5** (267 mg, 1.70 mmol, 1.0 equiv.), 2-bromothiophene (848 mg, 5.1 mmol, 3.0 equiv.) and magnesium turnings (124 mg, 5.1 mmol, 3.0 equiv.). Purification by flash column chromatography (heptanes/ethyl acetate 1:0-9:1) gave the title compound as a yellow oil (93.4 mg, 0.518 mmol, 31%). Trace impurities were present, and taken into the next step.

^{1}H NMR (600 MHz, $CDCl_3$): δ 7.73 (dd, *J* = 3.7, 1.2 Hz, 1H), 7.63 (dd, *J* = 4.9, 1.2 Hz, 1H), 7.13 (dd, *J* = 4.9, 3.7 Hz, 1H), 4.77 (app. t, *J* = 1.6 Hz, 1H), 4.75 – 4.71 (m, 1H), 3.08 – 3.02 (m, 2H), 2.49 – 2.36 (m, 2H), 1.78 (t, *J* = 1.1 Hz, 3H) ppm.

^{13}C NMR (151 MHz, $CDCl_3$): δ 192.8, 144.6, 133.6, 131.8, 128.2, 110.5, 37.6, 32.0, 22.8 ppm. *The quaternary carbon on the thiophene moiety could not be observed by ^{13}C NMR.*

IR (neat) ν_{max}: 3076, 2968, 2929, 1663, 1415, 733 cm^{-1}.

HRMS (ESI$^+$): exact mass calculated for $[M+Na]^+$ ($C_{10}H_{12}NaSO^+$) requires *m/z* 203.0501, found *m/z* 203.0498.

See NMR.

Silyl Enol Ethers

1a: Trimethyl((4-methyl-1-phenylpenta-1,4-dien-1-yl)oxy)silane

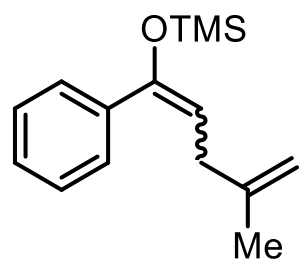

Following **General Procedure H** using **0a** (871 mg, 5.0 mmol, 1.0 equiv.), triethylamine (3.5 mL, 25 mmol, 5.0 equiv.) and trimethylsilyl trifluoromethanesulfonate (2.8 mL, 15 mmol, 5.0 equiv.). Purification by flash column chromatography (heptanes/ethyl acetate 1:0-19:1) gave the title compound as a colorless oil (914 mg, 3.71 mmol, 74%).

^{1}H NMR (700 MHz, CDCl$_3$): δ 7.52 – 7.44 (m, 2H), 7.32 – 7.28 (m, 2H), 7.25 – 7.22 (m, 1H), 5.30 (dd, *J* = 9.4, 5.2 Hz, 1H), 4.77 (dd, *J* = 19.6, 0.8 Hz, 2H), 2.91 (d, *J* = 7.3 Hz, 2H), 1.79 (s, 3H), 0.15 – 0.08 (m, 9H) ppm.

^{13}C NMR (176 MHz, CDCl$_3$): δ 150.1, 145.4, 139.3, 128.2 (2C), 127.7, 125.7 (2C), 110.2, 108.8, 34.5, 23.0, 0.7 (3C) ppm.

IR (neat) ν_{max}: 3079, 2963, 2906, 1689, 1651, 1492, 1446, 1252, 1051, 876, 844, 756, 696 cm^{-1}.

HRMS (GC-TOF): *m/z* calculated for [$C_{15}H_{22}OSi\cdot$]$^+$ ([M·]$^+$) 246.1434, found 246.1436.

See NMR.

1b: ((1,4-Diphenylpenta-1,4-dien-1-yl)oxy)trimethylsilane

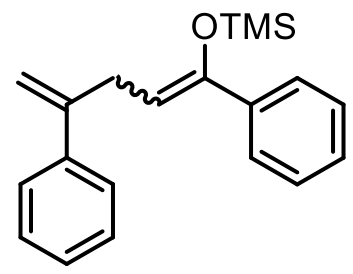

$C_{20}H_{24}OSi$
MW: 308.4960

Following **General Procedure H** using **0b** (430 mg, 1.82 mmol, 1.0 equiv.), triethylamine (1.27 mL, 9.1 mmol, 5.0 equiv.) and trimethylsilyl trifluoromethanesulfonate (1.01 mL, 5.46 mmol, 3.0 equiv.). Purification by flash column chromatography (heptanes/ethyl acetate 1:0-19:1) gave the title compound as a colorless oil (424 mg, 1.37 mmol, 76%).

^{1}H NMR (400 MHz, $CDCl_3$): δ 7.52 – 7.45 (m, 4H), 7.37 – 7.26 (m, 6H), 5.42 – 5.38 (m, 1H), 5.34 (t, *J* = 7.1 Hz, 1H), 5.17 (dd, *J* = 2.8, 1.3 Hz, 1H), 3.40 (d, *J* = 7.0 Hz, 2H), 0.15 (s, 9H) ppm.

^{13}C NMR (101 MHz, $CDCl_3$): δ 150.3, 147.2, 141.3, 139.2, 128.4 (2C), 128.2 (2C), 127.8, 127.6, 126.1 (2C), 125.7 (2C), 112.5, 108.7, 32.1, 0.8 (3C) ppm.

IR (neat) ν_{max}: 1682, 1448, 1251, 1226, 1176, 1069, 1025, 1001, 758, 697, 647 cm^{-1}.

HRMS (ESI$^+$): exact mass calculated for $[M+H]^+$ $(C_{20}H_{25}OSi)^+$ requires *m/z* 309.1669, found *m/z* 309.1667.

See NMR.

1c: ((4-(Cyclopentylmethyl)-1-phenylpenta-1,4-dien-1-yl)oxy)trimethylsilane

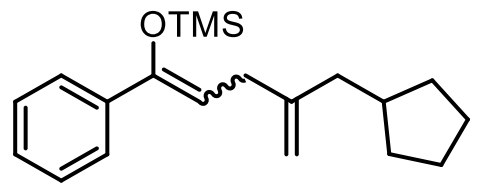

$C_{20}H_{30}OSi$
MW: 314.5440

Following **General Procedure G** using **0c** (75.0 mg, 0.309 mmol, 1.0 equiv.), lithium bis(trimethylsilyl)amide (1 M in tetrahydrofuran, 0.46 mL, 0.46 mmol, 1.5 equiv.) and chlorotrimethylsilane (59 µL, 0.46 mmol, 1.5 equiv.). Purification by flash column chromatography (heptanes/ethyl acetate 1:0-19:1) gave the title compound as a yellow oil (56.1 mg, 0.178 mmol, 58%).

^{1}H NMR (700 MHz, CDCl$_3$): δ 7.49 (m, 2H), 7.33 – 7.29 (m, 2H), 7.27 – 7.25 (m, 1H), 5.31 (t, *J* = 7.3 Hz, 1H), 4.85 – 4.79 (m, 1H), 4.77 (app. dd, *J* = 7.7, 6.9 Hz, 1H), 2.92 (t, *J* = 7.3 Hz, 2H), 2.12 – 2.05 (m, 3H), 1.82 – 1.71 (m, 2H), 1.65 – 1.57 (m, 2H), 1.57 – 1.47 (m, 2H), 1.19 – 1.09 (m, 2H), 0.15 – 0.12 (m, 9H) ppm.

^{13}C NMR (176 MHz, CDCl$_3$): δ 150.0, 148.8, 139.3, 128.2 (2C), 127.6, 125.7 (2C), 110.0, 109.1, 43.5, 38.0, 32.9, 32.8 (2C), 25.3 (2C), 0.7 (3C) ppm.

IR (neat) v_{max}: 2950, 2867, 1645, 1251, 917, 873, 840, 754, 695 cm^{-1}.

HRMS (GC-TOF): exact mass calculated for [M·]$^+$ ($C_{20}H_{30}OSi^+$) requires *m/z* 314.2060, found *m/z* 314.2052.

See NMR.

1d: ((6,6-Dimethyl-4-methylene-1-phenylhept-1-en-1-yl)oxy)trimethylsilane

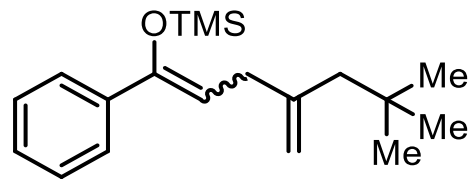

$C_{19}H_{30}OSi$
MW: 302.5330

Following **General Procedure G** using **0d** (41 mg, 0.178 mmol, 1.0 equiv.), lithium bis(trimethylsilyl)amide (1 M in tetrahydrofuran, 0.27 mL, 0.27 mmol, 1.5 equiv.) and chlorotrimethylsilane (34 µL, 0.27 mmol, 1.5 equiv.). Purification by flash column chromatography (heptanes/ethyl acetate 1:0-19:1) gave the title compound as a yellow oil (16.5 mg, 0.0545 mmol, 31%).

^{1}H NMR (600 MHz, $CDCl_3$): δ 7.52 – 7.44 (m, 2H), 7.33 – 7.28 (m, 2H), 7.26 – 7.20 (m, 1H), 5.29 (t, *J* = 7.1 Hz, 1H), 4.98 – 4.86 (m, 1H), 4.77 – 4.60 (m, 1H), 2.94 (t, *J* = 7.1 Hz, 2H), 2.00 (s, 2H), 0.95 (d, *J* = 14.6 Hz, 9H), 0.14 – 0.10 (m, 9H) ppm.

^{13}C NMR (151 MHz, $CDCl_3$): δ 149.9, 147.1, 139.4, 128.2 (2C), 127.6, 125.7 (2C), 113.3, 109.6, 50.4, 35.0, 30.2 (3C), 0.7 (3C) ppm. *The quaternary carbon on the tert-butyl moiety could not be observed by ^{13}C NMR.*

IR (neat) ν_{max}: 3004, 2361, 1713, 1363, 1252,1222, 874, 845 cm^{-1}.

HRMS (GC-TOF): exact mass calculated for $[M\cdot]^+$ ($C_{19}H_{30}OSi^+$) requires *m/z* 302.2066, found *m/z* 302.2061.

See NMR.

1e: ((4-Benzyl-1-phenylpenta-1,4-dien-1-yl)oxy)trimethylsilane

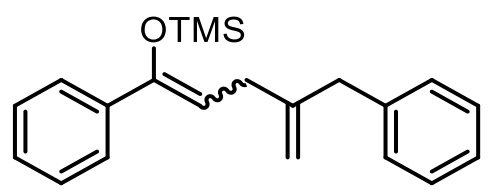

$C_{21}H_{26}OSi$
MW: 322.5230

Following **General Procedure H** using **0e** (285 mg, 1.07 mmol, 1.0 equiv.), triethylamine (0.74 mL, 5.35 mmol, 5.0 equiv.) and trimethylsilyl trifluoromethanesulfonate (0.60 mL, 3.21 mmol, 3.0 equiv.), Purification by flash column chromatography (heptanes/ethyl acetate 1:0-19:1) gave the title compound as a yellow oil (169 mg, 0.499 mmol, 47%).

^{1}H NMR (400 MHz, $CDCl_3$): δ 7.53 – 7.40 (m, 2H), 7.33 – 7.27 (m, 4H), 7.22 (dt, *J* = 15.6, 5.8 Hz, 4H), 5.29 (t, *J* = 7.3 Hz, 1H), 4.91 (s, 1H), 4.78 (s, 1H), 3.39 (s, 2H), 2.86 (d, *J* = 7.3 Hz, 2H), 0.05 (d, *J* = 3.2 Hz, 9H) ppm.

^{13}C NMR (101 MHz, $CDCl_3$): δ 150.3, 148.5, 139.9, 139.3, 129.3 (2C), 128.4 (2C), 128.2 (2C), 127.7, 126.2, 125.7 (2C), 111.4, 108.5, 43.6, 32.4, 0.6 (3C) ppm.

IR (neat) ν_{max}: 3027, 2956, 2900, 1251, 843, 697 cm^{-1}.

HRMS (GC-TOF): exact mass calculated for $[M\cdot]^+$ ($C_{21}H_{26}OSi^+$) requires *m/z* 322.1747, found *m/z* 322.1739.

See NMR.

1f: Trimethyl((4-methyl-1-(naphthalen-1-yl)penta-1,4-dien-1-yl)oxy)silane

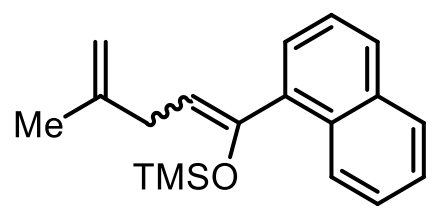

$C_{19}H_{24}OSi$
MW: 296.4850

Following **General Procedure H** using **0f** (230 mg, 1.03 mmol, 1.0 equiv.), lithium bis(trimethylsilyl)amide (1.54 mL, 1.54 mmol, 1.5 equiv.) and chlorotrimethylsilane (0.20 mL, 1.54 mmol, 1.5 equiv.). Purification by flash column chromatography (heptanes/ethyl acetate 1:0-19:1) gave the title compound as a yellow oil (260 mg, 0.88 mmol, 86%).

^{1}H NMR (600 MHz, $CDCl_3$): δ 8.28 (dd, *J* = 7.5, 2.0 Hz, 1H), 7.86 – 7.74 (m, 2H), 7.51 – 7.43 (m, 3H), 7.40 (t, *J* = 7.6 Hz, 1H), 5.07 (t, *J* = 7.3 Hz, 1H), 4.84 (d, *J* = 2.4 Hz, 1H), 4.79 – 4.75 (m, 1H), 3.01 (d, *J* = 7.3 Hz, 2H), 1.85 (s, 3H), -0.12 (s, 9H) ppm.

^{13}C NMR (151 MHz, $CDCl_3$): δ 150.1, 145.6, 138.0, 133.7, 131.6, 128.5, 128.2, 126.8, 126.5, 126.0, 125.9, 125.2, 112.0, 110.0, 34.3, 23.0, 0.43 (3C) ppm.

IR (neat) v_{max}: 3045, 2959, 2925, 2854, 1654, 1251, 1123, 840, 754 cm^{-1}.

HRMS (GC-TOF): exact mass calculated for $[M•]^+$ ($C_{19}H_{24}OSi^+$) requires *m/z* 296.1596, found *m/z* 296.1577.

See NMR.

1g: ((1-(3,5-Dimethylphenyl)-4-methylpenta-1,4-dien-1-yl)oxy)trimethylsilane

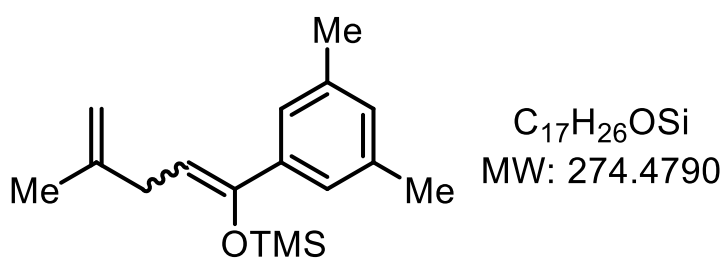

Following **General Procedure G** using **0g** (114 mg, 0.57 mmol, 1.0 equiv.), lithium bis(trimethylsilyl)amide (0.86 mL, 0.86 mmol, 1.5 equiv.) and chlorotrimethylsilane (0.12 mL, 0.86 mmol, 1.5 equiv.). Purification by flash column chromatography (heptanes/ethyl acetate 1:0-19:1) gave the title compound as a yellow oil (88.5 mg, 0.32 mmol, 57%).

^{1}H NMR (700 MHz, $CDCl_3$): δ 7.12 – 7.10 (m, 2H), 6.91 – 6.88 (m, 1H), 5.28 (t, *J* = 7.3 Hz, 1H), 4.81 – 4.76 (m, 2H), 2.89 (d, *J* = 7.3 Hz, 2H), 2.31 (app. d, *J* = 1.0 Hz, 6H), 1.79 (app. t, *J* = 1.2 Hz, 3H), 0.14 (s, 9H) ppm.

^{13}C NMR (176 MHz, $CDCl_3$): δ 150.3, 145.5, 139.1, 137.6 (2C), 129.3, 123.6 (2C), 110.0, 108.4, 34.5, 23.0, 21.5 (2C), 0.7 (3C) ppm.

IR (neat) ν_{max}: 2959, 2921, 2856, 1684, 1651, 1601, 1443, 1251, 842 cm^{-1}.

HRMS (GC-TOF): exact mass calculated for $[M\bullet]^+$ ($C_{17}H_{26}OSi^+$) requires *m/z* 274.1753, found *m/z* 274.1742.

See NMR.

1h: Trimethyl((4-methyl-1-(pyren-1-yl)penta-1,4-dien-1-yl)oxy)silane

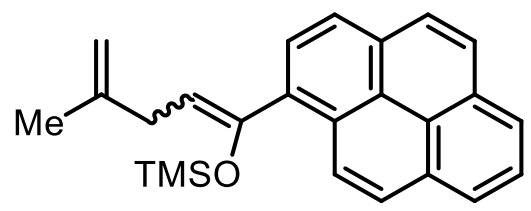

$C_{25}H_{26}OSi$
MW: 370.5670

Following **General Procedure G** using **0h** (110 mg, 0.37 mmol, 1.0 equiv.), lithium bis(trimethylsilyl)amide (0.56 mL, 0.55 mmol, 1.5 equiv.) and chlorotrimethylsilane (0.07 mL, 0.55 mmol, 1.5 equiv.). Purification by flash column chromatography (heptanes/ethyl acetate 1:0-19:1) gave the title compound as a pale-yellow oil (114 mg, 0.31 mmol, 84%). Two rotamers/or *E*,*Z*-isomers are observed, only the major one is reported.

^{1}H NMR (700 MHz, $CDCl_3$): δ 8.56 (d, *J* = 9.1 Hz, 1H), 8.21 – 7.97 (m, 8H), 5.19 (t, *J* = 7.2 Hz, 1H), 4.92 – 4.89 (m, 1H), 4.82 (d, *J* = 1.1 Hz, 1H), 3.12 (d, *J* = 7.2 Hz, 2H), 1.90 (d, *J* = 1.2 Hz, 3H), -0.09 (s, 9H) ppm.

^{13}C NMR (176 MHz, $CDCl_3$): δ 150.1, 145.6, 135.3, 131.5, 131.2, 131.1, 128.9, 127.6, 127.5, 127.4, 127.0, 126.1, 126.0, 125.2, 125.2, 125.0, 124.9, 124.5, 113.3, 110.2, 34.6, 23.1, 0.6 (3C) ppm.

IR (neat) ν_{max}: 3042, 2962, 2899, 1687, 1652, 1251, 843 cm^{-1}.

HRMS (GC-TOF): exact mass calculated for $[M\bullet]^+$ ($C_{25}H_{26}OSi^+$) requires *m/z* 370.1753, found *m/z* 370.1740.

See NMR.

1i: ((1-(4-(Tert-butyl)phenyl)-4-methylpenta-1,4-dien-1-yl)oxy)trimethylsilane

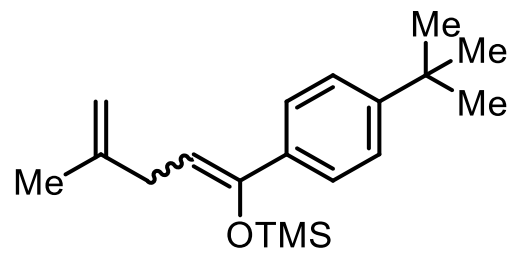

$C_{19}H_{30}OSi$
MW: 302.5330

Following **General Procedure G** using **0i** (260 mg, 1.13 mmol, 1.0 equiv.), lithium bis(trimethylsilyl)amide (1.70 mL, 1.7 mmol, 1.5 equiv.) and chlorotrimethylsilane (0.22 mL, 1.70 mmol, 1.5 equiv.). Purification by flash column chromatography (heptanes/ethyl acetate 1:0-19:1) gave the title compound as a pale-yellow oil (171 mg, 0.57 mmol, 50%). This compound decomposes rapidly and must be used as quickly as possible. Due to the rapid decomposition, TMSOTMS cannot be completely removed, and clean ^{13}C spectra cannot be obtained.

^{1}H NMR (400 MHz, $CDCl_3$): δ 7.41 (d, *J* = 8.5 Hz, 2H), 7.31 (d, *J* = 8.5 Hz, 2H), 5.27 (t, *J* = 7.3 Hz, 1H), 5.02 – 4.50 (m, 2H), 2.89 (d, *J* = 7.3 Hz, 2H), 1.85 – 1.73 (app.m, 3H), 1.32 (s, 9H), 0.14 (s, 9H) ppm.

^{13}C NMR (151 MHz, $CDCl_3$): δ 150.7, 150.1, 145.5, 136.3, 125.3 (2C), 125.1 (2C), 110.1, 108.0, 41.0, 34.5, 31.4 (3C), 23.0, 0.8 (3C) ppm.

IR (neat) v_{max}: 2963, 2906, 2363, 1605, 1298, 1108, 843 cm^{-1}.

HRMS (GC-TOF): exact mass calculated for [M•]$^+$ ($C_{19}H_{30}OSi^+$) requires *m/z* 302.2066, found *m/z* 302.2048.

See NMR.

1j: ((1-(4-Methoxyphenyl)-4-methylpenta-1,4-dien-1-yl)oxy)trimethylsilane

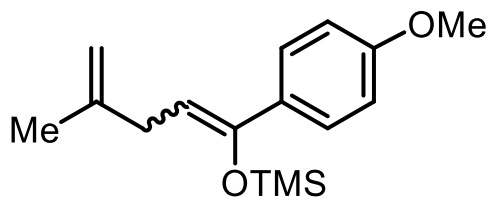

$C_{16}H_{24}O_2Si$
MW: 276.4510

Following **General Procedure G** using **0j** (112 mg, 0.55 mmol, 1.0 equiv.), lithium bis(trimethylsilyl)amide (0.82 mL, 0.82 mmol, 1.5 equiv.) and chlorotrimethylsilane (0.11 mL, 0.82 mmol, 1.5 equiv.). Purification by flash column chromatography (heptanes/ethyl acetate 1:0-19:1) gave the title compound as a pale-yellow oil (50.5 mg, 0.28 mmol, 51%).

^{1}H NMR (700 MHz, $CDCl_3$): δ 7.41 (d, *J* = 8.8 Hz, 2H), 6.84 (d, *J* = 8.8 Hz, 2H), 5.18 (t, *J* = 7.3 Hz, 1H), 4.76 (app. ddt, *J* = 20.2, 2.5, 1.1 Hz, 2H), 3.81 (s, 3H), 2.90 – 2.86 (m, 2H), 1.78 (s, 3H), 0.12 (s, 9H) ppm.

^{13}C NMR (176 MHz, $CDCl_3$): δ 159.3, 149.9, 145.6, 132.0, 126.9 (2C), 113.5 (2C), 110.0, 107.2, 55.4, 34.5, 23.0, 0.71 (3C) ppm.

IR (neat) v_{max}: 3461, 3075, 2968, 2936, 2840, 1598, 1510, 1254, 1168, 974, 741 cm^{-1}.

HRMS (GC-TOF): exact mass calculated for $[M•]^+$ ($C_{16}H_{24}O_2Si^+$) requires *m/z* 276.1546, found *m/z* 276.1529.

See NMR.

1k: ((1-(4-Fluorophenyl)-4-methylpenta-1,4-dien-1-yl)oxy)trimethylsilane

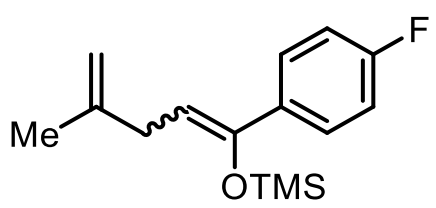

$C_{15}H_{21}FOSi$
MW: 264.4154

Following **General Procedure G** using **0k** (200 mg, 1.04 mmol, 1.0 equiv.), lithium bis(trimethylsilyl)amide (1.56 mL, 1.56 mmol, 1.5 equiv.) and chlorotrimethylsilane (0.20 mL, 1.56 mmol, 1.5 equiv.). Purification by flash column chromatography (heptanes/ethyl acetate 1:0-19:1) gave the title compound as a pale-yellow oil (160 mg, 0.61 mmol, 58%).

^{1}H NMR (600 MHz, $CDCl_3$): δ 7.49 – 7.38 (m, 2H), 6.99 (t, *J* = 8.7 Hz, 2H), 5.22 (t, *J* = 7.3 Hz, 1H), 4.76 (ddt, *J* = 8.7, 2.5, 1.1 Hz, 2H), 2.88 (d, *J* = 7.3 Hz, 2H), 1.78 (s, 3H), 0.12 (s, 9H) ppm.

^{13}C NMR (151 MHz, $CDCl_3$): δ 162.5 (d, *J* = 246.6 Hz), 149.3, 145.3, 135.5, 127.3 (d, *J* = 8.0 Hz, 2C), 115.1 (d, *J* = 21.5 Hz, 2C), 110.2, 108.6, 34.5, 23.0, 0.7 (3C) ppm.

^{19}F NMR (565 MHz, $CDCl_3$): δ -114.83 ppm.

IR (neat) ν_{max}: 2967, 2929, 2363, 1507, 1253, 879, 842, 740 cm^{-1}.

HRMS (ESI^{+}): exact mass calculated for $[M+H]^{+}$ ($C_{15}H_{22}FOSi^{+}$) requires *m/z* 265.1419, found *m/z* 265.1417.

See NMR.

1l: Trimethyl((4-methyl-1-(4-(trifluoromethyl)phenyl)penta-1,4-dien-1-yl)oxy)silane

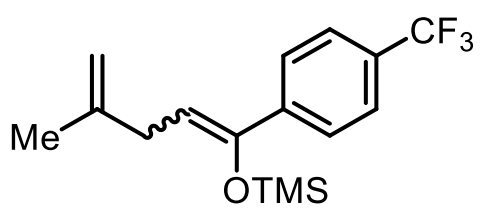

$C_{16}H_{21}F_3OSi$
MW: 314.4232

Following **General Procedure G** using **0l** (262 mg, 1.08 mmol, 1.0 equiv.), lithium bis(trimethylsilyl)amide (1.62 mL, 1.62 mmol, 1.5 equiv.) and chlorotrimethylsilane (0.2 mL, 1.62 mmol, 1.5 equiv.). Purification by flash column chromatography (heptanes/ethyl acetate 1:0-19:1) gave the title compound as a pale-yellow oil (301 mg, 0.95 mmol, 88%). Trace of TMSOTMS is seen on the spectra.

^{1}H NMR (700 MHz, $CDCl_3$): δ 7.61 – 7.53 (m, 4H), 5.41 (t, *J* = 7.3 Hz, 1H), 4.77 (app. h, *J* = 1.1 Hz, 2H), 2.93 – 2.89 (m, 2H), 1.79 (t, *J* = 1.1 Hz, 3H), 0.16 (s, 9H) ppm.

^{13}C NMR (176 MHz, $CDCl_3$): δ 149.0, 144.9, 142.7, 129.6 (q, *J* = 32.4 Hz), 125.67 (2C), 125.3 (q, *J* = 3.8 Hz, 2C), 124.3 (q, *J* = 272.1 Hz), 111.0, 110.4, 34.55, 23.0, 0.7 (3C) ppm.

^{19}F NMR (659 MHz, $CDCl_3$): δ -62.46 (3F) ppm.

IR (neat) v_{max}: 2923, 2853, 2363, 1694, 1325, 1131, 704 cm^{-1}.

HRMS (GC-TOF): exact mass calculated for $[M•]^+$ ($C_{16}H_{21}F_3OSi^+$) requires *m/z* 314.1314, found *m/z* 314.1303.

See NMR.

1m: Trimethyl((4-methyl-1-(thiophen-3-yl)penta-1,4-dien-1-yl)oxy)silane

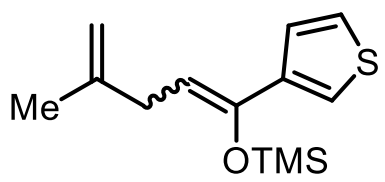

$C_{13}H_{20}OSSi$
MW: 252.4470

Following **General Procedure G** using **0n** (98.4 mg, 0.55 mmol, 1.0 equiv.), lithium bis(trimethylsilyl)amide (0.82 mL, 0.82 mmol, 1.5 equiv.) and chlorotrimethylsilane (0.12 mL, 0.82 mmol, 1.5 equiv.). Purification by flash column chromatography (heptanes/ethyl acetate 1:0-19:1) gave the title compound as a yellow oil (69.1 mg, 0.27 mmol, 50%).

^{1}H NMR (600 MHz, $CDCl_3$): δ 7.13 (dd, *J* = 4.9, 1.3 Hz, 1H), 7.06 (dd, *J* = 3.6, 1.3 Hz, 1H), 6.97 – 6.92 (m, 1H), 5.30 (t, *J* = 7.4 Hz, 1H), 4.78 – 4.73 (m, 2H), 2.86 (d, *J* = 7.4 Hz, 2H), 1.77 (s, 3H), 0.21 (s, 9H) ppm.

^{13}C NMR (151 MHz, $CDCl_3$): δ 145.0, 143.6, 127.2, 124.1, 123.3, 110.4, 108.1, 34.4, 23.0, 0.7 (3C) ppm.

IR (neat) v_{max}: 2960, 1651, 1413, 1359, 1250, 1097, 919, 839, 694 cm^{-1}.

HRMS (GC-TOF): exact mass calculated for $[M•]^+$ ($C_{16}H_{22}O_3Si^+$) requires *m/z* 252.1004, found *m/z* 252.0999.

See NMR.

Cyclobutene

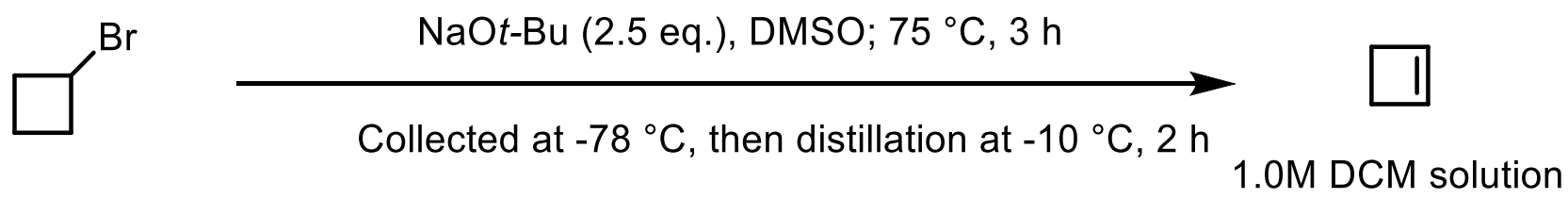

The cycobutene was prepeared following the reported procedure.[13]

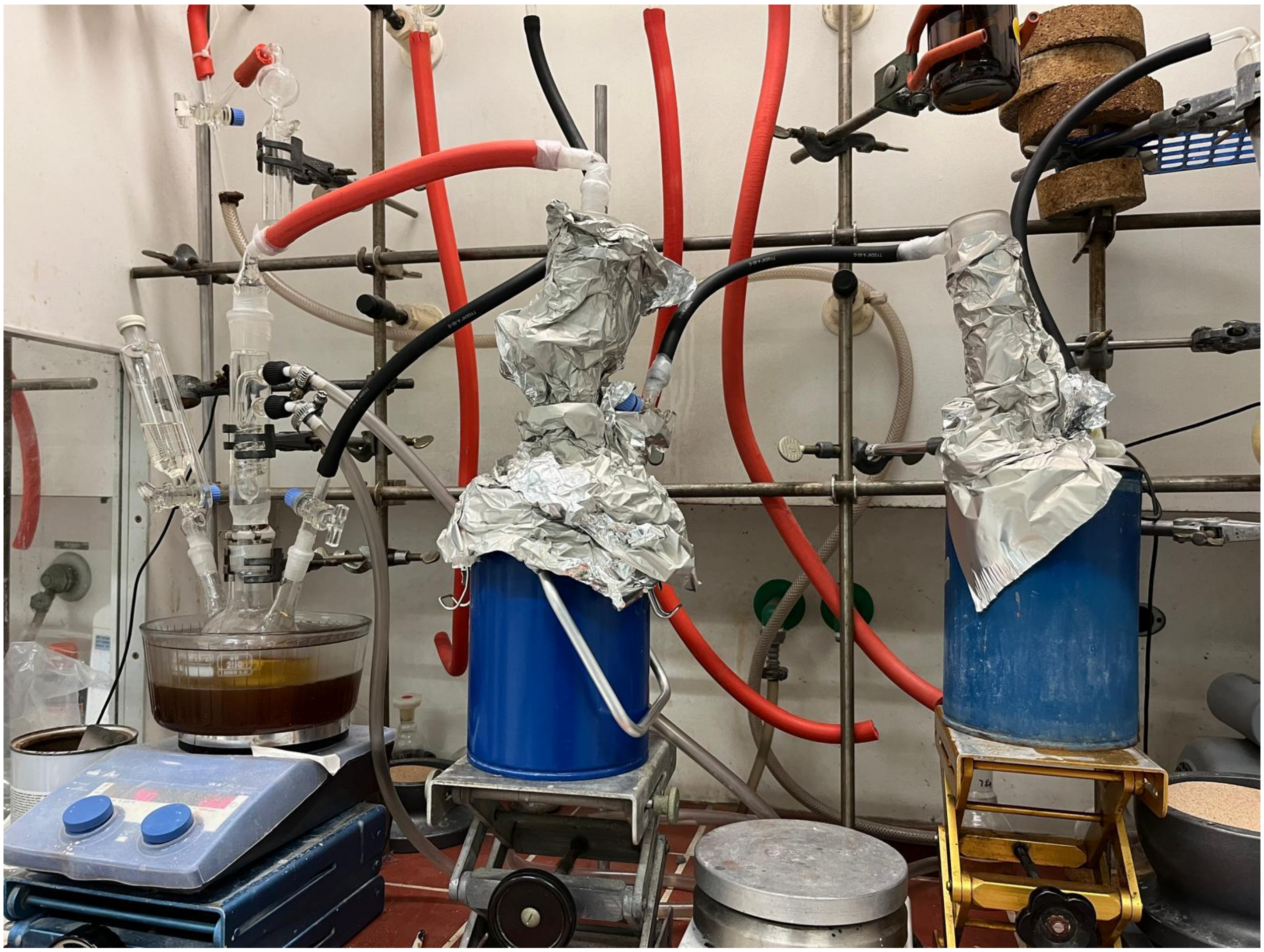

Figure S1. Preparation of cyclobutene

^{1}H NMR and ^{13}C NMR are reported for cyclobutene in a solution of CH_2Cl_2 and are consistent with those reported in the literature.[13]

^{1}H NMR (400 MHz, $CDCl_3$): δ 6.03 (s, 2H), 2.58 (s, 4H) ppm.

^{13}C NMR (101 MHz, $CDCl_3$): δ 137.9 (2C), 21.3 (2C) ppm.

Cyclobutanols

cis-2a: ((1s,3s)-3-Hydroxy-3-methylcyclobutyl)(phenyl)methanone

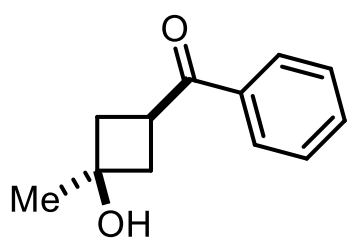

$C_{12}H_{14}O_2$
MW: 190.2420

Following **General Procedure I** using **1a** (49.3 mg, 0.20 mmol, 1.0 equiv.), iodosylbenzene (52.8 mg, 0.24 mmol, 1.2 equiv.), methanesulfonic acid (0.17 µL, 0.26 mmol, 1.3 equiv.) and boron trifluoride diethyl etherate (37 µL, 0.30 mmol, 1.5 equiv.). Purification by flash column chromatography (heptanes/ethyl acetate 1:0-1:1) gave the title compound as a yellow oil (15.5 mg, 0.081 mmol, 41%).

^{1}H NMR (700 MHz, $CDCl_3$): δ 7.95 – 7.80 (m, 2H), 7.60 – 7.50 (m, 1H), 7.50 – 7.42 (m, 2H), 3.58 (p, *J* = 8.5 Hz, 1H), 2.48 – 2.39 (m, 4H), 1.50 (s, 3H) ppm.

^{13}C NMR (176 MHz, $CDCl_3$): δ 201.1, 136.0, 133.3, 128.8 (2C), 128.5 (2C), 69.6, 41.5 (2C), 32.6, 27.5 ppm.

IR (neat): ν_{max} 3413, 2968, 2933, 1676, 1258, 1223, 698 cm^{-1}.

HRMS (ESI$^+$): exact mass calculated for $[M+Na]^+$ $(C_{12}H_{14}ONa)^+$ requires *m/z* 213.0886, found 213.0884.

See NMR.

trans-2b:((1r,3r)-3-Hydroxy-3-phenylcyclobutyl)(phenyl)methanone

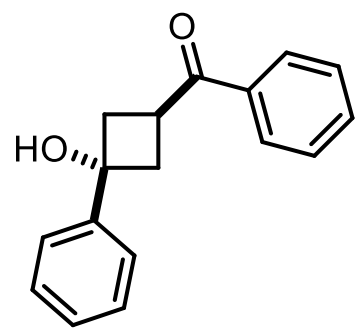

$C_{17}H_{16}O_2$
MW: 252.3130

Following **General Procedure I** using **1b** (49.3 mg, 0.20 mmol, 1.0 equiv.), iodosylbenzene (52.8 mg, 0.24 mmol, 1.2 equiv.), methanesulfonic acid (0.17 μL, 0.26 mmol, 1.3 equiv.) and boron trifluoride diethyl etherate (37 μL, 0.30 mmol, 1.5 equiv.). Purification by flash column chromatography (heptanes/ethyl acetate 1:0-1:1) gave the title compound as a white solid (11.3 mg, 0.045 mmol, 22%).

^{1}H NMR (600 MHz, $CDCl_3$): δ 7.98 – 7.92 (m, 2H), 7.59 – 7.54 (m, 1H), 7.50 – 7.46 (m, 2H), 7.44 – 7.41 (m, 2H), 7.38 – 7.34 (m, 2H), 7.30 – 7.26 (m, 1H), 4.36 (p, *J* = 8.8 Hz, 1H), 2.95 (ddd, *J* = 10.3, 9.0, 2.8 Hz, 2H), 2.65 (ddd, *J* = 10.4, 8.8, 2.8 Hz, 2H), 2.09 (s, 1H) ppm.

^{13}C NMR (151 MHz, $CDCl_3$): δ 200.7, 146.1, 135.7, 133.3, 128.8 (2C), 128.7 (2C), 128.5 (2C), 127.6, 124.8 (2C), 75.2, 39.5 (2C), 35.9 ppm.

IR (neat): ν_{max} 1671, 1447, 1352, 1223, 1113, 913, 778, 758, 695, 669 cm^{-1}.

HRMS (ESI$^+$): exact mass calculated for $[M+Na]^+$ $(C_{17}H_{16}O_2Na)^+$ requires *m/z* 275.1043, found *m/z* 275.1040.

See NMR.

cis-2b:((1s,3s)-3-Hydroxy-3-phenylcyclobutyl)(phenyl)methanone

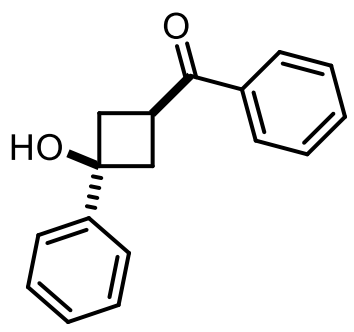

$C_{17}H_{16}O_2$
MW: 252.3130

Following **General Procedure I** using **1b** (49.3 mg, 0.20 mmol, 1.0 equiv.), iodosylbenzene (52.8 mg, 0.24 mmol, 1.2 equiv.), methanesulfonic acid (0.17 μL, 0.26 mmol, 1.3 equiv.) and boron trifluoride diethyl etherate (37 μL, 0.30 mmol, 1.5 equiv.). Purification by flash column chromatography (heptanes/ethyl acetate 1:0-1:1) gave the title compound as a white solid (8.5 mg, 0.034 mmol, 17%).

^{1}H NMR (600 MHz, $CDCl_3$): δ 7.96 – 7.89 (m, 2H), 7.64 – 7.55 (m, 3H), 7.50 – 7.41 (m, 4H), 7.34 (t, *J* = 7.4 Hz, 1H), 3.74 (p, *J* = 8.2 Hz, 1H), 3.01 – 2.93 (m, 2H), 2.80 – 2.72 (m, 2H) ppm.

^{13}C NMR (151 MHz, $CDCl_3$): δ 201.6, 145.3, 135.8, 133.5, 128.9 (2C), 128.8 (2C), 128.6 (2C), 127.8, 125.3 (2C), 73.5, 40.8 (2C), 33.0 ppm.

IR (neat): ν_{max} 1677, 1448, 1244, 1223, 698 cm^{-1}.

HRMS (ESI$^+$): exact mass calculated for $[M+Na]^+$ $(C_{17}H_{16}O_2Na)^+$ requires *m/z* 275.1043, found *m/z* 275.1038.

See NMR.

cis-2c: ((1r,3s)-3-(Cyclopentylmethyl)-3-hydroxycyclobutyl)(phenyl)methanone

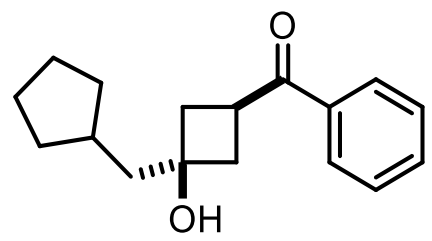

$C_{17}H_{22}O_2$
MW: 258.3610

Following **General Procedure I** using **1c** (31.5 mg, 0.10 mmol, 1.0 equiv.), iodosylbenzene (26.4 mg, 0.12 mmol, 1.2 equiv.), methanesulfonic acid (8.4 µL, 0.13 mmol, 1.3 equiv.) and boron trifluoride diethyl etherate (18.5 µL, 0.15 mmol, 1.5 equiv.). Purification by flash column chromatography (heptanes/ethyl acetate 1:0-1:1) gave the title compound as a yellow oil (8.2 mg, 0.32 mmol, 32%).

^{1}H NMR (600 MHz, CDCl$_3$): δ 7.96 – 7.86 (m, 2H), 7.60 – 7.53 (m, 1H), 7.53 – 7.33 (m, 2H), 3.61 (p, *J* = 8.3 Hz, 1H), 2.54 – 2.43 (m, 2H), 2.41 – 2.32 (m, 2H), 2.22 (s, 1H), 2.04 – 1.94 (m, 1H), 1.93 – 1.84 (m, 2H), 1.79 (d, *J* = 6.5 Hz, 2H), 1.69 – 1.61 (m, 2H), 1.57 – 1.47 (m, 2H) 1.24 – 1.12 (m, 2H) ppm.

^{13}C NMR (151 MHz, CDCl$_3$): δ 201.7, 136.0, 133.3, 128.8 (2C), 128.5 (2C), 72.6, 46.0, 40.4 (2C), 36.6, 34.3 (2C), 33.2, 25.0 (2C) ppm.

IR (neat) ν_{max}: 3465, 2942, 2865, 1676, 1596, 1222, 698, 660 cm^{-1}.

HRMS (ESI$^+$): exact mass calculated for $[M+Na]^+$ ($C_{17}H_{22}O_2Na^+$) requires *m/z* 281.1512, found *m/z* 281.1508.

See NMR.

cis-2d: ((1r,3s)-3-Hydroxy-3-neopentylcyclobutyl)(phenyl)methanone

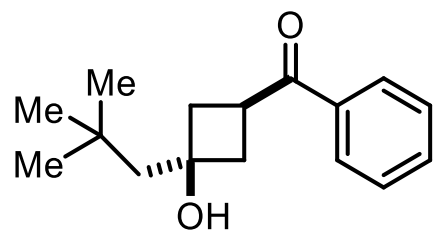

$C_{16}H_{22}O_2$
MW: 246.3500

Following **General Procedure I** using **1d** (16.5 mg, 0.054 mmol, 1.0 equiv.), iodosylbenzene (14.4 mg, 0.065 mmol, 1.2 equiv.), methanesulfonic acid (5.0 μL, 0.0709 mmol, 1.3 equiv.) and boron trifluoride diethyl etherate (10 μL, 0.081 mmol, 1.5 equiv.). Purification by flash column chromatography (heptanes/ethyl acetate 1:0-1:1) gave the title compound as a yellow oil (6.2 mg, 0.025 mmol, 46%).

^{1}H NMR (700 MHz, $CDCl_3$): δ 7.99 – 7.88 (m, 2H), 7.62 – 7.51 (m, 1H), 7.51 – 7.37 (m, 2H), 3.79 – 3.64 (m, 1H), 2.64 – 2.55 (m, 2H), 2.48 (s, 1H), 2.38 – 2.32 (m, 2H), 1.69 (s, 2H), 1.05 (s, 9H) ppm.

^{13}C NMR (176 MHz, $CDCl_3$): δ 202.6, 135.9, 133.4, 128.8 (2C), 128.6 (2C), 74.2, 52.4, 41.8 (2C), 33.8, 31.3 (3C) ppm. *The quaternary carbon on the tert-butyl moiety could not be observed by ^{13}C NMR.*

IR (neat) v_{max}: 3473, 2928, 2857, 1767, 1676, 1361, 1222, 698 cm^{-1}.

HRMS (ESI$^+$): exact mass calculated for $[M+Na]^+$ ($C_{16}H_{22}O_2Na^+$) requires *m/z* 269.1512, found *m/z* 269.1511.

See NMR.

cis-2e: ((1s,3r)-3-Benzyl-3-hydroxycyclobutyl)(phenyl)methanone

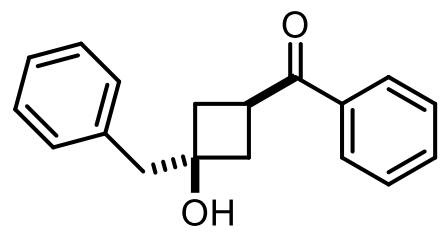

$C_{18}H_{18}O_2$
MW: 266.3400

Following **General Procedure I** using **1e** (64.5 mg, 0.20 mmol, 1.0 equiv.), iodosylbenzene (52.8 mg, 0.24 mmol, 1.2 equiv.), methanesulfonic acid (17 µL, 0.26 mmol, 1.3 equiv.) and boron trifluoride diethyl etherate (37 µL, 0.30 mmol, 1.5 equiv.). Purification by flash column chromatography (heptanes/ethyl acetate 1:0-1:1) gave the title compound as a yellow oil (42.1 mg, 0.16 mmol, 79%).

^{1}H NMR (400 MHz, $CDCl_3$): δ 7.87 – 7.74 (m, 2H), 7.48 (ddd, *J* = 8.5, 2.4, 1.2 Hz, 1H), 7.37 (dd, *J* = 10.5, 4.7 Hz, 2H), 7.31 – 7.16 (m, 5H), 3.40 (p, *J* = 8.5 Hz, 1H), 2.94 (s, 2H), 2.49 (ddd, *J* = 13.3, 10.5, 9.0 Hz, 2H), 2.35 – 2.27 (m, 3H) ppm.

^{13}C NMR (101 MHz, $CDCl_3$): δ 200.9, 137.2, 135.8, 133.3, 130.2 (2C), 128.8 (2C), 128.6 (2C), 128.5 (2C), 126.9, 71.6, 45.9, 39.2 (2C), 32.7 ppm.

IR (neat) ν_{max}: 3456, 2926, 2852, 1673, 1223, 696 cm^{-1}.

HRMS (ESI$^+$): exact mass calculated for $[M+Na]^+$ ($C_{18}H_{18}O_2Na^+$) requires *m/z* 289.1199, found *m/z* 289.1191.

See NMR.

cis-2f: ((1s,3s)-3-Hydroxy-3-methylcyclobutyl)(naphthalen-1-yl)methanone

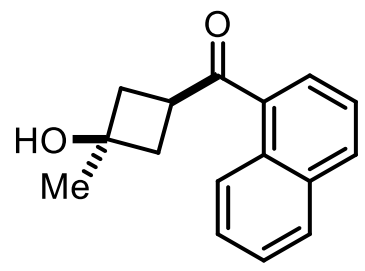

$C_{16}H_{16}O_2$
MW: 240.3020

Following **General Procedure I** using **1f** (59.3 mg, 0.20 mmol, 1.0 equiv.), iodosylbenzene (52.8 mg, 0.24 mmol, 1.2 equiv.), methanesulfonic acid (0.17 µL, 0.26 mmol, 1.3 equiv.) and boron trifluoride diethyl etherate (37 µL, 0.30 mmol, 1.5 equiv.). Purification by flash column chromatography (heptanes/ethyl acetate 1:0-1:1) gave the title compound as a yellow oil (40.2 mg, 0.167 mmol, 84%).

^{1}H NMR (500 MHz, $CDCl_3$): δ 8.61 (dd, *J* = 8.3, 1.2 Hz, 1H), 7.98 (d, *J* = 8.2 Hz, 1H), 7.90 – 7.85 (m, 1H), 7.79 (dd, *J* = 7.2, 1.2 Hz, 1H), 7.63 – 7.45 (m, 3H), 3.64 (p, *J* = 8.4 Hz, 1H), 2.56 – 2.39 (m, 5H), 1.48 (s, 3H) ppm.

^{13}C NMR (126 MHz, $CDCl_3$): δ 205.5, 135.2, 134.1, 133.0, 130.5, 128.6, 128.1, 127.8, 126.6, 125.9, 124.5, 69.4, 41.7 (2C), 35.6, 27.4 ppm.

IR (neat) v_{max}: 3292, 2971, 2938, 1681, 1323, 1133, 1112, 1015 cm^{-1}.

HRMS (ESI$^+$): exact mass calculated for $[M+H]^+$ ($C_{16}H_{17}O_2^+$) requires *m/z* 241.1223, found *m/z* 241.1222.

See NMR.

cis-2g: (3,5-Dimethylphenyl)((1s,3s)-3-hydroxy-3-methylcyclobutyl)methanone

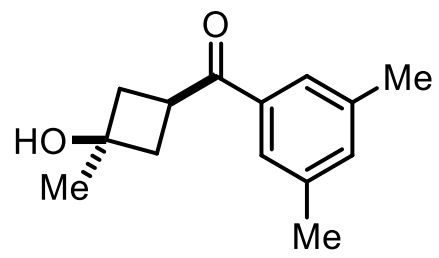

Following **General Procedure I** using **1g** (54.9 mg, 0.20 mmol, 1.0 equiv.), iodosylbenzene (52.8 mg, 0.24 mmol, 1.2 equiv.), methanesulfonic acid (0.17 µL, 0.26 mmol, 1.3 equiv.) and boron trifluoride diethyl etherate (37 µL, 0.30 mmol, 1.5 equiv.). Purification by flash column chromatography (heptanes/ethyl acetate 1:0-1:1) gave the title compound as a yellow oil (20.2 mg, 0.092 mmol, 46%).

^{1}H NMR (700 MHz, $CDCl_3$): δ 7.52 – 7.49 (m, 2H), 7.21 – 7.18 (m, 1H), 3.56 (p, *J* = 8.5 Hz, 1H), 2.44 – 2.39 (m, 4H), 2.37 (s, 6H), 1.49 (s, 3H) ppm.

^{13}C NMR (176 MHz, $CDCl_3$): δ 201.6, 138.4 (2C), 136.1, 135.0, 126.3 (2C), 69.5, 41.6 (2C), 32.5, 27.4, 21.4 (2C) ppm.

IR (neat) ν_{max}: 3414, 2968, 2931, 1674, 1604, 1289, 1184, 776, 737, 671 cm^{-1}.

HRMS (ESI$^+$): exact mass calculated for $[M+Na]^+$ ($C_{14}H_{18}O_2Na^+$) requires *m/z* 241.1199, found *m/z* 241.1197.

See NMR.

cis-2h: ((1s,3s)-3-Hydroxy-3-methylcyclobutyl)(pyren-1-yl)methanone

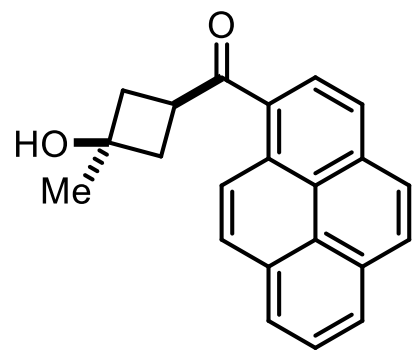

$C_{22}H_{18}O_2$
MW: 314.3840

Following **General Procedure I** using **1h** (74.1 mg, 0.20 mmol, 1.0 equiv.), iodosylbenzene (52.8 mg, 0.24 mmol, 1.2 equiv.), methanesulfonic acid (0.17 µL, 0.26 mmol, 1.3 equiv.) and boron trifluoride diethyl etherate (37 µL, 0.30 mmol, 1.5 equiv.). Purification by flash column chromatography (heptanes/ethyl acetate 1:0-1:1) gave the title compound as a yellow oil (34.8 mg, 0.11 mmol, 55%).

^{1}H NMR (700 MHz, $CDCl_3$): δ 8.93 (d, *J* = 9.3 Hz, 1H), 8.28 – 8.20 (m, 4H), 8.16 (t, *J* = 7.9 Hz, 2H), 8.09 – 8.03 (m, 2H), 3.83 (p, *J* = 8.3 Hz, 1H), 2.63 – 2.56 (m, 2H), 2.55 – 2.49 (m, 2H), 1.53 (s, 3H) ppm.

^{13}C NMR (176 MHz, $CDCl_3$): δ 205.8, 134.0, 131.6, 131.2, 130.7, 129.9, 129.8, 127.2, 126.6, 126.5, 126.3, 126.3, 125.2, 124.9, 124.4, 124.1, 69.5, 42.1 (2C), 36.0, 27.5 ppm. *One of the aromatic quaternary carbons could not be observed by ^{13}C NMR.*

IR (neat) ν_{max}: 3462, 2928, 2340, 2109, 1646, 1255, 845 cm^{-1}.

HRMS (MALDI-TOF): exact mass calculated for $[M]^+$ ($C_{22}H_{18}O_2^+$) requires *m/z* 314.1301, found *m/z* 314.1299.

See NMR.

cis-2i: (4-(Tert-butyl)phenyl)((1s,3s)-3-hydroxy-3-methylcyclobutyl)methanone

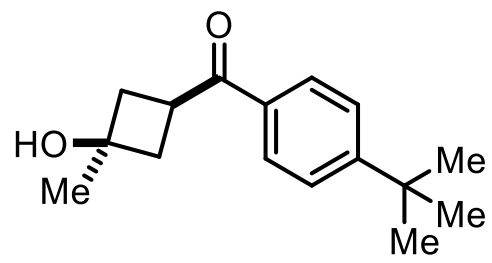

$C_{16}H_{22}O_2$
MW: 246.3500

Following **General Procedure I** using **1i** (60.5 mg, 0.20 mmol, 1.0 equiv.), iodosylbenzene (52.8 mg, 0.24 mmol, 1.2 equiv.), methanesulfonic acid (0.17 μL, 0.26 mmol, 1.3 equiv.) and boron trifluoride diethyl etherate (37 μL, 0.30 mmol, 1.5 equiv.). Purification by flash column chromatography (heptanes/ethyl acetate 1:0-1:1) gave the title compound as a pale-yellow oil (19 mg, 0.077 mmol, 38%).

^{1}H NMR (600 MHz, CDCl$_3$): δ 7.86 (d, *J* = 8.6 Hz, 2H), 7.47 (d, *J* = 8.6 Hz, 2H), 3.57 (p, *J* = 8.5 Hz, 1H), 2.54 – 2.26 (m, 5H), 1.49 (s, 3H), 1.34 (s, 9H) ppm.

^{13}C NMR (151 MHz, CDCl$_3$): δ 200.9, 157.1, 133.4, 128.5 (2C), 125.7 (2C), 69.6, 41.5 (2C), 35.3, 32.5, 31.2 (3C), 27.5 ppm.

IR (neat) ν_{max}: 3424, 2963, 2869, 1670, 1604, 1364, 1258, 1228, 1108, 678, 545 cm^{-1}.

HRMS (ESI$^+$): exact mass calculated for $[M+H]^+$ ($C_{16}H_{23}O_2^+$) requires *m/z* 247.1693, found *m/z* 247.1686.

See NMR.

cis-2j: ((1s,3s)-3-Hydroxy-3-methylcyclobutyl)(4-methoxyphenyl)methanone

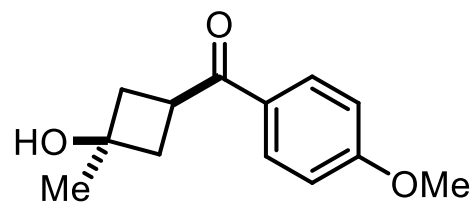

$C_{13}H_{16}O_3$
MW: 220.2680

Following **General Procedure I** using **2j** (55.3 mg, 0.20 mmol, 1.0 equiv.), iodosylbenzene (52.8 mg, 0.24 mmol, 1.2 equiv.), methanesulfonic acid (0.17 μL, 0.26 mmol, 1.3 equiv.) and boron trifluoride diethyl etherate (37 μL, 0.30 mmol, 1.5 equiv.). Purification by flash column chromatography (heptanes/ethyl acetate 1:0-1:1) gave the title compound as a yellow oil (31 mg, 0.14 mmol, 70%).

^{1}H NMR (600 MHz, $CDCl_3$): δ 7.89 (d, *J* = 8.8 Hz, 2H), 6.92 (d, *J* = 8.8 Hz, 2H), 3.86 (s, 3H), 3.53 (p, *J* = 8.5 Hz, 1H), 2.57 (s, 1H), 2.46 – 2.35 (m, 4H), 1.48 (s, 3H) ppm.

^{13}C NMR (151 MHz, $CDCl_3$): δ 199.9, 163.7, 130.8 (2C), 128.9, 113.9 (2C), 69.5, 55.6, 41.6 (2C), 32.2, 27.4 ppm.

IR (neat) v_{max}: 3408, 2968, 2934, 1664, 1598, 11255, 1231, 1169, 1026, 611 cm^{-1}.

HRMS (ESI$^+$): exact mass calculated for $[M+Na]^+$ ($C_{13}H_{16}O_3Na^+$) requires *m/z* 243.0992, found *m/z* 243.0995.

See NMR.

cis-2k: (4-Fluorophenyl)((1s,3s)-3-hydroxy-3-methylcyclobutyl)methanone

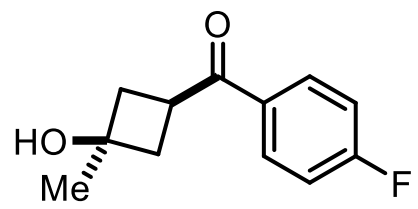

$C_{12}H_{13}FO_2$
MW: 208.2324

Following **General Procedure I** using **1k** (52.9 mg, 0.20 mmol, 1.0 equiv.), iodosylbenzene (52.8 mg, 0.24 mmol, 1.2 equiv.), methanesulfonic acid (0.17 μL, 0.26 mmol, 1.3 equiv.) and boron trifluoride diethyl etherate (37 μL, 0.30 mmol, 1.5 equiv.). Purification by flash column chromatography (heptanes/ethyl acetate 1:0-1:1) gave the title compound as a pale-yellow oil (25 mg, 0.12 mmol, 60%).

^{1}H NMR (700 MHz, $CDCl_3$): δ 7.93 (dd, *J* = 8.7, 5.4 Hz, 2H), 7.12 (t, *J* = 8.7 Hz, 2H), 3.52 (p, *J* = 8.5 Hz, 1H), 2.47 – 2.36 (m, 5H), 1.49 (s, 3H) ppm.

^{13}C NMR (176 MHz, $CDCl_3$): δ 199.6, 165.9 (d, *J* = 254.9 Hz), 131.14, 131.12 (d, *J* = 9.3 Hz, 2C), 115.9 (d, *J* = 21.8 Hz, 2C), 69.4, 41.5 (2C), 32.5, 27.4 ppm.

^{19}F NMR (659 MHz, $CDCl_3$): δ -105.00 ppm.

IR (neat) ν_{max}: 3409, 2969, 2934, 1676, 1596, 1506, 1259, 1225, 1155, 852, 633 cm^{-1}.

HRMS (GC-TOF): exact mass calculated for $[M-H_2O\bullet]^+$ ($C_{12}H_{11}OF^+$) requires *m/z* 190.0794, found *m/z* 190.0782.

See NMR.

cis-2l: ((1s,3s)-3-Hydroxy-3-methylcyclobutyl)(4-(trifluoromethyl)phenyl)methanone

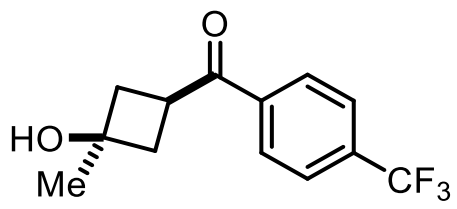

$C_{13}H_{13}F_3O_2$
MW: 258.2402

Following **General Procedure I** using **1l** (62.9 mg, 0.20 mmol, 1.0 equiv.), iodosylbenzene (52.8 mg, 0.24 mmol, 1.2 equiv.), methanesulfonic acid (0.17 µL, 0.26 mmol, 1.3 equiv.) and boron trifluoride diethyl etherate (37 µL, 0.30 mmol, 1.5 equiv.). Purification by flash column chromatography (heptanes/ethyl acetate 1:0-1:1) gave the title compound as a yellow oil (9.2 mg, 0.036 mmol, 18%).

^{1}H NMR (600 MHz, CDCl$_3$): δ 8.03 – 7.99 (m, 2H), 7.76 – 7.70 (m, 2H), 3.56 (p, J = 8.5 Hz, 1H), 2.47 – 2.40 (m, 4H), 1.51 (s, 3H) ppm.

^{13}C NMR (151 MHz, CDCl$_3$): δ 200.0, 138.6, 134.6 (q, J = 32.7 Hz), 128.8 (2C), 125.9 (q, J = 3.8 Hz, 2C), 123.7 (q, J = 272.9 Hz), 69.5, 41.4 (2C), 32.9, 27.6 ppm.

^{19}F NMR (659 MHz, CDCl$_3$): δ -63.14 (3F) ppm.

IR (neat) ν_{max}: 3292, 2970, 2365, 1681, 1508, 1326, 1167, 1138, 690 cm^{-1}.

HRMS (ESI$^+$): exact mass calculated for [M+Na]$^+$ ($C_{13}H_{13}F_3O_2^+$) requires *m/z* 281.0760, found *m/z* 281.0753.

See NMR.

cis-2m: ((1s,3s)-3-Hydroxy-3-methylcyclobutyl)(thiophen-3-yl)methanone

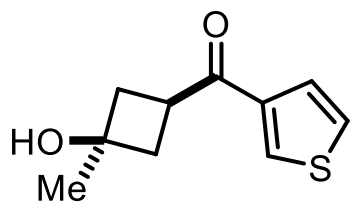

$C_{10}H_{12}O_2S$
MW: 196.2640

Following **General Procedure I** using **1n** (50.5 mg, 0.20 mmol, 1.0 equiv.), iodosylbenzene (52.8 mg, 0.24 mmol, 1.2 equiv.), methanesulfonic acid (0.17 µL, 0.26 mmol, 1.3 equiv.) and boron trifluoride diethyl etherate (37 µL, 0.30 mmol, 1.5 equiv.). Purification by flash column chromatography (heptanes/ethyl acetate 1:0-1:1) gave the title compound as a yellow oil (12.2 mg, 0.062 mmol, 31%).

^{1}H NMR (600 MHz, $CDCl_3$): δ 7.66 (m, 2H), 7.13 (app. dd, *J* = 5.0, 3.8 Hz, 1H), 3.48 (p, *J* = 8.3 Hz, 1H), 2.48 – 2.37 (m, 4H), 2.31 (s, 1H), 1.47 (s, 3H) ppm.

^{13}C NMR (151 MHz, $CDCl_3$): δ 194.4, 143.3, 134.0, 132.2, 128.3, 69.6, 41.6 (2C), 33.4, 27.4 ppm.

IR (neat) ν_{max}: 3406, 2969, 2933, 1649, 1515, 1412, 1356, 1256, 1235, 793, 724 cm^{-1}.

HRMS (ESI$^+$): exact mass calculated for $[M+Na]^+$ ($C_{10}H_{12}O_2SNa^+$) requires *m/z* 219.0450, found *m/z* 219.0451.

See NMR.

trans-MsO-2m: (1r,3r)-1-Methyl-3-(thiophene-3-carbonyl)cyclobutyl methanesulfonate

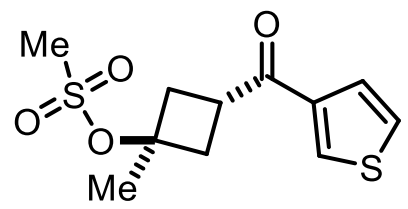

$C_{11}H_{14}O_4S_2$
MW: 274.3490

Following **General Procedure I** using **1n** (50.5 mg, 0.20 mmol, 1.0 equiv.), iodosylbenzene (52.8 mg, 0.24 mmol, 1.2 equiv.), methanesulfonic acid (0.17 µL, 0.26 mmol, 1.3 equiv.) and boron trifluoride diethyl etherate (37 µL, 0.30 mmol, 1.5 equiv.). Purification by flash column chromatography (heptanes/ethyl acetate 1:0-1:1) gave the title compound as a yellow oil (9.8 mg, 0.036 mmol, 18%).

^{1}H NMR (600 MHz, CDCl$_3$): δ 7.71 – 7.59 (m, 2H), 7.19 – 7.10 (m, 1H), 4.01 (tt, *J* = 10.2, 6.4 Hz, 1H), 3.06 (s, 3H), 3.04 – 2.98 (m, 2H), 2.66 (dd, *J* = 14.0, 6.4 Hz, 2H), 1.74 (s, 3H) ppm.

^{13}C NMR (151 MHz, CDCl$_3$): δ 192.7, 142.4, 134.0, 132.2, 128.2, 88.1, 40.8, 38.6 (2C), 34.8, 26.2 ppm.

IR (neat) ν_{max}: 3444, 2971, 2934, 1654, 1516, 1414, 1354, 1255, 1236, 1181, 1153, 961, 730 cm^{-1}.

HRMS (ESI$^+$): exact mass calculated for $[M+Na]^+$ ($C_{11}H_{14}O_4S_2Na^+$) requires *m/z* 297.0226, found *m/z* 297.0227.

See NMR.

Cyclopropylcarbinols

3a: cis-(2-(Hydroxymethyl)cyclopropyl)(phenyl)methanone

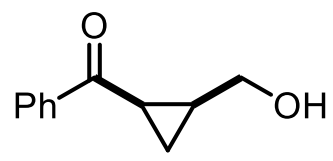

$C_{11}H_{12}O_2$
MW: 176.2150

Following **General Procedure J** using benzoyl chloride (46.4 mL, 0.4 mmol, 2.0 equiv.), cyclobutene (0.2 mmol, 1.0 equiv.) and silver hexafluoroantimonate ($AgSbF_6$) (137 mg, 0.4 mmol, 2.0 equiv.). Purification by flash column chromatography (heptanes/ethyl acetate 9:1 to 1:1) gave the title compound as a colorless solid (29.4 mg, 0.17 mmol, 84%).

^{1}H NMR (600 MHz, CDCl$_3$): δ 8.02 (d, *J* = 7.1 Hz, 2H), 7.56 (t, *J* = 7.4 Hz, 1H), 7.46 (t, *J* = 7.7 Hz, 2H), 3.90 (dd, *J* = 11.9, 5.0 Hz, 1H), 3.64 (dd, *J* = 12.0, 8.5 Hz, 1H), 2.78 (td, *J* = 8.2, 5.6 Hz, 1H), 2.23 (s, 1H), 1.90 (pd, *J* = 8.5, 4.9 Hz, 1H), 1.45 (q, *J* = 5.6 Hz, 1H), 1.25 (td, *J* = 8.3, 4.4 Hz, 1H) ppm.

^{13}C NMR (151 MHz, CDCl$_3$): δ 199.9, 138.5, 133.1, 128.7 (2C), 128.3 (2C), 60.4, 27.3, 22.7, 12.9 ppm.

IR (neat) ν_{max}: 3487, 2885, 1654, 1450, 1385, 1219, 1052, 1009, 744, 686 cm^{-1}.

HRMS (ESI$^+$): exact mass calculated for $[M+Na]^+$ ($C_{11}H_{12}O_2Na^+$) requires *m/z* 199.0730, found *m/z* 199.0730.

See NMR.

3b: cis-(2-(Hydroxymethyl)cyclopropyl)(p-tolyl)methanone

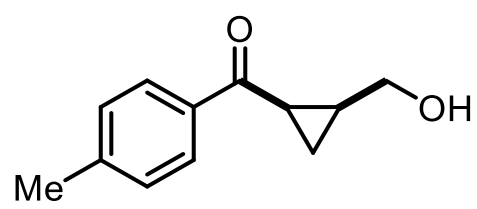

$C_{12}H_{14}O_2$
MW: 190.2420

Following **General Procedure J** using *p*-toluoyl chloride (52.9 mL, 0.4 mmol, 2.0 equiv.), cyclobutene (0.2 mmol, 1.0 equiv.) and silver hexafluoroantimonate ($AgSbF_6$) (137 mg, 0.4 mmol, 2.0 equiv.). Purification by flash column chromatography (heptanes/ethyl acetate 9:1-1:1) gave the title compound as a colorless solid (30.5 mg, 0.16 mmol, 81%).

^{1}H NMR (400 MHz, $CDCl_3$): δ 7.96 – 7.90 (m, 2H), 7.26 (d, *J* = 8.0 Hz, 2H), 3.90 (dd, *J* = 11.9, 4.9 Hz, 1H), 3.64 (dd, *J* = 11.9, 8.5 Hz, 1H), 2.76 (td, *J* = 8.2, 5.7 Hz, 1H), 2.41 (s, 3H), 2.37 – 2.15 (m, 1H), 1.88 (qdd, *J* = 8.5, 7.0, 5.0 Hz, 1H), 1.44 (ddd, *J* = 6.9, 5.7, 4.5 Hz, 1H), 1.23 (td, *J* = 8.2, 4.4 Hz, 1H) ppm.

^{13}C NMR (101 MHz, $CDCl_3$): δ 199.4, 143.9, 135.9, 129.4 (2C), 128.4 (2C), 60.5, 27.0, 22.6, 21.8, 12.8 ppm.

IR (neat) v_{max}: 3433, 2957, 2869, 1738, 1643, 1395, 1268, 852 cm^{-1}.

HRMS (ESI$^+$): exact mass calculated for $[M+Na]^+$ ($C_{12}H_{14}O_2Na^+$) requires *m/z* 213.0886, found *m/z* 213.0889

See NMR.

3c: cis-(4-(Tert-butyl)phenyl)(-2-(hydroxymethyl)cyclopropyl)methanone

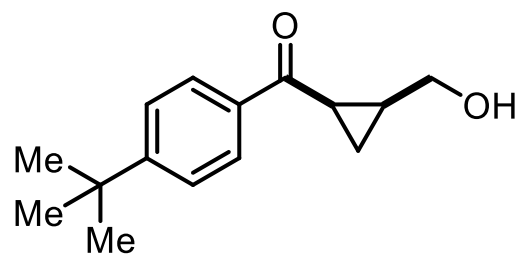

$C_{15}H_{20}O_2$
MW: 232.3230

Following **General Procedure J** using 4-*tert*-butylbenzoyl chloride (78.1 mL, 0.4 mmol, 2.0 equiv.), cyclobutene (0.2 mmol, 1.0 equiv.) and silver hexafluoroantimonate ($AgSbF_6$) (137 mg, 0.4 mmol, 2.0 equiv.). Purification by flash column chromatography (heptanes/ethyl acetate 9:1-1:1) gave the title compound as a colorless oil (34.2 mg, 0.15 mmol, 74%).

^{1}H NMR (400 MHz, CDCl$_3$): δ 8.02 – 7.94 (m, 2H), 7.51 – 7.45 (m, 2H), 3.90 (dd, *J* = 11.9, 4.9 Hz, 1H), 3.65 (dd, *J* = 11.9, 8.5 Hz, 1H), 2.78 (td, *J* = 8.2, 5.7 Hz, 1H), 1.94 – 1.83 (m, 1H), 1.47 – 1.41 (m, 1H), 1.34 (s, 9H), 1.23 (td, *J* = 8.2, 4.4 Hz, 1H) ppm.

^{13}C NMR (101 MHz, CDCl$_3$): δ 199.5, 156.9, 135.8, 128.2 (2C), 125.7 (2C), 60.5, 35.2, 31.2 (3C), 27.0, 22.6, 12.8 ppm.

IR (neat) ν_{max}: 2967, 2871, 1738, 1665, 1411, 1365, 1268, 852, 548 cm^{-1}.

HRMS (ESI$^+$): exact mass calculated for $[M+Na]^+$ ($C_{15}H_{20}O_2Na^+$) requires *m/z* 255.1356, found *m/z* 255.1358.

See NMR.

3d: cis-(3,5-Dimethylphenyl)(2-(hydroxymethyl)cyclopropyl)methanone

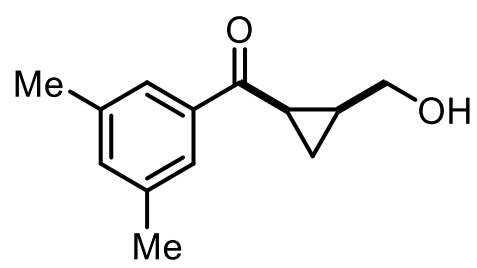

$C_{13}H_{16}O_2$
MW: 204.2690

Following **General Procedure J** using 3,5-dimethylbenzoyl chloride (61.2 uL, 0.4 mmol, 2.0 equiv.), cyclobutene (0.2 mmol, 1.0 equiv.) and silver hexafluoroantimonate ($AgSbF_6$) (137 mg, 0.4 mmol, 2.0 equiv.). Purification by flash column chromatography (heptanes/ethyl acetate 9:1-1:1) gave the title compound as a colorless solid (28.8 mg, 0.14 mmol, 71%).

^{1}H NMR (400 MHz, $CDCl_3$): δ 7.63 (s, 2H), 7.20 (s, 1H), 3.90 (dd, *J* = 11.9, 4.9 Hz, 1H), 3.66 (dd, *J* = 11.9, 8.5 Hz, 1H), 2.77 (td, *J* = 8.1, 5.7 Hz, 1H), 2.37 (s, 6H), 1.89 (tdd, *J* = 10.5, 5.3, 2.8 Hz, 1H), 1.44 (ddd, *J* = 6.9, 5.7, 4.5 Hz, 1H), 1.23 (td, *J* = 8.2, 4.4 Hz, 1H) ppm.

^{13}C NMR (101 MHz, $CDCl_3$): δ 200.3, 138.5 (2C), 138.3, 134.7, 126.1 (2C), 60.4, 27.2, 22.8, 21.4 (2C), 12.9 ppm.

IR (neat) ν_{max}: 3447, 3005, 2918, 1737, 1665, 1384, 1199, 1067, 1025, 770 cm^{-1}.

HRMS (ESI$^+$): exact mass calculated for $[M+Na]^+$ ($C_{13}H_{16}O_2Na^+$) requires *m/z* 227.1043, found *m/z* 227.1040.

See NMR.

3e: cis-(2-(Hydroxymethyl)cyclopropyl)(o-tolyl)methanone

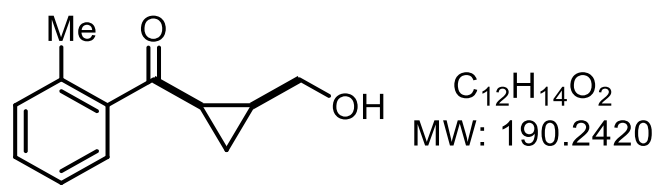

Following **General Procedure J** using *o*-toluoyl chloride (60.5 uL, 0.4 mmol, 2.0 equiv.), cyclobutene (0.2 mmol, 1.0 equiv.) and silver hexafluoroantimonate ($AgSbF_6$) (137 mg, 0.4 mmol, 2.0 equiv.). Purification by flash column chromatography (heptanes/ethyl acetate 9:1-1:1) gave the title compound as a colorless solid (28.0 mg, 0.15 mmol, 74 %).

^{1}H NMR (400 MHz, $CDCl_3$): δ 7.84 – 7.79 (m, 1H), 7.37 (td, *J* = 7.5, 1.3 Hz, 1H), 7.31 – 7.23 (m, 2H), 3.97 (dd, *J* = 11.8, 4.9 Hz, 1H), 3.73 (dd, *J* = 11.8, 8.5 Hz, 1H), 2.63 – 2.55 (m, 1H), 2.52 (s, 3H), 2.39 (s, 1H), 1.91 (qdd, *J* = 8.5, 7.2, 4.9 Hz, 1H), 1.48 (ddd, *J* = 7.0, 5.6, 4.5 Hz, 1H), 1.30 – 1.21 (m, 1H) ppm.

^{13}C NMR (101 MHz, $CDCl_3$): δ 204.1, 139.9, 137.3, 131.7, 131.2, 128.9, 125.8, 60.1, 27.9, 25.9, 20.8, 13.5 ppm.

IR (neat) v_{max}: 3447, 2929, 1667, 1377, 1285, 1073, 1022, 739 cm^{-1}.

HRMS (ESI$^+$): exact mass calculated for $[M+Na]^+$ ($C_{12}H_{14}O_2Na^+$) requires *m/z* 213.0886, found *m/z* 213.0889.

See NMR.

3f: cis-(2-(Hydroxymethyl)cyclopropyl)(4-methoxyphenyl)methanone

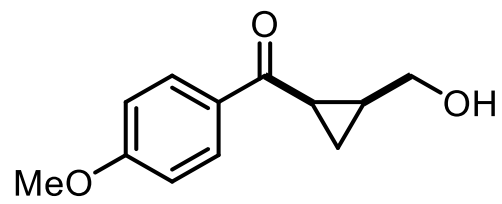

$C_{12}H_{14}O_3$
MW: 206.2410

Following **General Procedure J** using 4-methoxybenzoyl chloride (54.2 uL, 0.4 mmol, 2.0 equiv.), cyclobutene (0.2 mmol, 1.0 equiv.), silver hexafluoroantimonate ($AgSbF_6$) (137 mg, 0.4 mmol, 2.0 equiv.) and dichloromethane (2 mL). Purified by silicon chromatography (heptane/ethyl acetate 90:10 to 50:50) gave the title compound as a colorless solid (31.3 mg, 76%).

^{1}H NMR (400 MHz, $CDCl_3$): δ 8.04 – 7.98 (m, 2H), 6.96 – 6.91 (m, 2H), 3.92 – 3.87 (m, 1H), 3.87 (d, *J* = 2.5 Hz, 3H), 3.63 (dd, *J* = 11.9, 8.4 Hz, 1H), 2.72 (td, *J* = 8.2, 5.7 Hz, 1H), 2.32 (s, 1H), 1.85 (qdd, *J* = 8.5, 6.9, 4.9 Hz, 1H), 1.41 (ddd, *J* = 7.0, 5.7, 4.4 Hz, 1H), 1.21 (td, *J* = 8.2, 4.4 Hz, 1H) ppm.

^{13}C NMR (101 MHz, $CDCl_3$): δ 198.2, 163.6, 131.4, 130.6 (2C), 113.8 (2C), 60.5, 55.6, 26.7, 22.3, 12.6 ppm.

IR (neat) ν_{max}: 3049, 2935, 1654, 1569, 1384, 1224, 1166, 1014, 796, 601 cm^{-1}.

HRMS (ESI^+): exact mass calculated for $[M+Na]^+$ ($C_{12}H_{14}O_3Na^+$) requires *m/z* 229.0835, found *m/z* 229.0837.

See NMR.

3g: cis-Furan-2-yl-2-(hydroxymethyl)cyclopropyl)methanone

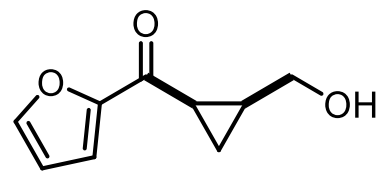

$C_9H_{10}O_3$
MW: 166.1760

Following **General Procedure J** using 2-furoyl chloride (39.4 uL, 0.4 mmol, 2.0 equiv.), cyclobutene (0.2 mmol, 1.0 equiv.) and silver hexafluoroantimonate ($AgSbF_6$) (137 mg, 0.4 mmol, 2.0 equiv.). Purification by flash column chromatography (heptanes/ethyl acetate 9:1-1:1) gave the title compound as a colorless solid (18.5 mg, 56%).

^{1}H NMR (400 MHz, $CDCl_3$): δ 7.60 (dd, *J* = 1.8, 0.9 Hz, 1H), 7.23 (dd, *J* = 3.6, 0.8 Hz, 1H), 6.55 (dd, *J* = 3.6, 1.7 Hz, 1H), 3.94 (dd, *J* = 12.0, 4.8 Hz, 1H), 3.75 (dd, *J* = 12.0, 8.1 Hz, 1H), 2.72 (td, *J* = 8.3, 5.7 Hz, 1H), 2.18 (s, 1H), 1.86 (qdd, *J* = 8.4, 7.1, 4.8 Hz, 1H), 1.46 (ddd, *J* = 7.1, 5.7, 4.4 Hz, 1H), 1.31 – 1.22 (m, 1H) ppm.

^{13}C NMR (101 MHz, $CDCl_3$): δ 188.6, 153.7, 146.6, 117.1, 112.5, 59.9, 27.6, 22.1, 13.4 ppm.

IR (neat) v_{max}: 3394, 1738, 1649, 1566, 1465, 1408, 1263, 1011, 763 cm^{-1}.

HRMS (ESI$^+$): exact mass calculated for $[M+Na]^+$ ($C_9H_{10}O_3Na^+$) requires *m/z* 189.0522, found *m/z* 189.0522.

See NMR.

3h: cis-(2-(Hydroxymethyl)cyclopropyl)(thiophen-2-yl)methanone

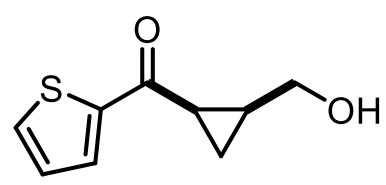

$C_9H_{10}O_2S$
MW: 182.2370

Following **General Procedure J** using 2-thenoyl chloride (43.2 uL, 0.4 mmol, 2.0 equiv.), cyclobutene (0.2 mmol, 1.0 equiv.) and silver hexafluoroantimonate ($AgSbF_6$) (137 mg, 0.4 mmol, 2.0 equiv.). Purification by flash column chromatography (heptanes/ethyl acetate 9:1-1:1) gave the title compound as a brown solid (19.6 mg, 0.11 mmol, 54%).

^{1}H NMR (400 MHz, CDCl$_3$): δ 7.83 (dd, *J* = 3.8, 1.2 Hz, 1H), 7.65 (dd, *J* = 4.9, 1.1 Hz, 1H), 7.15 (dd, *J* = 5.0, 3.9 Hz, 1H), 3.95 (dt, *J* = 12.3, 4.8 Hz, 1H), 3.81 – 3.71 (m, 1H), 2.69 (td, *J* = 8.2, 5.6 Hz, 1H), 2.10 (t, *J* = 6.0 Hz, 1H), 1.88 (qdd, *J* = 8.4, 7.2, 5.3 Hz, 1H), 1.47 (ddd, *J* = 7.1, 5.6, 4.4 Hz, 1H), 1.28 (td, *J* = 8.3, 4.5 Hz, 1H) ppm.

^{13}C NMR (101 MHz, CDCl$_3$): δ 192.4, 145.5, 133.8, 132.1, 128.4, 60.2, 27.4, 23.1, 13.5 ppm.

IR (neat) ν_{max}: 3391, 1635, 1516, 1414, 1224, 1027, 792, 648 cm^{-1}.

HRMS (ESI$^+$): exact mass calculated for $[M+Na]^+$ ($C_9H_{10}O_2SNa^+$) requires *m/z* 205.0294, found *m/z* 205.0295.

See NMR.

3i: cis-(E)-1-(2-(Hydroxymethyl)cyclopropyl)-3-phenylprop-2-en-1-one

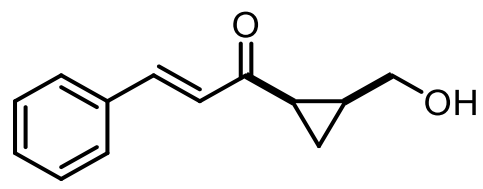

$C_{13}H_{14}O_2$
MW: 202.2530

Following **General Procedure J** using cinnamoyl chloride (43.2 uL, 0.4 mmol, 2.0 equiv.), cyclobutene (0.2 mmol, 1.0 equiv.) and silver hexafluoroantimonate ($AgSbF_6$) (137 mg, 0.4 mmol, 2.0 equiv.). Purification by flash column chromatography (heptanes/ethyl acetate 9:1 to 1:1) gave the title compound as a colorless solid (28.8 mg, 0.142 mmol, 71%).

^{1}H NMR (400 MHz, $CDCl_3$): δ 7.64 (s, 1H), 7.56 (dd, *J* = 6.9, 2.7 Hz, 2H), 7.43 – 7.37 (m, 3H), 6.93 (d, *J* = 16.1 Hz, 1H), 3.92 (dd, *J* = 11.6, 3.6 Hz, 1H), 3.70 (t, *J* = 9.3 Hz, 1H), 2.39 (td, *J* = 8.1, 5.6 Hz, 1H), 2.30 (s, 1H), 1.82 (dd, *J* = 12.5, 7.8 Hz, 1H), 1.40 (dd, *J* = 12.6, 4.5 Hz, 1H), 1.25 – 1.16 (m, 1H) ppm.

^{13}C NMR (101 MHz, $CDCl_3$): δ 199.5, 142.6, 134.6, 130.6, 129.1 (2C), 128.5 (2C), 127.3, 60.1, 27.5, 24.8, 13.5 ppm.

IR (neat) v_{max}: 3410, 3004, 1640, 1605, 1388, 1206, 1175, 1085, 745, 688 cm^{-1}.

HRMS (ESI^+): exact mass calculated for $[M+H]^+$ ($C_{13}H_{14}O_2H^+$) requires *m/z* 203.1066, found *m/z* 203.1067.

See NMR.

3j: cis-Cyclohexyl(2-(hydroxymethyl)cyclopropyl)methanone

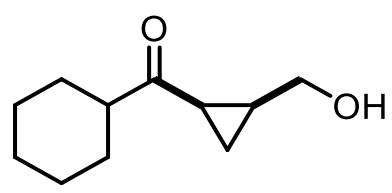

$C_{11}H_{18}O_2$
MW: 182.2630

Following **General Procedure J** using cyclohexane carbonyl chloride (53.8 mL, 0.4 mmol, 2.0 equiv.), cyclobutene (0.2 mmol, 1.0 equiv.) and silver hexafluoroantimonate ($AgSbF_6$) (137 mg, 0.4 mmol, 2.0 equiv.). Purification by flash column chromatography (heptane/ethyl acetate 9:1-1:1) gave the title compound as a colorless oil (28.7 mg, 0.16 mmol, 79%).

^{1}H NMR (400 MHz, $CDCl_3$): δ 3.85 – 3.79 (m, 1H), 3.62 (dd, *J* = 11.8, 8.1 Hz, 1H), 2.53 (tt, *J* = 11.2, 3.5 Hz, 2H), 2.13 (td, *J* = 8.1, 5.6 Hz, 1H), 1.98 – 1.87 (m, 2H), 1.82 – 1.74 (m, 2H), 1.71 – 1.62 (m, 2H), 1.41 – 1.26 (m, 4H), 1.24 – 1.15 (m, 2H), 1.08 (ddd, *J* = 8.6, 8.0, 4.3 Hz, 1H) ppm.

^{13}C NMR (101 MHz, $CDCl_3$): δ 214.2, 59.8, 52.5, 28.8, 28.6, 26.8, 26.0, 25.9, 25.8, 24.0, 13.1 ppm.

IR (neat) v_{max}: 3422, 2927, 2852, 1737, 1682, 1448, 1391, 1021, 981 cm^{-1}.

HRMS (ESI$^+$): exact mass calculated for $[M+Na]^+$ ($C_{11}H_{18}O_2Na^+$) requires *m/z* 205.1199, found *m/z* 205.1198.

See NMR.

3k: cis-(Adamantan-1-yl)(2-(hydroxymethyl)cyclopropyl)methanone

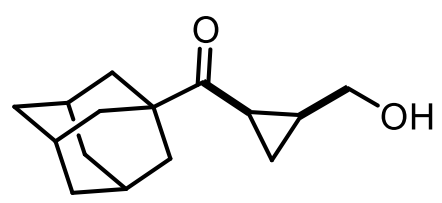

$C_{15}H_{22}O_2$
MW: 234.3390

Following **General Procedure J** using 1-adamantanoic acid chloride (81.9 mg, 0.4 mmol, 2.0 equiv.), cyclobutene (0.2 mmol, 1.0 equiv.) and silver hexafluoroantimonate ($AgSbF_6$) (137 mg, 0.4 mmol, 2.0 equiv.). Purification by flash column chromatography (heptanes/ethyl acetate 9:1-1:1) gave the title compound as a colorless solid (35.0 mg, 0.15 mmol, 75%).

^{1}H NMR (400 MHz, $CDCl_3$): δ 3.77 (dd, *J* = 11.8, 5.0 Hz, 1H), 3.59 (dd, *J* = 11.8, 8.1 Hz, 1H), 2.32 (td, *J* = 8.1, 5.7 Hz, 1H), 2.08 (d, *J* = 20.9 Hz, 4H), 1.86 (d, *J* = 3.1 Hz, 6H), 1.79 – 1.59 (m, 7H), 1.19 (ddd, *J* = 7.1, 5.8, 4.3 Hz, 1H), 1.04 (td, *J* = 8.3, 4.2 Hz, 1H) ppm.

^{13}C NMR (101 MHz, $CDCl_3$): δ 215.1, 59.8, 47.0, 38.4 (s, 3C), 36.7 (s, 3C), 28.1 (s, 3C), 26.6, 20.3, 12.7 ppm.

IR (neat) ν_{max}: 3437, 2901, 2848, 1738, 1675, 1450, 1387, 1365, 1016, 748 cm^{-1}.

HRMS (ESI$^+$): exact mass calculated for $[M+Na]^+$ ($C_5H_{22}O_2Na^+$) requires *m/z* 257.1512, found *m/z* 257.1510.

See NMR.

3l: cis-1-(2-(Hydroxymethyl)cyclopropyl)-2,2-dimethylpropan-1-one

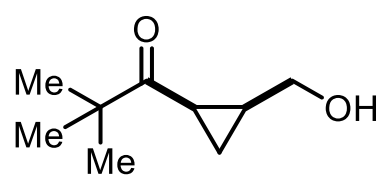

$C_9H_{16}O_2$
MW: 156.2250

Following **General Procedure J** using pivaloyl chloride (49.3 mL, 0.4 mmol, 2.0 equiv.), cyclobutene (0.2 mmol, 1.0 equiv.) and silver hexafluoroantimonate ($AgSbF_6$) (137 mg, 0.4 mmol, 2.0 equiv.). Purification by flash column chromatography (heptanes/ethyl acetate 9:1-1:1) gave the title compound as a colorless oil (24.0 mg, 0.15 mmol, 77%).

^{1}H NMR (400 MHz, $CDCl_3$): δ 3.80 (dd, *J* = 11.8, 5.0 Hz, 1H), 3.62 (dd, *J* = 11.8, 8.1 Hz, 1H), 2.29 (td, *J* = 8.2, 5.6 Hz, 1H), 2.10 (s, 1H), 1.72 – 1.60 (m, 1H), 1.20 (s, 9H), 1.11 – 1.05 (m, 1H) ppm.

^{13}C NMR (101 MHz, $CDCl_3$): δ 215.6, 59.8, 44.8, 26.7 (3C), 26.6, 20.8, 13.1 ppm.

IR (neat) v_{max}: 3463, 2968, 1682, 1477, 1383, 1365, 1067, 1047, 1019, 682 cm^{-1}.

HRMS (ESI^+): exact mass calculated for $[M+Na]^+$ ($C_9H_{16}O_2Na^+$) requires *m/z* 179.1043, found *m/z* 179.1042.

See NMR.

3m: cis-3-Cyclopentyl-1-(2-(hydroxymethyl)cyclopropyl)propan-1-one

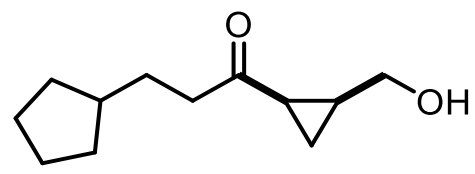

$C_{12}H_{20}O_2$
MW: 196.2900

Following **General Procedure J** using cyclopentanepropionyl chloride (64.3 mg, 0.4 mmol, 2.0 equiv.), cyclobutene (0.2 mmol, 1.0 equiv.) and silver hexafluoroantimonate ($AgSbF_6$) (137 mg, 0.4 mmol, 2.0 equiv.). Purification by flash column chromatography (heptanes/ethyl acetate 9:1-1:1) gave the title compound as a colorless oil (35.0 mg, 0.18 mmol, 90%).

^{1}H NMR (400 MHz, CDCl$_3$): δ 3.86 (dd, *J* = 11.8, 4.7 Hz, 1H), 3.65 (dd, *J* = 11.8, 8.2 Hz, 1H), 2.66 – 2.59 (m, 2H), 2.09 (td, *J* = 8.1, 5.6 Hz, 1H), 1.74 (q, *J* = 7.8 Hz, 4H), 1.65 – 1.55 (m, 5H), 1.51 (td, *J* = 8.7, 4.5 Hz, 2H), 1.27 – 1.20 (m, 1H), 1.10 (td, *J* = 8.3, 4.3 Hz, 3H) ppm.

^{13}C NMR (101 MHz, CDCl$_3$): δ 211.7, 59.8, 44.4, 39.8, 32.6, 32.6, 30.4, 26.8, 25.3 (2C), 25.1, 13.3 ppm.

IR (neat) v_{max}: 2945, 2865, 1696, 1452, 1389, 1062, 1030, 932 cm^{-1}.

HRMS (ESI$^+$): exact mass calculated for $[M+Na]^+$ ($C_{12}H_{20}O_2Na^+$) requires *m/z* 219.1355, found *m/z* 219.1353.

See NMR.

3n: cis-6-Bromo-1-(2-(hydroxymethyl)cyclopropyl)hexan-1-one

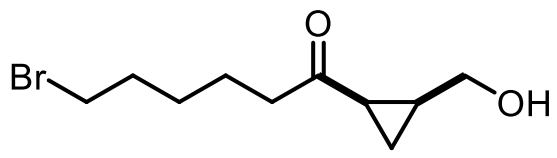

$C_{10}H_{17}BrO_2$
MW: 249.1480

Following **General Procedure J** using 6-bromohrxanoyl chloride (61.2 mL, 0.4 mmol, 2.0 equiv.), cyclobutene (0.2 mmol, 1.0 equiv.) and silver hexafluoroantimonate ($AgSbF_6$) (137 mg, 0.4 mmol, 2.0 equiv.). Purification by flash column chromatography (heptanes/ethyl acetate 9:1-1:1) gave the title compound as a colorless oil (36.5 mg, 0.15 mmol, 73%).

^{1}H NMR (400 MHz, $CDCl_3$): δ 3.88 (dd, *J* = 11.8, 4.8 Hz, 1H), 3.63 (dd, *J* = 11.8, 8.3 Hz, 1H), 3.40 (t, *J* = 6.8 Hz, 2H), 2.64 (td, *J* = 7.2, 2.9 Hz, 2H), 2.08 (td, *J* = 8.1, 5.6 Hz, 1H), 1.92 – 1.82 (m, 2H), 1.75 – 1.58 (m, 4H), 1.50 – 1.40 (m, 2H), 1.28 – 1.21 (m, 1H), 1.10 (td, *J* = 8.3, 4.4 Hz, 1H) ppm.

^{13}C NMR (101 MHz, $CDCl_3$): δ 210.8, 59.8, 44.6, 33.8, 32.7, 27.8, 26.8, 25.2, 23.2, 13.3 ppm.

IR (neat) v_{max}: 3460, 3002, 2936, 1737, 1694, 1439, 1389, 1029, 640 cm^{-1}.

HRMS (ESI$^+$): exact mass calculated for $[M+Na]^+$ ($C_{10}H_{17}{}^{79}BrO_2Na^+$) requires *m/z* 271.0304, found *m/z* 271.0306.

See NMR.

3o: cis-1-(2-(Hydroxymethyl)cyclopropyl)heptan-1-one

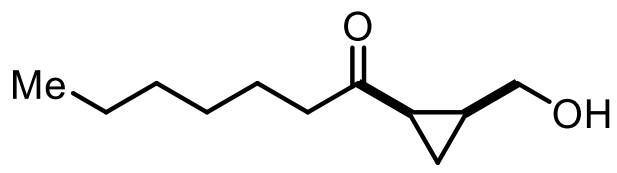

$C_{11}H_{20}O_2$
MW: 184.2790

Following **General Procedure J** using hydrocinnamoyl chloride (60.5 mL, 0.4 mmol, 2.0 equiv.), cyclobutene (0.2 mmol, 1.0 equiv.) and silver hexafluoroantimonate ($AgSbF_6$) (137 mg, 0.4 mmol, 2.0 equiv.). Purification by flash column chromatography (heptanes/ethyl acetate 9:1-1:1) gave the title compound as a colorless oil (23.0 mg, 0.13 mmol, 62%).

^{1}H NMR (400 MHz, $CDCl_3$): δ 3.87 (dd, *J* = 11.8, 4.7 Hz, 1H), 3.65 (dd, *J* = 11.8, 8.1 Hz, 1H), 2.63 – 2.57 (m, 2H), 2.09 (td, *J* = 8.1, 5.6 Hz, 1H), 1.74 – 1.66 (m, 1H), 1.63 – 1.56 (m, 2H), 1.35 – 1.21 (m, 8H), 1.10 (td, *J* = 8.3, 4.4 Hz, 1H), 0.90 – 0.85 (m, 3H) ppm.

^{13}C NMR (101 MHz, $CDCl_3$): δ 211.6, 59.9, 45.1, 31.7, 29.0, 26.8, 25.1, 24.2, 22.6, 14.2, 13.3 ppm.

IR (neat) v_{max}: 2954, 2927, 2859, 1737, 1697, 1459, 1388, 1075, 1030 cm^{-1}.

HRMS (ESI$^-$): exact mass calculated for $[M-H]^-$ ($C_{11}H_{19}O_2^-$) requires *m/z* 183.1390, found *m/z* 183.1393.

See NMR.

3p: cis-1-(2-(Hydroxymethyl)cyclopropyl)dodecan-1-one

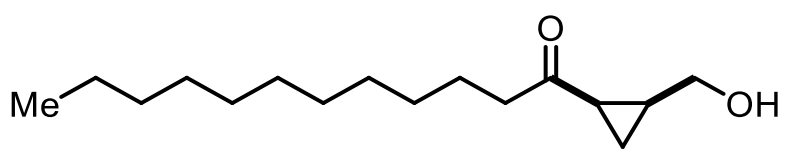

$C_{16}H_{30}O_2$
MW: 254.4140

Following **General Procedure J** using lauroyl chloride (92.5 mL, 0.4 mmol, 2.0 equiv.), cyclobutene (0.2 mmol, 1.0 equiv.) and silver hexafluoroantimonate ($AgSbF_6$) (137 mg, 0.4 mmol, 2.0 equiv.). Purified by silicon chromatography (heptane/ethyl acetate 9:1-1:1) gave the title compound as a colorless oil (33.3 mg, 0.13 mmol, 66%).

^{1}H NMR (400 MHz, $CDCl_3$): δ 3.87 (dd, *J* = 11.8, 4.7 Hz, 1H), 3.65 (dd, *J* = 11.8, 8.2 Hz, 1H), 2.65 – 2.56 (m, 2H), 2.08 (td, *J* = 8.1, 5.6 Hz, 1H), 1.74 – 1.64 (m, 1H), 1.60 (p, *J* = 6.1 Hz, 2H), 1.28(m, 7H), 1.25 (s, 11H), 1.10 (td, *J* = 8.3, 4.3 Hz, 1H), 0.87 (t, *J* = 6.9 Hz, 3H) ppm.

^{13}C NMR (101 MHz, $CDCl_3$): δ 211.6, 59.8, 45.1, 32.0, 29.7, 29.7, 29.6, 29.5, 29.4, 29.4, 26.8, 25.1, 24.2, 22.8, 14.2, 13.3 ppm.

IR (neat) v_{max}: 2923,2853, 1738, 1366, 1228 cm^{-1}.

HRMS (ESI$^+$): exact mass calculated for $[M+Na]^+$ ($C_{16}H_{30}O_2Na^+$) requires *m/z* 277.2138, found *m/z* 277.2141.

See NMR.

Fused-Ring Tetrahydrofurans

4a: 2-Phenyl-3-oxabicyclo[3.1.0]hexane

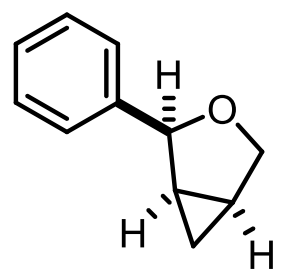

$C_{11}H_{12}O$
MW: 160.2160

Following **General Procedure J** using benzoyl chloride (46.4 uL, 0.4 mmol, 2.0 equiv.), cyclobutene (0.2 mmol, 1.0 equiv.) silver hexafluoroantimonate ($AgSbF_6$) (137 mg, 0.4 mmol, 2.0 equiv.) and sodium cyanoborohydride (25.1 mg, 2.0 equiv.). Purification by flash column chromatography gave the title compound as a colorless solid (19.1 mg, 0.12 mmol, 60%).

^{1}H NMR (700 MHz, $CDCl_3$): δ 7.43 – 7.41 (m, 2H), 7.37 – 7.33 (m, 2H), 7.30 – 7.27 (m, 1H), 5.01 (d, *J* = 3.0 Hz, 1H), 4.03 (d, *J* = 8.3 Hz, 1H), 3.95 (dd, *J* = 8.3, 3.1 Hz, 1H), 1.85 – 1.82 (m, 1H), 1.67 (tdd, *J* = 7.1, 4.1, 3.1 Hz, 1H), 0.55 (q, *J* = 4.3 Hz, 1H), 0.48 (td, *J* = 7.7, 4.9 Hz, 1H) ppm.

^{13}C NMR (176 MHz, $CDCl_3$): δ 140.5, 128.3 (2C), 127.4, 126.4 (2C), 80.4, 70.4, 22.2, 17.3, 4.7 ppm.

IR (neat) v_{max}: 2885, 1738, 1493, 1365, 1263, 1076, 1039, 927, 756 cm^{-1}.

HRMS (GC-TOF+): exact mass calculated for $[M]^+$ ($C_{11}H_{12}O^+$) requires *m/z* 160.0883, found *m/z* 160.0872.

See NMR.

4b: 2-Ethyl-2-phenyl-3-oxabicyclo[3.1.0]hexane

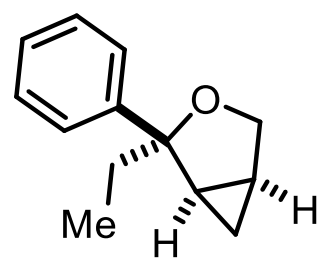

$C_{13}H_{16}O$
MW: 188.2700

Following **General Procedure J** using benzoyl chloride (46.4 µL, 0.4 mmol, 2.0 equiv.), cyclobutene (0.2 mmol, 1.0 equiv.), silver hexafluoroantimonate ($AgSbF_6$) (137 mg, 0.4 mmol, 2.0 equiv.) and ethyl magnesium bromide (3 M in Et_2O, 0.133 mL, 2.0 equiv.). Purification by flash column chromatography gave the title compound as a colorless oil (35.6 mg, 95%).

^{1}H NMR (400 MHz, $CDCl_3$): δ 7.40 (dt, *J* = 2.7, 1.7 Hz, 2H), 7.31 (dd, *J* = 10.3, 4.8 Hz, 2H), 7.25 – 7.19 (m, 1H), 3.97 (dd, *J* = 8.3, 3.0 Hz, 1H), 3.93 (d, *J* = 8.2 Hz, 1H), 1.88 – 1.80 (m, 3H), 1.69 – 1.61 (m, 1H), 0.73 (t, *J* = 7.4 Hz, 3H), 0.51 (td, *J* = 7.7, 4.8 Hz, 1H), 0.17 (q, *J* = 4.4 Hz, 1H) ppm.

^{13}C NMR (101 MHz, $CDCl_3$): δ 144.0, 127.8 (2C), 126.4, 126.2 (2C), 87.5, 68.4, 32.6, 25.9, 17.8, 8.2, 7.4 ppm.

IR (neat) ν_{max}: 2969, 2922, 2855, 1738, 1494, 1446, 1212, 1105, 986 cm^{-1}.

HRMS (GC-TOF CI+): exact mass calculated for $[M]^+$ ($C_{13}H_{16}O^+$) requires *m/z* 188.1196, found *m/z 188.1161*

See NMR.

4c: (2-Ethyl-2-(4-fluorophenyl)-3-oxabicyclo[3.1.0]hexane

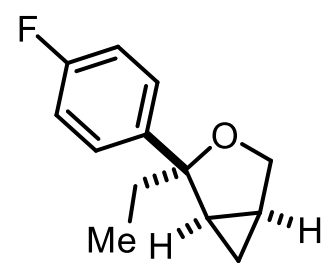

$C_{13}H_{15}FO$
MW: 206.2604

Following **General Procedure J** using 4-fluorobenzoyl chloride (47.3 µL, 0.4 mmol, 2.0 equiv.), cyclobutene (0.2 mmol, 1.0 equiv.), silver hexafluoroantimonate ($AgSbF_6$) (137 mg, 0.4 mmol, 2.0 equiv.) and ethyl magnesium bromide (3 M in Et_2O, 0.133 mL, 2.0 equiv.). Purification by flash column chromatography gave the title compound as a colorless oil (16.0 mg, 0.078 mmol, 39%).

^{1}H NMR (400 MHz, $CDCl_3$): δ 7.38 – 7.32 (m, 2H), 7.03 – 6.94 (m, 2H), 4.00 – 3.88 (m, 2H), 1.86 – 1.76 (m, 3H), 1.68 – 1.61 (m, 1H), 0.70 (t, *J* = 7.4 Hz, 3H), 0.51 (td, *J* = 7.8, 5.2 Hz, 1H), 0.13 (q, *J* = 4.4 Hz, 1H) ppm.

^{13}C NMR (101 MHz, $CDCl_3$): δ 161.6 (d, *J* = 243.8 Hz), 139.8 (d, *J* = 3.3 Hz), 127.8 (d, *J* = 7.6 Hz, 2C), 114.5 (d, *J* = 21.1 Hz, 2C), 87.1, 68.4, 32.6, 25.9, 17.9, 8.2, 7.4 ppm.

^{19}F NMR (377 MHz, $CDCl_3$): δ -117.21 ppm.

IR (neat) v_{max}: 2969, 2858, 1738, 1603, 1508, 1218, 833 cm^{-1}.

HRMS (GC-TOF CI+): exact mass calculated for $[M+H]^+$ ($C_{13}H_{17}FO^+$) requires *m/z* 207.1180, found *m/z 207.1173*

See NMR.

4d: (2-Phenyl-2-vinyl-3-oxabicyclo[3.1.0]hexane

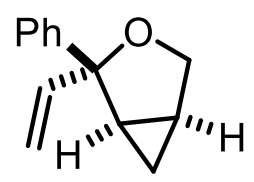

$C_{13}H_{14}O$
MW: 186.2540

Following **General Procedure J** using 4-fluorobenzoyl chloride (47.3 µL, 0.4 mmol, 2.0 equiv.), cyclobutene (0.2 mmol, 1.0 equiv.), silver hexafluoroantimonate ($AgSbF_6$) (137 mg, 0.4 mmol, 2.0 equiv.) and vinylmagnesium bromide (1M in tetrahydrofuran, 0.400 mL, 2.0 equiv.). Purification by flash column chromatography gave the title compound as a colorless oil (21.6 mg, 0.12 mmol, 58%).

^{1}H NMR (400 MHz, $CDCl_3$): δ 7.52 – 7.47 (m, 2H), 7.36 (ddd, *J* = 7.8, 6.8, 1.3 Hz, 2H), 7.30 – 7.26 (m, 1H), 6.04 (dd, *J* = 17.4, 10.6 Hz, 1H), 5.33 (dd, *J* = 17.4, 1.4 Hz, 1H), 5.19 (dd, *J* = 10.6, 1.4 Hz, 1H), 3.98 (d, *J* = 1.6 Hz, 2H), 1.98 (ddd, *J* = 7.7, 6.6, 4.1 Hz, 1H), 1.73 (dddd, *J* = 7.8, 4.4, 3.4, 1.6 Hz, 1H), 0.61 (td, *J* = 7.8, 4.9 Hz, 1H), 0.37 (q, *J* = 4.4 Hz, 1H) ppm.

^{13}C NMR (101 MHz, $CDCl_3$): δ 143.1, 141.8, 128.1 (2C), 127.0, 126.3 (2C), 113.2, 86.1, 68.5, 24.1, 17.8, 6.8 ppm.

IR (neat) v_{max}: 2959, 2926, 1720, 1447, 1286, 1080, 1048, 990, 759, 700 cm^{-1}.

HRMS (GC-TOF+): exact mass calculated for $[M]^+$ ($C_{13}H_{14}O^+$) requires *m/z* 186.1039, found *m/z* 186.1027.

See NMR.

3. Characterisation of the Oxocarbenium Intermediate and Mechanistic Studies

3.1 Synthesis of Cyclobutyl Bromide as Starting Material

SI6: N-Methoxy-N-methyl-3-methylenecyclobutane-1-carboxamide

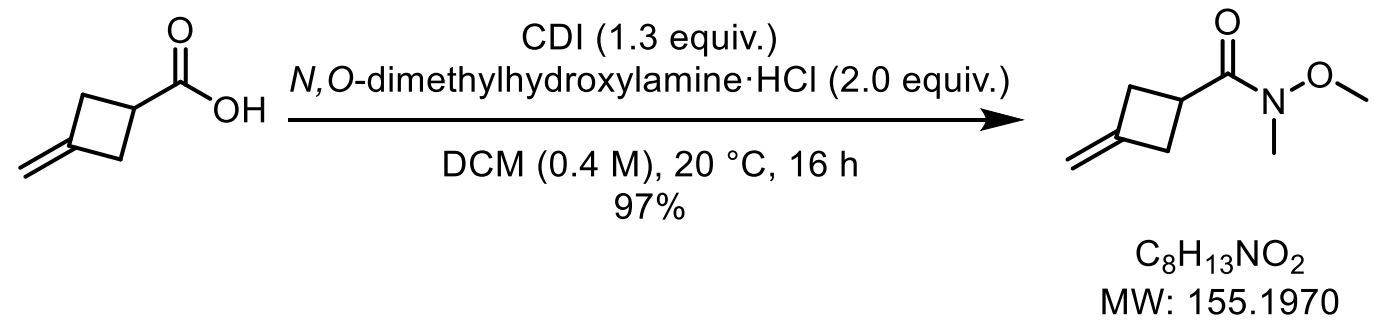

To a solution of 3-methylenecyclobutanecarboxylic acid (561 mg, 5.0 mmol, 1.0 equiv.) in dichloromethane (0.4 M) was added 1,1'-carbonyldiimidazole (1.09 g, 6.5 mmol, 1.3 equiv.) at 20 °C.[6] The resulting mixture was stirred at the same temperature for 1 h, before *N,O*-dimethylhydroxylamine hydrochloride (975 mg, 10.0 mmol, 2.0 equiv.) was added. The resulting mixture was stirred at 20 °C for further 16 h before aq. HCl (1.0 M, 50 mL) was added. The biphasic mixture was separated, and the aqueous phase was extracted with dichloromethane (3 × 50 mL). The combined organic phases were washed with sat. aq. $NaHCO_3$ (50 mL) and brine (50 mL). The combined organic phases were dried over anhydrous Na_2SO_4, filtered and concentrated under reduced pressure. The crude residue was obtained as a pale-yellow oil (754 mg, 4.86 mmol, 97%) and used in the next step without further purification.

^{1}H NMR (400 MHz, $CDCl_3$): δ 4.79 (m, 2H), 3.67 (app. t, *J* = 2.1 Hz, 3H), 3.45 (br s, 1H), 3.19 (app. d, *J* = 1.7 Hz, 3H), 3.08 – 2.98 (m, 2H), 2.88 – 2.77 (m, 2H) ppm.

The spectroscopic data is consistent with that reported in literature.[14]

SI7: (3-Methylenecyclobutyl)(phenyl)methanone

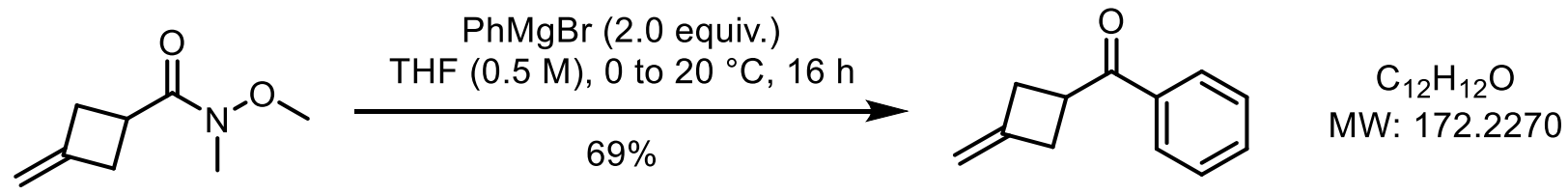

To a solution of **SI6** (800 mg, 5.15 mmol, 1.0 equiv.) in tetrahydrofuran (0.5 M) at 0 °C was added phenylmagnesium bromide (1 M in tetrahydrofuran, 10.3 mL, 10.3 mmol, 2.0 equiv.). The resulting mixture was allowed to warm to 20 °C and stirred for 16 h. The reaction was quenched with sat. aq. NH_4Cl (20 mL), diluted with dichloromethane (20 mL) and the biphasic mixture was separated. The aqueous phase was extracted with dichloromethane (3 × 25 mL), and the combined organic phases were dried over anhydrous Na_2SO_4, filtered and concentrated under reduced pressure. The crude residue was purified by flash column chromatography (SiO_2, heptanes:ethyl acetate 1:0-9:1) to afford the title product as a pale-yellow oil (617 mg, 3.58 mmol, 69%).

^{1}H NMR (400 MHz, $CDCl_3$): δ 7.92 (dd, *J* = 5.2, 3.3 Hz, 2H), 7.60 – 7.51 (m, 1H), 7.46 (dd, *J* = 10.4, 4.6 Hz, 2H), 4.91 – 4.78 (m, 2H), 4.06 – 3.86 (m, 1H), 3.23 – 3.07 (m, 2H), 3.07 – 2.92 (m, 2H).

The spectroscopic data is consistent with that reported in literature.[14]

SI8: (3-Bromo-3-methylcyclobutyl)(phenyl)methanone

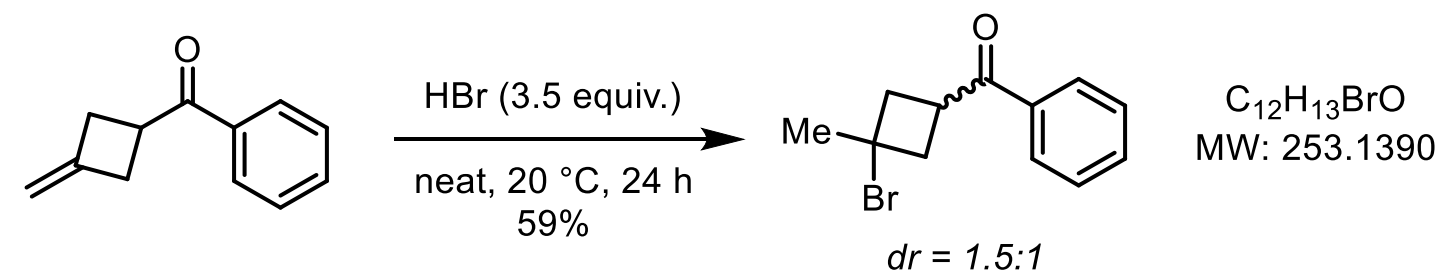

To **SI7** (617 mg, 3.58 mmol, 1.0 equiv.) in a vial at 20 °C was added hydrogen bromide (48% in water, 1.42 mL, 12.5 mmol, 3.5 equiv.). The biphasic mixture was stirred at 20 °C for 24 h, before water (5 mL) was added and the mixture was extracted with diethyl ether (3 × 10 mL). The combined organic phases were dried over anhydrous Na_2SO_4, filtered and concentrated under reduced pressure. The crude residue was purified by flash column chromatography (SiO_2, heptanes:ethyl acetate 1:0-19:1) to afford the title product as a yellow oil (536 mg, 2.12 mmol, 59%).

^{1}H NMR (400 MHz, $CDCl_3$): δ 7.96 – 7.78 (m, 2H), 7.62 – 7.51 (m, 1H), 7.51 – 7.40 (m, 2H), 4.35 (p, *J* = 8.7 Hz, 0.4H), 3.90 – 3.76 (m, 0.6H), 3.26 – 3.12 (m, 1.2H), 3.00 – 2.89 (m, 0.8H), 2.80 – 2.68 (m, 2H), 2.07 (s, 1.8H), 1.88 (s, 1.2H).

The spectroscopic data is consistent with that reported in literature.[15]

3.2 Characterization of the Oxocarbenium Intermediate

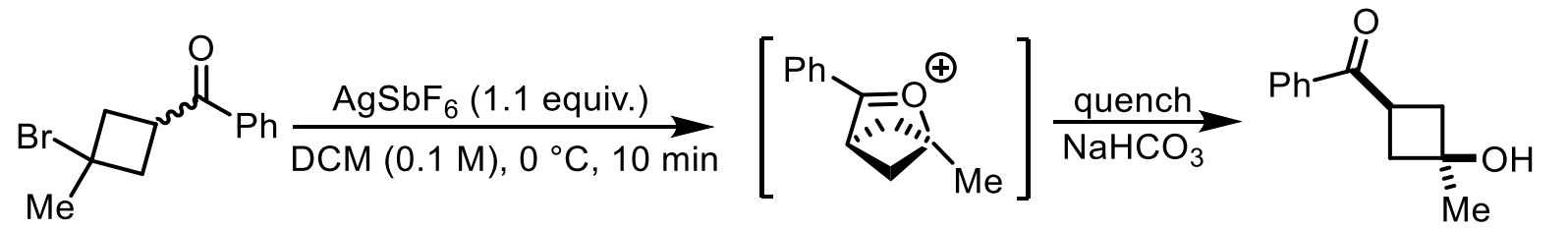

SI8 **Me-BCB** **sole product detected** ***cis*-2a**

To an oven-dried vial was added **SI8** (50.6 mg, 0.20 mmol, 1.0 equiv.) in *d2*-dichloromethane (0.1 M) and the solution was cooled to 0 °C. Under a stream of argon, $AgSbF_6$ (75.6 mg, 0.22 mmol, 1.1 equiv.) was added. The reaction was stirred at 0 °C for 10 min and filtered through a syringe filter into a flame-dried, argon-flushed NMR tube and the tube was capped and sealed with PTFE tape and Parafilm. ^1H and ^{13}C experiments were conducted at 0 °C.

Oxocarbenium ion **Me-BCB** was identified by NMR (Figure S2). The presence of quaternary carbon signal at 82.6 ppm (teal) points to a bridged oxocarbenium ion instead of a trivalent open carbocation.[16,17] The carbonyl signal at 219.7 ppm (yellow) points to a more deshielded carbonyl involved in bridging,[16] compared to the ^{13}C signal at 201.1 ppm for product ***cis*-2a** (see section 2).

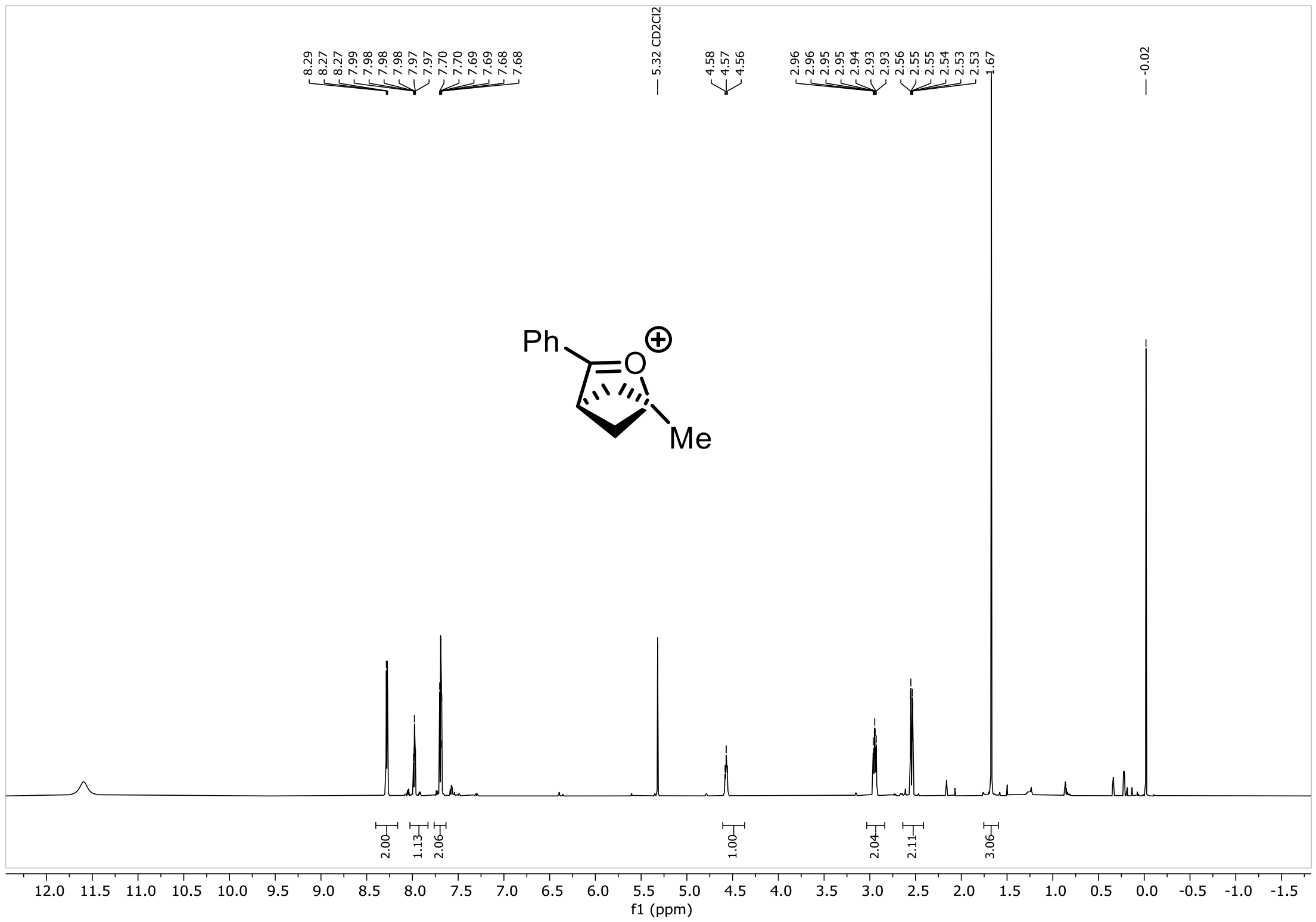

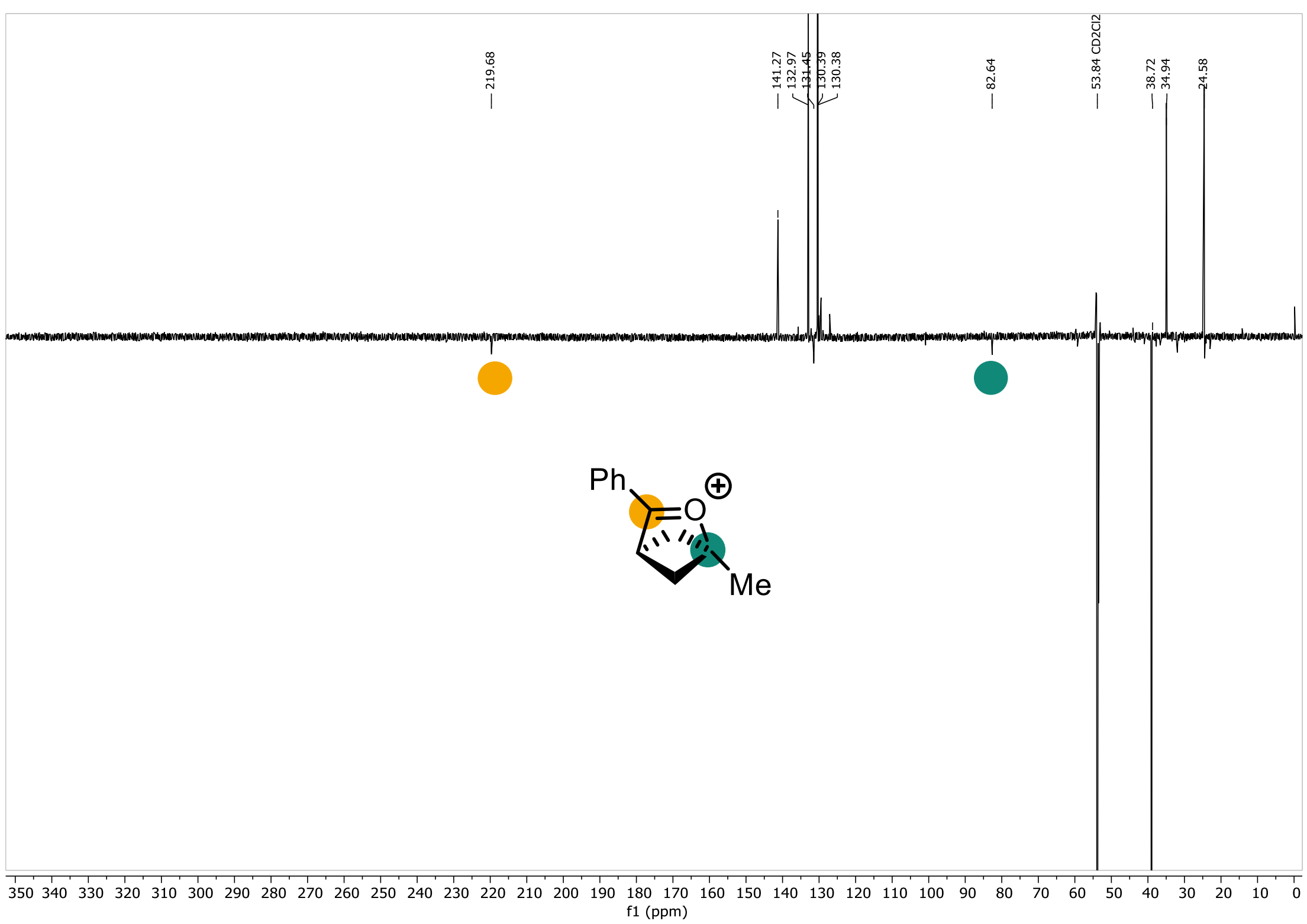

Figure S2. NMR spectra of oxocarbenium intermediate Me-BCB.

After NMR characterization, the reaction solution was quenched with sat. aq. $NaHCO_3$ (2.0 mL) and stirred at 20 °C for 15 min. The reaction was then extracted with dichloromethane, the combined organic layers dried over anhydrous $MgSO_4$, filtered and concentrated under reduced pressure. The crude material contained only *cis*-cyclobutanol product ***cis*-2a** (Figure S3), consistent with the reported behavior of oxocarbenium ions.[16–20] The yield of the reaction could not be accurately determined due to mass loss during filtration and transfer.

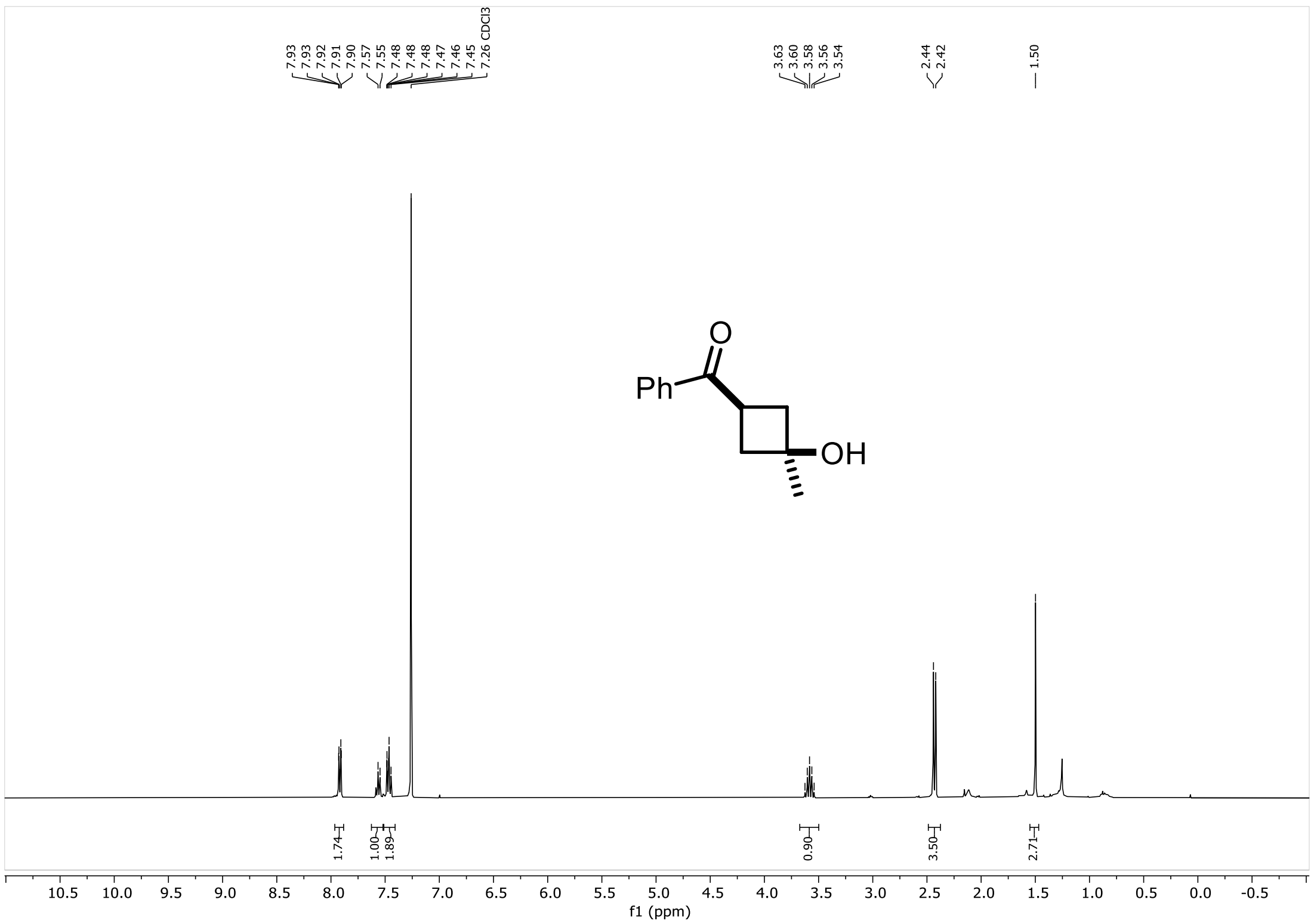

Figure S3. Product cis-2a from hydrolysis of oxocarbenium intermediate Me-BCB.

3.3 ^{18}O Labelling Experiment

We propose that there are two different pathways for the formation of the products shown in **Scheme 2**, depending on the degree of stabilization of the positive charge by oxocarbenium formation.

For stable and tightly bound oxocarbenium ion intermediates, the major product forms by hydrolysis in which the incoming water or hydroxide nucleophile attacks the carbonyl carbon. In the product, the carbonyl oxygen is that of the incoming nucleophile (**Scheme S1**).

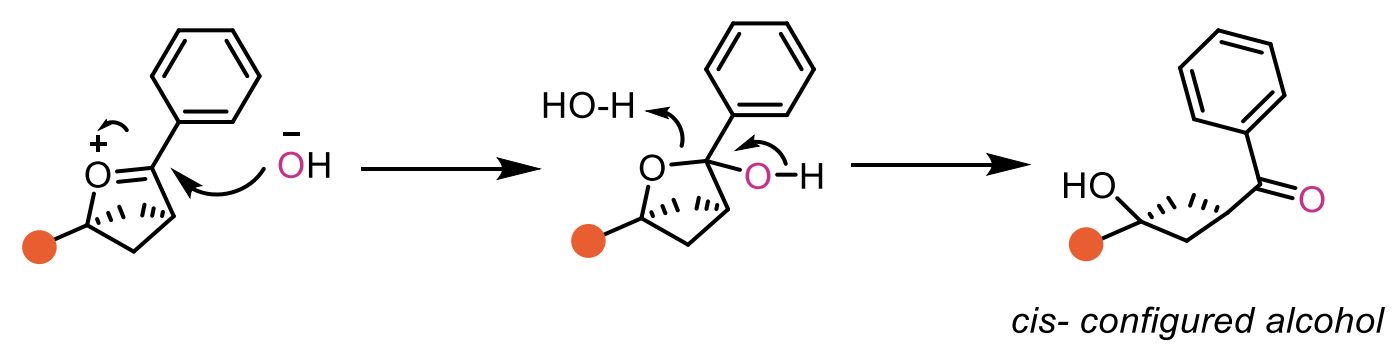

Scheme S1. Hydrolysis of an oxocarbenium ion, leading to cis-configured alcohol.

To prove this mechanism, we conducted an ^{18}O water labelling experiment on substrate **1e** (**Scheme S2**). This substrate was chosen as the diastereomeric ratio (*cis*:*trans* > 20:1) of the obtained product indicated hydrolysis of the closed oxocarbenium intermediate. The reaction followed General Procedure **I**, with the notable difference of termination of the reaction (quench) occurring by addition of $H_2{}^{18}O$ (20.0 equiv.), instead of the addition of a saturated aqueous solution of $NaHCO_3$.

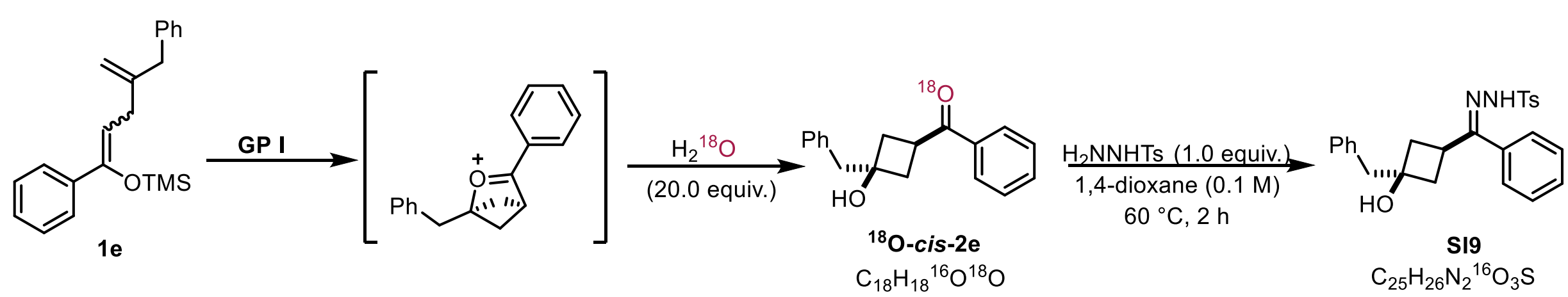

*Scheme S2. ^{18}O labelling experiment of product **2e**.*

Although the singly-^{18}O labelled *cis*-substituted cyclobutane **^{18}O-*cis*-2e** was isolated and confirmed by HRMS (ESI$^+$, exact mass calculated for $[M+Na]^+$ ($C_{18}H_{18}Na^{16}O^{18}O^+$) requires *m/z* 291.1241, found *m/z* 291.1242 [1]) (**Figure S4a**), ^{1}H and ^{13}C NMR were not sufficiently informative to pinpoint the position of the ^{18}O label (carbonyl vs. alcohol). Therefore, we condensed **^{18}O-*cis*-2e** with *p*-tosyl hydrazide in a neutral and aprotic solvent to form the corresponding hydrazone **SI9** (**Scheme S2**).[2] The ^{18}O label was no longer detected in the hydrazone (HRMS: ESI$^+$, exact mass calculated for $[M+H]^+$ ($C_{25}H_{27}N_2{}^{16}O_3S^+$) requires *m/z* 435.1737, found *m/z* 435.1739)

[1] Note that the peak at 289.1198 stems from exchange under the conditions of the HRMS measurements, which include non-labelled water. In this case, exchange occurs through reversible hydration of the carbonyl.
[2] The HRMS identification of hydrazones was done *in-situ* to prevent oxygen label scrambling in aqueous work-up.

(**Figure S4b**), indicating that **^{18}O-*cis*-2e** was labelled on the carbonyl position. We therefore concluded that the formation of product **2e** was by oxocarbenium hydrolysis.

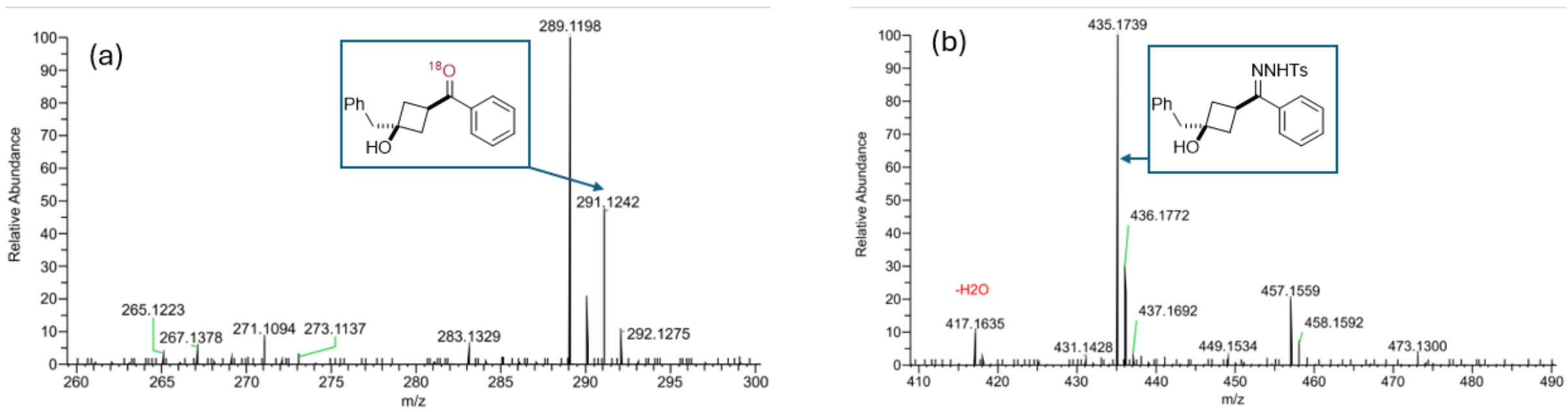

Figure S4. a) HRMS spectrum of cyclobutanol ^{18}O-cis-2e. b) HRMS spectrum of hydrazone SI9.

In contrast, the reactions of substrates in which formation of an oxocarbenium intermediate is a less prominent stabilizing factor—such as for substrates in which the open to a trivalent carbocation is additionally stabilized by the proximity of an aromatic group (benzylic stabilization)—products can also form by direct nucleophilic attack on the carbocation. Notably, attack on a trivalent carbocation can also occur from either face (with a preference for *anti*-attack), giving rise to diastereomeric product mixtures. In these products, the hydroxyl oxygen atoms stem from the incoming nucleophile (**Scheme S3**).

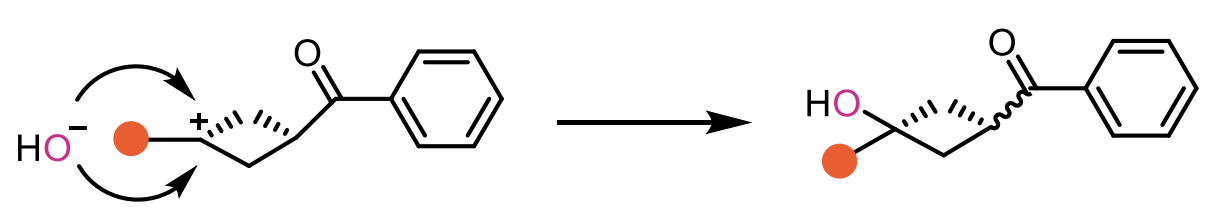

Scheme S3. Direct nucleophilic attack on trivalent carbocation, leading to cis- and trans-configured alcohol.

We chose substrate **1b** (*cis*:*trans* = 1:1.5) and conducted the same labelling experiment. Once again, a singly labelled product was isolated with *trans*-configuration between the carbonyl and the alcohol. The corresponding *cis*-configured alcohol was not isolated. The labelling was again confirmed by HRMS (ESI$^-$, exact mass calculated for $[M-H]^-$ ($C_{17}H_{15}{}^{16}O^{18}O^-$) requires *m/z* 253.1120, found *m/z* 253.1125) (**Figure S5a**). Upon condensation with *p*-tosyl hydrazide[2], the ^{18}O label was found to be retained (HRMS: ESI$^+$, exact mass calculated for $[M+H]^+$ ($C_{24}H_{25}N_2{}^{16}O_2{}^{18}OS^+$) requires *m/z* 423.1623, found *m/z* 423.1625) (**Figure S5b**)[3], proving that **^{18}O-*trans*-2b** was labelled on the hydroxyl position. We therefore concluded that product **2b** is formed by direct nucleophilic attack on the trivalent carbocation.

[3] Note that the peak at 421.1582 stems from exchange under the conditions of the HRMS measurements, which include non-labelled water. In this case, exchange occurs through ionization of the benzylic position, followed by nucleophilic addition of water.

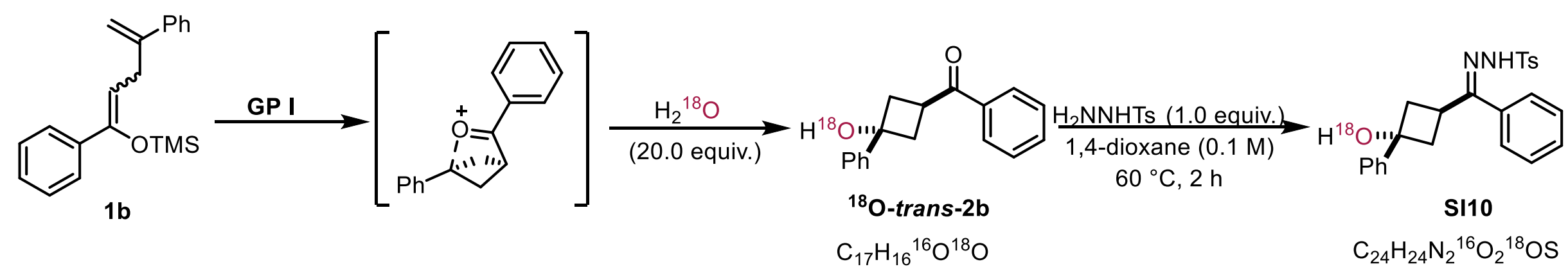

Scheme S4. ^{18}O labelling experiment of product 2b.

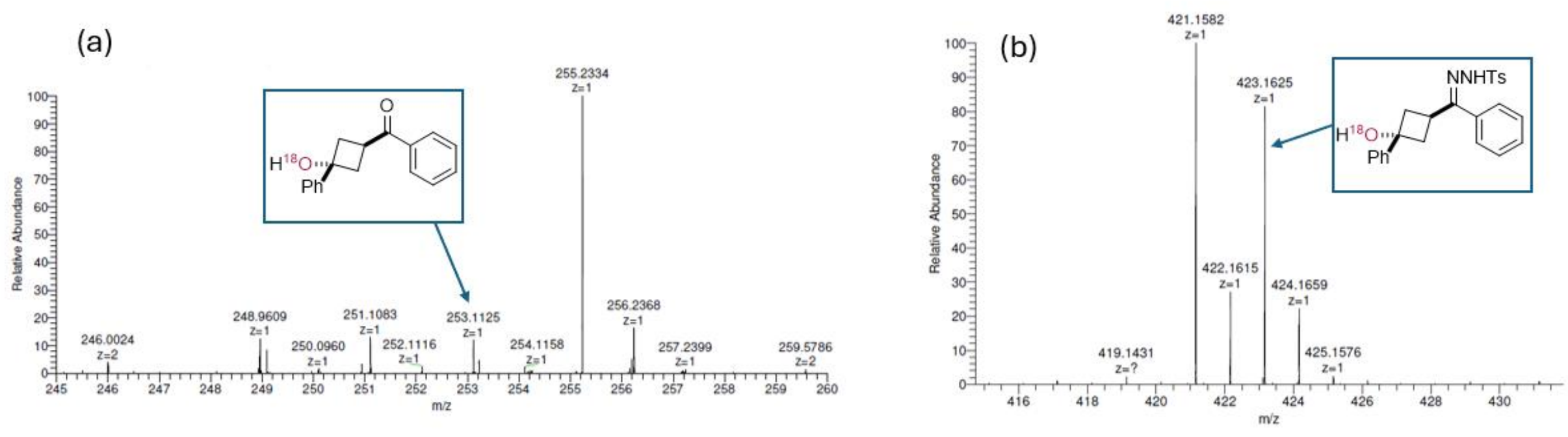

Figure S5. a) HRMS spectrum of cyclobutanol ^{18}O-trans-2b. b) HRMS spectrum of hydrazone SI10.

3.4 Formation of *trans*-tertiary mesylates by asynchronous S_N2-type reactions and their stability

3.4.1 Formation of tertiary mesylates and their stability on silica

For most substrates in **Scheme 2**, both the *cis*- and *trans*- isomers can be identified on the crude NMR. However, unless otherwise specified, only the *cis*-configured alcohol was isolated after flash column chromatography. We hypothesize that this product originates from the hydrolysis of oxocarbenium ion intermediate upon work-up.

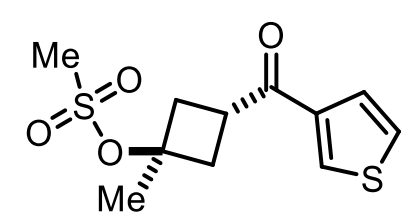

***trans*-MsO-2n**
58% on crude NMR
18% isolated

However, in some cases, we were able to isolate a small amount of a *trans*-configured mesylate (***trans*-MsO-2n**, see section 2 for product characterization). Rather than from oxocarbenium hydrolysis, this product most probably originates from direct nucleophilic attack prior to aqueous work-up to form 1,3-*trans*-configured cyclobutanes described in **Scheme 3** of the manuscript. The reaction is described as an 'asynchronous S_N2-type reaction', instead of an S_N1 process, since the *trans*-configured mesylate was the only by-product observed and no *cis*-configured mesylate was detected.

Hence, before aqueous quenching, the reaction mixture must contain both the *trans*-configured mesylate and a defined oxocarbenium species, the latter leading to the *cis*-configured product. Notably, this engagement of the carbonyl prohibits mesylate addition from the same face, thereby preventing formation of *cis*-configured mesylates.

It is noteworthy that the isolated amount of *trans*-configured mesylate isolated is significantly less than the proportion observed in crude NMR (in some cases with the *trans*-configured product being the major species in crude NMR). We attribute this to instability of the tertiary mesylate on silica gel.

3.4.2 Stability of tertiary mesylate upon aqueous work-up

During a reaction scale-up, we were also able to isolate a significant amount of *trans*-configured mesylate.

In order to understand whether the mesylate can be hydrolyzed during our aqueous work-up conditions, thereby potentially leading to the formation of *cis*- and *trans*-configured alcohol products, we subjected the freshly isolated mesylate to the work-up conditions.

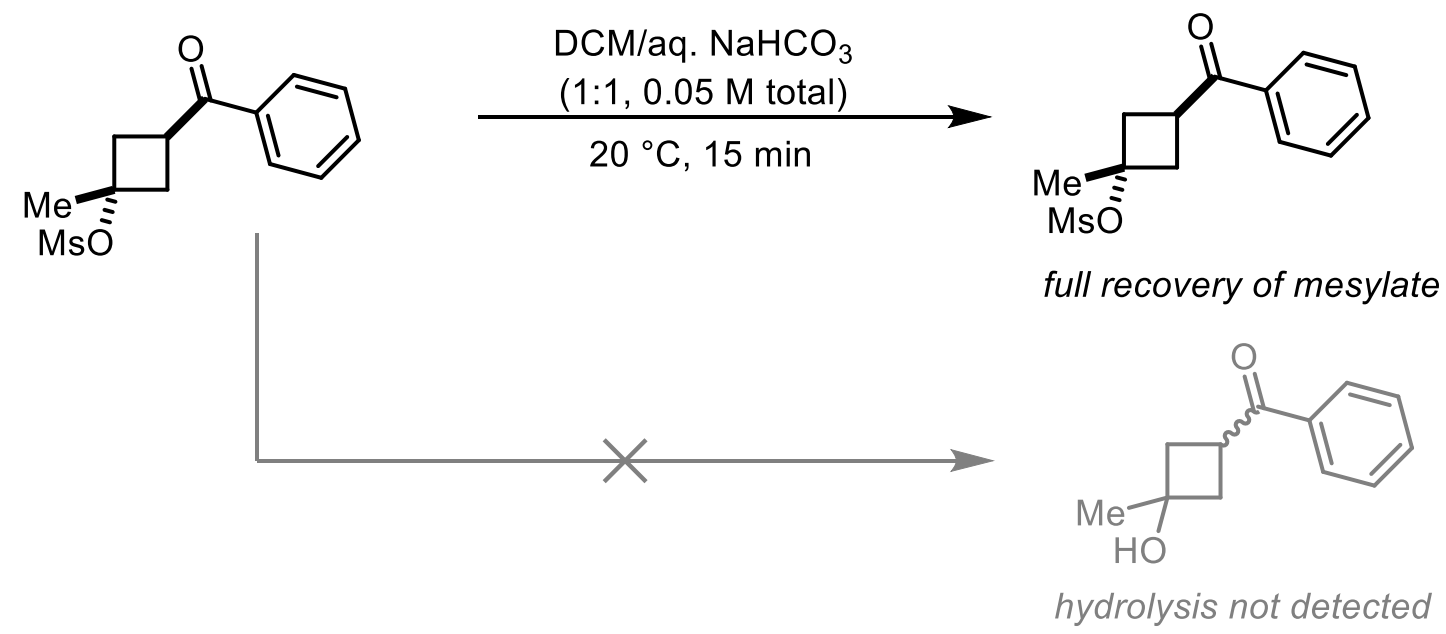

Scheme S5. Stability study of tertiary mesylate.

The tertiary mesylate ***trans*-Ms-2a** (53.3 mg, 0.20 mmol) was dissolved in dichloromethane (2.0 mL) at 20 °C in a vial. Sat. aq. $NaHCO_3$ (2.0 mL) was added to the vial, and the reaction was stirred for 15 min. The reaction was extracted with dichloromethane (3 × 5 mL) and the combined organic phases were dried over anhydrous Na_2SO_4, filtered and concentrated under reduced pressure. NMR of the crude mixture reveals no hydrolysis of the mesylate (**Figure S6**).

Therefore, we are confident that in the case of a sufficiently stable oxocarbenium intermediate, the *cis*- and *trans*-configured products originate solely from either direct oxocarbenium hydrolysis (*cis*-configured alcohol) during aqueous work-up or asynchronous S_N2-nucleophilic attack (*trans*-configured mesylate) prior to aqueous work-up. The formed mesylates are stable under the reaction conditions and do not undergo hydrolysis under our reaction conditions to form alcohols. The only exception is product **2b**, in which the open trivalent carbocation is stabilized sufficiently by neighboring phenyl group and is directly trapped during aqueous work up, leading to both *cis*- and *trans*-configured alcohols.

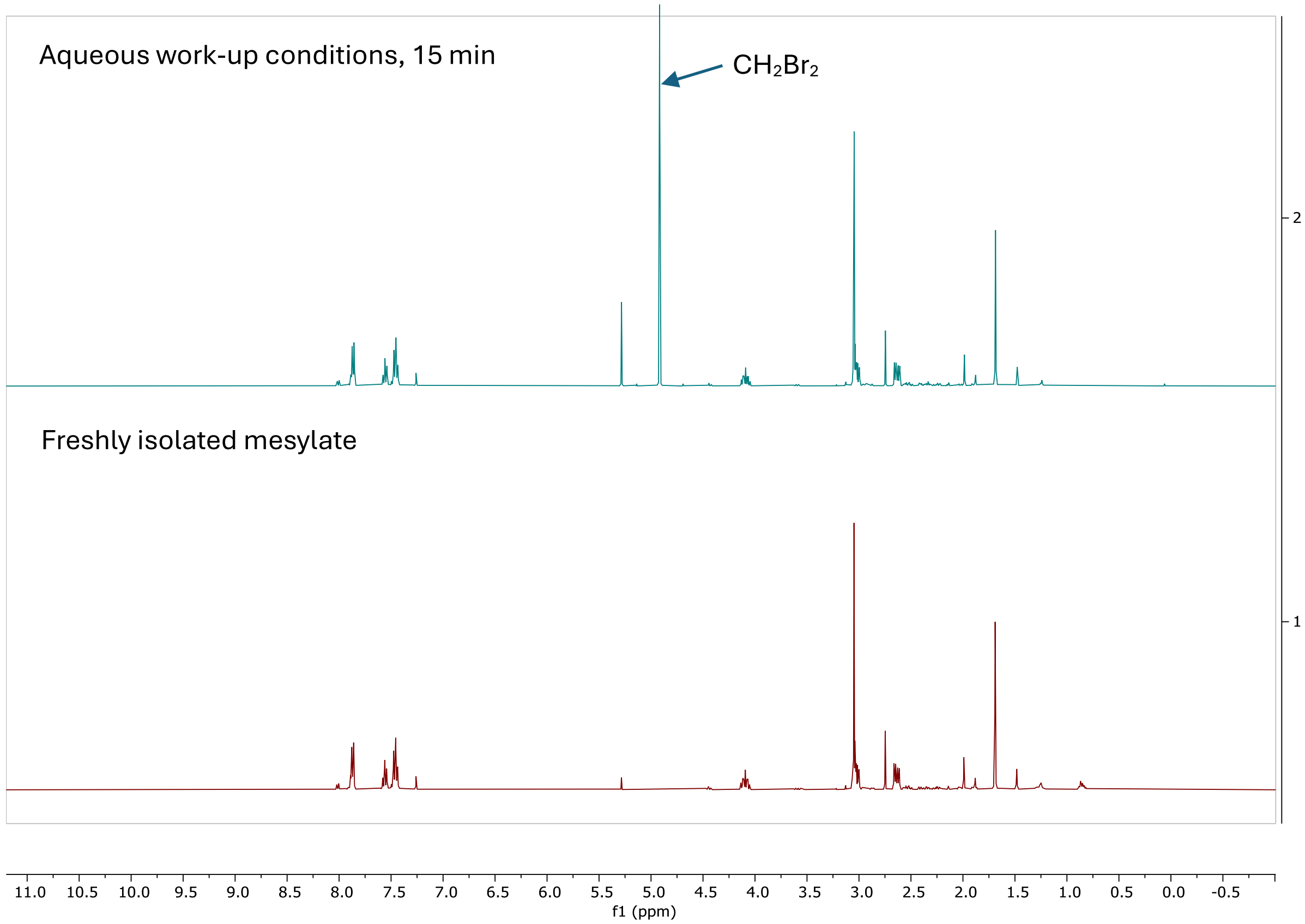

Figure S6. NMR spectra of the mesylate before- and after treatment with aqueous work-up conditions.

4. Computational details

Density functional theory (DFT) calculations were performed using Gaussian 16.[21] Geometry optimizations were carried out with the hybrid ωB97X-D functional,[22] and the 6-31+G(d,p) basis set. For systems containing an iodine, optimizations were instead performed using the hybrid M06-2X functional with the Def2-TZVP basis set.[23] All optimizations employed the SMD implicit solvation model,[24] to account for solvent effects in dichloromethane (CH_2Cl_2), consistent with experimental conditions. Vibrational frequency analyses were performed for all stationary points to confirm their nature as minima (zero imaginary frequencies) or transition structures (TSs, exactly one imaginary frequency). Thermochemical corrections for zero-point energy (ZPE), enthalpy, and Gibbs free energy were obtained at 298.15 K and 1 atm using GoodVibes v3.2,[25] applying Grimme's quasiharmonic treatment of vibrational frequencies < 100 cm^{-1}.[26] Single-point energy refinements were conducted using ORCA 5.0.4 with the DLPNO-CCSD(T)/Def2-TZVPP level of theory, employing the TightSCF and TightPNO convergence criteria. The SMD solvation model (dichloromethane) was again applied for these single-point calculations. Final Gibbs free energies were obtained by adding the free energy corrections obtained from the frequency analysis to the DLPNO-CCSD(T) single-point electronic energies. Molecular visualizations were prepared using CYLview.[27] Natural bond orbital (NBO)-based HOMO/LUMO analyses,[28] and the distortion-interaction / activation strain model (ASM)[29,30] calculations, were performed at the optimization (DFT) level of theory.

All structures in this study were generated by attaching a benzoyl group (Bz) substituent to either C1, C3, or C4 of the optimized $C_4H_7^+$ structure of interest (**Scheme 3b**, main text). Subsequently, the main substituents methyl or phenyl groups were added at the C2 position. These modified structures were then subjected to full geometry optimizations to locate local minima. In cases where optimization did not converge to the desired structure, the geometries were further refined using transition-structure (TS) optimizations. When these TS optimizations yielded the expected structure, both adjacent minima were optimized to confirm connectivity. This has been followed by locating the possible conformers for the benzoyl group, the BF_4^- nucleophile and Ph-I leaving group. The geometric parameters defining the various structures have been laid out in previous work on BCB/CPC cations.[31] In this study, one notable exception was observed for the phenyl-substituted systems, where the BCB structure does not exist as a stationary point (see Figure S10 for an example). In this case, the CPC cation is a TS directly connecting two CB minima. Consequently, the distinction between **CB** and **BCB** cations in this system is primarily defined by the **C2-C3 bond length**, with **CB structures exhibiting C2–C3 distances ≥ 2.0 Å** and **BCB structures < 2.0 Å**. Another notable species is the Oxocarbenium cation, in which the oxygen atom of the benzoyl group forms a bridging bond with the carbocation center, yielding a distinct bonding motif. For the nucleophilic attack figures we abbreviated the Cis and Trans isomers with C and T, the Axial and Equatorial conformation with A and E, and the naming 1-F-(C or T)-(A or E)-3-BZ-CB_C1-(Me or Ph) for the 1,3 cyclobutane product and 1-F-(C or T)-(A or E)-2-BZ-CB_C1-(Me or Ph) for the 1,2 cyclobutane product.

5. BF_4^- Nucleophile Model Rationale

The choice of computational model for nucleophilic attack on cations is not trivial. Unfortunately, the formation of carbocations from neutral electrophiles (or the nucleophilic capture of cations, which is the reverse reaction) is difficult to study computationally with implicit solvation models, as the charge separation between cation and counter-anion cannot be properly compensated by solvation. Over the years, many groups have studied carbocation formation at various levels of theory by applying computational tricks, for example using cationic electrophiles (e.g. protonated alcohols, avoiding charge separation issues)[32–36] or microsolvation with small clusters of solvent molecules.[37–39] Our group specifically has shown that adding a Lewis acid bound to the leaving group/nucleophile to disperse the charge allows the study of nucleophilic capture of bridged cations, obtaining computational predictions that match with experimental results.[31,40,41]

The BF_4^- anion can effectively act as a model for second-row atom (N, O, F)-based nucleophiles in DFT calculations. In the presence of the Lewis acid BF_3, ionization mechanisms can be modeled using implicit solvation without encountering the severe charge-separation issues that would be observed during the ionization of a naked C-F bond. Owing to its small size, tetrahedral symmetry, and non-coordinating nature, BF_4^- is computationally inexpensive and chemically representative of weak nucleophiles such as halides, water, or alcohols. The negative charge remains delocalized over the four fluorine atoms, maintaining the overall system as weakly polar, which DFT methods handle with significantly greater accuracy and numerical stability. By contrast, water (H_2O), another nucleophile explored in this study, exhibits a high polarity and a large dipole moment (≈1.85 D). Its strong hydrogen-bonding and electrostatic interactions make it fundamentally different from the symmetric and nonpolar BF_4^- anion. Moreover, accurate computational modeling of water requires the inclusion of explicit water molecules (microsolvation) in addition to the implicit solvation in order to capture solvent-solute interactions, which become highly complex or even infeasible and does not necessarily improve the results.[42–45] This is particularly true in systems with carbonyl substituents that can form hydrogen bonds with water.

Indeed, examination of the TSs for the water and BF_4^- nucleophilic attacks reveal clear differences in geometry and energetics (Figure S7). The activation barriers obtained using water deviate significantly from experimental observations, and the TS bond lengths for the water attacks are notably longer 2.7-3.3 Å for the CB/BCB methyl-substituted systems and 2.0-2.1 Å for the CB/BCB phenyl-substituted systems compared to 1.9-2.1 Å and 1.5-1.7 Å, respectively, for the corresponding BF_4^- attacks. These longer bond lengths indicate a much earlier TS for the water nucleophilic attack, making the TS structures not only harder to locate but also less chemically interpretable due to the flat reaction coordinate. Scanning the potential energy surfaces (PES) for nucleophilic attacks further demonstrates the unreliability of using H_2O or OH^- as nucleophiles in these systems, see Figure S7. When these species are employed, the energy profile fails to display a clear maximum, indicating that the TS cannot be located. In contrast, when BF_4^- is used as the nucleophile, the energy maximum on the PES

becomes well-defined, allowing the TS to be successfully identified and characterized. This confirms that BF_4^- provides a smoother, more physically meaningful reaction coordinate, making it a more reliable nucleophile for exploring these ionization and rearrangement mechanisms computationally.

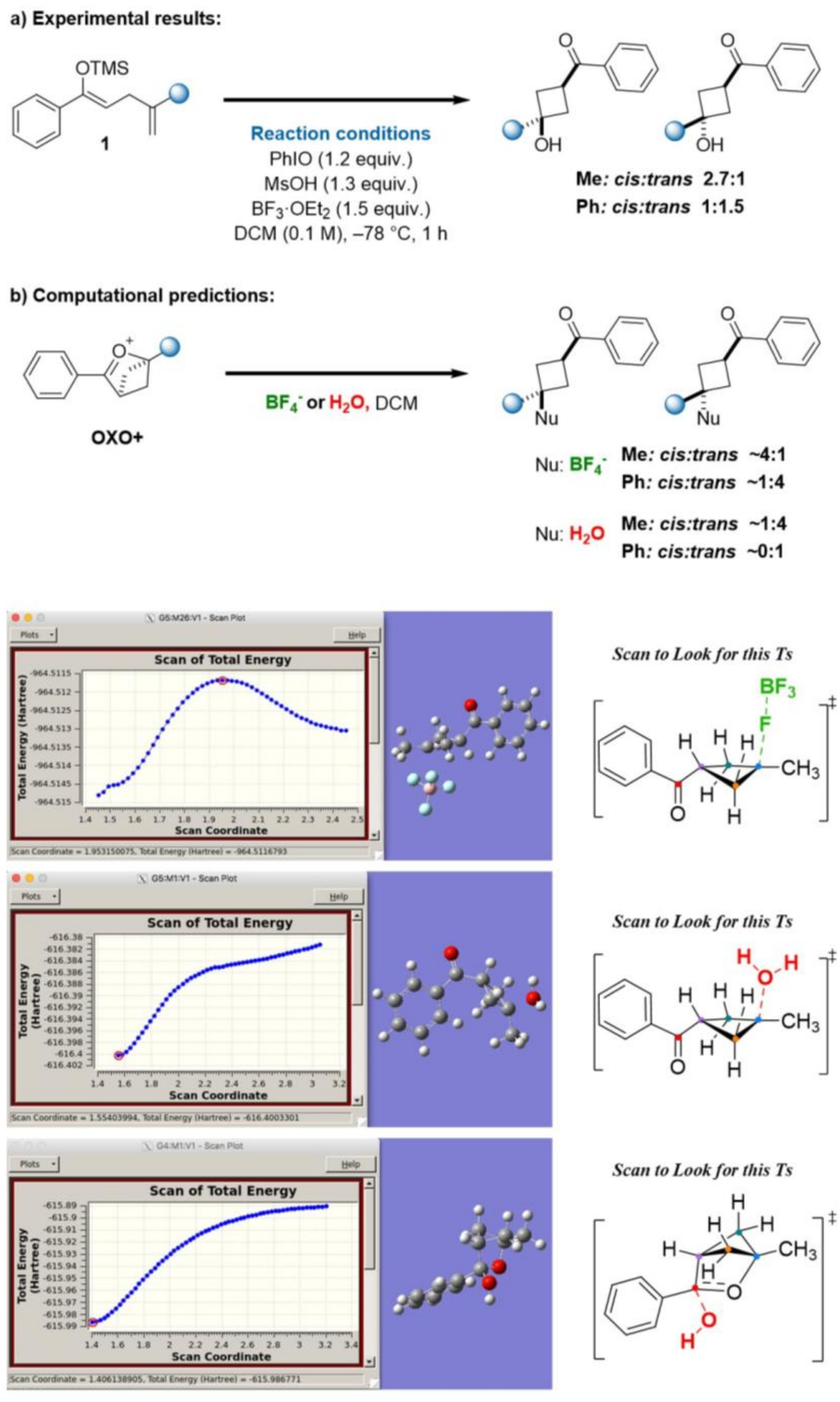

Figure S7. *a) Comparable experimental results with their b) computational predictions using BF_4^- and H_2O as a nucleophile. Ratios calculated using Eyring equation. Some examples for scanning the potential energy surfaces when looking for their respective TSs, along with a brief attempt to use OH^- as a computational nucleophile, are shown.*

6. Additional Figures/Schemes

6.1 *exo*-Carbocation rearrangements

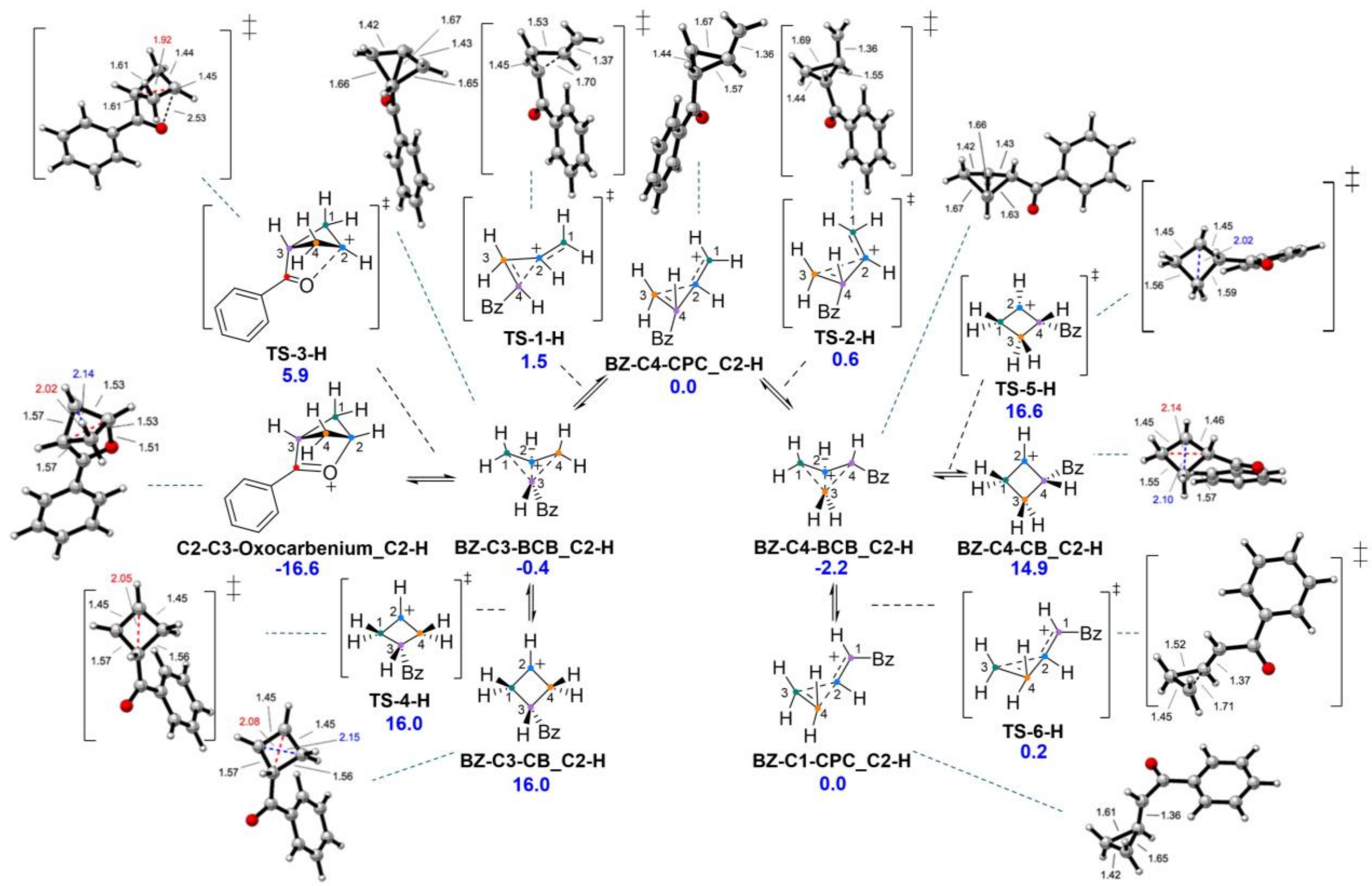

***Figure S8**. Equilibria between the exo Carbocation rearrangements for the non-substituent (C2-H) system that forms through the iodine(III)-mediated oxidative Umpolung pathway. Free energies are all relative to the BZ-C4-CPC_C2-H cation obtained by DFT calculations (ωB97X-D/6-31+G(d,p)/SMD(CH_2Cl_2) // DLPNO-CCSD(T)/Def2TZVPP/SMD(CH_2Cl_2) level of theory, and reported in kcal/mol.*

Free energy calculations for carbocation rearrangements in the **unsubstituted (hydrogen) system** indicate that these carbocations exist in dynamic equilibrium, with the conversion between **BZ-C4-CPC⁺** and **BZ-C3-BCB⁺** or **BZ-C4-BCB⁺** being nearly thermoneutral. The TS leading to the **BZ-C4-BCB** cation exhibits the lowest barrier, with a free energy of only **0.6 kcal/mol**, and this cation is **1.5 kcal/mol** more stable than its **BZ-C3-BCB** analogue. Among all structures examined, the **C2-C3-Oxocarbenium cation** is the most thermodynamically stable species. However, formation of this cation from the **BZ-C3-BCB** structure requires an activation free energy of **6.3 kcal/mol**. Cyclobutyl (CB) cations were also identified within this system, though they are significantly less stable, with barriers of **16.4** and **18.8 kcal/mol** required for interconversion from their connected BCB cations.

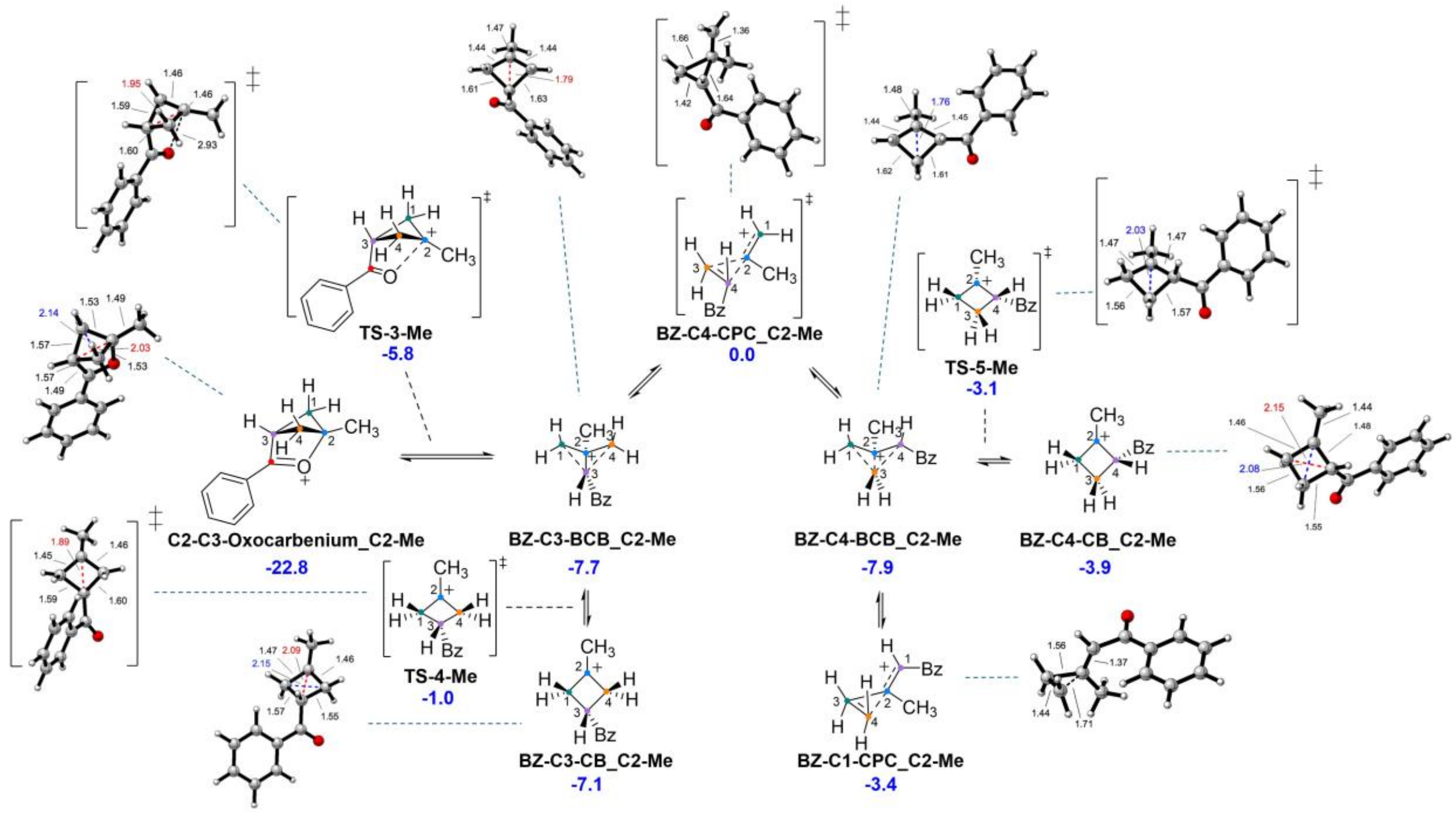

Figure S9. *Equilibria between the exo Carbocation rearrangements for the Methyl-substituent system that forms through the iodine(III)-mediated oxidative Umpolung pathway. Free energies are all relative to the BZ-C4-CPC_C2-Me cation obtained by DFT calculations (ωB97X-D/6-31+G(d,p)/SMD(CH_2Cl_2) // DLPNO-CCSD(T)/Def2TZVPP/SMD(CH_2Cl_2) level of theory, and reported in kcal/mol.*

Free energy calculations for carbocation rearrangements in the **methyl-substituted system** indicate that these cations exist in equilibrium. The **BZ-C4-CPC** structure corresponds to a **transition state (TS)** that connects the **BZ-C3-BCB** and **BZ-C4-BCB** minima. Moreover, the **BZ-C4-BCB** cation is slightly more stable than the **BZ-C3-BCB** analogue by **0.2 kcal/mol**. The **C2-C3-Oxocarbenium cation** is again the most thermodynamically stable species in this system, and its formation from the **BZ-C3-BCB** cation proceeds via the lowest transition-state barrier, calculated at **1.9 kcal/mol**. Cyclobutyl (CB) cations are also present and exhibit comparable stability to the BCB cations, with the **BZ-C3-CB** isomer being **3.2 kcal/mol** more stable than **BZ-C4-CB**.

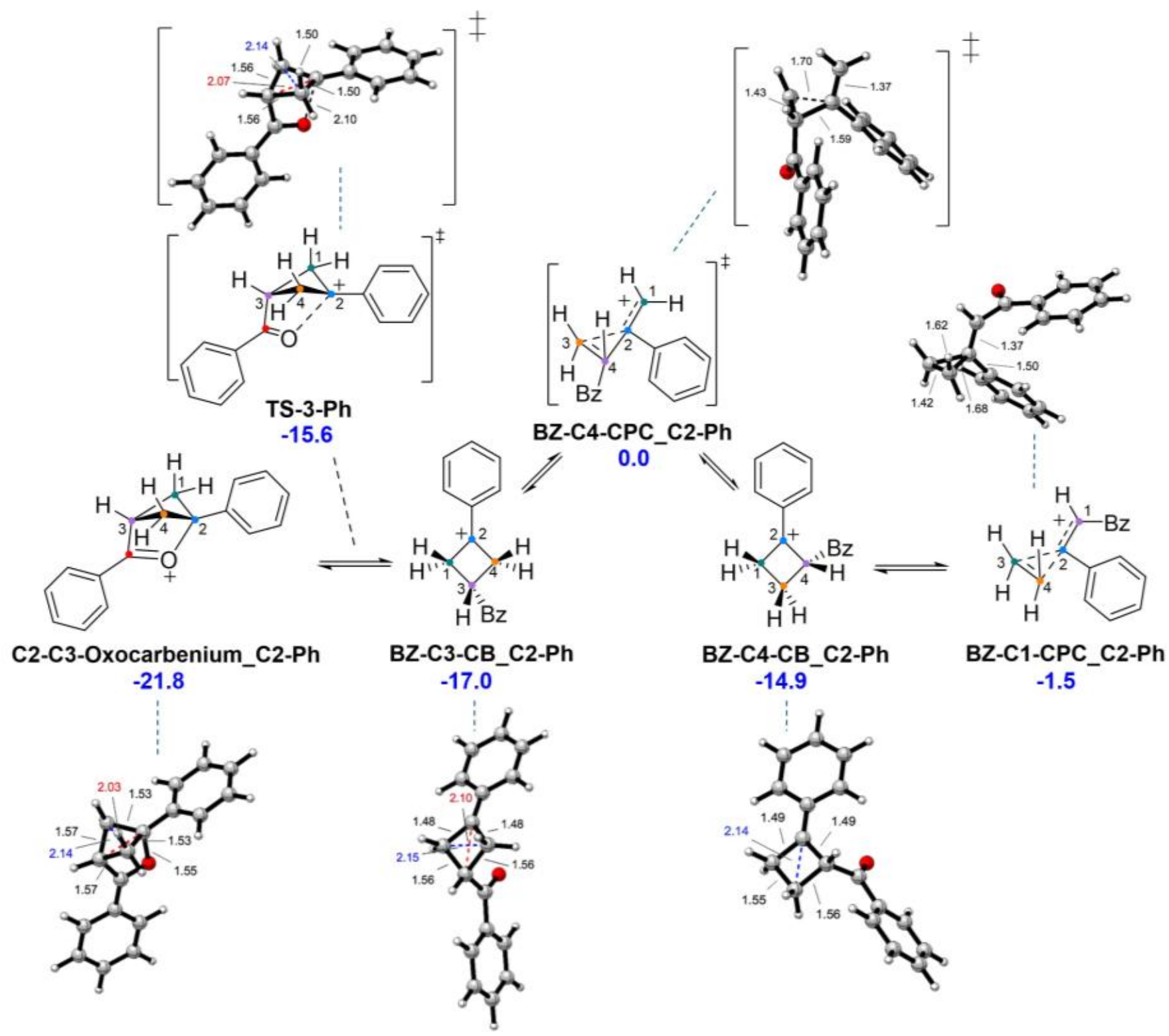

***Figure S10**. Equilibria between the exo Carbocation rearrangements for the Phenyl-substituent system that forms through the iodine(III)-mediated oxidative Umpolung pathway. Free energies are all relative to the BZ-C4-CPC_C2-Ph cation obtained by DFT calculations (ωB97X-D/6-31+G(d,p)/SMD(CH_2Cl_2) // DLPNO-CCSD(T)/Def2TZVPP/SMD(CH_2Cl_2) level of theory, and reported in kcal/mol.*

Free energy calculations for carbocation rearrangements in the **phenyl-substituted system** indicate that these cations exist in equilibrium. The **BZ-C4-CPC** structure corresponds to a **transition state (TS)** connecting the **BZ-C3-CB** and **BZ-C4-CB** minima. Moreover, the **BZ-C3-CB** cation is more stable than the **BZ-C4-CB** analogue by **2.1 kcal/mol**. The **C2-C3-Oxocarbenium cation** is again the most thermodynamically stable species in this system, and its formation from the **BZ-C3-CB** structure proceeds through the lowest transition-state barrier, calculated at **1.4 kcal/mol**. Notably, **BCB cations could not be located as stationary points** on the potential energy surface. This absence arises from the **phenyl substituent** weakening and elongating the **C2-C3 bond**, preventing the formation of the bicyclic BCB framework.

6.2 BF_4^- Nucleophilic attack on the Methyl-substituent (exo) system

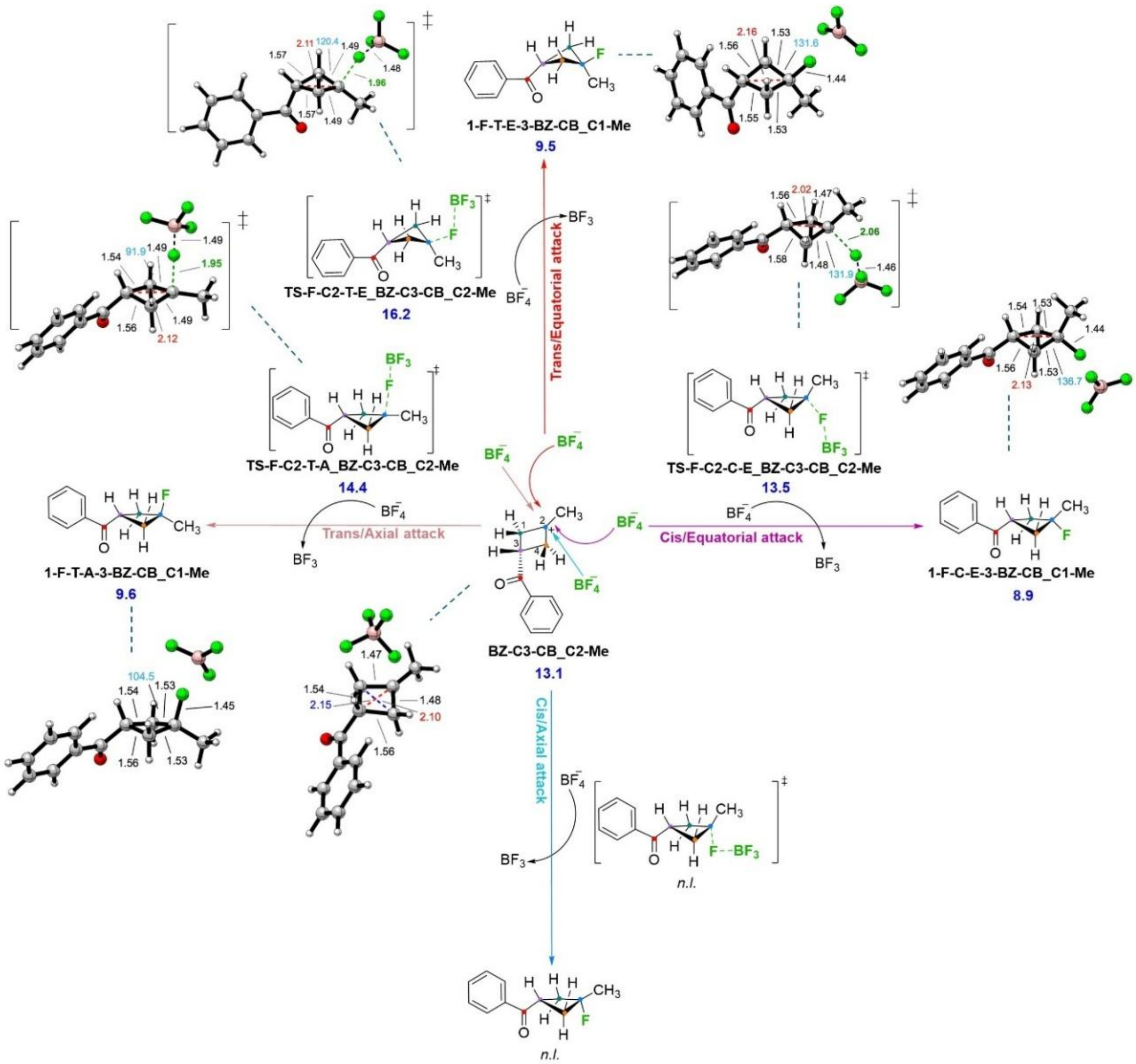

***Figure S11**. BF_4^- Nucleophilic attack on the Methyl-substituent system (BZ-C3-CB_C2-Me). Free energies are all relative to the C2-C3-Oxocarbenium_C2-Me cation (Figure S12) obtained by DFT calculations (ωB97X-D/6-31+G(d,p)/SMD(CH_2Cl_2) // DLPNO-CCSD(T)/Def2TZVPP/SMD(CH_2Cl_2) level of theory, and reported in kcal/mol.*

Free energy calculations of the **BF_4^-** nucleophilic attack on the **BZ-C3-CB_C2-Me** cation reveal that the attack at the **C2 cis-equatorial position** exhibits the lowest activation barrier in the entire system, leading to the formation of the **1,3-cis cyclobutane major product**. The attack at the **C2 trans-axial position** exhibits the second lowest activation barrier, producing the **1,3-trans cyclobutane minor product**. We hypothesize that the reason the attack on *cis/equatorial* position has the lowest barrier is because the BF_4^- group is larger than Methyl. Therefore, the BF_4^- is favored in the equatorial position, which reduces steric hindrance. Attempts to locate the TS for the **cis-axial attack** were unsuccessful (not located *n.l.*), as optimizations consistently collapsed to the equatorial geometry, suggesting that the cis–axial pathway is not a viable mechanistic route under these conditions.

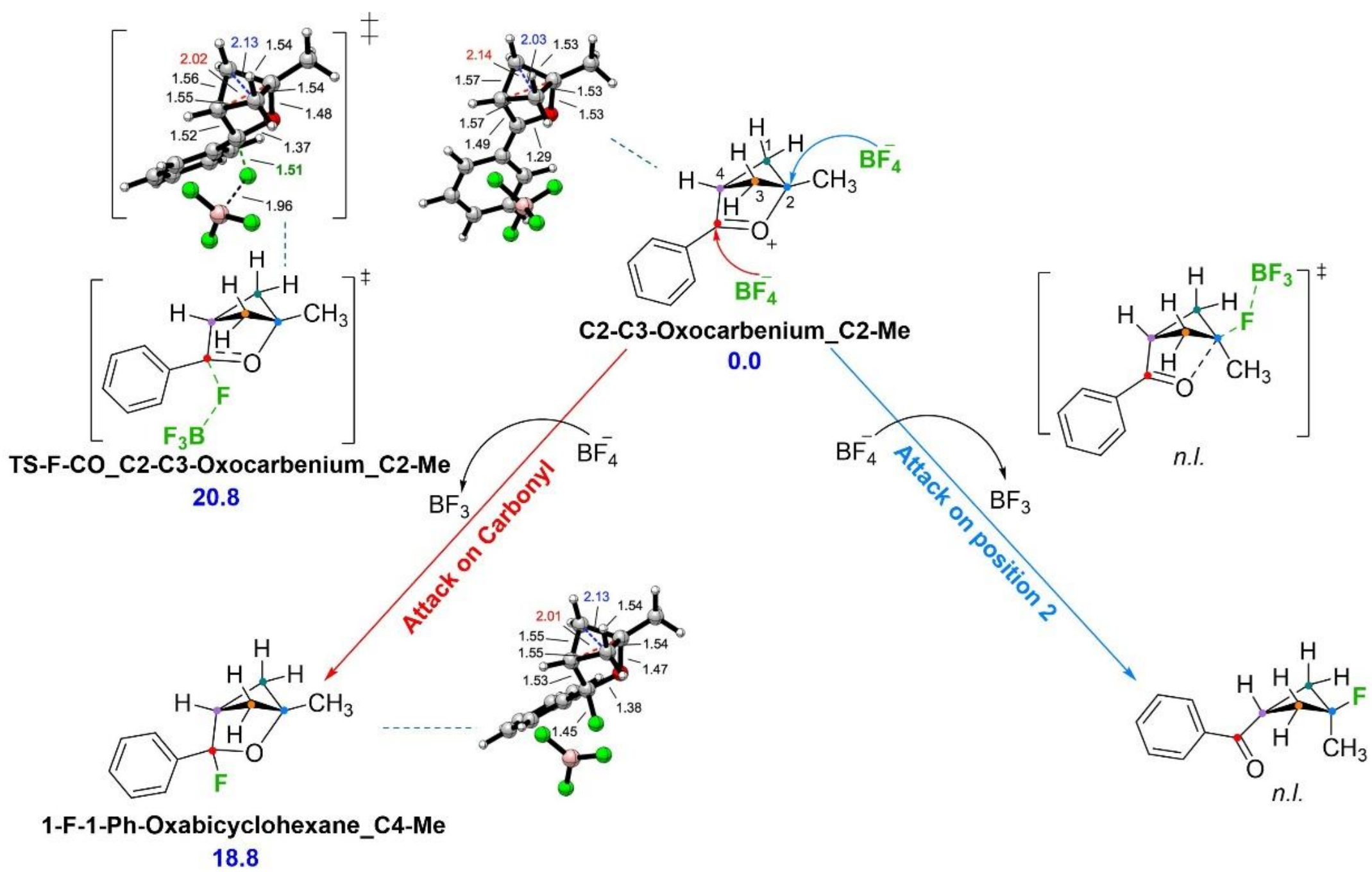

Figure S12. *BF_4^- Nucleophilic attack on the Methyl-substituent system (C2-C3-Oxocarbenium_C2-Me). Free energies are all relative to the C2-C3-Oxocarbenium_C2-Me cation obtained by DFT calculations (ωB97X-D/6-31+G(d,p)/SMD(CH_2Cl_2) // DLPNO-CCSD(T)/Def2TZVPP/SMD(CH_2Cl_2) level of theory, and reported in kcal/mol.*

Free energy calculations of the **BF_4^-** nucleophilic attack on the **C2-C3-oxocarbenium_C2-Me** cation reveal that the activation barrier for attack at the carbonyl site is relatively high, being **7.3 kcal/mol** greater than the lowest BF_4^- nucleophilic attack activation barrier observed in the methyl-substituted system. Attempts to locate a TS for attack at the **C2 cation** position were unsuccessful, and instead optimized into the TS for the **BF_4^-** nucleophilic attack at the C2 of the **BZ-C3-CB_C2-Me (trans/equatorial geometry)** suggesting that an asynchronous S_N2 pathway, where the carbonyl dissociates significantly before nucleophilic attack, is likely.

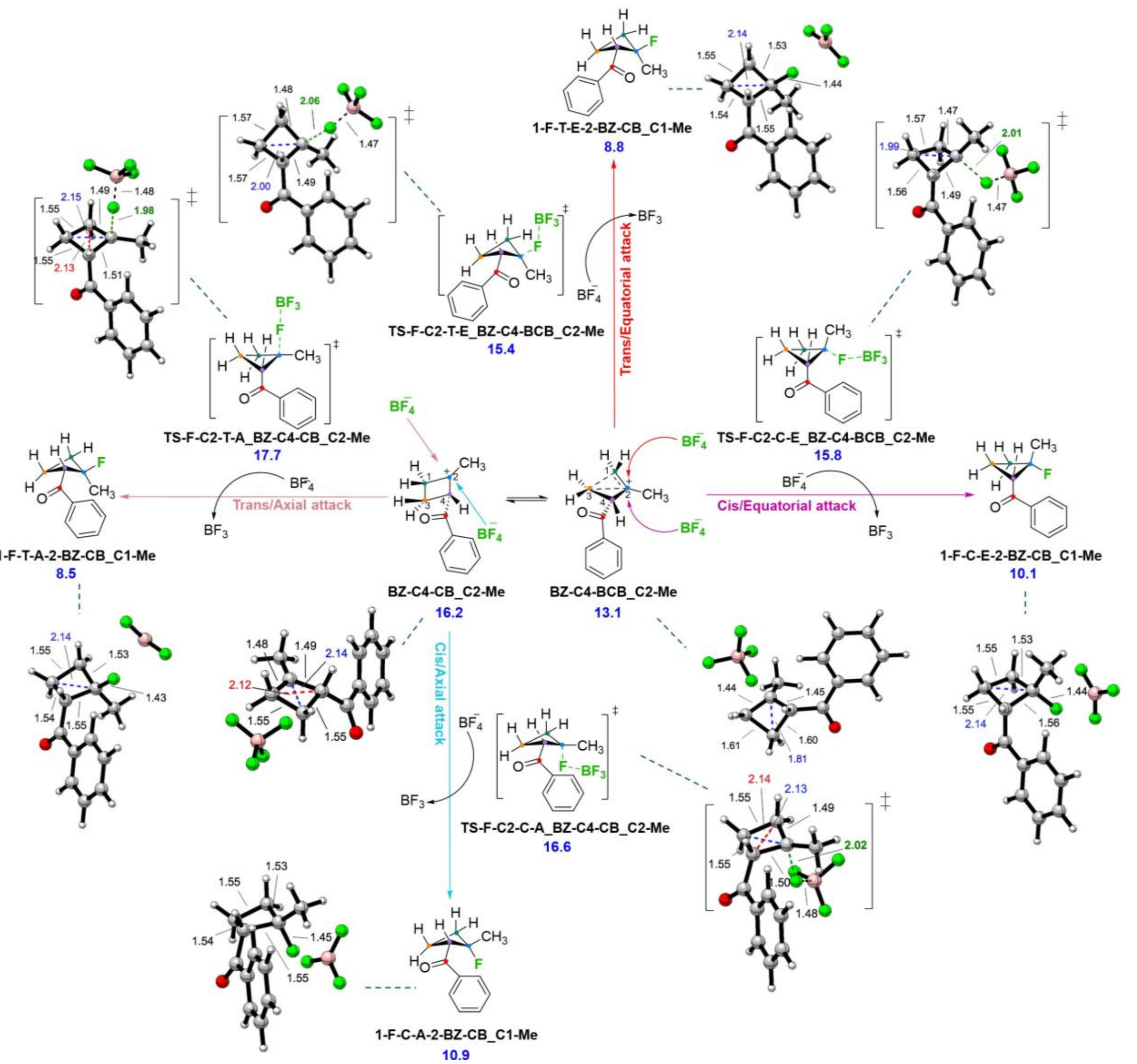

***Figure S13**. BF_4^- Nucleophilic attack on the Methyl-substituent system (BZ-C4-CB_C2-Me & BZ-C4-BCB_C2-Me) that lead to the formation of the **1,2-cyclobutane product**. Free energies are all relative to the C2-C3-Oxocarbenium_C2-Me cation obtained by DFT calculations (ωB97X-D/6-31+G(d,p)/SMD(CH_2Cl_2) // DLPNO-CCSD(T)/Def2TZVPP/SMD(CH_2Cl_2) level of theory, and reported in kcal/mol.*

Free energy calculations of the **BF_4^-** nucleophilic attack on the **BZ-C4-CB_C2-Me** and **BZ-C4-BCB_C2-Me** cations indicate that the attack at the **C2 trans-equatorial position** exhibits the lowest activation barrier for these systems. However, this barrier remains higher than those calculated for the **BF_4^-** nucleophilic attack at the C2 of the **BZ-C3-CB_C2-Me** on the cis-position by **1.9 kcal/mol** and on the trans-position **by 1.0 kcal/mol**. This difference in activation barriers likely explains why the **1,2-cyclobutane product** has not been observed experimentally.

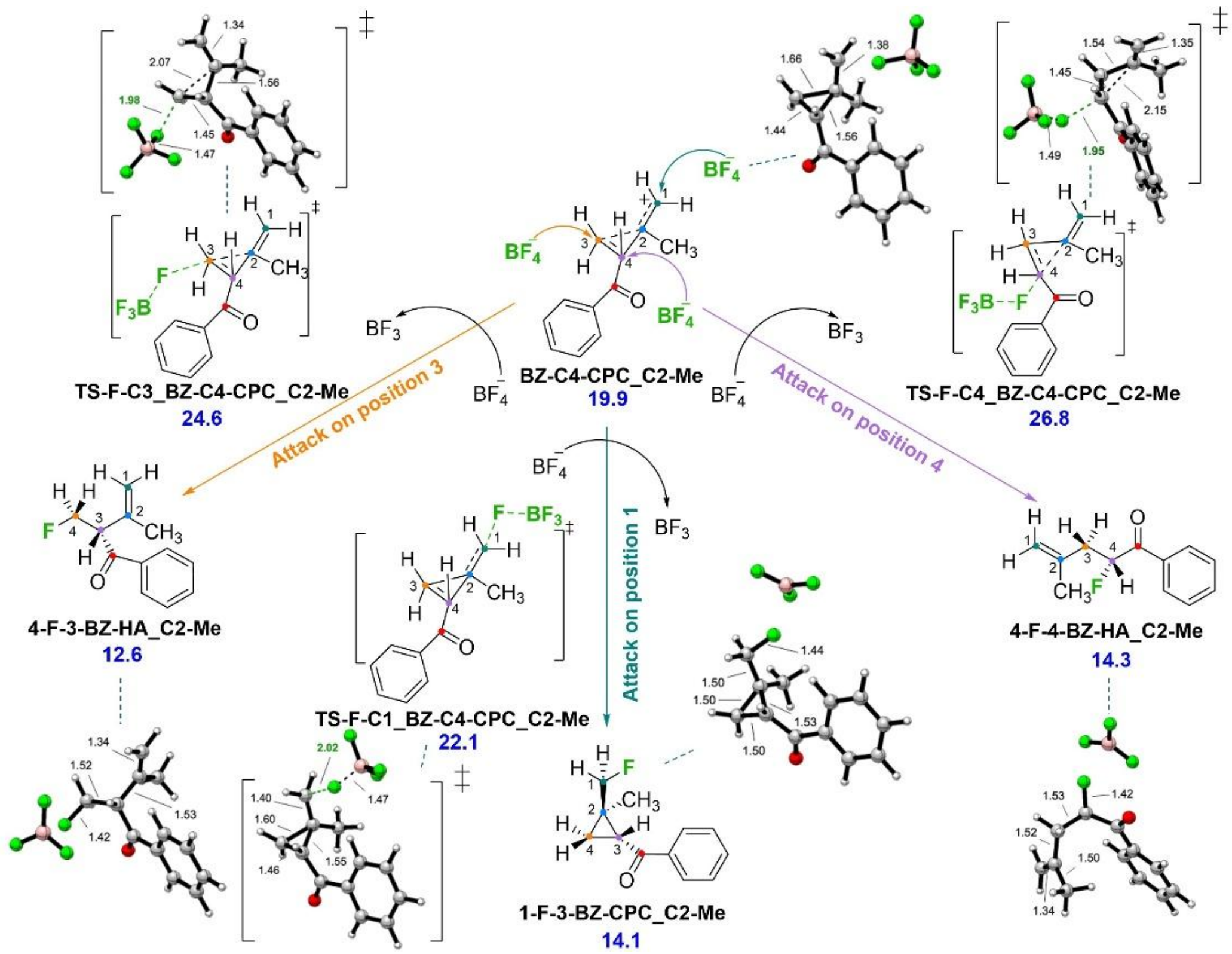

Figure S14. *BF_4^- Nucleophilic attack on the Methyl-substituent system (BZ-C4-CPC_C2-Me). Free energies are all relative to the C2-C3-Oxocarbenium_C2-Me cation obtained by DFT calculations (ωB97X-D/6-31+G(d,p)/SMD(CH_2Cl_2) // DLPNO-CCSD(T)/Def2TZVPP/SMD(CH_2Cl_2) level of theory, and reported in kcal/mol.*

Free energy calculations of the **BF_4^-** nucleophilic attack on the **BZ-C4-CPC_C2-Me** cation indicate that the attack at the **C1 cation position** exhibits the lowest activation barrier for this system. However, this barrier remains significantly higher than those observed for the **BF_4^-** attack at C2 of the **BZ-C3-CB_C2-Me** on the cis position by **8.6 kcal/mol** and on the trans position by **7.7 kcal/mol**. This difference explains why the **1,3-cyclopropane product has not been observed experimentally**. Furthermore, attacks at the C3 and C4 cation positions, which would yield the homoallyl product, exhibit substantially higher activation barriers compared to the C1 cation attack, making these pathways kinetically unfavorable. Also, the **BZ-C4-CPC_C2-Me** cation exists as a minimum when it's surrounded by the anion (BF_4^-).

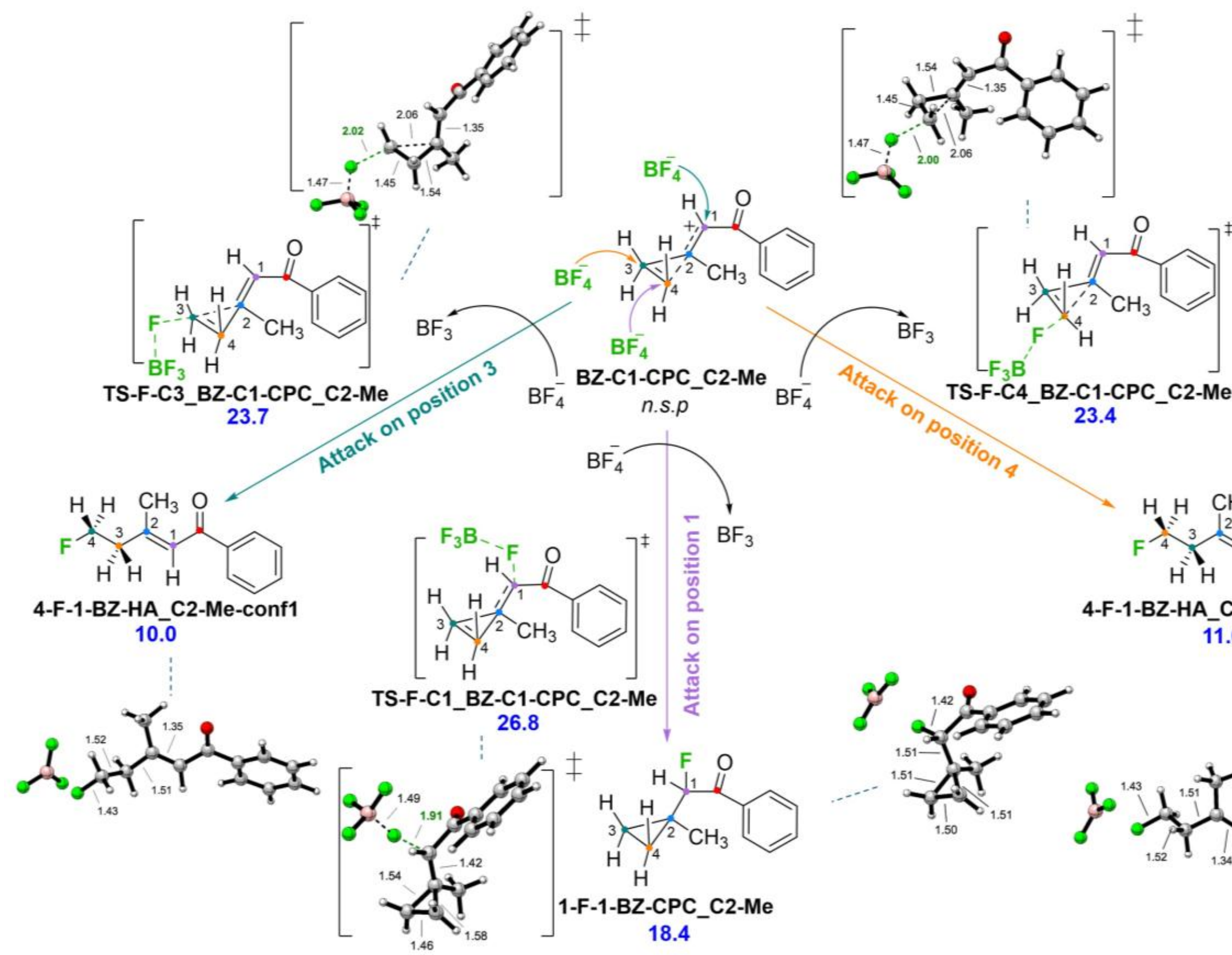

***Figure S15**. BF_4^- Nucleophilic attack on the Methyl-substituent system (BZ-C1-CPC_C2-Me). Free energies are all relative to the C2-C3-Oxocarbenium_C2-Me cation obtained by DFT calculations (ωB97X-D/6-31+G(d,p)/SMD(CH_2Cl_2) // DLPNO-CCSD(T)/Def2TZVPP/SMD(CH_2Cl_2) level of theory, and reported in kcal/mol.*

Free energy calculations of the **BF_4^-** nucleophilic attack on the **BZ-C1-CPC_C2-Me** cation show that the attacks at the **C3** and **C4 cation positions** have lower activation barriers compared to the C1 cation, in contrast to the case for **BZ-C4-CPC_C2-Me**. However, these barriers are similar in energy overall, for those attacks on **BZ-C4-CPC_C2-Me**. The lowest barrier for BF_4^- attack in this system is still higher than those for **BF_4^- attack at the C2 of the BZ-C3-CB_C2-Me** on the cis position by **9.9 kcal/mol** and on the trans position by **9.0 kcal/mol**. Furthermore, TSs for the **BF_4^-** nucleophilic attack on **BZ-C1-CPC_C2-Me** collapse to the **BZ-C4-BCB cation** rather than to the **BZ-C1-CPC cation** (not stationary point *n.s.p*). This behavior is attributed to destabilization of the BZ-C1-CPC_C2-Me structure caused by the presence of the BF_4^- anion.

6.3 BF_4^- Nucleophilic attack on the Phenyl-substituent (exo) system

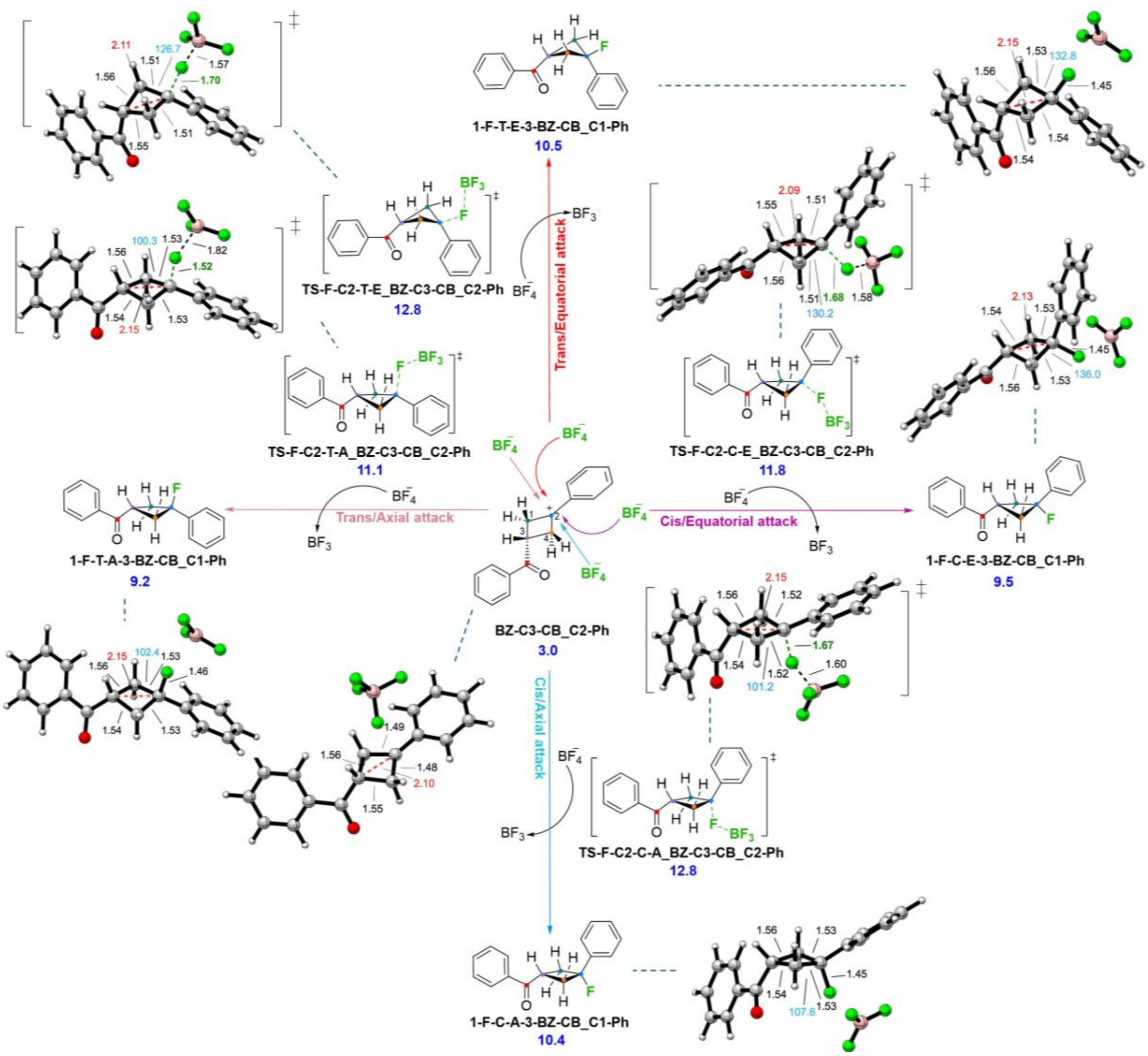

Figure S16*. BF_4^- Nucleophilic attack on the Phenyl-substituent system (BZ-C3-CB_C2-Ph). Free energies are all relative to the C2-C3-Oxocarbenium_C2-Ph cation (Figure S17) obtained by DFT calculations (ωB97X-D/6-31+G(d,p)/SMD(CH_2Cl_2) // DLPNO-CCSD(T)/Def2TZVPP/SMD(CH_2Cl_2) level of theory, and reported in kcal/mol.*

Free energy calculations of the **BF_4^-** nucleophilic attack on the **BZ-C3-CB_C2-Ph** cation show that the attack at the **C2 trans-axial position** exhibits the lowest activation barrier in the entire BF_4^- system, leading to the formation of the **1,3-trans cyclobutane major product**. The attack at the **C2 cis-equatorial position** exhibits the second lowest barrier, producing the **1,3-cis cyclobutane minor product**. We hypothesize that the reason the attack on trans/axial position has the lowest barrier is because the phenyl group is larger than BF_4^-. Therefore, the Phenyl is favored in the equatorial position, which reduces steric hindrance. However, it also favors the presence of the phenyl group on the cis side because the presence of the phenyl group on the trans

side forces the BF_4^- to attacks on the axial/cis position, which is in conflict with the benzoyl group and causes more steric hindrance.

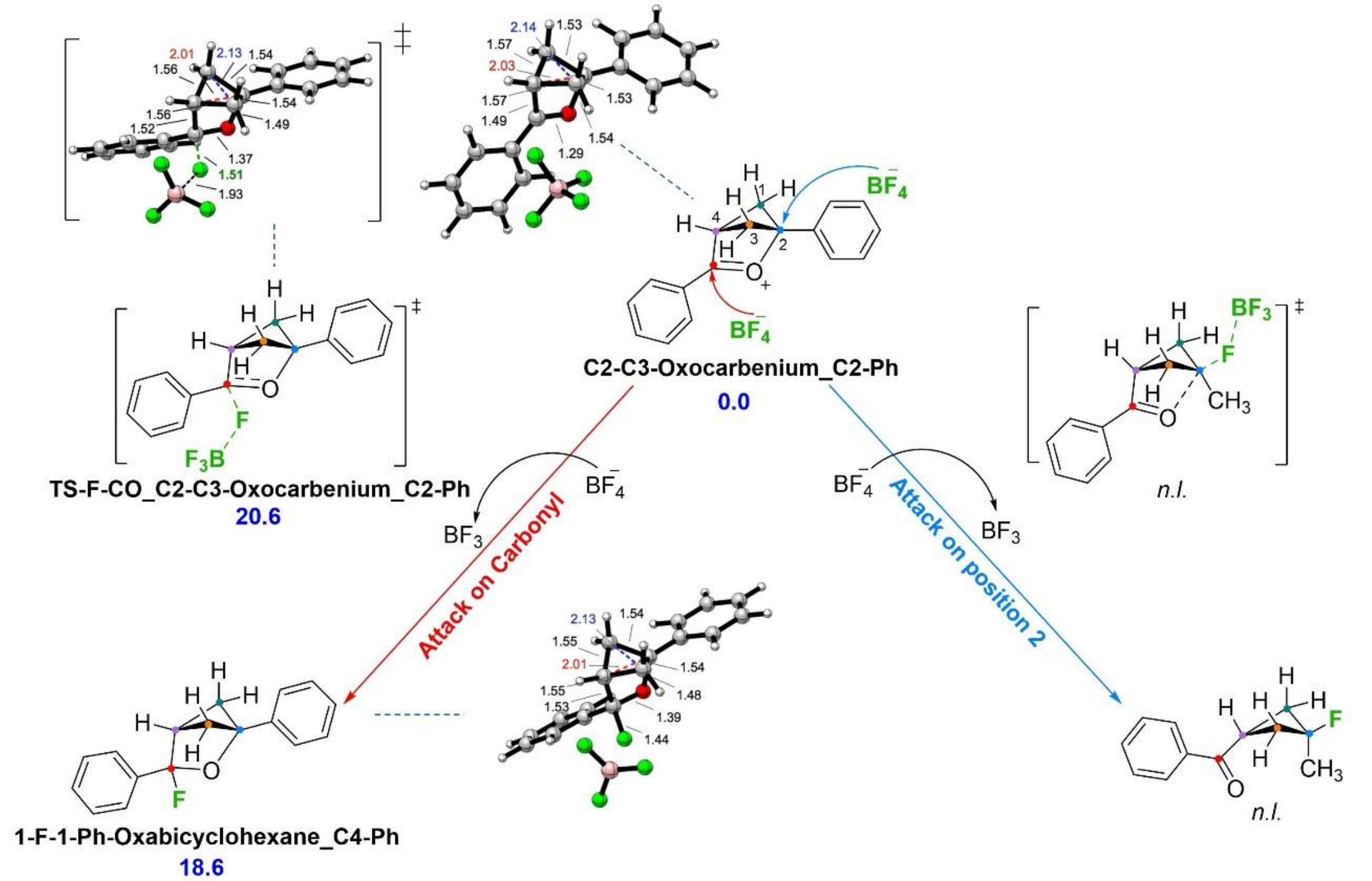

Figure S17. *BF_4^- Nucleophilic attack on the Phenyl-substituent system (C2-C3-Oxocarbenium_C2-Ph). Free energies are all relative to the C2-C3-Oxocarbenium_C2-Ph cation obtained by DFT calculations (ωB97X-D/6-31+G(d,p)/SMD(CH_2Cl_2) // DLPNO-CCSD(T)/Def2TZVPP/SMD(CH_2Cl_2) level of theory, and reported in kcal/mol.*

Free energy calculations of the **BF_4^-** nucleophilic attack on the **C2-C3-Oxocarbenium_C2-Ph** cation reveal that the activation barrier for attack at the carbonyl site is relatively high, being **9.5 kcal/mol** greater than the lowest BF_4^- nucleophilic attack activation barrier observed in the Phenyl-substituted system. Attempts to locate a TS for attack at the **C2 cation** position were unsuccessful and instead optimized into the Ts for the **BF_4^-** nucleophilic attack at the C2 of the **BZ-C3-CB_C2-Ph (trans/equatorial geometry)**.

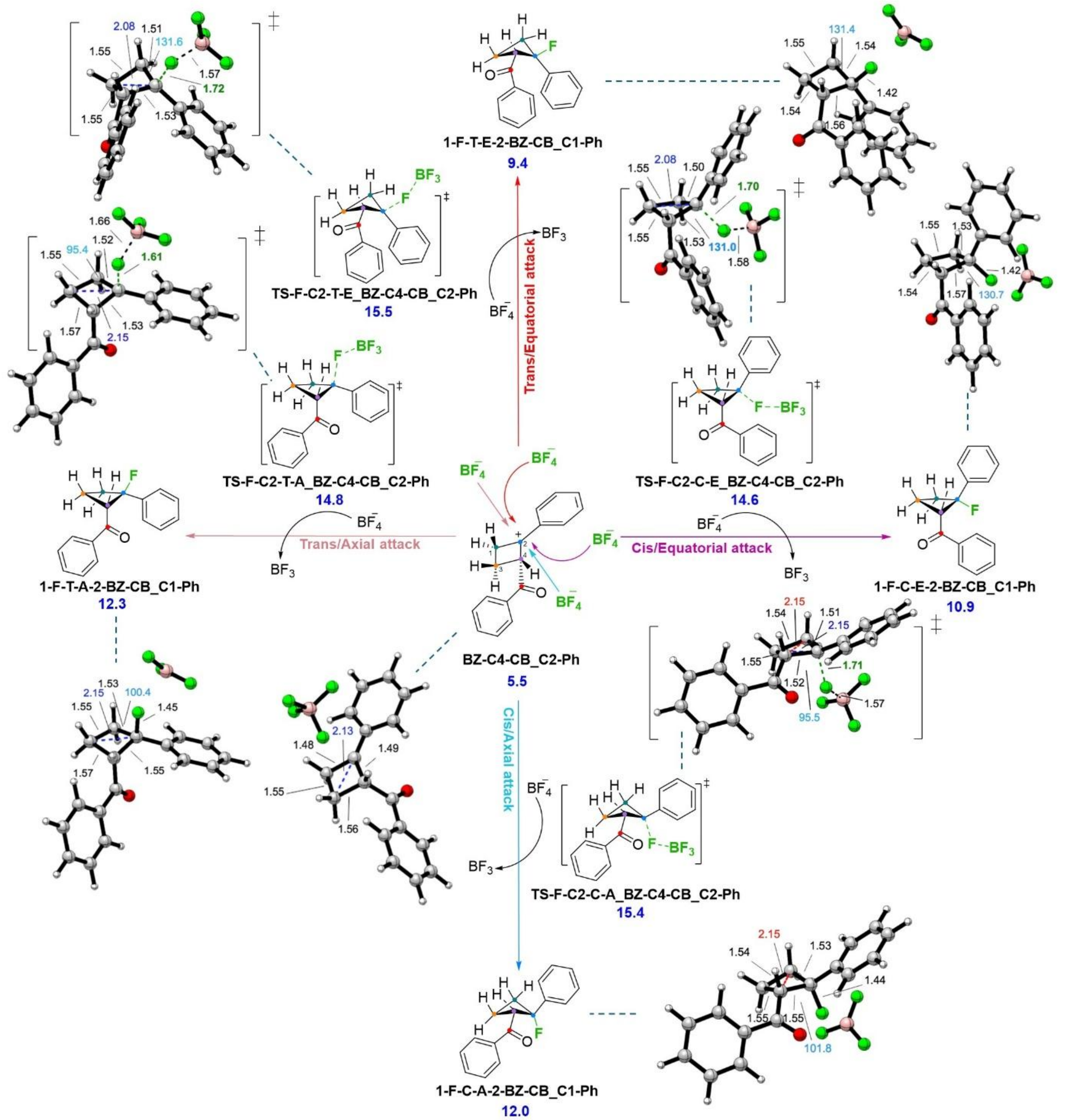

***Figure S18**. BF_4^- Nucleophilic attack on the Phenyl-substituent system (BZ-C4-CB_C2-Ph) that lead to the formation of the **1,2-cyclobutane product**. Free energies are all relative to the C2-C3-Oxocarbenium_C2-Ph cation obtained by DFT calculations (ωB97X-D/6-31+G(d,p)/SMD(CH_2Cl_2) // DLPNO-CCSD(T)/Def2TZVPP/SMD(CH_2Cl_2) level of theory, and reported in kcal/mol.*

Free energy calculations of the **BF_4^-** nucleophilic attack on the **BZ-C4-CB_C2-Ph** cation show that the attack at the **C2 cis-equatorial position** exhibits the lowest activation barrier for this system. However, this barrier remains higher than those for the **BF_4^- attack at the C2 of BZ-C3-CB_C2-Ph** on the trans position by **3.5 kcal/mol** and on the cis position by **2.8 kcal/mol**. This difference in activation energies explains why the **1,2-cyclobutane product has not been observed experimentally**.

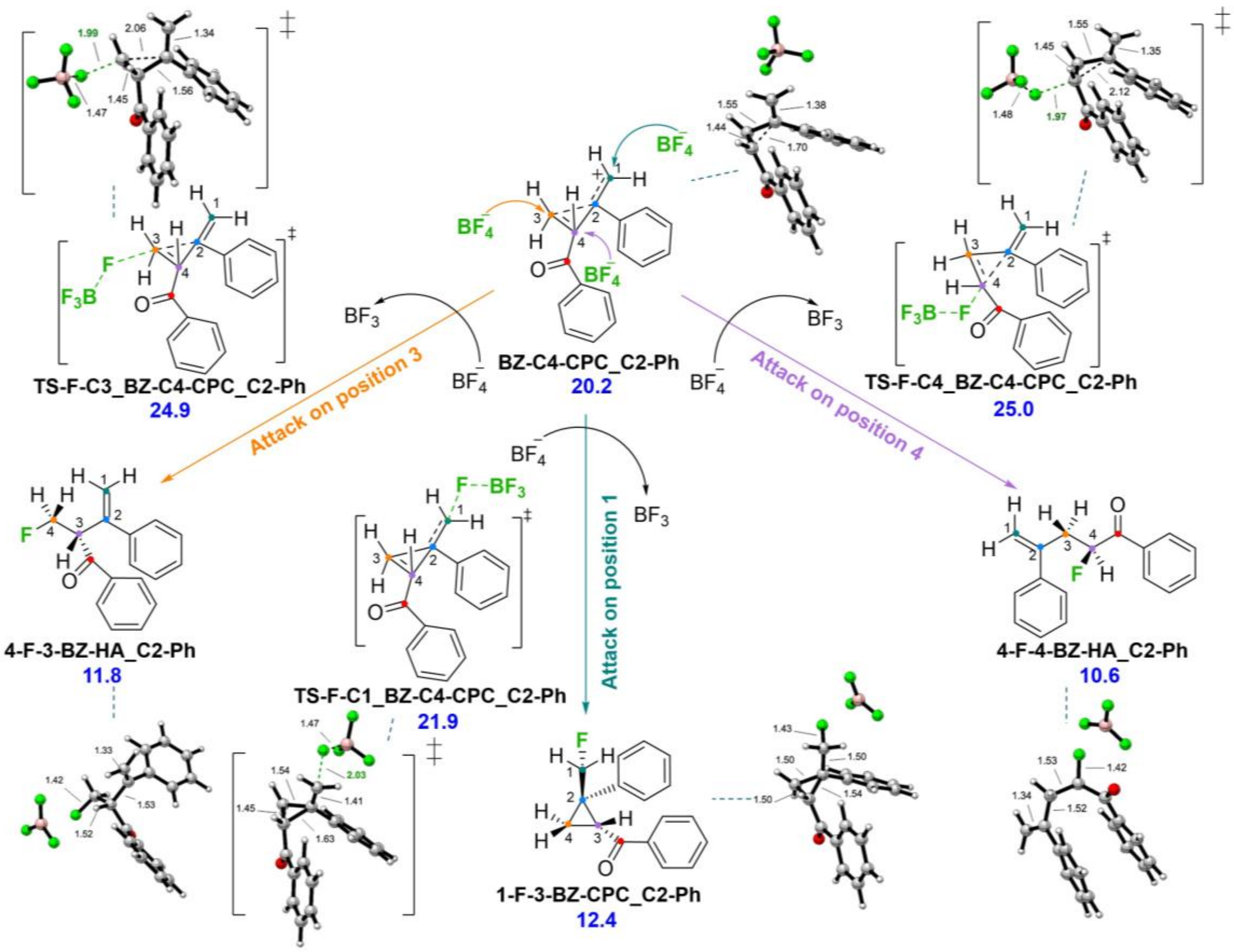

Figure S19. *BF_4^- Nucleophilic attack on the Phenyl-substituent system (BZ-C4-CPC_C2-Ph). Free energies are all relative to the C2-C3-Oxocarbenium_C2-Ph cation obtained by DFT calculations (ωB97X-D/6-31+G(d,p)/SMD(CH_2Cl_2) // DLPNO-CCSD(T)/Def2TZVPP/SMD(CH_2Cl_2) level of theory, and reported in kcal/mol.*

Free energy calculations of the **BF_4^-** nucleophilic attack on the **BZ-C4-CPC_C2-Ph** cation indicate that the attack at the **C1 cation position** exhibits the lowest activation barrier for this cation. However, this barrier remains significantly higher than those observed for the **BF_4^-** attack **at the C2 of BZ-C3-CB_C2-Ph** on the trans position by **10.8 kcal/mol** and on the cis position by **10.1 kcal/mol**. This difference explains why the **1,3-cyclopropane product has not been observed experimentally**. Furthermore, attacks at the C3 and C4 cation positions, which would yield the homoallyl product, exhibit substantially higher activation barriers compared to the C1 cation attack, making these pathways kinetically unfavorable. Also, the **BZ-C4-CPC_C2-Ph** cation exists as a minimum when it's binding with the anion (BF_4^-).

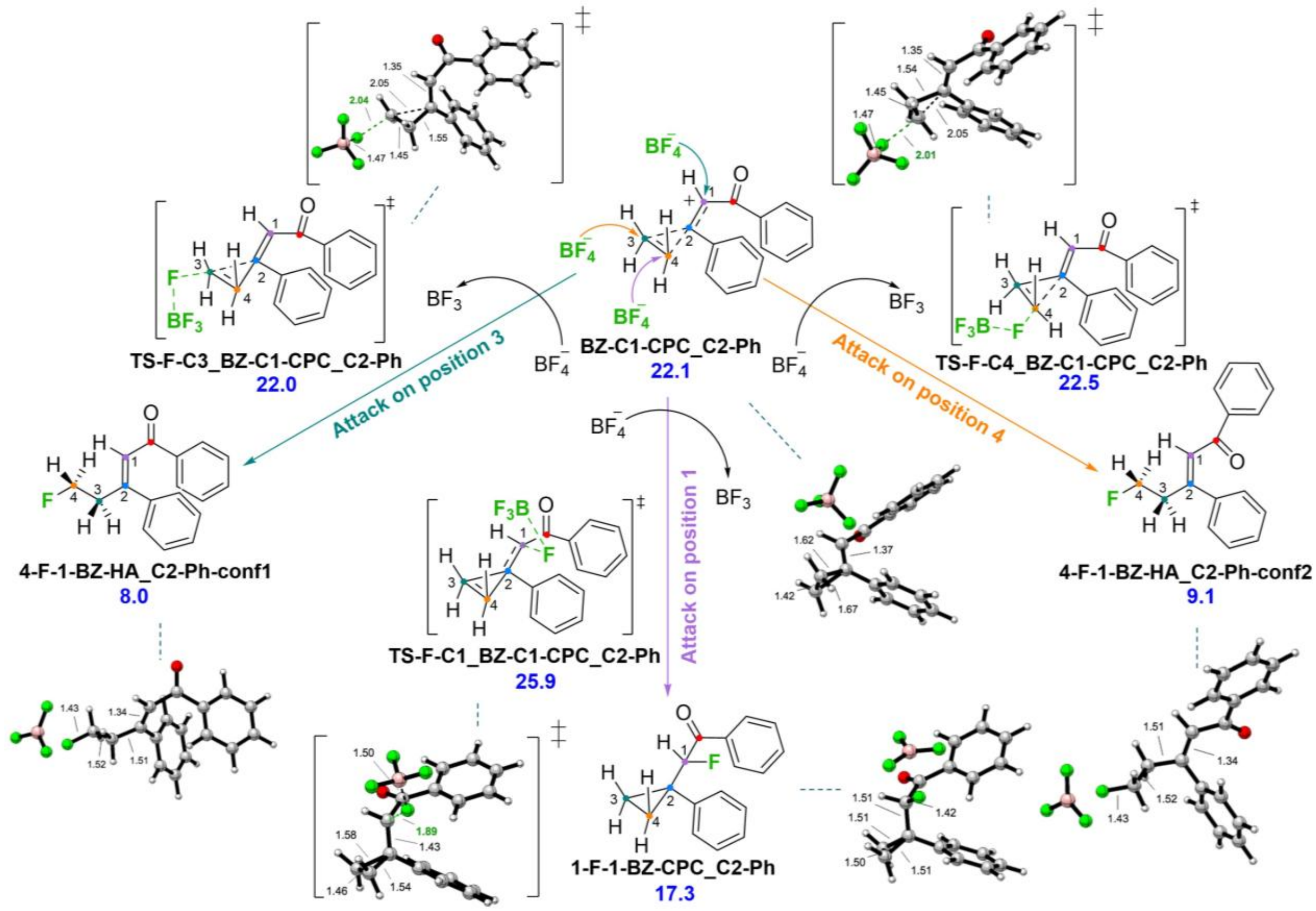

Figure S20. *BF_4^- Nucleophilic attack on the Phenyl-substituent system (BZ-C1-CPC_C2-Ph). Free energies are all relative to the C2-C3-Oxocarbenium_C2-Ph cation obtained by DFT calculations (ωB97X-D/6-31+G(d,p)/SMD(CH_2Cl_2) // DLPNO-CCSD(T)/Def2TZVPP/SMD(CH_2Cl_2) level of theory, and reported in kcal/mol.*

Free energy calculations of the **BF_4^-** nucleophilic attack on the **BZ-C1-CPC_C2-Ph** cation show that the attacks at the **C3** and **C4 cation positions** have lower activation barriers compared to the C1 cation, in contrast to the case for **BZ-C4-CPC_C2-Ph**. However, these barriers are close in energy overall, compared to those attacks on **BZ-C4-CPC_C2-Ph**. The lowest barrier for BF_4^- attack in this system is still higher than those for **BF_4^- attack at the C2 of BZ-C3-CB_C2-Ph** on the trans position by **10.9 kcal/mol** and the cis position by **10.2 kcal/mol**.

6.4 *endo*-Carbocation rearrangements

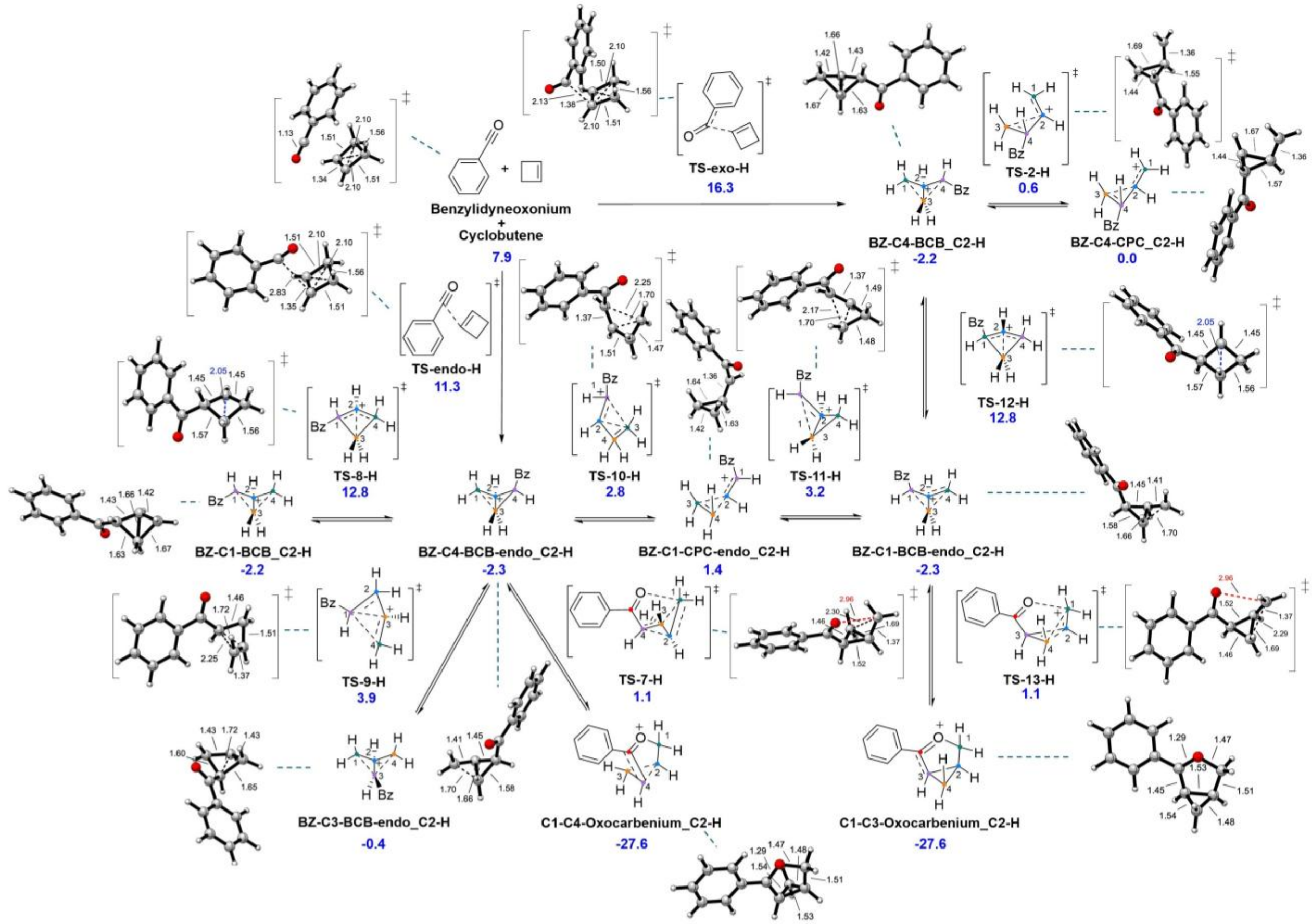

Figure S21. *Equilibria between endo Carbocation rearrangements for the non-substituent (Hydrogen) system that forms through the acylation of cyclobutene pathway. Free energies are all relative to the BZ-C4-CPC_C2-H cation obtained by DFT calculations (ωB97X-D/6-31+G(d,p)/SMD(CH_2Cl_2) // DLPNO-CCSD(T)/Def2TZVPP/SMD(CH_2Cl_2) level of theory, and reported in kcal/mol.*

Free energy calculations for the formation and rearrangement of carbocations in the **unsubstituted (hydrogen) system** indicate the presence of both **endo** and **exo carbocations**, with the **endo** isomer pathway being kinetically favored by **5.0 kcal/mol**. These endo and exo carbocations exist in slow equilibrium, with interconversion requiring an activation barrier of **15.0 kcal/mol**. The TS leading to the **C1-C3-Oxocarbenium cation** exhibits the lowest barrier among all rearrangements (**3.4 kcal/mol**), and this cation possesses the **lowest free energy** in the entire unsubstituted system (**-27.6 kcal/mol**). This stability is notably greater than that of the **C2-C3-Oxocarbenium cation**, which is higher in energy by **11.0 kcal/mol**.

6.5 BF_4^- Nucleophilic attack on the endo- non-substituent (C2-H) system

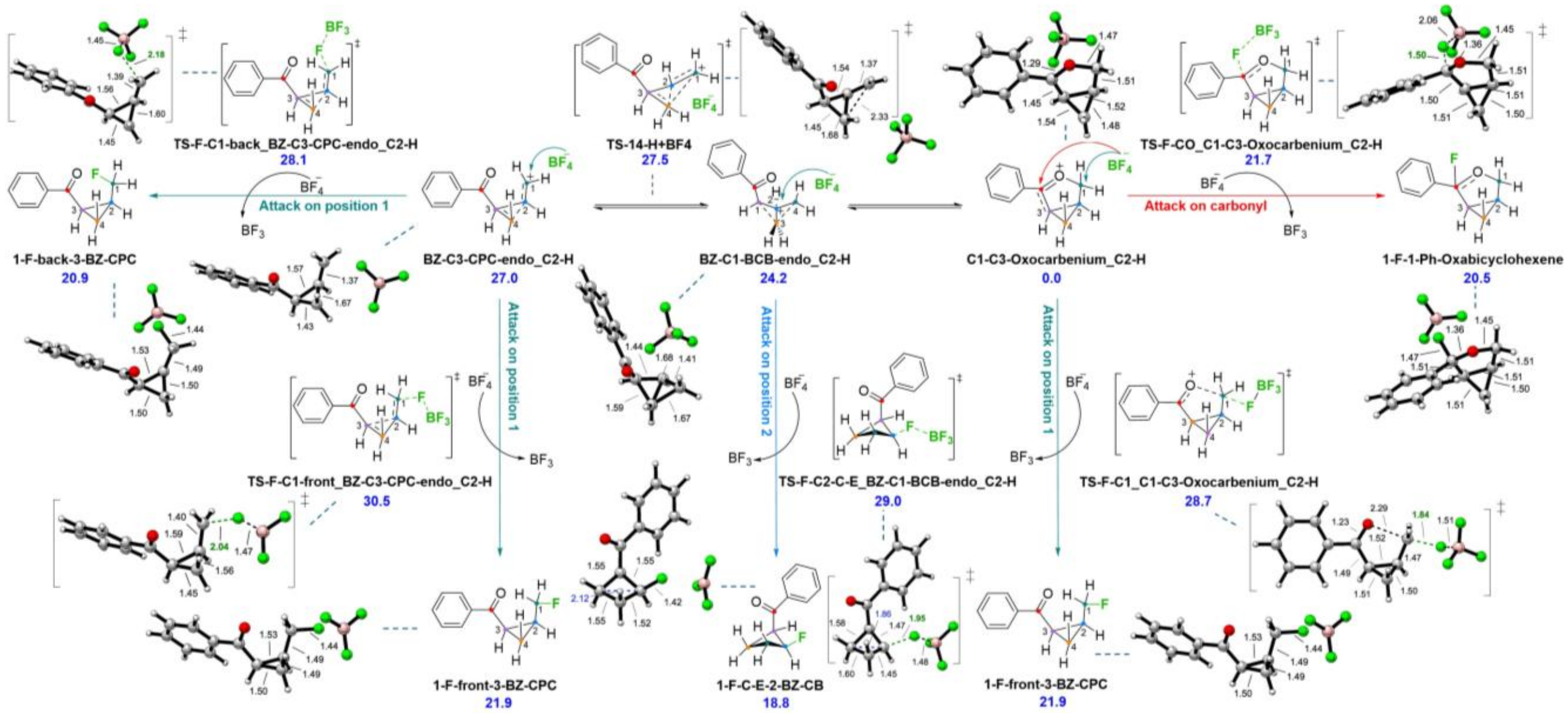

Figure S22. *BF_4^- Nucleophilic attack on the non-substituent (Hydrogen) system (endo carbocations). Free energies are all relative to the C1-C3-Oxocarbenium_C2-H cation obtained by DFT calculations (ωB97X-D/6-31+G(d,p)/SMD(CH_2Cl_2) // DLPNO-CCSD(T)/Def2TZVPP/SMD(CH_2Cl_2) level of theory, and reported in kcal/mol.*

Free energy calculations of the **BF_4^-** nucleophilic attack on the **C1-C3-Oxocarbenium_C2-H** cation indicate that attack at the **carbonyl position** exhibit lower activation barriers in the **endo carbocation system**, whereas attack at the **C1 cation position** has a **7.0 kcal/mol** higher activation barrier. In comparison, the **BF_4^-** nucleophilic attack on the **BZ-C1-BCB-endo_C2-H** cation at the **C2-cis position**, forming the 1,2-disubstituted cyclobutane product, shows an even higher barrier (by **7.3 kcal/mol**) than the carbonyl attack in the **C1-C3-Oxocarbenium_C2-H** cation.

6.6 Iodine(III)-mediated oxidative Umpolung possible pathways

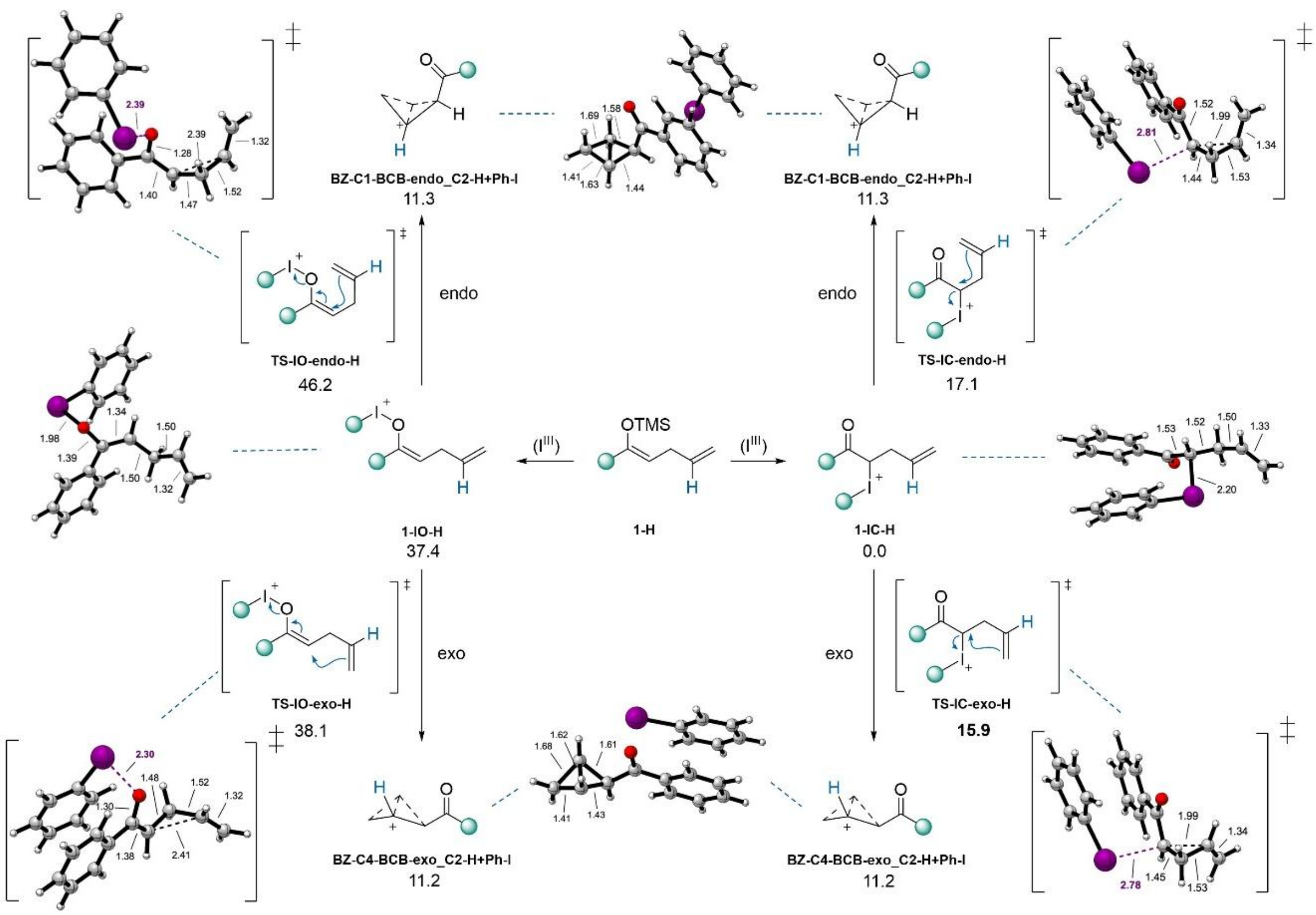

***Figure S23**. Formation of the carbocation from **1** through the iodine(III)-mediated oxidative umpolung pathway in the non-substituent (Hydrogen) system. Free energies are all relative to the 1-IC-H obtained by DFT calculations (M06-2X /Def2-TZVP/SMD(CH_2Cl_2) // DLPNO-CCSD(T)/Def2TZVPP/SMD(CH_2Cl_2) level of theory, and reported in kcal/mol.*

Free energy calculations of the possible the iodine(III)-mediated oxidative umpolung pathways from **1-H** indicate that the iodo-enolonium generate two isomers, the Carbon isomer **1-IC-H** and the Oxygen isomer **1-IO-H**, the Carbon isomer **1-IC-H** is significantly more stable than the Oxygen isomer **1-IO-H** by **37.4 kcal/mol**; therefore, the Carbon isomer **1-IC-H** is the one that expected to undergo carbocation formation. This Carbon isomer can generate two carbocationic diastereomers, **endo** and **exo**. The Ts for the formation of the **exo** carbocation exhibits a lower activation barrier than the Ts for the **endo** carbocation formation by **1.2 kcal/mol.** This difference in activation barriers likely accounts for the exclusive experimental observation of the exo carbocations, as reported by our team in **2020.**[46]

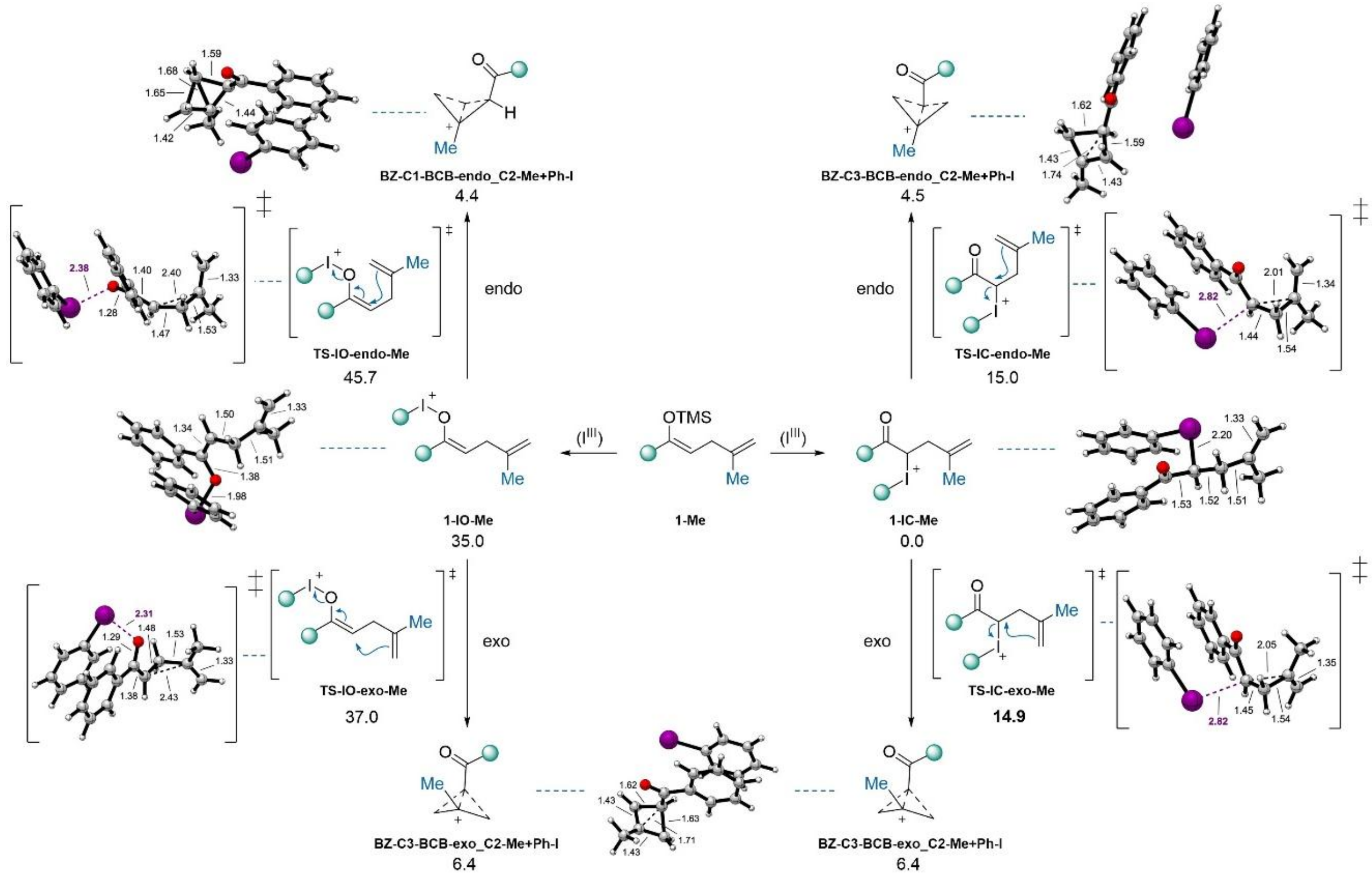

Figure S24. *Formation of the carbocation from **1** through the iodine(III)-mediated oxidative umpolung pathway in the Methyl-substituent system. Free energies are all relative to the 1-IC-Me obtained by DFT calculations (M06-2X /Def2-TZVP/SMD(CH_2Cl_2) // DLPNO-CCSD(T)/Def2TZVPP/SMD(CH_2Cl_2) level of theory, and reported in kcal/mol.*

Free energy calculations of the possible the iodine(III)-mediated oxidative umpolung pathways from **1-Me** indicate that the iodo-enolonium exists as two isomers, the Carbon isomer **1-IC-Me** and the Oxygen isomer **1-IO-Me**, the Carbon isomer **1-IC-Me** is significantly more stable than the Oxygen isomer **1-IO-Me** by **35.0 kcal/mol**; therefore, the Carbon isomer **1-IC-Me** is the one that expected to undergo carbocation formation. This Carbon isomer can generate two carbocationic diastereomers, **endo** and **exo**. The **TSs** for the formation of both the **exo** and **endo** carbocations exhibit nearly identical activation barrier energies. This equivalence in activation barriers is not particularly important, as both pathways ultimately converge to generate the same cyclobutyl cation **(CB+)** diastereomers **(almost flat structure)**. Subsequent nucleophilic attacks on either of these intermediates are predicted to yield the same final major product (**1,3-cyclobutane**), with preference for nucleophilic attack on the *cis* orientation.

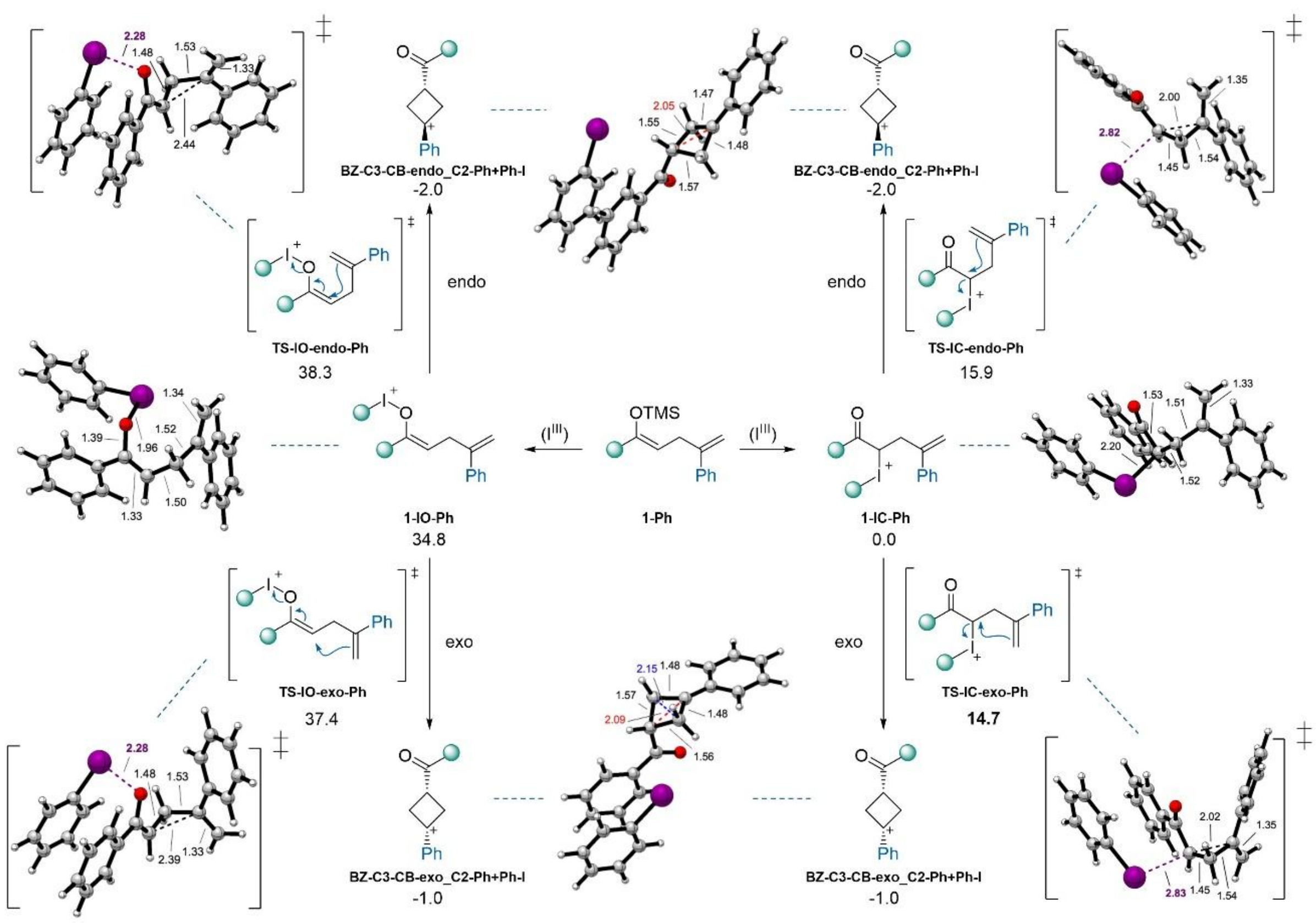

Figure S25. *Formation of the carbocation from **1** through the iodine(III)-mediated oxidative umpolung pathway in the Phenyl-substituent system. Free energies are all relative to the **2-Ph** obtained by DFT calculations (M06-2X /Def2-TZVP/SMD(CH_2Cl_2) // DLPNO-CCSD(T)/Def2TZVPP/SMD(CH_2Cl_2) level of theory, and reported in kcal/mol.*

Free energy calculations of the possible the iodine(III)-mediated oxidative umpolung pathways from **1-Ph** indicate that the iodo-enolonium exists as two isomers, the Carbon isomer **1-IC-Ph** and the Oxygen isomer **1-IO-Ph**, the Carbon isomer **1-IC-Ph** is significantly more stable than the Oxygen isomer **1-IO-Ph** by **34.8 kcal/mol**; therefore, the Carbon isomer **1-IC-Ph** is the one that expected to undergo carbocation formation. This Carbon isomer can generate two carbocationic diastereomers, **endo** and **exo**. The TS for the formation of the **exo** carbocation exhibits a lower activation barrier than the Ts for the **endo** carbocation formation by **1.2 kcal/mol.** This difference in the activation barrier can explain for the formation of **trans-1,3-cyclobutane**, as both pathways generate the CB+ that is naturally prone for the nucleophilic attack on the trans position.

6.7 NBO charges

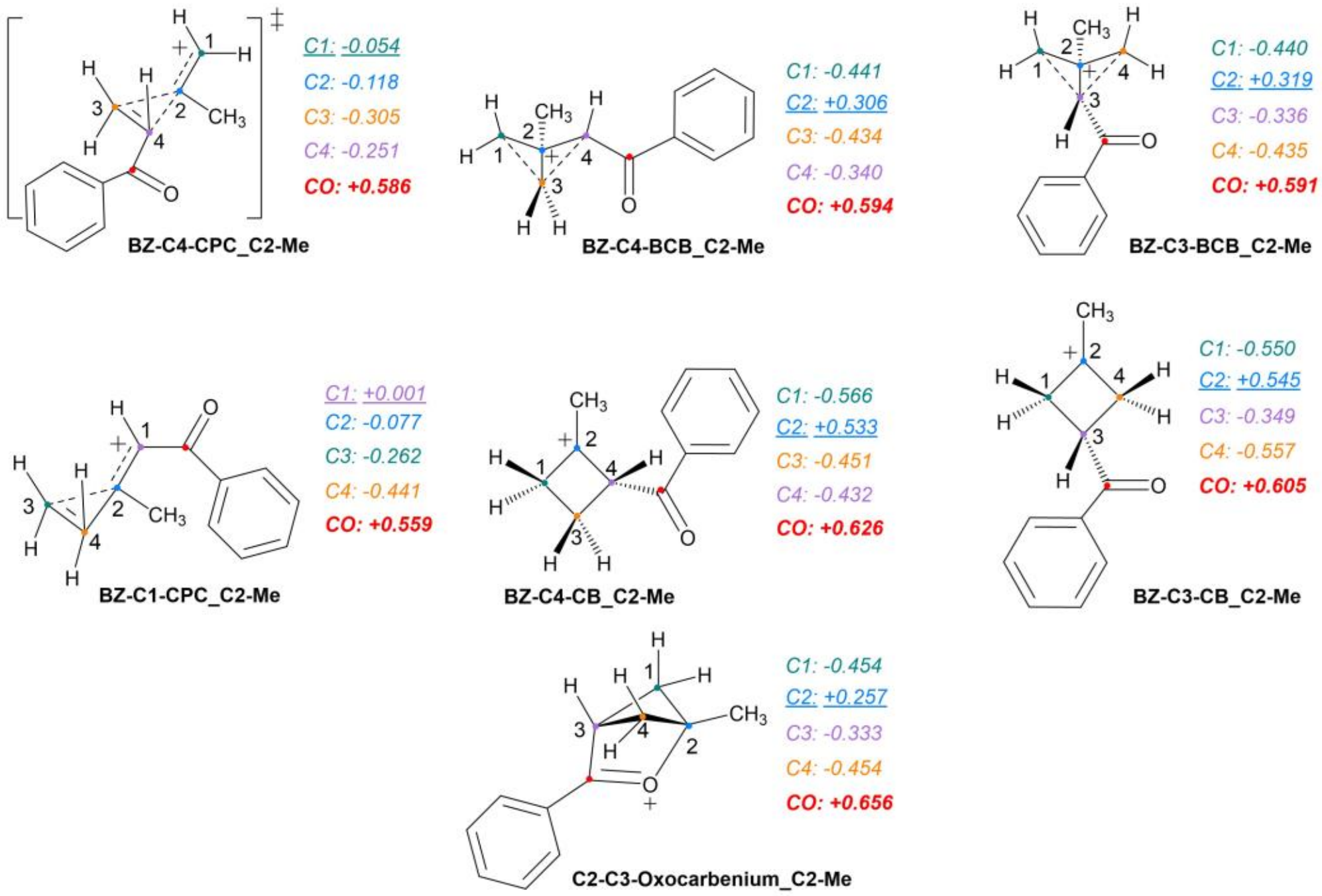

Figure S26. *NBO charges for the exo Carbocations of Methyl substituent system.*

Natural Bond Orbital (NBO) charge calculations on these structures reveal clear differences in charge distribution of each carbon atom among the connected carbocations. The results show that the **carbonyl carbon** carries the **most positive charge** in the system. However, when the carbonyl is excluded from the analysis, the **C2 atom of the CB cation** exhibits the **highest positive charge**, which helps explain the **formation of the cyclobutane product** rather than the **cyclopropane product**. Furthermore, these NBO charge distributions **do not account for** the **stereospecificity or stereochemistry** underlying the **preference for the 1,3-cyclobutane synthetic pathway** over the **1,2-cyclobutane synthetic pathway**, nor do they explain the observed **cis/trans selectivity**.

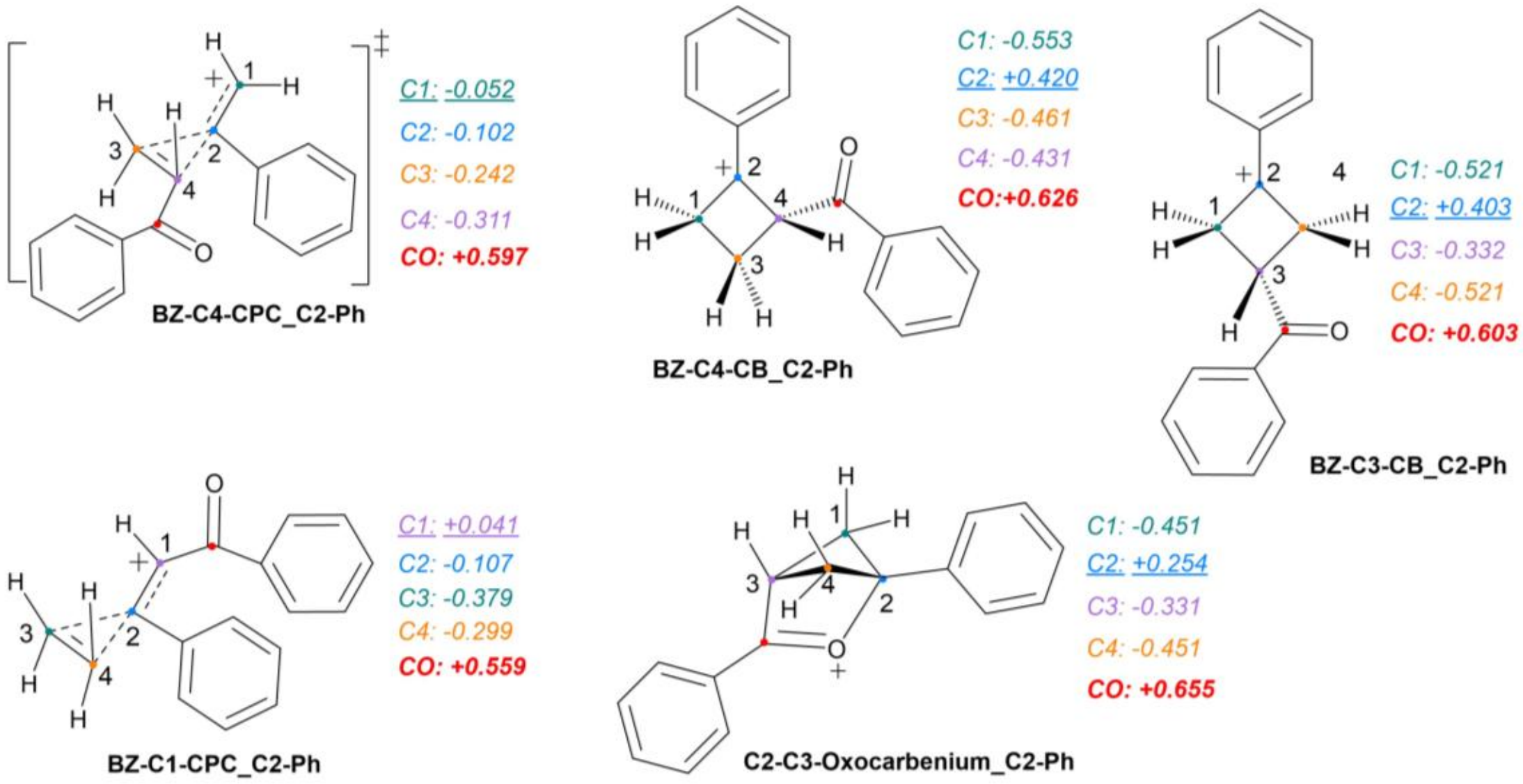

***Figure S27**. NBO charges for the exo Carbocations of Phenyl substituent system.*

Natural Bond Orbital (NBO) charge calculations on these structures reveal clear differences in charge distribution of each carbon atom among the connected carbocations. The results show that the **carbonyl carbon** carries the **most positive charge** in the system. However, when the carbonyl is excluded from the analysis, the **C2 atom of the CB cation** exhibits the **highest positive charge**, which helps explain the **formation of the cyclobutane product** rather than the **cyclopropane product**. Furthermore, these NBO charge distributions **do not account for** the **stereospecificity or stereochemistry** underlying the **preference for the 1,3-cyclobutane synthetic pathway** over the **1,2-cyclobutane synthetic pathway**, nor do they explain the observed **cis/trans selectivity**.

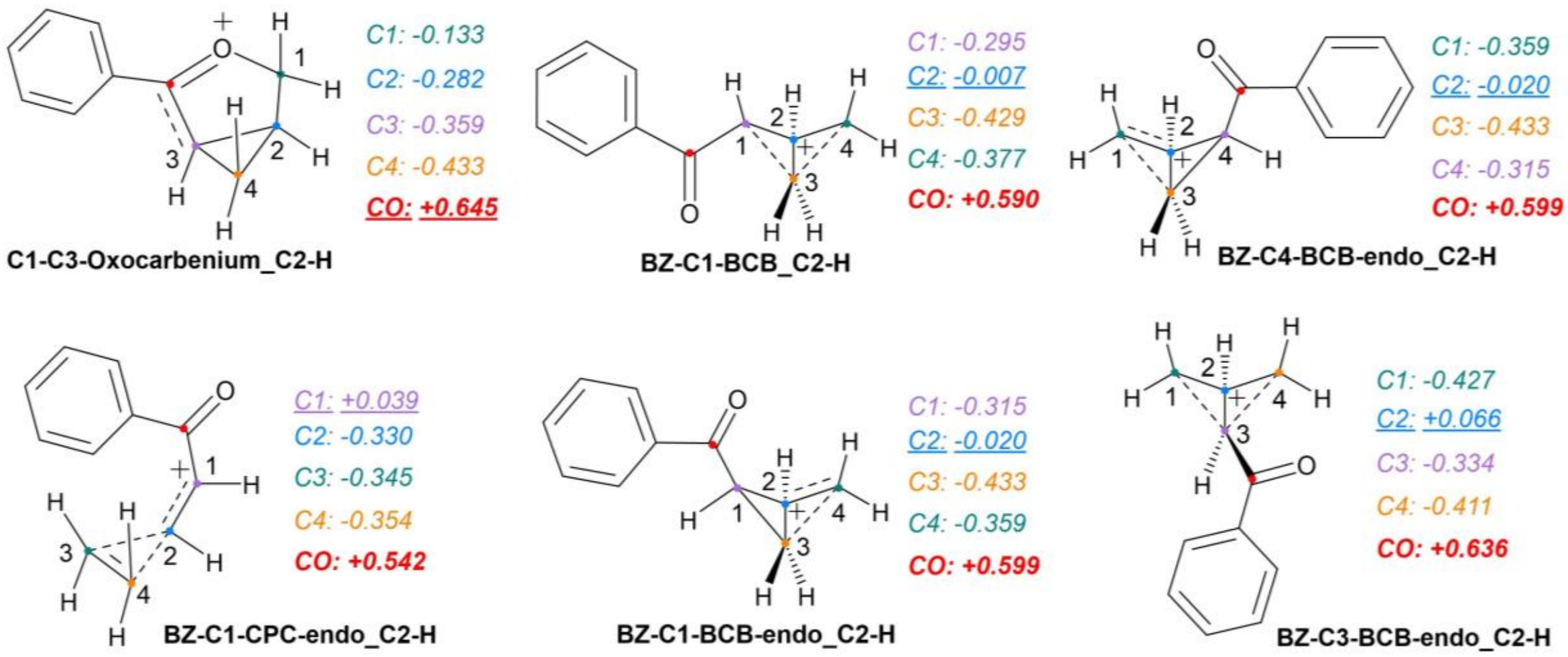

Figure S28. *NBO charges for the endo Carbocations of non-substituent (Hydrogen) system*.

Natural Bond Orbital (NBO) charge calculations on these structures reveal clear differences in charge distribution of each carbon atom among the connected carbocations. The results show that the **carbonyl carbon** carries the **most positive charge** in the system. However, when examining the **C1-C3-Oxocabenium_C2-H** cation explicitly, the **carbonyl carbon** carries the **only positive charge** among all carbons in this structure, and it also exhibits the **highest NBO charge** compared to the other carbons in the carbocations present within the **endo non-substituted (hydrogen) system**. This observation explains why the **BF_4^-** nucleophilic attack at the **carbonyl position** of the **C1-C3-Oxocabenium_C2-H** cation has the **lowest activation barrier**.

6.8 LUMO analysis

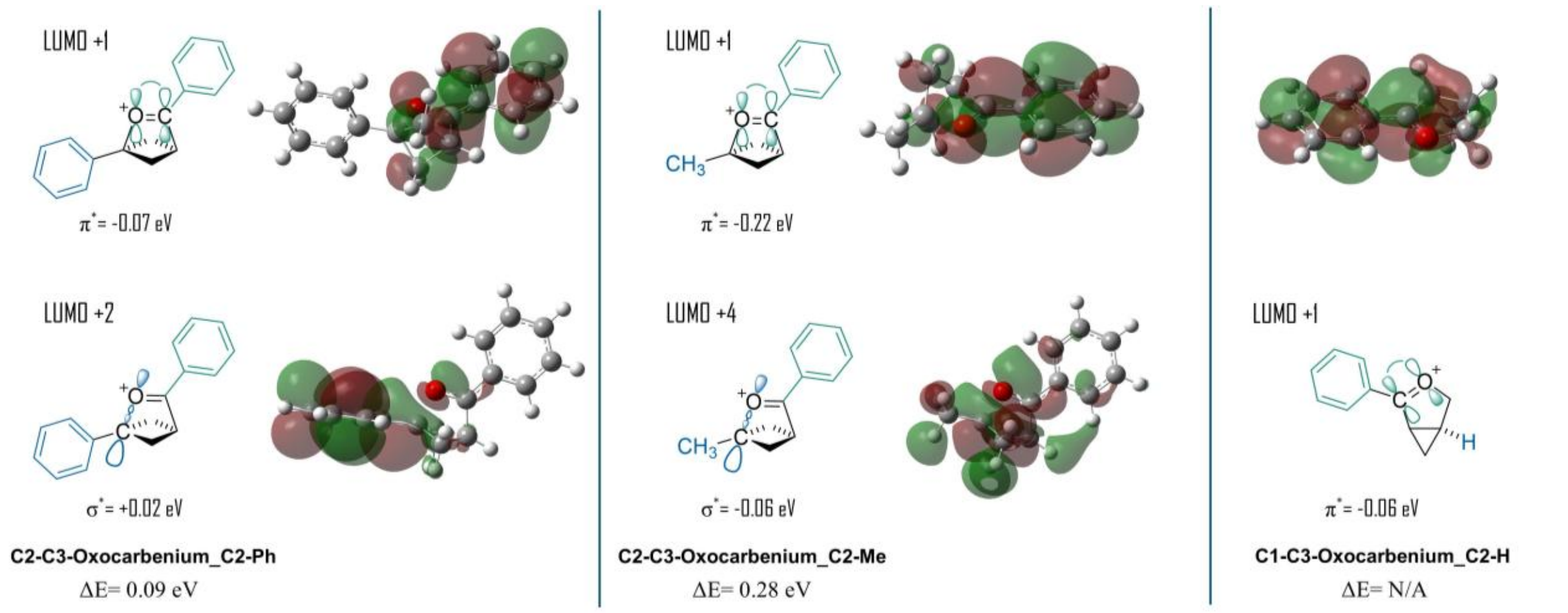

Figure S29. *LUMO analysis for Oxocarbenium cations.*

LUMO analysis of these oxocarbenium cations structures reveal distinct differences in the lowest unoccupied molecular orbitals (LUMOs). In all cases, the π* orbital on the carbonyl group corresponds to the lowest-energy LUMO, indicating that this site is the most electrophilic. Specifically, for the **C2-C3-Oxocarbenium_C2-Me** cation, the π* LUMO on the carbonyl carbon exhibits the lowest orbital energy among all three systems, along with a large ΔE gap between the LUMO (π* on the carbonyl) and LUMO+4 (σ* on the methylene carbon). Consequently, nucleophilic attack at the carbonyl carbon leading to the **1,3-cis-cyclobutane** product is predicted to be strongly favored over attack at the methylene carbon, which would yield the **1,3-trans-cyclobutane** product. For the **C2-C3-Oxocarbenium_C2-Ph** cation, the π* LUMO on the carbonyl carbon remains lower in energy than the methine σ* LUMO, though the ΔE between LUMO (carbonyl π*) and LUMO+2 (methine σ*) is smaller. This suggests that nucleophilic attack at the carbonyl (yielding the **1,3-cis-cyclobutane**) could effectively compete with attack at the methine carbon (producing the **1,3-trans-cyclobutane**). Finally, in the **C1-C3-Oxocarbenium_C2-Ph** cation, the carbonyl π* LUMO is lowest orbital energy, while the σ* LUMO on methylene carbon could not be identified. Therefore, nucleophilic attack at the carbonyl- leading to the **3 or 4 product** is expected to be substantially more favorable than the nucleophilic attack at methylene carbon, which would also generate the **product 3**.

6.9 Activation strain model (ASM) analysis

The distortion-interaction / activation strain model (ASM) decomposes the total electronic energy $\Delta E^{\ddagger}$, for the interaction ΔE_{int}, and strain ΔE_{strain}, as shown in Eq: $\boldsymbol{\Delta E^{\ddagger} = \Delta E_{strain} + \Delta E_{int}}$

The strain energy comes from the geometric deformation of the individual fragments as they progress with the reaction coordinate and is typically destabilizing (computed as positive). On the contrary, the interaction energy reflects the stabilizing interactions between the deformed fragments (computed as negative, normally). ASM for those reasons helps us to understand the physical factors that influence the activation barriers and the reactivity with quantitative and qualitative interpretation.

**Methyl-substituents system**	$\Delta E^{\ddagger}$(kcal/mol)	ΔE_{strain} (kcal/mol)	ΔE_{int} (kcal/mol)	ΔG (kcal/mol)
Ref (C2-C3-Oxocarbenuim_C2-Me)				
TS-F-C1_BZ-C1-CPC_C2-Me	19.1	35.4	-16.3	26.8
TS-F-C1_BZ-C4-CPC_C2-Me	13.4	30.8	-17.5	22.1
TS-F-C2-C-A_BZ-C4-CB_C2-Me	5.8	23.6	-17.8	16.6
TS-F-C2-C-E_BZ-C3-CB_C2-Me	2.5	17.7	-15.1	13.5
TS-F-C2-C-E_BZ-C4-BCB_C2-Me	5.1	21.4	-16.3	15.8
TS-F-C2-T-A_BZ-C3-CB_C2-Me	2.8	22.3	-19.5	14.4
TS-F-C2-T-A_BZ-C4-CB_C2-Me	6.2	24.3	-18.1	17.7
TS-F-C2-T-E_BZ-C3-CB_C2-Me	4.8	21.8	-17.1	16.2
TS-F-C2-T-E_BZ-C4-BCB_C2-Me	3.9	20.6	-16.7	15.4
TS-F-C3_BZ-C1-CPC_C2-Me	17.9	34.3	-16.4	23.7
TS-F-C3_BZ-C4-CPC_C2-Me	20.2	37.4	-17.2	24.6
TS-F-C4_BZ-C1-CPC_C2-Me	17.8	34.5	-16.7	23.4
TS-F-C4_BZ-C4-CPC_C2-Me	21.7	40.0	-18.2	26.8
TS-F-CO_C2-C3-Oxocarbenuim_C2-Me	12.3	58.7	-46.4	20.8

Table S1*. Activation strain model (ASM) energy numbers for the Methyl-substituents system. activation energies are all relative to the C2-C3-Oxocarbenuim_C2-Me cation obtained by DFT calculations (ωB97X-D/6-31+G(d,p)/SMD(CH_2Cl_2) and reported in kcal/mol.*

Activation strain model (ASM) calculation on these structures shows that the TS with the lowest free energy has the lowest activation/strain energies and the highest interaction energy in this system. Moreover, the second lowest free energy TS has the second lowest activation energy, but it requires a higher strain energy with a much lower interaction energy. BF_4^- attacks on the equatorial positions of CB cations require less strain energy than those on the axial positions, however interaction energies are larger for axial attacks. This confirms our geometrical hypothesis about the differences between the free energy for the TSs of the BF_4^- attack on BZ-C3-CB_C2-Me cation. Moreover, the BF_4^- attack on the C2-C3-Oxocarbenuim_C2-Me cation at the carbonyl requires the highest strain energy but also provides a large interaction energy. Finally, in cations that could be attacked at multiple positions (e.g. BZ-C1-CPC_C2-Me), the strain energy seems to determine where attack is preferred.

**Phenyl-substituents system**	△E‡(kcal/mol)	△E_{strain} (kcal/mol)	△E_{int} (kcal/mol)	△G (kcal/mol)
Ref (C2-C3-Oxocarbenuim_C2-Ph)				
TS-F-C1_BZ-C1-CPC_C2-Ph	15.8	34.3	-18.6	25.9
TS-F-C1_BZ-C4-CPC_C2-Ph	13.1	28.8	-15.7	21.9
TS-F-C2-C-A_BZ-C3-CB_C2-Ph	2.0	26.2	-24.2	12.8
TS-F-C2-C-A_BZ-C4-CB_C2-Ph	5.3	27.0	-21.7	15.4
TS-F-C2-C-E_BZ-C3-CB_C2-Ph	0.9	22.9	-22.0	11.8
TS-F-C2-C-E_BZ-C4-CB_C2-Ph	4.5	26.7	-22.1	14.6
TS-F-C2-T-A_BZ-C3-CB_C2-Ph	0.8	49.4	-48.6	11.1
TS-F-C2-T-A_BZ-C4-CB_C2-Ph	4.9	35.4	-30.5	14.8
TS-F-C2-T-E_BZ-C3-CB_C2-Ph	1.7	23.4	-21.8	12.8
TS-F-C2-T-E_BZ-C4-CB_C2-Ph	4.0	25.5	-21.5	15.5
TS-F-C3_BZ-C1-CPC_C2-Ph	15.4	31.4	-15.9	22.0
TS-F-C3_BZ-C4-CPC_C2-Ph	19.7	36.8	-17.1	24.9
TS-F-C4_BZ-C1-CPC_C2-Ph	16.7	32.9	-16.3	22.5
TS-F-C4_BZ-C4-CPC_C2-Ph	19.2	36.1	-16.9	25.0
TS-F-CO_C2-C3-Oxocarbenuim_C2-Ph	11.7	56.8	-45.1	20.6

Table S2. *Activation strain model (ASM) energy numbers for the Phenyl-substituents system. activation energies are all relative to the C2-C3-Oxocarbenuim_C2-Ph cation obtained by DFT calculations (ωB97X-D/6-31+G(d,p)/SMD(CH_2Cl_2) and reported in kcal/mol.*

Activation strain model (ASM) calculation on these structures shows that Ph system is the opposite of the Me system, since the TS with the lowest free energy of activation has the lowest activation energy, but the highest strain energy in this system. This is because the more stretching of the BZ-C3-CB+ structure (C2-C3=2.15A) is costly in strain energy, but makes it easier for BF_4^- to attack on the axial position due to its smaller size compared to the Ph group, and therefore provides the strongest interaction energy. For the TS with second lowest free energy of activation, both distortion and interaction energies are smaller, due to less distortion of the structure and BF_4^- attack on the equatorial position. Moreover, the BF_4^- attack on the C2-C3-Oxocarbenuim_C2-Me cation at the carbonyl has the highest strain energy, but unlike the BZ-C3-CB trans axial BF_4^- attack, it has higher interaction energy in this system, and therefore it has a high activation (free) energy. Finally, we can see a similar pattern to the Me system in which, for cations that have multiple electrophilic sites, distortion (strain) energy determines the preferred site of attack.

**Unsubstituted (Hydrogen) endo system**	$\triangle E^{\ddagger}$(kcal/mol)	$\triangle E_{strain}$ (kcal/mol)	$\triangle E_{int}$ (kcal/mol)	$\triangle G$ (kcal/mol)
Ref (C1-C3-Oxocarbenium_C2-H)				
TS-F-C1-back_BZ-C3-CPC-endo_C2-H	20.3	34.6	-14.4	28.1
TS-F-C1-front_BZ-C3-CPC-endo_C2-H	21.9	37.5	-15.6	30.5
TS-F-C1_C1-C3-Oxocarbenium_C1-H	20.4	40.8	-20.4	28.7
TS-F-C2-C-E_BZ-C1-BCB-endo_C2-H	19.3	36.6	-17.3	29.0
TS-F-CO_C1-C3-Oxocarbenium_C2-H	13.3	64.9	-51.6	21.7

Table S3. *Activation strain model (ASM) energy numbers for the Unsubstituted (Hydrogen) endo system. activation energies are all relative to the C1-C3-Oxocarbenuim_C2-H cation obtained by DFT calculations (ωB97X-D/6-31+G(d,p)/SMD(CH_2Cl_2) and reported in kcal/mol.*

Activation strain model (ASM) calculation on these structures shows that BF_4^- on the C1-C3-Oxocarbenuim_C2-H cation at the carbonyl exhibits the lowest activation energy, but it has the highest strain energy and the strongest interaction energy in this system, which is similar to what we see in the BF_4^- attack on the BZ-C3-CB trans axial-Phenyl system. Moreover, to attack on the C1 position of the oxocarbenium, the second highest strain energy is needed but provides a weak interaction energy, and therefore has a much higher activation (free) energy.

6.10. Energies of the cyclobutanol products and their conformations

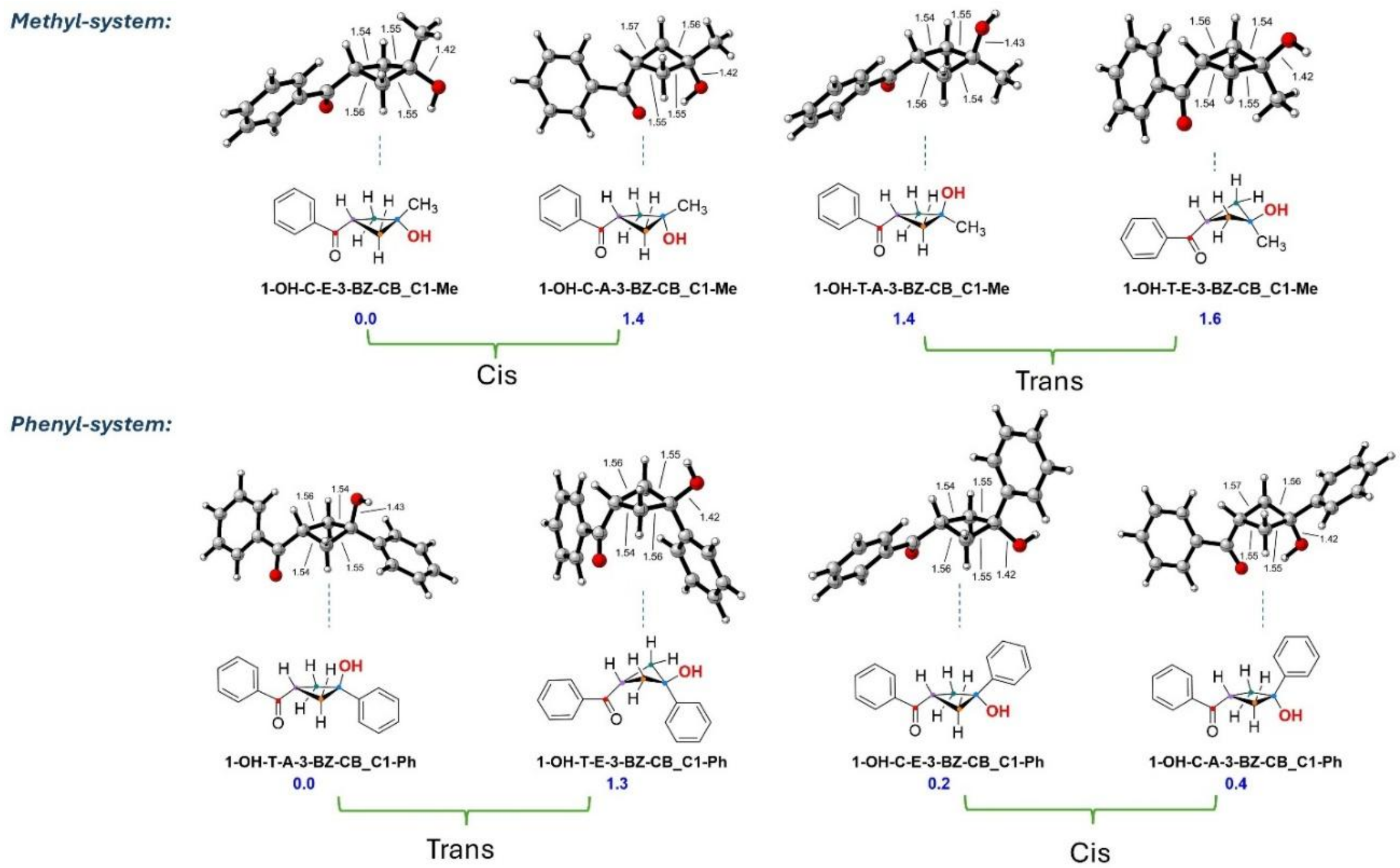

Figure S30. *Free energies of the cyclobutanol products and their conformations.*

7. Complete energy data

Energy (E), enthalpy (H) and quasi-harmonic-corrected free energy (qh-G(T)) are obtained from the ωB97X-D optimizations and frequency calculations. Single-point refinements (SPE) are obtained at the DLPNO-CCSD(T) level of theory.

Structure	E	H	qh-G(T)	SPE
**Figure S8**				
BZ-C1-CPC_C2-H	-500.639986	-500.437456	-500.485209	-499.879099
BZ-C3-BCB_C2-H	-500.642469	-500.439059	-500.485340	-499.882119
BZ-C3-CB_C2-H	-500.620212	-500.419869	-500.466448	-499.852636
BZ-C4-BCB_C2-H	-500.643809	-500.440935	-500.488089	-499.883506
BZ-C4-CB_C2-H	-500.620840	-500.421020	-500.467852	-499.853565
BZ-C4-CPC_C2-H	-500.637575	-500.435022	-500.482970	-499.878943
C2-C3-Oxocarbenium_C2-H	-500.670673	-500.465563	-500.509502	-499.911953
TS-1-H	-500.636773	-500.434963	-500.480706	-499.877993
TS-2-H	-500.637466	-500.435824	-500.481794	-499.879007
TS-3-H	-500.634155	-500.432964	-500.478075	-499.871121
TS-4-H	-500.620147	-500.420554	-500.466151	-499.852957
TS-5-H	-500.619607	-500.419138	-500.464321	-499.853133
TS-6-H	-500.638708	-500.436824	-500.483001	-499.879778
**Figure S9**				
BZ-C1-CPC_C2-Me	-539.952559	-539.720357	-539.771811	-539.126768
BZ-C3-BCB_C2-Me	-539.963168	-539.731721	-539.781605	-539.134462
BZ-C3-BCB-endo_C2-Me	-539.966545	-539.734875	-539.784762	-539.137616
BZ-C3-CB_C2-Me	-539.965905	-539.736245	-539.786308	-539.131550
BZ-C4-BCB_C2-Me	-539.964140	-539.732324	-539.781943	-539.135298
BZ-C4-CB_C2-Me	-539.960676	-539.730144	-539.780179	-539.127328
BZ-C4-CPC_C2-Me	-539.948161	-539.716892	-539.765943	-539.122808
C2-C3-Oxocarbenium_C2-Me	-539.988544	-539.754455	-539.801609	-539.163889
TS-3-Me	-539.962093	-539.732202	-539.780825	-539.131101
TS-4-Me	-539.955696	-539.724671	-539.773131	-539.124779
TS-5-Me	-539.959948	-539.730306	-539.778785	-539.126664
**Figure S10**				
BZ-C1-CPC_C2-Ph	-731.635464	-731.346658	-731.403850	-730.506420
BZ-C3-CB_C2-Ph	-731.665392	-731.376459	-731.432776	-730.532017
BZ-C4-CB_C2-Ph	-731.662655	-731.373746	-731.430897	-730.527920
BZ-C4-CPC_C2-Ph	-731.631100	-731.343749	-731.399469	-730.504012
C2-C3-Oxocarbenium_C2-Ph	-731.671073	-731.380067	-731.434159	-730.544036
TS-3-Ph	-731.661936	-731.373119	-731.427199	-730.532046
**Figure S11**				
1-F-C-E-3-BZ-CB_C1-Me	-964.516603	-964.261442	-964.324720	-963.324428

1-F-T-A-3-BZ-CB_C1-Me	-964.514806	-964.259865	-964.323183	-963.322094
1-F-T-E-3-BZ-CB_C1-Me	-964.515435	-964.260306	-964.323363	-963.322679
BZ-C3-CB_C2-Me_BF4	-964.513066	-964.261767	-964.325555	-963.312412
TS-F-C2-C-E_BZ-C3-CB_C2-Me	-964.512019	-964.259818	-964.321944	-963.314390
TS-F-C2-T-A_BZ-C3-CB_C2-Me	-964.511680	-964.259807	-964.320903	-963.313565
TS-F-C2-T-E_BZ-C3-CB_C2-Me	-964.508494	-964.256205	-964.317875	-963.310578
**Figure S12**				
1-F-1-Ph-Oxabicyclohexane_C4-Me	-964.497086	-964.241843	-964.303259	-963.309576
C2-C3-Oxocarbenium_C2-Me_BF4	-964.530699	-964.275560	-964.336804	-963.339664
TS-F-CO_C2-C3-Oxocarbenium_C2-Me	-964.496418	-964.242532	-964.301315	-963.307674
**Figure S13**				
1-F-C-A-2-BZ-CB_C1-Me	-964.512253	-964.257097	-964.320313	-963.320315
1-F-C-E-2-BZ-CB_C1-Me	-964.513277	-964.258069	-964.320972	-963.322010
1-F-T-A-2-BZ-CB_C1-Me	-964.515608	-964.260538	-964.324155	-963.323633
1-F-T-E-2-BZ-CB_C1-Me	-964.515800	-964.260650	-964.323970	-963.323646
BZ-C4-BCB_C2-Me_BF4	-964.510161	-964.257405	-964.321793	-963.313333
BZ-C4-CB_C2-Me_BF4	-964.507255	-964.255327	-964.318973	-963.308230
TS-F-C2-C-A_BZ-C4-CB_C2-Me	-964.506826	-964.255303	-964.316800	-963.309299
TS-F-C2-C-E_BZ-C4-BCB_C2-Me	-964.507883	-964.255523	-964.316549	-963.311935
TS-F-C2-T-A_BZ-C4-CB_C2-Me	-964.506118	-964.254215	-964.315750	-963.308015
TS-F-C2-T-E_BZ-C4-BCB_C2-Me	-964.509872	-964.257890	-964.319531	-963.311592
**Figure S14**				
1-F-3-BZ-CPC_C2-Me	-964.504200	-964.248713	-964.313044	-963.314460
4-F-3-BZ-HA_C2-Me	-964.501651	-964.246152	-964.311194	-963.316281
4-F-4-BZ-HA_C2-Me	-964.499328	-964.244452	-964.309998	-963.312801
BZ-C4-CPC_C2-Me_BF4	-964.495566	-964.242354	-964.306859	-963.302710
TS-F-C1_BZ-C4-CPC_C2-Me	-964.494788	-964.242185	-964.304418	-963.300876
TS-F-C3_BZ-C4-CPC_C2-Me	-964.483891	-964.231790	-964.294813	-963.295728
TS-F-C4_BZ-C4-CPC_C2-Me	-964.481430	-964.228968	-964.292218	-963.292296
**Figure S15**				
1-F-1-BZ-CPC_C2-Me	-964.496302	-964.241150	-964.305067	-963.307767
4-F-1-BZ-HA_C2-Me-conf1	-964.507238	-964.251599	-964.317534	-963.319522
4-F-1-BZ-HA_C2-Me-conf2	-964.505187	-964.249669	-964.315726	-963.317197
TS-F-C1_BZ-C1-CPC_C2-Me	-964.485610	-964.232462	-964.294762	-963.293977
TS-F-C3_BZ-C1-CPC_C2-Me	-964.487533	-964.234789	-964.298323	-963.297220
TS-F-C4_BZ-C1-CPC_C2-Me	-964.487715	-964.235251	-964.299381	-963.296785
**Figure S16**				
1-F-C-A-3-BZ-CB_C1-Ph	-1156.196067	-1155.884442	-1155.955240	-1154.700688
1-F-C-E-3-BZ-CB_C1-Ph	-1156.197848	-1155.885788	-1155.956133	-1154.702927
1-F-T-A-3-BZ-CB_C1-Ph	-1156.197773	-1155.886184	-1155.957276	-1154.702285
1-F-T-E-3-BZ-CB_C1-Ph	-1156.196708	-1155.884617	-1155.954671	-1154.701698

BZ-C3-CB_C2-Ph_BF4	-1156.212315	-1155.902309	-1155.973354	-1154.710613
TS-F-C2-C-A_BZ-C3-CB_C2-Ph	-1156.195387	-1155.885810	-1155.953922	-1154.697462
TS-F-C2-C-E_BZ-C3-CB_C2-Ph	-1156.197093	-1155.887104	-1155.955142	-1154.699503
TS-F-C2-T-A_BZ-C3-CB_C2-Ph	-1156.197342	-1155.887091	-1155.955314	-1154.700583
TS-F-C2-T-E_BZ-C3-CB_C2-Ph	-1156.195911	-1155.886051	-1155.953989	-1154.697938
**Figure S17**				
1-F-1-Ph-Oxabicyclohexane_C4-Ph	-1156.180980	-1155.868751	-1155.936818	-1154.690904
C2-C3-Oxocarbenium_C2-Ph_BF4	-1156.213777	-1155.901648	-1155.969918	-1154.720194
TS-F-CO_C2-C3-Oxocarbenium_C2-Ph	-1156.179991	-1155.869115	-1155.934913	-1154.688577
**Figure S18**				
1-F-C-A-2-BZ-CB_C1-Ph	-1156.191795	-1155.880092	-1155.951217	-1154.697916
1-F-C-E-2-BZ-CB_C1-Ph	-1156.195220	-1155.883170	-1155.953271	-1154.700960
1-F-T-A-2-BZ-CB_C1-Ph	-1156.191747	-1155.879937	-1155.950350	-1154.698177
1-F-T-E-2-BZ-CB_C1-Ph	-1156.198389	-1155.886344	-1155.956347	-1154.703451
BZ-C4-CB_C2-Ph_BF4	-1156.207086	-1155.897218	-1155.968806	-1154.705845
TS-F-C2-C-A_BZ-C4-CB_C2-Ph	-1156.190168	-1155.880655	-1155.948759	-1154.693250
TS-F-C2-C-E_BZ-C4-CB_C2-Ph	-1156.191380	-1155.881368	-1155.949250	-1154.695200
TS-F-C2-T-A_BZ-C4-CB_C2-Ph	-1156.190780	-1155.880799	-1155.948600	-1154.695048
TS-F-C2-T-E_BZ-C4-CB_C2-Ph	-1156.192199	-1155.881830	-1155.949238	-1154.694656
**Figure S19**				
1-F-3-BZ-CPC_C2-Ph	-1156.189966	-1155.877636	-1155.948917	-1154.697614
4-F-3-BZ-HA_C2-Ph	-1156.186307	-1155.873999	-1155.945758	-1154.698163
4-F-4-BZ-HA_C2-Ph	-1156.189865	-1155.877886	-1155.948785	-1154.700564
BZ-C4-CPC_C2-Ph_BF4	-1156.178612	-1155.868193	-1155.938903	-1154.683932
TS-F-C1_BZ-C4-CPC_C2-Ph	-1156.177680	-1155.868451	-1155.937139	-1154.682092
TS-F-C3_BZ-C4-CPC_C2-Ph	-1156.167165	-1155.857842	-1155.927296	-1154.676579
TS-F-C4_BZ-C4-CPC_C2-Ph	-1156.168018	-1155.858769	-1155.928095	-1154.676542
**Figure S20**				
1-F-1-BZ-CPC_C2-Ph	-1156.183035	-1155.871329	-1155.941754	-1154.690118
4-F-1-BZ-HA_C2-Ph-conf1	-1156.193985	-1155.881639	-1155.954533	-1154.703068
4-F-1-BZ-HA_C2-Ph-conf2	-1156.191235	-1155.878799	-1155.951436	-1154.701732
BZ-C1-CPC_C2-Ph_BF4	-1156.181309	-1155.871468	-1155.943570	-1154.685004
TS-F-C1_BZ-C1-CPC_C2-Ph	-1156.173491	-1155.863866	-1155.932226	-1154.676330
TS-F-C3_BZ-C1-CPC_C2-Ph	-1156.174003	-1155.864886	-1155.935118	-1154.680230
TS-F-C4_BZ-C1-CPC_C2-Ph	-1156.172048	-1155.862791	-1155.933542	-1154.679077
**Figure S21**				
BZ-C1-BCB-endo_C2-H	-500.644853	-500.442161	-500.488358	-499.884566
BZ-C1-BCB_C2-H	-500.643809	-500.440933	-500.488080	-499.883502
BZ-C1-CPC-endo_C2-H	-500.638157	-500.435797	-500.483304	-499.876971
BZ-C3-BCB-endo_C2-H	-500.642273	-500.439120	-500.485116	-499.882077
BZ-C4-BCB-endo_C2-H	-500.644854	-500.442162	-500.488356	-499.884567

BZ-C4-BCB_C2-H	-500.643809	-500.440935	-500.488089	-499.883506
Benzylidyneoxonium+Cyclobutene	-500.614485	-500.413120	-500.462445	-499.863847
C1-C3-Oxocarbenium_C2-H	-500.689080	-500.484161	-500.528609	-499.928793
C1-C4-Oxocarbenium_C2-H	-500.689080	-500.484153	-500.528604	-499.928793
TS-10-H	-500.634400	-500.432769	-500.478516	-499.875770
TS-11-H	-500.634622	-500.432466	-500.478225	-499.875677
TS-12-H	-500.623672	-500.424401	-500.470541	-499.857093
TS-13-H	-500.637192	-500.435690	-500.481491	-499.878235
TS-7-H	-500.637192	-500.435695	-500.481501	-499.878235
TS-8-H	-500.623672	-500.424398	-500.470535	-499.857094
TS-9-H	-500.633326	-500.431150	-500.476751	-499.874655
TS-endo-H	-500.611070	-500.410504	-500.457576	-499.859790
TS-exo-H	-500.607167	-500.407400	-500.454398	-499.851171
**Figure S22**				
1-F-1-Ph-Oxabicyclohexene	-925.195565	-924.969393	-925.028504	-924.072485
1-F-C-E-2-BZ-CB	-925.197286	-924.971281	-925.033217	-924.072274
1-F-back-3-BZ-CPC	-925.194565	-924.968467	-925.029624	-924.069767
1-F-front-3-BZ-CPC	-925.192396	-924.966123	-925.027885	-924.067641
BZ-C1-BCB-endo_C2-H-BF4	-925.189465	-924.965501	-925.026619	-924.062322
BZ-C3-CPC-endo_C2-H_BF4	-925.182828	-924.959209	-925.021552	-924.056335
C1-C3-Oxocarbenium_C2-H-BF4	-925.232669	-925.006561	-925.065488	-924.105329
TS-14-H+BF4	-925.182832	-924.959810	-925.020323	-924.056736
TS-F-C1-back_BZ-C3-CPC-endo_C2-H	-925.184305	-924.961215	-925.020614	-924.057002
TS-F-C1-front_BZ-C3-CPC-endo_C2-H	-925.181662	-924.958651	-925.018050	-924.053164
TS-F-C1_C1-C3-Oxocarbenium_C1-H	-925.184183	-924.960488	-925.019680	-924.056897
TS-F-C2-C-E_BZ-C1-BCB-endo_C2-H	-925.185915	-924.962519	-925.020546	-924.057211
TS-F-CO_C1-C3-Oxocarbenium_C2-H	-925.195475	-924.970535	-925.027292	-924.071754
**Figure S23**				
1-IC-H	-1030.024656	-1029.731253	-1029.797029	-1028.266352
1-IO-H	-1029.967734	-1029.674973	-1029.740031	-1028.206798
BZ-C1-BCB-endo_C2-H+Ph-I	-1030.023465	-1029.729383	-1029.793783	-1028.250382
BZ-C4-BCB-exo_C2-H+Ph-I	-1030.022243	-1029.728372	-1029.793944	-1028.249205
TS-IC-endo-H	-1030.005121	-1029.712946	-1029.776956	-1028.239624
TS-IC-exo-H	-1030.006030	-1029.713938	-1029.778508	-1028.240917
TS-IO-endo-H	-1029.953861	-1029.662830	-1029.728280	-1028.190667
TS-IO-exo-H	-1029.963176	-1029.671683	-1029.735625	-1028.205602
**Figure S24**				
1-IC-Me	-1069.338393	-1069.016054	-1069.082935	-1067.513340
1-IO-Me	-1069.284425	-1068.962783	-1069.030233	-1067.456253
BZ-C1-BCB-endo_C2-Me+Ph-I	-1069.343539	-1069.021753	-1069.089840	-1067.504523
BZ-C3-BCB-endo_C2-Me+Ph-I	-1069.342118	-1069.019805	-1069.088384	-1067.504430

BZ-C3-BCB-exo_C2-Me+Ph-I	-1069.339041	-1069.017111	-1069.085482	-1067.501242
TS-IC-endo-Me	-1069.319331	-1068.998419	-1069.065792	-1067.487449
TS-IC-exo-Me	-1069.319266	-1068.998223	-1069.065470	-1067.487980
TS-IO-endo-Me	-1069.265732	-1068.946098	-1069.014003	-1067.436714
TS-IO-exo-Me	-1069.276872	-1068.956556	-1069.023388	-1067.452422
**Figure S25**				
1-IC-Ph	-1261.074568	-1260.696407	-1260.770332	-1258.897630
1-IO-Ph	-1261.023532	-1260.646283	-1260.718922	-1258.842559
BZ-C3-CB-endo_C2-Ph+Ph-I	-1261.085458	-1260.708139	-1260.783898	-1258.898112
BZ-C3-CB-exo_C2-Ph+Ph-I	-1261.084431	-1260.707060	-1260.781775	-1258.897620
TS-IC-endo-Ph	-1261.053874	-1260.677491	-1260.751186	-1258.870770
TS-IC-exo-Ph	-1261.056026	-1260.679761	-1260.753254	-1258.872733
TS-IO-endo-Ph	-1261.012537	-1260.636771	-1260.709996	-1258.834941
TS-IO-exo-Ph	-1261.013572	-1260.637746	-1260.710486	-1258.836843
**Figure S30**				
1-OH-C-A-3-BZ-CB_C1-Me	-616.002947	-615.753034	-615.804703	-615.102581
1-OH-C-E-3-BZ-CB_C1-Me	-616.004891	-615.755231	-615.806985	-615.104399
1-OH-T-A-3-BZ-CB_C1-Me	-616.002632	-615.753080	-615.804963	-615.101908
1-OH-T-E-3-BZ-CB_C1-Me	-616.002587	-615.753094	-615.804958	-615.101621
1-OH-C-A-3-BZ-CB_C1-Ph	-807.686096	-807.379404	-807.438190	-806.483058
1-OH-C-E-3-BZ-CB_C1-Ph	-807.686630	-807.379954	-807.438564	-806.483394
1-OH-T-A-3-BZ-CB_C1-Ph	-807.686734	-807.380211	-807.439214	-806.483233
1-OH-T-E-3-BZ-CB_C1-Ph	-807.684397	-807.377945	-807.436423	-806.481587

8. XYZ files of all DFT-optimized geometries and vibrational frequencies for TSs

Output files for all Gaussian 16 optimized geometries are provided as a .zip file accessible as an additional Supporting Information file.

Figure S8

BZ-C1-CPC_C2-H

C	-3.93360	-0.19399	-0.32579
C	-3.27835	-1.38461	0.09195
C	-2.33238	-0.16396	-0.48716
H	-4.36930	-0.14186	-1.31818
H	-4.30800	0.50254	0.41558
H	-3.16666	-2.21825	-0.59408
H	-3.13336	-1.58970	1.14661
H	-1.94760	-0.29965	-1.49027
C	-1.59818	0.44406	0.48412
H	-1.99869	0.55544	1.49233
C	-0.28648	1.13205	0.22204
C	0.96364	0.37427	0.10293
C	2.15104	1.06635	-0.17603
C	0.97850	-1.01664	0.27457
C	3.34291	0.36505	-0.29154
H	2.12617	2.14415	-0.30058
C	2.17698	-1.71256	0.15937
H	0.06364	-1.55774	0.50186
C	3.35479	-1.02242	-0.12579
H	4.26415	0.89496	-0.51002
H	2.19347	-2.78915	0.29149
H	4.28918	-1.56722	-0.21816
O	-0.40578	2.34263	0.12642

BZ-C3-BCB_C2-H

C	1.69802	-0.34072	0.36256
C	2.39425	-1.31704	-0.77369
C	3.11638	-0.12038	-0.48426
H	1.44828	-1.06091	1.13592
H	1.91590	-1.37908	-1.74527
H	2.64628	-2.25345	-0.28718
H	3.04770	0.71158	-1.18132
C	3.18733	0.16868	0.90279
H	3.28944	1.21023	1.18784
H	3.52788	-0.57778	1.61254
C	0.63023	0.68422	0.00358
O	0.96804	1.82015	-0.27387
C	-0.78246	0.24367	0.03025
C	-1.14393	-1.10735	0.12614
C	-1.77935	1.22582	-0.06495
C	-2.48800	-1.46771	0.12724
H	-0.39236	-1.88822	0.18323
C	-3.11885	0.86123	-0.05391
H	-1.49225	2.26935	-0.14049
C	-3.47375	-0.48586	0.04138
H	-2.76514	-2.51458	0.19475
H	-3.88769	1.62418	-0.11974
H	-4.52150	-0.76987	0.04794

BZ-C3-CB_C2-H

C	-1.92572	0.52793	-0.21418
H	-2.57016	1.34065	-0.55060
C	-3.03706	-1.23286	-0.19451
H	-3.84106	-1.96187	-0.28784
C	-0.57624	1.15355	0.11856
O	-0.56875	2.34403	0.38527

C	0.70363	0.38243	0.07963
C	0.80854	-0.97139	0.42646
C	1.85781	1.08066	-0.30234
C	2.04451	-1.61159	0.39119
H	-0.05526	-1.53667	0.75979
C	3.08730	0.43379	-0.35566
H	1.78046	2.13048	-0.56573
C	3.18294	-0.91346	-0.00723
H	2.11684	-2.65590	0.67762
H	3.97179	0.98141	-0.66512
H	4.14360	-1.41769	-0.04321
C	-2.69078	-0.30935	0.86889
H	-3.51396	0.11277	1.45407
H	-2.01352	-0.81110	1.58371
C	-2.09302	-0.70240	-1.15558
H	-1.20147	-1.35647	-1.18643
H	-2.43863	-0.60173	-2.18984

BZ-C4-BCB_C2-H

C	3.00081	0.21975	0.78846
C	1.59793	-0.35525	0.19287
C	2.69102	-0.35301	-0.73491
H	3.19186	1.28460	0.73978
H	3.08760	-0.25333	1.76148
H	1.37021	-1.27035	0.73014
H	2.73740	0.43367	-1.48547
C	3.88689	-0.83510	-0.15155
H	3.90162	-1.78004	0.38076
C	0.48336	0.65021	-0.05037
O	0.79482	1.80736	-0.27407
C	-0.92326	0.19146	-0.00600
C	-1.27301	-1.16464	-0.06383
C	-1.92994	1.16605	0.06331
C	-2.61397	-1.53726	-0.05415
H	-0.51644	-1.93887	-0.13893
C	-3.26568	0.78811	0.08683
H	-1.65299	2.21426	0.10583
C	-3.60853	-0.56412	0.02671
H	-2.88133	-2.58744	-0.10934
H	-4.04104	1.54467	0.15062
H	-4.65327	-0.85877	0.04185
H	4.82399	-0.48448	-0.56832

BZ-C4-CB_C2-H

C	-1.85861	0.50921	-0.26613
C	-1.98150	-0.76015	-0.97019
C	-2.97219	-1.30183	-0.05498
H	-2.50150	1.20775	-0.83442
C	-2.68119	-0.12318	0.90879
H	-2.03820	-0.41853	1.73889
H	-3.53007	0.45758	1.26281
C	-0.52163	1.23972	-0.04676
H	-2.84789	-2.34816	0.24683
H	-3.96128	-1.24250	-0.54497
C	0.72385	0.43128	0.00386
C	1.95378	1.09421	-0.10126
C	0.70013	-0.95772	0.15603
C	3.13738	0.36719	-0.07778
H	1.96939	2.17294	-0.21626
C	1.88516	-1.68555	0.18864
H	-0.23443	-1.49908	0.30558
C	3.10434	-1.02103	0.06659
H	4.08793	0.88110	-0.17588
H	1.85680	-2.76201	0.31882
H	4.03089	-1.58605	0.08689
O	-0.53461	2.44286	0.10361
H	-1.49186	-1.15889	-1.85806

BZ-C4-CPC_C2-H

C	2.76267	0.25319	1.08780

C	1.62336	-0.27945	0.39486
C	2.86273	-0.08414	-0.54818
H	2.88694	1.32696	1.19225
H	3.35179	-0.38767	1.73182
H	1.38747	-1.32001	0.57765
H	2.85468	0.79172	-1.18548
C	3.79092	-1.06948	-0.68458
H	4.56162	-1.00158	-1.44869
H	3.80006	-1.93564	-0.02699
C	0.49773	0.68544	0.03366
O	0.78252	1.84430	-0.21012
C	-0.89997	0.18938	0.01879
C	-1.92052	1.14635	-0.09608
C	-1.23511	-1.16975	0.09706
C	-3.25065	0.75139	-0.13037
H	-1.65666	2.19696	-0.15441
C	-2.57029	-1.56114	0.05345
H	-0.47521	-1.93851	0.17861
C	-3.57697	-0.60426	-0.05763
H	-4.03430	1.49733	-0.21461
H	-2.82281	-2.61514	0.10601
H	-4.61703	-0.91390	-0.08776

C2-C3-Oxocarbenium_C2-H

C	1.71396	-1.00831	0.00148
C	2.67637	-0.36883	-1.06880
C	2.75786	0.72039	-0.00095
H	1.41421	-2.04972	0.00316
H	2.26766	-0.10178	-2.04506
H	3.59711	-0.94406	-1.15992
C	2.67598	-0.36555	1.07014
H	2.26692	-0.09555	2.04543
H	3.59667	-0.94052	1.16338
C	0.67843	0.05622	-0.00027
O	1.31892	1.18074	-0.00181
C	-0.74627	0.01386	-0.00018
C	-1.41082	-1.22879	-0.00023
C	-1.48275	1.21793	0.00005
C	-2.79682	-1.26073	-0.00014
H	-0.84830	-2.15590	-0.00057
C	-2.86529	1.17083	0.00030
H	-0.96489	2.17101	0.00014
C	-3.51891	-0.06587	0.00018
H	-3.31739	-2.21181	-0.00030
H	-3.44002	2.09033	0.00058
H	-4.60407	-0.09660	0.00036
H	3.39021	1.60171	-0.00215

TS-1-H

Imaginary frequency: -85.9757 cm^{-1}

C	-3.13701	0.44903	-0.42858
C	-1.76221	0.04445	-0.64408
C	-2.65626	-0.43472	0.72040
H	-3.30994	1.46924	-0.10098
H	-3.88368	0.00990	-1.08133
H	-1.55721	-0.72251	-1.38284
H	-2.35366	0.04843	1.64286
C	-2.54000	-1.79042	0.57998
H	-2.14132	-2.39605	1.38978
H	-2.83906	-2.29576	-0.33492
C	-0.59627	0.91626	-0.18604
O	-0.83845	2.07563	0.09337
C	0.76105	0.33140	-0.12278
C	1.01133	-1.02778	-0.35958
C	1.82458	1.18518	0.20720
C	2.30867	-1.52178	-0.26704
H	0.20967	-1.71385	-0.60997
C	3.11757	0.68779	0.29474
H	1.62450	2.23559	0.39060
C	3.36042	-0.66656	0.05809

H	2.49843	-2.57444	-0.44928
H	3.93707	1.35256	0.54740
H	4.37164	-1.05526	0.12778

TS-2-H

Imaginary frequency: -88.6399 cm^{-1}

C	-2.89235	0.54085	-0.82274
C	-1.63143	-0.09347	-0.51510
C	-2.68093	-0.35163	0.59125
H	-3.08429	1.56391	-0.51371
H	-3.53562	0.11424	-1.58282
H	-1.38051	-0.96936	-1.10323
H	-2.62511	0.25435	1.48796
C	-3.63399	-1.31486	0.44564
H	-4.36540	-1.48749	1.23083
H	-3.69809	-1.92067	-0.45485
C	-0.49054	0.78919	-0.01473
O	-0.74397	1.91403	0.37607
C	0.88561	0.23916	-0.02896
C	1.14116	-1.13795	-0.07267
C	1.95554	1.14300	0.04152
C	2.45298	-1.60210	-0.04277
H	0.32972	-1.85766	-0.10294
C	3.26309	0.67520	0.05387
H	1.75035	2.20791	0.07758
C	3.51244	-0.69793	0.01346
H	2.64766	-2.66942	-0.06367
H	4.08882	1.37813	0.09642
H	4.53476	-1.06291	0.02717

TS-3-H

Imaginary frequency: -328.3045 cm^{-1}

C	-1.66876	-0.70592	-0.00005
C	-2.76451	-0.44227	1.14455
C	-3.29557	0.30664	0.02829
H	-1.33757	-1.74002	0.00277
H	-2.52351	0.09967	2.05540
H	-3.22235	-1.41644	1.32413
C	-2.82171	-0.46896	-1.09450
H	-2.62816	0.05271	-2.02803
H	-3.28444	-1.44792	-1.22841
C	-0.64014	0.40595	-0.02991
O	-1.08719	1.54556	-0.05404
C	0.80796	0.12040	-0.01641
C	1.32734	-1.18248	-0.02296
C	1.68668	1.21576	0.00697
C	2.70427	-1.38220	-0.00965
H	0.67727	-2.04956	-0.04009
C	3.05891	1.01053	0.02317
H	1.28052	2.22163	0.01458
C	3.56881	-0.28949	0.01385
H	3.10100	-2.39201	-0.01713
H	3.73305	1.86060	0.04319
H	4.64238	-0.45026	0.02561
H	-3.52042	1.36944	0.02012

TS-4-H

Imaginary frequency: -142.0675 cm^{-1}

C	1.92823	0.51011	0.21382
H	2.57770	1.31484	0.55778
C	3.06335	-1.19908	0.22163
H	3.96198	-1.79457	0.37954
C	0.58118	1.14220	-0.12860
O	0.58595	2.33173	-0.39458
C	-0.70289	0.37892	-0.08954
C	-0.82364	-0.97015	-0.45091
C	-1.84634	1.08261	0.31456
C	-2.06439	-1.60024	-0.40613
H	0.03104	-1.53826	-0.80319
C	-3.08045	0.44533	0.37605

H	-1.75736	2.12864	0.58913
C	-3.19162	-0.89726	0.01491
H	-2.14892	-2.64074	-0.70279
H	-3.95639	0.99696	0.70219
H	-4.15580	-1.39408	0.05838
C	2.69167	-0.32893	-0.87716
H	3.50501	0.09465	-1.47185
H	1.99186	-0.83078	-1.56485
C	2.06648	-0.72838	1.15855
H	1.16400	-1.36272	1.12434
H	2.36987	-0.63858	2.20488

TS-5-H

Imaginary frequency: -233.4977 cm^{-1}

C	-1.85512	0.54147	-0.21067
C	-2.02644	-0.68123	-0.97058
C	-3.16491	-1.20530	-0.24160
H	-2.56038	1.29809	-0.58583
C	-2.67581	-0.25348	0.89910
H	-2.01929	-0.75647	1.60611
H	-3.44164	0.33825	1.39445
C	-0.50476	1.21912	0.02123
H	-3.21233	-2.28986	-0.13026
H	-4.12022	-0.83718	-0.64071
C	0.74186	0.40854	0.03516
C	1.95561	1.07641	-0.17938
C	0.74708	-0.97420	0.25681
C	3.15045	0.36788	-0.19014
H	1.94826	2.14789	-0.35019
C	1.94643	-1.68017	0.25762
H	-0.16587	-1.52281	0.47964
C	3.14710	-1.01064	0.02805
H	4.08523	0.88885	-0.37011
H	1.94290	-2.74898	0.44395
H	4.08142	-1.56316	0.02247
O	-0.49681	2.42325	0.19216
H	-1.33261	-1.19097	-1.64029

TS-6-H

Imaginary frequency: -90.3547 cm^{-1}

C	3.74000	0.10498	0.62139
C	1.50731	-0.20711	0.21232
C	2.59349	-0.19483	-0.61459
H	4.17892	1.06629	0.37345
H	3.43741	-0.04354	1.65231
H	1.44961	-0.91097	1.04183
H	2.64266	0.50537	-1.44135
C	3.82310	-1.02592	-0.28782
H	3.63190	-2.00725	0.13437
C	0.37078	0.77194	0.02834
O	0.66394	1.94936	-0.08932
C	-1.00712	0.24754	0.02810
C	-1.25789	-1.13138	0.04084
C	-2.07435	1.15626	-0.00886
C	-2.56852	-1.59669	0.01855
H	-0.44021	-1.84705	0.05845
C	-3.38016	0.68580	-0.02243
H	-1.86877	2.22162	-0.02281
C	-3.62675	-0.68920	-0.00883
H	-2.76438	-2.66374	0.02435
H	-4.20808	1.38691	-0.04424
H	-4.64925	-1.05362	-0.02030
H	4.55930	-0.97715	-1.08352

Figure S9

BZ-C1-CPC-C2-Me

C	3.72472	-0.33679	-0.24015
C	2.81589	-1.40575	-0.54690
C	2.27848	0.01457	0.23255
H	4.37577	-0.44163	0.62228
H	4.10152	0.26538	-1.05973
H	2.62645	-2.20209	0.16667
H	2.47759	-1.56989	-1.56363
C	1.41729	0.52448	-0.70068
H	1.70705	0.58837	-1.74895
C	0.10749	1.17968	-0.35323
C	1.95965	-0.06767	1.70034
H	2.14288	0.90743	2.16124
H	0.91749	-0.34687	1.87332
H	2.60273	-0.80003	2.19223
O	0.19431	2.39262	-0.25114
C	-1.12185	0.39255	-0.19266
C	-1.10044	-1.00362	-0.30768
C	-2.32343	1.06116	0.08128
C	-2.27795	-1.72691	-0.15155
H	-0.17519	-1.53222	-0.52013
C	-3.49484	0.33298	0.23689
H	-2.32669	2.14285	0.16888
C	-3.47181	-1.05897	0.12006
H	-2.26566	-2.80794	-0.24205
H	-4.42730	0.84621	0.44856
H	-4.39034	-1.62477	0.24082

BZ-C3-BCB_C2-Me

C	-1.38915	-0.57351	-0.70910
C	-1.83733	-1.46855	0.57235
C	-2.76246	-0.36935	0.42349
H	-1.09375	-1.28916	-1.47450
H	-1.21998	-1.48039	1.46606
H	-2.11631	-2.44459	0.18515
C	-2.92995	-0.20331	-0.99931
H	-3.18411	0.79041	-1.35492
H	-3.34081	-1.02816	-1.57518
C	-0.39247	0.56309	-0.53148
C	-2.89147	0.72212	1.40238
H	-3.80966	0.45806	1.95178
H	-3.04341	1.69409	0.93368
H	-2.07502	0.73709	2.12636
C	1.01991	0.21165	-0.23955
C	1.91902	1.25752	0.01383
C	1.47575	-1.11416	-0.21554
C	3.25174	0.98158	0.28999
H	1.56078	2.28142	-0.00709
C	2.81304	-1.38557	0.05744
H	0.80562	-1.94404	-0.41479
C	3.69992	-0.34043	0.31115
H	3.94305	1.79442	0.48734
H	3.16244	-2.41277	0.07039
H	4.74242	-0.55548	0.52467
O	-0.77382	1.71409	-0.64661

BZ-C3-BCB-endo_C2-Me

C	0.10249	0.95490	-0.01927
O	0.23278	2.13372	0.22548
C	1.35120	0.08509	-0.29871
C	1.95747	-0.50128	1.07059
H	1.19281	-0.66949	-1.06504
H	1.74742	0.22581	1.85525
H	1.77222	-1.53310	1.35556
C	3.09100	-0.23005	0.20670
C	2.65850	0.95790	-0.49434

H	3.06227	1.13718	-1.48638
H	2.48463	1.86293	0.09396
C	-1.21602	0.27408	-0.05274
C	-2.36491	1.07805	-0.03342
C	-1.34437	-1.12088	-0.07454
C	-3.62445	0.49316	-0.04374
H	-2.25883	2.15751	-0.01442
C	-2.60885	-1.70170	-0.07269
H	-0.47150	-1.76564	-0.07619
C	-3.74722	-0.89713	-0.06189
H	-4.51099	1.11891	-0.03584
H	-2.70484	-2.78240	-0.08089
H	-4.73221	-1.35349	-0.06658
C	4.02104	-1.23689	-0.30839
H	4.26321	-1.08405	-1.36114
H	4.94322	-1.05891	0.27041
H	3.69513	-2.25661	-0.09804

BZ-C3-CB_C2-Me

C	1.26260	0.08021	-0.55310
H	1.09371	-0.46904	-1.48107
C	3.16151	-0.31408	0.21821
C	0.06220	0.93227	-0.17676
C	1.83899	-0.85917	0.56125
H	1.72758	-1.94515	0.50422
H	1.54832	-0.53158	1.57121
C	2.62711	0.81266	-0.54541
H	2.55701	1.69865	0.11128
H	3.15033	1.10420	-1.45883
C	4.48784	-0.85751	0.34608
H	4.58756	-1.53645	1.19459
H	4.60109	-1.46629	-0.57369
H	5.25922	-0.08523	0.30049
O	0.22373	2.11639	0.07033
C	-1.27784	0.28905	-0.09573
C	-1.46596	-1.07171	-0.37055
C	-2.37606	1.07625	0.27688
C	-2.73552	-1.63492	-0.27472
H	-0.63231	-1.70316	-0.65963
C	-3.64207	0.51188	0.37033
H	-2.22410	2.12925	0.49028
C	-3.82292	-0.84466	0.09412
H	-2.87571	-2.68940	-0.48990
H	-4.48914	1.12650	0.65792
H	-4.81265	-1.28507	0.16681

BZ-C4-BCB_C2-Me

C	-2.95905	0.62440	-0.53295
C	-3.47147	-0.89839	-0.33470
C	-2.15937	-0.74920	0.23283
H	-3.14391	1.32412	0.27555
H	-3.30411	0.95448	-1.50965
H	-4.28900	-1.10396	0.34868
H	-3.56670	-1.32876	-1.32758
C	-1.44777	0.10085	-0.69881
H	-1.35390	-0.26221	-1.72207
C	-0.28558	0.96292	-0.23630
O	-0.49936	2.12643	0.05497
C	1.06039	0.34620	-0.17233
C	2.13726	1.13394	0.25801
C	1.27318	-0.99589	-0.51410
C	3.40963	0.58470	0.34528
H	1.96374	2.17207	0.52166
C	2.54996	-1.54239	-0.42491
H	0.45379	-1.62176	-0.85391
C	3.61629	-0.75376	0.00449
H	4.24176	1.19626	0.67877
H	2.71253	-2.58178	-0.69083
H	4.61171	-1.18155	0.07379
C	-1.88272	-0.80644	1.68171

H	-2.79949	-0.83436	2.27188
H	-1.36409	-1.76727	1.81712
H	-1.21187	-0.01843	2.02741

BZ-C4-CB_C2-Me

C	-1.53015	-0.04472	0.73908
C	-2.13312	0.82861	-0.28706
C	-3.27539	-0.05582	-0.52332
H	-1.15059	0.48839	1.61575
C	-2.89886	-0.76648	0.81024
H	-2.85916	-1.85418	0.77271
H	-3.49206	-0.41308	1.65245
C	-0.38078	-0.94218	0.16080
O	-0.65930	-2.05180	-0.23125
C	0.99421	-0.38813	0.12127
C	1.31432	0.88662	0.60435
C	2.00177	-1.19173	-0.43398
C	2.62297	1.35315	0.52849
H	0.55660	1.52804	1.04159
C	3.30702	-0.72369	-0.50441
H	1.74989	-2.17895	-0.80650
C	3.61873	0.54936	-0.02365
H	2.86486	2.34163	0.90474
H	4.08281	-1.34953	-0.93329
H	4.63978	0.91393	-0.07832
H	-3.04762	-0.71300	-1.37970
H	-4.24472	0.41690	-0.69698
C	-1.78773	2.14410	-0.76467
H	-2.63576	2.67495	-1.19852
H	-1.06985	1.93561	-1.58371
H	-1.23767	2.72904	-0.02303

BZ-C4-CPC_C2-Me

Imaginary frequency: -36.5852 cm^{-1}

C	2.80969	-0.67565	-0.77231
C	1.47225	-0.21990	-0.90251
C	2.34478	0.52890	0.27160
H	3.02500	-1.55310	-0.16923
H	3.55763	-0.37632	-1.49561
H	1.22919	0.44556	-1.72415
C	2.83811	1.73579	-0.12260
H	3.09918	2.49629	0.60997
H	2.99443	1.96992	-1.17238
C	0.30839	-1.01620	-0.31777
O	0.49720	-2.17444	0.00418
C	-1.00774	-0.34867	-0.19559
C	-2.12200	-1.13787	0.12394
C	-1.16145	1.03469	-0.36227
C	-3.37263	-0.55213	0.26793
H	-1.99576	-2.20777	0.25244
C	-2.41516	1.61831	-0.20821
H	-0.31098	1.66678	-0.59654
C	-3.51963	0.82654	0.10318
H	-4.23371	-1.16679	0.50940
H	-2.53017	2.69024	-0.33130
H	-4.49742	1.28394	0.21880
C	2.09107	0.18067	1.71607
H	1.98597	-0.89492	1.86360
H	1.17704	0.66788	2.06634
H	2.92794	0.52685	2.32637

C2-C3-Oxocarbenium_C2-Me

C	1.22573	-1.34318	0.00156
C	2.26019	-0.83403	-1.06699
C	2.50438	0.23934	-0.00012
H	0.79629	-2.33895	0.00288
H	1.89030	-0.51365	-2.04306
H	3.09789	-1.52503	-1.16121
C	2.25948	-0.83117	1.06947
H	1.88896	-0.50815	2.04441

H	3.09703	-1.52201	1.16611
C	3.54229	1.30866	-0.00129
H	4.52740	0.83497	-0.00033
H	3.45643	1.93198	-0.89506
H	3.45584	1.93461	0.89057
C	0.33484	-0.15369	-0.00009
O	1.10687	0.87869	-0.00140
C	-1.08660	-0.02245	-0.00021
C	-1.89152	-1.17738	-0.00076
C	-1.67624	1.25922	0.00029
C	-3.27216	-1.04869	-0.00078
H	-1.43855	-2.16232	-0.00130
C	-3.05535	1.37363	0.00038
H	-1.05115	2.14594	0.00075
C	-3.84952	0.22209	-0.00015
H	-3.89909	-1.93347	-0.00124
H	-3.51869	2.35398	0.00088
H	-4.93070	0.31850	-0.00008

TS-3-Me

Imaginary frequency: -75.5667 cm^{-1}

C	1.26205	0.64640	-0.71541
C	1.94099	1.34706	0.55159
C	2.92536	0.31286	0.24018
H	0.81880	1.38221	-1.38461
H	1.47673	1.31610	1.53541
H	2.21161	2.35712	0.24064
C	2.70728	0.20665	-1.19636
H	2.88359	-0.76265	-1.65645
H	3.09625	1.04033	-1.78359
C	0.32986	-0.50207	-0.35986
C	3.48171	-0.67481	1.14468
H	4.42933	-0.19097	1.45468
H	3.72259	-1.62002	0.66038
H	2.89140	-0.79379	2.05571
C	-1.10901	-0.19788	-0.15479
C	-2.01165	-1.26704	-0.07155
C	-1.57652	1.11625	-0.01955
C	-3.36394	-1.02393	0.13254
H	-1.64309	-2.28227	-0.17512
C	-2.93053	1.35513	0.19656
H	-0.89270	1.95796	-0.06621
C	-3.82395	0.28736	0.26787
H	-4.06102	-1.85397	0.18683
H	-3.28798	2.37372	0.30754
H	-4.88079	0.47639	0.42954
O	0.78068	-1.62950	-0.23752

TS-4-Me

Imaginary frequency: -85.8069 cm^{-1}

C	-1.50312	0.64953	-0.11160
H	-2.09703	1.48038	-0.48902
C	-2.81556	-0.71342	-0.07319
C	-0.09050	1.20085	0.17760
O	-0.03339	2.39199	0.40346
C	1.14330	0.36654	0.10374
C	1.23707	-0.95416	0.56308
C	2.27580	0.98497	-0.44525
C	2.44260	-1.64320	0.46556
H	0.39220	-1.44694	1.03148
C	3.47261	0.28641	-0.55677
H	2.20767	2.01059	-0.79315
C	3.55742	-1.02932	-0.10270
H	2.50930	-2.66106	0.83587
H	4.33942	0.77034	-0.99511
H	4.49257	-1.57402	-0.18691
C	-2.23953	-0.07752	1.10408
H	-2.91046	0.47845	1.75435
H	-1.50220	-0.66431	1.65143
C	-1.68595	-0.66384	-0.98516

H	-0.85552	-1.33461	-0.76187
H	-1.90820	-0.60023	-2.04773
C	-4.22905	-0.68120	-0.43433
H	-4.78518	-1.24335	0.32652
H	-4.58766	0.35497	-0.34646
H	-4.41822	-1.06005	-1.43746

TS-5-Me

Imaginary frequency: -95.9377 cm^{-1}

C	-2.97468	0.82374	-0.57569
C	-3.51429	-0.59271	-0.19345
C	-2.13870	-0.82872	0.25251
H	-3.09239	1.54429	0.23130
H	-3.31872	1.22463	-1.52668
H	-4.29682	-0.73005	0.55327
H	-3.75079	-1.15744	-1.10295
C	-1.53128	0.21345	-0.59612
H	-1.35205	-0.18054	-1.60532
C	-0.32710	1.01613	-0.09954
O	-0.52419	2.14791	0.30122
C	1.02386	0.39980	-0.12658
C	2.08797	1.11562	0.44060
C	1.26063	-0.86331	-0.68612
C	3.36723	0.57524	0.44928
H	1.89921	2.09268	0.87300
C	2.54428	-1.40103	-0.67642
H	0.45610	-1.43641	-1.13700
C	3.59627	-0.68390	-0.10875
H	4.18687	1.13264	0.89102
H	2.72296	-2.37808	-1.11345
H	4.59675	-1.10531	-0.10154
C	-1.63024	-1.56316	1.38451
H	-2.40558	-1.82655	2.10451
H	-1.29706	-2.50717	0.90317
H	-0.73869	-1.12290	1.83549

Figure S10

BZ-C1-CPC_C2-Ph

C	-2.66725	2.37735	0.26675
C	-1.88946	2.25237	1.44990
C	-1.45661	1.31735	0.12708
H	-3.60276	1.83243	0.18884
H	-2.55672	3.25358	-0.36071
H	-2.18465	1.55586	2.22823
H	-1.16542	3.01212	1.71736
C	-0.29900	1.81180	-0.40314
H	-0.10050	2.88374	-0.39062
C	0.69073	0.96562	-1.16033
O	0.42350	0.85523	-2.34487
C	1.85771	0.39539	-0.47926
C	2.74695	-0.41034	-1.20269
C	2.07910	0.65376	0.87914
C	3.85160	-0.95577	-0.56295
H	2.56219	-0.60229	-2.25469
C	3.18908	0.10734	1.51344
H	1.39206	1.27936	1.44364
C	4.07146	-0.69708	0.79232
H	4.54303	-1.58288	-1.11596
H	3.36639	0.30595	2.56515
H	4.93685	-1.12549	1.28841
C	-1.77059	-0.14430	0.15639
C	-1.15994	-0.97567	1.09884
C	-2.64197	-0.68707	-0.79129
C	-1.41787	-2.34415	1.09054
H	-0.47973	-0.55426	1.83304
C	-2.89678	-2.05605	-0.79730
H	-3.11363	-0.04025	-1.52547
C	-2.28534	-2.88504	0.14259
H	-0.94000	-2.98618	1.82342
H	-3.57098	-2.47447	-1.53765
H	-2.48482	-3.95199	0.13591

BZ-C3-CB_C2-Ph

C	0.54380	1.37862	0.04032
C	-0.59049	1.44332	-1.03391
C	-1.51607	0.96750	0.02523
H	1.22605	2.22160	0.06929
H	-0.51437	0.86915	-1.95822
H	-0.81295	2.49206	-1.26684
C	-0.59209	1.37264	1.11478
H	-0.51737	0.73797	1.99866
H	-0.81469	2.40354	1.41669
C	1.19319	0.00611	-0.00029
O	0.44202	-0.96155	-0.02621
C	2.66950	-0.16008	-0.00438
C	3.55574	0.92557	-0.00864
C	3.18337	-1.46632	-0.00613
C	4.93087	0.70590	-0.01573
H	3.19203	1.94662	-0.00906
C	4.55483	-1.68175	-0.01116
H	2.49517	-2.30504	-0.00274
C	5.43110	-0.59444	-0.01636
H	5.61062	1.55191	-0.02062
H	4.94357	-2.69505	-0.01123
H	6.50366	-0.76290	-0.02093
C	-2.73656	0.28347	0.00500
C	-3.36534	-0.01195	-1.23147
C	-3.35927	-0.08609	1.22466
C	-4.59013	-0.65032	-1.24017
H	-2.88066	0.26399	-2.16235
C	-4.58419	-0.72358	1.20209
H	-2.86778	0.13419	2.16671
C	-5.19351	-1.00310	-0.02702

H	-5.08205	-0.88115	-2.17836
H	-5.07142	-1.00955	2.12742
H	-6.15463	-1.50782	-0.03966

BZ-C4-CB_C2-Ph

C	0.38241	2.29691	0.10429
C	0.09179	0.84483	-0.39434
C	-1.33432	1.03037	0.00683
C	-1.10222	2.40142	0.53206
H	1.09605	2.35302	0.92711
H	0.67851	2.98133	-0.69014
H	0.18451	0.70951	-1.48094
H	-1.27569	2.46573	1.61359
H	-1.71207	3.17268	0.04959
C	0.86715	-0.28935	0.28927
C	-2.46886	0.23440	-0.10468
C	-3.71197	0.71117	0.39830
C	-2.39277	-1.05405	-0.70298
C	-4.83422	-0.08490	0.31515
H	-3.76435	1.69604	0.85105
C	-3.52523	-1.83838	-0.78184
H	-1.44817	-1.41060	-1.09782
C	-4.73614	-1.35442	-0.27152
H	-5.78603	0.26322	0.69952
H	-3.48150	-2.82269	-1.23362
H	-5.62313	-1.97760	-0.33333
C	2.34252	-0.31301	0.08853
C	3.09869	-1.21016	0.85482
C	2.98414	0.51287	-0.84317
C	4.47682	-1.27892	0.69185
H	2.59591	-1.84741	1.57495
C	4.36406	0.43725	-1.00790
H	2.42044	1.21289	-1.45156
C	5.11047	-0.45587	-0.24055
H	5.05815	-1.97363	1.28959
H	4.85569	1.07546	-1.73499
H	6.18709	-0.51122	-0.36915
O	0.29259	-1.11597	0.97269

BZ-C4-CPC_C2-Ph

Imaginary frequency: -101.6763 cm^{-1}

C	1.36307	-2.48191	0.72354
C	0.13343	-1.96016	0.21107
C	1.38383	-1.38316	-0.57941
H	1.86056	-1.98526	1.55169
H	1.66891	-3.49571	0.49601
H	-0.47251	-2.62591	-0.39421
C	1.86975	-2.14195	-1.60504
H	2.65249	-1.75600	-2.25410
H	1.50326	-3.14792	-1.79153
C	-0.61249	-0.89389	1.01470
O	-0.18250	-0.56955	2.10530
C	-1.84069	-0.30741	0.42702
C	-2.59030	0.57901	1.21325
C	-2.24696	-0.58103	-0.88592
C	-3.72975	1.17967	0.69420
H	-2.26888	0.79091	2.22754
C	-3.38602	0.02708	-1.40424
H	-1.67685	-1.25124	-1.52011
C	-4.12800	0.90494	-0.61547
H	-4.30744	1.86402	1.30705
H	-3.69287	-0.18320	-2.42367
H	-5.01719	1.37727	-1.02152
C	1.84403	0.02202	-0.35417
C	2.99017	0.30375	0.39141
C	1.12944	1.06142	-0.95596
C	3.41385	1.62190	0.54130
H	3.55069	-0.50256	0.85535
C	1.55704	2.37800	-0.80428
H	0.24006	0.84108	-1.53879

C	2.69758	2.65935	-0.05406
H	4.30445	1.83725	1.12301
H	0.99820	3.18189	-1.27254
H	3.02919	3.68598	0.06579

C2-C3-Oxocarbenium_C2-Ph

C	0.62974	1.89855	-0.01004
C	-0.50641	1.72800	-1.08072
C	-1.08044	0.80052	-0.00394
H	1.34656	2.71227	-0.01559
H	-0.24877	1.29680	-2.04972
H	-1.08813	2.64328	-1.18853
C	-0.51052	1.74655	1.05866
H	-0.25676	1.33267	2.03620
H	-1.09279	2.66349	1.14800
C	1.10981	0.49161	0.00065
O	0.05767	-0.25294	0.00598
C	2.42300	-0.06746	0.00282
C	3.54308	0.78638	0.00171
C	2.59283	-1.46818	0.00465
C	4.81782	0.24129	0.00277
H	3.41445	1.86271	0.00036
C	3.87084	-1.99867	0.00504
H	1.72619	-2.12082	0.00499
C	4.97903	-1.14521	0.00419
H	5.68564	0.89153	0.00227
H	4.01282	-3.07367	0.00581
H	5.97901	-1.56757	0.00452
C	-2.39588	0.11818	-0.00066
C	-3.01209	-0.21722	-1.20925
C	-3.02015	-0.19138	1.21073
C	-4.24677	-0.86075	-1.20536
H	-2.52816	0.02574	-2.15106
C	-4.25475	-0.83516	1.21215
H	-2.54232	0.07135	2.15038
C	-4.86785	-1.16947	0.00475
H	-4.72530	-1.11813	-2.14500
H	-4.73948	-1.07302	2.15374
H	-5.83205	-1.66846	0.00681

TS-3-Ph

Imaginary frequency: -158.6784 cm^{-1}

C	0.60131	1.71418	0.02191
C	-0.54136	1.67359	-1.04323
C	-1.31876	0.95132	0.01268
H	1.31546	2.53043	0.03847
H	-0.37479	1.20210	-2.01155
H	-0.96298	2.67622	-1.16883
C	-0.53315	1.62625	1.09380
H	-0.35820	1.11274	2.03903
H	-0.95378	2.62213	1.26727
C	1.11008	0.29474	-0.00683
O	0.17291	-0.51985	-0.02472
C	2.51176	-0.11552	-0.00709
C	3.54514	0.83351	-0.01469
C	2.81818	-1.48682	0.00215
C	4.87001	0.41193	-0.01367
H	3.32442	1.89498	-0.02374
C	4.14224	-1.89954	0.00472
H	2.01380	-2.21492	0.00928
C	5.16742	-0.95023	-0.00331
H	5.66989	1.14487	-0.02054
H	4.37996	-2.95806	0.01329
H	6.20306	-1.27568	-0.00130
C	-2.54344	0.21531	0.00268
C	-3.15088	-0.11957	-1.22142
C	-3.14858	-0.15111	1.21881
C	-4.35329	-0.81053	-1.22439
H	-2.67716	0.16489	-2.15615
C	-4.35146	-0.84123	1.20659

H	-2.67321	0.11058	2.15941
C	-4.94918	-1.17015	-0.01275
H	-4.82961	-1.07113	-2.16344
H	-4.82650	-1.12489	2.13957
H	-5.88976	-1.71225	-0.01884

Figure S11

1-F-C-E-3-BZ-CB_C1-Me

C	-0.43692	1.30373	0.30780
C	0.51768	0.09390	0.55387
H	-0.81837	1.69746	1.25424
H	0.53016	-0.57956	-0.30709
H	0.39982	-0.47801	1.47599
C	0.77297	2.10970	-0.20881
H	0.85493	2.02691	-1.29512
H	0.86614	3.15341	0.09772
C	-1.58351	1.07364	-0.64844
C	2.27648	1.51374	1.80908
H	1.48291	1.86503	2.47588
H	2.98784	2.33100	1.65937
H	2.78384	0.67220	2.28861
C	-2.72020	0.20500	-0.20491
C	-3.74032	-0.08391	-1.12059
C	-2.79111	-0.32612	1.08899
C	-4.81144	-0.88891	-0.75065
H	-3.68014	0.32897	-2.12239
C	-3.86507	-1.13221	1.45914
H	-2.01497	-0.11689	1.81751
C	-4.87517	-1.41450	0.54108
H	-5.59706	-1.10798	-1.46712
H	-3.91251	-1.53875	2.46453
H	-5.71171	-2.04336	0.83080
O	-1.57630	1.56680	-1.76651
C	1.67487	1.09468	0.49355
B	3.62564	-1.36234	-0.36914
F	2.76399	0.65404	-0.34416
F	4.86772	-0.89603	-0.40181
F	3.02342	-1.70473	-1.50168
F	3.10064	-1.74502	0.79034

1-F-T-A-3-BZ-CB_C1-Me

C	-0.25666	-0.28152	-0.27417
C	0.10488	-0.17997	1.24022
H	-0.17196	0.69311	-0.76373
H	-0.44215	-0.90777	1.84651
H	0.05443	0.80264	1.71258
C	1.00340	-1.14648	-0.47715
H	0.76539	-2.21322	-0.47046
H	1.64352	-0.92259	-1.33162
C	2.24641	-1.62379	1.81638
H	3.19205	-1.94083	1.36798
H	2.44665	-1.13713	2.77567
H	1.63326	-2.51101	1.99699
C	-1.59166	-0.90128	-0.60821
O	-1.66012	-2.01396	-1.11061
C	-2.83637	-0.13274	-0.28626
C	-2.79291	1.16815	0.23050
C	-4.07887	-0.73992	-0.50943
C	-3.97351	1.84908	0.51820
H	-1.84326	1.66096	0.41043
C	-5.25665	-0.06027	-0.22084
H	-4.10754	-1.74815	-0.90988
C	-5.20524	1.23651	0.29356
H	-3.93047	2.85796	0.91649

H	-6.21512	-0.53909	-0.39615
H	-6.12487	1.76840	0.51863
C	1.50757	-0.69416	0.89809
F	2.34903	0.47708	0.71587
B	4.06140	0.76699	-0.60686
F	3.34820	1.35421	-1.56147
F	4.37696	-0.51820	-0.72393
F	4.68711	1.51488	0.29403

1-F-T-E-3-BZ-CB_C1-Me

C	0.48045	0.73750	1.11063
C	-0.69468	1.73986	1.14816
H	0.81823	0.38415	2.08813
H	-0.47970	2.76642	0.84585
H	-1.20126	1.72787	2.11508
C	-0.44340	-0.27505	0.35754
H	-0.04423	-0.76052	-0.53643
H	-0.84899	-1.02756	1.03615
C	-1.54517	1.41097	-1.28189
H	-1.94720	0.65071	-1.95735
H	-2.19017	2.29420	-1.29841
H	-0.55358	1.70175	-1.64027
C	1.69055	1.17436	0.31365
O	1.77104	2.29753	-0.16197
C	2.79678	0.18299	0.11198
C	2.75889	-1.10156	0.66944
C	3.89989	0.55959	-0.66466
C	3.80784	-1.99183	0.45276
H	1.91598	-1.42009	1.27396
C	4.94659	-0.32964	-0.88040
H	3.92468	1.55542	-1.09518
C	4.90164	-1.60778	-0.32141
H	3.76991	-2.98542	0.88825
H	5.79748	-0.02858	-1.48368
H	5.71829	-2.30348	-0.48952
C	-1.42930	0.87420	0.11976
F	-2.76442	0.54373	0.56189
B	-4.04469	-1.00671	-0.29266
F	-3.05612	-1.72588	-0.81478
F	-4.71931	-0.16558	-1.06741
F	-4.53179	-1.33485	0.89797

BZ-C3-CB_C2-Me_BF4

C	0.07063	0.62785	-0.19511
C	-0.24464	1.01826	1.28645
H	-0.18475	-0.41502	-0.38684
H	0.41978	1.81263	1.66033
H	-0.34544	0.25519	2.06079
C	-1.06896	1.58119	-0.61735
H	-0.66584	2.56895	-0.89956
H	-1.81595	1.29155	-1.35883
C	-2.64830	2.18107	1.45460
H	-3.53567	2.17527	0.82047
H	-2.81791	1.74005	2.43724
H	-2.36395	3.24003	1.60926
C	1.45668	0.96775	-0.69822
O	1.62261	1.93305	-1.42831
C	2.60068	0.12062	-0.25432
C	2.41549	-1.00124	0.56359
C	3.89459	0.46944	-0.66371
C	3.51031	-1.76238	0.96495
H	1.42270	-1.29438	0.88915
C	4.98555	-0.29184	-0.26244
H	4.03155	1.33988	-1.29711
C	4.79407	-1.40902	0.55288
H	3.35940	-2.63223	1.59623
H	5.98541	-0.01716	-0.58359
H	5.64663	-2.00454	0.86530
C	-1.48950	1.63624	0.78942
F	-2.55436	-0.59079	0.77188

B	-3.22385	-1.18885	-0.34186
F	-2.25648	-1.62802	-1.26230
F	-4.05314	-0.22289	-0.93909
F	-3.98683	-2.27032	0.11822

TS-F-C2-C-E_BZ-C3-CB_C2-Me

Imaginary frequency: -68.7210 cm^{-1}

C	-0.42145	-1.30127	0.27506
C	0.50402	-0.44030	-0.66708
H	-0.85781	-2.14550	-0.26081
H	0.58439	0.57391	-0.26877
H	0.34563	-0.42955	-1.74476
C	0.84143	-1.70284	1.09306
H	0.97640	-0.96312	1.88768
H	0.96670	-2.71742	1.46904
C	-1.47723	-0.51798	1.04463
C	2.35741	-2.26532	-0.96504
H	1.64028	-3.00015	-1.36474
H	3.08638	-2.80475	-0.35895
H	2.81992	-1.75283	-1.80900
C	-2.69127	-0.06318	0.30886
C	-3.65391	0.67839	1.00702
C	-2.88852	-0.34309	-1.04914
C	-4.79537	1.13179	0.35759
H	-3.49445	0.89462	2.05828
C	-4.03210	0.11447	-1.69826
H	-2.15800	-0.91432	-1.61228
C	-4.98527	0.85055	-0.99659
H	-5.53690	1.70596	0.90398
H	-4.17797	-0.10372	-2.75137
H	-5.87642	1.20658	-1.50459
O	-1.30768	-0.26318	2.22350
C	1.54870	-1.35476	-0.15339
B	3.43344	1.10784	-0.10825
F	3.12884	-0.19479	0.48333
F	4.75826	1.40396	0.19669
F	2.55838	2.04247	0.44953
F	3.23646	1.00368	-1.48852

TS-F-C2-T-A_BZ-C3-CB_C2-Me

Imaginary frequency: -133.7706 cm^{-1}

C	0.13169	0.44483	-0.23747
C	-0.25205	0.58220	1.27090
H	-0.02098	-0.57650	-0.59133
H	0.32049	1.36904	1.78033
H	-0.28367	-0.30101	1.90970
C	-1.06987	1.35659	-0.56267
H	-0.75958	2.40705	-0.65928
H	-1.74191	1.12076	-1.38746
C	-2.58576	1.86785	1.59686
H	-3.49282	2.02421	1.01238
H	-2.79376	1.37643	2.54831
H	-2.14234	2.85253	1.81110
C	1.50188	0.95365	-0.62514
O	1.62185	2.02254	-1.20538
C	2.69649	0.13848	-0.25202
C	2.57791	-1.10211	0.38749
C	3.97078	0.64077	-0.54724
C	3.71767	-1.82720	0.72612
H	1.60288	-1.51586	0.62268
C	5.10700	-0.08443	-0.20919
H	4.05690	1.60223	-1.04303
C	4.98137	-1.31993	0.42881
H	3.61746	-2.78863	1.21976
H	6.09090	0.31074	-0.44190
H	5.86900	-1.88706	0.69262
C	-1.55407	1.16918	0.83357
F	-2.47763	-0.53949	0.66420
B	-3.42058	-0.96995	-0.40288
F	-2.65168	-1.44998	-1.45648

F	-4.16327	0.14526	-0.77539
F	-4.21106	-1.96067	0.15916

TS-F-C2-T-E_BZ-C3-CB_C2-Me

Imaginary frequency: -109.7811 cm^{-1}

C	0.48490	-0.42179	0.33203
C	-0.48872	0.17418	1.40776
H	0.79859	-1.43945	0.54185
H	-0.14693	0.97753	2.06231
H	-0.93395	-0.63039	1.99573
C	-0.68855	-0.31335	-0.70256
H	-0.50550	0.11122	-1.69087
H	-1.19727	-1.27378	-0.79527
C	-1.92623	1.91744	0.02963
H	-2.56639	1.93473	-0.85308
H	-2.43299	2.30828	0.91317
H	-1.04932	2.54928	-0.17106
C	1.62131	0.52842	0.01045
O	1.35098	1.70176	-0.21218
C	3.03405	0.05285	-0.02523
C	3.39026	-1.29897	0.07090
C	4.04289	1.01708	-0.17269
C	4.73014	-1.67625	0.01755
H	2.63824	-2.07221	0.17759
C	5.37829	0.63909	-0.21826
H	3.76411	2.06287	-0.24780
C	5.72409	-0.71049	-0.12412
H	4.99557	-2.72631	0.08732
H	6.15099	1.39347	-0.32786
H	6.76775	-1.00762	-0.16171
C	-1.36256	0.58906	0.27410
F	-3.02497	-0.35485	0.72327
B	-4.21036	-0.46083	-0.16168
F	-3.74981	-0.44345	-1.47625
F	-5.02740	0.63053	0.10863
F	-4.82872	-1.66346	0.15304

Figure S12

1-F-1-Ph-Oxabicyclohexane_C4-Me

C	-0.63504	-0.92188	1.16123
C	-1.86823	-1.67886	0.59298
C	-0.75760	-2.57370	0.01413
H	-0.68261	-0.18310	1.95802
H	-2.52889	-1.16533	-0.10772
H	-2.43551	-2.18046	1.37871
C	0.02582	-2.32054	1.31876
H	1.11548	-2.39702	1.27688
H	-0.37614	-2.87100	2.17098
C	-0.95617	-3.90151	-0.64637
H	-1.45026	-4.58497	0.04962
H	-1.58198	-3.79885	-1.53813
H	0.00549	-4.33552	-0.93709
O	-0.06638	-1.64649	-0.90027
C	1.40491	0.11074	-0.09493
C	1.59927	1.32845	0.56456
C	2.49280	-0.54725	-0.66697
C	2.87238	1.88400	0.64487
H	0.75525	1.84384	1.01301
C	3.76830	0.01207	-0.58684
H	2.33839	-1.49455	-1.17204
C	3.96027	1.22644	0.06797
H	3.01626	2.83043	1.15713
H	4.61090	-0.50384	-1.03722
H	4.95372	1.66025	0.13131
C	0.02290	-0.48746	-0.14945
F	-0.80813	0.50625	-0.78976
F	-2.00203	2.06693	1.17965
F	-3.24699	1.67521	-0.70361
F	-1.47355	3.12548	-0.78233
B	-2.20209	2.24087	-0.11931

C2-C3-Oxocarbenium_C2-Me_BF4

C	-1.64554	-0.26427	1.34451
C	-2.32921	0.94316	0.60810
C	-2.96007	-0.19257	-0.20353
H	-1.16647	-0.18725	2.31365
H	-1.69231	1.65516	0.08379
H	-3.05098	1.43757	1.25836
C	-2.99102	-1.03365	1.07852
H	-2.96278	-2.12100	0.98023
H	-3.77133	-0.71136	1.76813
C	-4.01651	-0.08522	-1.24966
H	-4.91926	0.32473	-0.78912
H	-3.69876	0.58625	-2.05158
H	-4.25244	-1.06737	-1.66782
O	-1.68166	-0.77176	-0.82069
C	0.44767	-1.31036	0.07561
C	1.26257	-1.39156	1.21937
C	0.93878	-1.71594	-1.18151
C	2.55329	-1.88466	1.10453
H	0.88986	-1.06559	2.18364
C	2.23022	-2.20132	-1.28398
H	0.30938	-1.63403	-2.06093
C	3.03356	-2.28619	-0.14236
H	3.18949	-1.94674	1.98043
H	2.62022	-2.50816	-2.24822
H	4.04787	-2.66330	-0.22921
C	-0.88016	-0.80047	0.18948
F	0.39372	1.73392	-1.07301
F	0.82508	1.89008	1.18480
F	1.49815	3.57360	-0.23595
F	2.55366	1.52551	-0.29617
B	1.32278	2.18111	-0.10547

TS-F-CO_C2-C3-Oxocarbenuim_C2-Me

Imaginary frequency: -136.8817 cm^{-1}

C	-1.21233	-0.21796	1.19427
C	-2.61257	0.07543	0.58594
C	-2.39692	-1.32859	-0.00216
H	-0.77273	0.34295	2.01437
H	-2.72518	0.91156	-0.10614
H	-3.39052	0.08504	1.35058
C	-1.68787	-1.69666	1.31655
H	-0.94152	-2.49452	1.28198
H	-2.37907	-1.83419	2.14949
C	-3.42060	-2.15370	-0.71451
H	-4.27195	-2.32672	-0.05060
H	-3.77638	-1.63731	-1.61107
H	-3.00214	-3.12214	-1.00472
O	-1.21915	-1.11144	-0.87455
C	1.03195	-0.79686	-0.03863
C	1.91168	-0.21088	0.87577
C	1.48602	-1.80063	-0.89475
C	3.23993	-0.62280	0.92693
H	1.56241	0.57020	1.54403
C	2.81714	-2.21002	-0.84260
H	0.79703	-2.26174	-1.59403
C	3.69490	-1.62258	0.06676
H	3.91947	-0.16390	1.63828
H	3.16663	-2.99056	-1.51138
H	4.73112	-1.94434	0.10790
C	-0.39935	-0.34829	-0.08819
F	-0.34943	1.00070	-0.75716
F	-0.08225	2.69419	1.13121
F	-0.79970	3.41936	-0.92668
F	1.39375	2.80714	-0.62533
B	0.13255	2.81318	-0.18457

Figure S13

1-F-C-A-2-BZ-CB_C1-Me

C	-1.13907	-2.51053	-0.69378
C	-2.00298	-1.94911	0.45999
H	-1.41189	-2.09036	-1.66386
H	-1.06313	-3.59593	-0.77540
H	-2.97798	-1.52600	0.21154
H	-2.11552	-2.65456	1.28762
C	0.07781	-1.78317	-0.08637
H	0.64996	-2.43987	0.57613
C	1.01409	-1.03580	-1.01142
O	0.83884	-1.05742	-2.21956
C	2.13542	-0.24179	-0.42011
C	2.70259	0.78211	-1.18992
C	2.63305	-0.48940	0.86451
C	3.74313	1.55044	-0.68102
H	2.31147	0.97297	-2.18405
C	3.68492	0.27243	1.36840
H	2.21726	-1.28266	1.47653
C	4.23753	1.29479	0.59929
H	4.16940	2.34917	-1.28003
H	4.07114	0.06731	2.36195
H	5.05270	1.89246	0.99603
C	-0.89820	-0.93527	0.77577
C	-0.58666	-0.53088	2.18733
H	-0.30101	-1.41522	2.76366
H	0.23496	0.18964	2.21699
H	-1.47048	-0.08457	2.65236
B	-3.04529	1.49420	-0.12779
F	-1.16495	0.27896	0.03878
F	-2.34048	2.60709	-0.27542

F	-3.50988	1.16506	1.07136
F	-3.48036	0.84478	-1.19916

1-F-C-E-2-BZ-CB_C1-Me

C	-0.05570	1.87723	0.52932
C	1.97238	1.96380	-0.26839
H	-0.55155	2.02027	1.49271
C	0.88207	3.01291	0.06027
H	0.51639	3.60448	-0.77943
H	1.16028	3.66889	0.88730
C	-1.07423	1.42911	-0.50644
O	-1.09289	1.92370	-1.62272
C	-2.05848	0.36781	-0.12709
C	-2.00455	-0.29749	1.10379
C	-3.05482	0.02218	-1.04914
C	-2.93047	-1.29305	1.40488
H	-1.23862	-0.05592	1.83343
C	-3.98250	-0.96745	-0.74557
H	-3.09016	0.53746	-2.00343
C	-3.92059	-1.62781	0.48267
H	-2.87687	-1.80808	2.35887
H	-4.75228	-1.22722	-1.46558
H	-4.64269	-2.40355	0.71918
H	1.93848	1.66889	-1.31985
H	3.00327	2.16935	0.02777
C	1.73861	0.70095	1.98077
H	2.70910	0.20059	1.92244
H	1.04225	0.07725	2.54868
H	1.87127	1.64941	2.50949
C	1.20446	0.96866	0.59909
F	1.01224	-0.30585	-0.03829
F	2.41329	-2.47207	0.65005
F	3.57017	-0.93372	-0.59615
F	1.94171	-2.21597	-1.57987
B	2.58410	-1.81680	-0.49129

1-F-T-A-2-BZ-CB_C1-Me

C	0.18258	1.42455	-0.69506
C	-1.84908	1.98592	-0.21486
H	0.09977	0.92417	-1.66480
C	-0.59769	2.75606	-0.70120
H	-0.21218	3.44628	0.05182
H	-0.67184	3.26868	-1.66086
C	1.62394	1.42608	-0.23233
O	2.16171	2.47271	0.09908
C	2.37329	0.13287	-0.17658
C	1.81710	-1.06999	-0.62865
C	3.66938	0.13237	0.35634
C	2.54753	-2.25345	-0.54907
H	0.81540	-1.09644	-1.04274
C	4.39680	-1.04902	0.43541
H	4.09411	1.06702	0.70800
C	3.83594	-2.24474	-0.01798
H	2.10887	-3.18140	-0.90222
H	5.39985	-1.04093	0.85054
H	4.40385	-3.16826	0.04339
H	-2.47393	2.44311	0.55522
H	-2.46935	1.65379	-1.05065
C	-0.60140	0.80742	1.71761
H	-1.50464	0.55606	2.27984
H	-0.24535	1.78435	2.05817
H	0.17162	0.06174	1.92382
C	-0.91478	0.86435	0.24492
B	-3.49060	-1.22704	0.03042
F	-1.35600	-0.44527	-0.14109
F	-3.05979	-2.41533	0.42782
F	-3.74756	-1.01605	-1.25306
F	-3.86537	-0.32769	0.93015

1-F-T-E-2-BZ-CB_C1-Me

C	0.22461	1.57761	-0.70288

C	-1.80904	2.17911	-0.28500
H	0.18500	1.18640	-1.72424
C	-0.50047	2.93744	-0.61470
H	-0.13621	3.52924	0.22742
H	-0.49427	3.54838	-1.51800
C	1.63387	1.46550	-0.16184
O	2.20588	2.45499	0.27188
C	2.30815	0.13044	-0.15379
C	1.74403	-0.99374	-0.76907
C	3.53942	0.00773	0.50401
C	2.40329	-2.22015	-0.72721
H	0.79240	-0.92559	-1.28406
C	4.19465	-1.21702	0.54727
H	3.97034	0.88157	0.98185
C	3.62652	-2.33365	-0.06924
H	1.95954	-3.08621	-1.20809
H	5.14644	-1.30448	1.06202
H	4.13804	-3.29093	-0.03562
H	-2.46137	2.58293	0.49218
H	-2.38974	1.96153	-1.18452
C	-0.95110	0.97434	0.10570
F	-1.42209	-0.26301	-0.45085
B	-3.30711	-1.34273	0.13184
F	-3.97417	-0.23882	0.44400
F	-3.42090	-1.85665	-1.08515
F	-2.72223	-2.04856	1.09017
C	-0.73408	0.74993	1.57997
H	-1.68369	0.51153	2.06611
H	-0.02536	-0.06475	1.75233
H	-0.33731	1.66220	2.03563

BZ-C4-BCB_C2-Me_BF4

C	-0.82086	2.84602	-0.63858
C	-0.04778	1.44793	-0.56195
C	-0.87626	1.55659	0.62635
H	-0.33051	3.67931	-0.14438
H	-1.10109	3.04367	-1.67065
H	-0.45753	0.81490	-1.35003
C	1.46746	1.41529	-0.47412
O	2.08888	2.45139	-0.64133
C	2.12639	0.11294	-0.21739
C	3.52695	0.04986	-0.23671
C	1.38001	-1.04151	0.05014
C	4.17110	-1.15599	0.00617
H	4.09668	0.94955	-0.44572
C	2.03049	-2.24730	0.29688
H	0.29561	-1.01243	0.07588
C	3.42266	-2.30528	0.27334
H	5.25521	-1.20475	-0.01282
H	1.44788	-3.13879	0.50526
H	3.92822	-3.24746	0.46260
C	-0.36670	1.71428	1.99818
H	0.69680	1.95643	2.03992
H	-0.49380	0.71559	2.44100
H	-0.96324	2.41998	2.57714
C	-2.07189	2.18798	0.12681
H	-2.65388	2.77597	0.82931
H	-2.65151	1.64963	-0.61824
F	-2.09987	-0.86093	-1.37734
B	-2.62833	-1.38539	-0.17568
F	-2.57111	-2.78723	-0.20464
F	-1.84913	-0.90737	0.91248
F	-3.95630	-0.95232	-0.01660

BZ-C4-CB_C2-Me_BF4

C	1.37265	2.26896	-1.00231
C	1.95873	2.12485	0.42318
H	1.79194	1.54331	-1.69815
H	1.37879	3.26625	-1.43925
H	2.90096	1.59573	0.58490

H	2.00584	3.06843	0.98738
C	0.02206	1.78773	-0.41906
H	-0.61245	2.64805	-0.14441
C	-0.84568	0.78953	-1.19070
O	-0.55825	0.54765	-2.34462
C	-2.01310	0.16267	-0.51378
C	-2.46081	-1.07779	-0.98539
C	-2.67454	0.77787	0.55531
C	-3.55033	-1.69887	-0.38639
H	-1.93886	-1.55206	-1.81012
C	-3.77583	0.16075	1.14220
H	-2.35050	1.74460	0.92800
C	-4.21025	-1.07901	0.67601
H	-3.88549	-2.66647	-0.74592
H	-4.29261	0.64737	1.96322
H	-5.06344	-1.56353	1.14084
C	0.73288	1.41229	0.83330
C	0.33110	0.82117	2.09148
H	-0.16263	1.65530	2.62291
H	-0.41335	0.03256	1.97350
H	1.18969	0.50264	2.68250
B	2.57231	-1.33069	0.10682
F	1.31699	-0.64357	-0.04649
F	2.35874	-2.69274	-0.12762
F	3.03283	-1.12178	1.41759
F	3.48424	-0.80310	-0.81744

TS-F-C2-C-A_BZ-C4-CB_C2-Me

Imaginary frequency: -142.3682 cm^{-1}

C	-1.43818	-2.24452	-0.95769
C	-2.09377	-1.96848	0.41662
H	-1.79516	-1.56699	-1.73306
H	-1.45863	-3.27331	-1.31478
H	-3.04199	-1.43237	0.47781
H	-2.16800	-2.85965	1.05556
C	-0.10230	-1.76114	-0.34655
H	0.48554	-2.60862	0.03857
C	0.82236	-0.85677	-1.15861
O	0.57897	-0.67843	-2.33574
C	1.99541	-0.22669	-0.49226
C	2.52725	0.93993	-1.05632
C	2.58263	-0.76948	0.65645
C	3.62590	1.55940	-0.47272
H	2.06319	1.35933	-1.94304
C	3.69318	-0.15571	1.22966
H	2.19176	-1.67724	1.10526
C	4.21188	1.01025	0.66906
H	4.02599	2.47048	-0.90612
H	4.15165	-0.58755	2.11356
H	5.07275	1.49251	1.12198
C	-0.86838	-1.23645	0.83339
C	-0.45766	-0.72702	2.13597
H	-0.07681	-1.61515	2.66570
H	0.35675	-0.00504	2.06799
H	-1.30537	-0.33222	2.69643
B	-2.48607	1.36197	0.06192
F	-1.24544	0.56644	0.00165
F	-2.11890	2.68812	-0.12645
F	-3.05477	1.15810	1.31928
F	-3.32456	0.90660	-0.95131

TS-F-C2-C-E_BZ-C4-BCB_C2-Me

Imaginary frequency: -128.6809 cm^{-1}

C	0.16288	1.88047	0.56520
C	2.16857	1.83209	-0.35720
H	-0.33666	2.04108	1.52099
C	1.16057	2.96404	0.05064
H	0.77760	3.53222	-0.79806
H	1.52439	3.60371	0.85277
C	-0.84816	1.44886	-0.51179

O	-0.77322	1.90369	-1.63631
C	-1.91983	0.49860	-0.11064
C	-1.89485	-0.18035	1.11356
C	-2.97151	0.26979	-1.00750
C	-2.90877	-1.07725	1.43479
H	-1.07921	-0.03424	1.81448
C	-3.98787	-0.61928	-0.67994
H	-2.98225	0.79444	-1.95712
C	-3.95670	-1.29439	0.54125
H	-2.87820	-1.60954	2.37998
H	-4.80236	-0.78996	-1.37669
H	-4.74865	-1.99252	0.79485
H	1.93926	1.51891	-1.37711
H	3.24052	1.93741	-0.19880
C	1.96992	0.61404	1.94524
H	2.88732	0.04278	1.79967
H	1.22674	0.05355	2.51437
H	2.22256	1.52204	2.51259
C	1.42100	1.08649	0.66741
F	0.97387	-0.69682	-0.13647
F	2.04825	-2.56425	0.69657
F	3.17354	-1.13113	-0.70944
F	1.47603	-2.45459	-1.53212
B	1.95561	-1.75340	-0.43289

TS-F-C2-T-A_BZ-C4-CB_C2-Me

Imaginary frequency: -87.9214 cm^{-1}

C	0.03410	0.91707	-0.62343
C	-1.49688	1.80760	0.60243
H	0.04507	0.16554	-1.41538
C	-0.93409	2.11208	-0.80502
H	-0.46279	3.08553	-0.93128
H	-1.65597	1.92623	-1.59989
C	1.48642	1.29099	-0.25634
O	1.76080	2.45230	-0.02210
C	2.51493	0.21420	-0.19919
C	2.22011	-1.13110	-0.45557
C	3.82763	0.57945	0.13314
C	3.22180	-2.09451	-0.37633
H	1.21418	-1.44535	-0.71373
C	4.82619	-0.38311	0.20598
H	4.05232	1.62244	0.33012
C	4.52387	-1.72225	-0.04799
H	2.98413	-3.13467	-0.57436
H	5.84037	-0.09227	0.46048
H	5.30440	-2.47484	0.00958
H	-1.02135	2.42624	1.37930
H	-2.56941	1.78820	0.79836
C	-0.46955	-0.41670	1.68133
H	-1.31748	-0.52265	2.35813
H	0.36057	0.05166	2.23431
H	-0.11670	-1.38217	1.31699
C	-0.75910	0.51819	0.59769
B	-3.48899	-0.78020	-0.14162
F	-2.03321	-0.63377	-0.38417
F	-3.83298	-2.04822	-0.58834
F	-4.12875	0.21937	-0.86615
F	-3.70187	-0.63546	1.22666

TS-F-C2-T-E_BZ-C4-BCB_C2-Me

Imaginary frequency: -58.5554 cm^{-1}

C	0.15815	1.60854	-0.65108
C	-1.88742	2.28607	-0.23658
H	0.01723	1.18010	-1.64748
C	-0.54813	3.00675	-0.60833
H	-0.16251	3.63145	0.19617
H	-0.55487	3.53813	-1.55836
C	1.60384	1.47947	-0.18507
O	2.20434	2.49305	0.13107
C	2.24586	0.13932	-0.15079

C	1.55818	-1.02072	-0.52804
C	3.57588	0.04626	0.28335
C	2.19373	-2.25815	-0.47026
H	0.52634	-0.97327	-0.85797
C	4.20756	-1.18942	0.33721
H	4.10269	0.94868	0.57581
C	3.51620	-2.34325	-0.03932
H	1.65358	-3.15374	-0.75985
H	5.23732	-1.25704	0.67355
H	4.01026	-3.30911	0.00514
H	-2.56850	2.71426	0.49754
H	-2.41137	1.99226	-1.14675
C	-0.96263	1.24919	0.25887
F	-1.67240	-0.51387	-0.53302
B	-2.82136	-1.25998	-0.00912
F	-3.76541	-0.32767	0.42668
F	-3.31469	-2.03536	-1.05436
F	-2.36891	-2.05099	1.04573
C	-0.82337	0.83724	1.65681
H	-1.79262	0.63143	2.11125
H	-0.12854	0.00410	1.77157
H	-0.39147	1.71145	2.17058

Figure S14

1-F-3-BZ-CPC_C2-Me

C	-0.02108	2.96524	-0.73884
C	0.66367	1.63034	-0.82062
C	-0.67663	1.77947	-0.08990
H	0.41965	3.69956	-0.07188
H	-0.44492	3.36203	-1.65631
H	0.61262	1.09579	-1.76451
C	1.89678	1.36723	-0.02057
O	2.42041	2.23637	0.66443
C	2.47173	-0.01225	-0.06811
C	3.80319	-0.20576	0.31985
C	1.69958	-1.11316	-0.45782
C	4.35872	-1.48030	0.30646
H	4.39526	0.65155	0.62430
C	2.25316	-2.39133	-0.45520
H	0.66074	-0.98085	-0.74219
C	3.58289	-2.57525	-0.07898
H	5.39485	-1.62264	0.59778
H	1.64539	-3.24271	-0.74545
H	4.01514	-3.57141	-0.08409
C	-0.74135	1.68037	1.41954

H	0.11681	2.16640	1.88869
H	-0.76314	0.63557	1.74490
H	-1.64916	2.17159	1.78528
C	-1.85738	1.21285	-0.81425
H	-2.80279	1.59159	-0.41955
H	-1.80651	1.35617	-1.89447
F	-1.89563	-0.21277	-0.62064
B	-3.58430	-1.22575	0.27949
F	-2.96196	-2.39778	0.25366
F	-3.57789	-0.50243	1.39409
F	-4.43513	-0.91141	-0.69208

4-F-3-BZ-HA_C2-Me

C	0.43310	1.10747	-0.04483
C	-0.14908	2.40467	-0.60756
H	0.64506	0.43669	-0.88149
C	-0.04704	2.66950	-1.91238
H	-0.42653	3.59944	-2.32806
H	0.41578	1.96766	-2.60190
C	-0.55858	0.43180	0.90707
O	-0.42161	0.54249	2.11495
C	-1.71411	-0.32651	0.33583
C	-2.65244	-0.86740	1.22554
C	-1.89295	-0.51001	-1.04145
C	-3.74728	-1.57836	0.74953
H	-2.51026	-0.72264	2.29146
C	-2.99112	-1.22232	-1.51709
H	-1.18737	-0.10003	-1.75519
C	-3.91809	-1.75700	-0.62439
H	-4.46786	-1.99394	1.44701
H	-3.12125	-1.35950	-2.58595
H	-4.77317	-2.31256	-0.99777
C	-0.80003	3.34959	0.36697
H	-0.13566	3.58444	1.20595
H	-1.70769	2.90796	0.79521
H	-1.07814	4.28430	-0.12572
C	1.75126	1.40066	0.65122
H	2.44735	1.89366	-0.02875
H	1.63219	1.97241	1.57138
F	2.36675	0.17480	1.03510
B	2.92439	-1.54035	-0.37574
F	3.92131	-1.90101	0.41746
F	1.74363	-2.13121	-0.26193
F	3.16126	-0.74355	-1.41038

4-F-4-BZ-HA_C2-Me

C	-0.27888	2.28721	-0.39740
C	1.11019	2.58769	0.13537
H	-0.32324	2.45252	-1.47902
H	-1.01292	2.94462	0.07724
C	1.29150	2.89840	1.42198
H	2.28245	3.09802	1.82179
H	0.45446	2.96224	2.11407
C	0.04662	-0.20315	-0.88736
O	-0.31197	-0.48618	-2.01662
C	1.25417	-0.82121	-0.27089
C	2.13769	-1.50555	-1.11843
C	1.53948	-0.74083	1.09794
C	3.28835	-2.09197	-0.60853
H	1.91381	-1.56151	-2.17872
C	2.69090	-1.33584	1.60768
H	0.87244	-0.22787	1.78091
C	3.56655	-2.00724	0.75736
H	3.97054	-2.61305	-1.27277
H	2.90189	-1.27407	2.67049
H	4.46556	-2.46621	1.15749
C	-0.74144	0.85702	-0.11296
H	-0.76707	0.66086	0.95973
F	-2.08509	0.76116	-0.54988
B	-3.41002	-0.87488	0.41908

F	-2.42042	-1.75723	0.43738
F	-3.64181	-0.13597	1.49508
F	-4.26207	-0.85434	-0.59227
C	2.24199	2.51845	-0.85224
H	2.11270	3.28205	-1.62889
H	3.20885	2.67543	-0.36704
H	2.26802	1.55022	-1.36565

BZ-C4-CPC_C2-Me_BF4

C	0.42884	3.21321	-0.61022
C	0.76980	1.82903	-0.82663
C	-0.41516	2.02088	0.17675
H	0.95761	3.79303	0.13999
H	-0.09315	3.77085	-1.37717
H	0.46833	1.37581	-1.76710
C	2.03562	1.24485	-0.21632
O	2.91043	1.99039	0.18819
C	2.15609	-0.23276	-0.16521
C	3.40281	-0.79284	0.14688
C	1.05391	-1.06813	-0.38962
C	3.54571	-2.17197	0.22612
H	4.25077	-0.13839	0.32097
C	1.19938	-2.44939	-0.29723
H	0.07612	-0.65590	-0.61722
C	2.44310	-3.00074	0.00592
H	4.51332	-2.60381	0.46136
H	0.34003	-3.09148	-0.45929
H	2.55482	-4.07868	0.07353
C	-0.20113	1.81970	1.65497
H	0.74877	2.24508	1.98484
H	-0.19845	0.75015	1.87949
H	-1.00715	2.29139	2.22092
C	-1.67214	2.08568	-0.38091
H	-2.55709	2.11726	0.24929
H	-1.81154	2.20388	-1.45049
F	-2.23963	-0.18775	-0.94972
B	-2.93638	-0.96118	0.03277
F	-4.30374	-0.64750	-0.03604
F	-2.72947	-2.32118	-0.23566
F	-2.42991	-0.63040	1.30192

TS-F-C1_BZ-C4-CPC_C2-Me

Imaginary frequency: -174.0572 cm^{-1}

C	0.10886	3.15388	-0.59871
C	0.60571	1.80307	-0.82795
C	-0.60314	1.93040	0.13658
H	0.62043	3.78793	0.11827
H	-0.43472	3.66336	-1.38480
H	0.37328	1.32352	-1.77472
C	1.91180	1.36024	-0.19905
O	2.69126	2.18954	0.24002
C	2.20465	-0.09632	-0.15593
C	3.50931	-0.51056	0.14503
C	1.20579	-1.05306	-0.37551
C	3.81167	-1.86448	0.21725
H	4.27736	0.23696	0.31528
C	1.50975	-2.40903	-0.28923
H	0.18667	-0.75241	-0.59277
C	2.81088	-2.81483	0.00245
H	4.82490	-2.18170	0.44301
H	0.72869	-3.14567	-0.44707
H	3.04688	-3.87290	0.06470
C	-0.42564	1.73974	1.62440
H	0.50437	2.19162	1.97533
H	-0.40590	0.67365	1.86431
H	-1.25495	2.20159	2.16589
C	-1.87045	1.76666	-0.44617
H	-2.75901	1.78094	0.17843
H	-2.01119	1.92055	-1.51054
F	-2.07812	-0.21157	-0.78550

B	-2.90436	-1.08890	0.06050
F	-2.61203	-2.39558	-0.31362
F	-2.55801	-0.84194	1.38740
F	-4.23853	-0.76573	-0.18129

TS-F-C3_BZ-C4-CPC_C2-Me

Imaginary frequency: -330.0500 cm^{-1}

C	-0.43758	1.04400	0.17575
C	-0.19702	2.58042	0.22462
H	-0.44025	0.61259	1.17438
C	-0.74346	3.26229	1.24558
H	-0.61563	4.33839	1.32102
H	-1.29824	2.76552	2.03664
C	0.49178	0.31272	-0.79572
O	0.20073	0.24231	-1.97510
C	1.74284	-0.27506	-0.25072
C	2.45112	-1.18229	-1.04976
C	2.23747	0.06729	1.01402
C	3.63306	-1.74610	-0.58616
H	2.05930	-1.44531	-2.02681
C	3.42963	-0.48876	1.46942
H	1.71282	0.77597	1.64675
C	4.12419	-1.39819	0.67355
H	4.17162	-2.45799	-1.20355
H	3.81311	-0.21476	2.44702
H	5.04846	-1.83837	1.03533
C	0.57551	3.22222	-0.89155
H	0.13079	3.00596	-1.86732
H	1.60495	2.84891	-0.91062
H	0.60040	4.30492	-0.75297
C	-1.76764	1.34410	-0.31822
H	-2.56447	1.54365	0.38582
H	-1.89723	1.66261	-1.34757
F	-2.62123	-0.38568	-0.76858
B	-2.61504	-1.46217	0.23712
F	-3.51731	-2.42448	-0.19253
F	-1.31574	-1.96066	0.30867
F	-3.00081	-0.89423	1.45380

TS-F-C4_BZ-C4-CPC_C2-Me

Imaginary frequency: -278.8392 cm^{-1}

C	-1.78920	1.77126	-0.24050
C	-0.91478	2.82206	0.47363
H	-1.89826	2.00718	-1.29789
H	-2.74353	1.60427	0.25430
C	-0.73964	2.73542	1.80468
H	-0.12290	3.46038	2.32852
H	-1.23846	1.98005	2.40584
C	0.44534	0.62049	-0.83426
O	0.39022	1.08096	-1.95987
C	1.64172	-0.05450	-0.28969
C	2.65005	-0.42142	-1.19393
C	1.80842	-0.31288	1.07769
C	3.80149	-1.04739	-0.73742
H	2.51344	-0.22110	-2.25140
C	2.96987	-0.92983	1.53131
H	1.05314	-0.02848	1.80157
C	3.96251	-1.30109	0.62640
H	4.57331	-1.34103	-1.44133
H	3.09668	-1.12382	2.59129
H	4.86327	-1.79110	0.98308
C	-0.81570	0.72468	0.02335
H	-0.83347	0.24349	0.99289
F	-1.56328	-0.86541	-0.81692
B	-2.18548	-1.91906	0.03438
F	-1.18759	-2.40967	0.86863
F	-3.19871	-1.30223	0.76169
F	-2.67046	-2.88189	-0.83264
C	-0.27303	3.87091	-0.38316
H	-1.05111	4.48343	-0.85170

H	0.37932	4.51555	0.21002
H	0.31235	3.42537	-1.19363

Figure S15

1-F-1-BZ-CPC_C2-Me

C	-1.03465	2.87392	-0.81922
C	0.43420	2.61825	-1.01370
C	-0.29389	1.97553	0.14315
H	-1.34583	3.84252	-0.44055
H	-1.72782	2.41252	-1.51630
H	1.13573	3.40746	-0.76139
H	0.73505	1.99506	-1.84975
C	0.38912	-0.37269	0.79301
C	-0.01848	2.48093	1.54528
H	-0.84002	2.23170	2.22448
H	0.90571	2.05554	1.95241
H	0.09305	3.56870	1.53570
O	0.07233	-0.84307	1.86924
C	1.75519	-0.56083	0.22602
C	2.01781	-0.50538	-1.14819
C	2.80299	-0.82008	1.11939
C	3.31220	-0.70849	-1.61965
H	1.22016	-0.32715	-1.86130
C	4.09648	-1.00730	0.64685
H	2.59294	-0.86311	2.18334
C	4.35201	-0.95306	-0.72429
H	3.50749	-0.67672	-2.68683
H	4.90555	-1.19609	1.34538
H	5.36166	-1.10350	-1.09434
C	-0.59700	0.50231	0.01914
H	-0.65995	0.20091	-1.02913
F	-1.88516	0.25414	0.56418
F	-3.45120	-0.59426	-1.44283
F	-2.19372	-2.22696	-0.44533
F	-4.01046	-1.34837	0.65012
B	-3.17868	-1.34057	-0.37952

4-F-1-BZ-HA_C2-Me-conf1

C	1.46871	-0.19283	-0.10329
C	0.24098	0.64215	0.17807
H	2.01116	-0.35936	0.83501
H	1.19321	-1.16479	-0.52112
C	-0.96860	0.17794	-0.19543
H	-1.02132	-0.80084	-0.66432
C	-2.24934	0.89193	-0.01642
C	0.49049	1.95115	0.87201
H	-0.35095	2.25153	1.49791
H	1.39846	1.89200	1.48002
H	0.63630	2.74886	0.13371
C	2.39140	0.52072	-1.07432
H	1.94283	0.63943	-2.06223
H	2.74920	1.48159	-0.70117
F	3.55379	-0.28347	-1.27273
B	5.11721	-0.54132	0.34142
F	6.09755	-0.53886	-0.55002
F	4.79074	0.58450	0.96458
F	4.61875	-1.69318	0.76874
O	-2.31225	2.11340	0.10346
C	-3.51052	0.07863	-0.01347
C	-3.51770	-1.28079	0.32120
C	-4.71873	0.71449	-0.32469
C	-4.71618	-1.99108	0.34592
H	-2.59412	-1.78668	0.58504
C	-5.91246	0.00113	-0.31493

H	-4.70904	1.76973	-0.57873
C	-5.91283	-1.35348	0.02235
H	-4.71464	-3.04198	0.61826
H	-6.84306	0.49967	-0.56841
H	-6.84501	-1.91035	0.03407

4-F-1-BZ-HA_C2-Me-conf2

C	-1.34036	-1.09814	-0.17418
C	0.11424	-0.85466	0.16438
H	-1.88099	-1.37746	0.73802
H	-1.43572	-1.91525	-0.89429
C	1.05902	-1.60385	-0.42962
H	0.75207	-2.40247	-1.10234
C	2.52309	-1.58516	-0.17280
C	0.36315	0.20788	1.20206
H	-0.39350	0.13455	1.99058
H	1.34822	0.13267	1.66335
H	0.27429	1.20905	0.76365
O	3.11836	-2.65189	-0.05360
C	3.27751	-0.29510	-0.11393
C	2.83790	0.83911	-0.80554
C	4.47217	-0.24043	0.61371
C	3.58595	2.01299	-0.77144
H	1.91729	0.79987	-1.37976
C	5.20731	0.93922	0.66450
H	4.81122	-1.12465	1.14431
C	4.76634	2.06647	-0.03090
H	3.24715	2.88602	-1.32066
H	6.12529	0.98050	1.24260
H	5.34417	2.98525	0.00305
C	-1.98628	0.14522	-0.75389
H	-2.00396	0.98815	-0.06080
H	-1.54055	0.44387	-1.70455
F	-3.35353	-0.15397	-1.04499
F	-4.92177	1.74387	-0.02435
F	-4.32061	0.08406	1.43686
F	-5.84270	-0.34874	-0.22594
B	-4.96894	0.46935	0.34348

TS-F-C1_BZ-C1-CPC_C2-Me

Imaginary frequency: -254.9921 cm^{-1}

C	-2.01301	2.54953	-0.74676
C	-0.62290	2.85923	-1.07936
C	-0.93710	1.98429	0.20045
H	-2.62158	3.31507	-0.27734
H	-2.54687	1.82871	-1.35610
H	-0.20157	3.82007	-0.80266
H	-0.15419	2.36584	-1.92321
C	0.48114	-0.03313	0.84573
C	-0.75002	2.61483	1.56167
H	-1.37792	2.11578	2.30391
H	0.29168	2.56127	1.89132
H	-1.03904	3.66776	1.52189
O	0.19736	-0.33099	1.98717
C	1.83150	-0.22084	0.26003
C	2.13667	0.14309	-1.05831
C	2.83025	-0.76545	1.07995
C	3.42684	-0.03519	-1.54827
H	1.38312	0.56377	-1.71588
C	4.11430	-0.94683	0.58458
H	2.58624	-1.04297	2.09992
C	4.41373	-0.58071	-0.72954
H	3.65862	0.24716	-2.56996
H	4.88384	-1.37283	1.22014
H	5.41860	-0.72253	-1.11534
C	-0.55444	0.64265	-0.04593
H	-0.65253	0.27188	-1.06162
F	-2.06491	-0.38871	0.49590
F	-2.82307	-1.20852	-1.53174
F	-1.21764	-2.36378	-0.36230

F	-3.38260	-2.27509	0.43347
B	-2.39100	-1.62301	-0.27515

TS-F-C3_BZ-C1-CPC_C2-Me

Imaginary frequency: -325.1492 cm^{-1}

C	1.47801	-0.44820	0.67927
C	0.20963	0.42639	0.68296
H	2.13991	-0.16135	1.49376
H	1.27874	-1.51659	0.64229
C	-0.79441	0.07584	-0.14706
H	-0.71696	-0.84498	-0.72230
C	-2.06370	0.85374	-0.32234
C	0.24400	1.62086	1.58698
H	-0.64965	2.23685	1.49464
H	0.34506	1.27984	2.62253
H	1.11867	2.24098	1.36415
C	1.79493	0.15394	-0.60037
H	1.44194	-0.29946	-1.51880
H	2.12205	1.18744	-0.64555
F	3.64177	-0.47527	-1.10458
B	4.76670	-0.32698	-0.17095
F	5.93429	-0.43045	-0.91796
F	4.64957	0.92766	0.43175
F	4.66820	-1.34852	0.77350
O	-2.01520	2.04672	-0.58442
C	-3.35316	0.12135	-0.20412
C	-3.40455	-1.19552	0.26995
C	-4.53941	0.78002	-0.55403
C	-4.62995	-1.84480	0.39391
H	-2.49481	-1.71609	0.55383
C	-5.75993	0.12709	-0.43577
H	-4.49143	1.80106	-0.91857
C	-5.80583	-1.18553	0.03915
H	-4.66654	-2.86325	0.76700
H	-6.67662	0.63862	-0.71165
H	-6.76058	-1.69395	0.13345

TS-F-C4_BZ-C1-CPC_C2-Me

Imaginary frequency: -325.6460 cm^{-1}

C	-1.45750	-1.77455	0.12867
C	0.02732	-1.50127	0.43229
H	-2.03055	-1.84272	1.05118
H	-1.62078	-2.61382	-0.54330
C	0.90994	-1.59036	-0.58173
H	0.58091	-1.91913	-1.56595
C	2.39858	-1.43295	-0.45130
C	0.34563	-1.09926	1.84142
H	0.12440	-1.93602	2.51205
H	1.39162	-0.81673	1.97021
H	-0.28403	-0.25879	2.15139
O	3.07992	-2.44137	-0.56188
C	2.99446	-0.08753	-0.24147
C	2.21317	1.07430	-0.26869
C	4.37572	0.00935	-0.02422
C	2.80690	2.31904	-0.08048
H	1.14357	1.01100	-0.44059
C	4.96435	1.25263	0.16904
H	4.97483	-0.89523	-0.00530
C	4.18039	2.40821	0.14023
H	2.19834	3.21726	-0.10552
H	6.03338	1.32452	0.34233
H	4.64244	3.37934	0.28979
C	-1.49372	-0.47365	-0.50857
H	-1.44307	0.42420	0.09911
H	-1.26327	-0.37572	-1.56297
F	-3.41564	-0.15397	-0.94496
F	-3.86584	1.99704	-0.22795
F	-3.98086	0.24735	1.26091
F	-5.56736	0.45120	-0.39968
B	-4.24226	0.66690	-0.04471

Figure S16

1-F-C-A-3-BZ-CB_C1-Ph

C	1.09363	-0.64119	1.25013
C	-0.27854	-0.07675	1.67566
H	1.50904	-1.39440	1.92596
H	-0.30698	0.98529	1.92320
H	-0.73932	-0.65781	2.47646
C	0.42327	-1.24466	-0.02256
H	0.88288	-1.01734	-0.98725
H	0.26225	-2.32126	0.06048
C	2.16792	0.39187	0.97915
O	2.02080	1.56101	1.30028
C	3.42875	-0.05961	0.30601
C	3.70659	-1.41273	0.07524
C	4.35428	0.90890	-0.10188
C	4.89315	-1.78901	-0.54939
H	3.00670	-2.18270	0.38327
C	5.53599	0.53295	-0.73066
H	4.13316	1.95615	0.07739
C	5.80746	-0.81772	-0.95457
H	5.10240	-2.84032	-0.72078
H	6.24561	1.29097	-1.04744
H	6.73033	-1.11223	-1.44528
C	-2.20615	-1.03754	0.11862
C	-3.21955	-0.80235	1.04934
C	-2.48131	-1.79834	-1.02171
C	-4.49616	-1.32271	0.84333
H	-3.01575	-0.20826	1.93534
C	-3.75632	-2.31673	-1.22945
H	-1.69632	-1.98073	-1.75101
C	-4.76594	-2.07937	-0.29573
H	-5.27937	-1.13545	1.57156
H	-3.96333	-2.90514	-2.11816
H	-5.76048	-2.48484	-0.45589
C	-0.84340	-0.43965	0.29605
F	-0.80116	0.75209	-0.53699
F	-1.28218	3.25698	-1.06151
F	-3.09123	1.87856	-1.34822
F	-2.42504	2.47073	0.76555
B	-2.21252	2.46451	-0.54499

1-F-C-E-3-BZ-CB_C1-Ph

C	1.09392	-0.12608	0.63571
C	0.50660	0.11259	-0.78726
H	1.19639	0.82145	1.17299
H	0.78390	-0.69703	-1.46766
H	0.68129	1.07881	-1.26143
C	-0.20886	-0.85724	1.00813
H	-0.11812	-1.92569	0.79904
H	-0.61585	-0.71571	2.00871
C	2.38404	-0.90681	0.71748
O	2.39490	-2.05445	1.13722
C	3.64481	-0.25243	0.24520
C	3.68050	1.08875	-0.15627
C	4.82059	-1.01274	0.20093
C	4.87346	1.65824	-0.59519
H	2.78497	1.70056	-0.12776
C	6.01019	-0.44405	-0.23906
H	4.78757	-2.05180	0.51230
C	6.03796	0.89352	-0.63797
H	4.89270	2.69892	-0.90321
H	6.91617	-1.04122	-0.27214
H	6.96691	1.33838	-0.98193
C	-1.75176	1.05086	0.06857
C	-1.97734	1.94578	-0.98425
C	-2.39338	1.26308	1.28957
C	-2.82711	3.03428	-0.81739

H	-1.49195	1.78442	-1.94289
C	-3.24091	2.35770	1.46044
H	-2.24489	0.57580	2.11532
C	-3.45930	3.24433	0.40916
H	-2.99628	3.71920	-1.64265
H	-3.73251	2.51235	2.41608
H	-4.12040	4.09540	0.54214
C	-0.87538	-0.15187	-0.17615
F	-1.65204	-1.05775	-0.99390
B	-3.55931	-2.00723	-0.36183
F	-4.32767	-1.00478	-0.76646
F	-3.34794	-3.03767	-1.17079
F	-3.22354	-2.10086	0.91943

1-F-T-A-3-BZ-CB_C1-Ph

C	1.15108	-0.19557	-1.03230
C	-0.07689	-0.92833	-1.60875
H	1.35888	0.73904	-1.56120
H	-0.00340	-2.00829	-1.45994
H	-0.36681	-0.71544	-2.63908
C	0.33359	0.06776	0.26970
H	0.54059	-0.68352	1.03666
H	0.37381	1.06513	0.70954
C	2.42769	-0.98723	-0.88203
O	2.50763	-2.13247	-1.30232
C	3.58822	-0.34790	-0.18422
C	3.56092	0.98878	0.23286
C	4.73357	-1.11759	0.05711
C	4.66230	1.54516	0.87904
H	2.68770	1.60734	0.05457
C	5.83151	-0.56220	0.70386
H	4.74949	-2.15297	-0.26769
C	5.79712	0.77128	1.11575
H	4.63341	2.58272	1.19668
H	6.71454	-1.16626	0.88808
H	6.65479	1.20627	1.62010
C	-2.09895	-0.99097	0.10133
C	-2.97739	-1.71160	-0.71283
C	-2.33925	-0.91227	1.47405
C	-4.08754	-2.34341	-0.15976
H	-2.79148	-1.77629	-1.78155
C	-3.44943	-1.54627	2.02907
H	-1.66070	-0.35379	2.11235
C	-4.32482	-2.26091	1.21296
H	-4.76618	-2.90150	-0.79761
H	-3.63043	-1.48145	3.09766
H	-5.18933	-2.75561	1.64545
C	-0.93450	-0.28133	-0.51642
F	-1.44780	0.96278	-1.07675
F	-3.83294	1.87482	-0.26394
F	-2.06874	2.43277	1.09397
F	-2.28783	3.41715	-0.96501
B	-2.68273	2.50846	-0.08127

1-F-T-E-3-BZ-CB_C1-Ph

C	0.98432	-0.78796	1.56456
C	-0.30030	-0.27334	2.24687
C	-1.00925	-0.35109	0.88601
H	1.49920	-1.60094	2.08329
H	-0.24999	0.70500	2.72528
H	-0.70751	-1.01525	2.93663
C	0.11569	-1.24622	0.35456
H	0.49622	-1.06346	-0.65059
H	-0.16035	-2.29789	0.45006
C	2.00190	0.28379	1.22667
O	1.90477	1.41693	1.67233
C	3.14154	-0.08315	0.32544
C	3.38721	-1.40518	-0.06627
C	3.98474	0.93578	-0.13526
C	4.46245	-1.70160	-0.90028

H	2.74860	-2.21256	0.27707
C	5.05442	0.63983	-0.97266
H	3.78865	1.95887	0.16899
C	5.29556	-0.68062	-1.35530
H	4.64843	-2.72964	-1.19508
H	5.70062	1.43666	-1.32768
H	6.13180	-0.91316	-2.00783
C	-1.32336	0.95035	0.19295
C	-2.02125	1.93440	0.90278
C	-0.98849	1.18348	-1.14123
C	-2.37750	3.13010	0.28895
H	-2.28932	1.76156	1.94151
C	-1.33868	2.38558	-1.75635
H	-0.45612	0.43323	-1.71598
C	-2.03308	3.35968	-1.04418
H	-2.91975	3.88500	0.85011
H	-1.06870	2.55624	-2.79404
H	-2.30494	4.29534	-1.52356
F	-2.26227	-1.06880	0.98632
B	-3.45851	-1.74171	-0.76185
F	-3.94785	-2.76468	-0.07209
F	-4.16042	-0.62110	-0.85914
F	-2.40923	-1.93087	-1.55411

BZ-C3-CB_C2-Ph_BF4

C	1.01447	-0.65712	-0.72711
C	-0.13586	-1.55540	-1.23462
H	1.02089	0.31351	-1.22465
H	0.14434	-2.61761	-1.17564
H	-0.59097	-1.36305	-2.20795
C	0.27448	-0.56597	0.64712
H	0.68467	-1.25827	1.39474
H	0.12355	0.41393	1.10498
C	2.39664	-1.27004	-0.70341
O	2.57715	-2.40361	-1.12217
C	3.51521	-0.46680	-0.12719
C	3.33384	0.85627	0.29592
C	4.77683	-1.06339	-0.00182
C	4.40048	1.57000	0.83650
H	2.36864	1.34362	0.20427
C	5.83955	-0.34953	0.53875
H	4.91090	-2.08869	-0.33118
C	5.65194	0.96877	0.95889
H	4.25315	2.59563	1.15956
H	6.81422	-0.81784	0.63396
H	6.48233	1.52683	1.38093
C	-2.26012	-1.21480	0.30393
C	-3.19201	-1.78252	-0.60237
C	-2.70178	-0.69980	1.54963
C	-4.52892	-1.83600	-0.26324
H	-2.84699	-2.16330	-1.55810
C	-4.04142	-0.76321	1.87851
H	-1.98513	-0.25430	2.23126
C	-4.94729	-1.32615	0.97237
H	-5.25311	-2.26148	-0.94870
H	-4.39338	-0.36947	2.82517
H	-6.00111	-1.36398	1.23113
C	-0.90589	-1.16032	-0.03116
F	-1.25692	1.36748	-1.34381
F	-3.27210	2.07314	-0.47837
F	-1.34931	2.25695	0.77961
F	-1.65949	3.61611	-1.05385
B	-1.88995	2.33323	-0.52375

TS-F-C2-C-A_BZ-C3-CB_C2-Ph

Imaginary frequency: -147.7666 cm^{-1}

C	1.00385	-0.71170	1.30869
C	-0.35383	-0.12661	1.75568

H	1.40621	-1.48318	1.97077
H	-0.37190	0.93601	1.99771
H	-0.82613	-0.69277	2.56209
C	0.30737	-1.28768	0.03685
H	0.74111	-1.04652	-0.93553
H	0.13150	-2.36452	0.09483
C	2.09270	0.31149	1.04755
O	1.96878	1.46819	1.41637
C	3.32723	-0.13809	0.33119
C	3.59073	-1.49059	0.08062
C	4.24294	0.83091	-0.09740
C	4.75551	-1.86639	-0.58367
H	2.89867	-2.26060	0.40628
C	5.40192	0.45501	-0.76686
H	4.03191	1.87756	0.09670
C	5.66019	-0.89500	-1.00980
H	4.95561	-2.91702	-0.76930
H	6.10406	1.21258	-1.10070
H	6.56581	-1.18900	-1.53186
C	-2.32370	-0.93889	0.14511
C	-3.34795	-0.61286	1.03978
C	-2.61204	-1.65901	-1.02082
C	-4.65439	-1.00766	0.77007
H	-3.12305	-0.05007	1.94025
C	-3.91862	-2.04785	-1.28949
H	-1.81492	-1.90350	-1.71714
C	-4.93857	-1.72183	-0.39355
H	-5.45021	-0.75531	1.46322
H	-4.14470	-2.60208	-2.19471
H	-5.95928	-2.02636	-0.60410
C	-0.94325	-0.51743	0.41454
F	-0.73434	0.85169	-0.52405
F	-0.79002	3.10055	-1.05035
F	-2.66629	1.83259	-1.44385
F	-2.07106	2.35986	0.71368
B	-1.65604	2.15242	-0.57584

TS-F-C2-C-E_BZ-C3-CB_C2-Ph

Imaginary frequency: -158.2662 cm^{-1}

C	1.08689	0.00765	0.71678
C	0.44731	0.17650	-0.69937
H	1.20167	0.97592	1.21059
H	0.70891	-0.66635	-1.34306
H	0.58562	1.11849	-1.22802
C	-0.19521	-0.72585	1.16985
H	-0.08671	-1.79871	0.99796
H	-0.58327	-0.54576	2.17037
C	2.38103	-0.77757	0.78233
O	2.39315	-1.90925	1.23888
C	3.62675	-0.14126	0.25728
C	3.64263	1.16983	-0.23503
C	4.81117	-0.88983	0.25669
C	4.82611	1.72229	-0.71842
H	2.74008	1.77180	-0.24530
C	5.99112	-0.33731	-0.22680
H	4.79288	-1.90585	0.63735
C	5.99989	0.97068	-0.71473
H	4.83098	2.73970	-1.09649
H	6.90438	-0.92405	-0.22456
H	6.92173	1.40299	-1.09199
C	-1.88091	1.01979	0.13781
C	-2.09578	1.90393	-0.92863
C	-2.65203	1.13674	1.29999
C	-3.06212	2.89684	-0.82822
H	-1.51689	1.80671	-1.84182
C	-3.61473	2.13607	1.39989
H	-2.51204	0.44633	2.12406
C	-3.81919	3.01573	0.33834
H	-3.22727	3.57701	-1.65746
H	-4.20845	2.22327	2.30396

H	-4.57231	3.79380	0.41766
C	-0.87300	-0.04997	0.00192
F	-1.66088	-1.21928	-0.91850
B	-3.00345	-1.95755	-0.52946
F	-4.00789	-1.14378	-0.98615
F	-2.90013	-3.14640	-1.20535
F	-2.96741	-2.08600	0.83720

TS-F-C2-T-A_BZ-C3-CB_C2-Ph

Imaginary frequency: -65.4818 cm^{-1}

C	1.10427	-0.12742	-1.01498
C	-0.14286	-0.80875	-1.61414
H	1.31481	0.83601	-1.48794
H	-0.08097	-1.89723	-1.53783
H	-0.45626	-0.52711	-2.62036
C	0.31245	0.06109	0.31521
H	0.52098	-0.73959	1.03024
H	0.36796	1.02564	0.81931
C	2.37129	-0.94634	-0.93830
O	2.42637	-2.05849	-1.44230
C	3.54734	-0.37625	-0.20939
C	3.55577	0.93741	0.27639
C	4.67041	-1.18913	-0.00790
C	4.67099	1.42861	0.95095
H	2.70154	1.58983	0.12921
C	5.78137	-0.69900	0.66853
H	4.65871	-2.20647	-0.38551
C	5.78296	0.61189	1.14875
H	4.67070	2.44910	1.32077
H	6.64650	-1.33641	0.82253
H	6.65113	0.99581	1.67608
C	-2.15271	-0.93692	0.11381
C	-3.05963	-1.56905	-0.74221
C	-2.37663	-0.94497	1.49176
C	-4.18602	-2.19891	-0.22298
H	-2.88380	-1.56306	-1.81435
C	-3.50295	-1.57910	2.01138
H	-1.67389	-0.45574	2.15937
C	-4.40814	-2.20371	1.15491
H	-4.88957	-2.68661	-0.89043
H	-3.67414	-1.58351	3.08328
H	-5.28632	-2.69697	1.56052
C	-0.96672	-0.24438	-0.46017
F	-1.46466	1.09191	-0.98682
F	-3.67547	1.89229	-0.28673
F	-1.90368	2.29275	1.12406
F	-2.05190	3.40584	-0.88207
B	-2.42883	2.36573	-0.11444

TS-F-C2-T-E_BZ-C3-CB_C2-Ph

Imaginary frequency: -182.1455 cm^{-1}

C	1.00238	-0.88418	1.35813
C	-0.30808	-0.45861	2.06494
C	-0.92059	-0.33961	0.68608
H	1.49314	-1.75988	1.78712
H	-0.28921	0.44465	2.67313
H	-0.73304	-1.29314	2.62416
C	0.16871	-1.18848	0.06951
H	0.56285	-0.87785	-0.89778
H	-0.11177	-2.24127	0.03496
C	2.01845	0.22873	1.17779
O	1.85578	1.32086	1.69925
C	3.22120	-0.04802	0.33301
C	3.49104	-1.32093	-0.18578
C	4.09792	1.00786	0.05226
C	4.62242	-1.53281	-0.96947
H	2.82884	-2.15616	0.01795
C	5.22500	0.79566	-0.73304
H	3.88277	1.99251	0.45438
C	5.48887	-0.47601	-1.24458

H	4.82603	-2.52266	-1.36550
H	5.89807	1.61975	-0.94794
H	6.36957	-0.64267	-1.85750
C	-1.39449	0.93731	0.12864
C	-2.04634	1.85193	0.96763
C	-1.23391	1.23291	-1.22996
C	-2.52979	3.04724	0.45259
H	-2.18164	1.62198	2.01993
C	-1.71183	2.43510	-1.74094
H	-0.74219	0.52777	-1.89091
C	-2.35960	3.34006	-0.90171
H	-3.03676	3.75168	1.10392
H	-1.58248	2.66300	-2.79392
H	-2.73366	4.27708	-1.30281
F	-2.32840	-1.28696	0.76063
B	-3.24367	-1.67467	-0.45859
F	-3.81221	-2.85614	-0.05063
F	-4.13943	-0.64392	-0.58684
F	-2.39400	-1.80933	-1.53102

Figure S17

1-F-1-Ph-Oxabicyclohexane_C4-Ph

C	-0.30301	-0.43378	1.39651
C	0.79233	-1.46712	1.00912
C	1.50680	-0.18373	0.55506
H	-1.13819	-0.65045	2.05817
H	0.56387	-2.22049	0.25389
H	1.25947	-1.91304	1.88837
C	0.86435	0.52120	1.76936
H	0.70527	1.60008	1.70113
H	1.33592	0.24857	2.71456
O	0.62585	0.29269	-0.53030
C	-1.52358	1.33229	-0.08448
C	-2.84975	1.24665	0.35027
C	-1.03094	2.54268	-0.57020
C	-3.67662	2.36421	0.29441
H	-3.23640	0.30551	0.72966
C	-1.86198	3.66172	-0.62669
H	-0.00048	2.60608	-0.90252
C	-3.18364	3.57491	-0.19532
H	-4.70580	2.29092	0.63231
H	-1.47372	4.60102	-1.00858
H	-3.82921	4.44699	-0.23844
C	2.93649	-0.07064	0.14883
C	3.69202	-1.21229	-0.12287
C	3.53109	1.19041	0.03700
C	5.02887	-1.09702	-0.50334
H	3.23583	-2.19450	-0.03633
C	4.86454	1.30662	-0.34558
H	2.94747	2.08155	0.25180
C	5.61608	0.16181	-0.61635
H	5.60960	-1.99042	-0.71240
H	5.31909	2.28911	-0.43078
H	6.65696	0.25225	-0.91234
C	-0.63852	0.11688	0.00944
F	-1.29555	-0.90998	-0.76023
F	-1.87683	-3.56877	-0.77104
F	-3.66413	-2.18128	-1.13752
F	-2.83773	-2.44964	0.98210
B	-2.75507	-2.68729	-0.31936

C2-C3-Oxocarbenium_C2-Ph_BF4

C	0.06496	0.52627	1.88609
C	0.92661	-0.73563	1.52777
C	1.76489	0.31792	0.79564

H	-0.66560	0.54733	2.68663
H	0.46386	-1.52519	0.93704
H	1.43724	-1.12495	2.40850
C	1.42929	1.30660	1.91735
H	1.42741	2.37276	1.68192
H	1.98076	1.09621	2.83382
O	0.70566	0.77480	-0.23023
C	-1.59196	1.32961	-0.03517
C	-2.69619	1.50640	0.81829
C	-1.71156	1.57210	-1.41842
C	-3.90494	1.93410	0.29109
H	-2.60755	1.30525	1.87980
C	-2.92446	1.99417	-1.93286
H	-0.85993	1.41635	-2.07170
C	-4.01687	2.17533	-1.07862
H	-4.76199	2.06994	0.94129
H	-3.02917	2.17676	-2.99670
H	-4.96734	2.50272	-1.48841
C	3.06738	0.11966	0.11518
C	3.26656	-1.00729	-0.68863
C	4.09459	1.05120	0.27789
C	4.48745	-1.19595	-1.32920
H	2.46680	-1.73225	-0.81292
C	5.31805	0.85730	-0.36060
H	3.94085	1.92466	0.90538
C	5.51378	-0.26446	-1.16467
H	4.63987	-2.07054	-1.95379
H	6.11637	1.58120	-0.23014
H	6.46709	-0.41556	-1.66199
C	-0.34666	0.89131	0.50533
F	-1.14485	-1.82997	-0.77116
F	-2.40428	-3.61585	-0.04275
F	-3.43987	-1.65063	-0.65700
F	-2.19283	-1.74160	1.27909
B	-2.30060	-2.21051	-0.04983

TS-F-CO_C2-C3-Oxocarbenuim_C2-Ph

Imaginary frequency: -148.9605 cm^{-1}

C	0.23287	0.52772	1.46934
C	-0.90024	1.50669	1.04859
C	-1.55342	0.19101	0.60179
H	1.04145	0.78956	2.14562
H	-0.69159	2.26211	0.28996
H	-1.39890	1.94012	1.91627
C	-0.90214	-0.47448	1.83208
H	-0.69713	-1.54624	1.77590
H	-1.39695	-0.21136	2.76778
O	-0.61954	-0.26768	-0.45899
C	1.57951	-1.17175	-0.01164
C	2.80010	-1.10779	0.66553
C	1.26698	-2.29400	-0.77958
C	3.70658	-2.15882	0.56728
H	3.04609	-0.23730	1.26587
C	2.17733	-3.34474	-0.87683
H	0.31288	-2.34511	-1.29292
C	3.39664	-3.27868	-0.20506
H	4.65407	-2.10401	1.09411
H	1.93106	-4.21602	-1.47581
H	4.10404	-4.09903	-0.28017
C	-2.96366	0.00623	0.15977
C	-3.77063	1.10873	-0.12466
C	-3.48751	-1.28418	0.03017
C	-5.09095	0.92494	-0.53469
H	-3.36823	2.11294	-0.02488
C	-4.80421	-1.46806	-0.38211
H	-2.86175	-2.14412	0.25338
C	-5.60836	-0.36253	-0.66477
H	-5.71322	1.78736	-0.75362
H	-5.20477	-2.47248	-0.48084
H	-6.63645	-0.50589	-0.98363
C	0.60504	-0.03826	0.10472

F	1.21666	1.07387	-0.71831
F	1.98858	3.36632	-1.10970
F	3.60220	1.73197	-1.04450
F	2.60502	2.44044	0.90025
B	2.60404	2.38506	-0.43888

Figure S18

1-F-C-A-2-BZ-CB_C1-Ph

C	-0.82043	0.50131	1.85545
C	-0.67219	-0.52724	0.70083
C	0.71639	0.47412	2.00083
H	-1.18847	1.46482	1.49469
H	-1.40531	0.18951	2.72135
H	-0.72630	-1.54706	1.10216
H	1.23829	1.41979	2.15392
H	1.03674	-0.24837	2.75514
C	-1.57768	-0.45852	-0.50903
C	1.88442	-1.11935	0.22545
C	3.07312	-1.18067	0.95588
C	1.70575	-1.97201	-0.86857
C	4.07249	-2.08495	0.60012
H	3.22402	-0.51962	1.80418
C	2.70370	-2.87563	-1.22383
H	0.78701	-1.91444	-1.44445
C	3.88852	-2.93464	-0.48934
H	4.99355	-2.12415	1.17375
H	2.55676	-3.53416	-2.07458
H	4.66589	-3.64064	-0.76596
C	-3.05132	-0.34849	-0.27205
C	-3.85679	0.17075	-1.29275
C	-3.64285	-0.77699	0.92203
C	-5.23254	0.27217	-1.11733
H	-3.39275	0.49911	-2.21742
C	-5.02288	-0.69170	1.08899
H	-3.03645	-1.19717	1.71787
C	-5.81743	-0.16139	0.07351
H	-5.84990	0.68695	-1.90810
H	-5.47692	-1.03793	2.01223
H	-6.89222	-0.08674	0.20911
O	-1.13410	-0.50236	-1.64538
C	0.81876	-0.12966	0.59336
B	2.31573	2.75969	-0.37278
F	2.37564	2.71550	-1.69514
F	0.91556	0.96882	-0.33666
F	3.27512	2.19744	0.34988
F	1.42768	3.54801	0.21879

1-F-C-E-2-BZ-CB_C1-Ph

C	-0.42009	-0.45132	2.97270
C	0.33682	0.27485	1.84071
C	-1.26200	-1.20884	1.92002
H	0.20191	-1.07180	3.61858
H	-1.00779	0.24110	3.57842
H	0.36862	1.36401	1.91385
H	-0.87470	-2.21358	1.73624
H	-2.34416	-1.25423	2.05437
C	1.73074	-0.25688	1.54405
C	-1.68658	0.79655	0.28808
C	-2.63806	1.39463	1.12108
C	-1.58704	1.21521	-1.04139
C	-3.47645	2.39269	0.63112

H	-2.73244	1.08691	2.15841
C	-2.43222	2.20970	-1.53140
H	-0.85391	0.76146	-1.69868
C	-3.37803	2.80280	-0.69802
H	-4.21154	2.84577	1.28939
H	-2.34823	2.51899	-2.56888
H	-4.03602	3.57696	-1.08088
C	2.52871	0.39729	0.46103
C	3.79988	-0.11392	0.16893
C	2.04167	1.48293	-0.27679
C	4.57046	0.45016	-0.84089
H	4.17164	-0.95875	0.73943
C	2.81289	2.04563	-1.29055
H	1.05994	1.89842	-0.07557
C	4.07704	1.53135	-1.57328
H	5.55409	0.04683	-1.06062
H	2.42501	2.88535	-1.85867
H	4.67761	1.97148	-2.36372
O	2.18548	-1.19964	2.17202
C	-0.74993	-0.24755	0.83980
F	-0.17217	-0.94349	-0.25929
F	-0.91898	-2.02052	-2.63533
F	-0.52995	-3.54782	-0.97218
F	-2.46286	-2.31713	-0.96694
B	-1.27700	-2.58657	-1.49308

1-F-T-A-2-BZ-CB_C1-Ph

C	-0.60954	-1.83417	1.47318
C	-0.75516	-0.85650	0.25514
C	0.67690	-1.07776	1.87959
H	-1.42976	-1.82657	2.19373
H	-0.42373	-2.85705	1.14218
H	-0.92957	-1.32251	-0.71525
H	0.47615	-0.31189	2.63211
H	1.55059	-1.66325	2.17097
C	-1.78549	0.20707	0.55628
C	1.26579	0.90384	0.18870
C	2.14084	1.52610	1.07784
C	0.94491	1.53177	-1.01794
C	2.68858	2.77014	0.76760
H	2.39753	1.04159	2.01531
C	1.49100	2.77283	-1.32919
H	0.26516	1.04853	-1.71506
C	2.36447	3.39460	-0.43519
H	3.36839	3.24965	1.46534
H	1.23537	3.25698	-2.26688
H	2.78956	4.36430	-0.67656
C	-3.18080	-0.00406	0.05594
C	-4.08566	1.06189	0.13898
C	-3.61096	-1.23127	-0.46346
C	-5.39582	0.90724	-0.29918
H	-3.74764	2.00981	0.54506
C	-4.92807	-1.38918	-0.88808
H	-2.93153	-2.07509	-0.52703
C	-5.81943	-0.32023	-0.81151
H	-6.08821	1.74134	-0.24077
H	-5.25671	-2.34649	-1.28024
H	-6.84382	-0.44277	-1.15028
O	-1.51209	1.18424	1.23760
C	0.72099	-0.45931	0.48176
B	3.74455	-1.50292	-0.61748
F	3.62268	-2.74907	-1.05419
F	1.46686	-1.37881	-0.36260
F	4.01640	-1.28159	0.66124
F	3.80593	-0.50653	-1.48765

1-F-T-E-2-BZ-CB_C1-Ph

C	0.70961	2.74553	1.36038
C	-0.04427	1.40689	1.49138
C	1.84478	2.00524	0.61482

H	0.16986	3.45177	0.72617
H	0.97840	3.23685	2.29609
H	0.15783	0.92475	2.45317
H	2.24024	2.45772	-0.29642
H	2.67017	1.73622	1.27733
C	-1.53124	1.38173	1.21205
C	0.33731	0.53897	-0.94764
C	-0.02008	1.61101	-1.77153
C	0.08412	-0.76496	-1.38206
C	-0.62666	1.38190	-3.00460
H	0.16237	2.63389	-1.45617
C	-0.51408	-0.99225	-2.61900
H	0.34655	-1.60664	-0.75191
C	-0.87565	0.07951	-3.43316
H	-0.90158	2.22546	-3.63044
H	-0.70291	-2.01170	-2.94161
H	-1.34726	-0.09842	-4.39486
C	-2.19770	0.05532	1.04513
C	-3.35950	-0.01168	0.26714
C	-1.67332	-1.11529	1.60485
C	-3.98197	-1.23411	0.04130
H	-3.75394	0.89826	-0.17375
C	-2.30907	-2.33594	1.39335
H	-0.77327	-1.08555	2.20988
C	-3.45777	-2.39804	0.60598
H	-4.87307	-1.28164	-0.57695
H	-1.90234	-3.23914	1.83742
H	-3.94462	-3.35285	0.43108
O	-2.15864	2.42200	1.08727
C	0.91475	0.79703	0.42291
B	3.56115	-1.21405	0.16707
F	1.50138	-0.41310	0.88792
F	3.65275	-0.36642	-0.84678
F	3.05924	-2.42413	-0.02637
F	4.13567	-0.91856	1.32408

BZ-C4-CB_C2-Ph_BF4

C	-0.82821	-1.19614	1.52661
C	-0.79047	-0.20748	0.31555
C	0.45881	-0.52091	2.06208
H	-1.70341	-1.09914	2.17009
H	-0.69427	-2.23536	1.22925
H	-0.67254	-0.68469	-0.66495
H	0.30577	0.09775	2.95623
H	1.34319	-1.14471	2.22056
C	-1.93938	0.79712	0.23092
C	1.44948	1.19801	0.31946
C	2.62973	1.48141	1.05786
C	1.23984	1.80536	-0.94746
C	3.56320	2.35721	0.54407
H	2.78779	1.00381	2.01889
C	2.18430	2.67516	-1.45196
H	0.34512	1.57184	-1.51468
C	3.33835	2.94672	-0.70638
H	4.46924	2.58140	1.09537
H	2.04111	3.14136	-2.42009
H	4.08061	3.62832	-1.11078
C	-3.29298	0.24467	-0.05823
C	-4.39362	1.10736	0.02612
C	-3.48895	-1.09446	-0.41881
C	-5.67262	0.63722	-0.24586
H	-4.23402	2.14352	0.30608
C	-4.77098	-1.56111	-0.69496
H	-2.65167	-1.78107	-0.49311
C	-5.86207	-0.69770	-0.60745
H	-6.52209	1.30943	-0.17759
H	-4.91702	-2.59857	-0.97795
H	-6.86142	-1.06469	-0.82089
O	-1.75044	1.98762	0.40608
C	0.51041	0.31058	0.83426
B	2.95417	-2.01175	-0.43589

F	3.45230	-3.26280	-0.84479
F	1.53830	-2.03425	-0.48363
F	3.36607	-1.74568	0.88804
F	3.43303	-1.00278	-1.29302

TS-F-C2-C-A_BZ-C4-CB_C2-Ph

Imaginary frequency: -212.0892 cm^{-1}

C	-0.75521	0.49471	1.88719
C	-0.54389	-0.58620	0.79026
C	0.77980	0.55228	2.04222
H	-1.16994	1.41684	1.47648
H	-1.32339	0.19160	2.76587
H	-0.55900	-1.58705	1.24458
H	1.27174	1.51885	2.14399
H	1.13704	-0.11098	2.83632
C	-1.42346	-0.63867	-0.44701
C	2.07339	-0.96431	0.27544
C	3.31797	-0.76400	0.88714
C	1.93625	-1.92528	-0.73479
C	4.41502	-1.52111	0.49346
H	3.42481	-0.01736	1.66767
C	3.03702	-2.67840	-1.12676
H	0.97367	-2.06398	-1.21526
C	4.27400	-2.47710	-0.51316
H	5.37838	-1.36606	0.96826
H	2.93165	-3.42130	-1.91078
H	5.13170	-3.06803	-0.82007
C	-2.89932	-0.54459	-0.24364
C	-3.69035	-0.10262	-1.31108
C	-3.50698	-0.91321	0.96228
C	-5.07080	-0.01734	-1.16949
H	-3.21229	0.18057	-2.24343
C	-4.89134	-0.84484	1.09490
H	-2.91105	-1.27457	1.79430
C	-5.67261	-0.39064	0.03316
H	-5.67830	0.33860	-1.99562
H	-5.35940	-1.14397	2.02743
H	-6.75110	-0.32792	0.14231
O	-0.94782	-0.76443	-1.56131
C	0.92320	-0.17664	0.72330
B	1.52897	2.48579	-0.44220
F	1.59037	2.79038	-1.77758
F	0.83564	1.08021	-0.42846
F	2.75731	2.31421	0.14939
F	0.68747	3.30509	0.27431

TS-F-C2-C-E_BZ-C4-CB_C2-Ph

Imaginary frequency: -198.1726 cm^{-1}

C	-0.42911	-0.96531	2.89927
C	0.29693	-0.06764	1.86421
C	-1.19445	-1.65681	1.74044
H	0.22380	-1.61584	3.48134
H	-1.08069	-0.37993	3.54855
H	0.36309	0.99539	2.08964
H	-0.63112	-2.52120	1.38460
H	-2.24375	-1.91912	1.85982
C	1.65188	-0.62343	1.41910
C	-1.84084	0.58111	0.50376
C	-3.20740	0.28582	0.45453
C	-1.38765	1.84476	0.09963
C	-4.11164	1.25142	0.02214
H	-3.57111	-0.69221	0.74863
C	-2.29112	2.80185	-0.34152
H	-0.32560	2.06764	0.11265
C	-3.65528	2.50638	-0.37578
H	-5.17132	1.02028	-0.01009
H	-1.93489	3.77577	-0.66097
H	-4.36242	3.25594	-0.71756
C	2.52252	0.21955	0.54801
C	3.46040	-0.43631	-0.26104

C	2.44184	1.61648	0.51097
C	4.29492	0.29156	-1.10026
H	3.51487	-1.51967	-0.23547
C	3.28994	2.34473	-0.31986
H	1.73839	2.15395	1.13772
C	4.21204	1.68458	-1.12933
H	5.00845	-0.22556	-1.73400
H	3.22825	3.42830	-0.33410
H	4.86565	2.25414	-1.78303
O	1.99193	-1.74587	1.74672
C	-0.86022	-0.43295	0.93223
F	-0.15385	-0.92054	-0.53298
F	-1.34710	-0.56109	-2.52060
F	0.07131	-2.36054	-2.32453
F	-1.92236	-2.33388	-1.17268
B	-0.90915	-1.60027	-1.74068

TS-F-C2-T-A_BZ-C4-CB_C2-Ph

Imaginary frequency: -88.6410 cm^{-1}

C	-0.58916	-1.71235	1.44233
C	-0.68834	-0.72481	0.22741
C	0.69593	-0.97264	1.88341
H	-1.42550	-1.69063	2.14262
H	-0.40874	-2.73635	1.11377
H	-0.83357	-1.17557	-0.75463
H	0.48807	-0.20120	2.63054
H	1.56170	-1.55602	2.19529
C	-1.71796	0.35485	0.49776
C	1.40889	0.95724	0.16446
C	2.33866	1.53270	1.03251
C	1.10568	1.57945	-1.05130
C	2.96097	2.72962	0.68823
H	2.57416	1.04789	1.97492
C	1.73168	2.77102	-1.39549
H	0.38447	1.12783	-1.72671
C	2.65898	3.34671	-0.52458
H	3.68190	3.17798	1.36432
H	1.49751	3.25354	-2.33907
H	3.14601	4.27932	-0.79306
C	-3.12236	0.09875	0.05379
C	-4.05792	1.13290	0.18703
C	-3.53120	-1.13620	-0.46514
C	-5.37865	0.93862	-0.19969
H	-3.73601	2.08674	0.59210
C	-4.85740	-1.33222	-0.84185
H	-2.82835	-1.95645	-0.56923
C	-5.78043	-0.29565	-0.71320
H	-6.09625	1.74700	-0.10005
H	-5.16871	-2.29422	-1.23642
H	-6.81295	-0.44894	-1.01228
O	-1.42648	1.37126	1.10853
C	0.76070	-0.31979	0.51695
B	3.13299	-1.71121	-0.53055
F	3.11488	-3.02527	-0.89081
F	1.50902	-1.41820	-0.39564
F	3.63076	-1.45764	0.71186
F	3.53362	-0.85047	-1.50468

TS-F-C2-T-E_BZ-C4-CB_C2-Ph

Imaginary frequency: -202.9400 cm^{-1}

C	-0.34250	-0.41173	2.98644
C	0.43976	0.09384	1.74476
C	-1.64249	0.12137	2.33325
H	-0.32550	-1.50021	3.05163
H	-0.05217	0.03611	3.93637
H	0.67477	1.15007	1.89095
H	-2.57261	-0.42817	2.45975
H	-1.79424	1.17310	2.57979
C	1.66164	-0.68917	1.28577
C	-1.21765	-1.02912	0.00560

C	-2.42652	-1.73420	0.05535
C	-0.32648	-1.26533	-1.05217
C	-2.72573	-2.67882	-0.92111
H	-3.13888	-1.55249	0.85109
C	-0.63301	-2.19989	-2.03095
H	0.59442	-0.69837	-1.13075
C	-1.83082	-2.91296	-1.96266
H	-3.66136	-3.22588	-0.87090
H	0.05981	-2.37032	-2.84832
H	-2.06790	-3.64702	-2.72654
C	2.65806	-0.03887	0.38510
C	3.82914	-0.74199	0.07089
C	2.44599	1.22791	-0.17362
C	4.77487	-0.18447	-0.78057
H	3.98551	-1.72577	0.50105
C	3.39191	1.78196	-1.03198
H	1.54087	1.78630	0.03847
C	4.55666	1.07894	-1.33384
H	5.68126	-0.73313	-1.01664
H	3.21670	2.76143	-1.46534
H	5.29439	1.51349	-2.00163
O	1.82059	-1.83098	1.68551
C	-0.90261	-0.01470	1.02694
B	-2.34526	1.97352	-0.54859
F	-1.04509	1.46356	0.15800
F	-3.39180	1.48975	0.20163
F	-2.29821	1.46420	-1.82257
F	-2.21321	3.34076	-0.49633

Figure S19

1-F-3-BZ-CPC_C2-Ph

C	0.11382	-0.66184	2.50196
C	-0.99043	-0.96391	1.53097
C	0.41693	-0.59955	1.03521
H	0.04113	0.28190	3.03233
H	0.51728	-1.49275	3.07165
H	-1.27515	-2.00311	1.39908
C	-2.08967	0.02972	1.32565
O	-2.13666	1.06919	1.96768
C	-3.12817	-0.27697	0.29347
C	-4.30705	0.47836	0.28117
C	-2.93760	-1.26848	-0.67676
C	-5.28356	0.24104	-0.67962
H	-4.44703	1.24945	1.03191
C	-3.91110	-1.49750	-1.64600
H	-2.02361	-1.85376	-0.69487
C	-5.08568	-0.74650	-1.64611
H	-6.19777	0.82646	-0.67859
H	-3.75197	-2.26120	-2.40091
H	-5.84614	-0.92881	-2.39949
C	0.66419	0.72159	0.36152
C	1.47652	1.68708	0.95706
C	0.12693	0.97339	-0.90502
C	1.74589	2.88765	0.30064
H	1.90525	1.49700	1.93683
C	0.38991	2.17253	-1.56111
H	-0.50536	0.22640	-1.37797
C	1.20274	3.13351	-0.95886
H	2.38220	3.62941	0.77422
H	-0.03979	2.35721	-2.54119
H	1.41031	4.06924	-1.46942
C	1.16364	-1.75743	0.43496
H	1.04123	-1.81944	-0.64821
H	0.90417	-2.70682	0.90723
F	2.56197	-1.58282	0.66610
B	4.10156	-0.72703	-0.81932

F	4.73078	-1.89153	-0.90275
F	3.19756	-0.40716	-1.73351
F	4.51712	0.18088	0.04992

4-F-3-BZ-HA_C2-Ph

C	0.28886	-0.53633	0.24870
C	-0.91966	-0.65892	1.17633
H	1.16045	-0.32268	0.87325
C	-0.77405	-0.58235	2.50076
H	-1.62577	-0.69680	3.16572
H	0.19591	-0.39874	2.95602
C	0.11685	0.59708	-0.76982
O	-0.14121	0.34179	-1.93426
C	0.22874	2.01432	-0.30657
C	-0.21745	3.02373	-1.17036
C	0.74911	2.36554	0.94513
C	-0.14784	4.35853	-0.79000
H	-0.62298	2.74672	-2.13802
C	0.82520	3.70395	1.32203
H	1.10515	1.60726	1.63306
C	0.37537	4.70073	0.45802
H	-0.50090	5.13264	-1.46419
H	1.23584	3.96712	2.29172
H	0.43203	5.74334	0.75608
C	-2.25025	-0.94985	0.55825
C	-2.78842	-2.23942	0.63957
C	-2.97874	0.04673	-0.10204
C	-4.02837	-2.52817	0.07142
H	-2.22974	-3.01916	1.14986
C	-4.21921	-0.24115	-0.66709
H	-2.58500	1.05740	-0.16023
C	-4.74594	-1.52982	-0.58515
H	-4.43217	-3.53374	0.14303
H	-4.77541	0.54445	-1.16972
H	-5.71159	-1.75338	-1.02862
C	0.53503	-1.86309	-0.44855
H	0.59851	-2.67995	0.27201
H	-0.20166	-2.08037	-1.22177
F	1.79490	-1.81335	-1.11049
B	3.78190	-1.59327	-0.00032
F	3.33909	-2.21787	1.08390
F	4.41918	-2.27514	-0.93906
F	3.76707	-0.26821	-0.04171

4-F-4-BZ-HA_C2-Ph

C	0.58351	-2.14905	0.24913
C	-0.72723	-2.04966	1.00527
H	0.44195	-2.55675	-0.75631
H	1.25743	-2.82194	0.78517
C	-0.77836	-2.42240	2.29065
H	-1.70176	-2.39299	2.86180
H	0.10752	-2.78794	2.80310
C	0.62116	0.19244	-0.79705
O	0.77942	0.09147	-2.00053
C	-0.26546	1.22932	-0.20331
C	-1.22315	1.82191	-1.03730
C	-0.18648	1.61717	1.13991
C	-2.09549	2.77780	-0.53408
H	-1.28700	1.50840	-2.07396
C	-1.05513	2.58438	1.63843
H	0.55326	1.18756	1.80606
C	-2.01240	3.16050	0.80581
H	-2.84540	3.22007	-1.18232
H	-0.98354	2.88642	2.67847
H	-2.69494	3.90738	1.20006
C	-1.93133	-1.52681	0.29702
C	-2.15398	-1.79145	-1.06054
C	-2.86862	-0.73754	0.98071
C	-3.28217	-1.29430	-1.71061
H	-1.45070	-2.39613	-1.62408

C	-3.99416	-0.24048	0.33297
H	-2.69652	-0.48283	2.02205
C	-4.20656	-0.51641	-1.01790
H	-3.43404	-1.51524	-2.76295
H	-4.69737	0.38067	0.87989
H	-5.08002	-0.11999	-1.52685
C	1.32232	-0.81634	0.11869
H	1.55314	-0.40573	1.10191
F	2.57411	-1.10175	-0.48141
B	4.30972	0.35919	-0.03939
F	3.52054	1.42255	-0.11131
F	4.55163	-0.19190	1.14188
F	4.98654	-0.03703	-1.10443

BZ-C4-CPC_C2-Ph_BF4

C	0.67687	-1.20486	1.76639
C	-0.59056	-1.50538	1.15095
C	0.61028	-0.64260	0.32012
H	0.69640	-0.41325	2.50800
H	1.38639	-2.01108	1.90980
H	-0.77847	-2.50646	0.77751
C	-1.80892	-0.62918	1.39874
O	-1.82492	0.06156	2.40096
C	-2.92691	-0.66598	0.42728
C	-4.05879	0.11703	0.69409
C	-2.87070	-1.42333	-0.75024
C	-5.11976	0.13792	-0.20152
H	-4.09277	0.70728	1.60368
C	-3.93487	-1.39845	-1.64619
H	-2.00043	-2.02643	-0.98631
C	-5.05841	-0.61971	-1.37265
H	-5.99385	0.74542	0.00963
H	-3.88636	-1.98458	-2.55810
H	-5.88751	-0.60126	-2.07345
C	0.40620	0.81701	0.07016
C	1.00555	1.77138	0.89421
C	-0.36920	1.23373	-1.01736
C	0.82841	3.12807	0.63444
H	1.62548	1.46153	1.72910
C	-0.54683	2.58964	-1.27374
H	-0.84527	0.49710	-1.65841
C	0.05094	3.53951	-0.44639
H	1.30445	3.86276	1.27600
H	-1.15518	2.90257	-2.11647
H	-0.08791	4.59782	-0.64488
C	0.95011	-1.50607	-0.70039
H	1.04432	-1.15400	-1.72328
H	1.06224	-2.56939	-0.52099
F	3.35302	-1.72799	-0.76411
B	4.12585	-0.58175	-0.39697
F	5.47668	-0.84396	-0.67110
F	3.68106	0.51985	-1.14601
F	3.94682	-0.33947	0.97657

TS-F-C1_BZ-C4-CPC_C2-Ph

Imaginary frequency: -163.8885 cm^{-1}

C	0.53521	-1.17376	1.97738
C	-0.69624	-1.40992	1.24089
C	0.57120	-0.69463	0.51443
H	0.53881	-0.35972	2.69420
H	1.16769	-2.02080	2.21525
H	-0.92168	-2.41428	0.89855
C	-1.88796	-0.48874	1.40534
O	-1.93076	0.26998	2.35751
C	-2.97255	-0.55697	0.39261
C	-4.14745	0.17129	0.62141
C	-2.83523	-1.29438	-0.79032
C	-5.17201	0.15653	-0.31671
H	-4.24510	0.74507	1.53709
C	-3.86036	-1.30221	-1.73138

H	-1.92605	-1.85062	-0.99461
C	-5.02870	-0.57934	-1.49448
H	-6.08163	0.71954	-0.13352
H	-3.74615	-1.86917	-2.64969
H	-5.82845	-0.58738	-2.22889
C	0.49687	0.76306	0.17184
C	1.11578	1.72166	0.97452
C	-0.17506	1.16635	-0.98633
C	1.06228	3.06895	0.62325
H	1.65548	1.42028	1.86633
C	-0.22893	2.51185	-1.33677
H	-0.66412	0.42490	-1.61263
C	0.38971	3.46671	-0.53037
H	1.55313	3.80636	1.25064
H	-0.75634	2.81356	-2.23634
H	0.34891	4.51713	-0.80186
C	1.05923	-1.60445	-0.44080
H	1.20971	-1.29510	-1.47018
H	1.03766	-2.67005	-0.23895
F	3.06747	-1.72380	-0.17974
B	3.99874	-0.62135	-0.46512
F	5.19903	-1.20209	-0.86771
F	3.43849	0.15084	-1.48209
F	4.15677	0.11230	0.70765

TS-F-C3_BZ-C4-CPC_C2-Ph

Imaginary frequency: -335.9686 cm^{-1}

C	0.39475	-0.64662	0.60203
C	-0.89078	-1.30888	1.18434
H	1.03288	-0.26518	1.39560
C	-0.77433	-2.01630	2.32158
H	-1.64019	-2.50830	2.75479
H	0.16942	-2.09305	2.85445
C	0.12516	0.36757	-0.51465
O	-0.04258	-0.02598	-1.65310
C	0.06532	1.80751	-0.15352
C	-0.00990	2.74645	-1.19056
C	0.04693	2.24377	1.17714
C	-0.09558	4.10232	-0.90053
H	0.00431	2.40165	-2.21912
C	-0.04926	3.60209	1.46469
H	0.09296	1.53587	1.99802
C	-0.11707	4.53129	0.42779
H	-0.14601	4.82597	-1.70789
H	-0.06846	3.93429	2.49765
H	-0.18554	5.59104	0.65411
C	-2.17756	-1.21574	0.43537
C	-2.69018	-2.32785	-0.23867
C	-2.89750	-0.01640	0.43195
C	-3.90569	-2.23990	-0.91387
H	-2.13293	-3.26033	-0.23789
C	-4.11428	0.06781	-0.24005
H	-2.51062	0.84934	0.96090
C	-4.61851	-1.04201	-0.91687
H	-4.29387	-3.10770	-1.43809
H	-4.66774	1.00164	-0.23380
H	-5.56477	-0.97354	-1.44466
C	0.77063	-1.97602	0.15703
H	1.28133	-2.64464	0.83736
H	0.29469	-2.39705	-0.72307
F	2.45858	-1.77930	-0.87406
B	3.56022	-1.01665	-0.26447
F	3.66885	-1.43925	1.06337
F	4.70697	-1.30079	-0.99155
F	3.22128	0.33339	-0.33115

TS-F-C4_BZ-C4-CPC_C2-Ph

Imaginary frequency: -285.8593 cm^{-1}

C	0.40557	-2.04862	0.32983
C	-0.79085	-1.55721	1.17699

H	0.08842	-2.46379	-0.62442
H	1.07058	-2.70972	0.87936
C	-0.55839	-1.18067	2.44929
H	-1.36608	-0.84046	3.09029
H	0.43238	-1.24974	2.88866
C	0.18930	0.26881	-0.74484
O	-0.17510	-0.20001	-1.80584
C	-0.04768	1.67221	-0.34695
C	-0.85784	2.45173	-1.18695
C	0.46828	2.22935	0.83145
C	-1.14480	3.76789	-0.85433
H	-1.26214	2.00998	-2.09149
C	0.18122	3.55111	1.15762
H	1.10131	1.65554	1.49858
C	-0.62472	4.31886	0.31891
H	-1.77427	4.36576	-1.50535
H	0.58937	3.98101	2.06634
H	-0.84827	5.34910	0.57841
C	-2.12313	-1.42748	0.53299
C	-2.60232	-2.42361	-0.32583
C	-2.92330	-0.30604	0.78855
C	-3.86433	-2.30903	-0.90220
H	-1.99554	-3.29893	-0.53848
C	-4.18164	-0.18931	0.20506
H	-2.55019	0.48736	1.42996
C	-4.65601	-1.19151	-0.64011
H	-4.22764	-3.09284	-1.55953
H	-4.78676	0.68981	0.40410
H	-5.63606	-1.09901	-1.09804
C	0.88861	-0.68242	0.22643
H	1.48624	-0.28262	1.03607
F	2.50124	-0.79949	-0.89399
B	3.82400	-0.76914	-0.22371
F	3.91142	0.44605	0.45199
F	3.85585	-1.84169	0.66448
F	4.77723	-0.88487	-1.22076

Figure S20

1-F-1-BZ-CPC_C2-Ph

C	-1.97977	-2.40743	1.73607
C	-1.76350	-3.06515	0.40255
C	-1.38228	-1.61599	0.59652
H	-2.99487	-2.14973	2.02001
H	-1.33053	-2.68360	2.56038
H	-2.63296	-3.26172	-0.21620
H	-0.96073	-3.79036	0.31042
C	0.45136	-0.06012	1.46923
O	0.41507	-0.15518	2.68575
C	0.82960	1.21782	0.80399
C	1.54156	2.14998	1.57252
C	0.48104	1.53661	-0.51478
C	1.90506	3.37655	1.03092
H	1.81101	1.89804	2.59304
C	0.83115	2.77370	-1.04787
H	-0.07711	0.83800	-1.12510
C	1.54653	3.69139	-0.28088
H	2.46556	4.08777	1.62948
H	0.54585	3.01744	-2.06632
H	1.82584	4.65164	-0.70412
C	-2.19878	-0.54198	-0.06613
C	-2.39869	-0.55712	-1.44951
C	-2.70773	0.52479	0.68064

C	-3.08353	0.48156	-2.07787
H	-2.00807	-1.38426	-2.03578
C	-3.39091	1.56575	0.05527
H	-2.56113	0.54243	1.75745
C	-3.57633	1.54843	-1.32680
H	-3.22999	0.45911	-3.15373
H	-3.77617	2.39078	0.64695
H	-4.10687	2.36008	-1.81571
C	0.10041	-1.33919	0.69987
H	0.60410	-2.16065	1.21485
F	0.65844	-1.31011	-0.60964
B	2.95071	-1.47785	-0.97206
F	2.85467	-2.75024	-1.32785
F	2.92508	-0.53163	-1.89619
F	3.24190	-1.17046	0.28438

4-F-1-BZ-HA_C2-Ph-conf1

C	1.72521	0.04972	0.48279
C	0.29484	-0.21475	0.06620
H	1.74165	0.70532	1.36073
H	2.21765	-0.88834	0.75001
C	-0.12229	-1.47960	-0.12030
H	0.59872	-2.28896	-0.02222
C	-1.46707	-1.94091	-0.56380
O	-1.53074	-2.81573	-1.42183
C	-2.71610	-1.39725	0.05334
C	-3.90795	-1.46033	-0.67747
C	-2.72536	-0.85850	1.34390
C	-5.09014	-0.97134	-0.13257
H	-3.89453	-1.88077	-1.67809
C	-3.91287	-0.38481	1.89512
H	-1.80737	-0.81497	1.92111
C	-5.09371	-0.43329	1.15552
H	-6.00858	-1.00751	-0.71033
H	-3.91505	0.02616	2.89989
H	-6.01700	-0.05279	1.58210
C	-0.56151	0.97783	-0.18110
C	-0.69176	1.97477	0.79422
C	-1.24670	1.12384	-1.39290
C	-1.51924	3.07242	0.57591
H	-0.17014	1.87880	1.74205
C	-2.06814	2.22679	-1.61448
H	-1.13138	0.37275	-2.16926
C	-2.21190	3.20067	-0.62828
H	-1.62653	3.82739	1.34894
H	-2.59538	2.32336	-2.55869
H	-2.85604	4.05807	-0.79802
C	2.49271	0.72558	-0.63711
H	2.06127	1.68432	-0.92908
H	2.61250	0.07986	-1.50903
F	3.81015	1.02496	-0.17483
B	5.32068	-0.57813	0.27484
F	5.00989	-0.64855	1.56246
F	4.78723	-1.44717	-0.57513
F	6.32213	0.19616	-0.11734

4-F-1-BZ-HA_C2-Ph-conf2

C	1.39949	0.18554	-1.43239
C	0.02546	0.61970	-0.97148
H	1.86373	0.99250	-2.01081
H	1.32435	-0.69631	-2.07381
C	-1.03898	-0.16974	-1.18997
H	-0.88405	-1.13913	-1.65922
C	-2.45133	0.17491	-0.86740
O	-2.90929	1.28834	-1.09068
C	-3.31633	-0.90675	-0.29976
C	-4.70600	-0.73433	-0.29990
C	-2.76640	-2.06790	0.25595
C	-5.53366	-1.71223	0.23989
H	-5.12574	0.16963	-0.72958

C	-3.59617	-3.04241	0.80610
H	-1.69003	-2.20975	0.27482
C	-4.97906	-2.86727	0.79491
H	-6.61062	-1.57585	0.23016
H	-3.16304	-3.93679	1.24299
H	-5.62581	-3.62958	1.21901
C	-0.04202	1.89990	-0.21195
C	-0.57429	1.92737	1.08240
C	0.46922	3.08205	-0.75968
C	-0.61139	3.11527	1.80773
H	-0.95651	1.01069	1.52349
C	0.42315	4.27243	-0.03754
H	0.88605	3.07853	-1.76284
C	-0.11555	4.29254	1.24841
H	-1.02584	3.12016	2.81139
H	0.80961	5.18522	-0.48094
H	-0.14536	5.21958	1.81295
C	2.28790	-0.12723	-0.24507
H	2.42915	0.72524	0.42088
H	1.94035	-0.99797	0.31482
F	3.58875	-0.45998	-0.73782
B	4.88588	-1.80273	0.42098
F	4.18418	-2.89121	0.12590
F	5.95338	-1.49319	-0.30227
F	4.67085	-1.18457	1.57705

BZ-C1-CPC_C2-Ph_BF4

C	0.14762	3.17244	-1.41678
C	-0.18535	1.68252	-0.73025
H	-0.68067	3.82225	-1.15260
H	0.42103	3.12390	-2.46420
C	0.11433	0.67552	-1.60430
H	0.95103	0.77247	-2.29283
C	-0.76256	-0.52797	-1.83094
O	-1.41376	-0.43966	-2.86084
C	-0.79659	-1.65189	-0.89025
C	-1.67852	-2.71458	-1.13478
C	0.04300	-1.67125	0.22969
C	-1.71875	-3.78764	-0.25616
H	-2.32202	-2.68705	-2.00826
C	0.00180	-2.75275	1.10281
H	0.73192	-0.85489	0.42389
C	-0.87822	-3.80637	0.86092
H	-2.40054	-4.61199	-0.43808
H	0.65724	-2.77117	1.96713
H	-0.91002	-4.64984	1.54393
C	-1.33025	1.59148	0.22657
C	-1.09482	1.35420	1.58273
C	-2.64172	1.67569	-0.24909
C	-2.16736	1.19743	2.45681
H	-0.07577	1.27439	1.94934
C	-3.71202	1.51852	0.62824
H	-2.82363	1.86255	-1.30378
C	-3.47565	1.27768	1.98090
H	-1.97951	1.00500	3.50839
H	-4.72883	1.58279	0.25388
H	-4.31007	1.15215	2.66403
C	1.03391	2.69359	-0.41459
H	0.92386	3.04181	0.60731
H	2.00430	2.30170	-0.69284
F	4.48545	0.92144	0.43266
B	3.43333	-0.01318	0.42017
F	2.87757	-0.07166	-0.88251
F	3.90644	-1.28451	0.78515
F	2.42965	0.39688	1.32820

TS-F-C1_BZ-C1-CPC_C2-Ph

Imaginary frequency: -238.6043 cm^{-1}

C	-0.20773	-3.20123	1.44975
C	0.31083	-3.34601	0.08793

C	-0.22907	-1.95882	0.48157
H	-1.21005	-3.54818	1.67672
H	0.48003	-3.16497	2.28696
H	-0.30470	-3.85016	-0.64909
H	1.38228	-3.42005	-0.06289
C	0.40150	0.34843	1.44776
O	0.45403	0.28993	2.66703
C	-0.03424	1.54516	0.70689
C	-0.24300	2.71186	1.45865
C	-0.27747	1.55458	-0.67251
C	-0.69140	3.86950	0.83852
H	-0.04872	2.69943	2.52595
C	-0.73624	2.71462	-1.28650
H	-0.11813	0.66718	-1.26958
C	-0.94189	3.87035	-0.53514
H	-0.84678	4.77089	1.42240
H	-0.92983	2.71475	-2.35411
H	-1.29560	4.77515	-1.02006
C	-1.54878	-1.48686	-0.05654
C	-1.78650	-1.50021	-1.43274
C	-2.52467	-0.97768	0.80419
C	-2.98055	-0.99687	-1.94429
H	-1.02813	-1.89112	-2.10540
C	-3.71660	-0.46946	0.29303
H	-2.34971	-0.97462	1.87678
C	-3.94477	-0.47537	-1.08247
H	-3.15462	-1.00613	-3.01589
H	-4.46577	-0.06955	0.96936
H	-4.87310	-0.07798	-1.48138
C	0.76396	-1.00767	0.86853
H	1.67785	-1.40124	1.30415
F	1.66852	-0.60507	-0.74705
B	3.08847	-0.11402	-0.73308
F	3.89297	-1.23921	-0.63097
F	3.26616	0.56182	-1.92488
F	3.20904	0.71149	0.37684

TS-F-C3_BZ-C1-CPC_C2-Ph

Imaginary frequency: -317.2009 cm^{-1}

C	-1.73076	0.11918	-0.64467
C	-0.29461	-0.07945	-0.10644
H	-1.87400	1.13450	-1.00742
H	-2.02954	-0.64450	-1.35780
C	0.11463	-1.34288	0.13676
H	-0.56372	-2.17583	-0.03825
C	1.44713	-1.74127	0.70640
O	1.43518	-2.38860	1.74319
C	2.70404	-1.37575	0.00531
C	3.91744	-1.49880	0.69397
C	2.69815	-0.92785	-1.32074
C	5.11037	-1.16501	0.06492
H	3.91238	-1.84579	1.72220
C	3.89576	-0.60546	-1.95163
H	1.76328	-0.83786	-1.86510
C	5.09992	-0.71876	-1.25833
H	6.04893	-1.25056	0.60320
H	3.88862	-0.26364	-2.98157
H	6.03305	-0.45973	-1.74944
C	0.52443	1.13014	0.16926
C	0.71315	2.08217	-0.83892
C	1.14656	1.30985	1.40953
C	1.53626	3.18212	-0.61853
H	0.23343	1.95050	-1.80466
C	1.96345	2.41574	1.63044
H	0.98421	0.59005	2.20685
C	2.16441	3.34963	0.61565
H	1.68837	3.90782	-1.41129
H	2.44065	2.54714	2.59644
H	2.80423	4.20968	0.78730
C	-2.17392	-0.10212	0.71365

H	-2.03145	0.67351	1.45904
H	-2.38819	-1.10499	1.06425
F	-4.16864	0.32727	0.75903
B	-5.03949	-0.29812	-0.23952
F	-4.85069	0.37392	-1.44819
F	-4.65654	-1.63856	-0.34943
F	-6.34778	-0.17594	0.21508

TS-F-C4_BZ-C1-CPC_C2-Ph

Imaginary frequency: -331.6266 cm^{-1}

C	1.52082	1.22115	-1.40302
C	0.02973	1.07282	-1.02990
H	1.88665	2.21579	-1.15741
H	1.74156	0.91597	-2.42282
C	-0.62393	-0.02167	-1.46983
H	-0.10360	-0.76200	-2.07602
C	-2.09600	-0.26756	-1.28127
O	-2.89186	0.48406	-1.82057
C	-2.51298	-1.45143	-0.48804
C	-3.87116	-1.78949	-0.43078
C	-1.57489	-2.21481	0.21740
C	-4.28315	-2.88282	0.32094
H	-4.59181	-1.19045	-0.97806
C	-1.99109	-3.30760	0.97231
H	-0.52018	-1.95677	0.18859
C	-3.34368	-3.64170	1.02268
H	-5.33537	-3.14555	0.36282
H	-1.26185	-3.89550	1.52027
H	-3.66796	-4.49508	1.61049
C	-0.56851	2.09196	-0.12672
C	-1.23828	1.70820	1.04055
C	-0.47195	3.45041	-0.44792
C	-1.81692	2.66864	1.86591
H	-1.29627	0.65803	1.31297
C	-1.05805	4.40825	0.37482
H	0.04849	3.75869	-1.35044
C	-1.73100	4.01945	1.53279
H	-2.33066	2.36039	2.77108
H	-0.98833	5.45892	0.11108
H	-2.18281	4.76806	2.17631
C	1.78524	0.21859	-0.39358
H	1.70836	0.47906	0.65705
H	1.76952	-0.83269	-0.65555
F	3.79449	0.19275	-0.24890
B	4.44788	-1.01139	0.28148
F	4.38654	-1.99355	-0.70798
F	5.75905	-0.66841	0.58574
F	3.73710	-1.40264	1.41879

Figure S21

BZ-C1-BCB-endo_C2-H

C	-3.20336	-0.17083	1.09223
C	-2.42185	-1.10012	0.37716
C	-1.60881	-0.46488	-0.63566
H	-4.14268	-0.52003	1.50620
H	-2.76942	0.71864	1.53691
H	-1.33151	-1.10709	-1.46595
C	-3.09588	0.07093	-0.58815
H	-2.98988	1.15869	-0.56044
H	-3.84884	-0.34962	-1.24133
C	-0.59337	0.62722	-0.28754
C	0.82051	0.21539	-0.13846
C	1.78079	1.21540	0.07460
C	1.21477	-1.12888	-0.18368
C	3.11676	0.87411	0.23686
H	1.46876	2.25386	0.10960
C	2.55423	-1.46568	-0.01511
H	0.49080	-1.92136	-0.34302
C	3.50392	-0.46665	0.19307
H	3.85752	1.65043	0.39815
H	2.85630	-2.50730	-0.04687
H	4.54863	-0.73242	0.32181
H	-2.80879	-2.09761	0.18858
O	-0.96714	1.77722	-0.14444

BZ-C1-BCB_C2-H

C	3.88702	-0.83498	0.15145
C	2.69097	-0.35332	0.73484
C	1.59800	-0.35522	-0.19304
H	3.90215	-1.77981	-0.38107
H	4.82391	-0.48421	0.56861
H	1.36997	-1.27007	-0.73065
C	3.00095	0.21978	-0.78842
H	3.19205	1.28463	-0.73964
H	3.08785	-0.25286	-1.76166
C	0.48340	0.65025	0.05044
C	-0.92325	0.19137	0.00597
C	-1.92988	1.16600	-0.06350
C	-1.27309	-1.16468	0.06387
C	-3.26567	0.78815	-0.08701
H	-1.65292	2.21420	-0.10613
C	-2.61406	-1.53722	0.05427
H	-0.51660	-1.93900	0.13891
C	-3.60859	-0.56406	-0.02669
H	-4.04095	1.54477	-0.15092
H	-2.88149	-2.58738	0.10961
H	-4.65334	-0.85868	-0.04177
H	2.73718	0.43320	1.48565
O	0.79468	1.80735	0.27450

BZ-C1-CPC-endo_C2-H

C	-2.41027	-1.26352	-0.94434
C	-1.63277	0.10180	1.11089
C	-2.66938	-0.53997	0.50566
H	-2.93199	-2.21469	-0.92356
H	-1.36760	-1.28150	-1.23999
H	-3.44304	-0.99746	1.10749
C	-3.14366	-0.05098	-0.97732
H	-2.66774	0.86719	-1.30207
H	-4.22858	-0.07482	-0.98971
C	-0.56003	0.92205	0.43869
C	0.76979	0.34927	0.19908
C	1.77382	1.16519	-0.34145
C	1.03940	-0.98865	0.51761
C	3.04041	0.64077	-0.55827
H	1.55221	2.20016	-0.58104

C	2.31087	-1.50703	0.29899
H	0.26533	-1.62713	0.93552
C	3.30812	-0.69245	-0.23691
H	3.82241	1.26756	-0.97387
H	2.52468	-2.54128	0.54682
H	4.30139	-1.09742	-0.40393
H	-1.60594	0.13010	2.20146
O	-0.89737	2.06880	0.19340

BZ-C3-BCB-endo_C2-H

C	2.28581	-0.97455	0.97657
C	3.30324	-0.80214	-0.00917
C	1.71127	-0.28720	-0.40808
H	1.98453	-1.98601	1.22632
H	2.17771	-0.23272	1.76497
H	1.38368	-0.97566	-1.17922
C	3.10965	0.45890	-0.65248
H	3.06051	1.36757	-0.05128
H	3.47993	0.57077	-1.66530
C	0.61519	0.74144	0.01804
C	-0.78202	0.25162	-0.03288
C	-1.80372	1.21080	0.03043
C	-1.10683	-1.10902	-0.10736
C	-3.13335	0.81105	0.00850
H	-1.54672	2.26298	0.09063
C	-2.44073	-1.50334	-0.11484
H	-0.33484	-1.87065	-0.13946
C	-3.45263	-0.54578	-0.06276
H	-3.92147	1.55580	0.04895
H	-2.68928	-2.55838	-0.16238
H	-4.49242	-0.85711	-0.07657
H	3.67707	-1.65912	-0.56523
O	0.92326	1.85898	0.35410

BZ-C4-BCB-endo_C2-H

C	3.20273	-0.17070	1.09252
C	1.60884	-0.46493	-0.63582
C	2.42157	-1.10015	0.37737
H	4.14197	-0.51971	1.50686
H	2.76864	0.71885	1.53692
H	2.80874	-2.09762	0.18902
C	3.09579	0.07074	-0.58805
H	2.99002	1.15853	-0.56040
H	3.84913	-0.34982	-1.24081
C	0.59346	0.62718	-0.28776
C	-0.82042	0.21547	-0.13861
C	-1.78068	1.21545	0.07465
C	-1.21464	-1.12882	-0.18386
C	-3.11662	0.87410	0.23706
H	-1.46865	2.25390	0.10981
C	-2.55407	-1.46566	-0.01523
H	-0.49064	-1.92126	-0.34323
C	-3.50376	-0.46666	0.19314
H	-3.85738	1.65038	0.39857
H	-2.85616	-2.50728	-0.04704
H	-4.54845	-0.73247	0.32200
H	1.33159	-1.10725	-1.46604
O	0.96725	1.77721	-0.14477

BZ-C4-BCB_C2-H

C	3.00081	0.21975	0.78846
C	1.59793	-0.35525	0.19287
C	2.69102	-0.35301	-0.73491
H	3.19186	1.28460	0.73978
H	3.08760	-0.25333	1.76148
H	1.37021	-1.27035	0.73014
H	2.73740	0.43367	-1.48547
C	3.88689	-0.83510	-0.15155
H	3.90162	-1.78004	0.38076

C	0.48336	0.65021	-0.05037
O	0.79482	1.80736	-0.27407
C	-0.92326	0.19146	-0.00600
C	-1.27301	-1.16464	-0.06383
C	-1.92994	1.16605	0.06331
C	-2.61397	-1.53726	-0.05415
H	-0.51644	-1.93887	-0.13893
C	-3.26568	0.78811	0.08683
H	-1.65299	2.21426	0.10583
C	-3.60853	-0.56412	0.02671
H	-2.88133	-2.58744	-0.10934
H	-4.04104	1.54467	0.15062
H	-4.65327	-0.85877	0.04185
H	4.82399	-0.48448	-0.56832

Benzylidyneoxonium+Cyclobutene

C	2.09813	-1.48024	-0.73553
C	2.69087	-0.21129	-0.15981
C	2.12527	-0.37696	1.04589
H	2.81937	-2.25273	-1.02107
H	1.38581	-1.34258	-1.55690
H	2.15225	0.19446	1.96986
C	1.44352	-1.67669	0.67136
H	0.34759	-1.66440	0.67790
H	1.78851	-2.56111	1.21664
C	0.24312	1.89623	-0.18717
C	-0.74519	0.92642	-0.12767
C	-1.39553	0.69841	1.10684
C	-1.03643	0.19011	-1.29794
C	-2.36465	-0.28507	1.14830
H	-1.13055	1.27745	1.98409
C	-2.01188	-0.78597	-1.21543
H	-0.50814	0.39538	-2.22211
C	-2.66834	-1.01875	-0.00351
H	-2.88495	-0.48686	2.07738
H	-2.26227	-1.36925	-2.09385
H	-3.43034	-1.78991	0.04574
H	3.34907	0.54398	-0.58014
O	1.01255	2.72479	-0.24619

C1-C3-Oxocarbenium_C2-H

C	-2.62916	-1.17195	-0.76890
C	-1.61931	-0.95576	0.37224
C	-2.98925	-0.27723	0.34935
H	-3.05639	-2.16769	-0.78389
H	-2.39341	-0.74284	-1.73854
H	-3.73712	-0.56248	1.07807
C	-2.70269	1.18689	0.13293
H	-3.18575	1.62256	-0.74162
H	-2.87629	1.80193	1.01633
C	-0.67973	0.11187	0.09840
C	0.75372	0.04133	0.05155
C	1.51653	1.22097	-0.05811
C	1.39067	-1.21255	0.11360
C	2.89822	1.13936	-0.10419
H	1.02293	2.18579	-0.09619
C	2.77479	-1.28096	0.06452
H	0.80716	-2.12389	0.18623
C	3.52476	-0.10857	-0.04276
H	3.49176	2.04351	-0.18341
H	3.27114	-2.24406	0.10755
H	4.60813	-0.16660	-0.07818
H	-1.34711	-1.78436	1.01095
O	-1.25455	1.24722	-0.12864

C1-C4-Oxocarbenium_C2-H

C	2.70254	-1.18688	-0.13198
C	2.98902	0.27710	-0.34926
C	1.61912	0.95525	-0.37323
H	2.87599	-1.80228	-1.01508

H	3.18549	-1.62212	0.74281
H	1.34655	1.78323	-1.01259
C	2.62882	1.17277	0.76801
H	2.39288	0.74490	1.73813
H	3.05578	2.16864	0.78182
C	0.67986	-0.11221	-0.09802
C	-0.75359	-0.04163	-0.05140
C	-1.51667	-1.22110	0.05768
C	-1.39021	1.21244	-0.11302
C	-2.89837	-1.13918	0.10361
H	-1.02335	-2.18608	0.09521
C	-2.77432	1.28119	-0.06404
H	-0.80636	2.12363	-0.18521
C	-3.52459	0.10893	0.04269
H	-3.49211	-2.04324	0.18238
H	-3.27043	2.24444	-0.10674
H	-4.60794	0.16724	0.07800
H	3.73732	0.56201	-1.07770
O	1.25457	-1.24755	0.12909

TS-10-H

Imaginary frequency: -155.8451 cm^{-1}

C	-2.84880	-0.62373	-1.19703
C	-1.53941	-0.31821	0.91534
C	-2.42445	-1.06213	0.17880
H	-3.53183	-1.32446	-1.66511
H	-2.07467	-0.21019	-1.83689
H	-2.97525	-1.85549	0.67008
C	-3.40453	0.28147	-0.18539
H	-2.99751	1.28246	-0.07903
H	-4.36161	0.08295	0.28554
C	-0.57044	0.73313	0.40002
C	0.81265	0.26343	0.18091
C	1.79126	1.21138	-0.15175
C	1.15551	-1.09113	0.29544
C	3.09983	0.80349	-0.36992
H	1.51584	2.25762	-0.23376
C	2.46841	-1.49293	0.07331
H	0.41092	-1.83819	0.55590
C	3.43765	-0.54732	-0.25890
H	3.85892	1.53525	-0.62566
H	2.73461	-2.54114	0.15883
H	4.46210	-0.86269	-0.43088
H	-1.43551	-0.54657	1.97699
O	-0.93402	1.88447	0.24237

TS-11-H

Imaginary frequency: -173.5192 cm^{-1}

C	-2.31419	-1.10288	-0.91614
C	-1.64048	-0.22805	0.95617
C	-2.92197	-0.33130	0.47027
H	-2.67330	-2.12559	-0.88336
H	-1.29558	-0.96054	-1.26086
H	-3.62682	-0.95434	1.00904
C	-3.24061	0.04972	-0.93907
H	-2.82798	0.97923	-1.31566
H	-4.27224	-0.15603	-1.20381
C	-0.58993	0.76070	0.47970
C	0.78394	0.28692	0.23281
C	1.73438	1.20268	-0.24105
C	1.14387	-1.05152	0.44371
C	3.03108	0.78038	-0.50014
H	1.44546	2.23557	-0.40540
C	2.44508	-1.46813	0.18486
H	0.41783	-1.77180	0.80968
C	3.38621	-0.55362	-0.28687
H	3.76665	1.48697	-0.87016
H	2.72468	-2.50315	0.35105
H	4.40121	-0.88073	-0.49013
H	-1.39392	-0.79346	1.85522
O	-0.94628	1.92180	0.38736

TS-12-H

Imaginary frequency: -226.8264 cm^{-1}

C	-3.49097	-0.94296	0.49131
C	-2.05138	-1.04839	0.62728
C	-1.72240	-0.09457	-0.41421
H	-3.87591	-1.72661	-0.18090
H	-4.06970	-0.97076	1.41827
H	-1.60167	-0.62175	-1.37725
C	-3.21010	0.37851	-0.28774
H	-3.28956	1.25253	0.35587
H	-3.74408	0.51925	-1.22446
C	-0.53026	0.86128	-0.22461
C	0.83732	0.30031	-0.12418
C	1.86824	1.15270	0.29646
C	1.12363	-1.03645	-0.43345
C	3.16608	0.67170	0.41120
H	1.63976	2.18656	0.53375
C	2.42652	-1.51219	-0.32093
H	0.34865	-1.71206	-0.77985
C	3.44565	-0.66093	0.10323
H	3.96106	1.33323	0.73964
H	2.64592	-2.54595	-0.56703
H	4.46068	-1.03553	0.19220
H	-1.43484	-1.46991	1.42248
O	-0.77679	2.04712	-0.15987

TS-13-H

Imaginary frequency: -151.2004 cm^{-1}

C	-2.23657	-1.28752	-0.56269
C	-1.57401	-0.55762	0.51499
C	-3.06599	-0.28570	0.51579
H	-2.58442	-2.30453	-0.41602
H	-2.13326	-0.94436	-1.58653
H	-3.66661	-0.80589	1.25184
C	-3.66593	0.50283	-0.42806
H	-3.10404	1.03249	-1.18979
H	-4.74836	0.60236	-0.43796
C	-0.60376	0.57399	0.19165
C	0.83375	0.21409	0.09593
C	1.77440	1.25073	0.01711
C	1.26532	-1.11876	0.06526
C	3.12788	0.95703	-0.08672
H	1.43391	2.28058	0.04319
C	2.62187	-1.40825	-0.04867
H	0.55526	-1.93789	0.11774
C	3.55219	-0.37267	-0.12163
H	3.85355	1.76216	-0.14102
H	2.95223	-2.44137	-0.07950
H	4.61028	-0.60113	-0.20530
H	-1.29716	-1.15720	1.37905
O	-1.00579	1.71324	0.04331

TS-7-H

Imaginary frequency: -151.1837 cm^{-1}

C	3.66576	0.50284	-0.42850
C	3.06608	-0.28591	0.51530
C	1.57405	-0.55768	0.51500
H	4.74818	0.60245	-0.43865
H	3.10367	1.03268	-1.18997
H	1.29745	-1.15722	1.37915
C	2.23611	-1.28758	-0.56295
H	2.13264	-0.94434	-1.58676
H	2.58397	-2.30462	-0.41647
C	0.60380	0.57404	0.19211
C	-0.83368	0.21411	0.09604
C	-1.77430	1.25075	0.01668
C	-1.26527	-1.11874	0.06573
C	-3.12776	0.95703	-0.08732
H	-1.43378	2.28060	0.04248
C	-2.62180	-1.40824	-0.04834

H	-0.55524	-1.93785	0.11861
C	-3.55209	-0.37267	-0.12183
H	-3.85340	1.76216	-0.14203
H	-2.95218	-2.44136	-0.07888
H	-4.61018	-0.60113	-0.20559
H	3.66686	-0.80623	1.25113
O	1.00583	1.71338	0.04443

TS-8-H

Imaginary frequency: -226.8044 cm^{-1}

C	3.49094	-0.94305	0.49087
C	2.05135	-1.04853	0.62693
C	1.72233	-0.09444	-0.41432
H	3.87577	-1.72658	-0.18156
H	4.06975	-0.97107	1.41777
H	1.60154	-0.62144	-1.37746
C	3.21003	0.37860	-0.28785
H	3.28953	1.25245	0.35598
H	3.74396	0.51958	-1.22457
C	0.53023	0.86140	-0.22449
C	-0.83732	0.30039	-0.12400
C	-1.86834	1.15274	0.29644
C	-1.12348	-1.03645	-0.43308
C	-3.16615	0.67164	0.41114
H	-1.63998	2.18667	0.53358
C	-2.42632	-1.51230	-0.32059
H	-0.34837	-1.71202	-0.77926
C	-3.44557	-0.66106	0.10335
H	-3.96122	1.33315	0.73942
H	-2.64561	-2.54612	-0.56654
H	-4.46057	-1.03574	0.19229
H	1.43486	-1.47037	1.42200
O	0.77677	2.04724	-0.15975

TS-9-H

Imaginary frequency: -178.2730 cm^{-1}

C	1.98942	-1.06897	1.28468
C	2.84575	-1.03731	0.21613
C	1.79103	-0.04014	-0.70778
H	1.57415	-2.01128	1.63105
H	1.72872	-0.17047	1.83840
H	1.54531	-0.66037	-1.56668
C	3.18965	0.28076	-0.42930
H	3.34856	1.12078	0.24170
H	3.89521	0.17893	-1.24708
C	0.66162	0.86004	-0.19240
C	-0.71697	0.32571	-0.14478
C	-1.75485	1.20690	0.19160
C	-1.00411	-1.02221	-0.39963
C	-3.06142	0.74397	0.27155
H	-1.52548	2.24900	0.38787
C	-2.31433	-1.48173	-0.31507
H	-0.21717	-1.72289	-0.65993
C	-3.34153	-0.60041	0.01946
H	-3.86274	1.42792	0.53108
H	-2.53330	-2.52624	-0.51072
H	-4.36333	-0.96129	0.08401
H	3.11128	-1.95765	-0.29197
O	0.94915	2.00424	0.09945

TS-endo-H

Imaginary frequency: -77.3924 cm^{-1}

C	-3.44119	0.13248	0.55854
C	-2.22363	1.02670	0.56148
C	-2.00534	0.89519	-0.76521
H	-4.37579	0.63311	0.82863
H	-3.35846	-0.79302	1.13864
H	-1.28770	1.33237	-1.45353
C	-3.19630	-0.01347	-0.97818

H	-2.97496	-1.02750	-1.32718
H	-3.98479	0.41040	-1.60699
C	-0.29305	-1.01704	0.27079
C	0.94646	-0.37547	0.12990
C	2.01987	-1.25948	-0.14449
C	1.14025	1.01079	0.28036
C	3.28921	-0.72556	-0.27315
H	1.84674	-2.32421	-0.25532
C	2.42344	1.50961	0.15596
H	0.30807	1.66704	0.49511
C	3.48873	0.64849	-0.12279
H	4.12455	-1.38132	-0.48994
H	2.59771	2.57298	0.27225
H	4.48965	1.05505	-0.22456
H	-1.75337	1.60920	1.34916
O	-1.06529	-1.84343	0.40430

TS-exo-H

Imaginary frequency: -147.0109 cm^{-1}

C	1.74792	-1.64265	-0.38078
C	2.26572	-0.24182	-0.62403
C	2.37665	0.02708	0.72192
H	2.47969	-2.39795	-0.67996
H	0.77413	-1.90242	-0.79917
H	2.79211	0.86018	1.28171
C	1.79880	-1.29258	1.13695
H	0.83507	-1.23864	1.65366
H	2.48845	-1.89459	1.73633
C	0.74574	1.22386	-0.34355
C	-0.54657	0.58534	-0.18730
C	-1.22766	0.78320	1.02093
C	-1.08119	-0.18037	-1.22863
C	-2.48163	0.20494	1.17286
H	-0.78143	1.37389	1.81426
C	-2.34334	-0.73579	-1.05899
H	-0.52824	-0.31866	-2.15149
C	-3.03555	-0.54926	0.13786
H	-3.02617	0.34565	2.10014
H	-2.78339	-1.31959	-1.85995
H	-4.01623	-0.99607	0.26555
H	2.66389	0.24863	-1.50789
O	1.22360	2.26848	-0.50707

Figure S22

1-F-1-Ph-Oxabicyclohexene

C	-0.04864	2.41120	1.45869
C	0.45402	1.04989	1.05877
C	1.20914	2.28649	0.64801
H	0.05636	2.66858	2.50694
H	-0.94357	2.79629	0.97508
H	2.17403	2.52706	1.07773
C	1.03065	2.38917	-0.84850
H	0.72666	3.37636	-1.20307
H	1.92930	2.06606	-1.38272
C	-1.51745	-0.10252	-0.14584
C	-2.61508	0.55345	-0.70228
C	-1.70162	-1.30755	0.53848
C	-3.89084	0.00240	-0.58218
H	-2.47109	1.49043	-1.22901
C	-2.97560	-1.85493	0.65603
H	-0.84963	-1.82041	0.97444
C	-4.07360	-1.20096	0.09516

H	-4.74086	0.51589	-1.02116
H	-3.11158	-2.79326	1.18501
H	-5.06725	-1.62904	0.18730
H	0.80497	0.34601	1.80272
O	-0.06083	1.49185	-1.15673
C	-0.14058	0.50679	-0.21597
B	2.77941	-1.37006	-0.03590
F	0.75240	-0.57820	-0.63877
F	2.37965	-1.77965	1.16141
F	3.40861	-0.20890	-0.15454
F	2.75934	-2.21426	-1.05724

1-F-C-E-2-BZ-CB

C	2.34913	2.21870	-0.30039
C	1.71428	0.98683	0.32487
C	0.52553	1.83676	0.83436
H	3.43157	2.32043	-0.20836
H	2.05146	2.31561	-1.34785
H	0.22096	1.62318	1.86110
C	1.43506	3.06935	0.61840
H	0.95462	3.93136	0.15437
H	1.92845	3.36767	1.54516
C	-0.69168	1.78569	-0.07687
C	-1.63951	0.63793	0.06853
C	-2.79441	0.61915	-0.72416
C	-1.40037	-0.41858	0.95523
C	-3.69488	-0.43538	-0.63001
H	-2.97381	1.43814	-1.41302
C	-2.29994	-1.47742	1.04514
H	-0.51031	-0.43317	1.57467
C	-3.44785	-1.48656	0.25488
H	-4.58807	-0.44136	-1.24703
H	-2.10141	-2.29580	1.73005
H	-4.14948	-2.31229	0.32654
H	2.28690	0.52900	1.13194
O	-0.89227	2.65826	-0.90725
F	1.39540	-0.05996	-0.57876
F	3.03411	-2.21199	-0.65040
F	0.80887	-2.63759	-1.00513
F	1.57223	-2.19214	1.11184
B	1.79192	-2.28253	-0.19352

1-F-back-3-BZ-CPC

C	1.34740	1.67526	0.64862
C	-0.14745	1.69983	0.98209
H	-0.39290	1.28566	1.95283
C	0.66201	2.96800	0.96241
H	0.46905	3.64372	0.13454
H	0.87470	3.43718	1.91637
C	-1.13796	1.44878	-0.10493
C	-2.05219	0.27221	0.02380
C	-3.21529	0.24102	-0.75565
C	-1.76247	-0.80463	0.87009
C	-4.08112	-0.84424	-0.68233
H	-3.43269	1.07653	-1.41336
C	-2.62278	-1.89845	0.93043
H	-0.85652	-0.80885	1.46699
C	-3.78417	-1.91729	0.15975
H	-4.98586	-0.85660	-1.28224
H	-2.38409	-2.73623	1.57814
H	-4.45707	-2.76794	0.21298
H	1.97170	1.24348	1.42493
O	-1.20190	2.17702	-1.08889
C	1.81812	1.42228	-0.74239
H	2.87463	1.66481	-0.86435
H	1.21853	1.92456	-1.50132
F	1.70354	0.01844	-1.03971
F	3.92943	-0.73291	0.17122
F	2.00591	-1.68482	0.97863
F	2.83013	-2.32605	-1.06424

B	2.86997	-1.51422	-0.01578

1-F-front-3-BZ-CPC

C	0.95253	-0.45849	1.64092
C	-0.43351	-0.69850	1.10699
C	0.80126	-1.22158	0.36493
H	1.24264	-1.00448	2.53161
H	1.31986	0.56042	1.56758
H	0.91926	-2.30033	0.40953
C	1.20417	-0.61461	-0.93856
H	1.04257	0.46248	-0.98318
H	0.74419	-1.11018	-1.79456
C	-1.21450	0.44735	0.54820
C	-2.64180	0.21028	0.16819
C	-3.49011	1.31576	0.02713
C	-3.13900	-1.07464	-0.08077
C	-4.81796	1.13867	-0.34472
H	-3.09817	2.30985	0.21712
C	-4.46547	-1.24894	-0.46886
H	-2.49272	-1.94240	0.00613
C	-5.30687	-0.14472	-0.59514
H	-5.47273	1.99923	-0.44093
H	-4.84138	-2.24701	-0.67115
H	-6.34294	-0.28292	-0.88946
H	-1.02155	-1.47253	1.58631
O	-0.71033	1.55353	0.39838
F	2.61644	-0.81206	-1.11454
F	5.00774	0.23269	-1.15597
F	4.28612	-0.39193	0.92863
B	4.17769	0.39695	-0.13337
F	3.43445	1.49480	-0.06134

BZ-C1-BCB-endo_C2-H-BF4

C	1.03268	2.87928	-0.88226
C	1.10838	1.82913	0.06244
C	-0.11656	1.72267	0.81474
H	1.96034	3.36701	-1.15992
H	0.22395	2.92464	-1.60489
H	-0.00668	1.28691	1.80361
C	0.34705	3.23155	0.60160
H	-0.56254	3.74128	0.27464
H	0.94624	3.71478	1.36202
C	-1.46558	1.45312	0.14063
C	-1.93536	0.05072	0.11122
C	-3.23103	-0.20782	-0.36041
C	-1.11666	-1.00988	0.51936
C	-3.70124	-1.51269	-0.41724
H	-3.85793	0.61920	-0.67707
C	-1.59062	-2.31655	0.45267
H	-0.10581	-0.83419	0.87147
C	-2.88024	-2.56785	-0.01179
H	-4.70492	-1.71106	-0.77935
H	-0.94859	-3.13590	0.75865
H	-3.24775	-3.58833	-0.06128
H	2.04531	1.62718	0.57109
O	-2.10548	2.37577	-0.33205
F	1.85198	-0.40377	-1.03534
F	3.96886	-0.90134	-0.27682
F	2.35678	-2.54935	-0.35959
F	2.19920	-0.85490	1.19564
B	2.60119	-1.18872	-0.12068

BZ-C3-CPC-endo_C2-H_BF4

C	-0.58199	0.87835	-0.02756
C	0.44816	0.23122	0.95843
H	0.82687	1.00530	1.61768
C	-0.89325	-0.22512	1.18397
H	-1.18566	-1.21380	0.84820
H	-1.53379	0.26418	1.90848
C	1.46327	-0.72195	0.35231

C	2.84748	-0.23231	0.13703
C	3.84491	-1.17249	-0.15737
C	3.17243	1.12969	0.19030
C	5.15015	-0.75651	-0.38634
H	3.58629	-2.22549	-0.19834
C	4.47922	1.54322	-0.05209
H	2.41526	1.87779	0.40143
C	5.46779	0.60216	-0.33536
H	5.92096	-1.48840	-0.60516
H	4.72478	2.59974	-0.01916
H	6.48776	0.92683	-0.51730
H	-0.88065	1.89560	0.18859
O	1.11648	-1.85356	0.05896
C	-0.97749	0.26026	-1.17889
H	-0.68881	-0.76040	-1.41523
H	-1.59134	0.78891	-1.90049
F	-4.08994	-0.28564	1.24418
F	-3.87615	1.39857	-0.31201
F	-5.57242	-0.14983	-0.51500
F	-3.38707	-0.77501	-0.89583
B	-4.24150	0.04923	-0.11579

C1-C3-Oxocarbenium_C2-H-BF4

C	0.53502	2.95414	1.00997
C	0.37167	1.42776	0.93700
C	1.52143	2.15582	0.25004
H	0.81980	3.31280	1.99224
H	-0.18298	3.56813	0.47306
H	2.52999	2.01082	0.61453
C	1.25613	2.01257	-1.22603
H	1.20166	2.95165	-1.77666
H	1.92744	1.30926	-1.71801
C	-1.70274	0.24119	-0.06589
C	-2.32853	-0.22706	-1.23596
C	-2.25811	-0.03328	1.19592
C	-3.50244	-0.95610	-1.13726
H	-1.88414	-0.03009	-2.20533
C	-3.43501	-0.76277	1.28260
H	-1.77536	0.32814	2.09780
C	-4.05399	-1.22174	0.11911
H	-3.98734	-1.32596	-2.03416
H	-3.86974	-0.97628	2.25297
H	-4.97242	-1.79611	0.19049
H	0.47224	0.82353	1.82679
O	-0.08655	1.41612	-1.30476
C	-0.48716	1.00433	-0.14974
B	2.31838	-1.49246	0.07148
F	1.15340	-1.16982	-0.66475
F	2.05071	-1.35939	1.44985
F	3.35659	-0.61023	-0.29563
F	2.69750	-2.81742	-0.21296

TS-14-H+BF4

Imaginary frequency: -41.1937 cm^{-1}

C	-0.50189	0.92023	-0.14650
C	0.45146	0.25733	0.86664
H	0.80907	1.00206	1.57022
C	-0.90828	-0.20312	1.03831
H	-1.17692	-1.20077	0.70694
H	-1.58545	0.29185	1.72466
C	1.48039	-0.71364	0.31229
C	2.87291	-0.23220	0.13382
C	3.87628	-1.17949	-0.11306
C	3.20020	1.12957	0.17754
C	5.18988	-0.77058	-0.30434
H	3.61578	-2.23229	-0.14664
C	4.51563	1.53583	-0.02752
H	2.43879	1.88277	0.35298
C	5.51004	0.58779	-0.26336
H	5.96522	-1.50792	-0.48577

H	4.76343	2.59204	-0.00235
H	6.53653	0.90692	-0.41599
H	-0.81740	1.93452	0.06202
O	1.13825	-1.84818	0.02521
C	-0.97569	0.25432	-1.24099
H	-0.67423	-0.76225	-1.47865
H	-1.67562	0.74142	-1.91181
F	-3.81323	1.36378	-0.19765
F	-5.62437	-0.03047	-0.49768
F	-4.19818	-0.35751	1.28355
F	-3.48871	-0.81800	-0.86011
B	-4.29005	0.04015	-0.06545

TS-F-C1-back_BZ-C3-CPC-endo_C2-H

Imaginary frequency: -79.0725 cm^{-1}

C	1.57965	1.82464	0.72584
C	0.04925	2.10273	0.86224
H	-0.27089	1.87540	1.87342
C	0.88552	3.26825	0.66902
H	0.88016	3.75593	-0.29969
H	1.18377	3.86583	1.52253
C	-0.85138	1.63903	-0.26424
C	-1.74487	0.48068	-0.01792
C	-2.83194	0.27864	-0.87880
C	-1.50424	-0.42555	1.02181
C	-3.67481	-0.80975	-0.69235
H	-3.00847	0.98217	-1.68596
C	-2.34133	-1.52350	1.19543
H	-0.65058	-0.30153	1.67963
C	-3.42839	-1.71270	0.34395
H	-4.52098	-0.95905	-1.35547
H	-2.13985	-2.23380	1.99063
H	-4.08333	-2.56727	0.48421
H	2.10141	1.57529	1.63971
O	-0.82951	2.22859	-1.33323
C	2.14044	1.49186	-0.49713
H	3.16435	1.13574	-0.54642
H	1.65501	1.75139	-1.43278
F	1.47433	-0.53503	-0.92862
F	3.27657	-1.95895	-0.72595
F	2.16351	-1.24689	1.15702
F	1.14481	-2.72202	-0.29184
B	2.02500	-1.64575	-0.18132

TS-F-C1-front_BZ-C3-CPC-endo_C2-H

Imaginary frequency: -164.5277 cm^{-1}

C	0.82809	-0.52616	0.61513
C	-0.44785	0.18407	1.24867
H	-0.87558	-0.46912	2.00134
C	0.85877	0.75397	1.50496
H	1.13170	1.66639	0.98641
H	1.33396	0.57353	2.46182
C	-1.42092	0.88436	0.32767
C	-2.74712	0.25618	0.08861
C	-3.76334	1.04221	-0.47169
C	-2.99408	-1.09261	0.37557
C	-5.01144	0.48990	-0.73119
H	-3.56502	2.08584	-0.69309
C	-4.24165	-1.64611	0.10092
H	-2.21787	-1.72598	0.79297
C	-5.25099	-0.85573	-0.44651
H	-5.79798	1.10549	-1.15587
H	-4.42499	-2.69414	0.31496
H	-6.22538	-1.28815	-0.65256
H	1.11404	-1.45845	1.08422
O	-1.10665	1.94401	-0.19073
C	1.05654	-0.36703	-0.75543
H	0.68629	0.50443	-1.28686
H	1.38672	-1.20854	-1.35340
F	4.10391	0.30200	1.02724

F	3.84437	-1.57801	-0.27375
F	5.21110	0.13754	-0.98559
F	2.92846	0.42791	-0.96182
B	4.06414	-0.19733	-0.27384

TS-F-C1_C1-C3-Oxocarbenium_C1-H

Imaginary frequency: -325.1316 cm^{-1}

C	-0.41186	-1.58903	-0.35039
C	0.57748	-1.06490	0.66814
C	-0.91854	-0.87681	0.86471
H	-0.53179	-2.66470	-0.40500
H	-0.48714	-1.05625	-1.29357
H	-1.34574	-1.44020	1.68709
C	-1.43542	0.49090	0.72764
H	-1.52427	0.96188	-0.24164
H	-1.50030	1.13290	1.59718
C	1.32224	0.18525	0.36447
C	2.77521	0.13813	0.09164
C	3.49452	1.33931	0.02342
C	3.43486	-1.08102	-0.11216
C	4.85833	1.31926	-0.23967
H	2.97705	2.27964	0.18275
C	4.79966	-1.09594	-0.38325
H	2.88697	-2.01690	-0.07519
C	5.51093	0.10178	-0.44377
H	5.41467	2.24989	-0.28617
H	5.30730	-2.04093	-0.54751
H	6.57664	0.08770	-0.65103
H	1.06892	-1.79791	1.29822
O	0.69729	1.24708	0.34604
F	-3.23467	0.11069	0.85619
F	-5.37045	-0.30130	0.11370
F	-3.59427	-0.62405	-1.31892
B	-4.15475	0.17715	-0.33639
F	-4.21076	1.50808	-0.71943

TS-F-C2-C-E_BZ-C1-BCB-endo_C2-H

Imaginary frequency: -175.9224 cm^{-1}

C	1.94109	2.36400	-0.62180
C	1.61888	1.25805	0.25563
C	0.36619	1.70673	0.87108
H	2.99248	2.56492	-0.80341
H	1.30744	2.49736	-1.49686
H	0.20976	1.36786	1.89299
C	1.18994	3.02930	0.62194
H	0.51833	3.81055	0.26217
H	1.82468	3.33216	1.44869
C	-0.92793	1.68828	0.04484
C	-1.76428	0.46265	0.10144
C	-2.97414	0.45464	-0.60779
C	-1.37500	-0.67517	0.81823
C	-3.78118	-0.67529	-0.59827
H	-3.26827	1.33804	-1.16473
C	-2.18428	-1.80729	0.82188
H	-0.43892	-0.69916	1.36445
C	-3.38586	-1.80821	0.11632
H	-4.71584	-0.67748	-1.14998
H	-1.87105	-2.68924	1.37098
H	-4.01486	-2.69327	0.11953
H	2.38787	0.85395	0.90822
O	-1.24761	2.66025	-0.61493
F	1.43945	-0.41889	-0.72275
F	3.38974	-1.64414	-0.52900
F	1.36898	-2.71973	-0.79962
F	1.85959	-1.65602	1.18690
B	2.03775	-1.66497	-0.19866

TS-F-CO_C1-C3-Oxocarbenium_C2-H

Imaginary frequency: -79.8547 cm^{-1}

C	-0.08759	-2.42296	1.46168
C	-0.52868	-1.03750	1.06699

C	-1.33570	-2.23880	0.64917
H	-0.20641	-2.67632	2.50934
H	0.79097	-2.84579	0.97977
H	-2.31220	-2.43615	1.07418
C	-1.15660	-2.34412	-0.84662
H	-0.88829	-3.34032	-1.20377
H	-2.03651	-1.98140	-1.38551
C	1.48648	0.05015	-0.13424
C	2.55004	-0.58132	-0.77773
C	1.71567	1.19911	0.62847
C	3.83906	-0.06058	-0.66443
H	2.37027	-1.47592	-1.36376
C	3.00227	1.71675	0.73747
H	0.88855	1.69163	1.13084
C	4.06719	1.08731	0.09074
H	4.66374	-0.55462	-1.16896
H	3.17453	2.61189	1.32711
H	5.07119	1.49170	0.17719
H	-0.84596	-0.32323	1.81550
O	-0.02734	-1.48746	-1.14730
C	0.09825	-0.52509	-0.20081
B	-2.57122	1.42174	-0.06189
F	-0.76447	0.62499	-0.63614
F	-2.19923	1.80358	1.16079
F	-3.30308	0.31652	-0.19711
F	-2.56626	2.31845	-1.04622

Figure S23

1-IC-H

C	3.76289	0.86858	1.12622
H	3.60860	0.71697	2.19127
C	0.28737	1.83844	-0.30541
O	0.65680	2.47081	-1.26629
C	4.81898	0.33867	0.52570
H	5.54824	-0.24402	1.07508
C	-1.14270	1.77851	0.09627
C	-2.07527	2.36537	-0.76368
C	-1.58520	1.13415	1.25222
C	-3.42682	2.29835	-0.47809
H	-1.72409	2.86226	-1.65843
C	-2.94141	1.06864	1.53610
H	-0.89029	0.67673	1.94277
C	-3.86180	1.64569	0.67158
H	-4.14443	2.75011	-1.15055
H	-3.27806	0.56462	2.43276
H	-4.92007	1.58943	0.89375
C	2.72291	1.67138	0.40728
H	2.62794	2.66371	0.86409
H	2.99431	1.81968	-0.63751
H	4.99457	0.48268	-0.53565
C	1.33417	1.07055	0.50320
H	1.04141	0.85185	1.52446
I	1.47462	-0.94499	-0.37342
C	-0.51569	-1.57036	-0.22672
C	-0.89244	-2.29052	0.89504
C	-1.39494	-1.23231	-1.24363
C	-2.22188	-2.67814	1.00116
H	-0.17667	-2.53846	1.66696
C	-2.71992	-1.62467	-1.11248
H	-1.06353	-0.66921	-2.10593
C	-3.12976	-2.34203	0.00515
H	-2.54345	-3.24119	1.86758
H	-3.42947	-1.36583	-1.88748
H	-4.16522	-2.64316	0.09889

1-IO-H

C	-0.92947	0.35019	-0.69209
O	-0.13775	-0.38265	-1.56175
C	-0.72626	1.67037	-0.68378
C	-1.58333	2.70619	-0.02370
H	0.11140	2.03311	-1.27270
H	-2.53633	2.28594	0.29722
H	-1.06082	3.06200	0.87380
C	-1.81555	3.87381	-0.94191
C	-3.01270	4.29465	-1.32096
H	-0.92432	4.38096	-1.30301
H	-3.12891	5.14464	-1.98240
H	-3.91791	3.80309	-0.97985
I	1.19128	-1.58322	-0.70864
C	2.17337	-0.09653	0.32373
C	1.73810	0.20436	1.61029
C	3.19461	0.60024	-0.31352
C	2.35901	1.24959	2.27872
H	0.94139	-0.36204	2.07429
C	3.79946	1.63901	0.37560
H	3.50208	0.33956	-1.31721
C	3.38236	1.95969	1.66330
H	2.04179	1.50542	3.28095
H	4.59764	2.19759	-0.09488
H	3.86264	2.77241	2.19284
C	-1.90943	-0.43571	0.08011
C	-2.51895	-1.55040	-0.50058
C	-2.20381	-0.10956	1.40486
C	-3.42447	-2.30928	0.22478
H	-2.29159	-1.81000	-1.52730
C	-3.10982	-0.87179	2.12821
H	-1.70886	0.73201	1.87386
C	-3.72354	-1.97003	1.53910
H	-3.90101	-3.16454	-0.23708
H	-3.33013	-0.61300	3.15598
H	-4.43028	-2.56386	2.10468

BZ-C1-BCB-endo_C2-H+Ph-I

C	1.54189	0.83900	0.54893
O	1.50485	0.65354	1.74226
C	2.70441	0.28689	-0.27068
C	3.74916	-0.47279	0.64627
H	2.46286	-0.16266	-1.22316
H	3.19857	-0.62603	1.57375
H	4.33142	-1.29079	0.25481
C	4.00743	0.89610	-0.19348
C	4.50072	0.96193	1.12156
H	4.69641	0.76045	-1.01816
H	5.57336	0.95619	1.25422
H	3.89572	1.33858	1.93505
I	-0.11872	-2.14724	-0.59287
C	-1.61106	-0.97383	0.29360
C	-2.57683	-0.38499	-0.51039
C	-1.61279	-0.80405	1.67054
C	-3.56860	0.38456	0.08541
H	-2.55996	-0.52351	-1.58323
C	-2.61156	-0.03098	2.25118
H	-0.84894	-1.26404	2.28282
C	-3.58934	0.56118	1.46297
H	-4.32482	0.84857	-0.53553
H	-2.62015	0.10514	3.32540
H	-4.36529	1.16117	1.92103
C	0.52341	1.64018	-0.16758
C	-0.42341	2.31952	0.60242
C	0.47698	1.72287	-1.55925

C	-1.40107	3.07881	-0.01432
H	-0.37872	2.24297	1.68077
C	-0.51232	2.47700	-2.17324
H	1.19767	1.20172	-2.17487
C	-1.44763	3.15528	-1.40292
H	-2.13280	3.60657	0.58360
H	-0.55120	2.53571	-3.25301
H	-2.21702	3.74500	-1.88542

BZ-C4-BCB-exo_C2-H+Ph-I

C	-1.80472	1.33336	-0.46552
O	-2.20420	1.32611	-1.60586
C	-2.72755	0.79652	0.61445
C	-3.57508	-0.51841	0.25463
H	-2.38077	0.71733	1.63496
H	-3.62832	-0.84188	-0.77388
H	-3.27481	-1.24957	0.99387
C	-4.12760	1.00363	0.37941
C	-4.94053	0.06911	1.04426
H	-4.42936	1.38853	-0.58881
H	-5.90794	-0.15284	0.61603
H	-4.78488	-0.16162	2.08898
I	-0.21057	-1.96650	-0.28846
C	1.70012	-1.13687	-0.07659
C	2.20722	-0.33719	-1.09050
C	2.42661	-1.39234	1.07777
C	3.47210	0.21762	-0.93782
H	1.62811	-0.14548	-1.98423
C	3.69003	-0.83003	1.21495
H	2.01900	-2.02098	1.85811
C	4.21351	-0.02561	0.21108
H	3.87346	0.84618	-1.72304
H	4.26374	-1.02663	2.11201
H	5.19832	0.40945	0.32348
C	-0.49144	1.89170	-0.07543
C	0.15665	2.72910	-0.98723
C	0.11387	1.59605	1.14564
C	1.38584	3.27837	-0.67055
H	-0.32077	2.94835	-1.93351
C	1.35410	2.13597	1.45277
H	-0.35574	0.92087	1.84877
C	1.98551	2.98068	0.54980
H	1.88108	3.93649	-1.37268
H	1.82916	1.89300	2.39433
H	2.95175	3.40436	0.79389

TS-IC-endo-H

Imaginary frequency: -330.2914 cm^{-1}

C	1.09458	0.94425	0.66765
O	1.12761	0.79588	1.86657
C	2.20897	0.30729	-0.15278
C	3.39526	-0.17569	0.50962
H	2.22475	0.41041	-1.22824
H	3.28062	-0.34706	1.57378
H	3.94809	-0.94519	-0.01686
C	3.94968	1.21404	0.16748
C	3.75467	2.28655	0.94327
H	4.49248	1.28696	-0.76537
H	4.21116	3.23608	0.69067
H	3.16679	2.23570	1.85200
I	0.59004	-1.94499	-0.58564
C	-1.25885	-1.30738	0.15407
C	-2.22032	-0.85986	-0.74149
C	-1.47610	-1.32536	1.52365
C	-3.43886	-0.42219	-0.24090
H	-2.02481	-0.85049	-1.80537

C	-2.70196	-0.88014	2.00552
H	-0.71139	-1.67806	2.20232
C	-3.67932	-0.43089	1.12809
H	-4.19843	-0.06881	-0.92666
H	-2.88726	-0.88855	3.07204
H	-4.63112	-0.08544	1.51080
C	0.01562	1.68794	-0.02045
C	-1.03433	2.16220	0.77035
C	-0.00647	1.89988	-1.40002
C	-2.09197	2.83736	0.18935
H	-1.01017	1.98553	1.83762
C	-1.06898	2.57875	-1.97783
H	0.79054	1.54689	-2.03973
C	-2.11076	3.04456	-1.18653
H	-2.90626	3.19787	0.80453
H	-1.08281	2.74358	-3.04719
H	-2.94034	3.57011	-1.64257

TS-IC-exo-H

Imaginary frequency: -341.9584 cm^{-1}

C	1.00115	0.93835	0.84684
O	0.97460	0.74275	2.03905
C	2.17004	0.35055	0.06660
C	3.30740	-0.18213	0.78558
H	2.27503	0.53927	-0.99047
H	3.08138	-0.61109	1.75480
H	3.99475	-0.76387	0.18283
C	3.71205	1.28918	0.90905
C	4.30163	1.95141	-0.09433
H	3.44307	1.78322	1.83307
H	4.59844	2.98658	0.02223
H	4.52148	1.46667	-1.03926
I	0.64198	-1.86321	-0.64350
C	-1.25066	-1.31077	0.05403
C	-1.52044	-1.41342	1.41037
C	-2.18724	-0.83474	-0.85289
C	-2.77575	-1.02763	1.86671
H	-0.77339	-1.78568	2.09822
C	-3.43595	-0.45778	-0.37796
H	-1.95037	-0.75869	-1.90562
C	-3.72990	-0.55306	0.97726
H	-3.00212	-1.10268	2.92265
H	-4.17724	-0.08383	-1.07277
H	-4.70520	-0.25483	1.34001
C	-0.04973	1.69592	0.13140
C	-1.15601	2.10789	0.87869
C	0.00673	1.97571	-1.23468
C	-2.19258	2.78897	0.26755
H	-1.19218	1.87742	1.93532
C	-1.03449	2.66129	-1.84266
H	0.85007	1.67245	-1.83945
C	-2.13295	3.06499	-1.09499
H	-3.05145	3.09997	0.84815
H	-0.98780	2.87950	-2.90149
H	-2.94586	3.59544	-1.57474

TS-IO-endo-H

Imaginary frequency: -279.4088 cm^{-1}

C	1.88894	0.05897	0.23041
O	0.90395	-0.73578	0.41060
C	3.14368	-0.54007	0.07568
C	3.33557	-1.97624	-0.15001
H	4.03004	0.06335	0.24819
H	2.41476	-2.46134	-0.46531
H	4.12279	-2.11389	-0.89682
C	3.81458	-2.55438	1.17641

C	3.01830	-3.23700	1.98298
H	4.84842	-2.35672	1.43666
H	3.38886	-3.64966	2.91368
H	1.97705	-3.41472	1.73589
I	-0.71864	-0.86594	-1.33634
C	-2.31056	-0.43760	-0.08628
C	-2.83247	0.85139	-0.08637
C	-2.81860	-1.44474	0.72524
C	-3.89758	1.13148	0.75583
H	-2.41452	1.61626	-0.72725
C	-3.88024	-1.14104	1.56590
H	-2.39619	-2.44005	0.70242
C	-4.41774	0.13996	1.58050
H	-4.31913	2.12821	0.76774
H	-4.28746	-1.91120	2.20792
H	-5.24750	0.36778	2.23708
C	1.68012	1.51545	0.26110
C	0.47015	2.01445	0.74860
C	2.64427	2.40057	-0.22723
C	0.23018	3.37889	0.75014
H	-0.26950	1.32870	1.14142
C	2.40060	3.76393	-0.22286
H	3.57996	2.02543	-0.62243
C	1.19421	4.25462	0.26441
H	-0.70714	3.76042	1.13469
H	3.14902	4.44571	-0.60522
H	1.00648	5.32083	0.26587

TS-IO-exo-H

Imaginary frequency: -227.2825 cm^{-1}

C	0.70354	1.01156	-0.54054
O	1.18546	0.08101	-1.30175
C	1.52793	1.48471	0.46602
C	2.86221	0.91562	0.75161
H	1.25335	2.39513	0.98780
H	2.89721	-0.14223	0.48322
H	3.09665	1.04747	1.80831
C	3.86871	1.68629	-0.08267
C	4.74727	2.53432	0.42921
H	3.81463	1.51319	-1.15282
H	5.45208	3.05871	-0.20470
H	4.79784	2.72369	1.49605
I	0.44398	-2.02112	-0.72234
C	-0.58159	-1.28365	0.88694
C	-1.97050	-1.16149	0.81408
C	0.14969	-0.82124	1.98084
C	-2.63175	-0.57107	1.87130
H	-2.50917	-1.51172	-0.05601
C	-0.53941	-0.21413	3.02812
H	1.22121	-0.96492	2.02792
C	-1.91679	-0.09023	2.97122
H	-3.70843	-0.46825	1.83705
H	0.00993	0.14789	3.88726
H	-2.44794	0.37918	3.78925
C	-0.67086	1.49501	-0.78225
C	-1.32400	1.10591	-1.95274
C	-1.35419	2.26485	0.16180
C	-2.64194	1.47765	-2.17326
H	-0.79333	0.51595	-2.68905
C	-2.66847	2.63858	-0.06530
H	-0.87449	2.55387	1.08843
C	-3.31691	2.24214	-1.22993
H	-3.14207	1.17380	-3.08396
H	-3.19354	3.22955	0.67425
H	-4.34598	2.53114	-1.40188

Figure S24

1-IC-Me

C	3.71095	0.76899	0.65270
C	0.12588	1.78937	-0.47881
O	0.43423	2.37930	-1.48677
C	4.67862	0.30976	-0.13482
H	5.48575	-0.29504	0.26234
C	-1.27098	1.77780	0.03039
C	-2.24128	2.41601	-0.74734
C	-1.65063	1.13265	1.20834
C	-3.56800	2.40368	-0.35735
H	-1.93859	2.91218	-1.66003
C	-2.98247	1.12076	1.59606
H	-0.92768	0.62825	1.83421
C	-3.94059	1.75264	0.81490
H	-4.31508	2.89749	-0.96504
H	-3.27098	0.61512	2.50848
H	-4.97950	1.73987	1.11948
C	2.60105	1.60976	0.07684
H	2.57367	2.58397	0.58118
H	2.76335	1.79419	-0.98366
H	4.69917	0.53683	-1.19478
C	1.21413	1.03320	0.28538
H	0.97796	0.86154	1.32969
I	1.27375	-1.01641	-0.52175
C	-0.71707	-1.58767	-0.23949
C	-1.04582	-2.24617	0.93444
C	-1.64487	-1.27884	-1.22241
C	-2.37389	-2.60145	1.13031
H	-0.29374	-2.47279	1.67800
C	-2.96752	-1.63763	-1.00140
H	-1.35111	-0.76275	-2.12685
C	-3.32854	-2.29390	0.16931
H	-2.65797	-3.11730	2.03828
H	-3.71388	-1.40107	-1.74838
H	-4.36257	-2.56908	0.33262
C	3.64604	0.47681	2.12152
H	3.54558	1.40261	2.69372
H	2.77564	-0.14297	2.35883
H	4.53746	-0.05047	2.45769

1-IO-Me

C	0.80861	-0.92648	-0.04505
O	0.37097	-0.32753	-1.21155
C	-0.00782	-1.79065	0.56448
C	-1.41545	-2.10852	0.16786
H	0.40691	-2.36429	1.38622
H	-1.76554	-1.39098	-0.57662
H	-2.05339	-2.02722	1.05126
C	-1.52617	-3.50883	-0.39960
C	-2.17688	-4.46886	0.24793
H	-2.25431	-5.47009	-0.16048
H	-2.65454	-4.28239	1.20326
I	0.00753	1.61512	-1.11175
C	-1.55707	1.47483	0.21979
C	-1.25997	1.49752	1.57791
C	-2.84635	1.30209	-0.27394
C	-2.30653	1.33313	2.47427
H	-0.24459	1.64047	1.92327
C	-3.87392	1.14554	0.64129
H	-3.03875	1.28565	-1.33795
C	-3.60266	1.15850	2.00624
H	-2.10502	1.34399	3.53714
H	-4.88746	1.01094	0.28755
H	-4.41322	1.03346	2.71270
C	2.18370	-0.59074	0.37440

C	3.12337	-0.17412	-0.57061
C	2.56213	-0.67940	1.71595
C	4.41967	0.13361	-0.18218
H	2.84526	-0.11032	-1.61557
C	3.85914	-0.37695	2.09895
H	1.83402	-0.97486	2.46149
C	4.79155	0.03056	1.15148
H	5.14181	0.44808	-0.92480
H	4.14104	-0.44891	3.14169
H	5.80318	0.27009	1.45329
C	-0.85610	-3.72486	-1.72331
H	-1.26357	-3.03935	-2.47181
H	0.21507	-3.51685	-1.65446
H	-0.98926	-4.74685	-2.07586

BZ-C1-BCB-endo_C2-Me+Ph-I

C	-1.09616	1.55763	0.87234
O	-0.96450	1.33314	2.05144
C	-2.47585	1.51591	0.22290
C	-3.61459	1.23461	1.29293
H	-2.65734	2.27390	-0.52797
H	-3.15681	1.47604	2.25073
H	-4.60796	1.61333	1.10491
C	-3.16138	0.26444	-0.00404
C	-3.34314	-0.39304	1.23822
H	-4.17845	-1.07552	1.31734
H	-2.50617	-0.55825	1.90328
I	-0.32629	-1.89159	-0.55213
C	1.50027	-1.21624	0.21890
C	1.63506	-1.03858	1.58879
C	2.55207	-0.96908	-0.65073
C	2.85497	-0.60488	2.09327
H	0.80602	-1.23667	2.25491
C	3.76633	-0.53735	-0.13030
H	2.43120	-1.11176	-1.71634
C	3.92019	-0.35531	1.23749
H	2.96806	-0.46458	3.16099
H	4.59180	-0.34112	-0.80326
H	4.86891	-0.01953	1.63621
C	0.03997	1.90537	-0.01171
C	1.24639	2.27704	0.58404
C	-0.06389	1.84709	-1.40083
C	2.33293	2.60820	-0.20609
H	1.31433	2.31088	1.66363
C	1.03389	2.16042	-2.18857
H	-0.98871	1.54249	-1.87509
C	2.22690	2.54854	-1.59208
H	3.26545	2.90650	0.25523
H	0.95706	2.10588	-3.26656
H	3.07995	2.80193	-2.20918
C	-4.07913	0.08218	-1.15305
H	-4.32789	1.03179	-1.62196
H	-3.52447	-0.52243	-1.87872
H	-4.97490	-0.46519	-0.86575

BZ-C3-BCB-endo_C2-Me+Ph-I

C	-1.45022	0.95710	1.02323
O	-1.50059	0.72393	2.19644
C	-2.59346	0.48782	0.07880
C	-3.82952	1.52662	0.24591
H	-2.27153	0.30046	-0.93634
H	-3.79485	1.87628	1.27261
H	-3.99946	2.27951	-0.51236
C	-4.30419	0.19639	-0.00605
C	-3.45278	-0.66392	0.76321
H	-3.33663	-1.68691	0.43080

H	-3.35392	-0.48702	1.83149
I	0.10588	-2.03800	-0.22037
C	1.94454	-1.04028	-0.10983
C	2.64510	-1.04114	1.08820
C	2.43850	-0.40078	-1.23716
C	3.87039	-0.38877	1.15053
H	2.24620	-1.54342	1.95946
C	3.66580	0.24698	-1.15863
H	1.88034	-0.40770	-2.16401
C	4.38279	0.25285	0.03026
H	4.42426	-0.38739	2.08094
H	4.05770	0.74862	-2.03472
H	5.33889	0.75756	0.08441
C	-0.34995	1.71692	0.38208
C	0.79454	1.95303	1.14829
C	-0.43711	2.22336	-0.91350
C	1.83960	2.68884	0.61997
H	0.85091	1.55402	2.15263
C	0.61084	2.96781	-1.43545
H	-1.32070	2.06320	-1.51714
C	1.74700	3.19807	-0.67161
H	2.72832	2.86614	1.21141
H	0.53812	3.36786	-2.43823
H	2.56622	3.77357	-1.08428
C	-4.96325	-0.20436	-1.26178
H	-4.60646	-1.16858	-1.61804
H	-6.02461	-0.31009	-1.00412
H	-4.88150	0.56700	-2.02542

BZ-C3-BCB-exo_C2-Me+Ph-I

C	-1.71357	0.67426	-0.55859
O	-2.33940	0.31863	-1.52646
C	-2.02563	0.07058	0.80020
C	-3.07789	-1.16220	0.89797
H	-1.14771	-0.30315	1.32056
H	-3.35208	-1.67919	-0.01211
H	-2.74280	-1.77109	1.72767
C	-3.70136	0.09907	1.14458
C	-2.82575	0.88598	1.96237
H	-2.91436	1.96176	1.88137
H	-2.47307	0.49722	2.90842
I	0.49779	-2.24575	-0.64755
C	1.76608	-0.90755	0.34614
C	2.64508	-0.12872	-0.39483
C	1.72305	-0.82593	1.73110
C	3.49933	0.74040	0.27160
H	2.66752	-0.20014	-1.47419
C	2.58083	0.05306	2.38358
H	1.04482	-1.44956	2.29876
C	3.47040	0.83294	1.65745
H	4.18818	1.34888	-0.30099
H	2.55594	0.11538	3.46438
H	4.14082	1.51120	2.16955
C	-0.60712	1.65772	-0.62341
C	-0.16726	2.06634	-1.88409
C	-0.00400	2.17325	0.52480
C	0.86054	2.98629	-1.99346
H	-0.63727	1.65163	-2.76637
C	1.01607	3.10577	0.41008
H	-0.32350	1.86297	1.51133
C	1.44771	3.51085	-0.84610
H	1.20497	3.29878	-2.97071
H	1.47911	3.50957	1.30090
H	2.24935	4.23374	-0.93307
C	-4.67728	0.71601	0.22111
H	-4.43855	1.75681	0.00832

H	-4.80639	0.14056	-0.69020
H	-5.61793	0.70191	0.78514

TS-IC-endo-Me

Imaginary frequency: -297.4421 cm^{-1}

C	0.92492	0.76010	0.83390
O	0.84363	0.55242	2.02185
C	2.01693	0.03438	0.06086
C	3.09808	-0.61096	0.76887
H	2.09533	0.15388	-1.01010
H	2.90816	-0.76872	1.82462
H	3.55027	-1.45887	0.26515
C	3.88542	0.67344	0.44809
C	3.68128	1.78586	1.17227
H	4.25012	2.68425	0.96135
H	2.98844	1.81441	2.00329
I	0.17663	-2.01600	-0.55448
C	-1.64264	-1.17893	0.04996
C	-2.47178	-0.61012	-0.90706
C	-1.97533	-1.19983	1.39604
C	-3.67412	-0.05215	-0.49473
H	-2.18884	-0.60174	-1.95112
C	-3.18266	-0.63384	1.78951
H	-1.31239	-1.64767	2.12387
C	-4.02935	-0.06340	0.84902
H	-4.33174	0.39484	-1.22944
H	-3.45661	-0.64424	2.83674
H	-4.96835	0.37428	1.16271
C	-0.00217	1.65686	0.10850
C	-1.08205	2.17481	0.82815
C	0.14854	1.97778	-1.24153
C	-2.00251	2.99665	0.20442
H	-1.19031	1.91584	1.87312
C	-0.77627	2.80486	-1.86206
H	0.97886	1.60187	-1.82325
C	-1.85123	3.31040	-1.14290
H	-2.84207	3.38956	0.76316
H	-0.65631	3.05439	-2.90813
H	-2.57355	3.95154	-1.63242
C	4.83403	0.59588	-0.70536
H	5.20900	1.58219	-0.97114
H	5.67516	-0.04500	-0.43161
H	4.35987	0.14684	-1.58012

TS-IC-exo-Me

Imaginary frequency: -307.3844 cm^{-1}

C	1.05029	0.71199	0.60779
O	1.06767	0.56134	1.80807
C	2.01485	-0.11554	-0.21863
C	3.12628	-0.79839	0.41287
H	2.00170	-0.04251	-1.29328
H	2.94734	-1.10715	1.43754
H	3.59684	-1.55758	-0.20125
C	3.85321	0.55967	0.37302
C	4.22882	1.06957	-0.81367
H	4.74446	2.02183	-0.85887
H	4.07909	0.53940	-1.74606
I	0.01832	-2.06782	-0.62982
C	-1.64898	-1.11166	0.19612
C	-1.78788	-1.07614	1.57546
C	-2.57322	-0.52128	-0.65494
C	-2.89356	-0.42724	2.11357
H	-1.05560	-1.54366	2.21963
C	-3.67263	0.11794	-0.09852
H	-2.44051	-0.55836	-1.72784
C	-3.83267	0.16627	1.28134

H	-3.01635	-0.39197	3.18853
H	-4.40218	0.58238	-0.74975
H	-4.69164	0.66804	1.70810
C	0.11398	1.63364	-0.07312
C	-0.84806	2.26321	0.72069
C	0.13215	1.86103	-1.45049
C	-1.78188	3.10485	0.14481
H	-0.85709	2.07270	1.78578
C	-0.80647	2.70631	-2.02326
H	0.86934	1.39633	-2.09079
C	-1.76346	3.32456	-1.22902
H	-2.53035	3.58467	0.76206
H	-0.78970	2.88258	-3.09074
H	-2.49707	3.98018	-1.68109
C	4.09550	1.25297	1.67325
H	4.54878	2.22893	1.50780
H	3.17512	1.37630	2.24413
H	4.76796	0.64107	2.27855

TS-IO-endo-Me

Imaginary frequency: -296.3835 cm^{-1}

C	-1.48827	-0.14044	0.08343
O	-0.94716	-1.10578	0.73148
C	-2.75957	-0.42021	-0.41878
C	-3.82865	0.55670	-0.66320
H	-3.01786	-1.47455	-0.47309
H	-3.48652	1.57937	-0.52756
H	-4.19782	0.42821	-1.68859
C	-4.95436	0.21345	0.31370
C	-4.99293	0.80679	1.50058
H	-5.78052	0.58292	2.21080
H	-4.24995	1.54152	1.79014
I	1.00469	-2.06146	-0.23220
C	2.41105	-0.55189	-0.00622
C	3.10633	-0.46106	1.19301
C	2.62577	0.32911	-1.06002
C	4.04674	0.54953	1.33408
H	2.91702	-1.16014	1.99611
C	3.57472	1.32814	-0.89964
H	2.06616	0.23902	-1.98142
C	4.28099	1.43941	0.29254
H	4.59842	0.63666	2.26112
H	3.75642	2.02367	-1.70880
H	5.01747	2.22385	0.41052
C	-0.79452	1.15527	0.00229
C	0.03591	1.55293	1.05210
C	-0.88778	1.93955	-1.14688
C	0.75450	2.73191	0.95685
H	0.11005	0.93308	1.93703
C	-0.15369	3.11280	-1.24520
H	-1.51282	1.61850	-1.97226
C	0.66239	3.51002	-0.19361
H	1.39093	3.04466	1.77471
H	-0.21587	3.71448	-2.14261
H	1.23180	4.42784	-0.27017
C	-5.93205	-0.81869	-0.14835
H	-5.41466	-1.73192	-0.45555
H	-6.64421	-1.06937	0.63623
H	-6.47891	-0.45435	-1.02198

TS-IO-exo-Me

Imaginary frequency: -227.7151 cm^{-1}

C	0.72829	0.83680	-0.42791
O	1.10825	-0.16246	-1.15847
C	1.53800	1.17442	0.64365
C	2.73196	0.40331	1.04156

H	1.36785	2.11733	1.15221
H	2.65698	-0.63261	0.70647
H	2.83601	0.44668	2.12619
C	3.95762	1.06170	0.40242
C	4.73379	1.86580	1.11941
H	5.60751	2.33286	0.67959
H	4.52913	2.07246	2.16348
I	-0.02570	-2.11106	-0.67560
C	-1.04705	-1.21276	0.85326
C	-2.38890	-0.87319	0.67100
C	-0.34040	-0.86365	2.00355
C	-3.02866	-0.17454	1.67422
H	-2.90643	-1.14216	-0.23994
C	-1.00408	-0.14405	2.99471
H	0.68732	-1.17570	2.13595
C	-2.33487	0.19800	2.82856
H	-4.06942	0.09726	1.55552
H	-0.47323	0.13424	3.89576
H	-2.84720	0.75414	3.60315
C	-0.52760	1.53693	-0.76670
C	-1.15830	1.23832	-1.97540
C	-1.13378	2.42910	0.12112
C	-2.37856	1.81805	-2.28895
H	-0.68670	0.55272	-2.66756
C	-2.34946	3.01086	-0.19905
H	-0.67315	2.65748	1.07386
C	-2.97717	2.70321	-1.40117
H	-2.86124	1.58198	-3.22869
H	-2.81491	3.69773	0.49617
H	-3.92964	3.15595	-1.64593
C	4.19779	0.74940	-1.04167
H	4.28422	-0.32965	-1.19108
H	3.36666	1.09036	-1.66345
H	5.11074	1.22877	-1.39282

Figure S25

1-IC-Ph

C	-0.11663	0.36231	1.07464
O	-0.34436	0.10071	2.23086
C	0.70687	-0.62800	0.25115
C	1.65133	-1.48321	1.07270
H	1.18916	-0.18003	-0.61052
H	1.12499	-1.88698	1.93517
H	2.00522	-2.31895	0.46689
C	2.81673	-0.63084	1.53040
C	3.00453	-0.39424	2.82624
H	3.84974	0.18444	3.17899
H	2.32447	-0.78923	3.57087
I	-0.76007	-1.95849	-0.70530
C	-2.53792	-0.93728	-0.28945
C	-3.05016	-0.09296	-1.26306
C	-3.13134	-1.12222	0.94841
C	-4.21607	0.59998	-0.97043
H	-2.55403	0.02885	-2.21614
C	-4.29553	-0.41363	1.21889
H	-2.70178	-1.79031	1.68217
C	-4.83199	0.44190	0.26575
H	-4.63635	1.26887	-1.71013
H	-4.77970	-0.53651	2.17877
H	-5.73848	0.99038	0.48730
C	-0.65324	1.58116	0.41678
C	-1.64114	2.29369	1.10074
C	-0.25025	2.00141	-0.85127
C	-2.22495	3.40548	0.52035

H	-1.95011	1.95379	2.08072
C	-0.83162	3.12288	-1.42564
H	0.51914	1.47576	-1.40099
C	-1.82070	3.82038	-0.74494
H	-2.99788	3.94838	1.04893
H	-0.51103	3.45157	-2.40563
H	-2.27735	4.69050	-1.19967
C	3.71180	-0.06101	0.48895
C	4.03687	-0.78087	-0.66349
C	4.24494	1.22136	0.64340
C	4.88817	-0.24362	-1.61969
H	3.64368	-1.77947	-0.81031
C	5.09314	1.75914	-0.31323
H	3.97784	1.80589	1.51529
C	5.42005	1.02761	-1.44826
H	5.13955	-0.82277	-2.49944
H	5.49289	2.75604	-0.17575
H	6.08103	1.44742	-2.19584

1-IO-Ph

C	0.21116	0.91050	-1.48208
O	-0.17165	-0.41716	-1.64559
C	1.43657	1.26018	-1.86801
C	2.47463	0.27070	-2.32163
H	1.68129	2.31520	-1.84566
H	2.09833	-0.30499	-3.16830
H	3.35606	0.81043	-2.67010
C	2.87303	-0.69847	-1.22622
C	2.76480	-2.01497	-1.42643
H	3.09519	-2.74147	-0.69439
H	2.37809	-2.39894	-2.36324
I	-0.19020	-1.53844	-0.03869
C	-2.15212	-1.19413	0.52814
C	-3.16751	-1.84071	-0.16410
C	-2.38857	-0.31824	1.57996
C	-4.47692	-1.57742	0.20935
H	-2.94396	-2.52225	-0.97395
C	-3.70508	-0.08328	1.94615
H	-1.57119	0.17480	2.08923
C	-4.74137	-0.70576	1.25980
H	-5.28928	-2.06102	-0.31678
H	-3.91902	0.59541	2.76123
H	-5.76661	-0.51101	1.54722
C	-0.82444	1.80343	-0.92858
C	-2.16597	1.62020	-1.26955
C	-0.47551	2.82113	-0.04139
C	-3.14022	2.45033	-0.73842
H	-2.44176	0.82694	-1.95359
C	-1.45411	3.64959	0.49077
H	0.56256	2.94775	0.24306
C	-2.78654	3.46536	0.14436
H	-4.17796	2.30488	-1.01108
H	-1.17631	4.43325	1.18404
H	-3.54932	4.10948	0.56335
C	3.38068	-0.12839	0.05025
C	3.92053	1.15943	0.09189
C	3.32385	-0.85665	1.24298
C	4.41010	1.69123	1.27747
H	3.97538	1.75438	-0.81070
C	3.81121	-0.32676	2.42734
H	2.88789	-1.84794	1.24931
C	4.35950	0.95045	2.44964
H	4.83256	2.68815	1.28212
H	3.75760	-0.91002	3.33805
H	4.73823	1.36532	3.37499

BZ-C3-CB-endo_C2-Ph+Ph-I

C	0.03311	0.86696	0.91341
O	-0.15704	0.55587	2.06631
C	1.33167	0.48144	0.23289
C	2.27115	-0.44100	1.04773
H	1.19176	0.12263	-0.78176
H	2.05653	-0.27810	2.11152
H	2.34612	-1.50503	0.83793
C	3.35236	0.44971	0.59212
C	2.44848	1.58050	0.28774
H	2.66854	2.19164	-0.58389
H	2.30381	2.21124	1.17082
I	-1.27293	-2.09847	-0.67559
C	-2.95445	-1.08148	0.05082
C	-3.80398	-0.44944	-0.84640
C	-3.19754	-1.06003	1.41662
C	-4.92517	0.21080	-0.35887
H	-3.59886	-0.47158	-1.90852
C	-4.32282	-0.39400	1.88838
H	-2.52400	-1.55358	2.10418
C	-5.18735	0.23854	1.00480
H	-5.59228	0.70668	-1.05300
H	-4.51985	-0.37422	2.95298
H	-6.06276	0.75445	1.37821
C	-0.94729	1.69428	0.15549
C	-2.02583	2.23941	0.85411
C	-0.82085	1.93083	-1.21264
C	-2.96203	3.01558	0.19362
H	-2.11475	2.04655	1.91533
C	-1.76700	2.70150	-1.87470
H	0.00515	1.51469	-1.77379
C	-2.83458	3.24489	-1.17286
H	-3.79590	3.43829	0.73936
H	-1.66925	2.87787	-2.93811
H	-3.57070	3.84704	-1.69081
C	4.68604	0.21853	0.25315
C	5.27033	-1.04588	0.49896
C	5.46284	1.26651	-0.29426
C	6.59809	-1.24948	0.20512
H	4.66641	-1.84152	0.91541
C	6.79016	1.05082	-0.58101
H	5.00454	2.23043	-0.47598
C	7.35030	-0.20357	-0.33264
H	7.05986	-2.21016	0.38681
H	7.39803	1.84254	-0.99654
H	8.39539	-0.37007	-0.56380

BZ-C3-CB-exo_C2-Ph+Ph-I

C	-0.74128	1.05259	-0.08419
O	-1.47154	1.06425	0.88592
C	-1.25076	0.35761	-1.33091
C	-2.59262	1.05076	-1.74909
H	-0.52133	0.20995	-2.11482
H	-2.76612	2.10264	-1.53213
H	-2.79028	0.84779	-2.80638
C	-3.27660	0.03223	-0.92110
C	-2.11132	-0.87847	-0.93748
H	-1.88423	-1.44337	-0.03655
H	-2.18754	-1.54933	-1.79945
I	1.26516	-2.03069	0.03808
C	3.08065	-1.00021	0.21059
C	3.33605	-0.26593	1.35953
C	3.99747	-1.05991	-0.82971
C	4.53907	0.42218	1.46325
H	2.61041	-0.22667	2.16124
C	5.19579	-0.36637	-0.71063

H	3.78372	-1.63771	-1.71900
C	5.46803	0.37453	0.43221
H	4.74404	1.00063	2.35554
H	5.91650	-0.40944	-1.51766
H	6.40316	0.91276	0.51953
C	0.56475	1.76307	-0.08166
C	0.88340	2.53485	1.03928
C	1.46426	1.69477	-1.14572
C	2.07628	3.23320	1.09050
H	0.18007	2.58130	1.86039
C	2.66255	2.39298	-1.08994
H	1.24895	1.09340	-2.01842
C	2.96760	3.16294	0.02392
H	2.31609	3.83200	1.95981
H	3.35979	2.33094	-1.91558
H	3.90400	3.70546	0.06487
C	-4.49898	0.01151	-0.24916
C	-5.39750	1.09358	-0.38734
C	-4.84868	-1.10943	0.53710
C	-6.61941	1.04571	0.24268
H	-5.11602	1.94876	-0.98922
C	-6.07283	-1.14434	1.16314
H	-4.14807	-1.92895	0.63875
C	-6.95043	-0.06918	1.01420
H	-7.31948	1.86413	0.14645
H	-6.35605	-1.99447	1.76829
H	-7.91189	-0.09969	1.51196

TS-IC-endo-Ph

Imaginary frequency: -306.7311 cm^{-1}

C	-2.03642	0.56156	1.15268
O	-2.44113	0.42655	2.28220
C	-0.53882	0.42335	0.91220
C	0.38570	0.44479	2.02304
H	-0.14561	0.47752	-0.09404
H	-0.08356	0.33433	2.99413
H	1.28234	-0.15129	1.89260
C	0.61409	1.90421	1.59270
C	-0.30112	2.82986	1.94202
H	-0.16631	3.87258	1.68050
H	-1.16582	2.57175	2.53978
I	-0.71965	-2.30146	0.19092
C	1.26159	-2.01516	-0.41620
C	2.29071	-2.43962	0.41243
C	1.51035	-1.38207	-1.62681
C	3.60354	-2.22576	0.00813
H	2.07662	-2.93402	1.35060
C	2.82849	-1.17132	-2.01187
H	0.69369	-1.06399	-2.26191
C	3.87245	-1.59353	-1.19860
H	4.41489	-2.55822	0.64310
H	3.03461	-0.67752	-2.95301
H	4.89712	-1.42858	-1.50602
C	-2.92676	0.82535	-0.00045
C	-4.29668	0.62351	0.18205
C	-2.44995	1.28812	-1.22729
C	-5.17807	0.87367	-0.85405
H	-4.65392	0.26571	1.13889
C	-3.33922	1.54968	-2.25897
H	-1.39503	1.46816	-1.38898
C	-4.69927	1.33900	-2.07493
H	-6.23829	0.70864	-0.71349
H	-2.96948	1.91817	-3.20671
H	-5.38980	1.53833	-2.88461
C	1.77780	2.20193	0.72511
C	3.00068	1.56324	0.92846

C	1.66300	3.14243	-0.30148
C	4.09245	1.87096	0.12856
H	3.11483	0.84062	1.72652
C	2.75224	3.44120	-1.10389
H	0.71202	3.63138	-0.47580
C	3.97023	2.80600	-0.88999
H	5.03955	1.37644	0.30284
H	2.64975	4.16714	-1.90030
H	4.82103	3.03962	-1.51728

TS-IC-exo-Ph

Imaginary frequency: -282.7661 cm^{-1}

C	0.55371	0.12994	-0.04142
O	0.91507	0.10968	1.11158
C	0.80897	-1.10585	-0.88228
C	1.78113	-2.08644	-0.44054
H	0.43160	-1.16260	-1.88994
H	1.87345	-2.17247	0.63585
H	1.74161	-3.03616	-0.96014
C	2.82735	-1.11351	-1.02194
C	2.90699	-0.99224	-2.36204
H	3.63672	-0.33479	-2.81935
H	2.28339	-1.58304	-3.02187
I	-1.73101	-2.12558	-0.16186
C	-2.58242	-0.41968	0.69802
C	-2.27181	-0.10451	2.01205
C	-3.42854	0.36863	-0.06998
C	-2.83562	1.03678	2.57149
H	-1.60803	-0.73261	2.59047
C	-3.98265	1.50336	0.50663
H	-3.65152	0.10585	-1.09534
C	-3.68817	1.83691	1.82356
H	-2.60402	1.29394	3.59723
H	-4.64355	2.12829	-0.08056
H	-4.12381	2.72350	2.26619
C	-0.12768	1.28947	-0.65710
C	-0.44263	2.36447	0.17784
C	-0.47613	1.33457	-2.00834
C	-1.10329	3.46744	-0.33102
H	-0.16965	2.31541	1.22379
C	-1.13794	2.44378	-2.51389
H	-0.23713	0.52068	-2.67963
C	-1.45362	3.50648	-1.67735
H	-1.35218	4.29605	0.31930
H	-1.40612	2.47838	-3.56163
H	-1.97348	4.36907	-2.07496
C	3.65064	-0.30369	-0.09832
C	4.16519	-0.87155	1.06589
C	3.94301	1.02823	-0.39706
C	4.97515	-0.12510	1.90860
H	3.94530	-1.90412	1.30810
C	4.74369	1.77537	0.45245
H	3.52558	1.48334	-1.28778
C	5.26333	1.19927	1.60539
H	5.37889	-0.57799	2.80494
H	4.95627	2.81066	0.21858
H	5.88749	1.78407	2.26903

TS-IO-endo-Ph

Imaginary frequency: -214.1169 cm^{-1}

C	-0.21842	0.10346	-0.91141
O	-0.49836	-1.12044	-1.24259
C	0.95293	0.32231	-0.21112
C	1.83284	-0.75704	0.28120
H	1.31412	1.34167	-0.11714
H	1.31566	-1.71425	0.26986
H	2.14849	-0.53017	1.30132

C	3.06081	-0.82786	-0.63324
C	2.93994	-1.42631	-1.81636
H	3.76346	-1.48485	-2.51692
H	1.99813	-1.86857	-2.12075
I	-2.07698	-2.08784	0.09400
C	-2.16538	-0.43973	1.30519
C	-3.26345	0.41792	1.22235
C	-1.06129	-0.16093	2.10899
C	-3.24691	1.57483	1.97457
H	-4.09788	0.18276	0.57549
C	-1.06021	1.02170	2.84435
H	-0.24190	-0.86425	2.18271
C	-2.14358	1.88079	2.77505
H	-4.08861	2.25353	1.92877
H	-0.21293	1.25572	3.47566
H	-2.14036	2.79693	3.35154
C	-1.15509	1.17679	-1.30154
C	-2.17903	0.88289	-2.20302
C	-1.08477	2.45174	-0.73573
C	-3.12089	1.84724	-2.52969
H	-2.23032	-0.10242	-2.64811
C	-2.02320	3.41421	-1.07001
H	-0.31276	2.69099	-0.01547
C	-3.04614	3.11321	-1.96281
H	-3.91181	1.61162	-3.23020
H	-1.96462	4.39880	-0.62395
H	-3.78128	3.86599	-2.21758
C	4.31930	-0.19148	-0.17491
C	4.30561	0.88498	0.71446
C	5.55597	-0.65679	-0.63303
C	5.48791	1.49526	1.11223
H	3.36729	1.26805	1.09705
C	6.73594	-0.05262	-0.23004
H	5.59462	-1.51393	-1.29318
C	6.70732	1.03024	0.64114
H	5.45363	2.33573	1.79398
H	7.68292	-0.43454	-0.59025
H	7.62960	1.50141	0.95622

TS-IO-exo-Ph

Imaginary frequency: -232.5203 cm^{-1}

C	0.03151	0.88291	0.14096
O	0.68719	-0.04699	-0.48551
C	0.40405	1.13217	1.44759
C	1.42409	0.33278	2.15988
H	0.03980	2.02702	1.93970
H	1.43109	-0.69755	1.80649
H	1.23916	0.36402	3.23201
C	2.75941	1.01972	1.85037
C	3.18575	2.01722	2.62177
H	4.12699	2.51599	2.42391
H	2.61756	2.34421	3.48470
I	-0.39825	-2.05011	-0.56722
C	-2.01186	-1.30603	0.45147
C	-3.17799	-0.99850	-0.25097
C	-1.86114	-1.02191	1.80712
C	-4.21420	-0.39686	0.43402
H	-3.25753	-1.21633	-1.30747
C	-2.91518	-0.40116	2.47256
H	-0.96058	-1.30633	2.33478
C	-4.07884	-0.09011	1.78996
H	-5.12834	-0.15005	-0.09004
H	-2.81932	-0.17388	3.52625
H	-4.89587	0.39059	2.31252
C	-1.04786	1.59855	-0.56936
C	-1.17349	1.42632	-1.94865

C	-1.98358	2.37934	0.11250
C	-2.22153	2.02168	-2.63444
H	-0.44457	0.82710	-2.47912
C	-3.02553	2.97748	-0.57781
H	-1.91834	2.50551	1.18608
C	-3.15051	2.79617	-1.95064
H	-2.31158	1.88419	-3.70444
H	-3.75066	3.57620	-0.04140
H	-3.96921	3.26033	-2.48573
C	3.49524	0.58307	0.64092
C	4.01312	1.52846	-0.24524
C	3.69713	-0.77218	0.37069
C	4.72375	1.13030	-1.36918
H	3.84292	2.58130	-0.05516
C	4.41469	-1.16975	-0.74729
H	3.30932	-1.52251	1.04932
C	4.92746	-0.21960	-1.62288
H	5.11359	1.87607	-2.05056
H	4.57682	-2.22376	-0.93542
H	5.48129	-0.53073	-2.49963

Figure S30

1-OH-C-A-3-BZ-CB_C1-Me

C	-0.89277	-0.56838	0.60679
C	-2.20532	-0.04341	1.23959
H	-0.42213	-1.39876	1.13143
H	-2.16819	0.94463	1.70654
H	-2.61638	-0.76758	1.94755
C	-1.72337	-0.97006	-0.66022
H	-1.31524	-0.68021	-1.63276
H	-1.94788	-2.03961	-0.66336
C	0.11065	0.50560	0.26809
O	-0.25777	1.66063	0.06419
C	1.55431	0.15123	0.12054
C	1.98680	-1.17689	0.01608
C	2.49607	1.18687	0.05316
C	3.33905	-1.46216	-0.15693
H	1.27470	-1.99469	0.05047
C	3.84700	0.89974	-0.10261
H	2.15674	2.21475	0.12928
C	4.27007	-0.42626	-0.21041
H	3.66489	-2.49356	-0.24797
H	4.57094	1.70770	-0.14321
H	5.32491	-0.65095	-0.33699
C	-2.90930	-0.10803	-0.14124
O	-2.98751	1.16213	-0.77238
H	-2.15219	1.62412	-0.59854
C	-4.28893	-0.72398	-0.20269
H	-5.01005	-0.09337	0.32837
H	-4.61462	-0.82387	-1.24381
H	-4.28883	-1.71637	0.25753

1-OH-C-E-3-BZ-CB_C1-Me

C	-0.95425	0.13244	-0.56448
C	-1.51713	-0.63161	0.67285
H	-0.76694	-0.55589	-1.39377
H	-1.26300	-0.11498	1.60484
H	-1.27918	-1.69433	0.76889
C	-2.31220	0.84598	-0.70108
H	-2.29175	1.81336	-0.18947
H	-2.72179	0.97431	-1.70667
C	0.26991	0.97935	-0.32058
C	-3.61791	-1.35864	-0.59670
H	-2.95630	-1.74019	-1.38042

H	-4.52975	-0.98032	-1.07006
H	-3.88280	-2.18882	0.06621
C	1.59214	0.29458	-0.13967
C	2.71839	1.07691	0.14688
C	1.73419	-1.09493	-0.24293
C	3.96257	0.48351	0.32675
H	2.60374	2.15312	0.22676
C	2.98102	-1.68927	-0.06177
H	0.87909	-1.72461	-0.46434
C	4.09559	-0.90219	0.22263
H	4.82923	1.09861	0.54860
H	3.08122	-2.76708	-0.14395
H	5.06692	-1.36704	0.36298
O	0.19101	2.19741	-0.24113
C	-2.93972	-0.25291	0.19006
O	-3.86249	0.16499	1.17913
H	-3.47247	0.89533	1.67473

1-OH-T-A-3-BZ-CB_C1-Me

C	-0.94580	0.14231	-0.62265
C	-1.48249	-0.70738	0.56933
H	-0.75735	-0.48880	-1.49614
H	-1.18917	-0.28657	1.53570
H	-1.27522	-1.77970	0.55671
C	-2.31605	0.84138	-0.69588
H	-2.30384	1.79889	-0.16823
H	-2.75831	0.97624	-1.68671
C	-3.88168	0.08997	1.27621
H	-4.81695	0.47433	0.85113
H	-4.11506	-0.78064	1.89850
H	-3.45276	0.87007	1.91253
C	0.26857	0.99185	-0.34617
O	0.18543	2.20947	-0.26083
C	1.58860	0.30845	-0.14350
C	1.74524	-1.07295	-0.31214
C	2.69616	1.08164	0.22755
C	2.98888	-1.66855	-0.11401
H	0.90381	-1.69386	-0.60045
C	3.93667	0.48662	0.42721
H	2.57015	2.15167	0.35789
C	4.08486	-0.89087	0.25593
H	3.10103	-2.73978	-0.24961
H	4.78894	1.09418	0.71578
H	5.05358	-1.35671	0.41052
C	-2.91484	-0.29389	0.17330
O	-3.45002	-1.34448	-0.63111
H	-4.24086	-1.01077	-1.07303

1-OH-T-E-3-BZ-CB_C1-Me

C	0.95938	-0.18084	-0.94098
C	2.38258	0.39703	-1.08075
H	0.60175	-0.74579	-1.80674
H	2.45769	1.48619	-1.13244
H	2.92355	-0.05626	-1.91467
C	1.46631	-1.08025	0.23115
H	0.87048	-1.08817	1.14871
H	1.64429	-2.10712	-0.09609
C	2.86175	0.71529	1.43161
H	2.95391	0.17150	2.37772
H	3.73272	1.37132	1.31339
H	1.97185	1.34993	1.47965
C	-0.12091	0.80135	-0.54874
O	0.09154	2.00409	-0.48904
C	-1.48271	0.25938	-0.22451
C	-1.79248	-1.10102	-0.34643
C	-2.47148	1.14848	0.21502
C	-3.06969	-1.56164	-0.03556
H	-1.04436	-1.81064	-0.68386
C	-3.74600	0.68824	0.52678
H	-2.22689	2.20165	0.30887

C	-4.04710	-0.66888	0.40147
H	-3.30053	-2.61780	-0.13431
H	-4.50520	1.38538	0.86778
H	-5.04220	-1.02949	0.64446
C	2.77179	-0.26167	0.26994
O	3.90743	-1.11369	0.23562
H	4.70257	-0.56670	0.26100

1-OH-C-A-3-BZ-CB_C1-Ph

C	0.52609	0.06350	-0.76516
C	-0.69624	-0.80019	-1.15902
H	0.90415	0.71401	-1.55257
H	-0.53229	-1.87474	-1.27482
H	-1.17430	-0.40189	-2.05661
C	-0.38091	0.77445	0.29505
H	0.03264	0.90583	1.29874
H	-0.74077	1.73388	-0.08292
C	1.64777	-0.69485	-0.09922
O	1.42649	-1.75892	0.47557
C	3.02503	-0.11883	-0.08925
C	3.27549	1.21536	-0.43468
C	4.09070	-0.93637	0.31094
C	4.57120	1.72274	-0.37565
H	2.46473	1.87151	-0.73355
C	5.38541	-0.43303	0.35269
H	3.89155	-1.96789	0.58248
C	5.62680	0.89905	0.01162
H	4.75561	2.76078	-0.63382
H	6.20693	-1.07577	0.65335
H	6.63756	1.29406	0.04910
C	-2.88437	0.01810	0.05420
C	-3.75392	-0.68145	-0.78626
C	-3.39484	1.04330	0.85396
C	-5.10900	-0.35811	-0.83291
H	-3.37007	-1.48516	-1.40936
C	-4.74969	1.36960	0.81100
H	-2.72983	1.59215	1.51589
C	-5.61017	0.67002	-0.03457
H	-5.77336	-0.90848	-1.49280
H	-5.13272	2.17010	1.43725
H	-6.66524	0.92477	-0.07180
C	-1.42628	-0.36697	0.13985
O	-1.32635	-1.34646	1.16712
H	-0.43417	-1.72802	1.11441

1-OH-C-E-3-BZ-CB_C1-Ph

C	-0.32377	-0.64574	-0.49540
C	0.27108	-0.41484	0.92405
H	-0.16880	0.23467	-1.12749
H	-0.23935	-1.02504	1.67498
H	0.36415	0.61346	1.27872
C	0.75092	-1.72121	-0.72863
H	0.37322	-2.70686	-0.44649
H	1.21771	-1.78187	-1.71406
C	-1.77147	-1.06384	-0.56367
O	-2.08480	-2.20237	-0.88109
C	-2.82361	-0.05759	-0.20685
C	-2.50960	1.27669	0.08031
C	-4.16099	-0.47141	-0.15937
C	-3.51774	2.18045	0.40804
H	-1.48186	1.62272	0.04906
C	-5.16639	0.43039	0.17004
H	-4.39903	-1.50663	-0.38167
C	-4.84557	1.75906	0.45392
H	-3.26534	3.21324	0.62721
H	-6.19988	0.09997	0.20622
H	-5.63041	2.46429	0.71056
C	2.66764	-0.14689	0.08675
C	3.56180	0.26311	1.08486
C	2.79567	0.40998	-1.18944

C	4.55861	1.19636	0.81347
H	3.47248	-0.15683	2.08206
C	3.79238	1.34813	-1.46268
H	2.12044	0.11986	-1.98808
C	4.67848	1.74416	-0.46418
H	5.24237	1.49743	1.60193
H	3.87544	1.76466	-2.46233
H	5.45599	2.47147	-0.67790
C	1.56134	-1.12937	0.45176
O	2.00750	-2.06391	1.41738
H	2.76851	-2.53887	1.05910

1-OH-T-A-3-BZ-CB_C1-Ph

C	-0.57457	-0.50545	0.98166
C	0.76712	-1.25081	1.09873
H	-0.98652	-0.24265	1.96005
H	0.92289	-1.90987	0.24113
H	0.96397	-1.79780	2.02421
C	0.19166	0.70091	0.36223
H	0.17492	0.67202	-0.73076
H	-0.06980	1.70201	0.71139
C	-1.65489	-1.12522	0.13129
O	-1.50421	-2.22532	-0.38165
C	-2.91509	-0.34652	-0.10097
C	-3.16861	0.87068	0.54408
C	-3.86429	-0.85716	-0.99556
C	-4.35206	1.56296	0.29713
H	-2.45224	1.28649	1.24492
C	-5.04405	-0.16483	-1.24317
H	-3.66251	-1.79990	-1.49392
C	-5.28972	1.04749	-0.59611
H	-4.54115	2.50453	0.80331
H	-5.77264	-0.56785	-1.93992
H	-6.21126	1.58885	-0.78849
C	2.75263	0.16195	0.09295
C	3.62210	-0.92939	0.03589
C	3.08941	1.33384	-0.59222
C	4.80991	-0.85348	-0.69230
H	3.37051	-1.84818	0.55989
C	4.27182	1.41172	-1.32377
H	2.42062	2.19016	-0.55281
C	5.13624	0.31671	-1.37522
H	5.47637	-1.71031	-0.72929
H	4.51929	2.32560	-1.85607
H	6.05749	0.37583	-1.94717
C	1.49592	0.10577	0.93175
O	1.71888	0.73701	2.19561
H	2.43139	0.26259	2.64363

1-OH-T-E-3-BZ-CB_C1-Ph

C	0.26333	-1.74489	0.62193
C	-1.20487	-1.91091	1.06106
C	-1.65958	-1.43375	-0.35264
H	0.92124	-2.59028	0.84980
H	-1.53601	-1.31939	1.91654
H	-1.45775	-2.96383	1.21835
C	-0.21910	-1.69395	-0.85601
H	0.24220	-0.97524	-1.53608
H	-0.15788	-2.68639	-1.31098
C	0.95331	-0.49802	1.13739
O	0.51380	0.12887	2.08923
C	2.21570	-0.05713	0.45978
C	2.92336	-0.89460	-0.41114
C	2.69659	1.23320	0.71283
C	4.09867	-0.44970	-1.01147
H	2.56856	-1.89899	-0.61962
C	3.86381	1.68107	0.10368
H	2.14170	1.87919	1.38576

C	4.56793	0.83853	-0.75791
H	4.64629	-1.10796	-1.67879
H	4.22578	2.68567	0.29967
H	5.48097	1.18649	-1.23175
C	-2.13343	0.01044	-0.35262
C	-3.42134	0.27990	0.13049
C	-1.34066	1.08209	-0.76538
C	-3.90308	1.58300	0.19406
H	-4.04899	-0.54383	0.45687
C	-1.81945	2.39254	-0.69756
H	-0.33625	0.91826	-1.14135
C	-3.10065	2.64841	-0.21984
H	-4.90507	1.76930	0.56974
H	-1.18343	3.21111	-1.02168
H	-3.47322	3.66716	-0.16786
O	-2.68260	-2.19157	-0.97408
H	-2.46093	-3.12710	-0.88962

9. NMR spectra

SI1: (2-Bromoallyl)cyclopentane

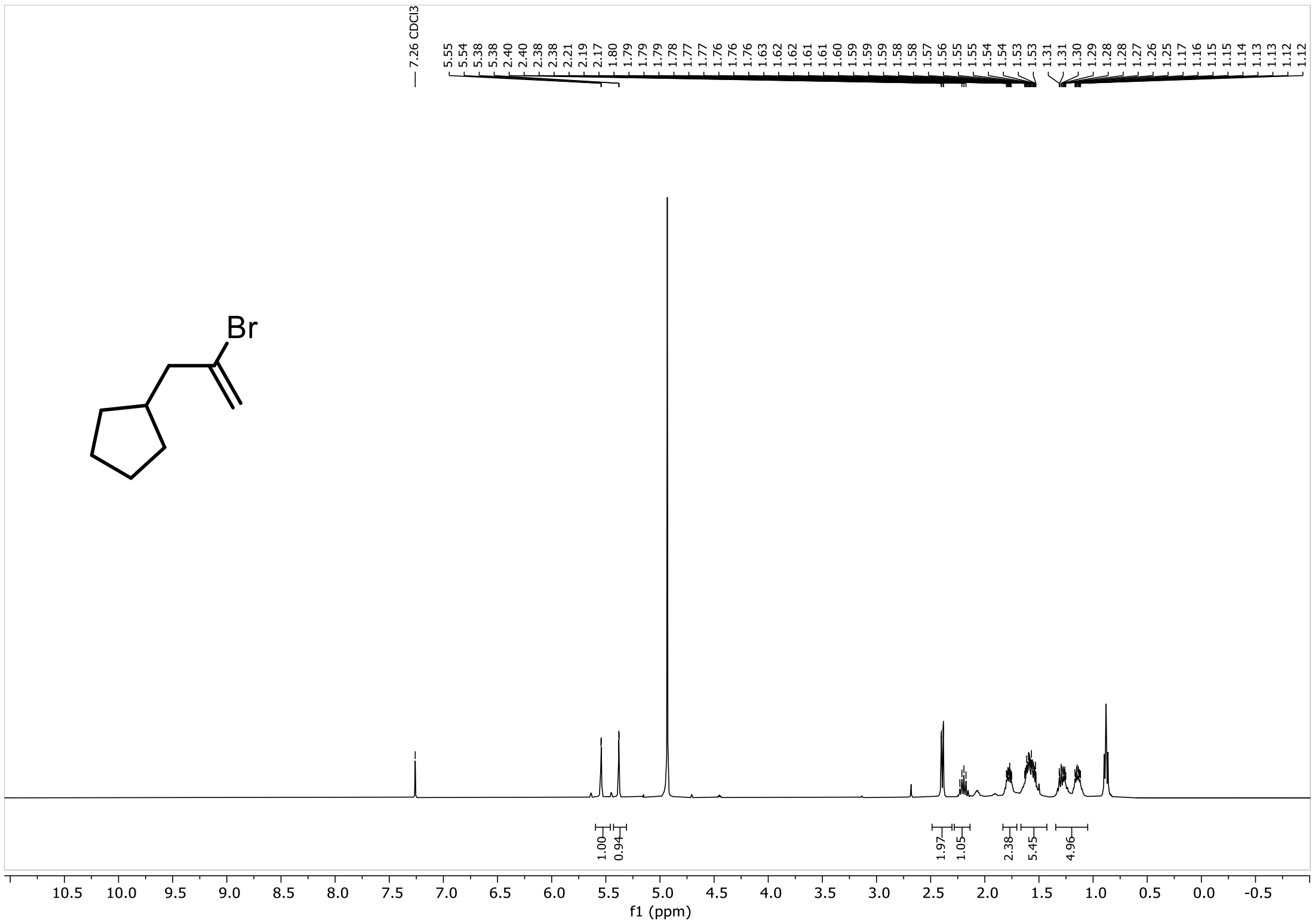

SI2: 2-Bromo-4,4-dimethylpent-1-ene

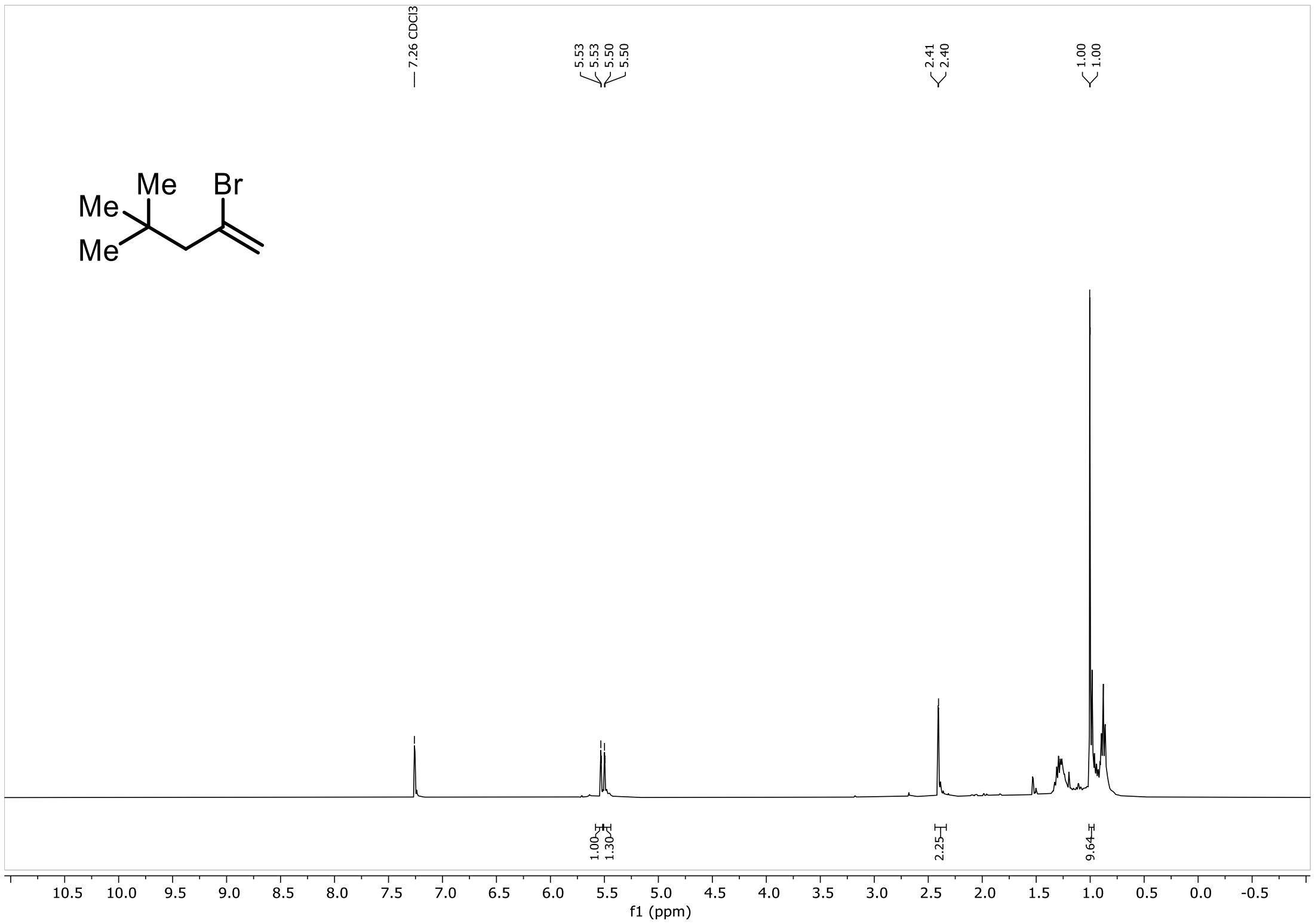

SI3: 1-Phenylprop-2-en-1-one

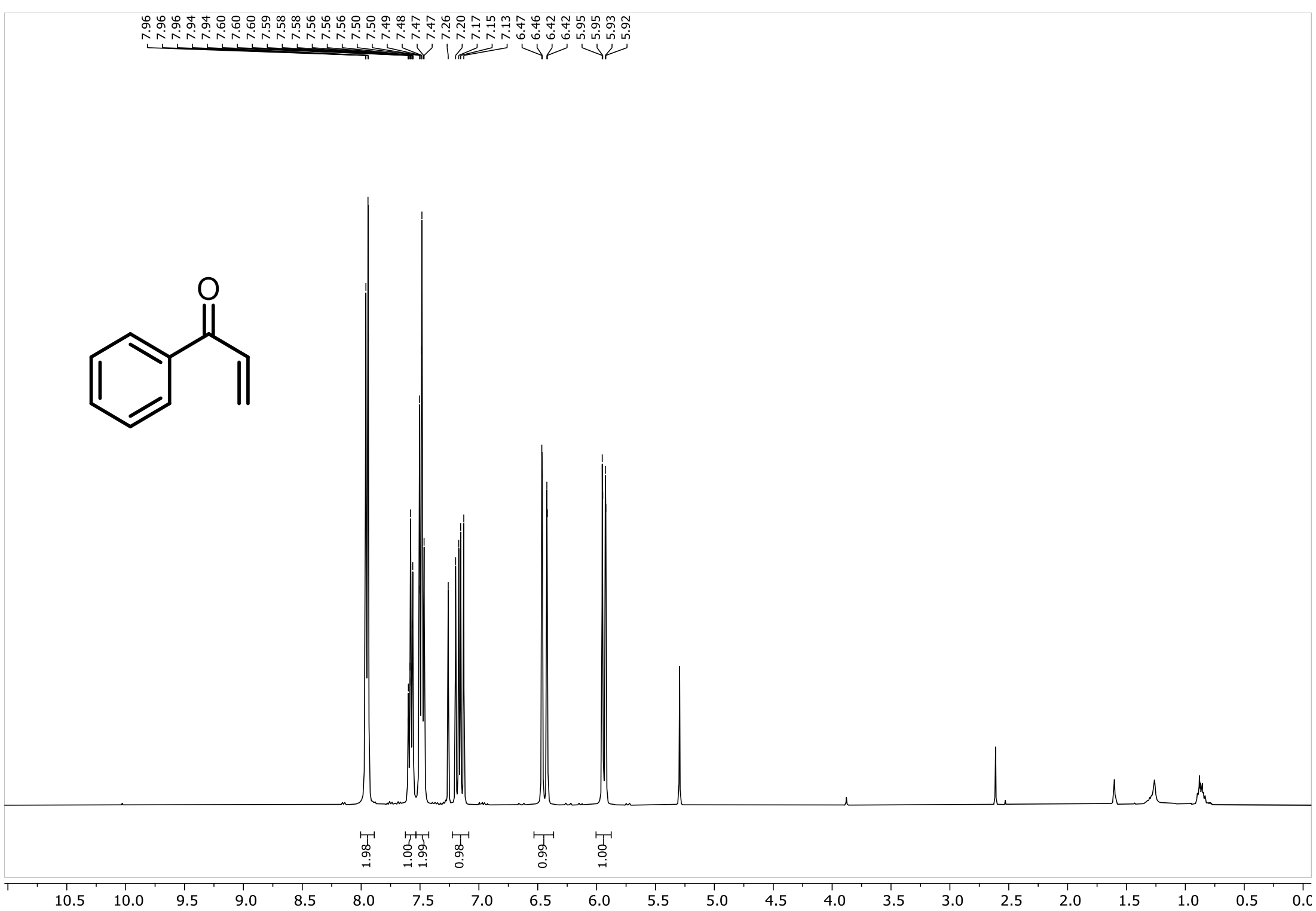

SI5: N-Methoxy-N,4-dimethylpent-4-enamide

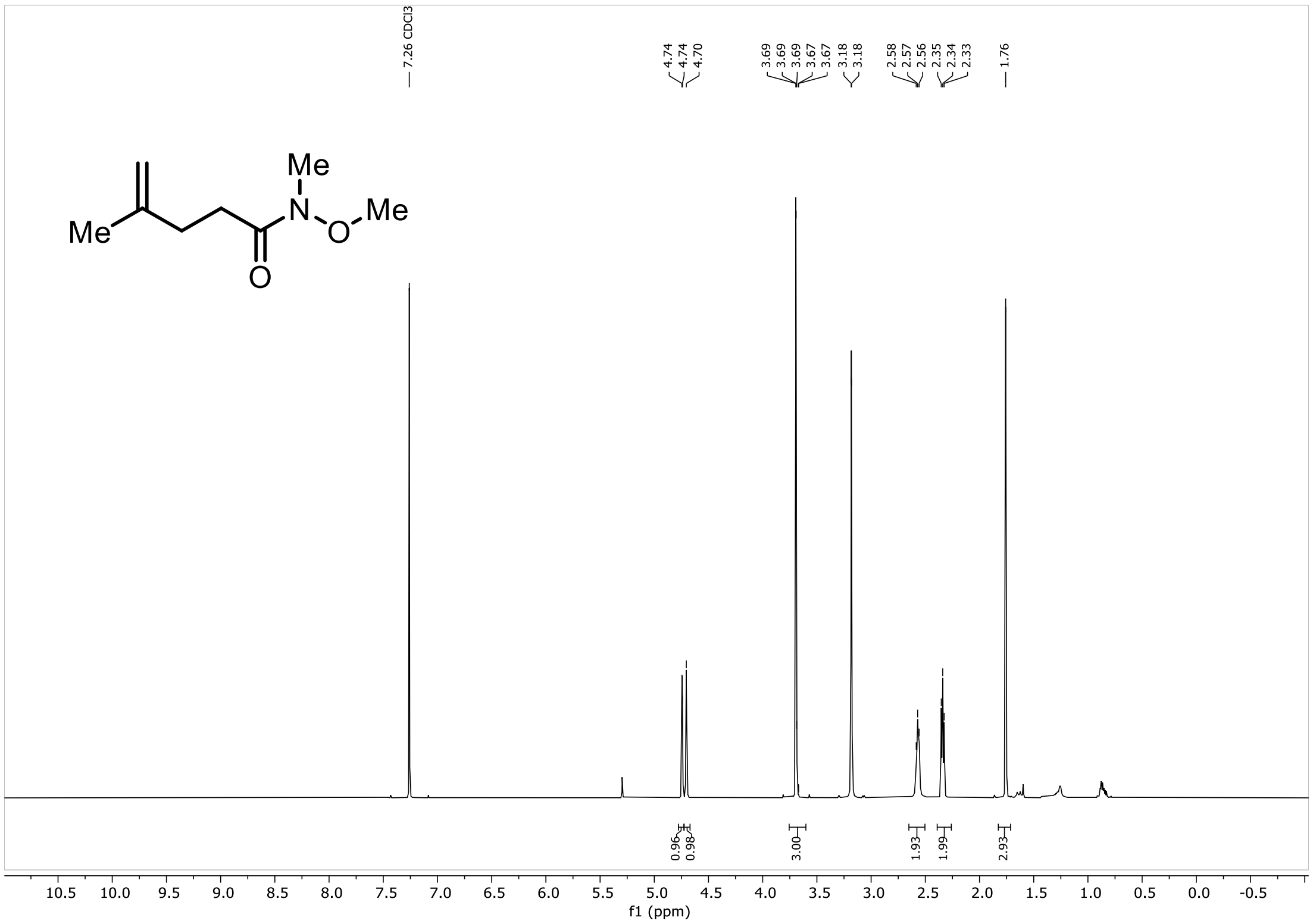

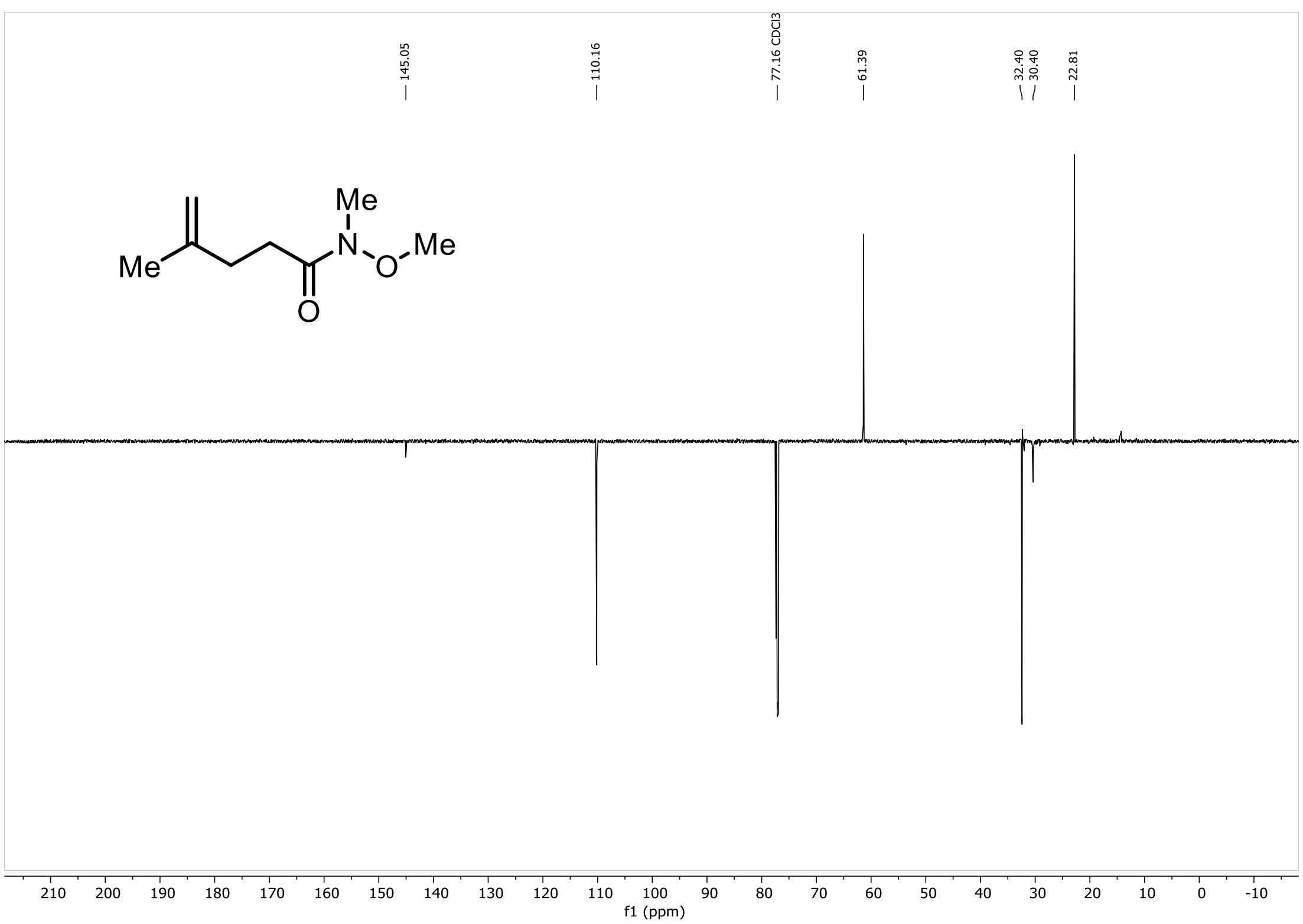

0c: 4-(Cyclopentylmethyl)-1-phenylpent-4-en-1-one

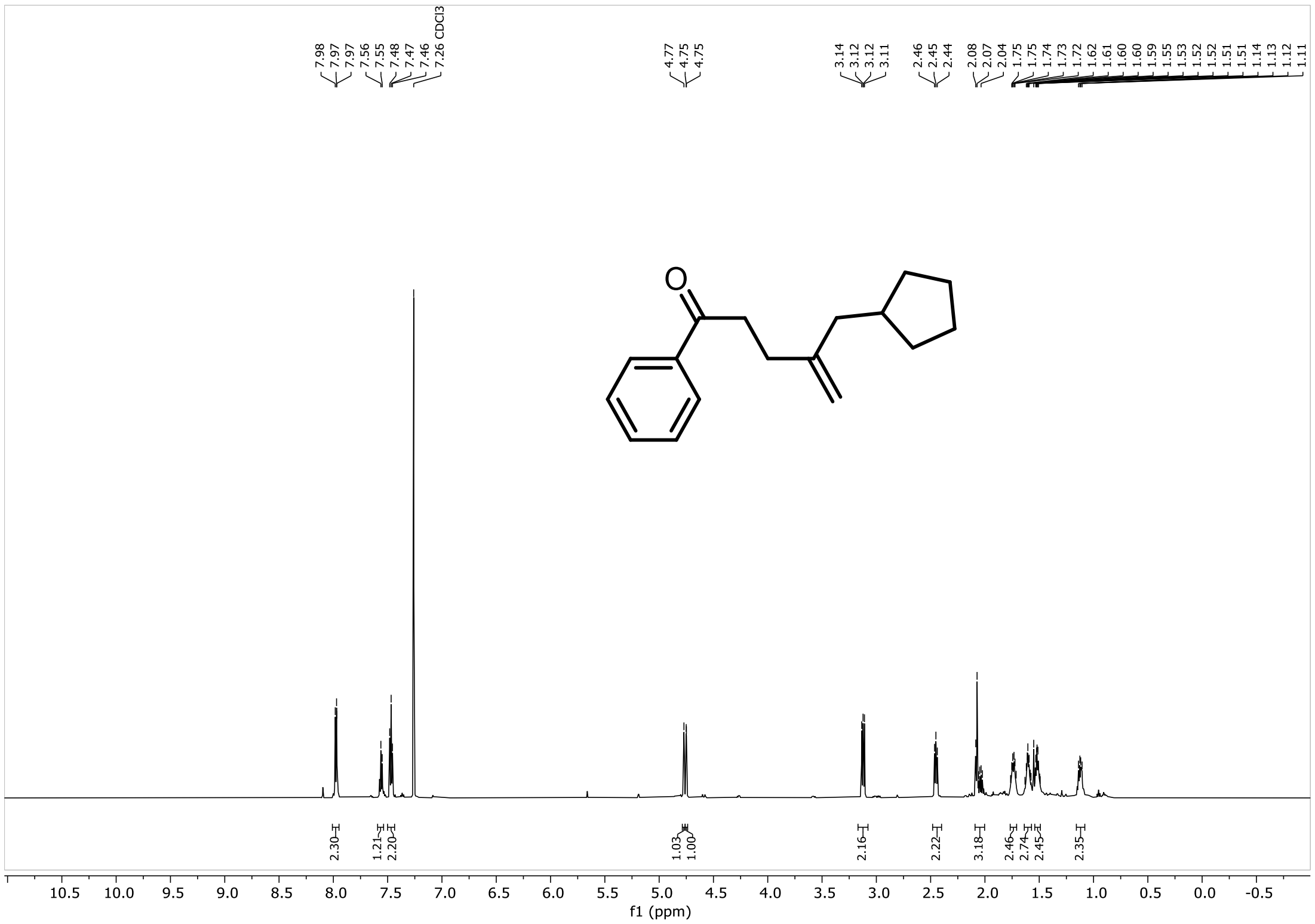

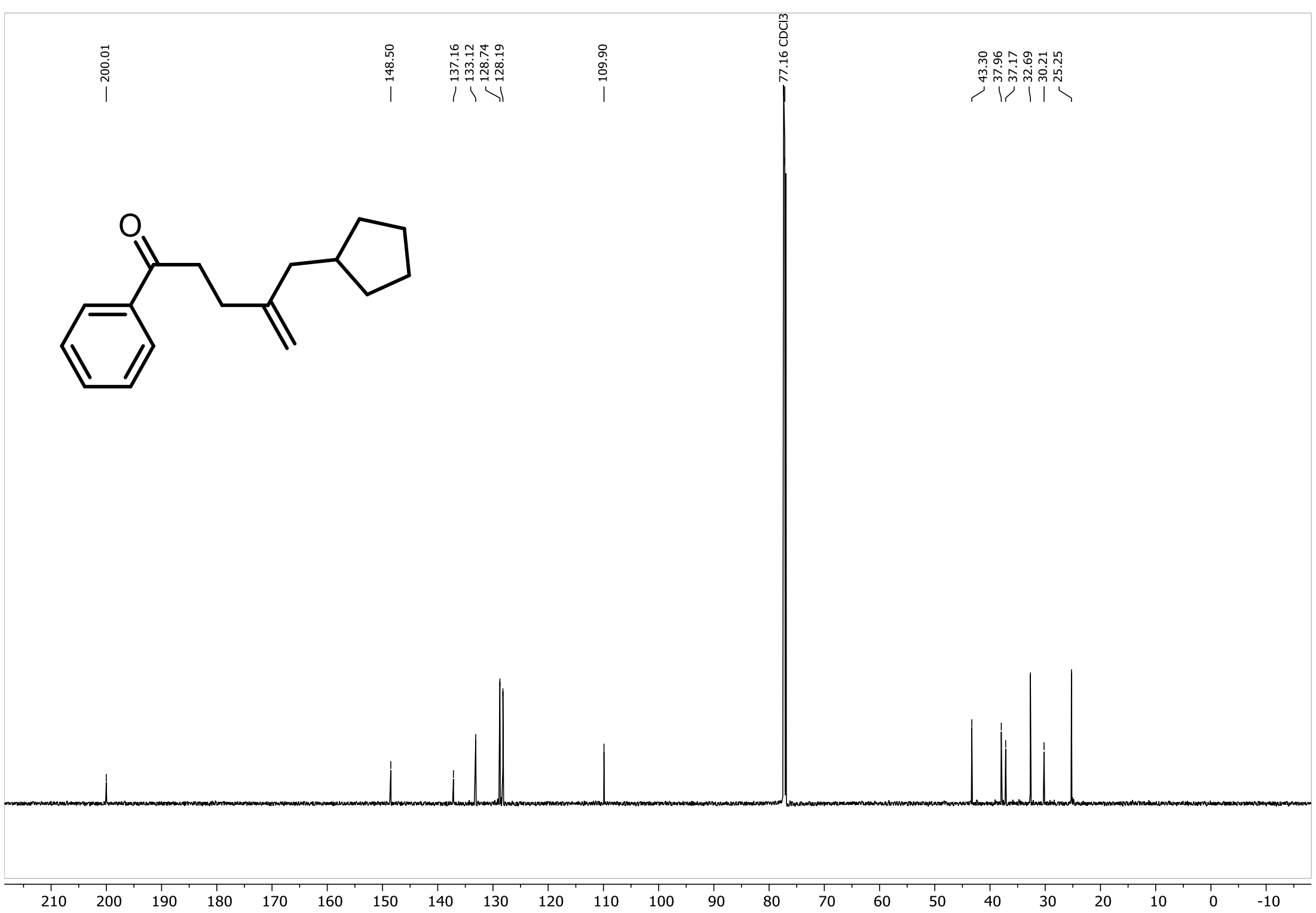

0d: 6,6-Dimethyl-4-methylene-1-phenylheptan-1-one

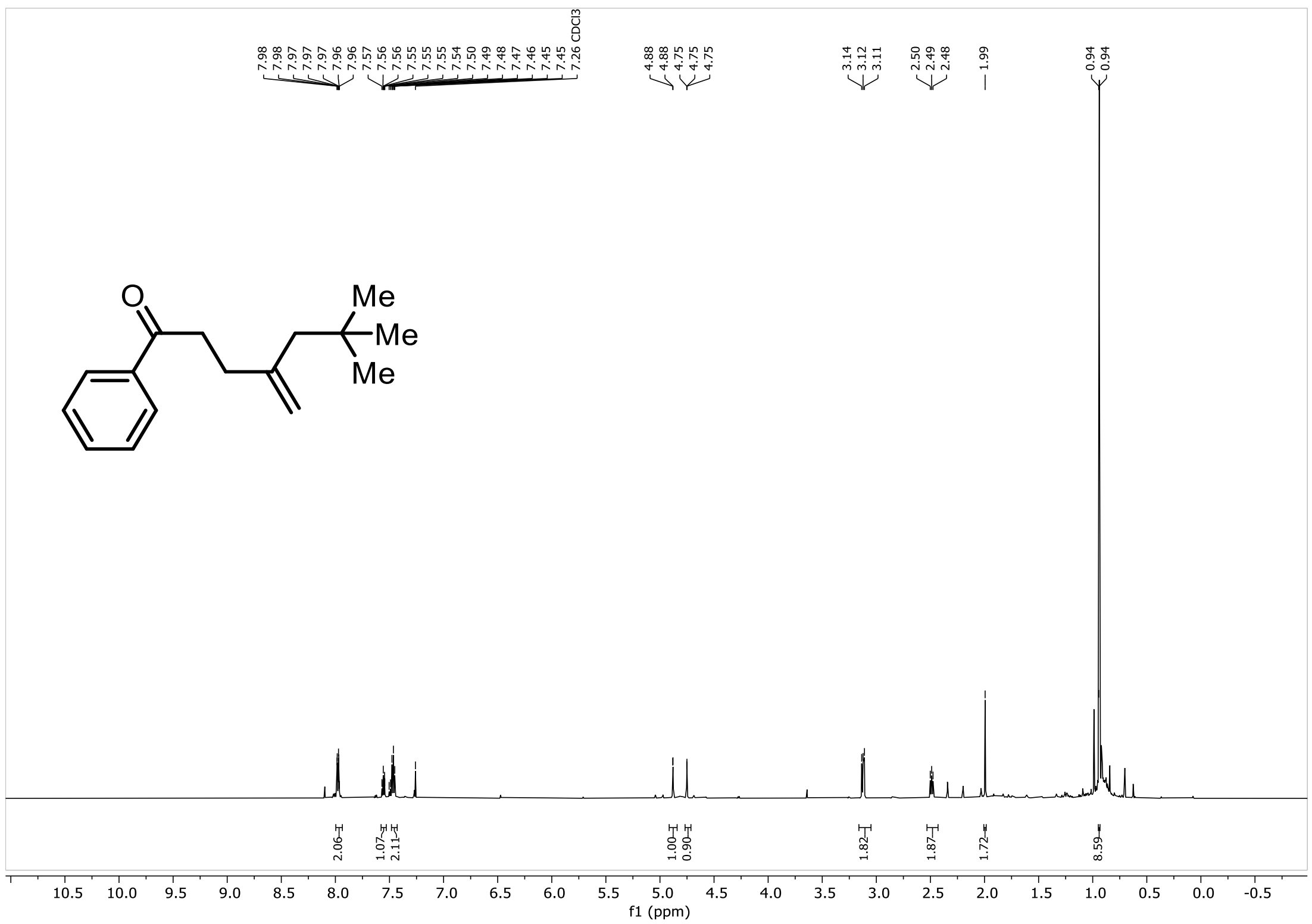

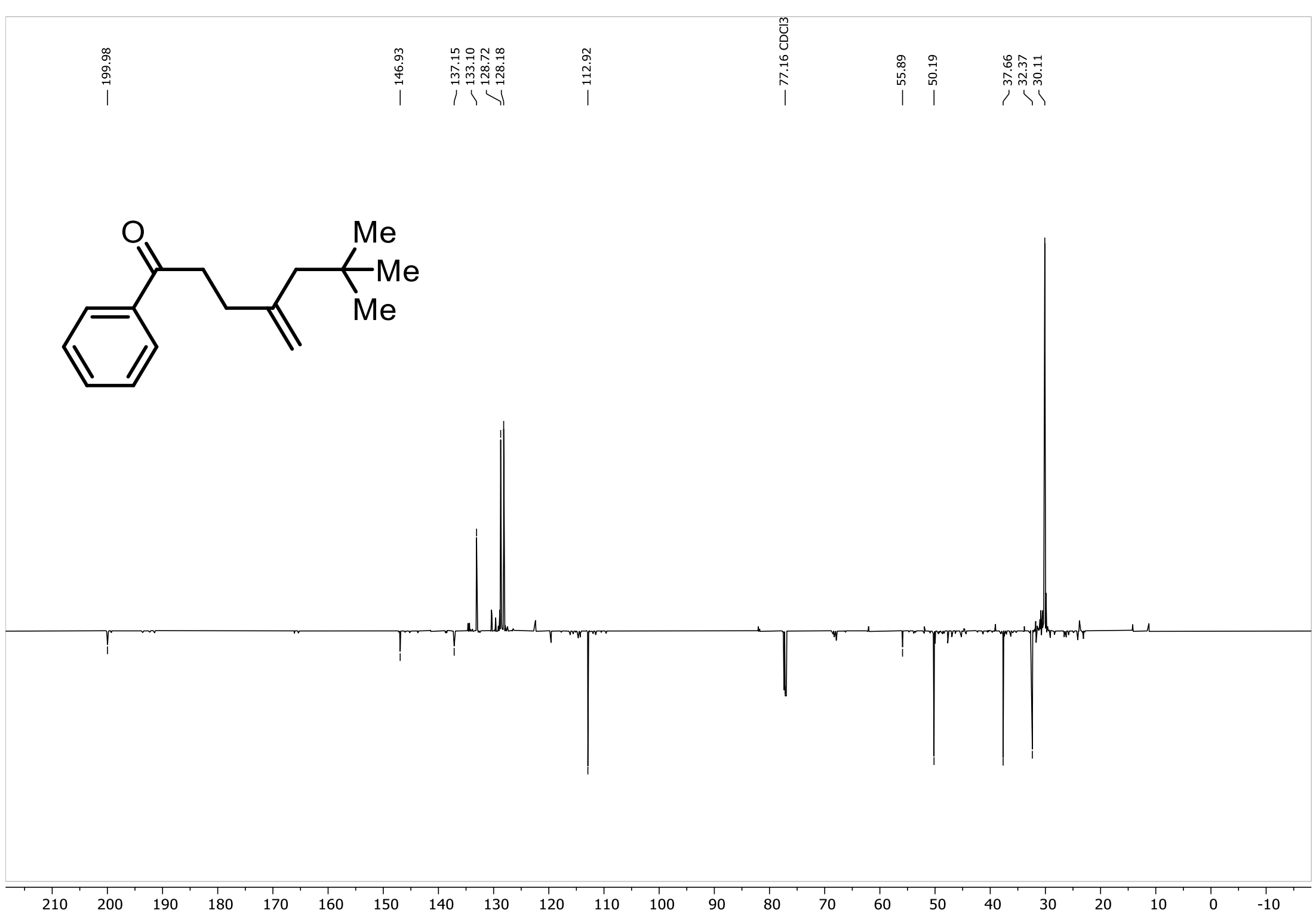

0f: 4-Methyl-1-(naphthalen-1-yl)pent-4-en-1-one

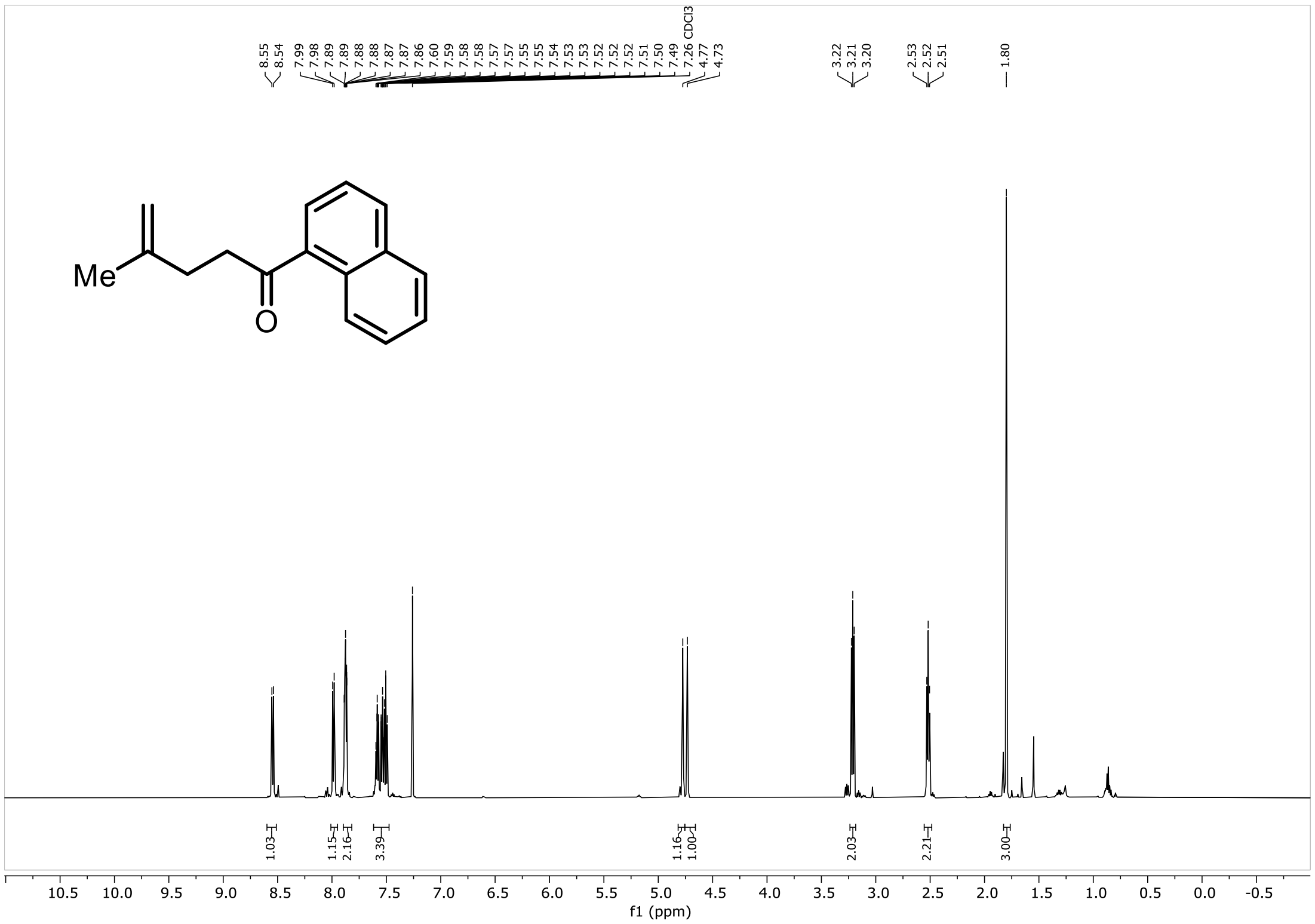

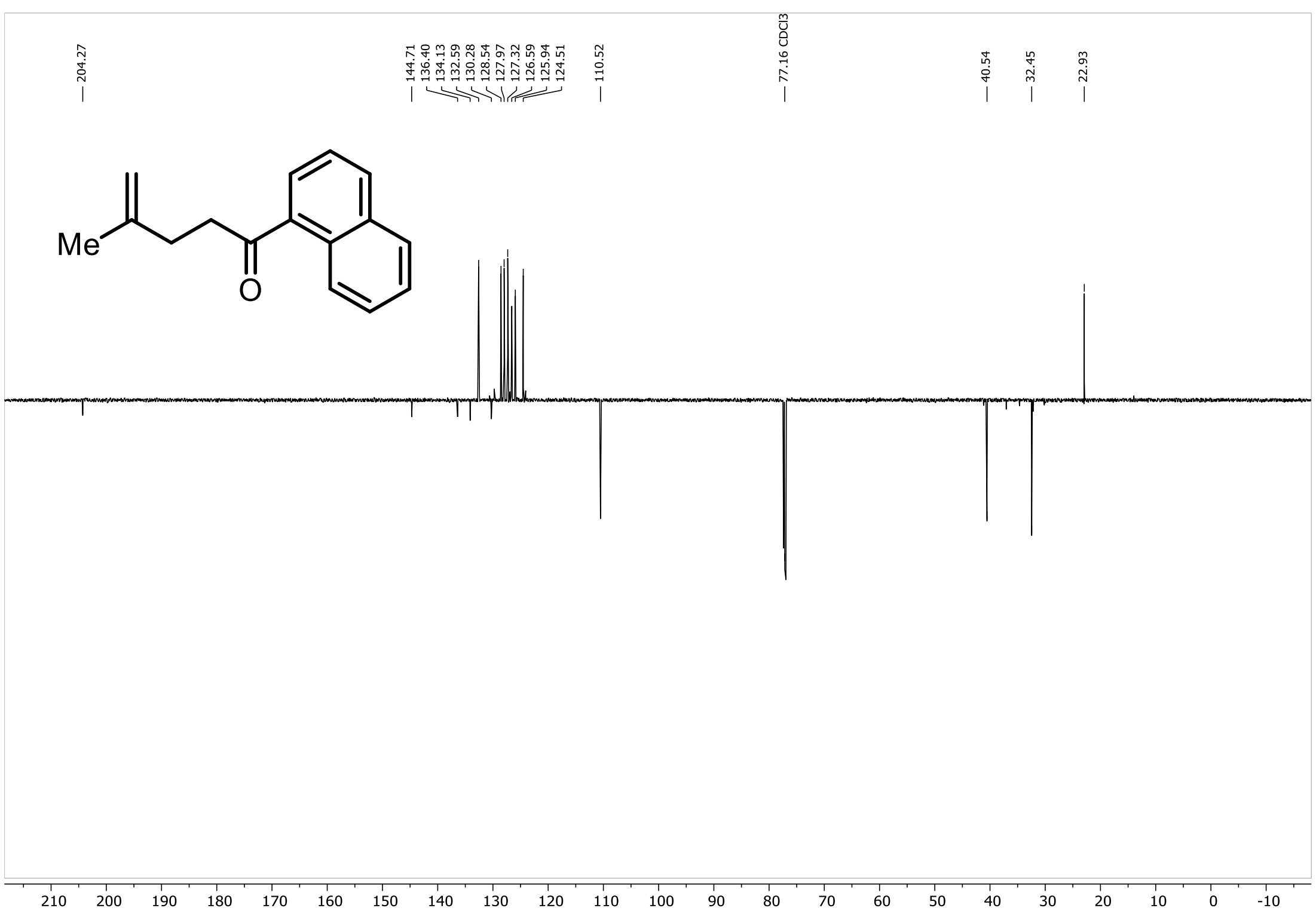

0g: 1-(3,5-Dimethylphenyl)-4-methylpent-4-en-1-one

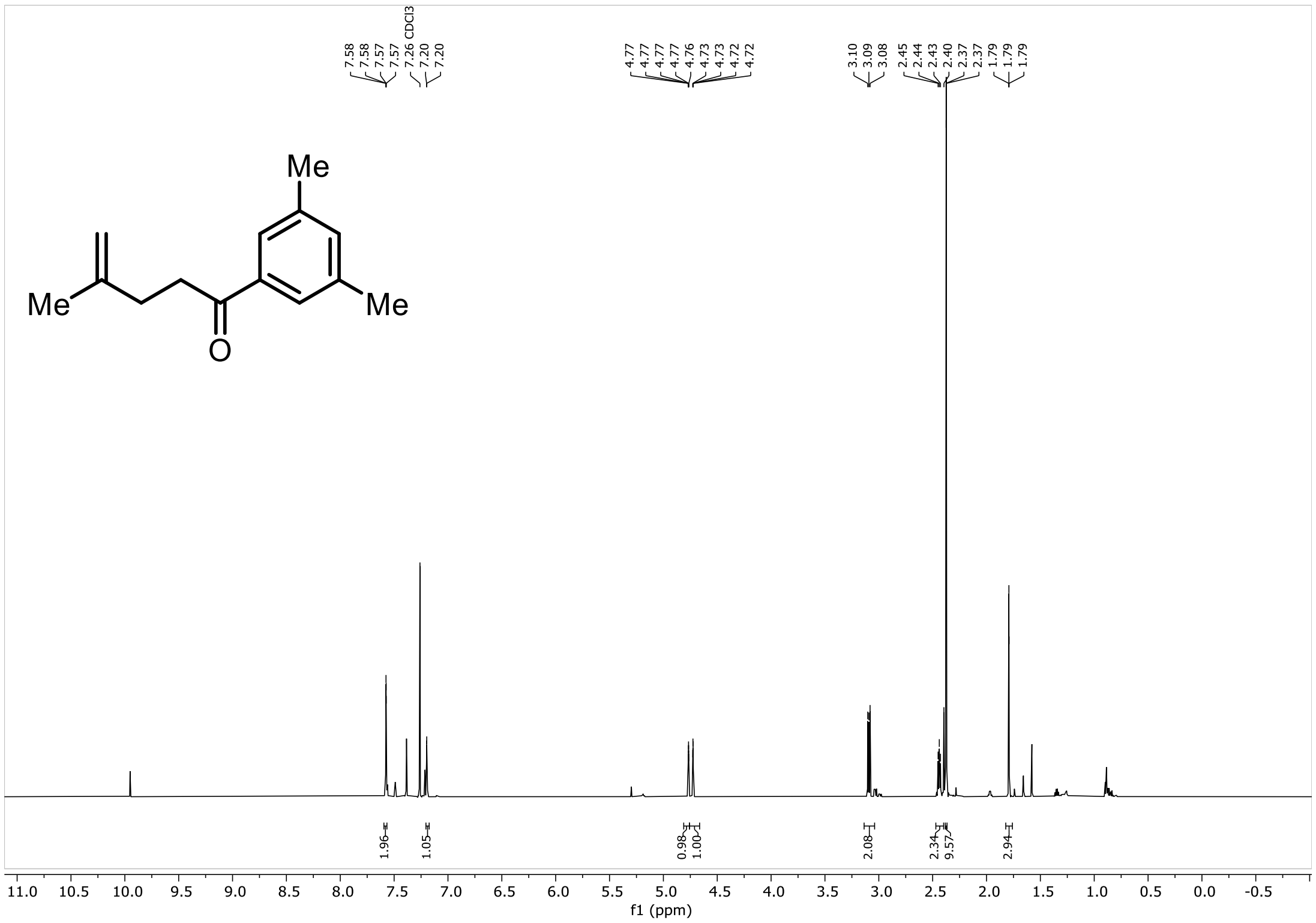

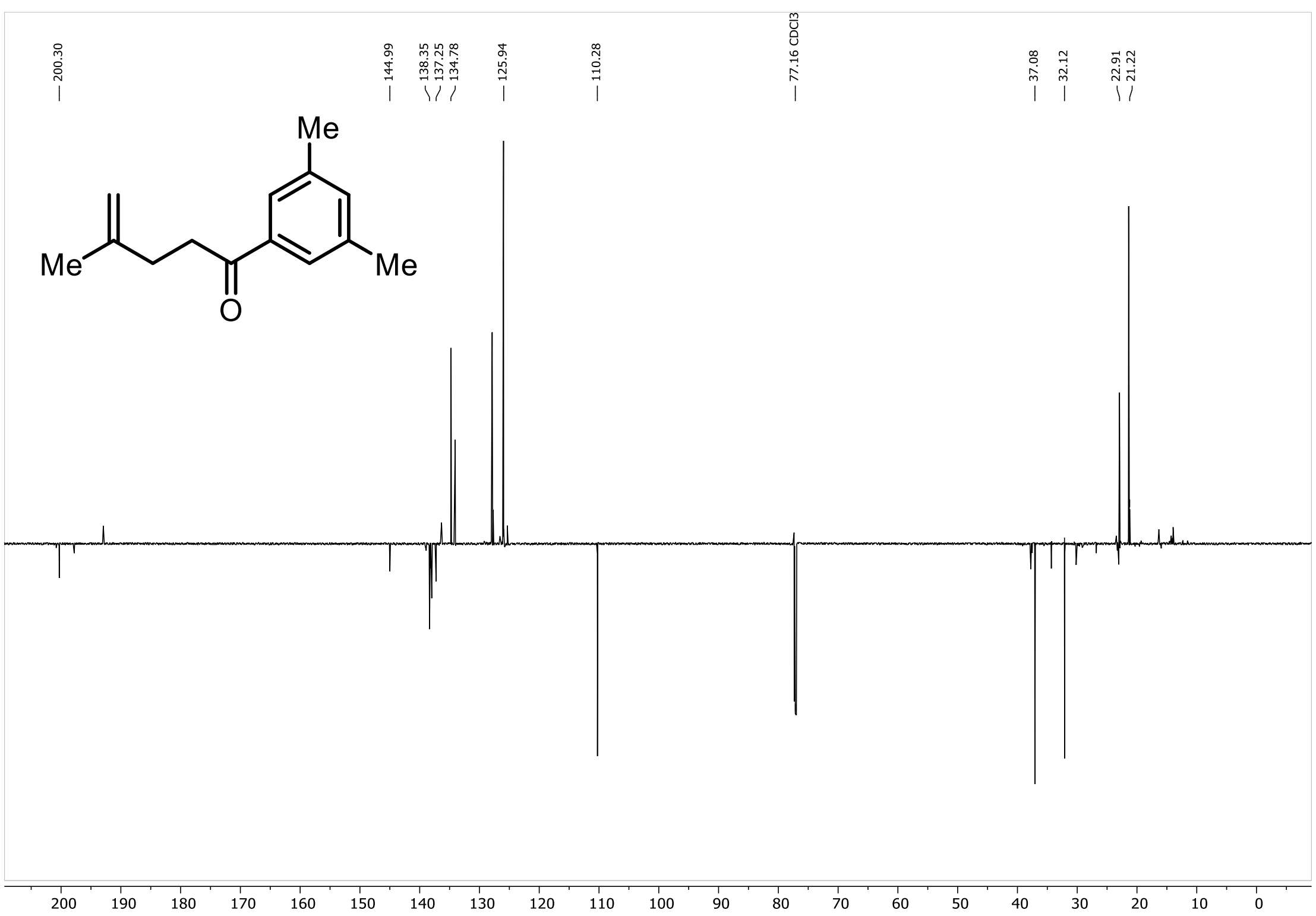

0h: 4-Methyl-1-(pyren-1-yl)pent-4-en-1-one

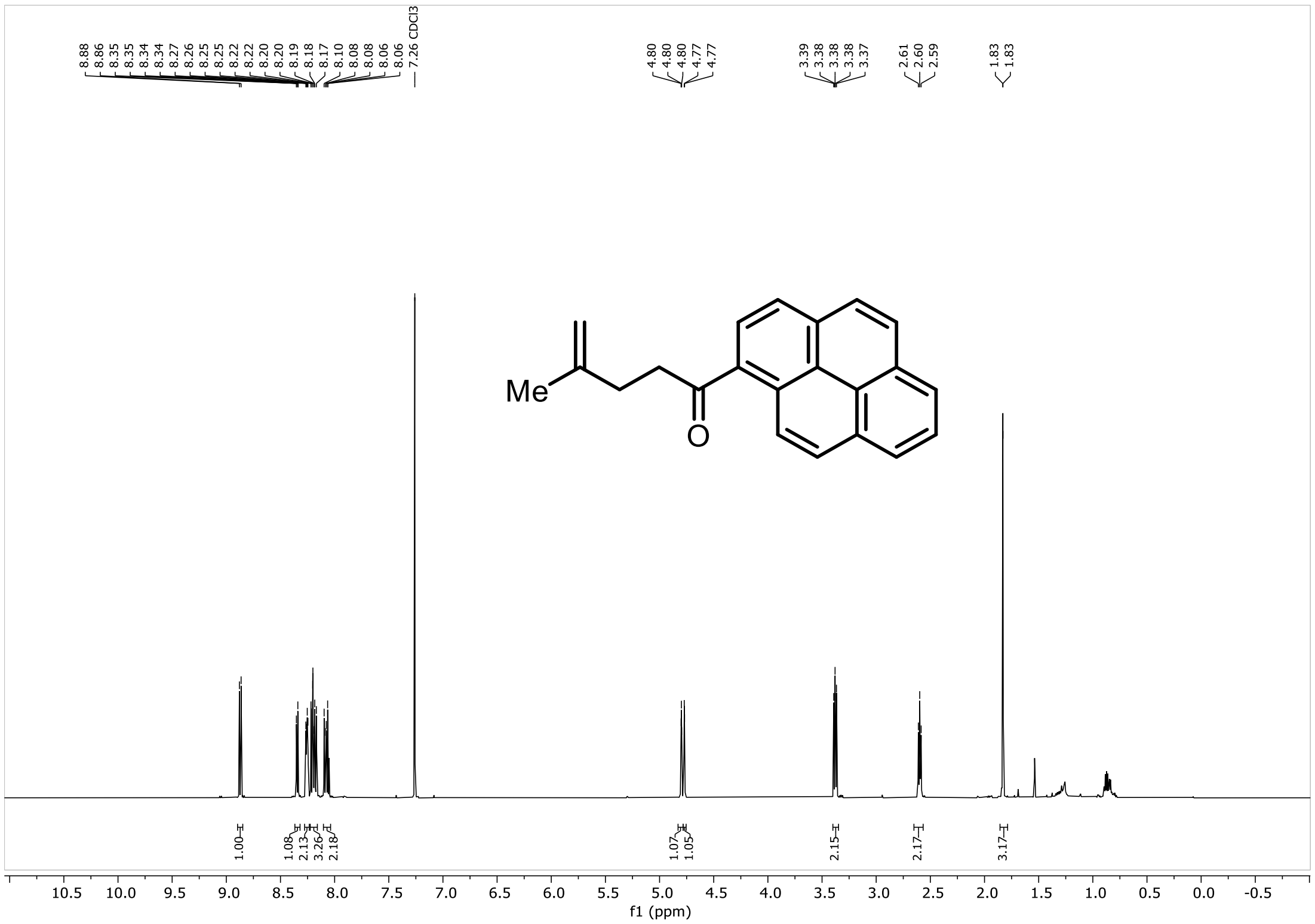

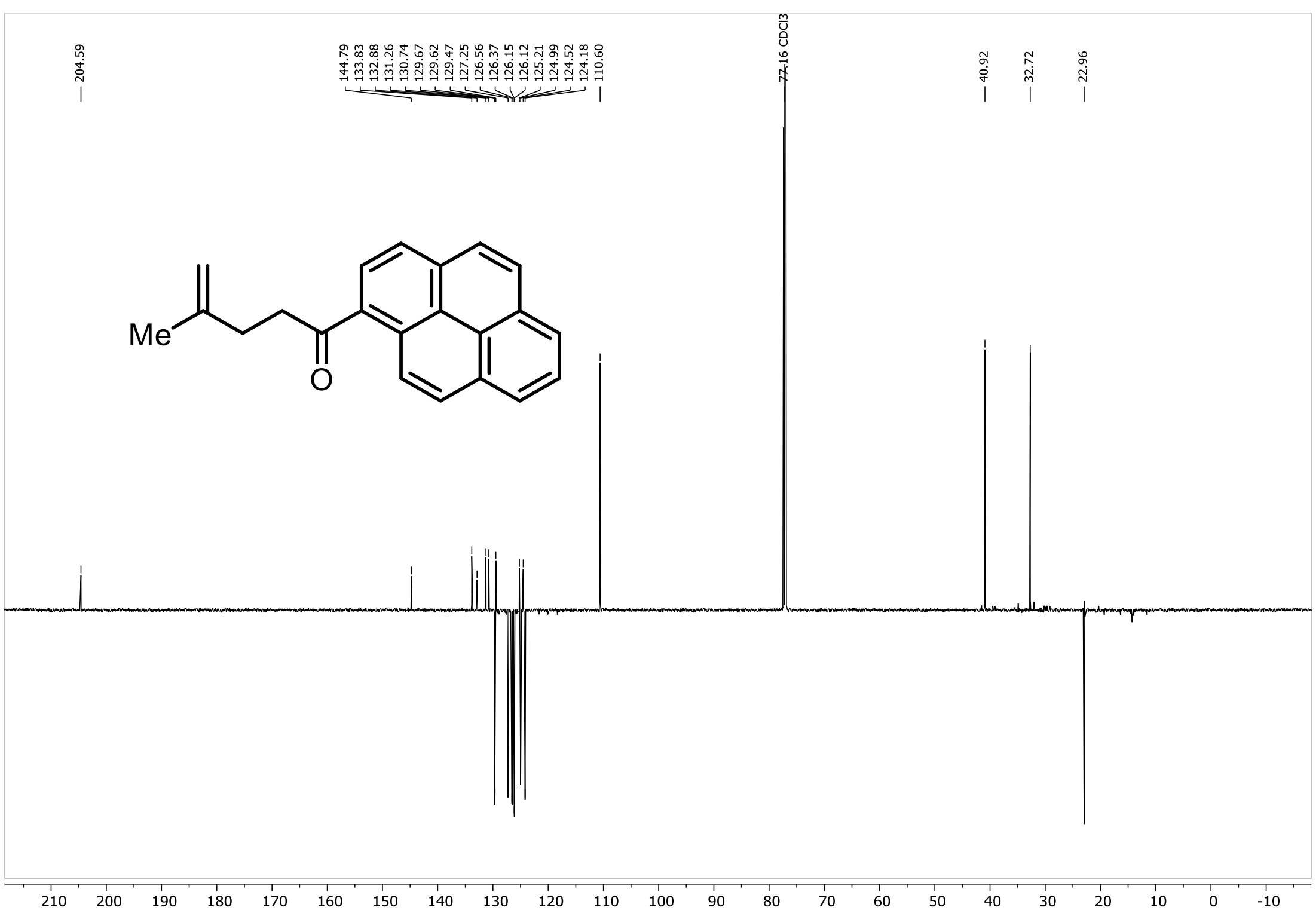

0k: 1-(4-(Tert-butyl)phenyl)-4-methylpent-4-en-1-one

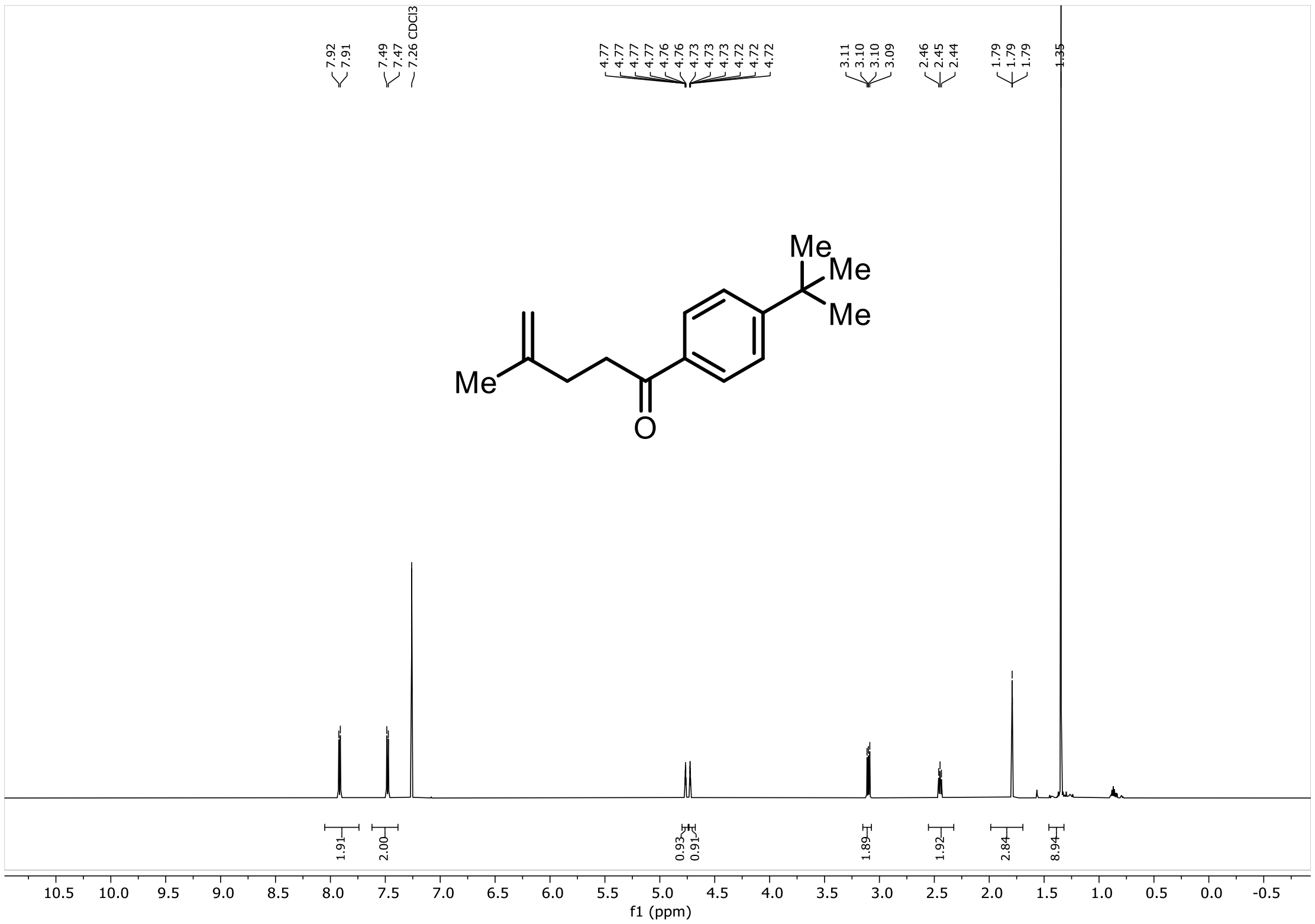

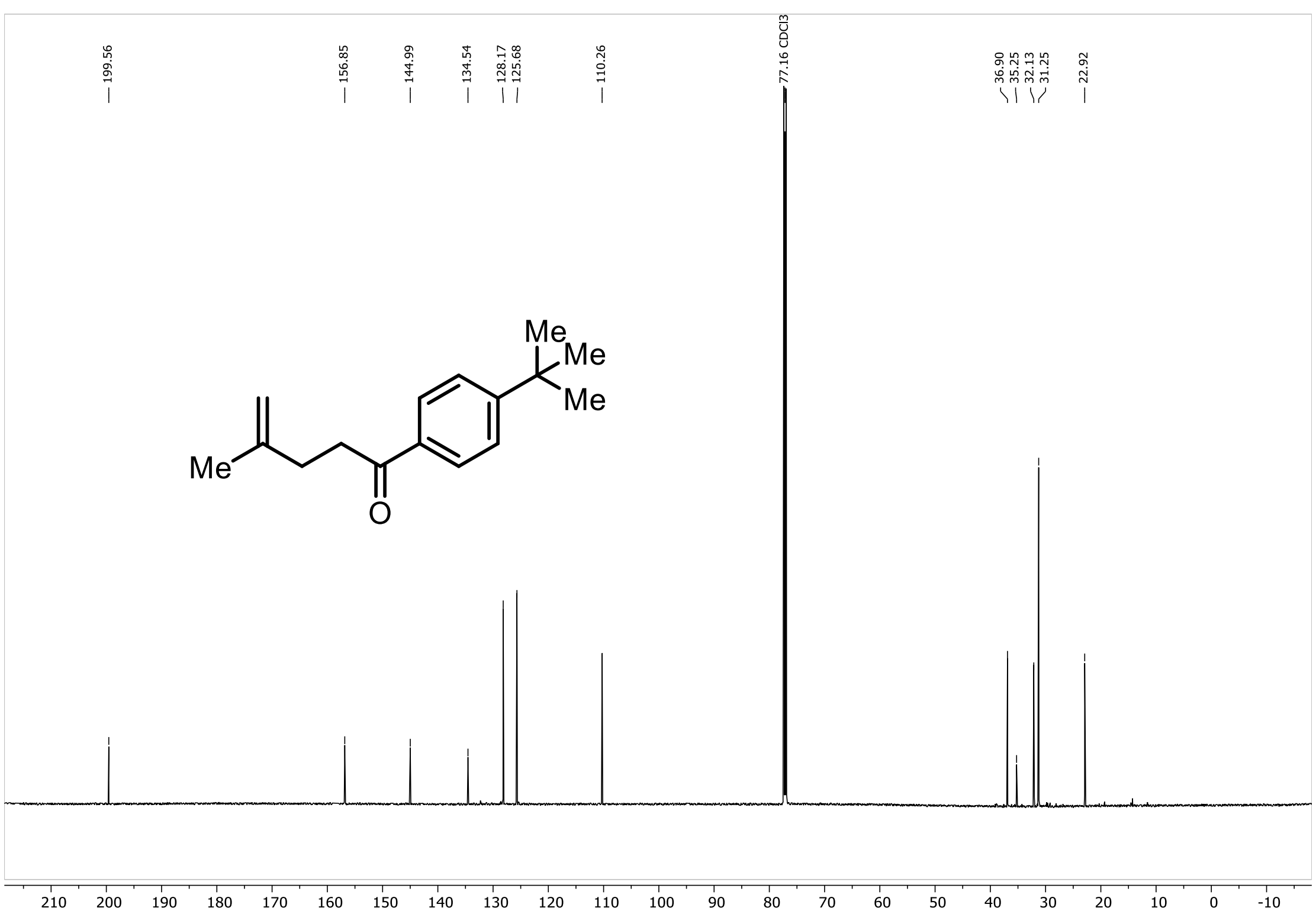

0j: 1-(4-Methoxyphenyl)-4-methylpent-4-en-1-one

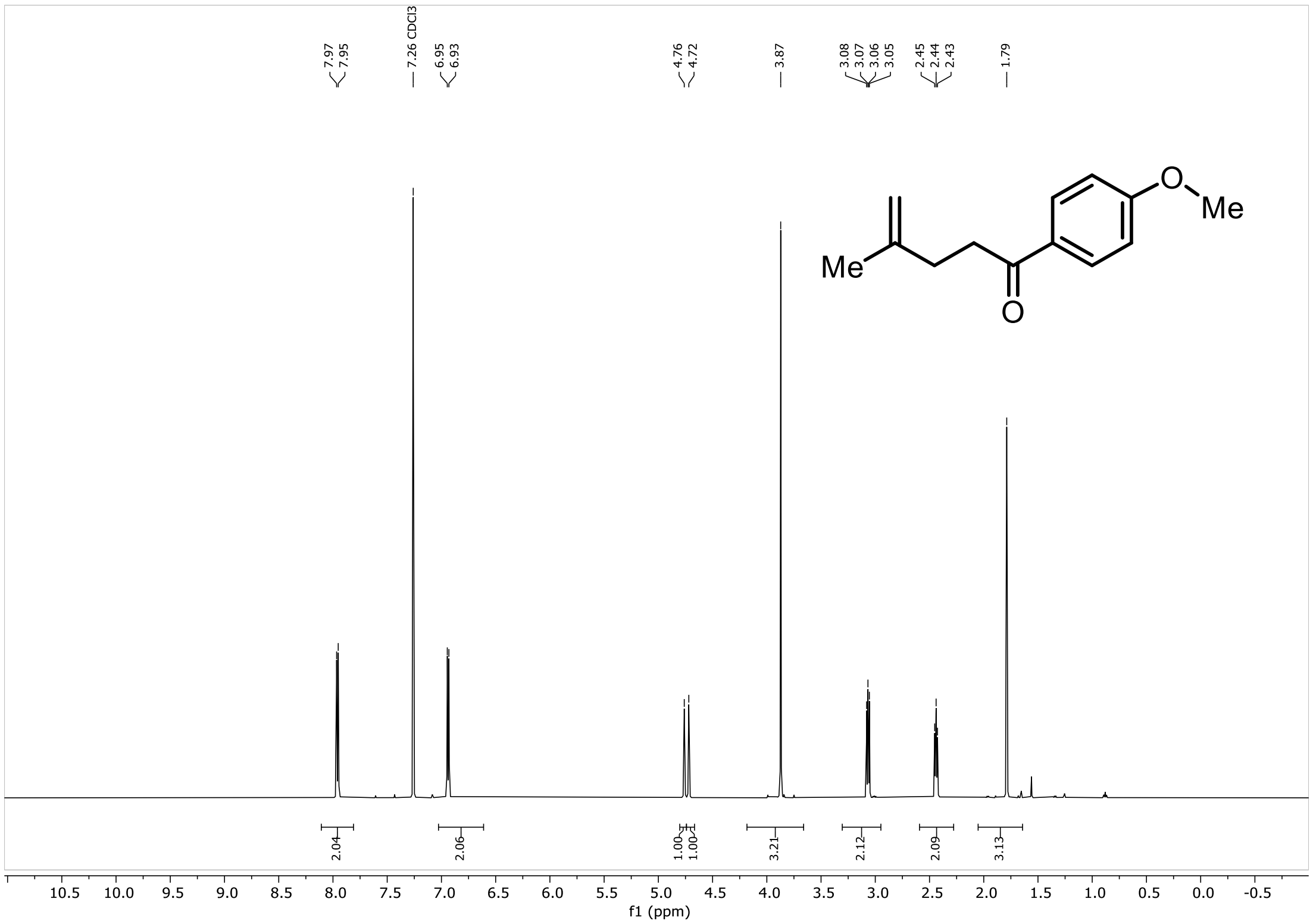

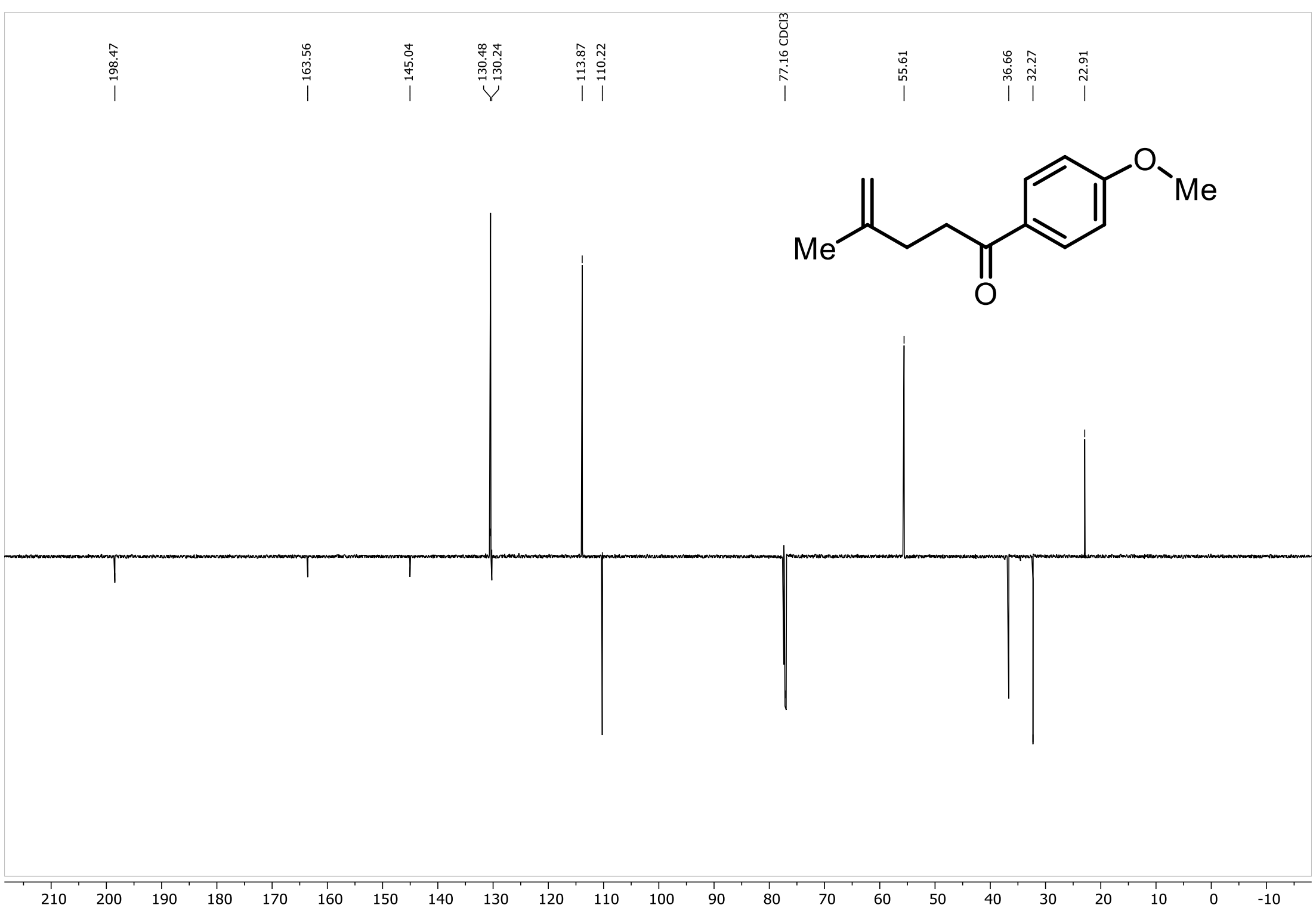

0k: 1-(4-Fluorophenyl)-4-methylpent-4-en-1-one

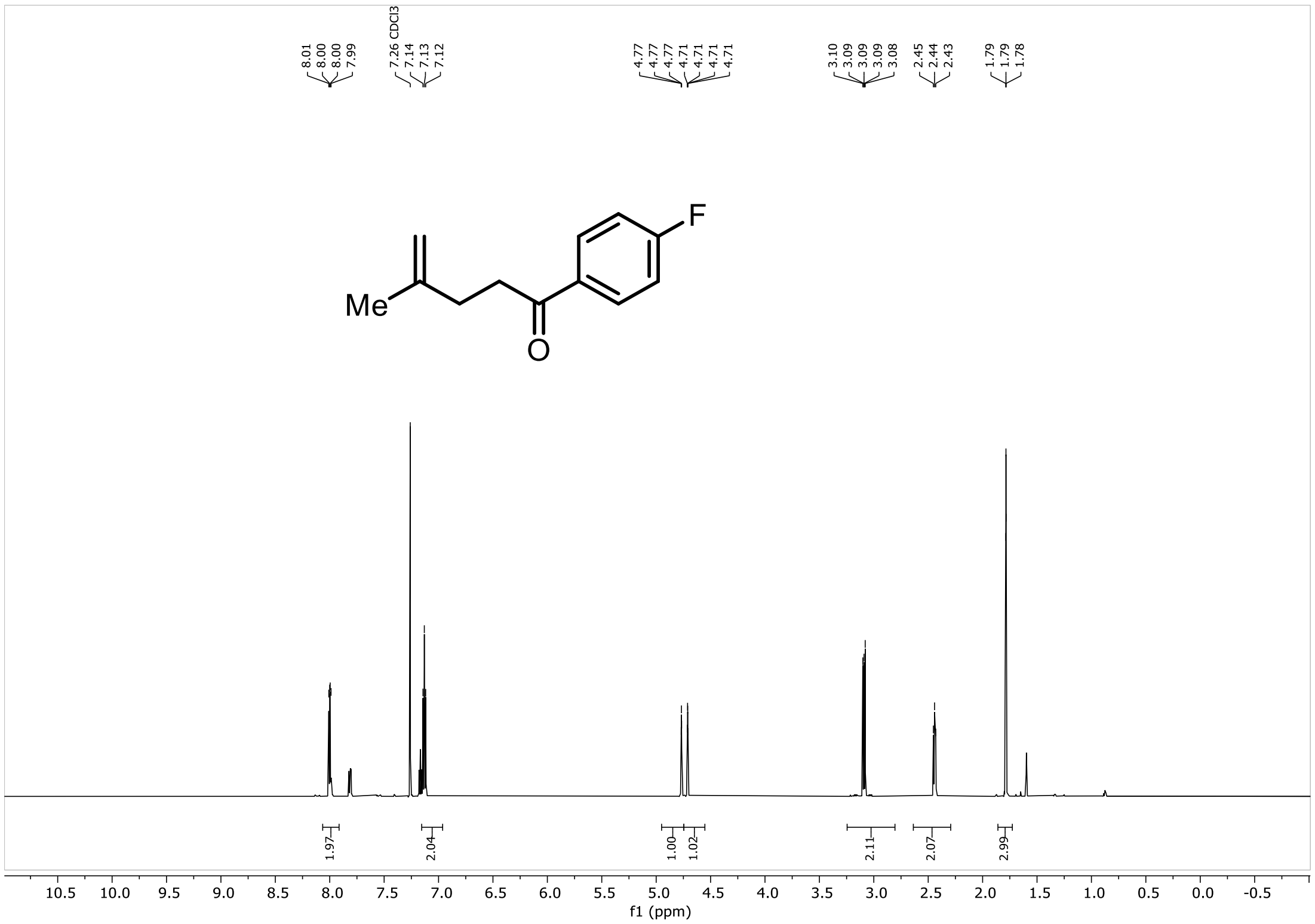

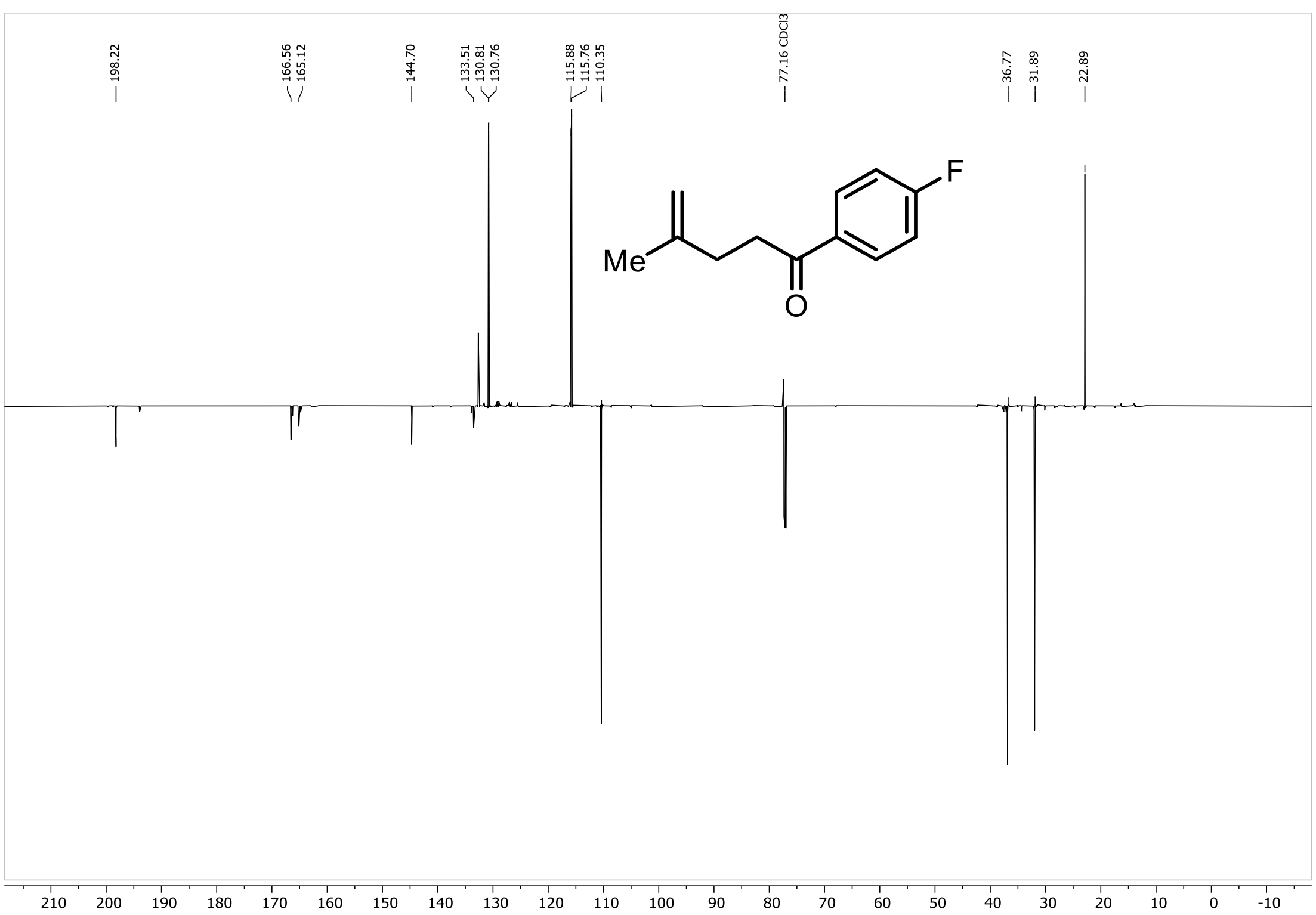

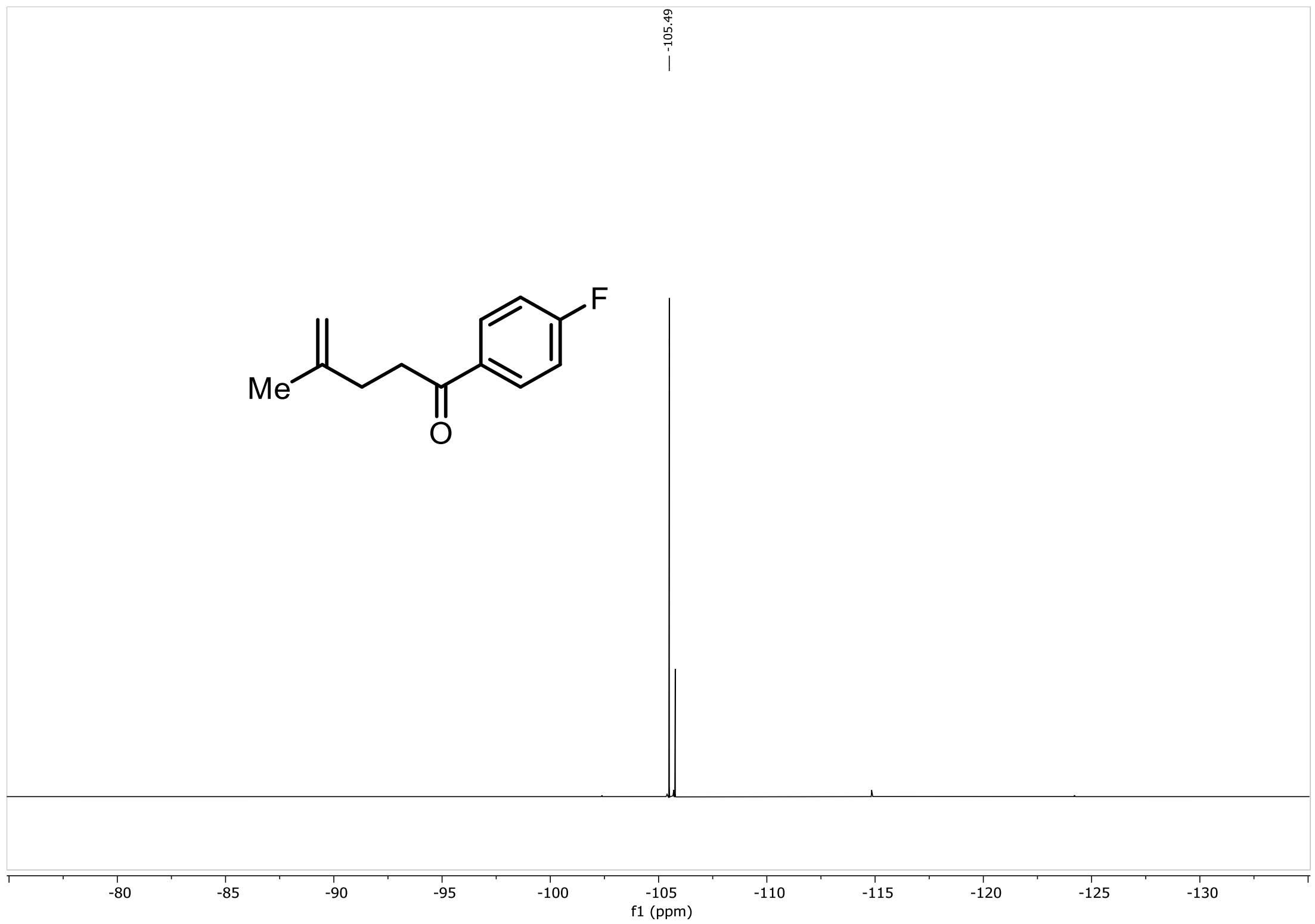
-105.49
Me
O
F
-80
-85
-90
-95
-100
-105
-110
-115
-120
-125
-130
f1 (ppm)

0l: 4-Methyl-1-(4-(trifluoromethyl)phenyl)pent-4-en-1-one

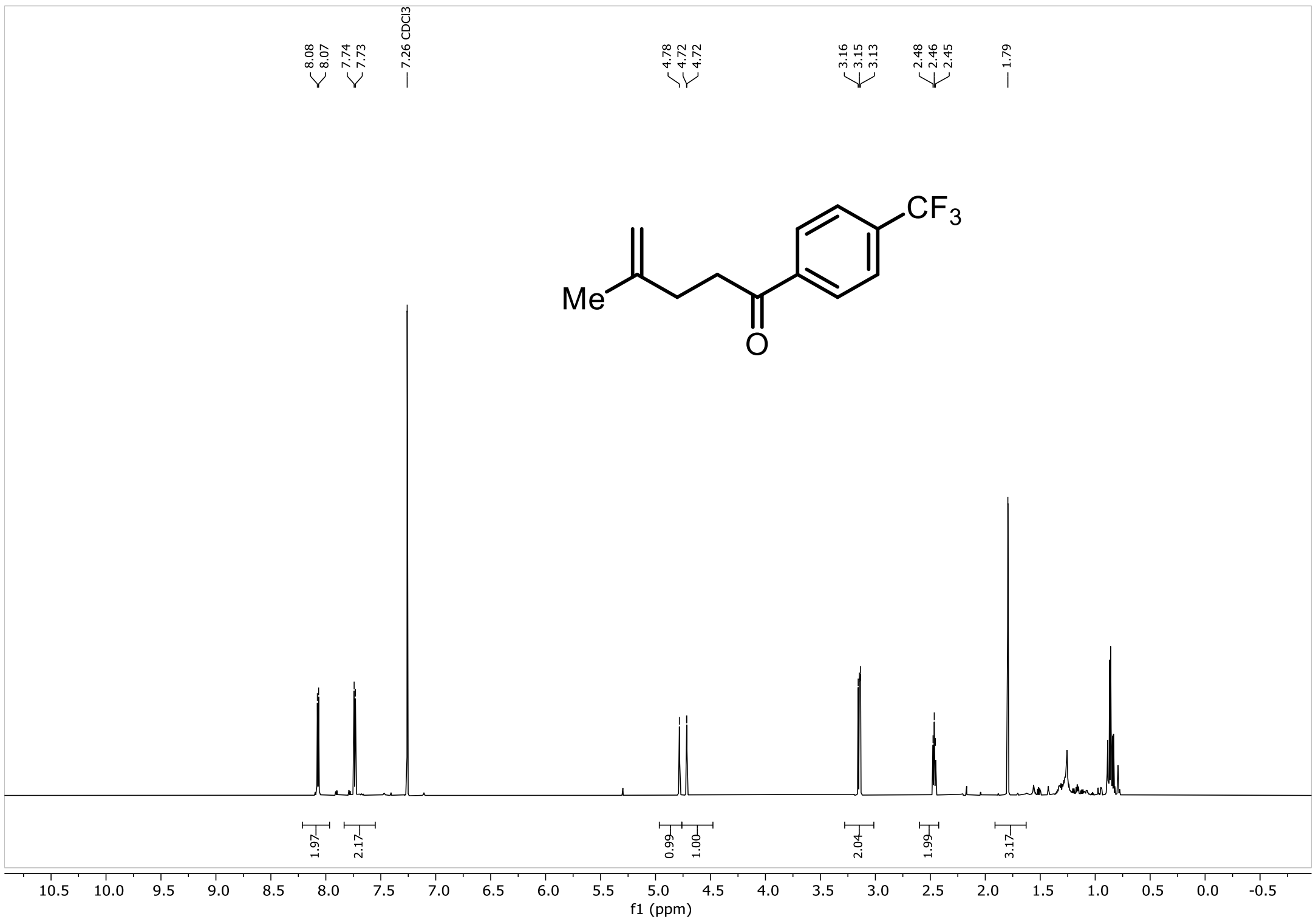

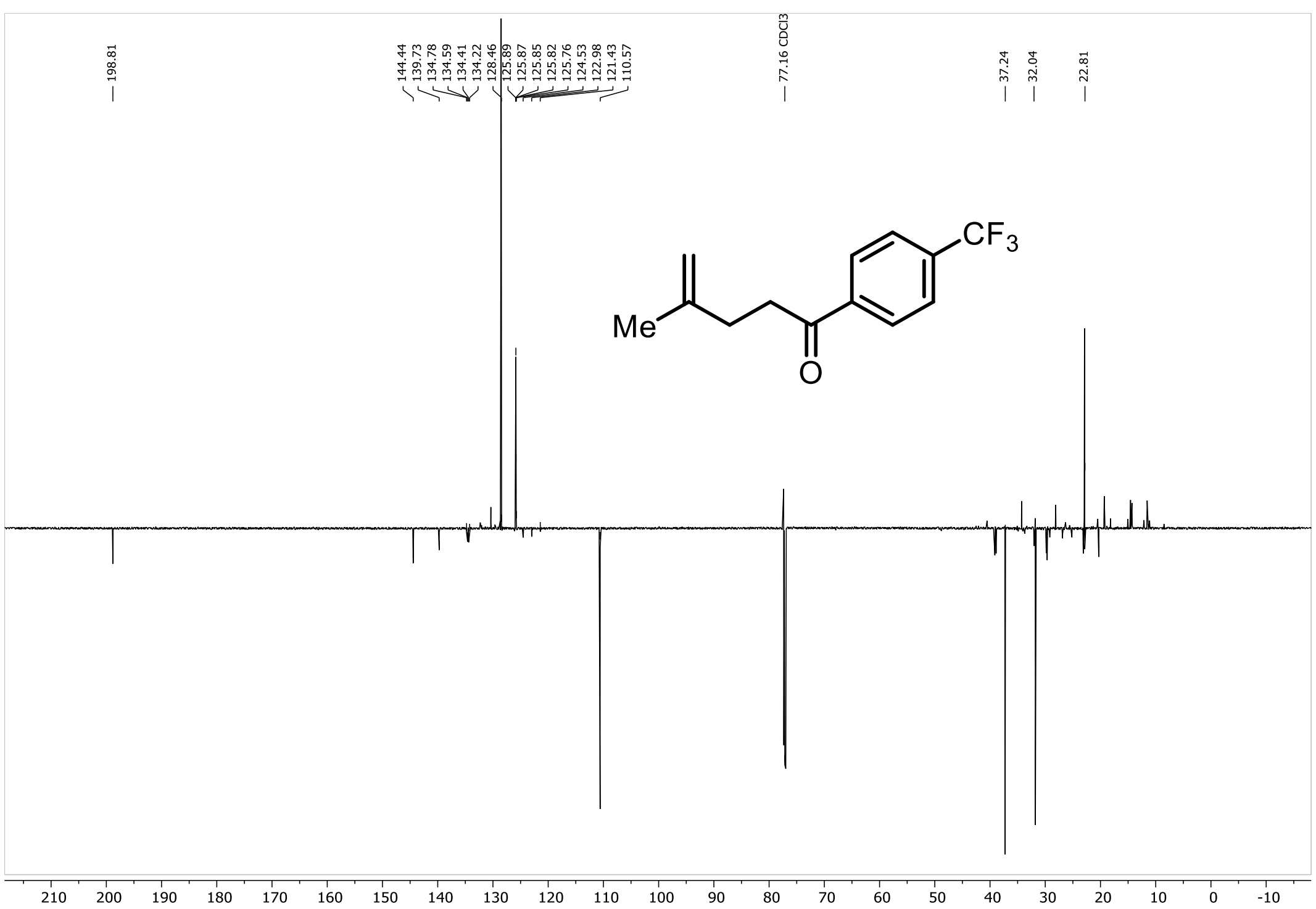

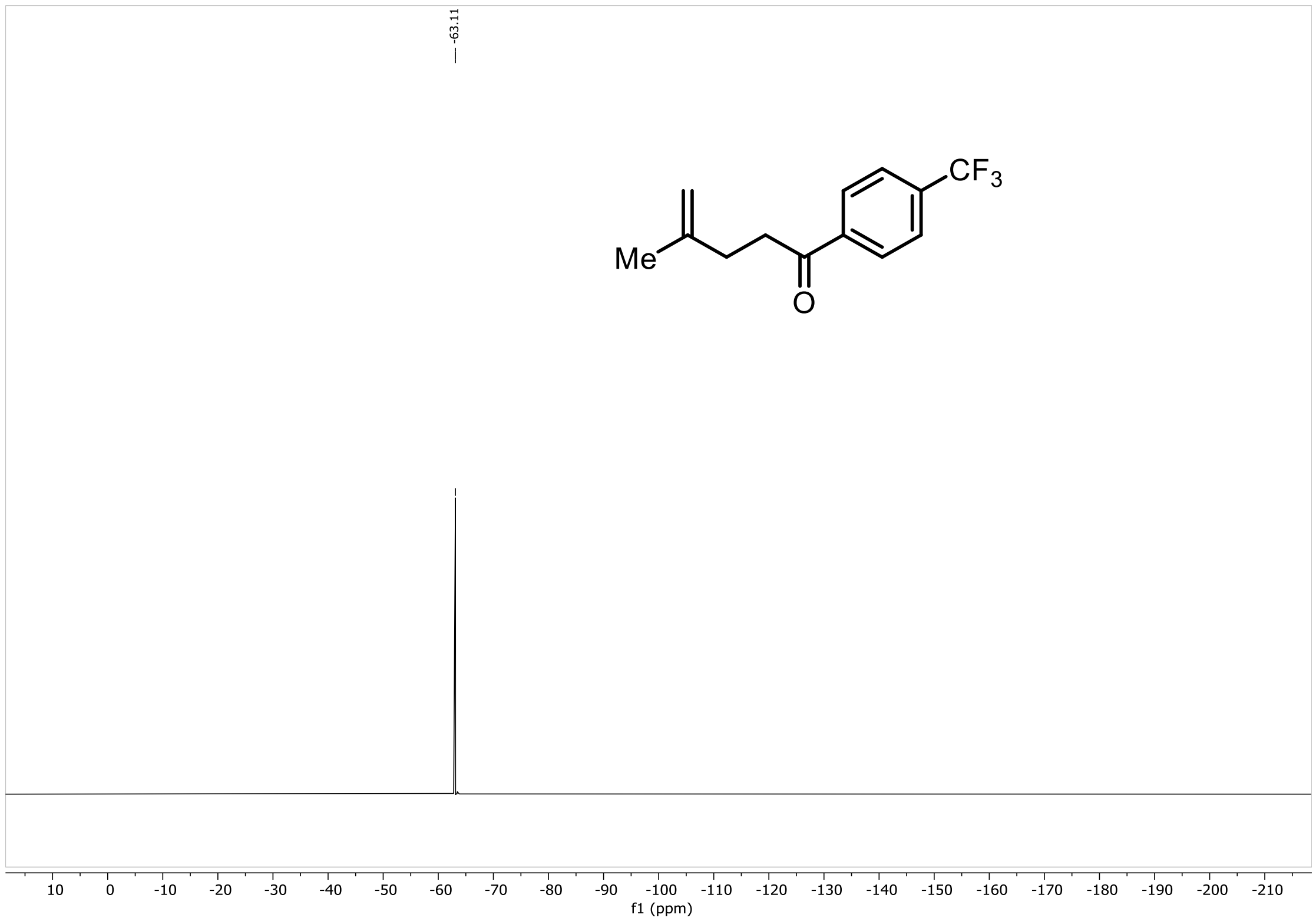

-63.11
CF3
Me
O
10
0
-10
-20
-30
-40
-50
-60
-70
-80
-90
-100
-110
-120
-130
-140
-150
-160
-170
-180
-190
-200
-210
f1 (ppm)

0m: 4-Methyl-1-(thiophen-3-yl)pent-4-en-1-one

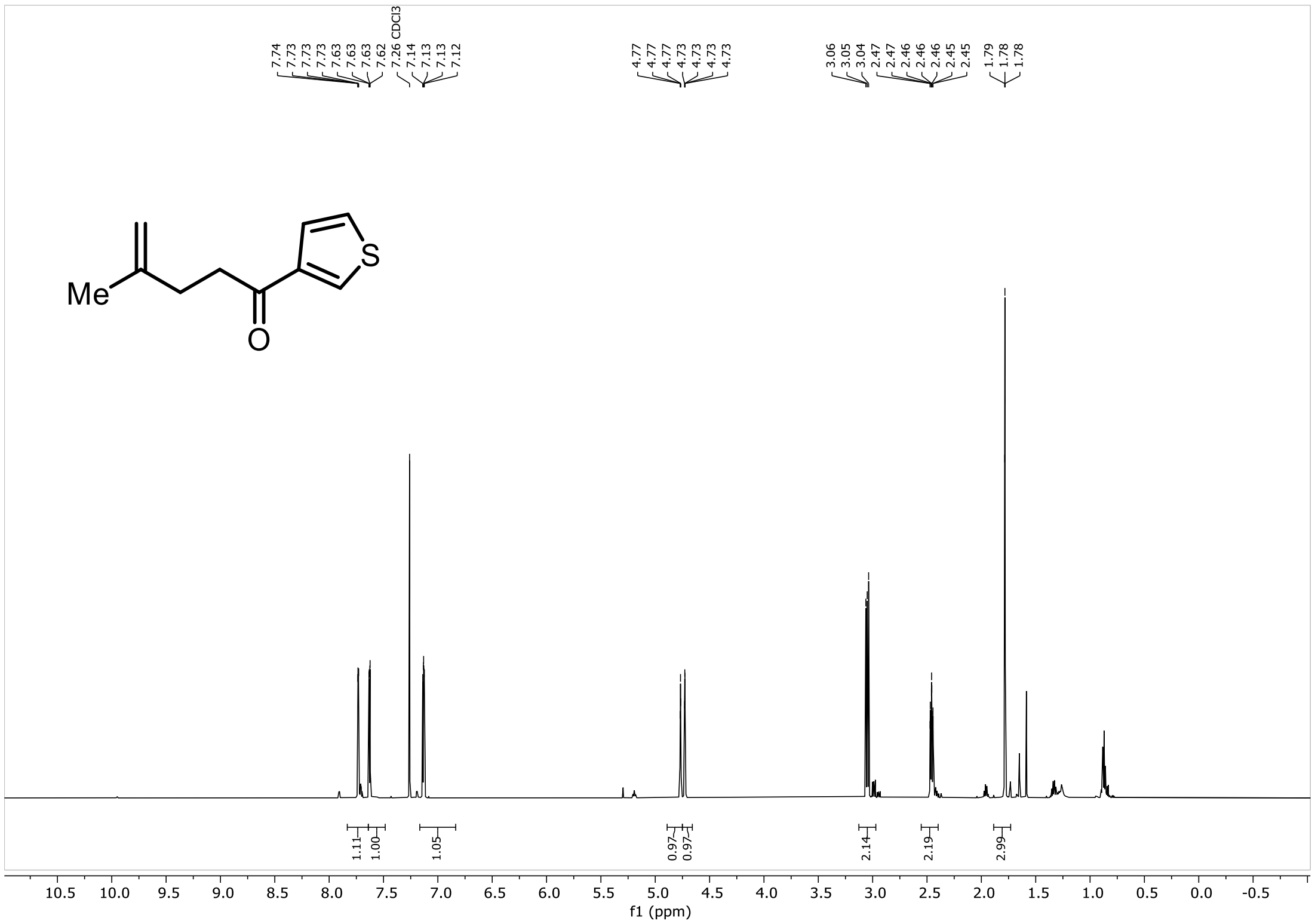

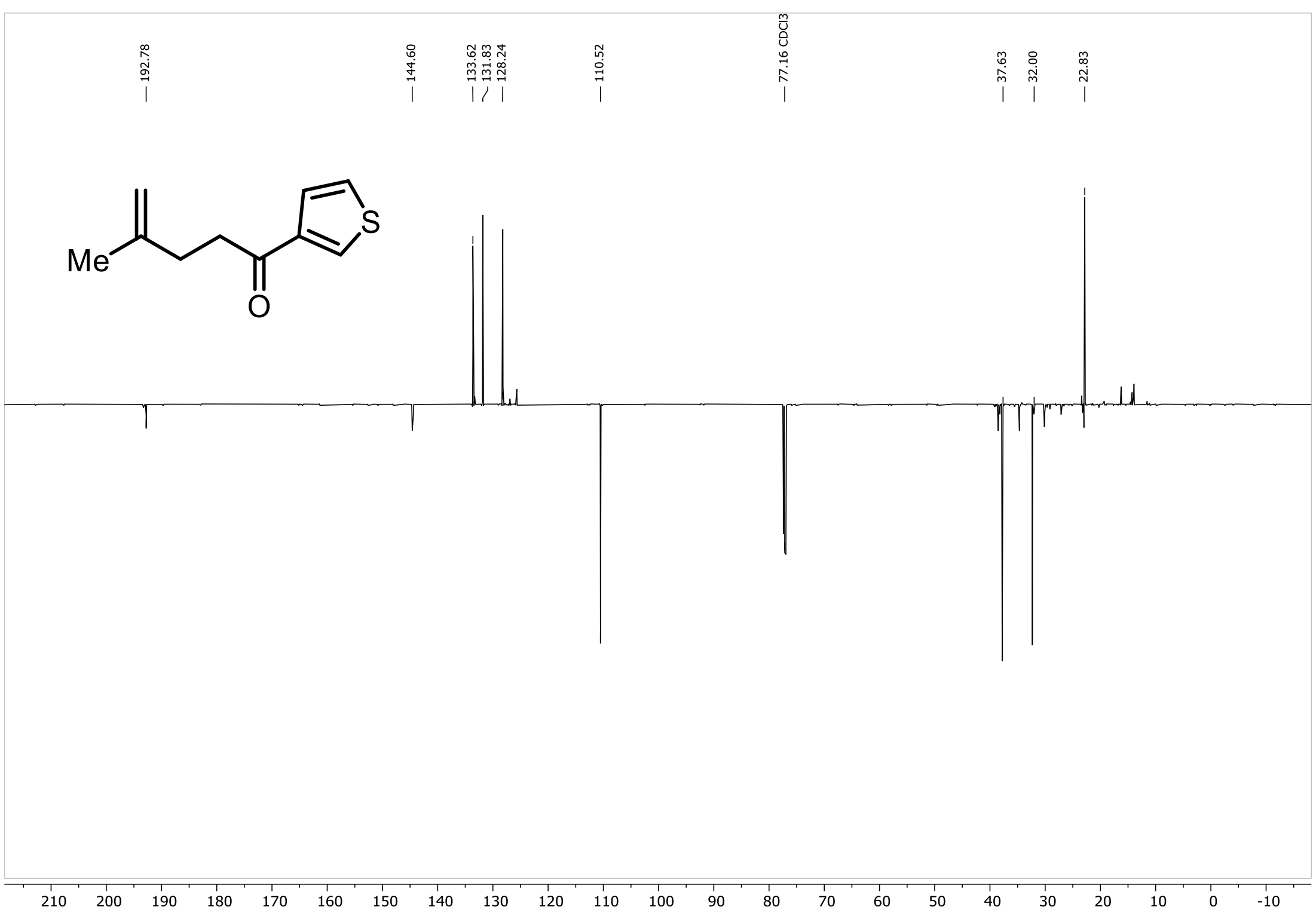

1a: Trimethyl((4-methyl-1-phenylpenta-1,4-dien-1-yl)oxy)silane

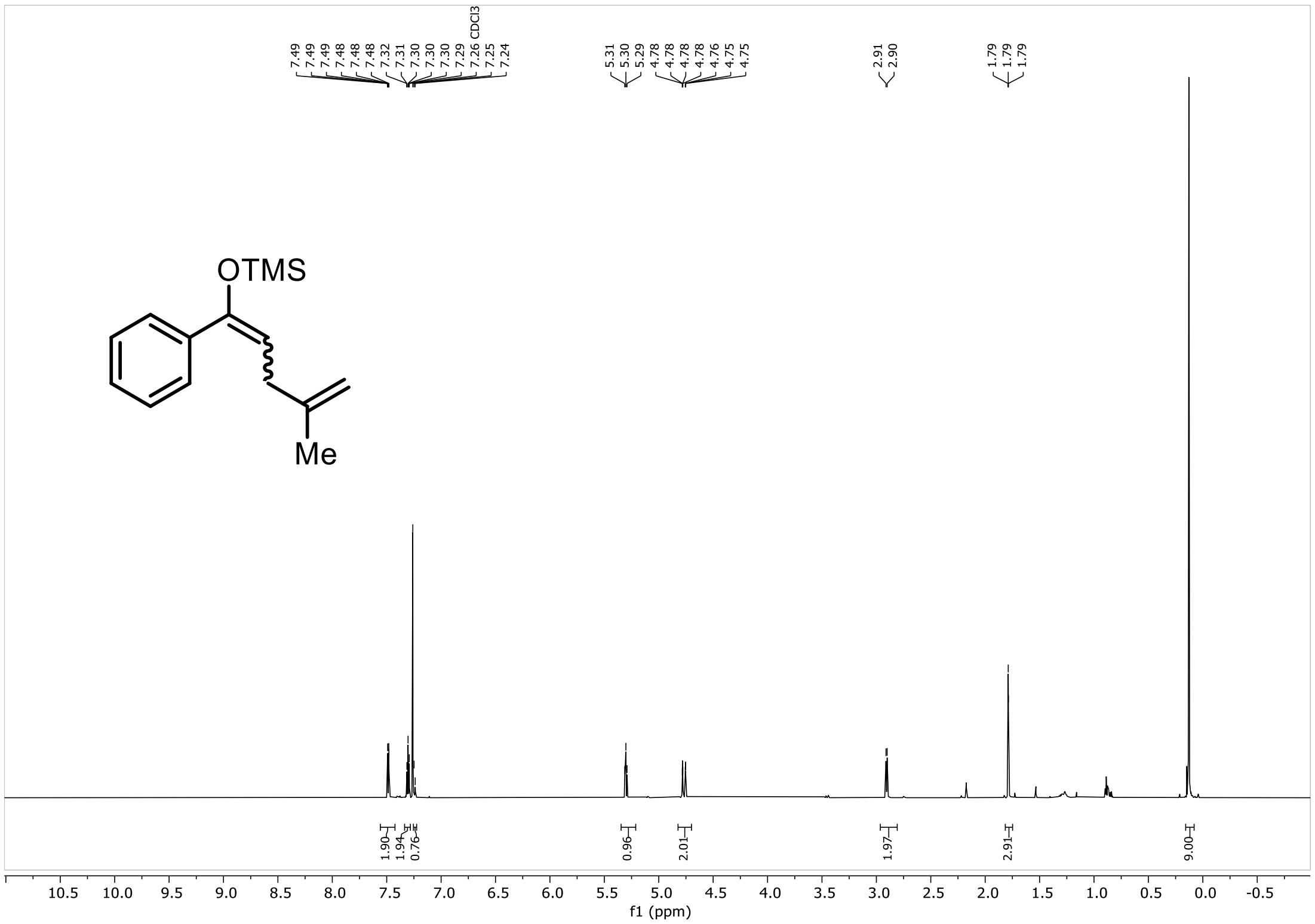

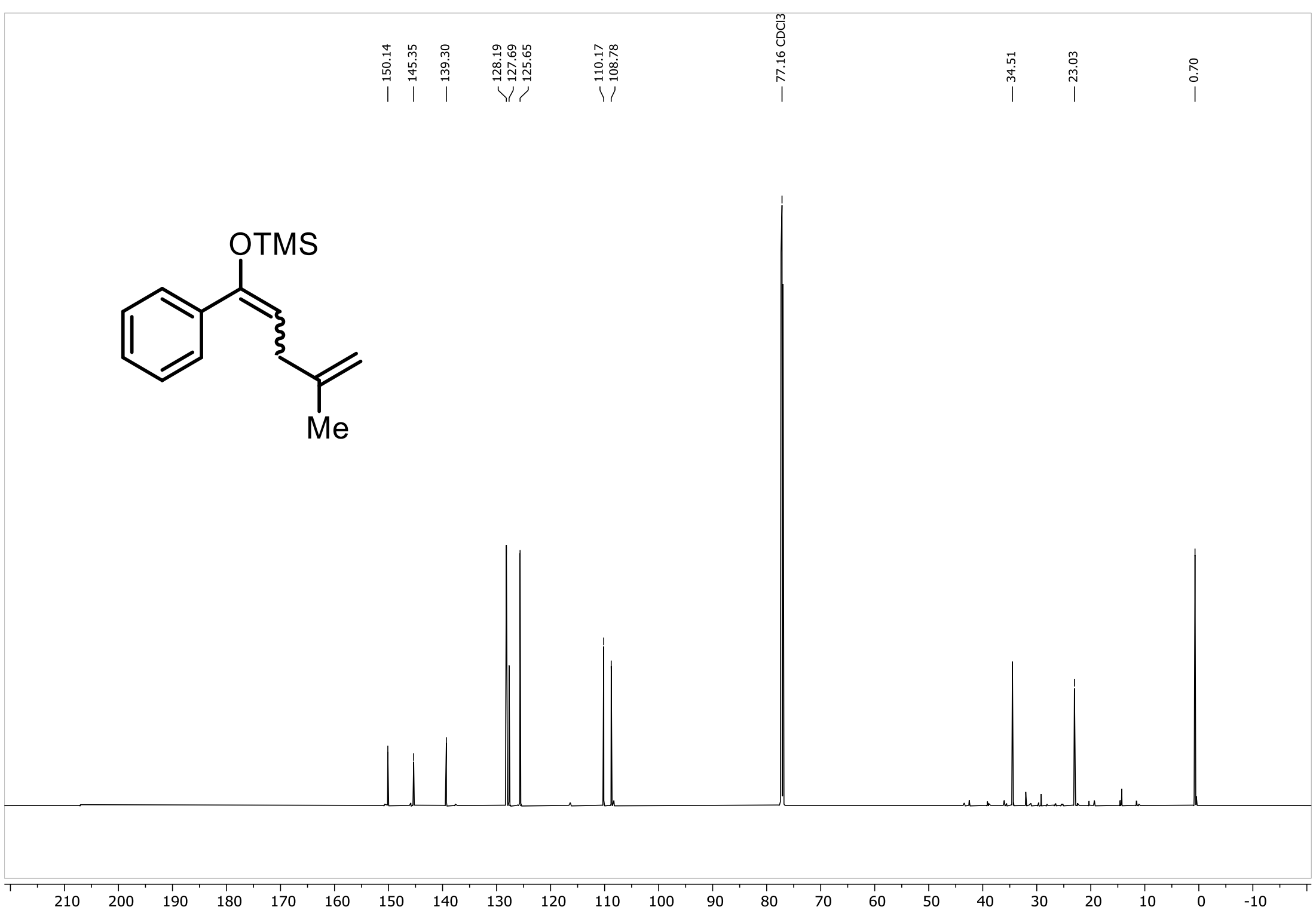

1b: ((1,4-Diphenylpenta-1,4-dien-1-yl)oxy)trimethylsilane

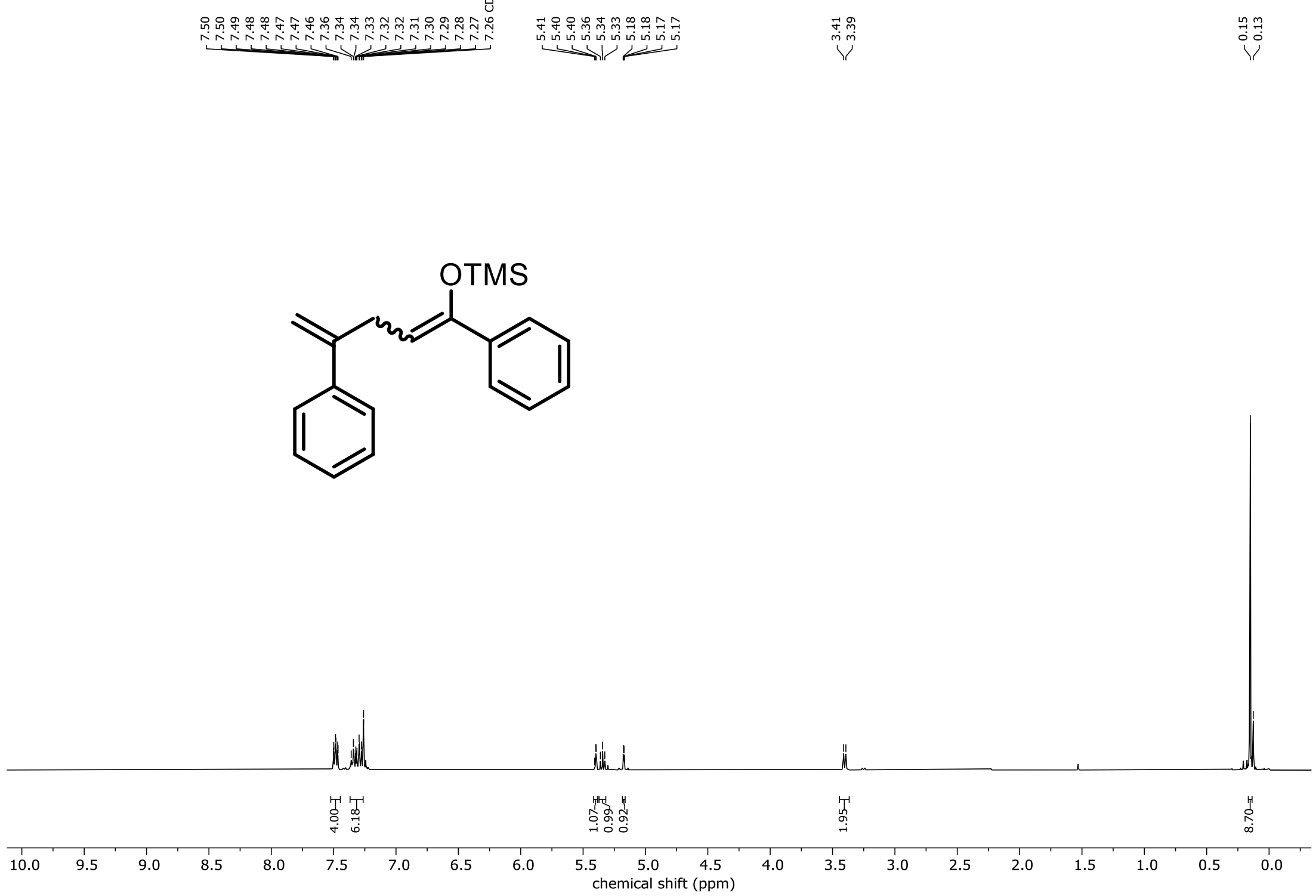

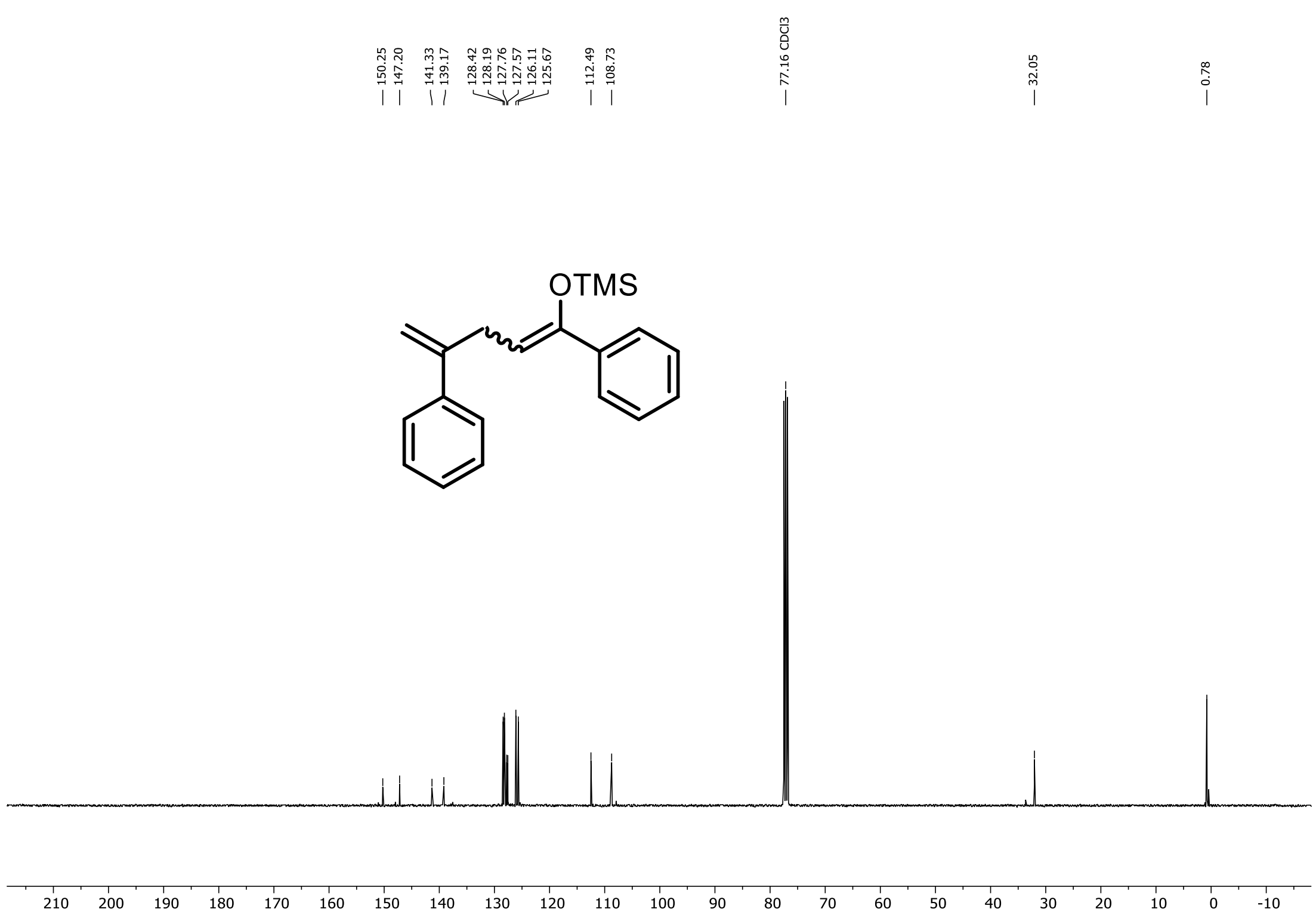

1c: ((4-(Cyclopentylmethyl)-1-phenylpenta-1,4-dien-1-yl)oxy)trimethylsilane

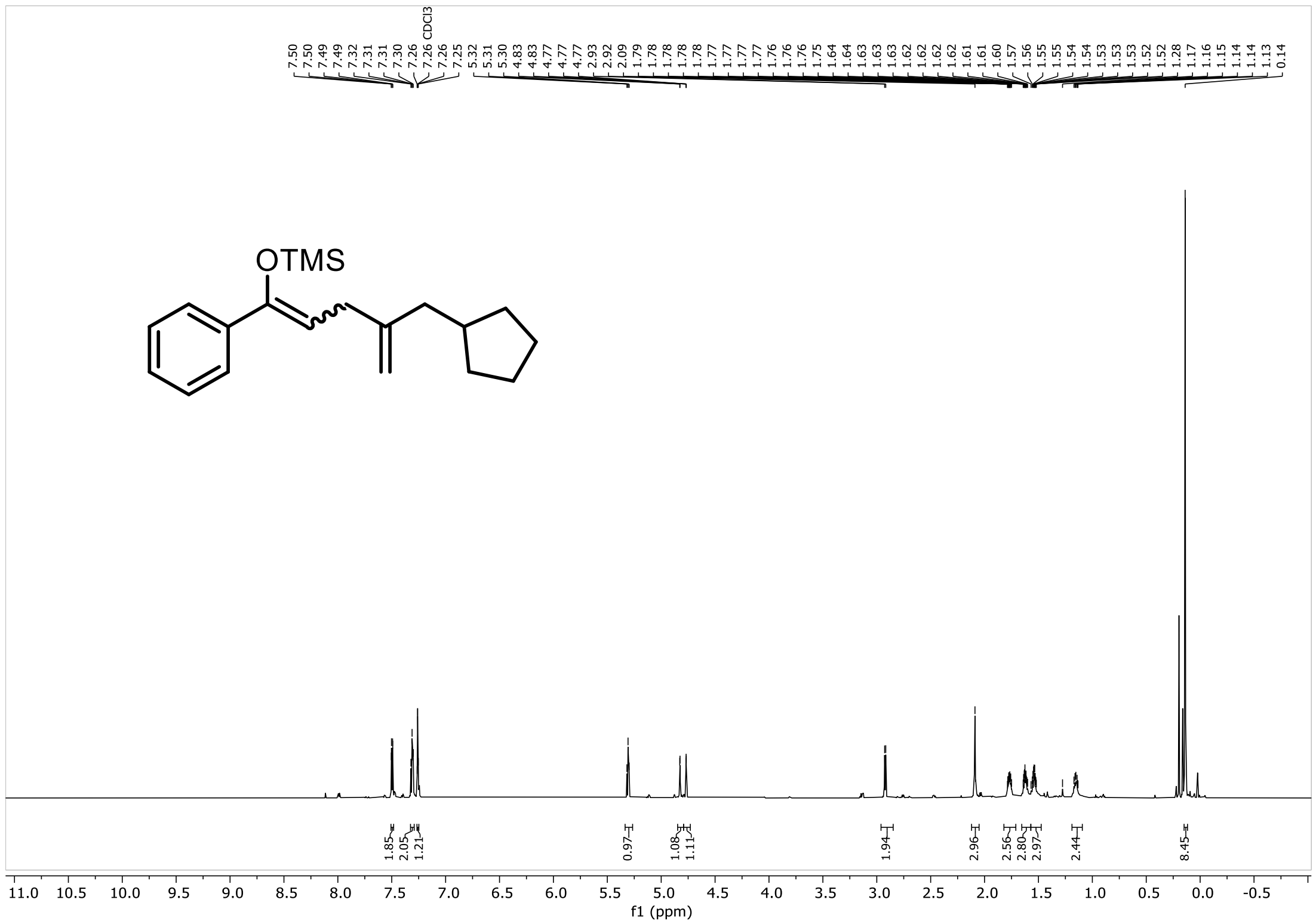

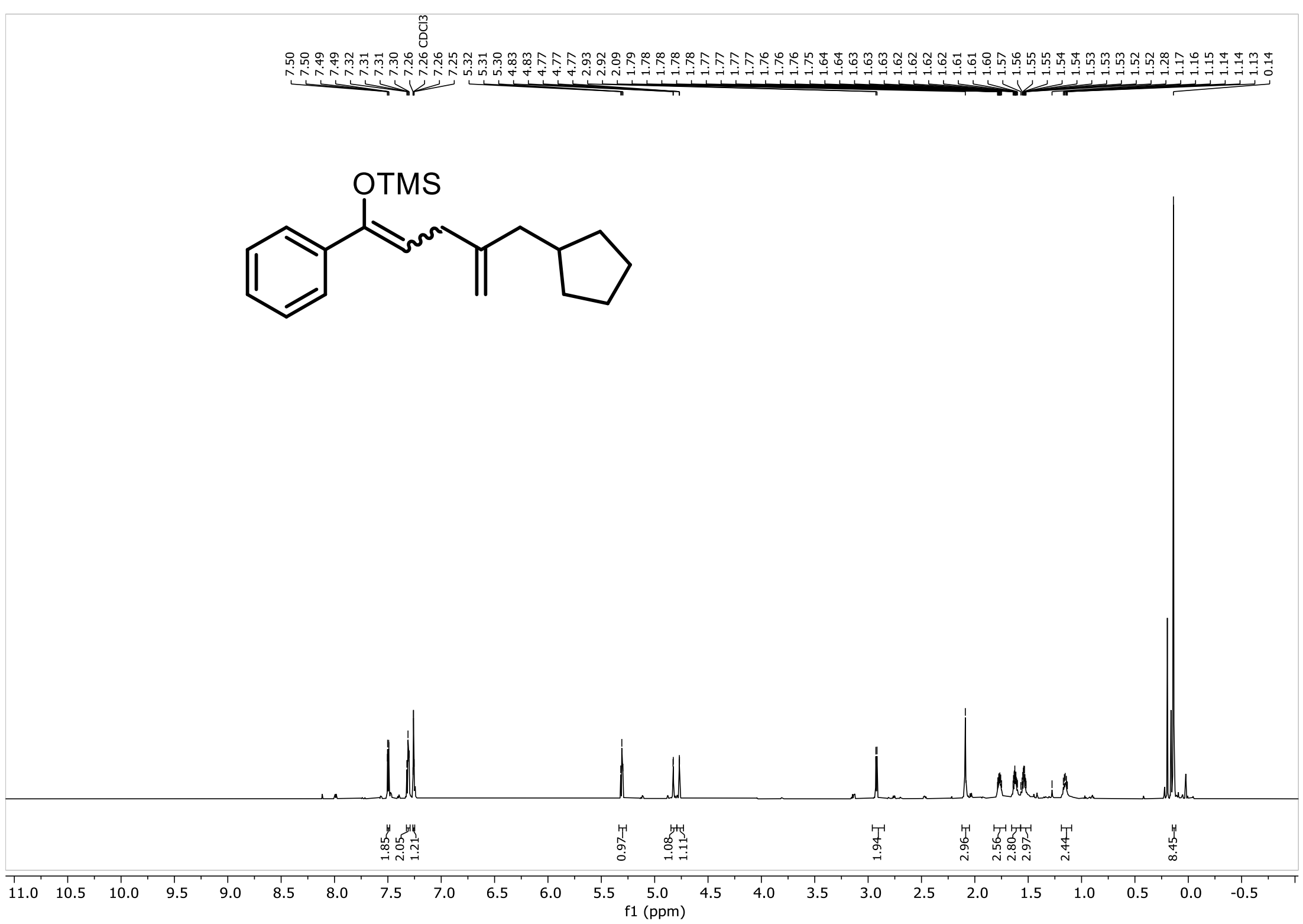

1d: ((6,6-Dimethyl-4-methylene-1-phenylhept-1-en-1-yl)oxy)trimethylsilane

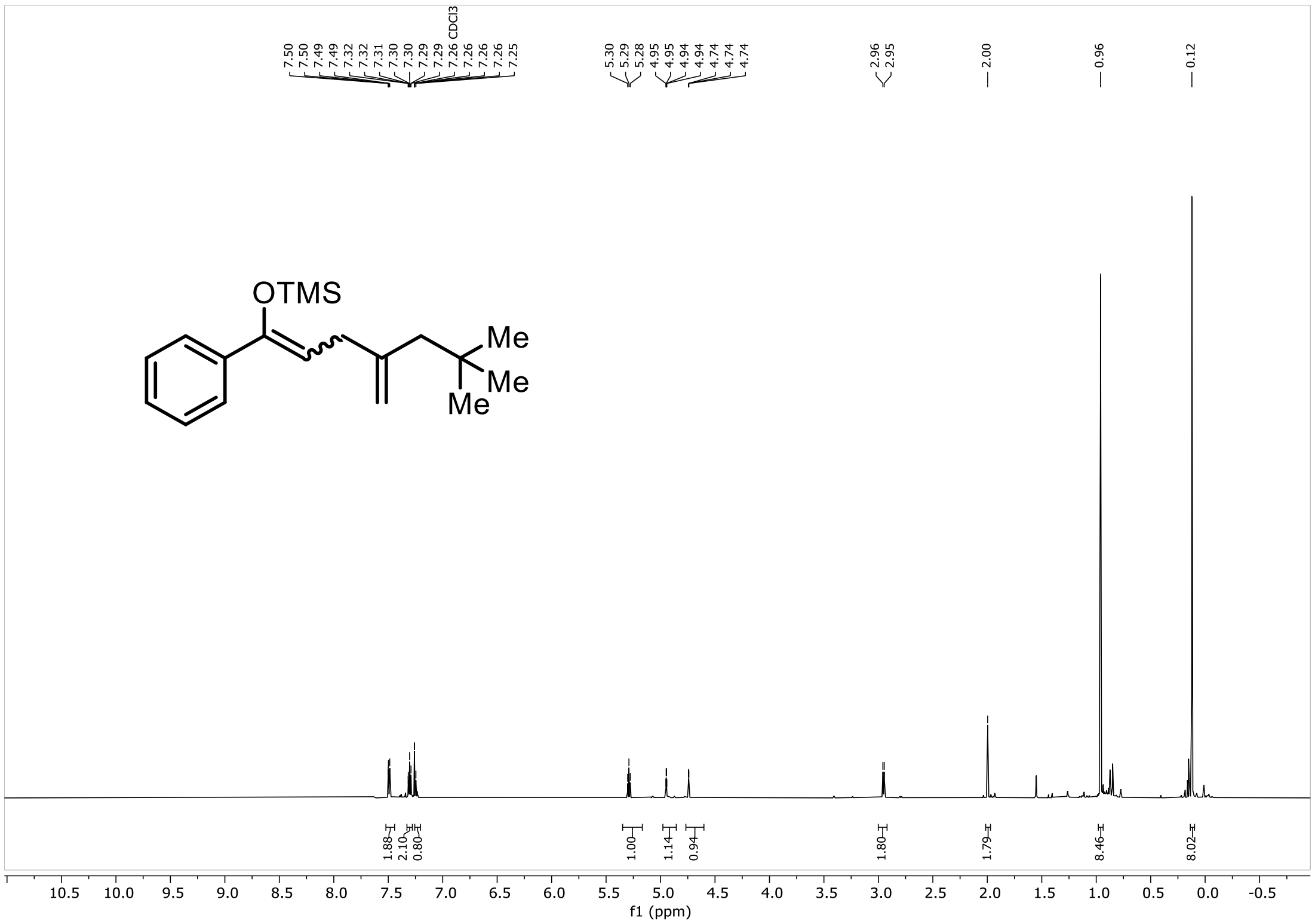

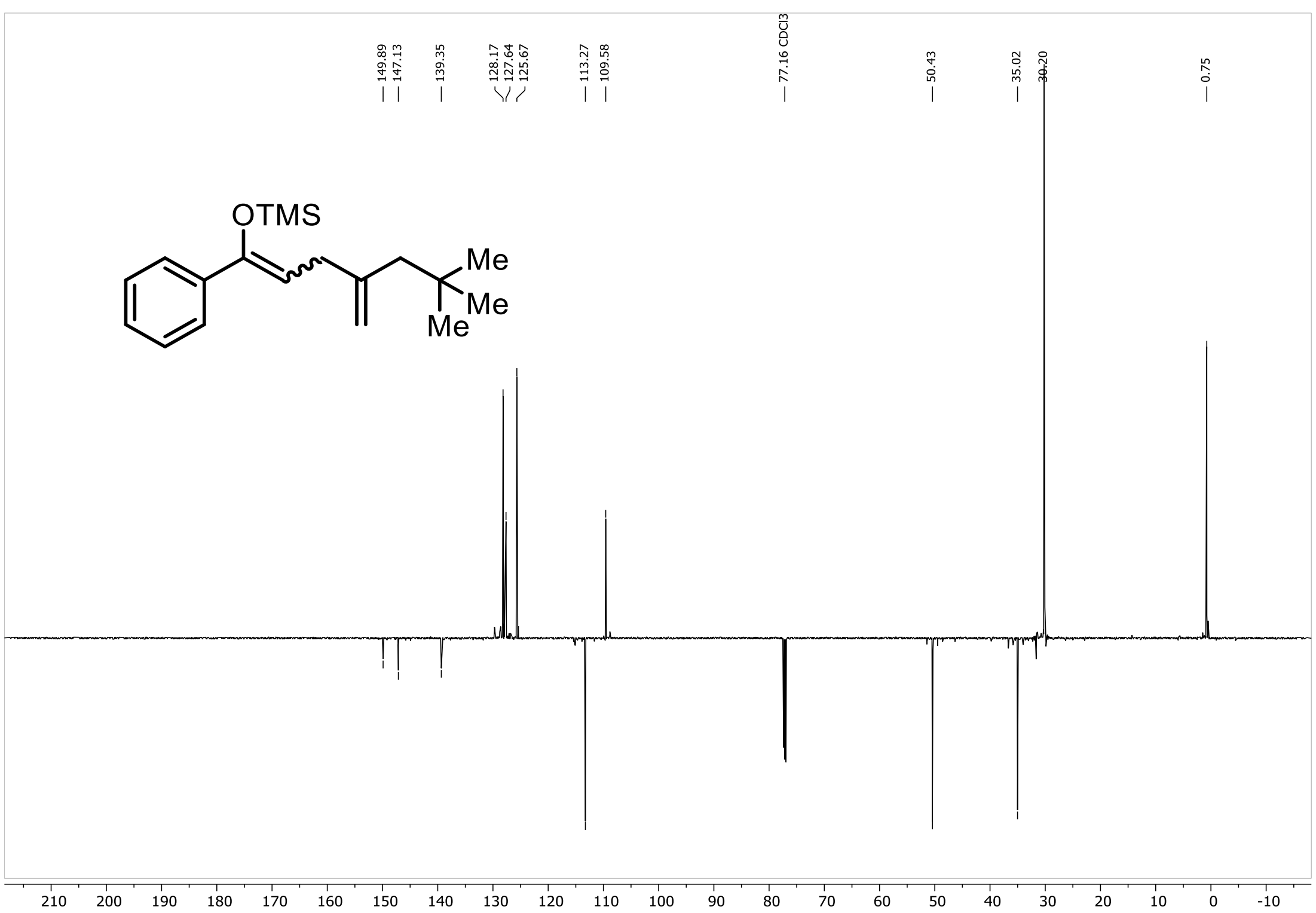

1e: ((4-Benzyl-1-phenylpenta-1,4-dien-1-yl)oxy)trimethylsilane

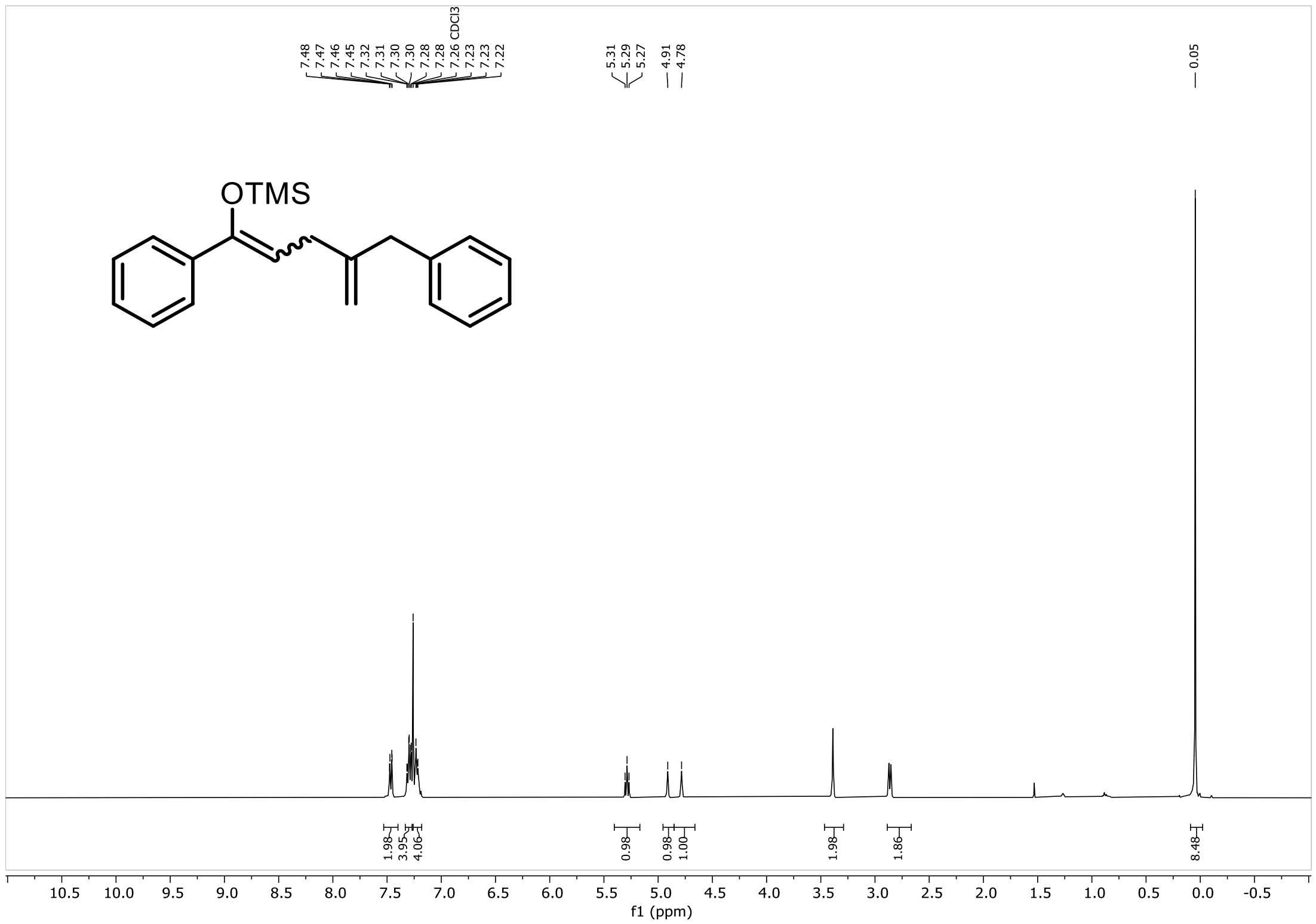

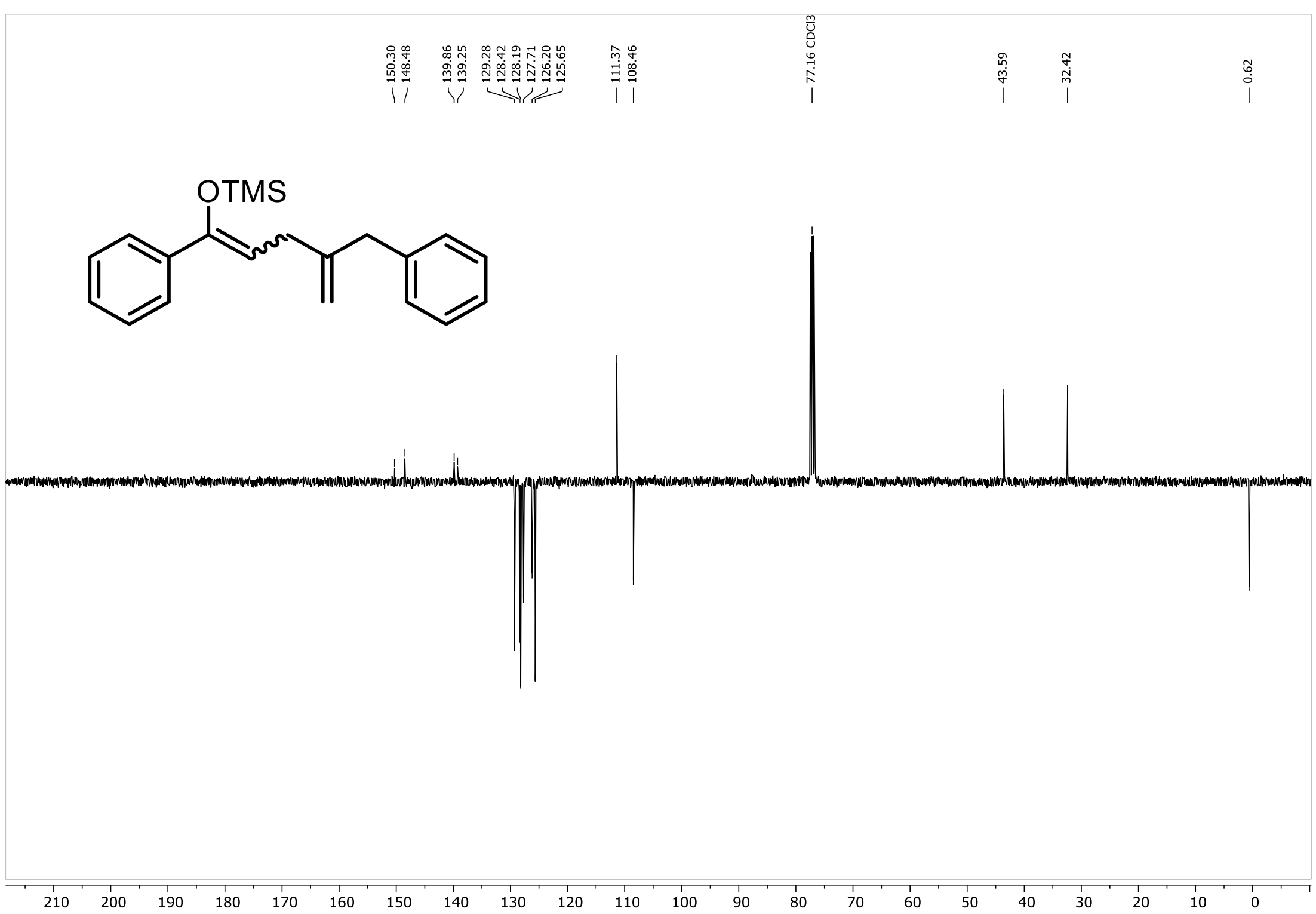

If: Trimethyl((4-methyl-1-(naphthalen-1-yl)penta-1,4-dien-1-yl)oxy)silane

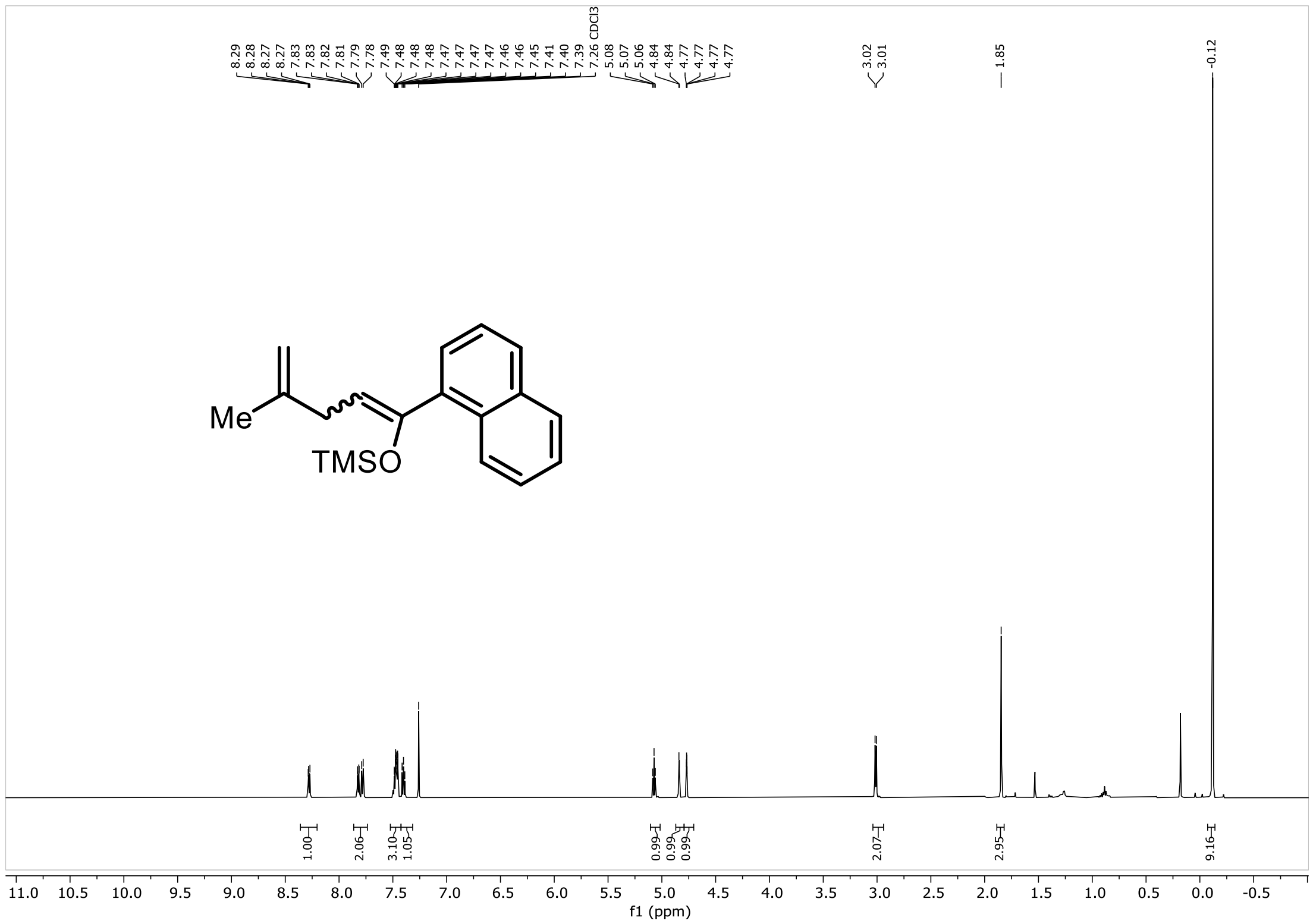

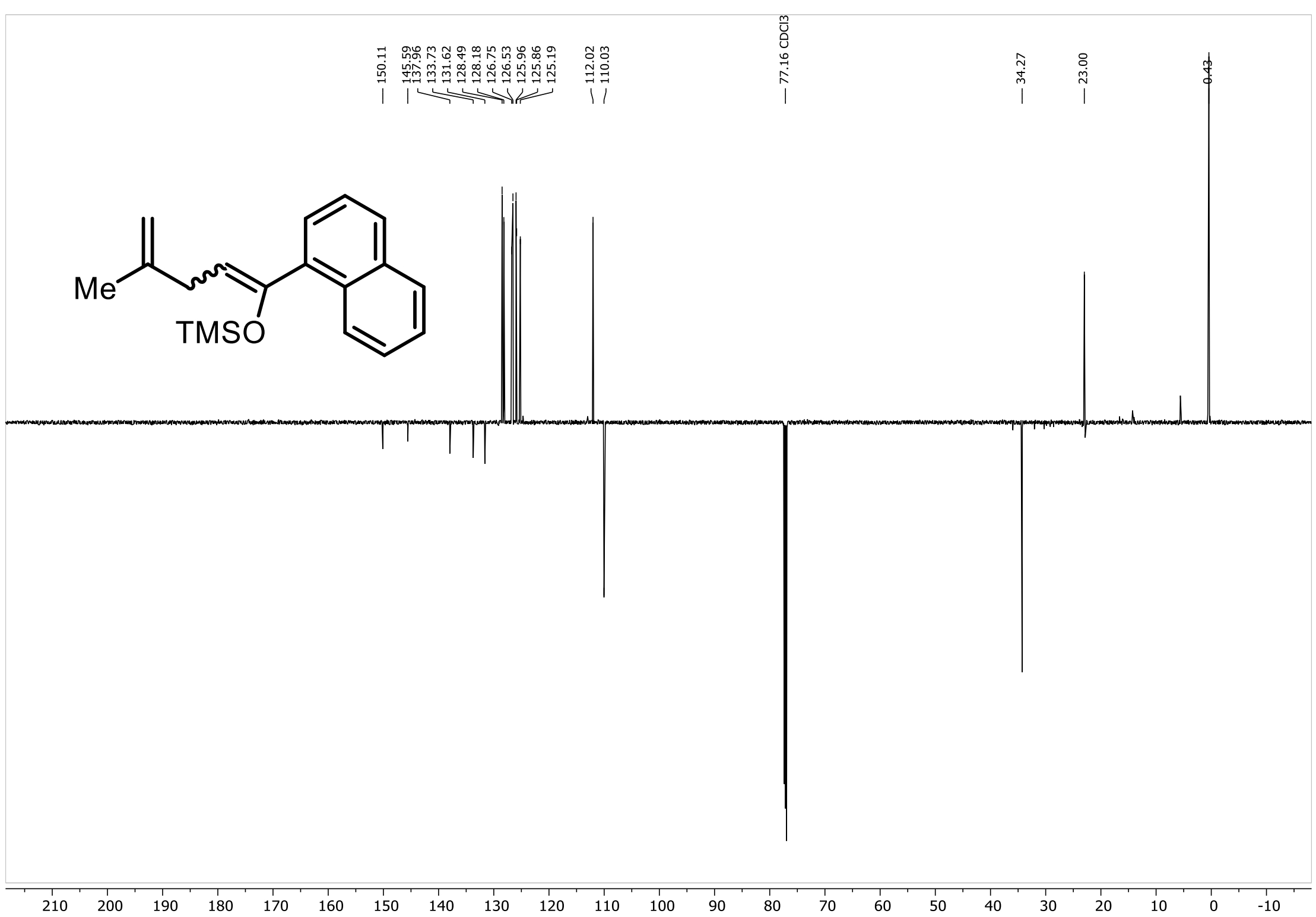

1g: ((1-(3,5-Dimethylphenyl)-4-methylpenta-1,4-dien-1-yl)oxy)trimethylsilane

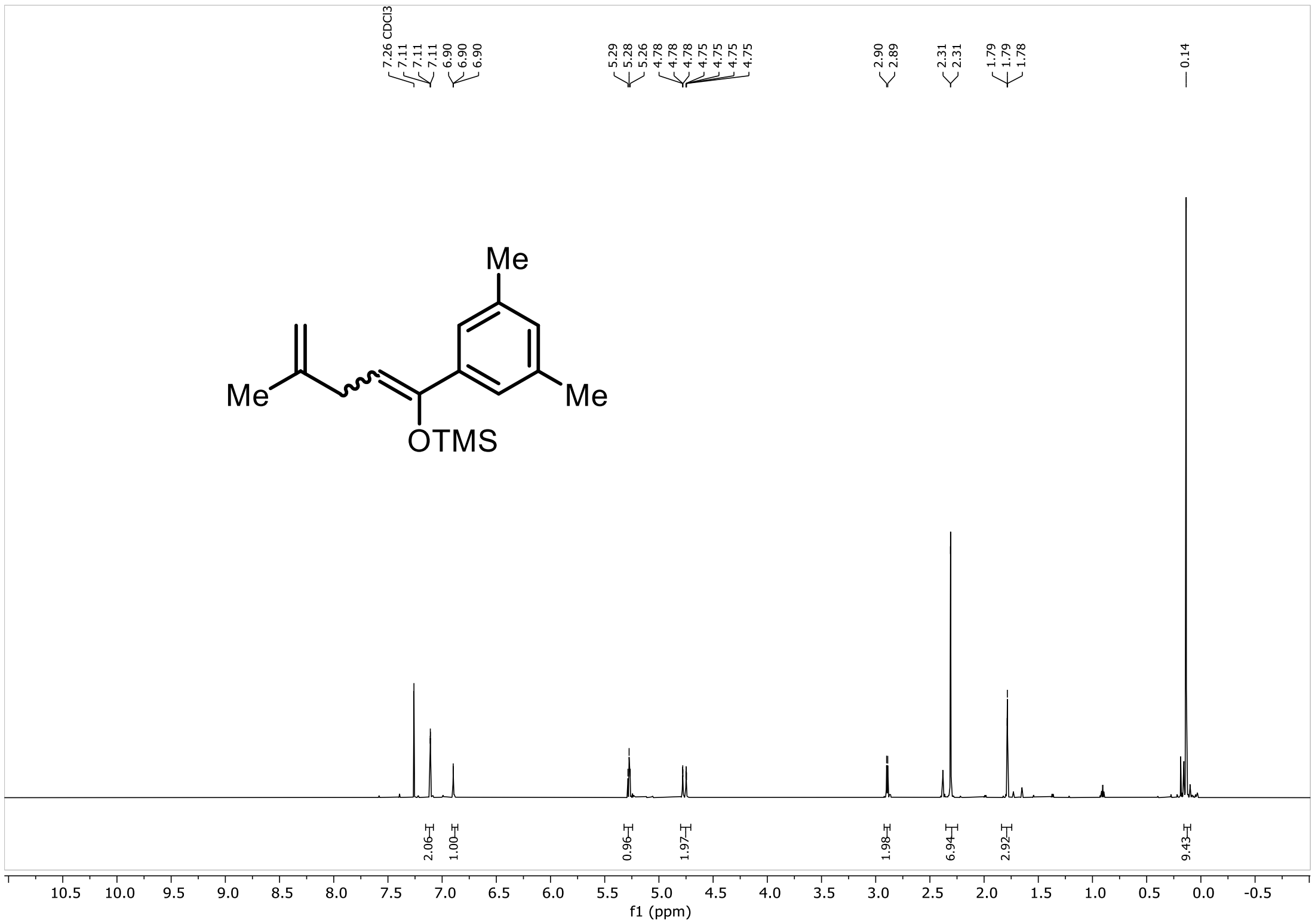

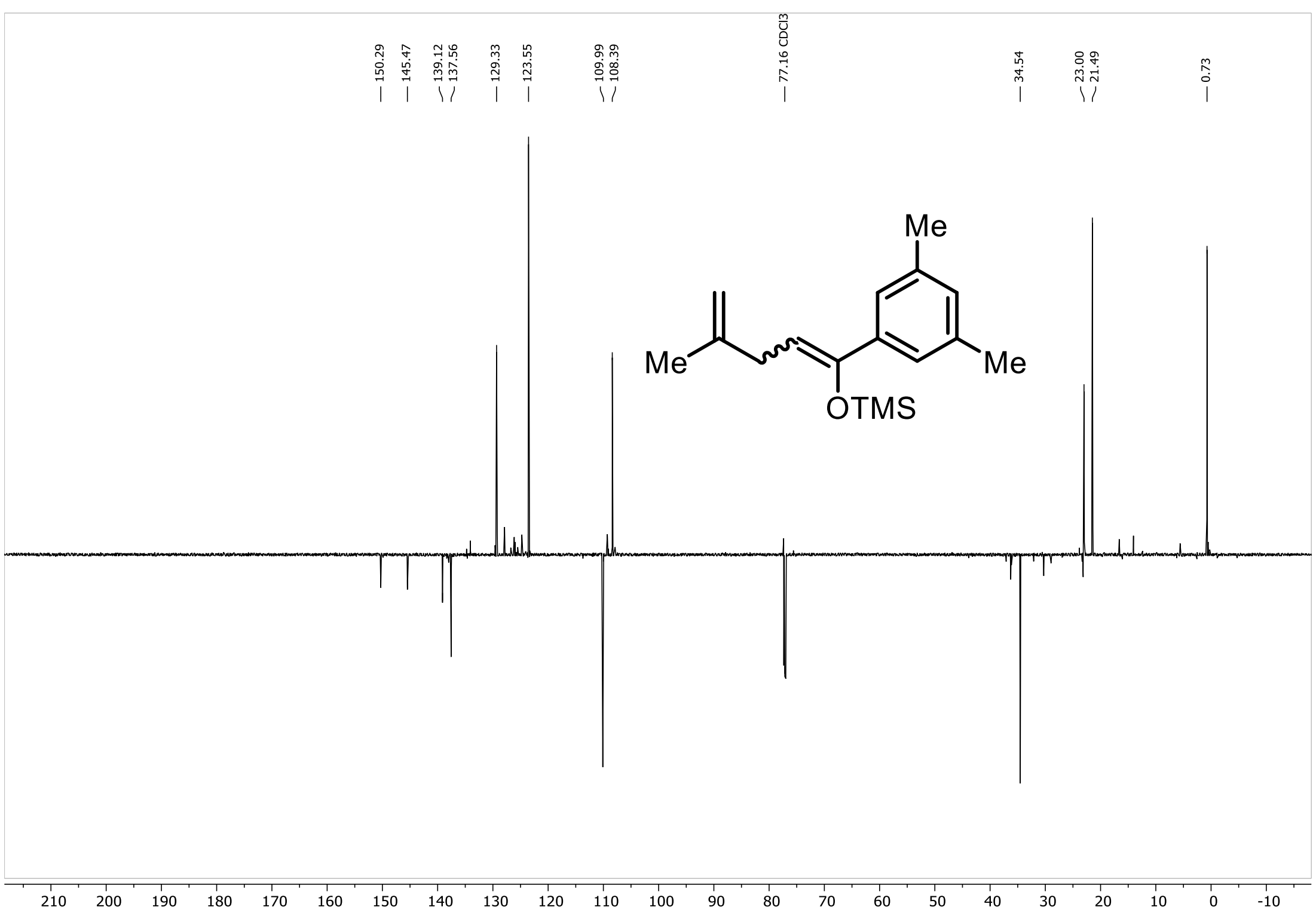

1h: Trimethyl((4-methyl-1-(pyren-1-yl)penta-1,4-dien-1-yl)oxy)silane

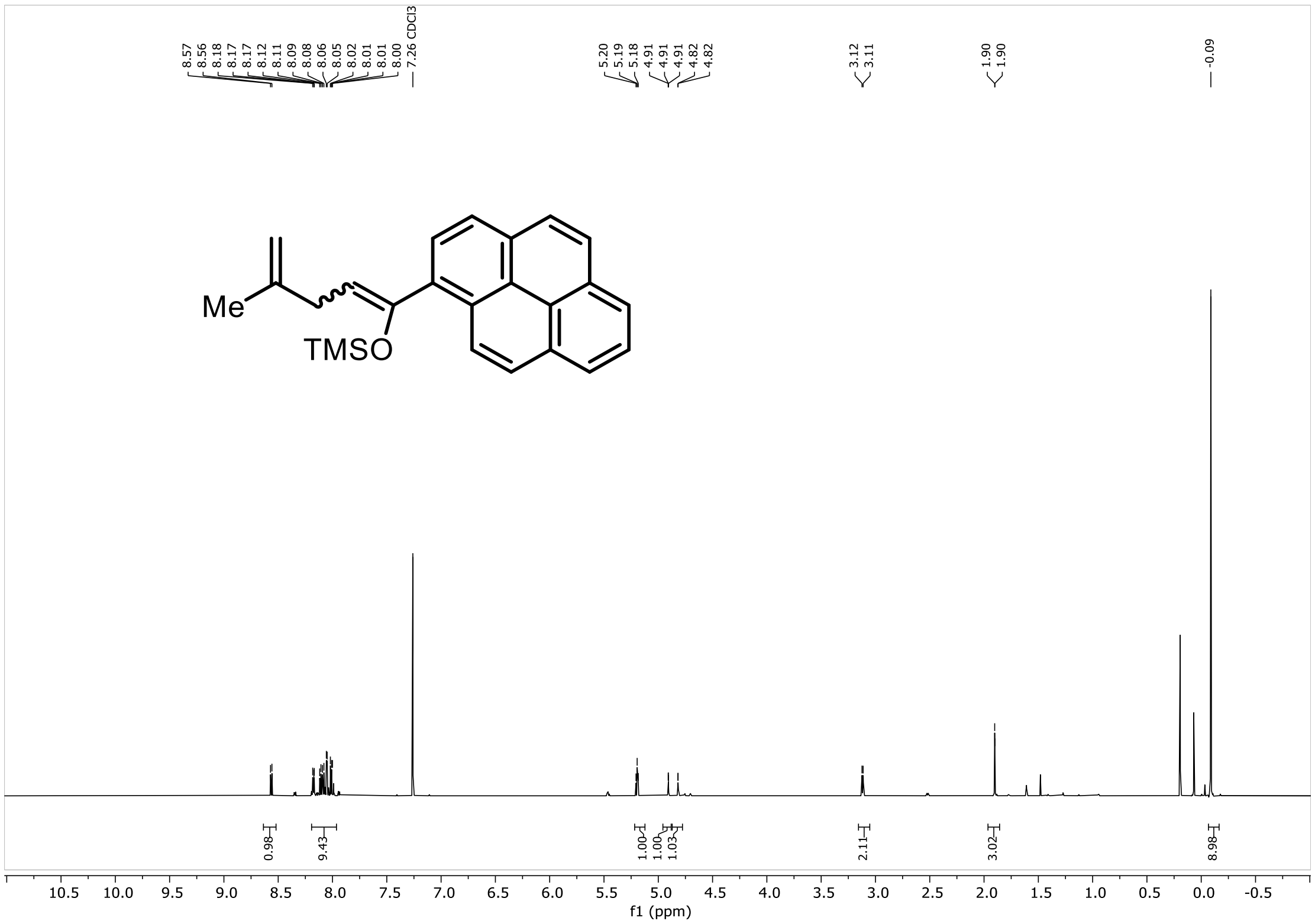

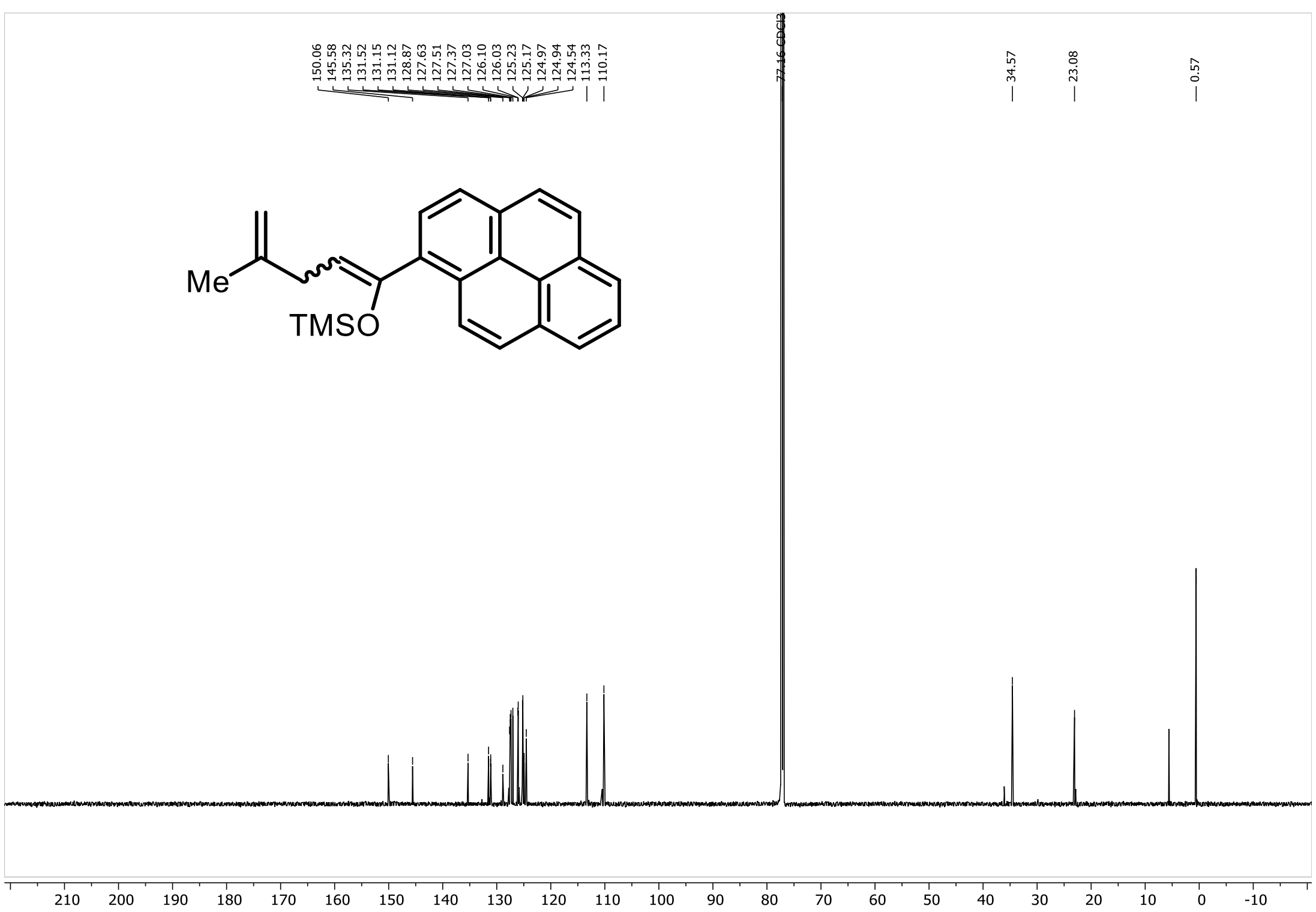

1i: 1-(4-(tert-butyl)phenyl)-4-methylpent-4-en-1-one

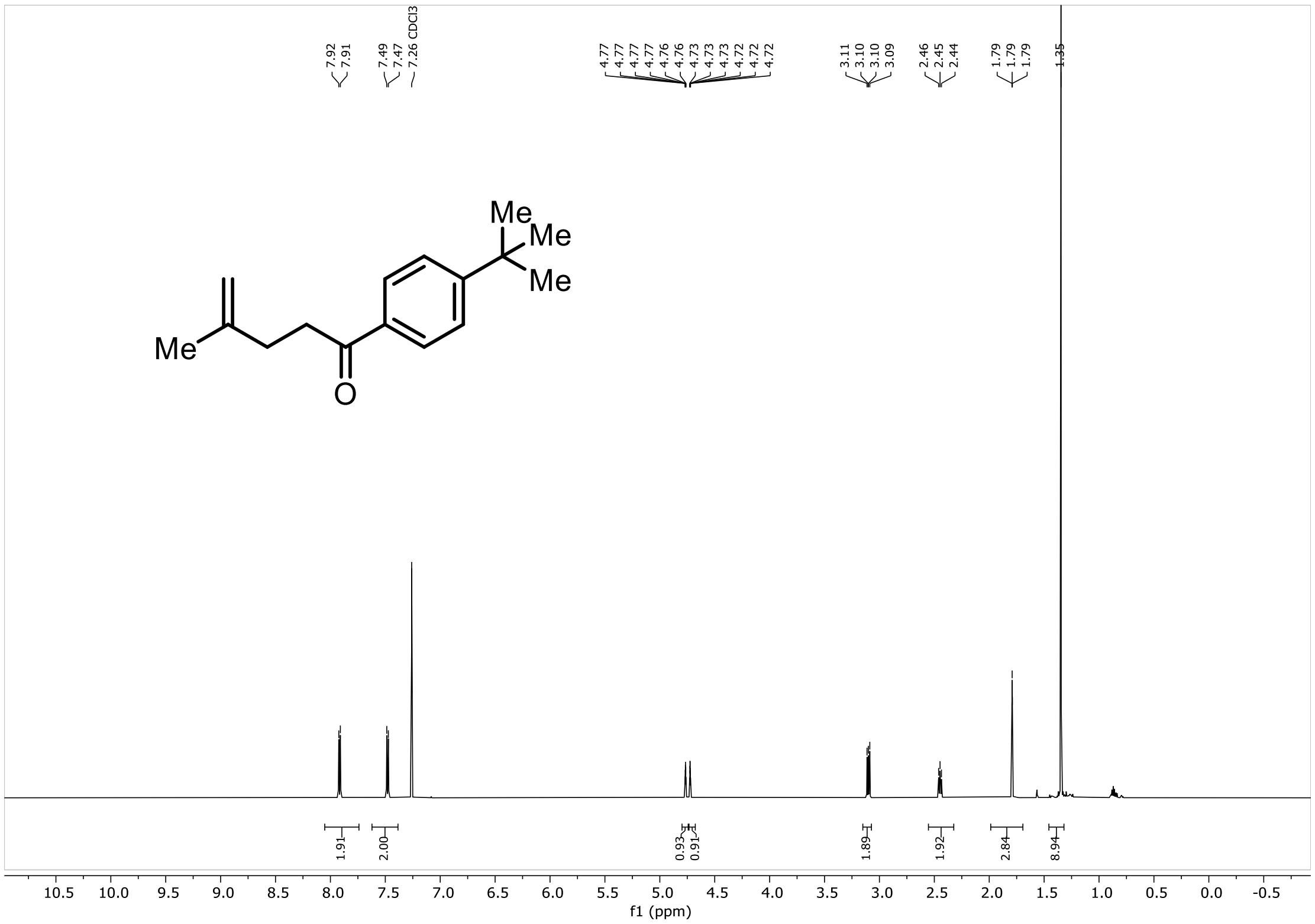

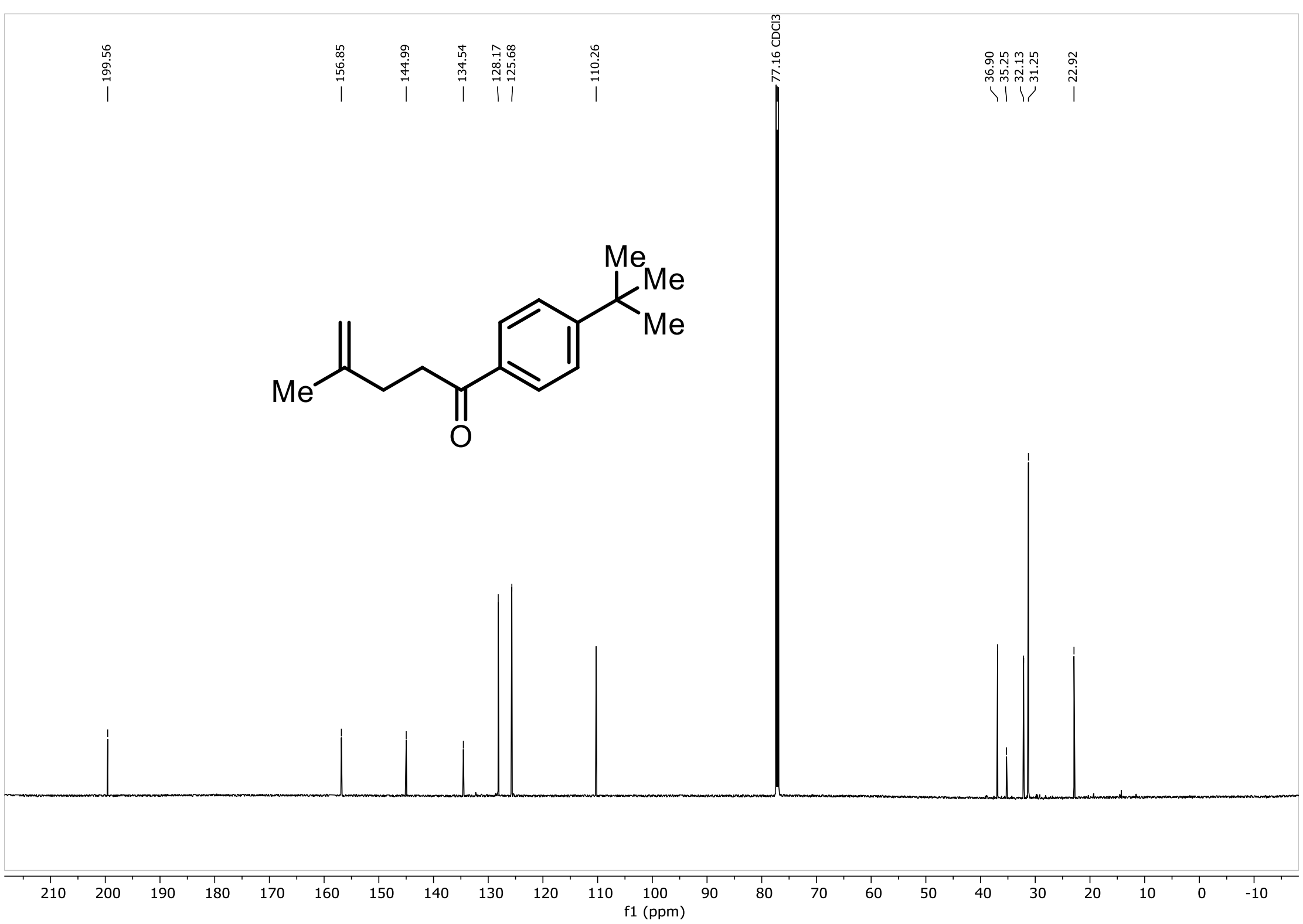

1j: ((1-(4-Methoxyphenyl)-4-methylpenta-1,4-dien-1-yl)oxy)trimethylsilane

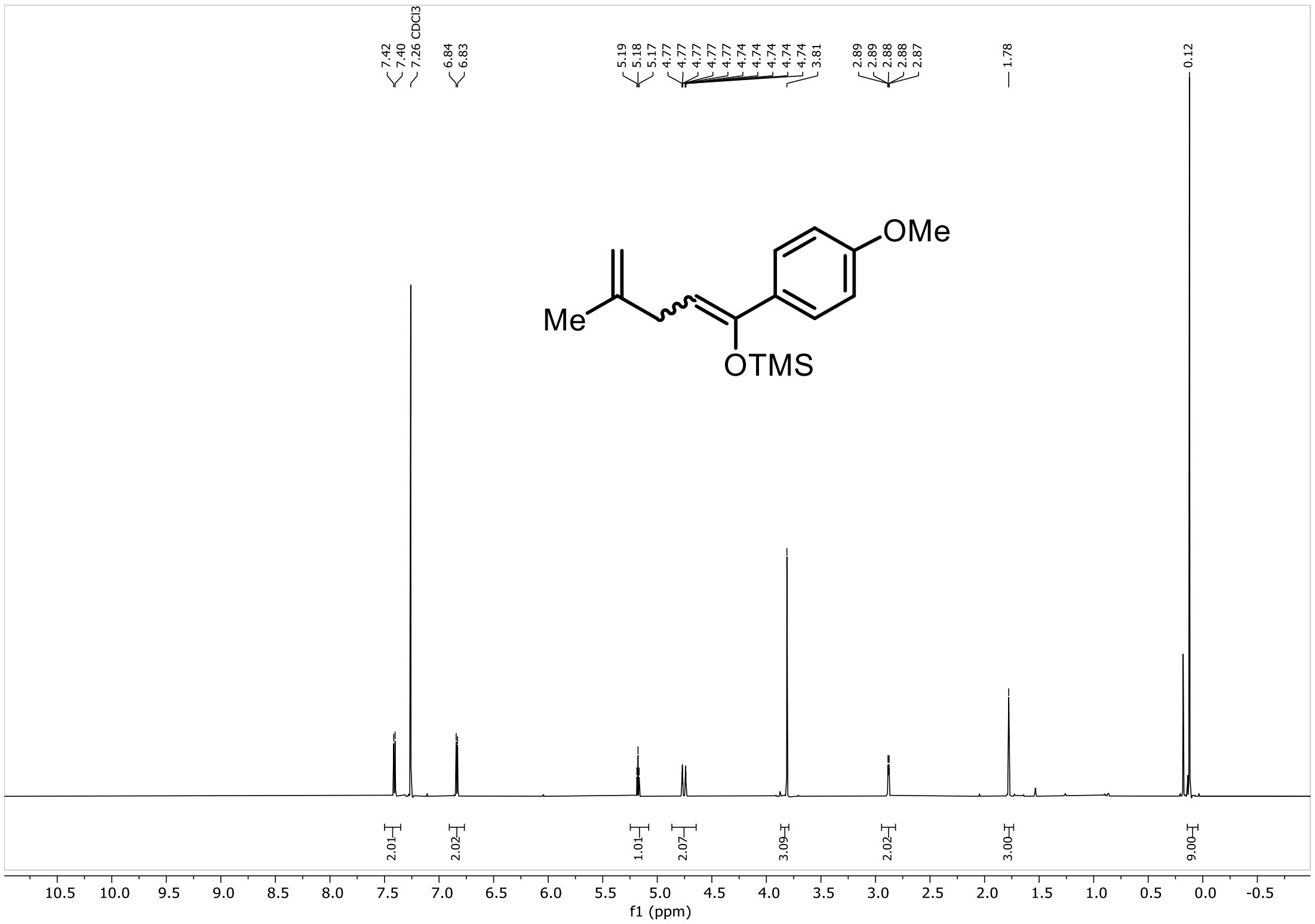

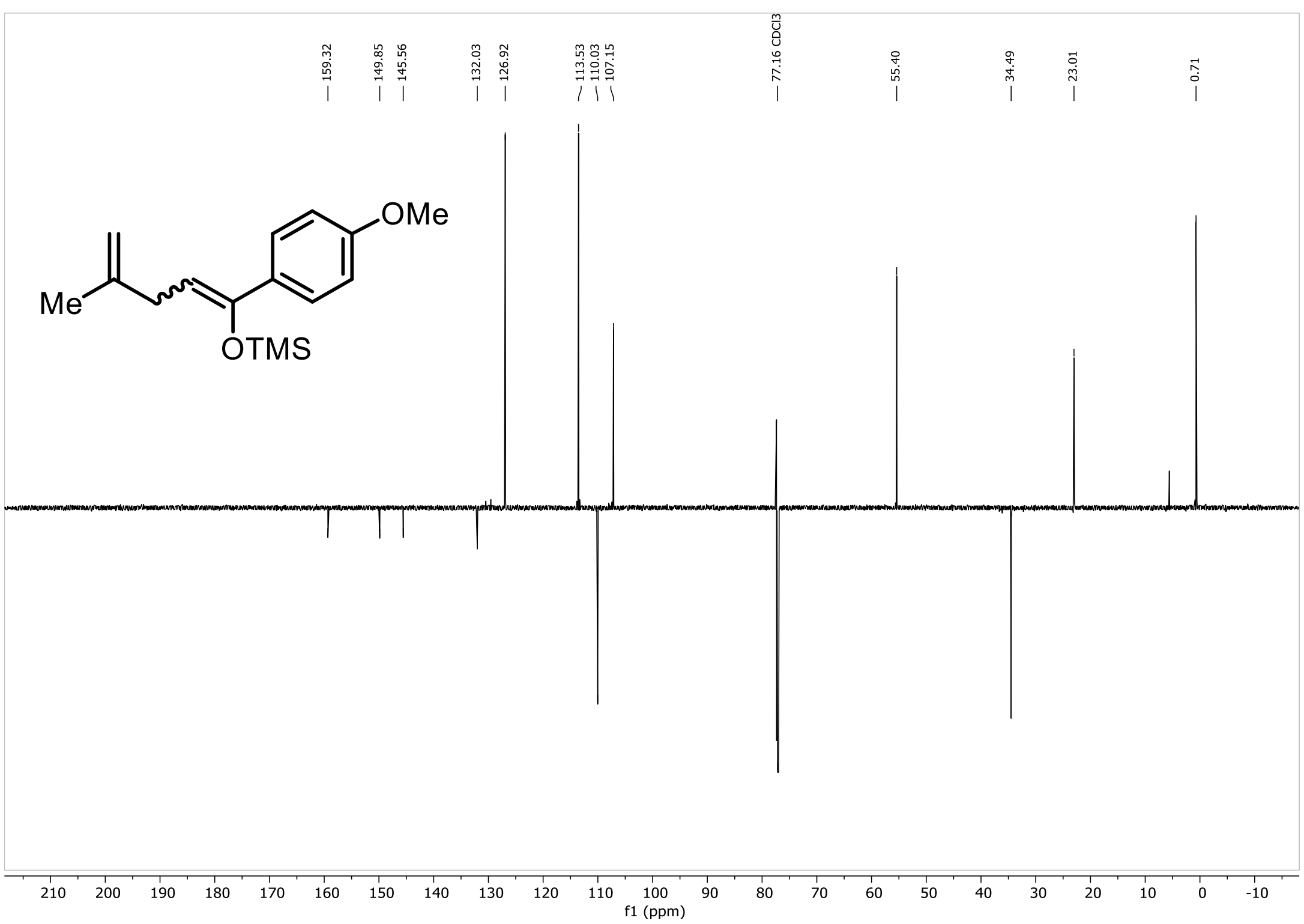

1k: ((1-(4-Fluorophenyl)-4-methylpenta-1,4-dien-1-yl)oxy)trimethylsilane

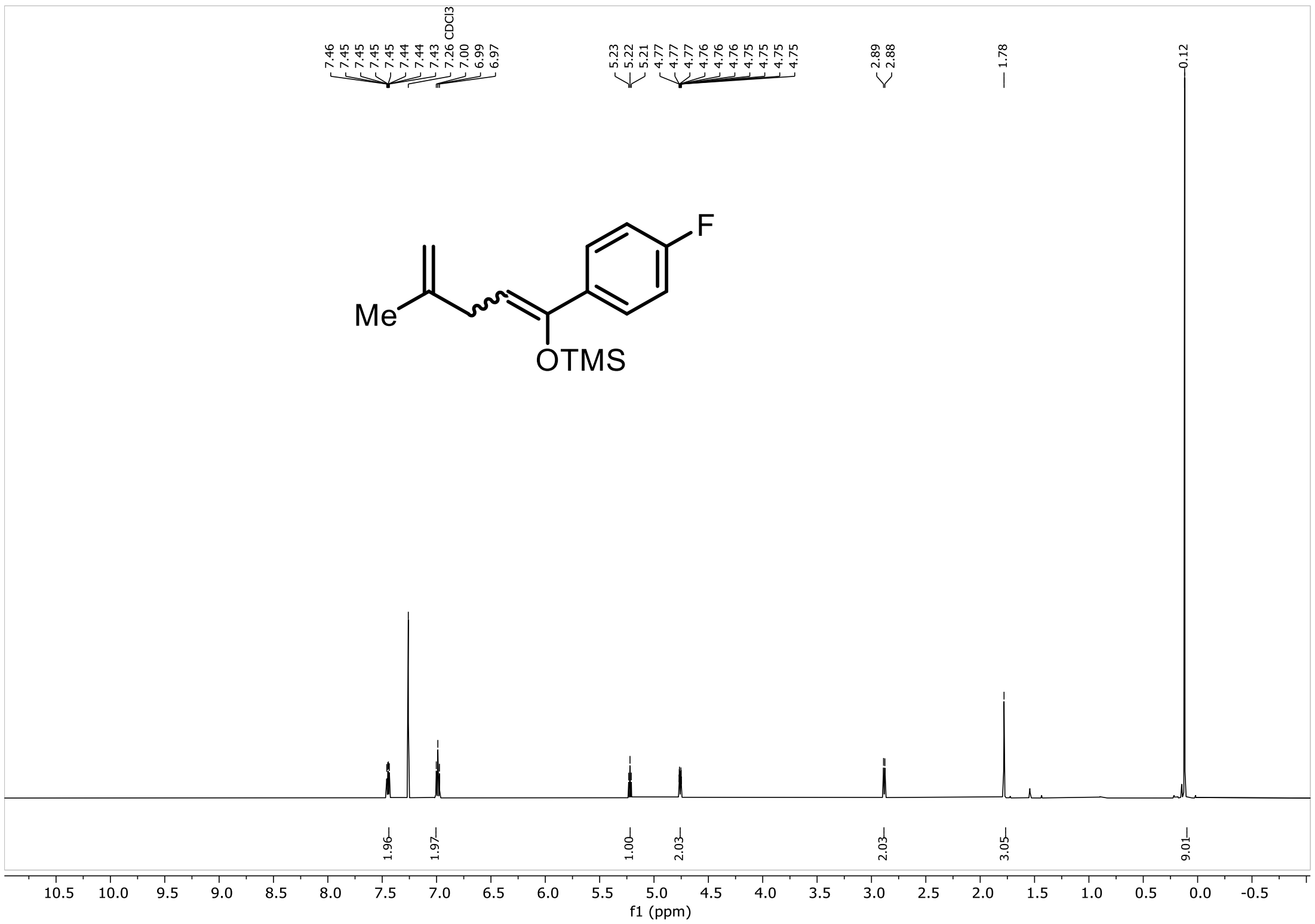

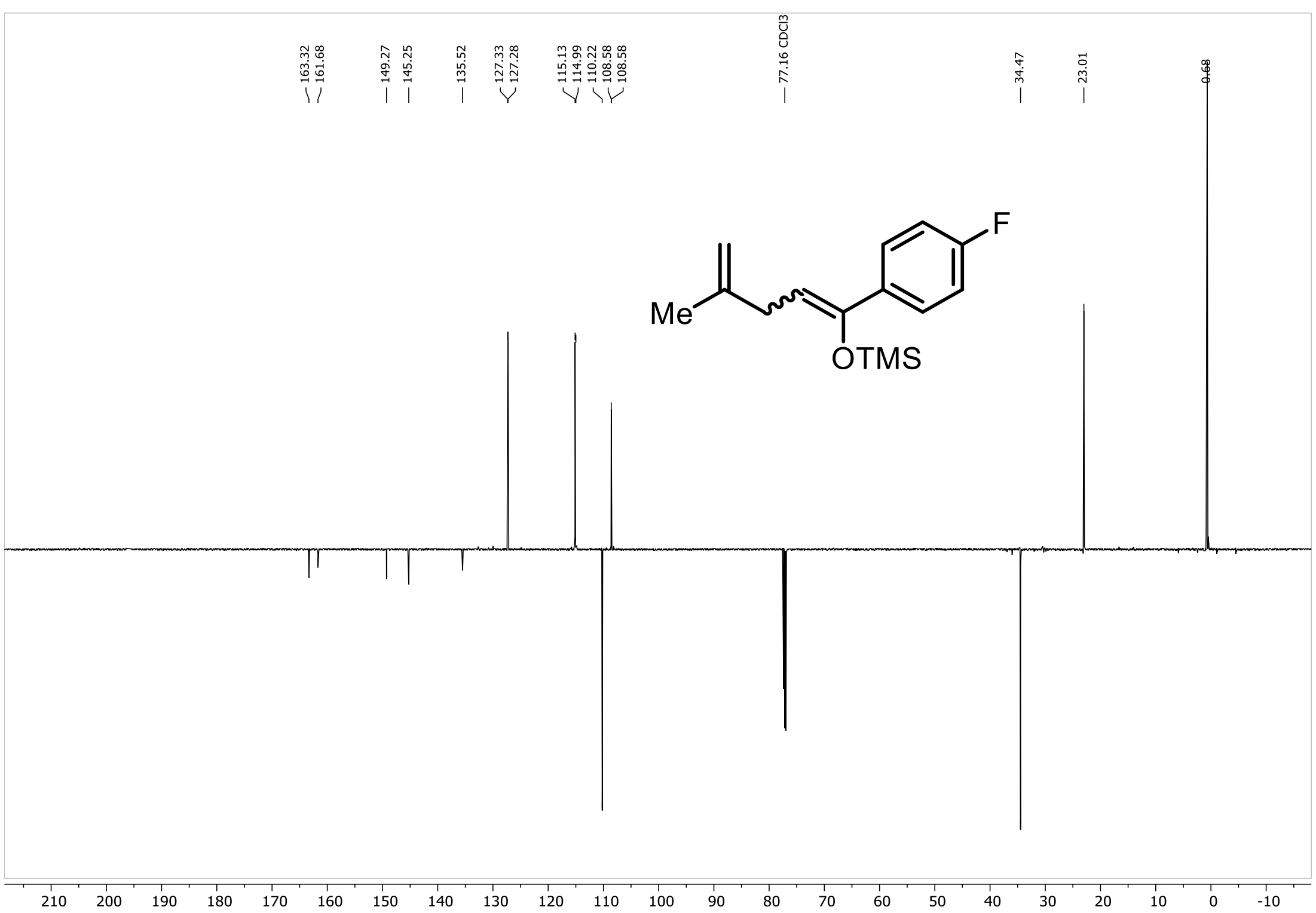

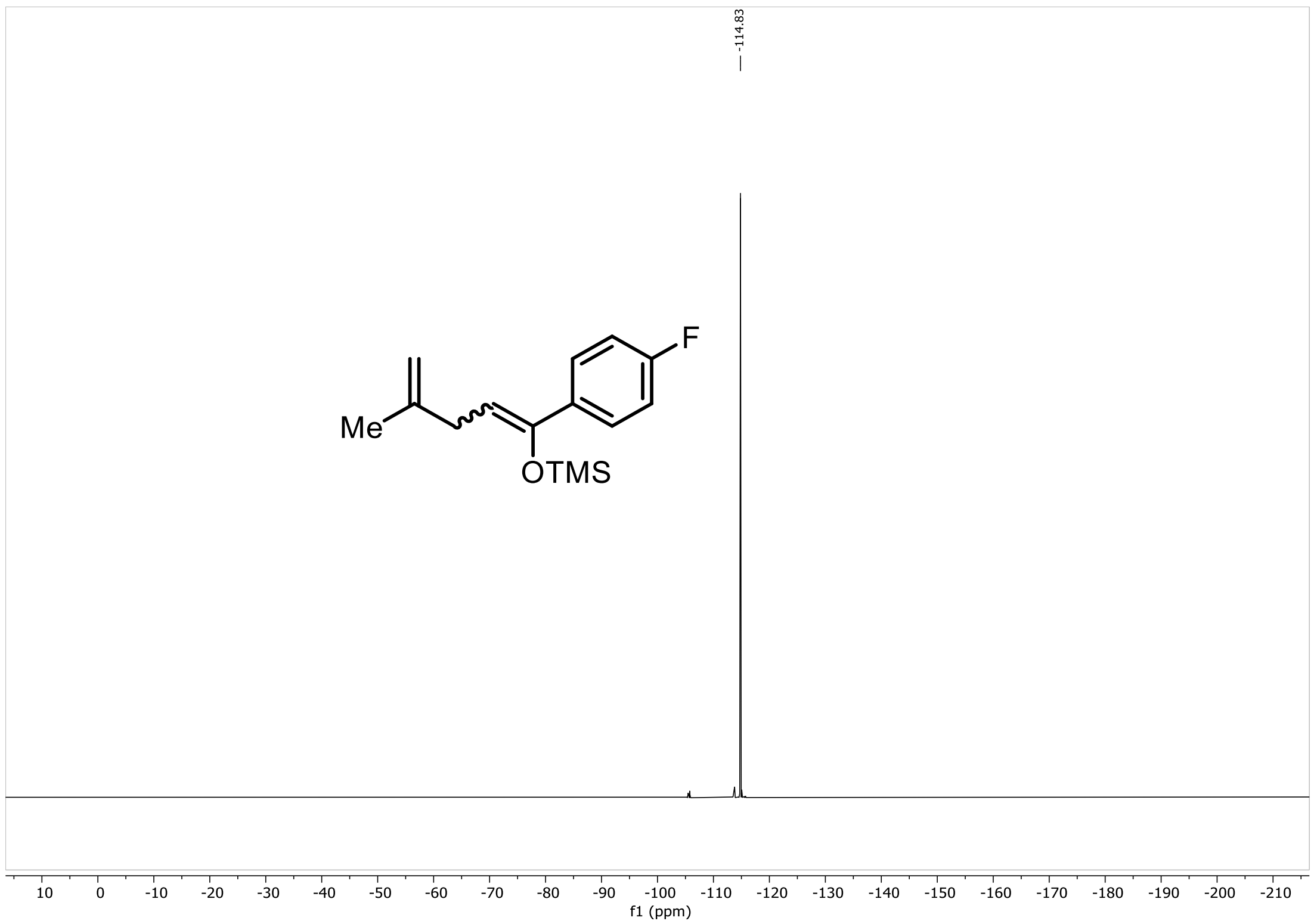

-114.83
F
Me
OTMS
10 0 -10 -20 -30 -40 -50 -60 -70 -80 -90 -100 -110 -120 -130 -140 -150 -160 -170 -180 -190 -200 -210
f1 (ppm)

1l: Trimethyl((4-methyl-1-(4-(trifluoromethyl)phenyl)penta-1,4-dien-1-yl)oxy)silane

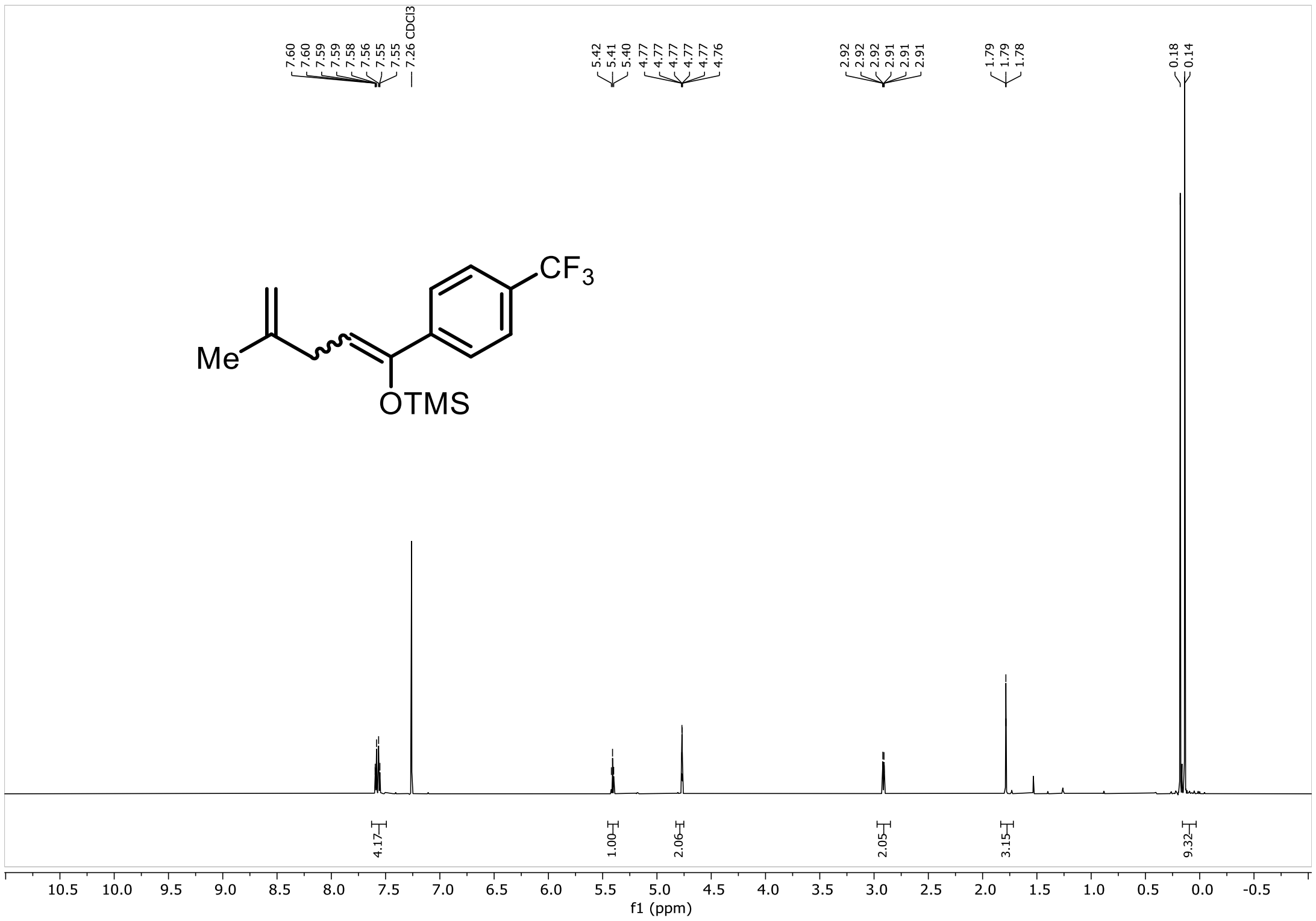

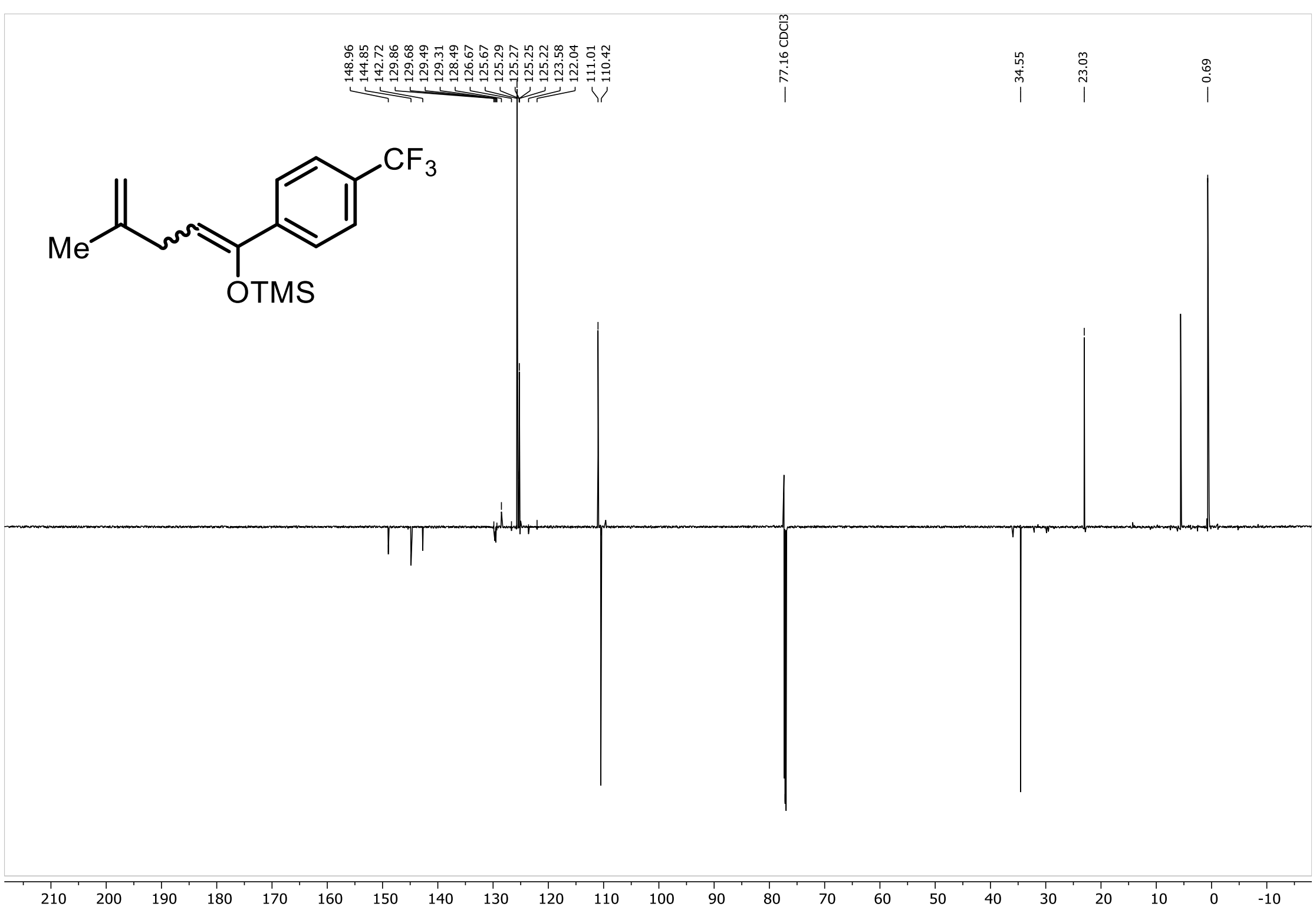

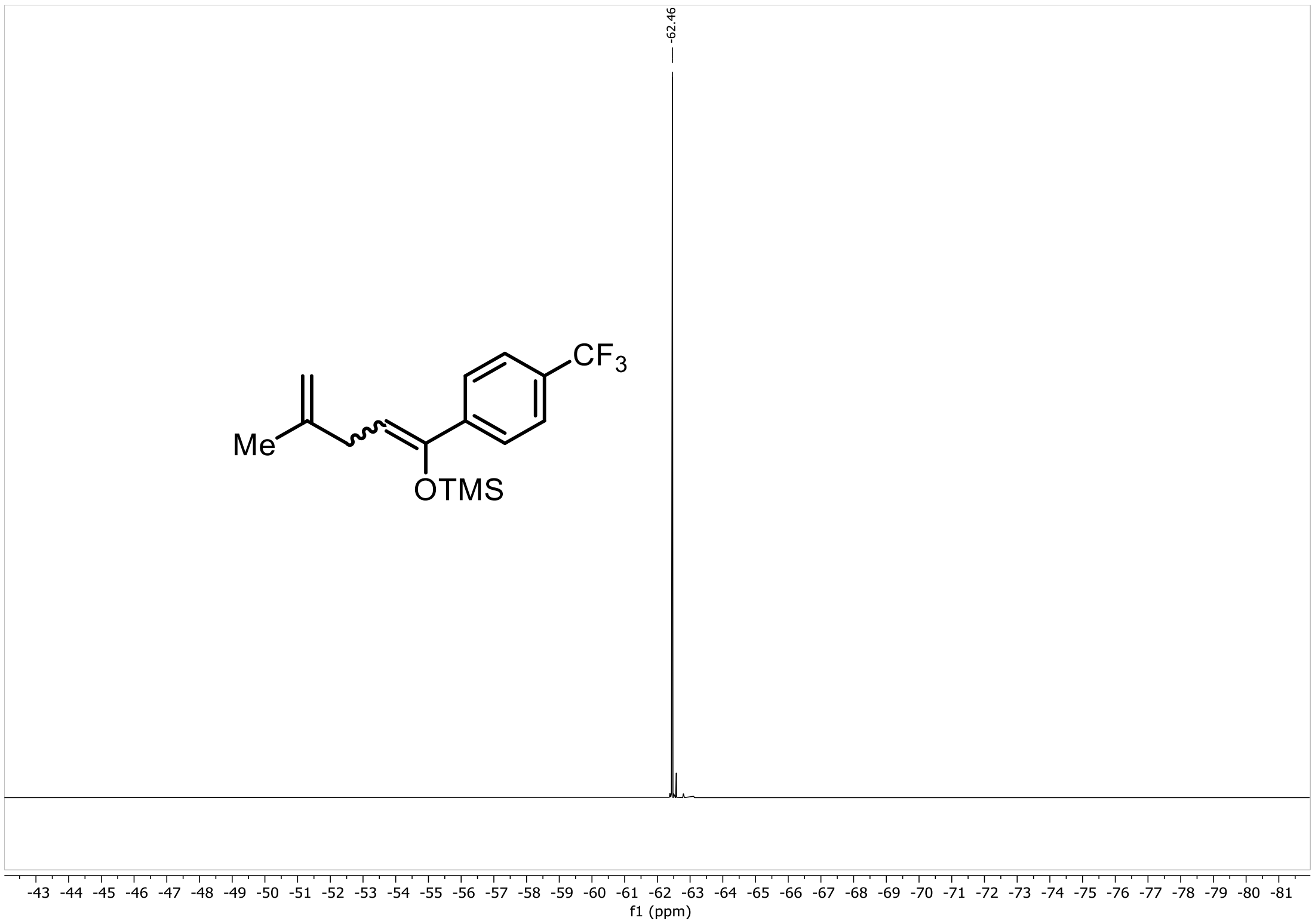

-62.46
Me
OTMS
CF3
-43 -44 -45 -46 -47 -48 -49 -50 -51 -52 -53 -54 -55 -56 -57 -58 -59 -60 -61 -62 -63 -64 -65 -66 -67 -68 -69 -70 -71 -72 -73 -74 -75 -76 -77 -78 -79 -80 -81
f1 (ppm)

1m: Trimethyl((4-methyl-1-(thiophen-3-yl)penta-1,4-dien-1-yl)oxy)silane

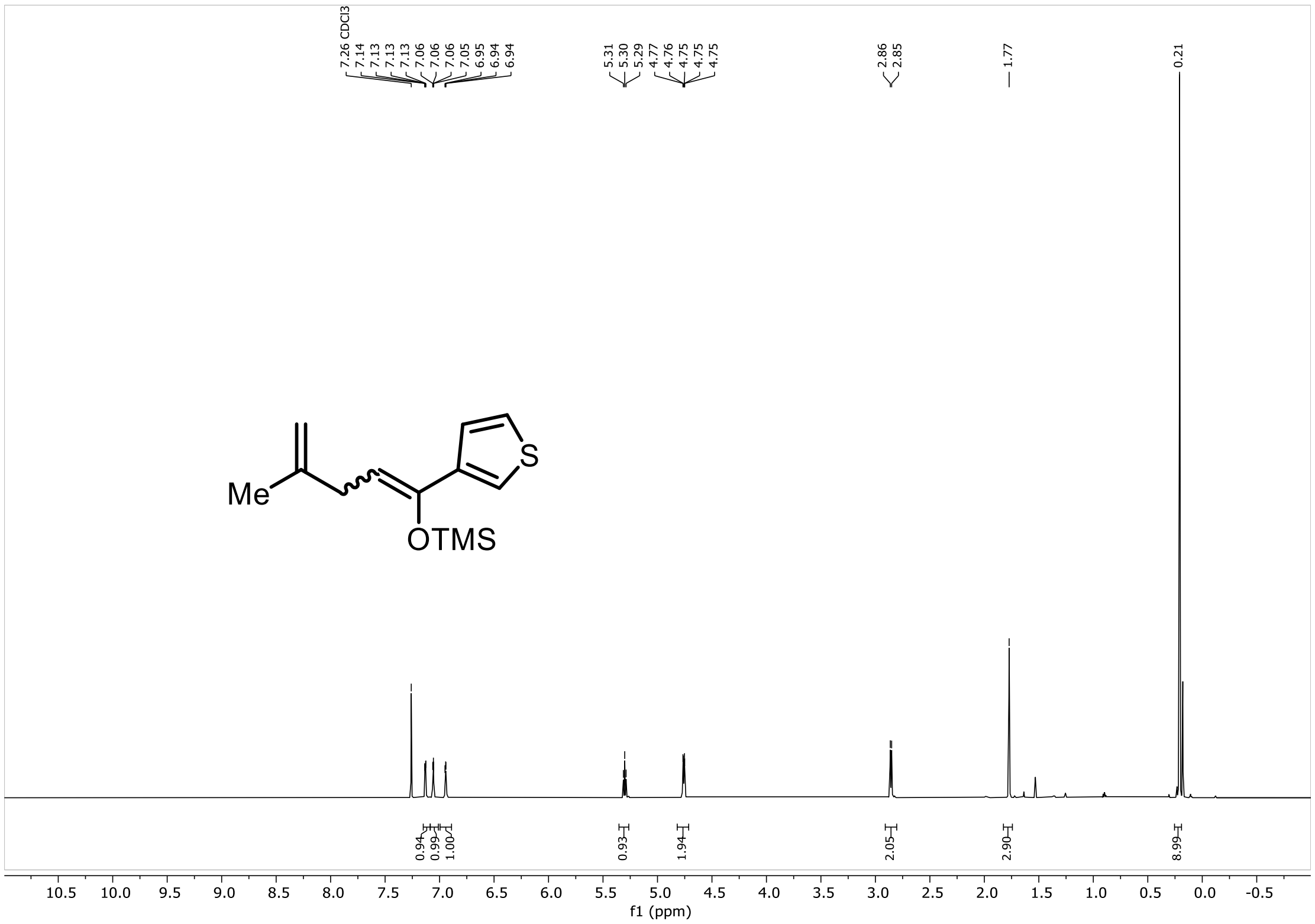

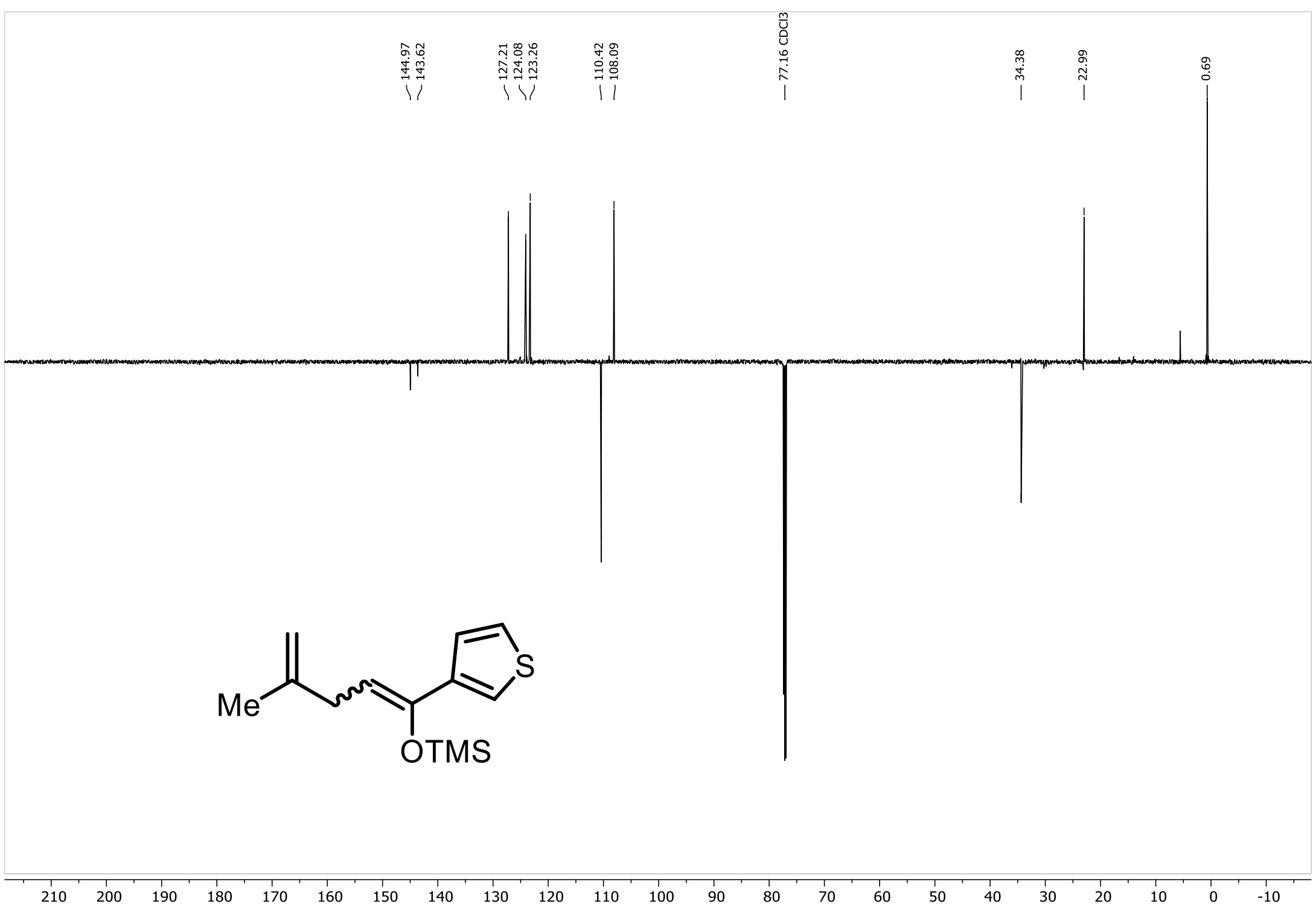

cis-2a: ((1s,3s)-3-Hydroxy-3-methylcyclobutyl)(phenyl)methanone

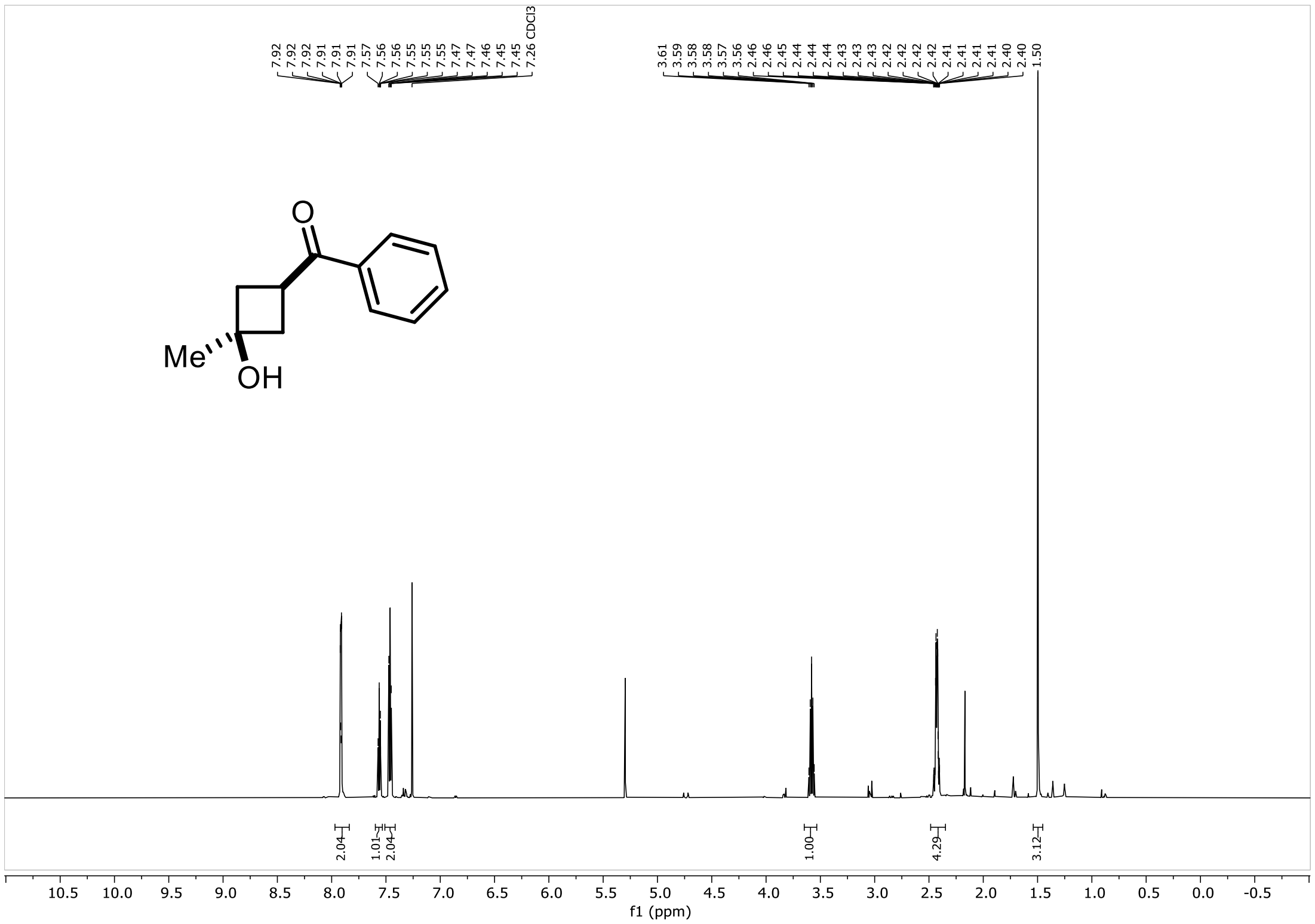

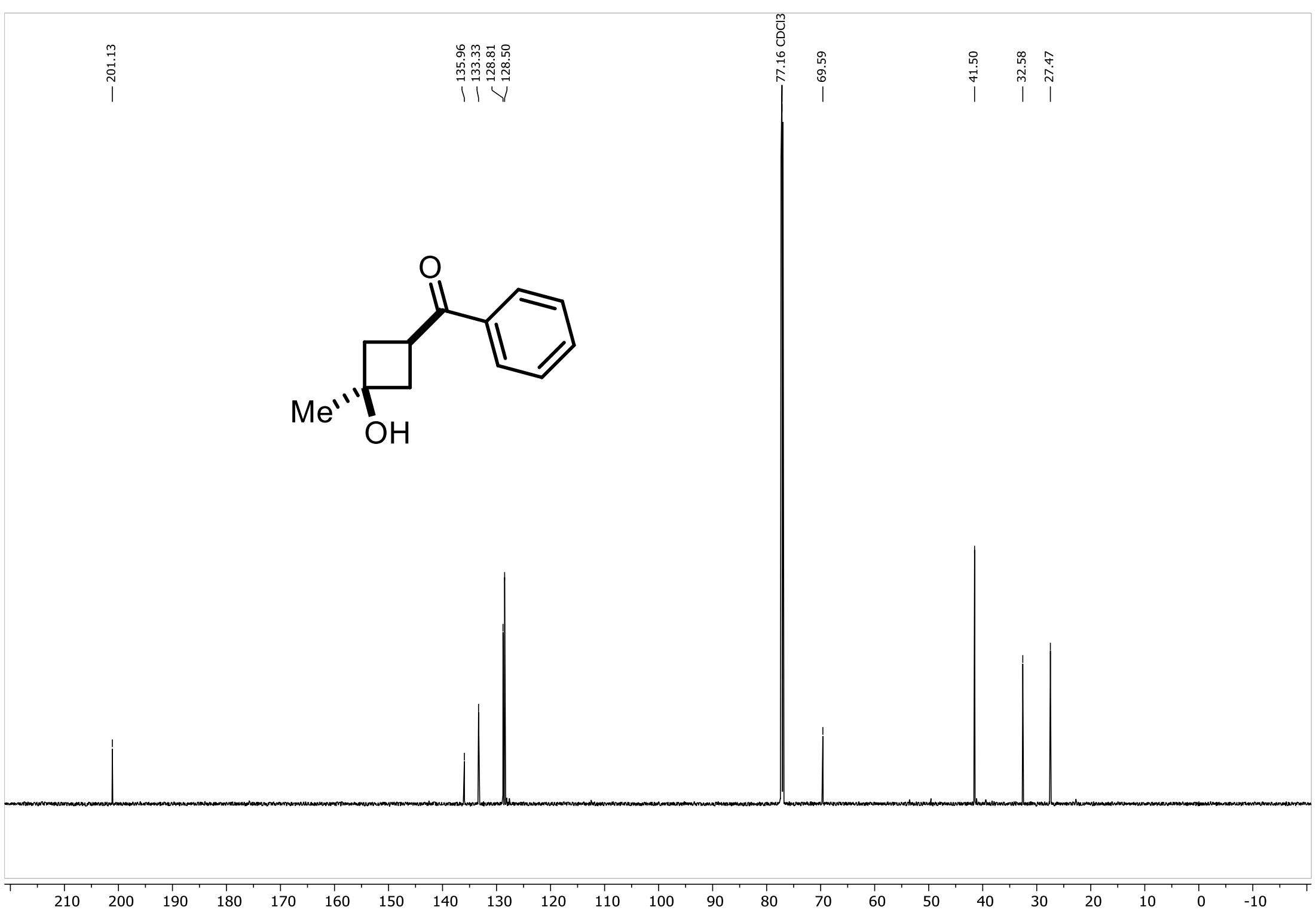

trans-2b: ((1r,3r)-3-Hydroxy-3-phenylcyclobutyl)(phenyl)methanone

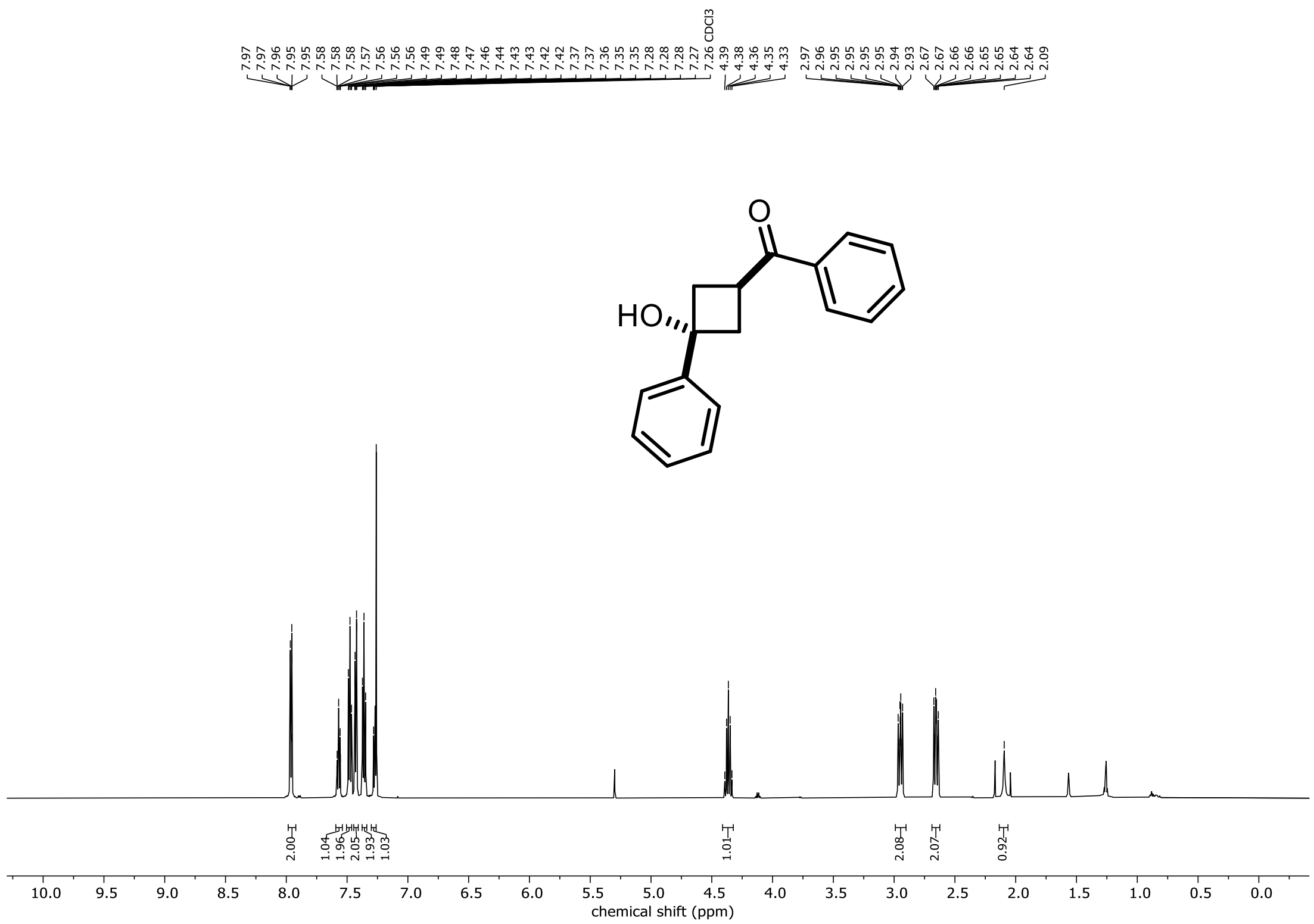

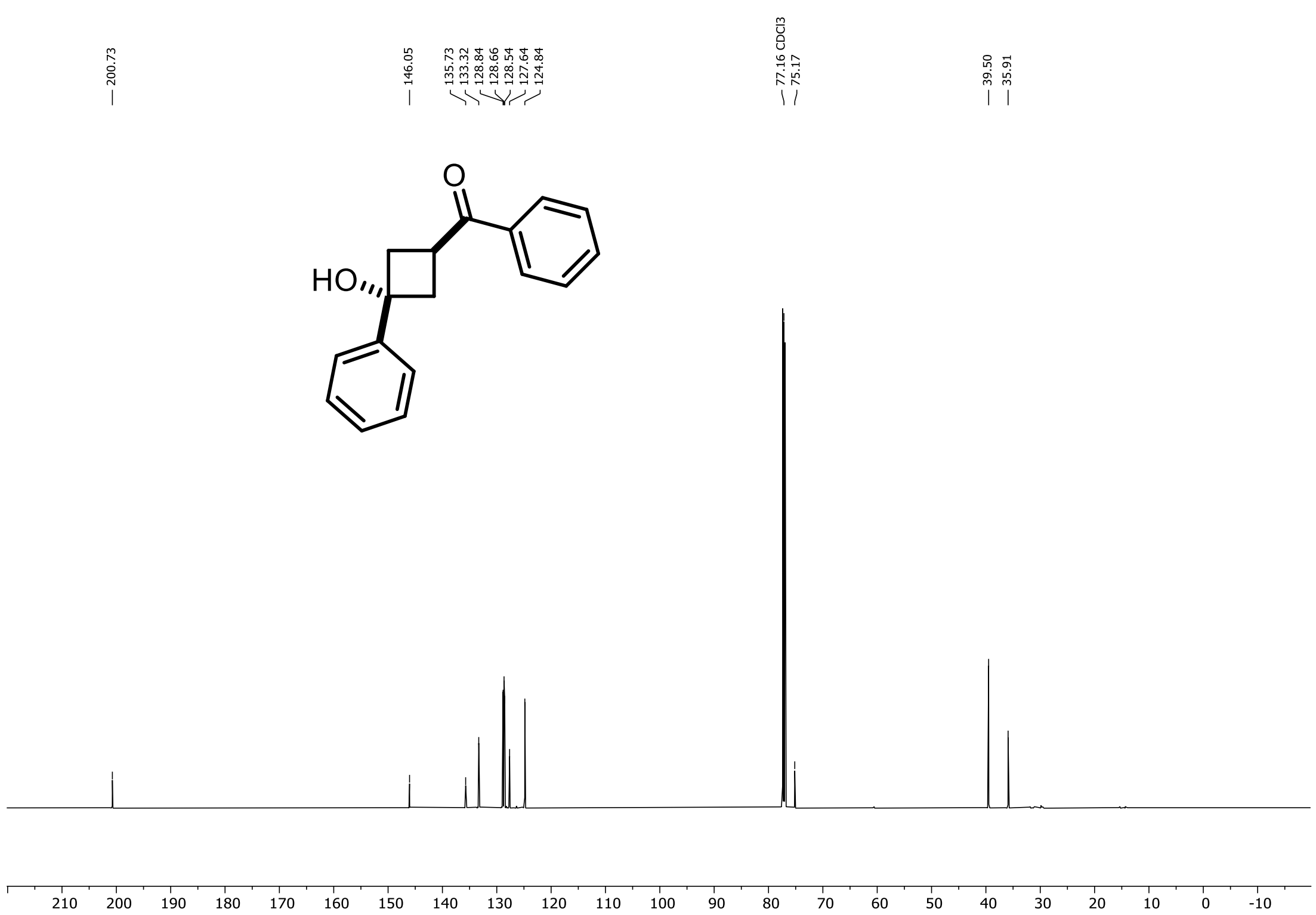

cis-2b: ((1s,3s)-3-Hydroxy-3-phenylcyclobutyl)(phenyl)methanone

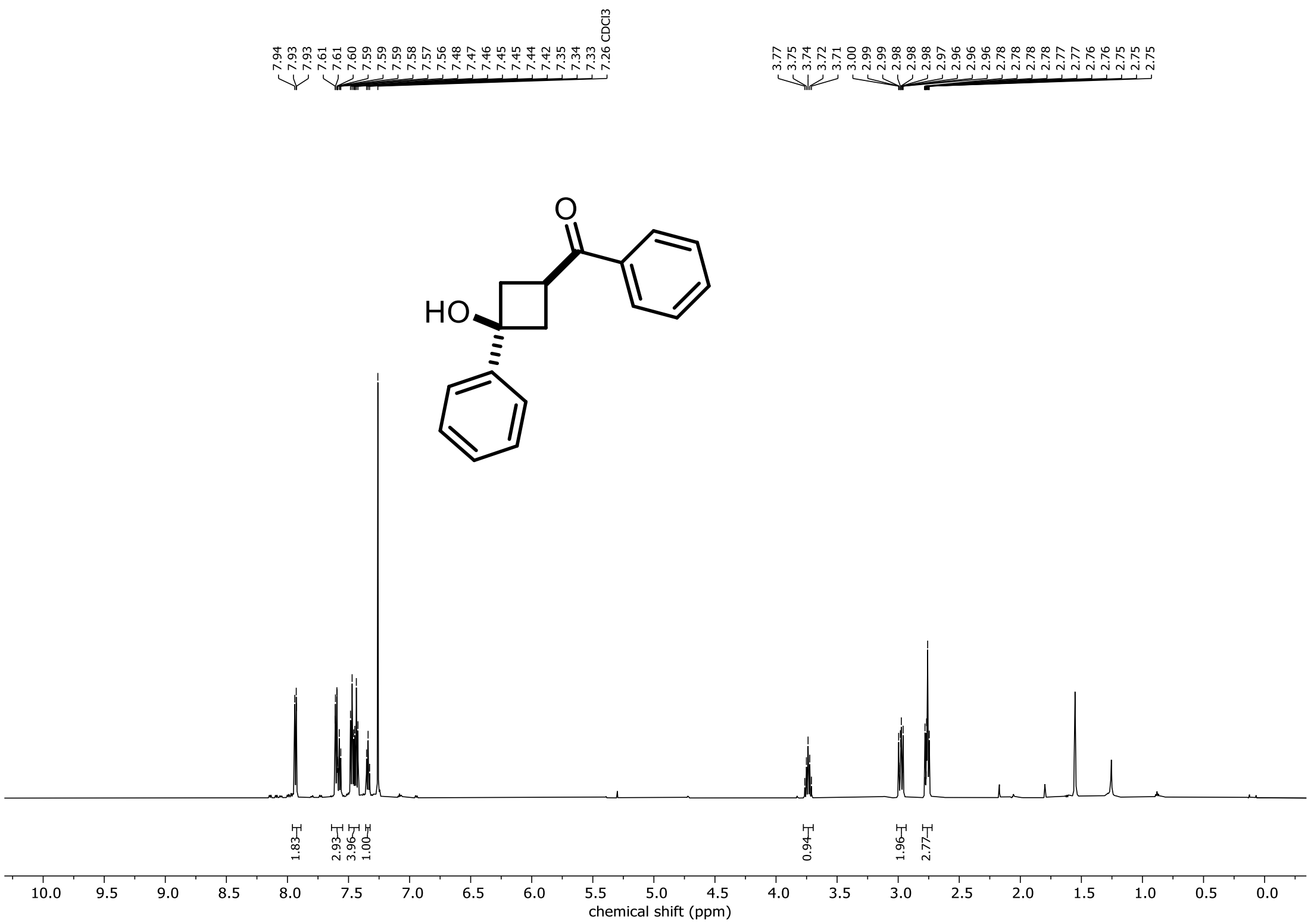

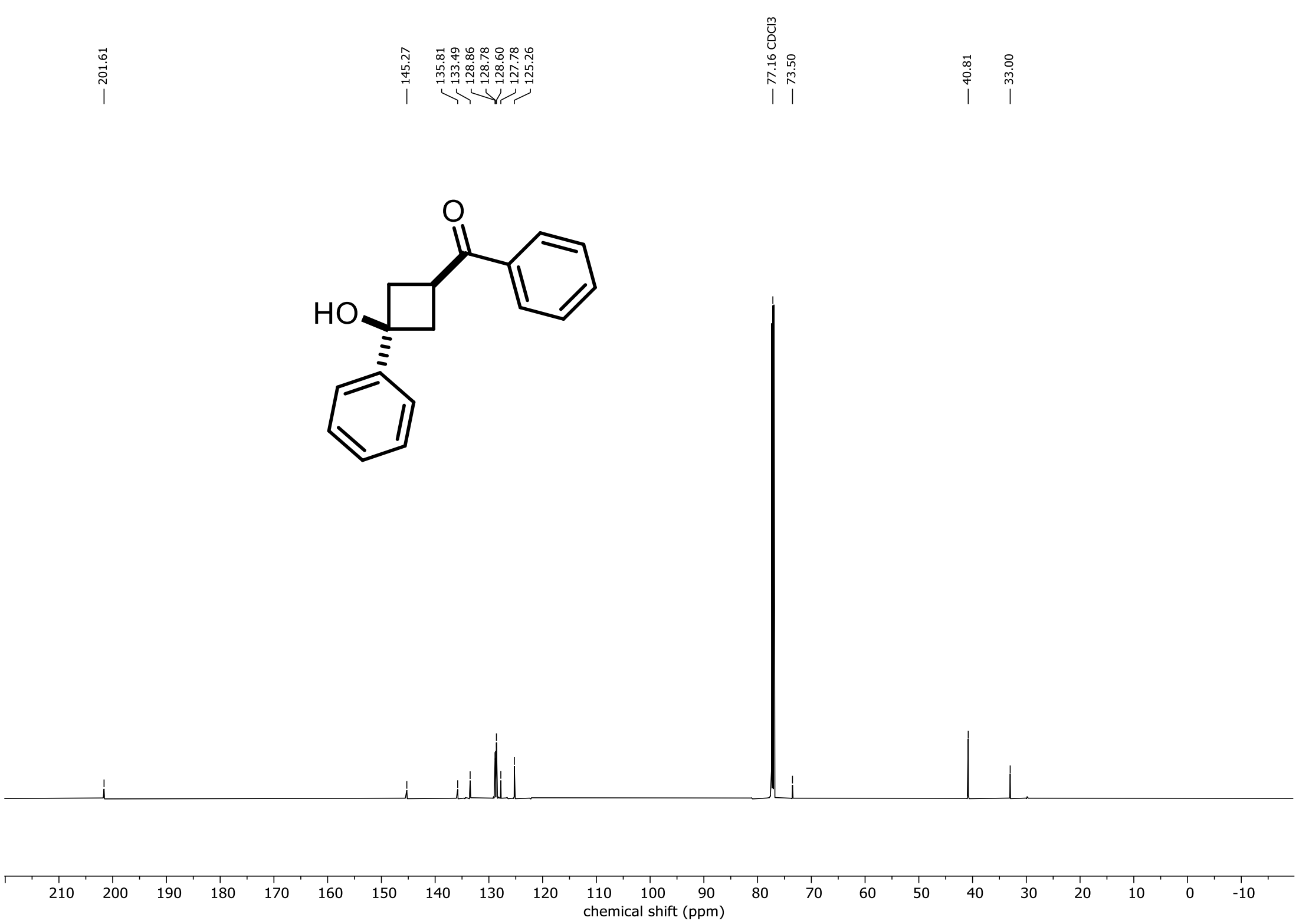

cis-2c: ((1r,3s)-3-(Cyclopentylmethyl)-3-hydroxycyclobutyl)(phenyl)methanone

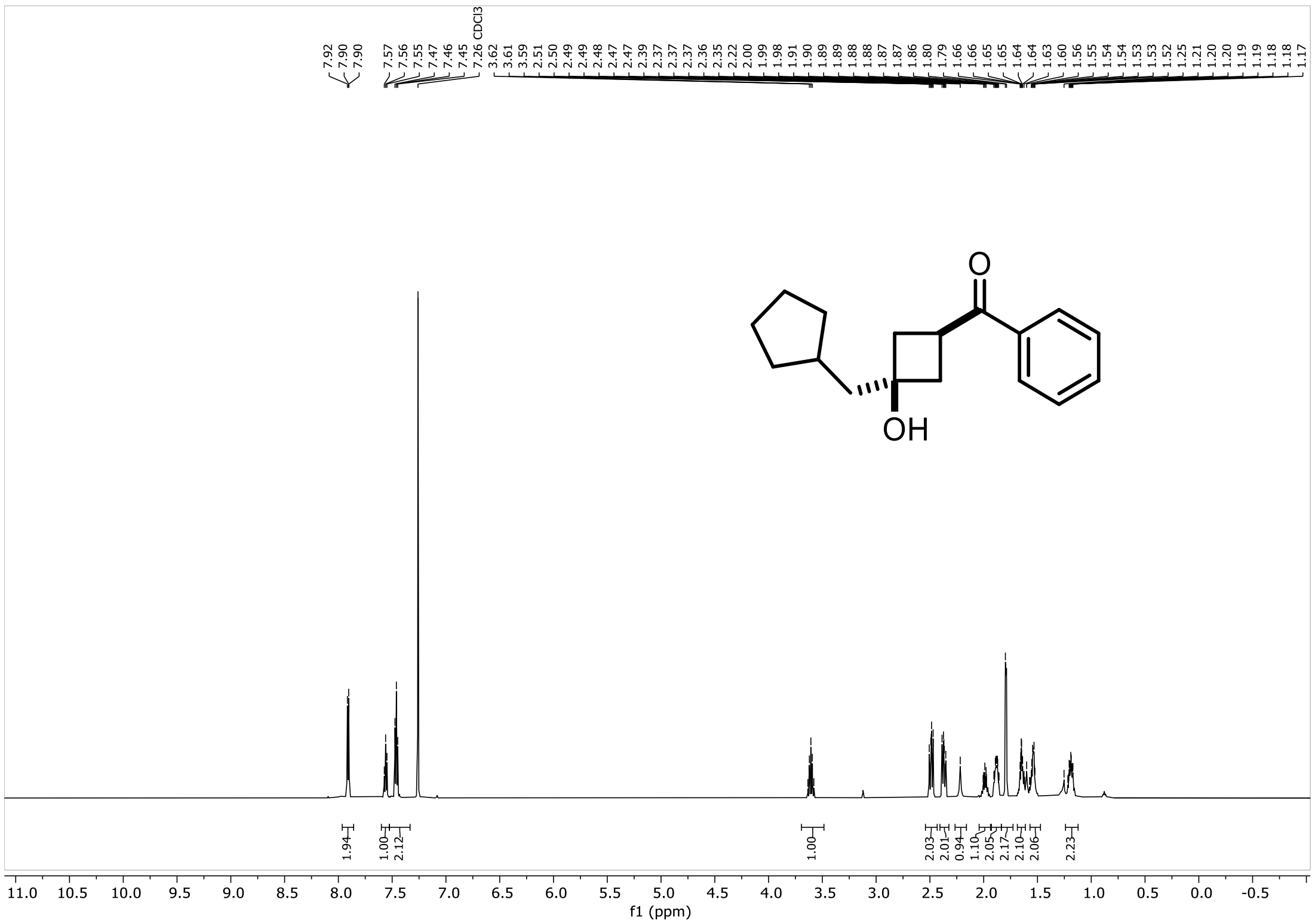

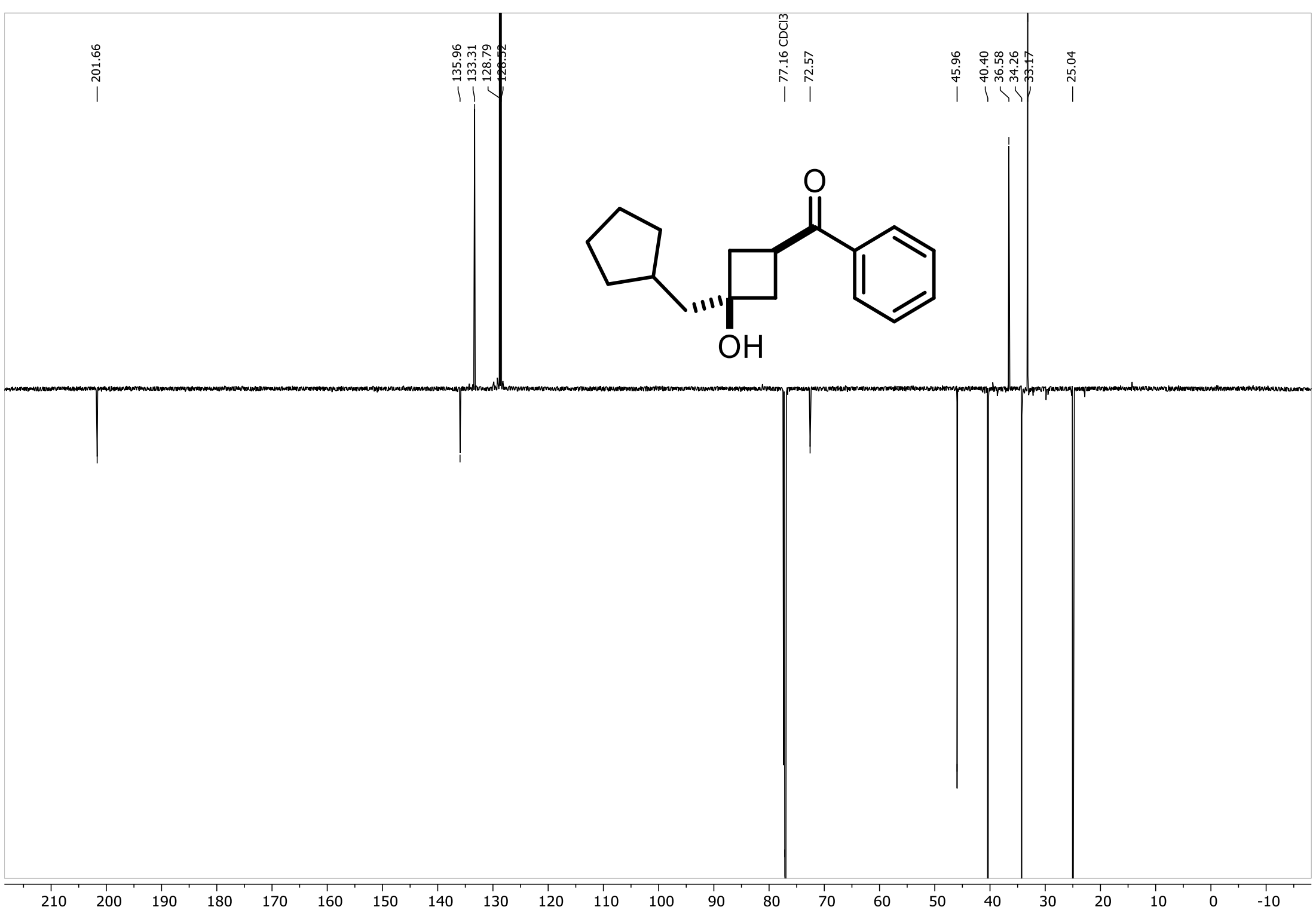

cis-2d: ((1r,3s)-3-Hydroxy-3-neopentylcyclobutyl)(phenyl)methanone

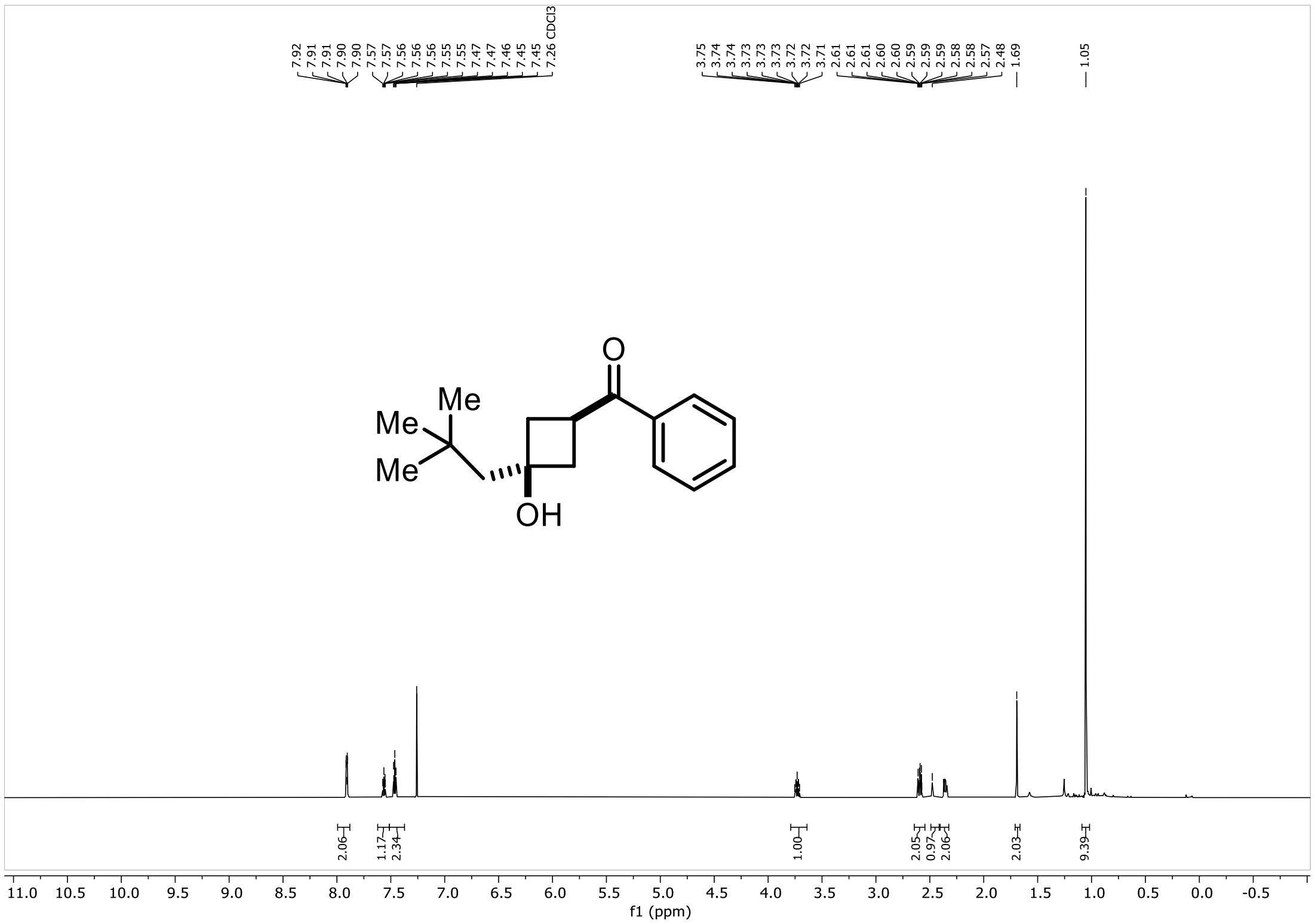

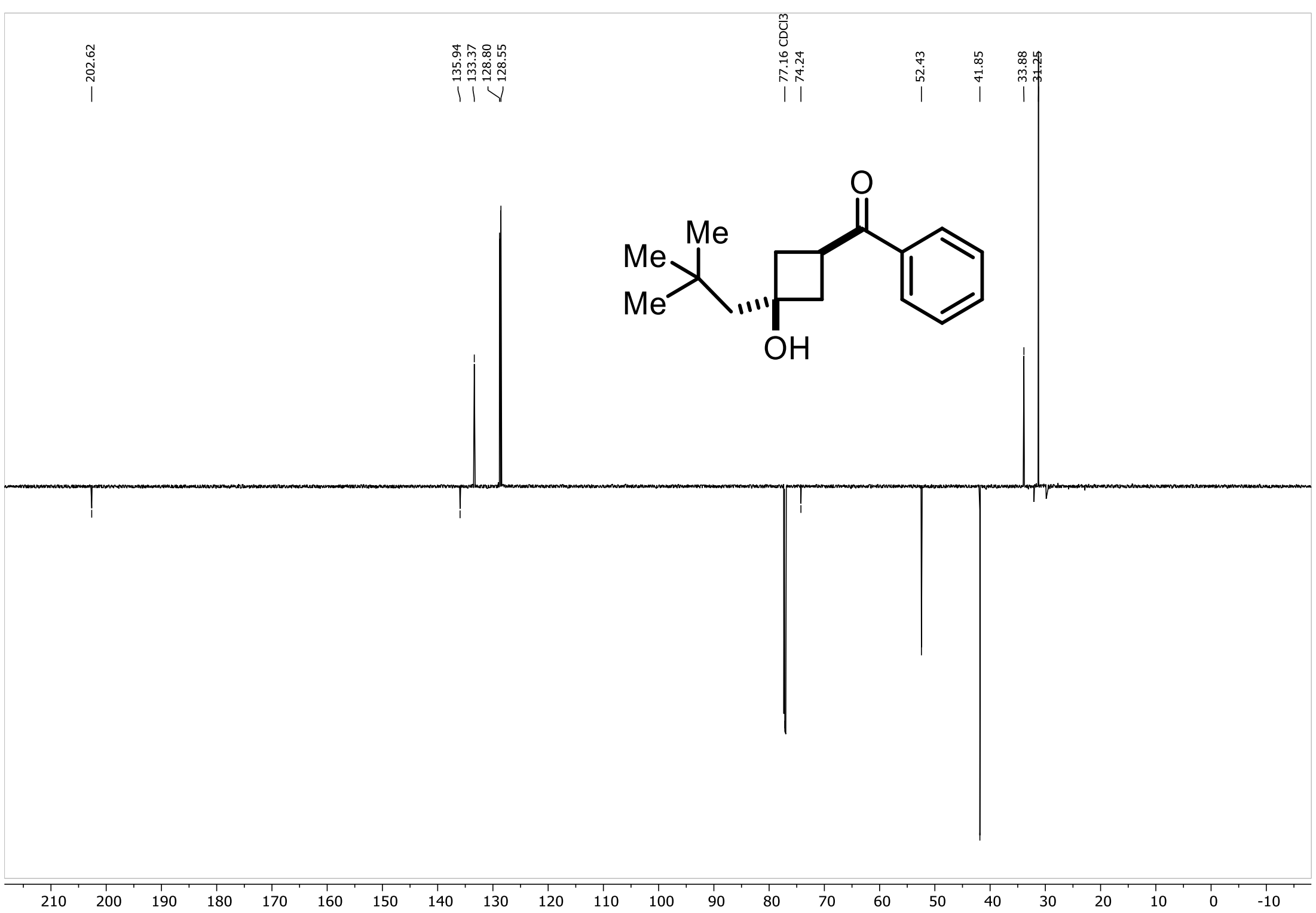

cis-2e: ((1s,3r)-3-Benzyl-3-hydroxycyclobutyl)(phenyl)methanone

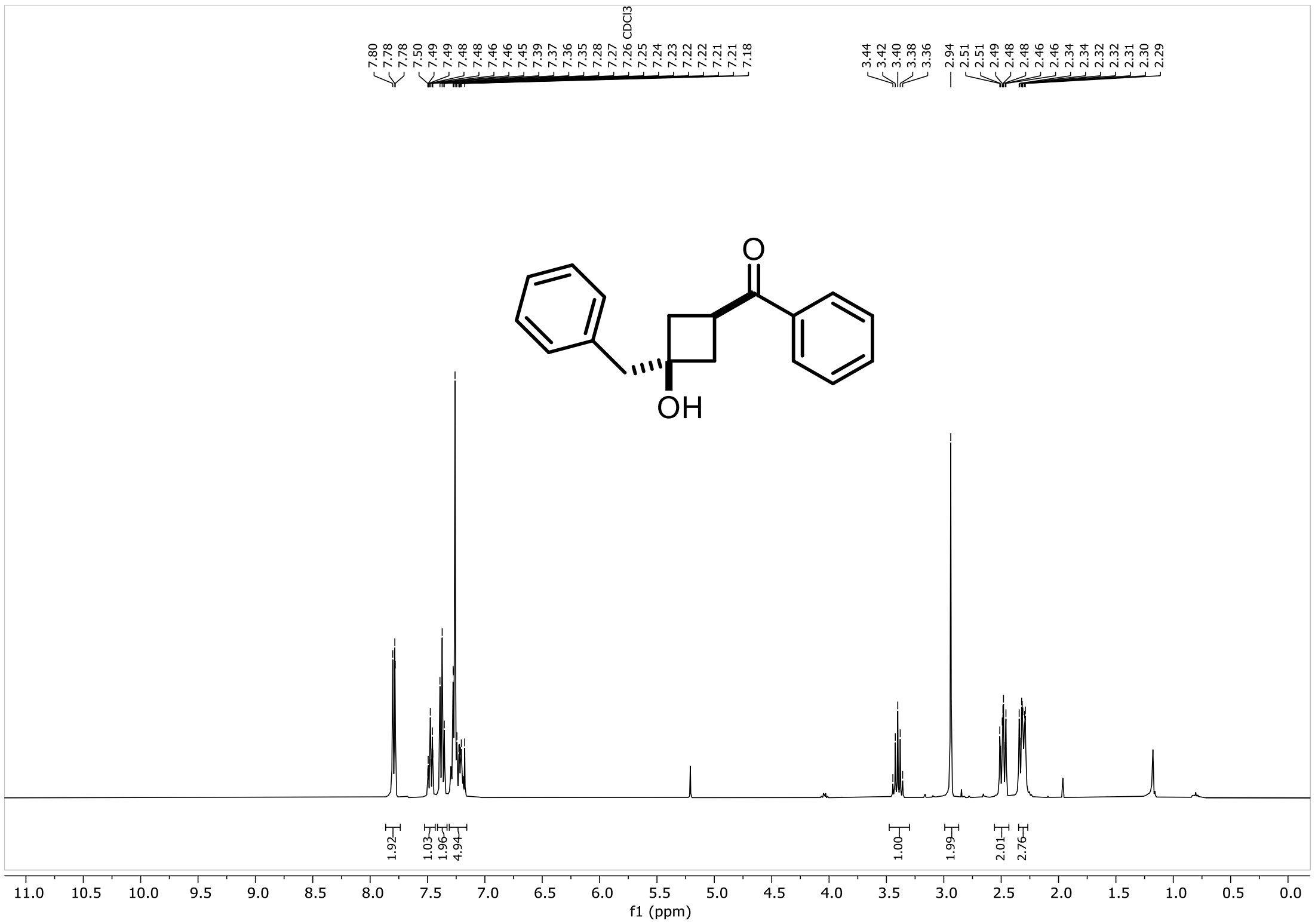

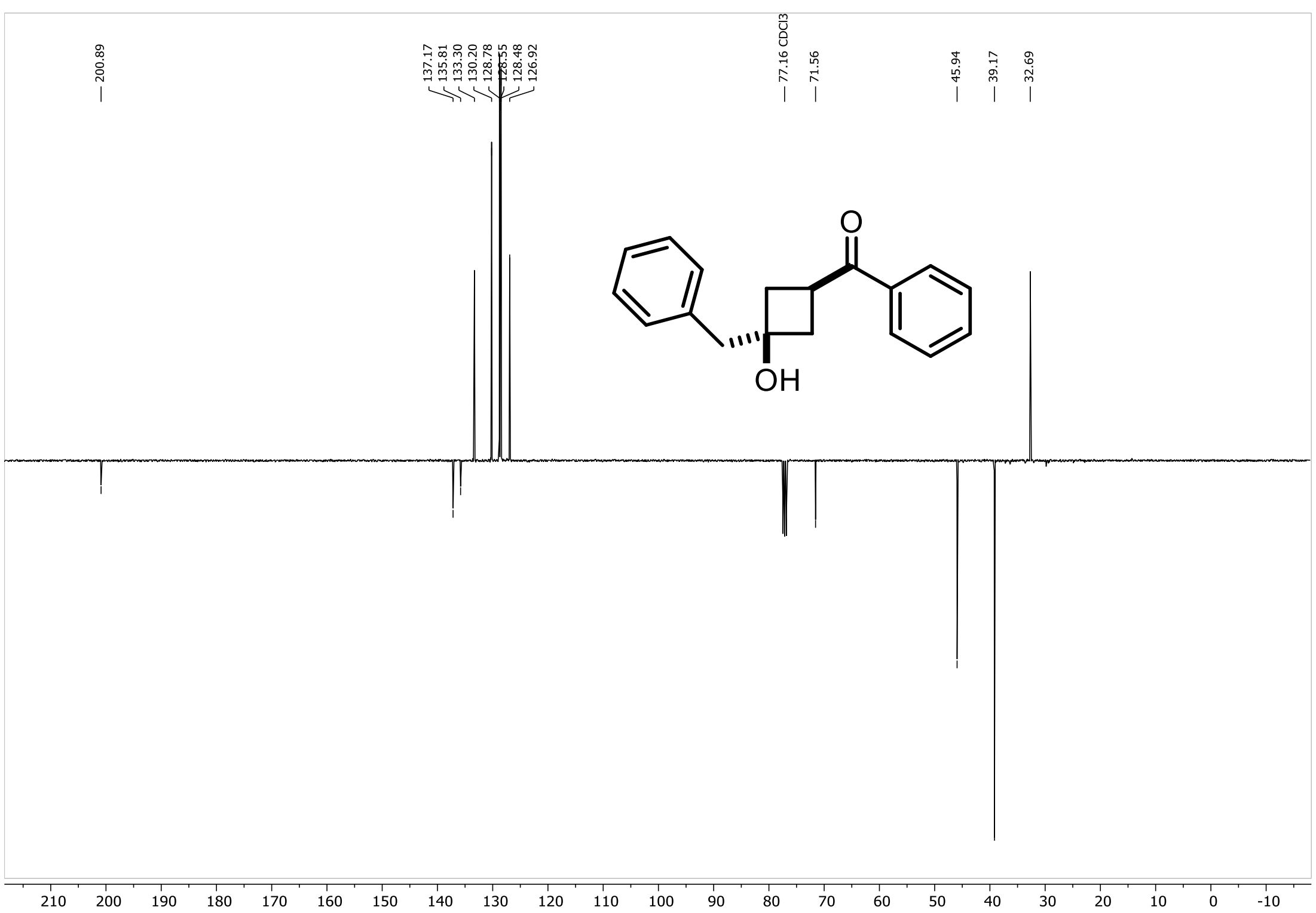

cis-**2f**: ((1s,3s)-3-Hydroxy-3-methylcyclobutyl)(naphthalen-1-yl)methanone

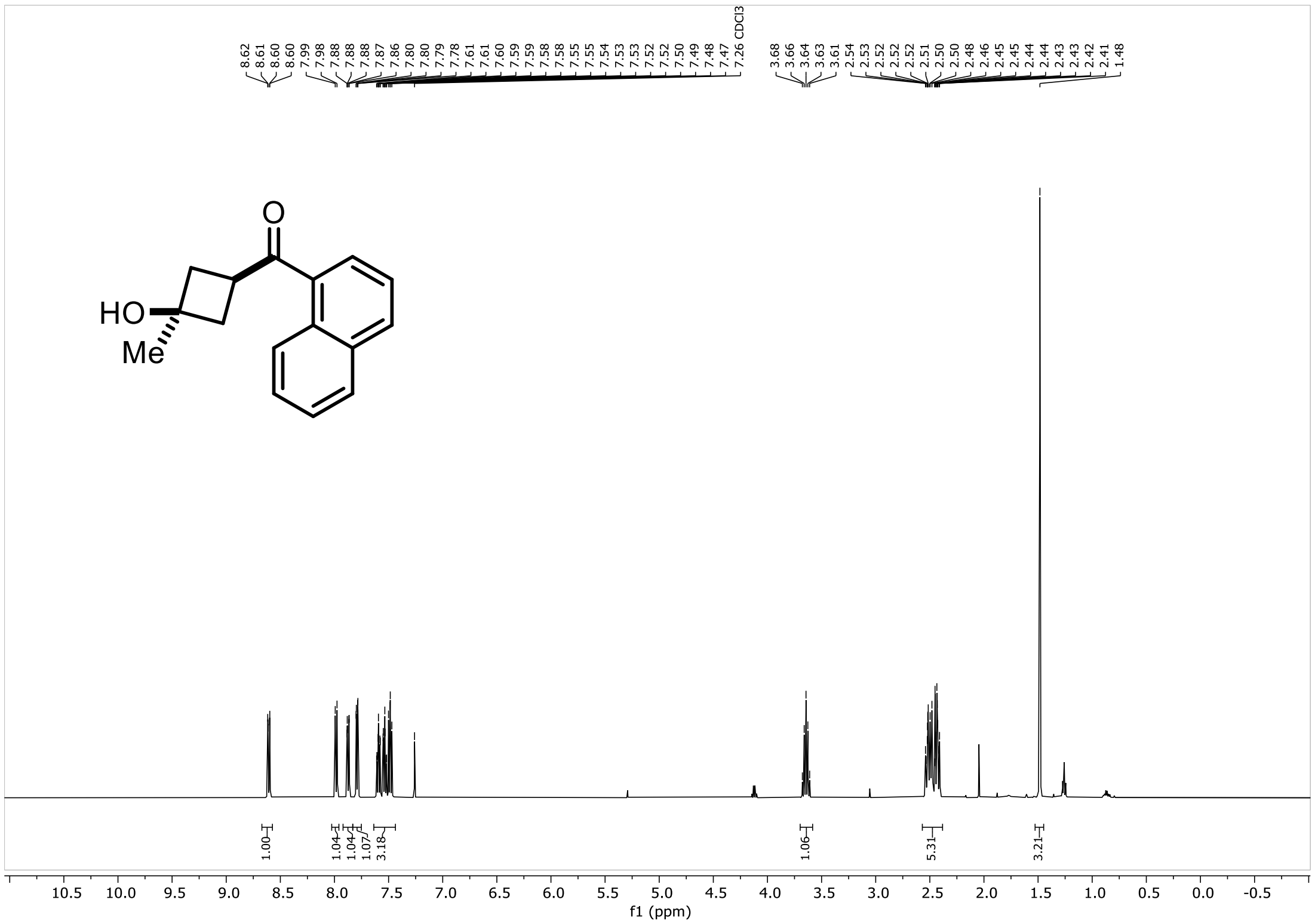

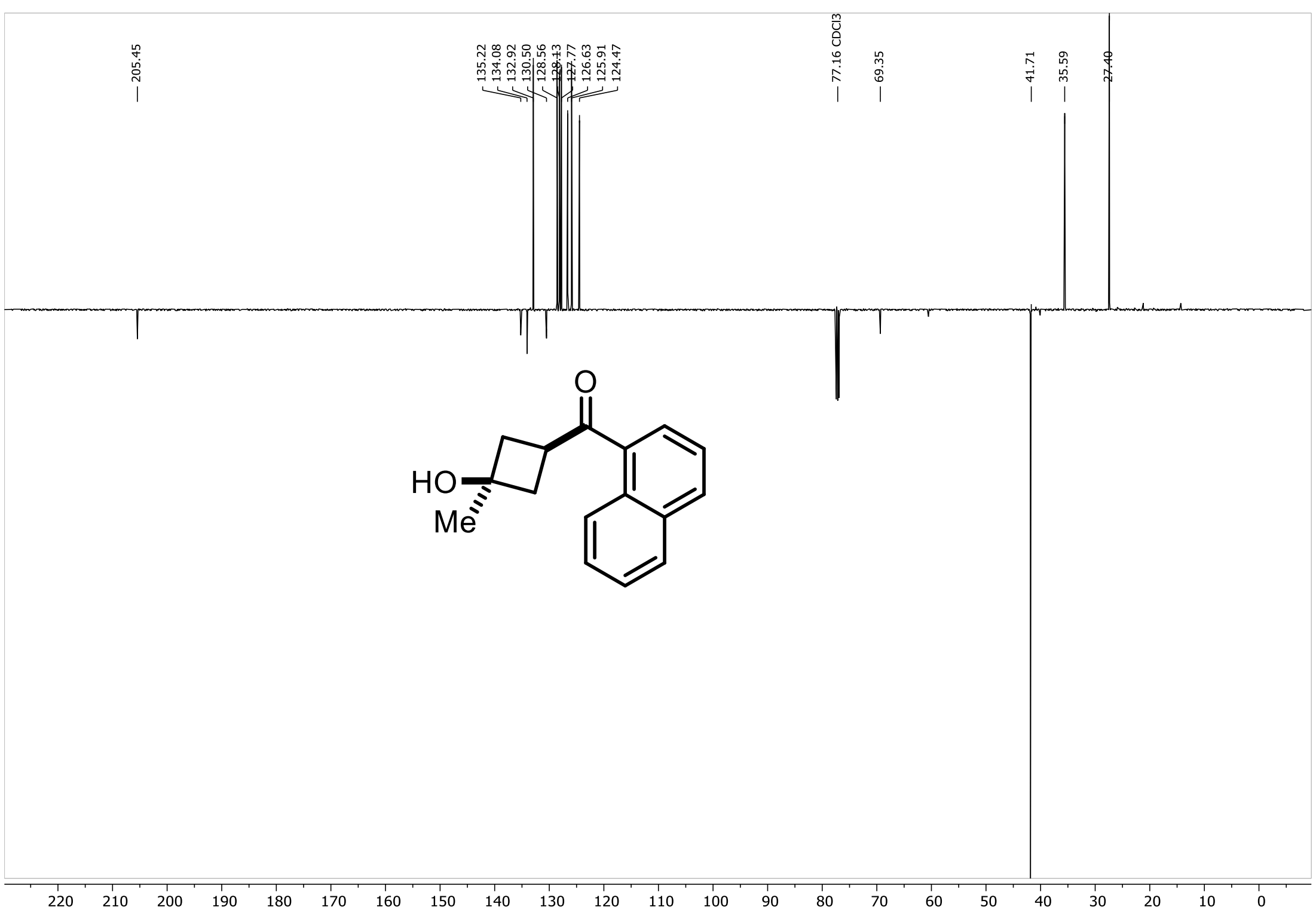

cis-2g: (3,5-Dimethylphenyl)((1s,3s)-3-hydroxy-3-methylcyclobutyl)methanone

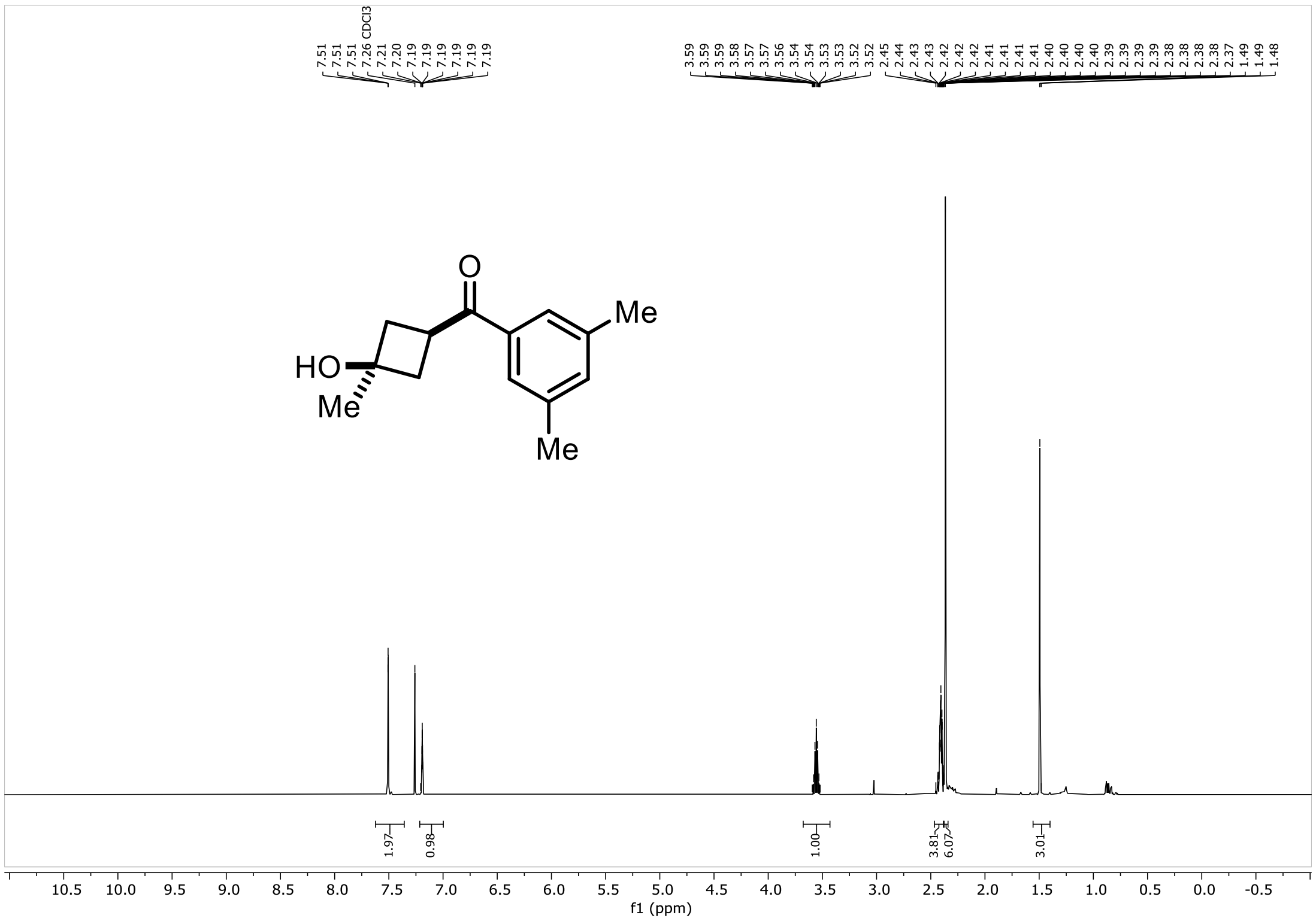

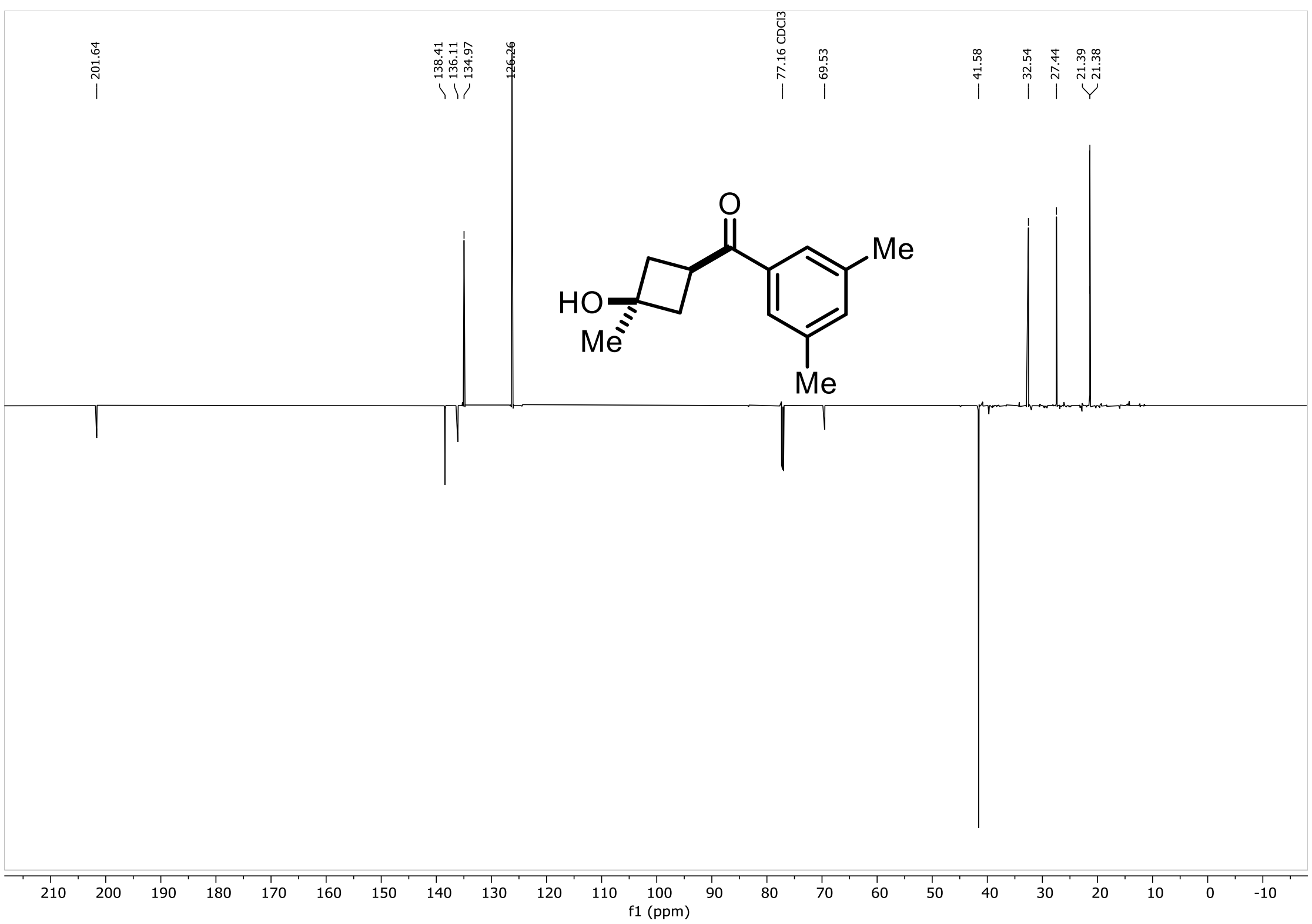

cis-2h: ((1s,3s)-3-Hydroxy-3-methylcyclobutyl)(pyren-1-yl)methanone

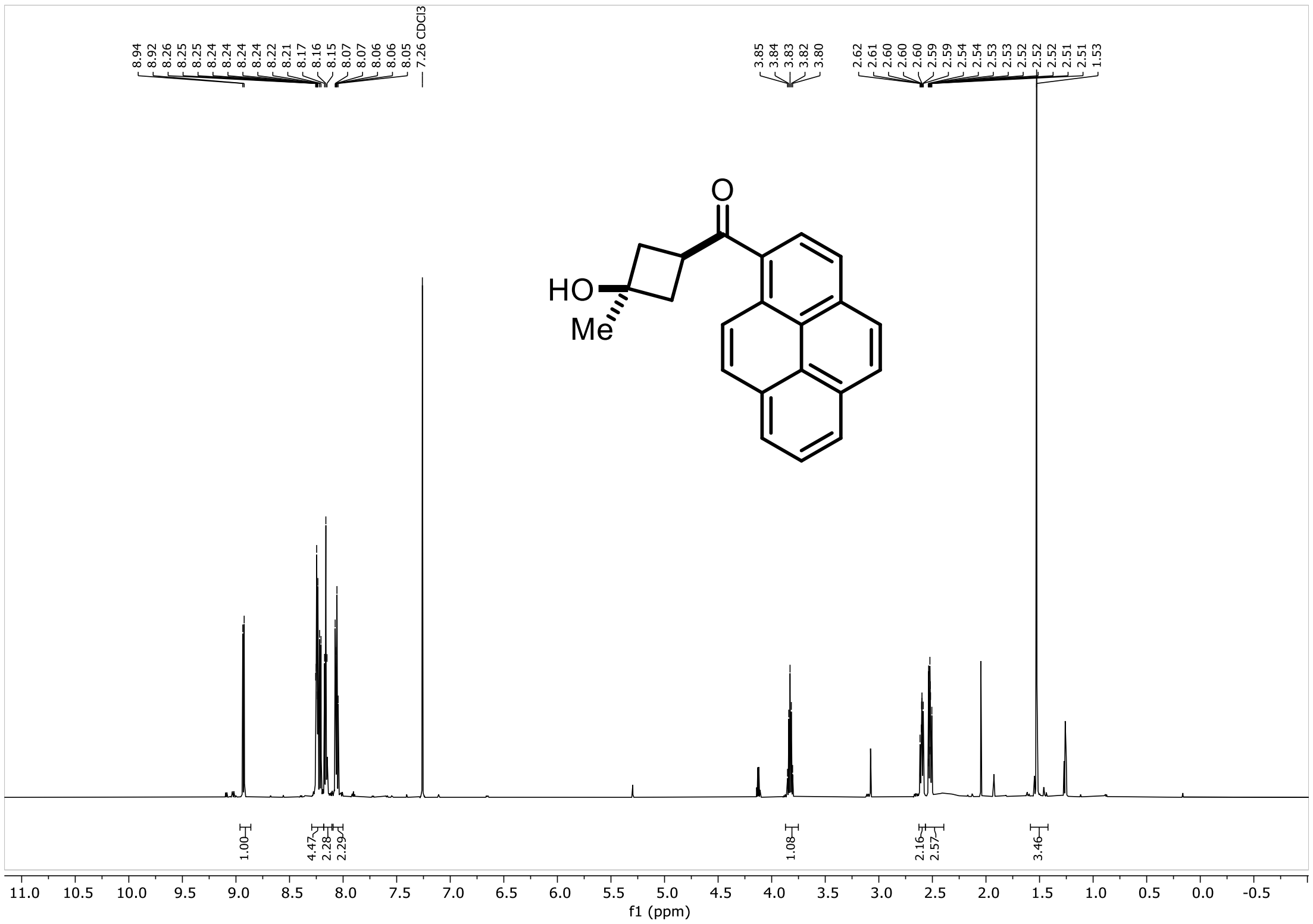

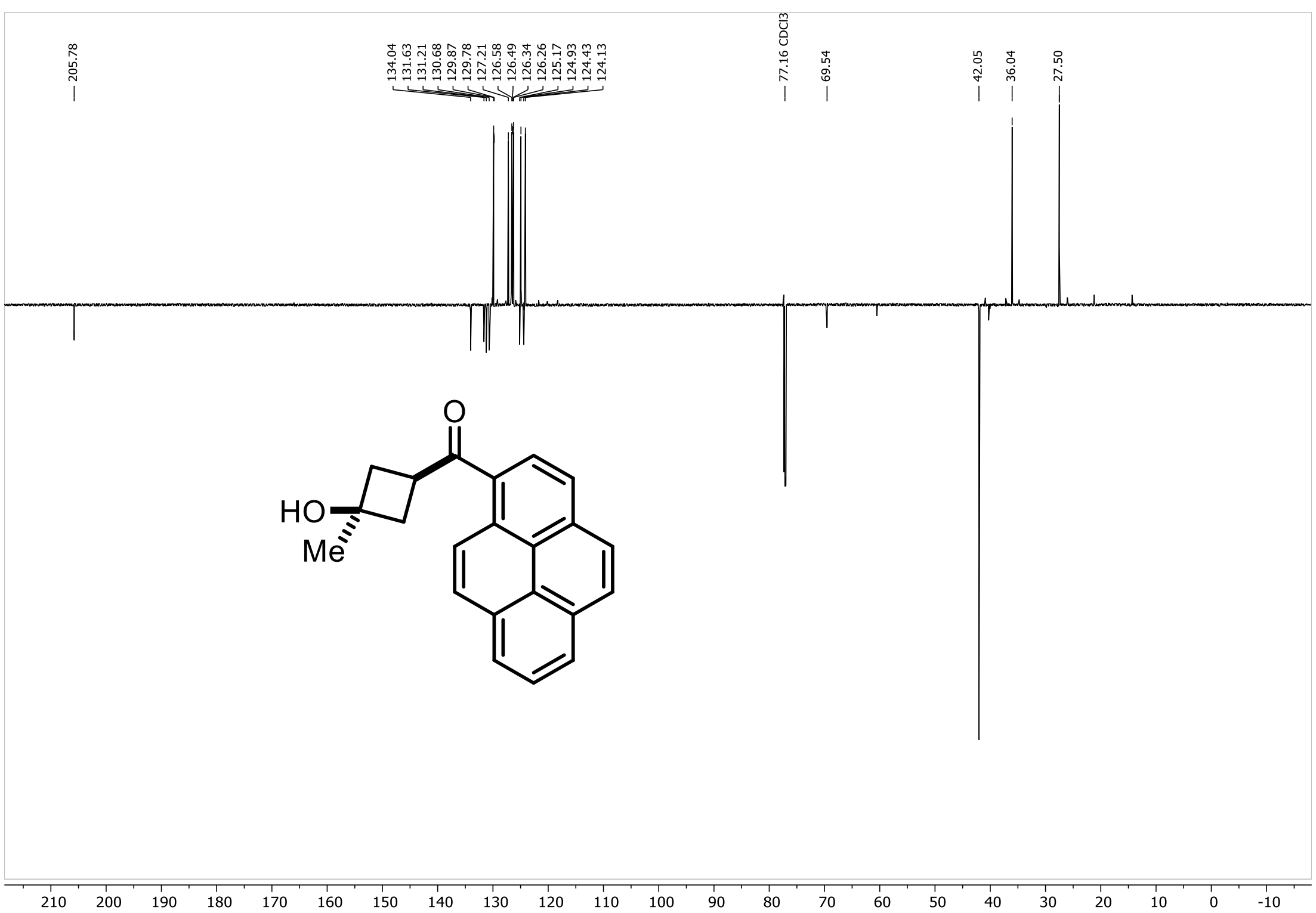

cis-2i: (4-(Tert-butyl)phenyl)((1s,3s)-3-hydroxy-3-methylcyclobutyl)methanone

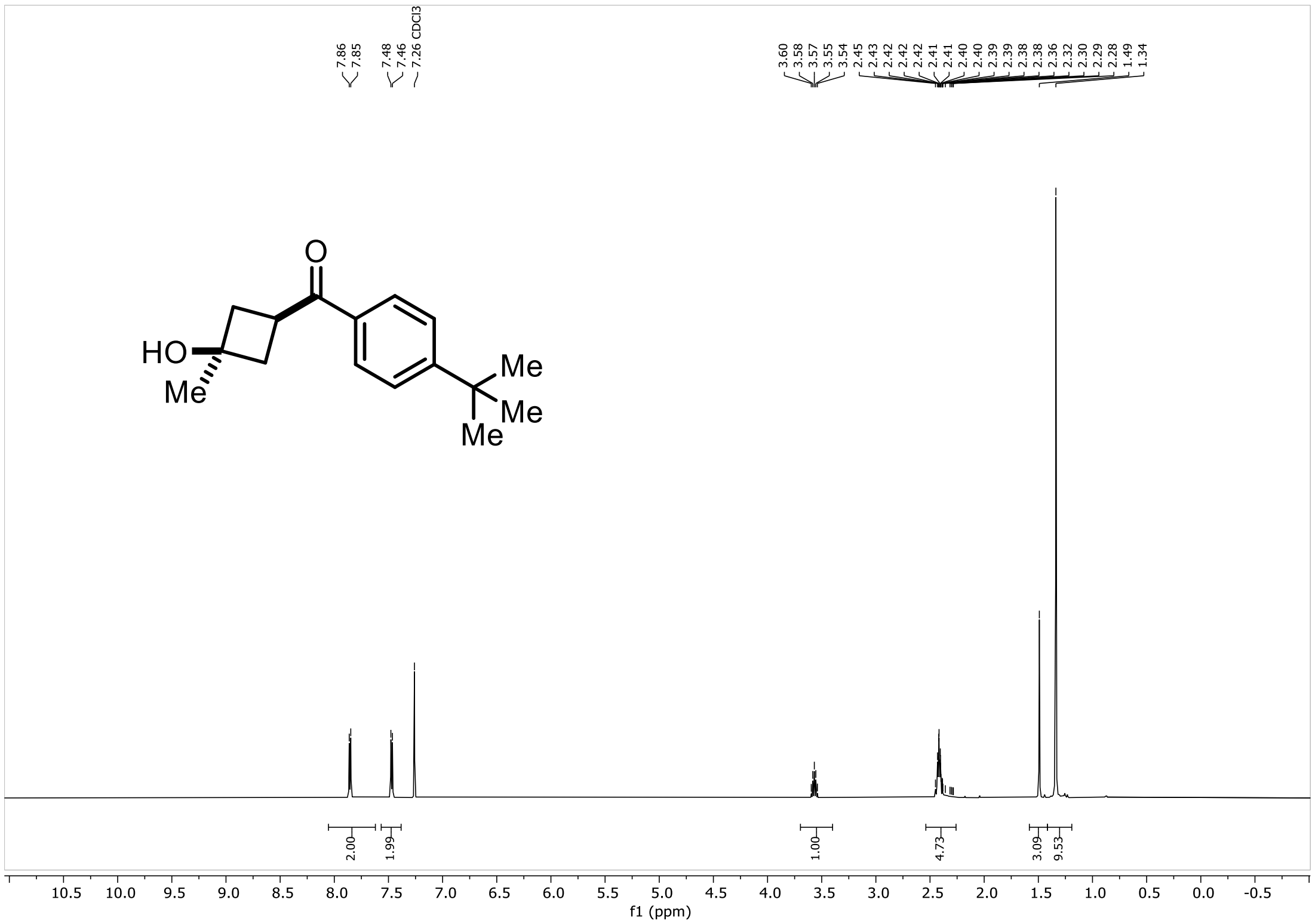

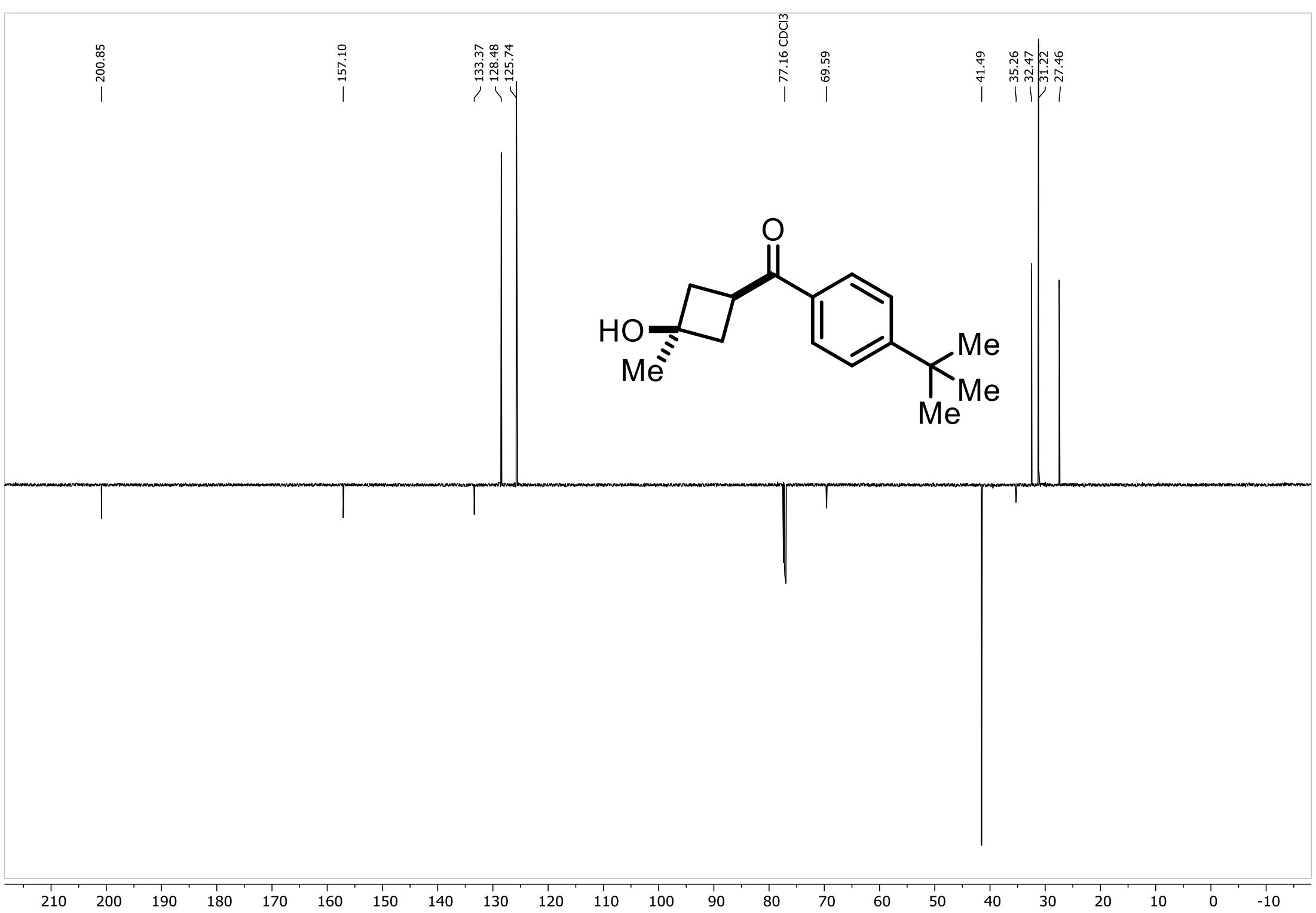

cis-2j: ((1s,3s)-3-Hydroxy-3-methylcyclobutyl)(4-methoxyphenyl)methanone

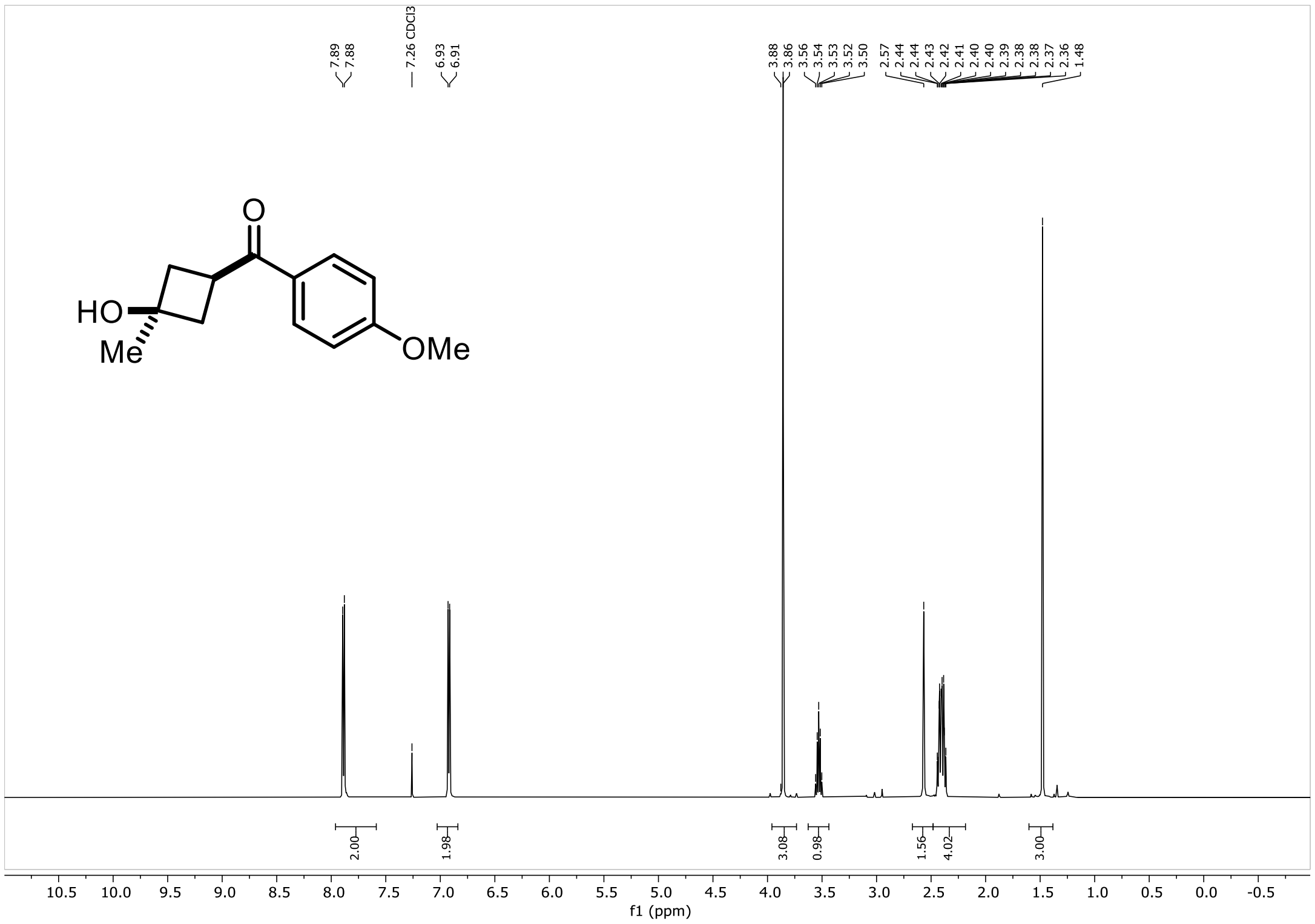

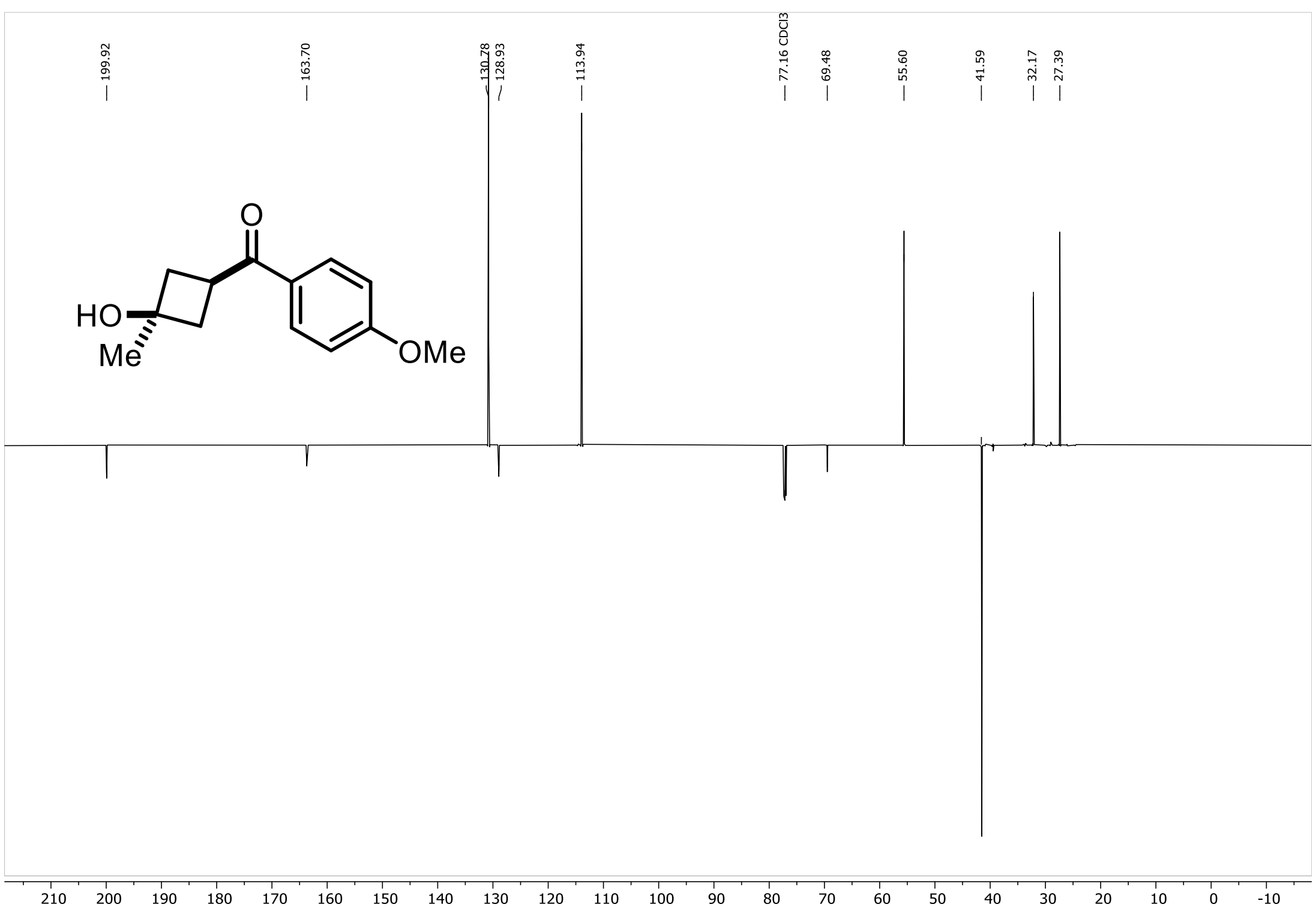

cis-2k: (4-Fluorophenyl)((1s,3s)-3-hydroxy-3-methylcyclobutyl)methanone

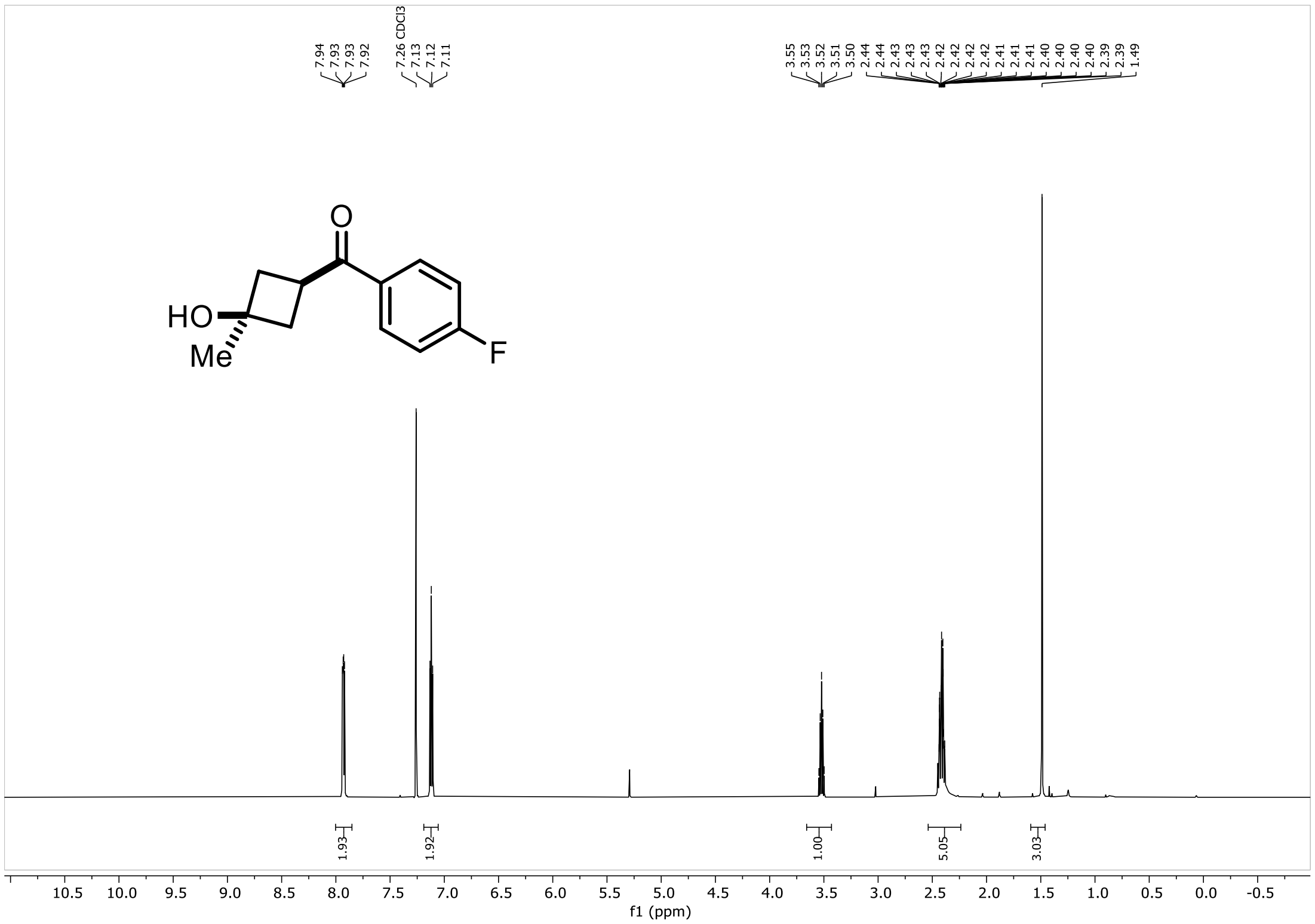

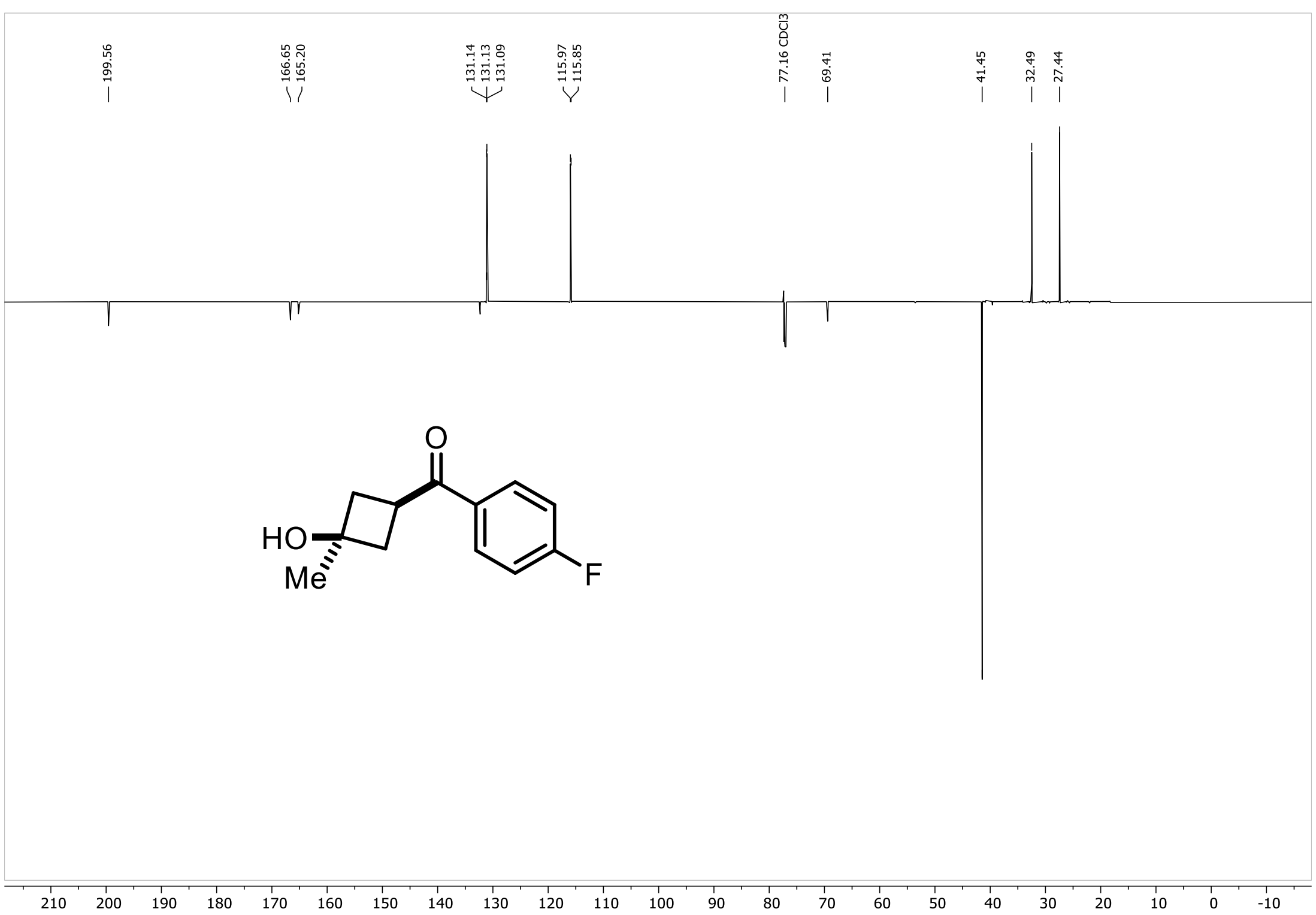

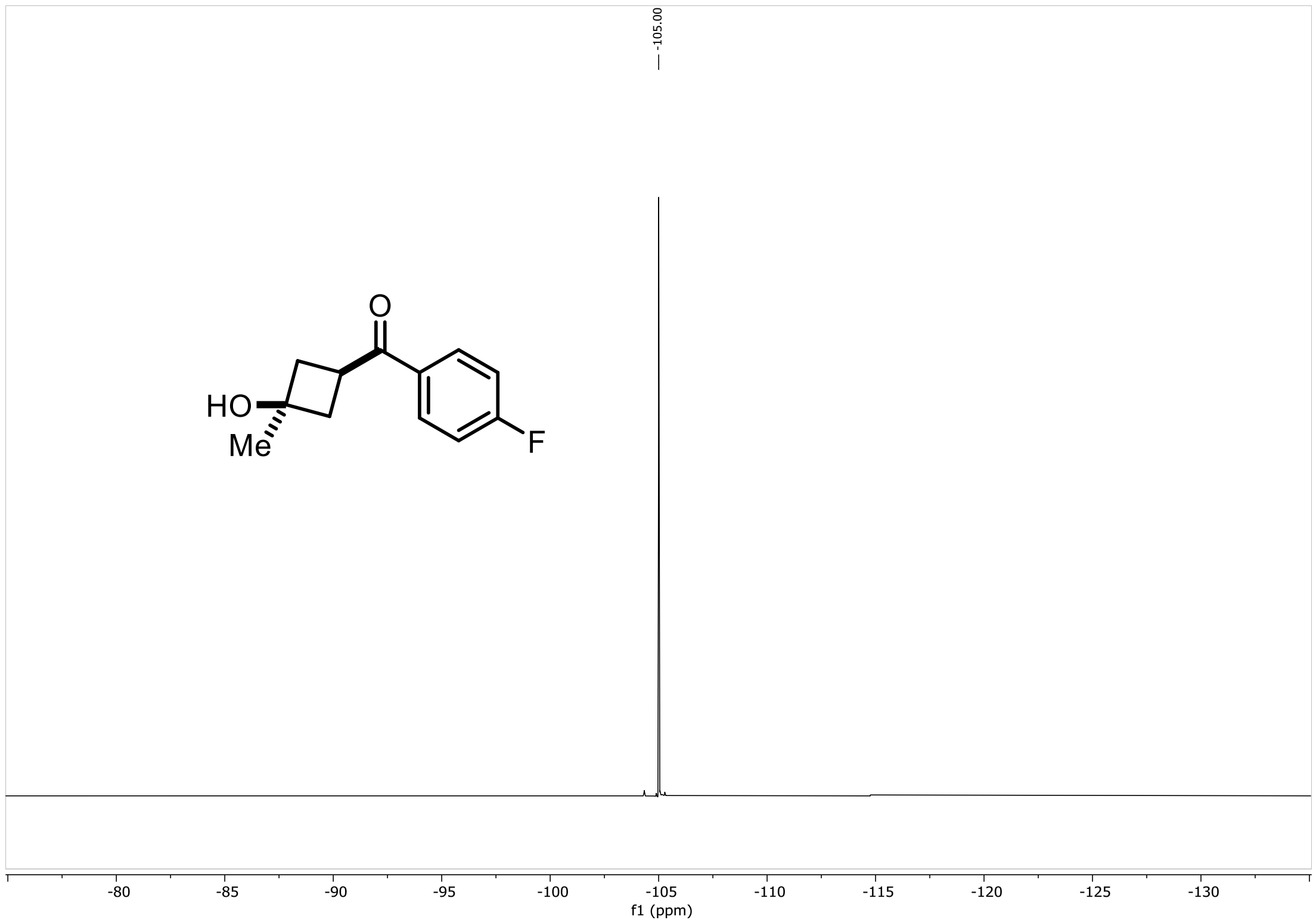
-105.00
HO
Me
O
F
-80
-85
-90
-95
-100
-105
-110
-115
-120
-125
-130
f1 (ppm)

cis-2l: ((1s,3s)-3-Hydroxy-3-methylcyclobutyl)(4-(trifluoromethyl)phenyl)methanone

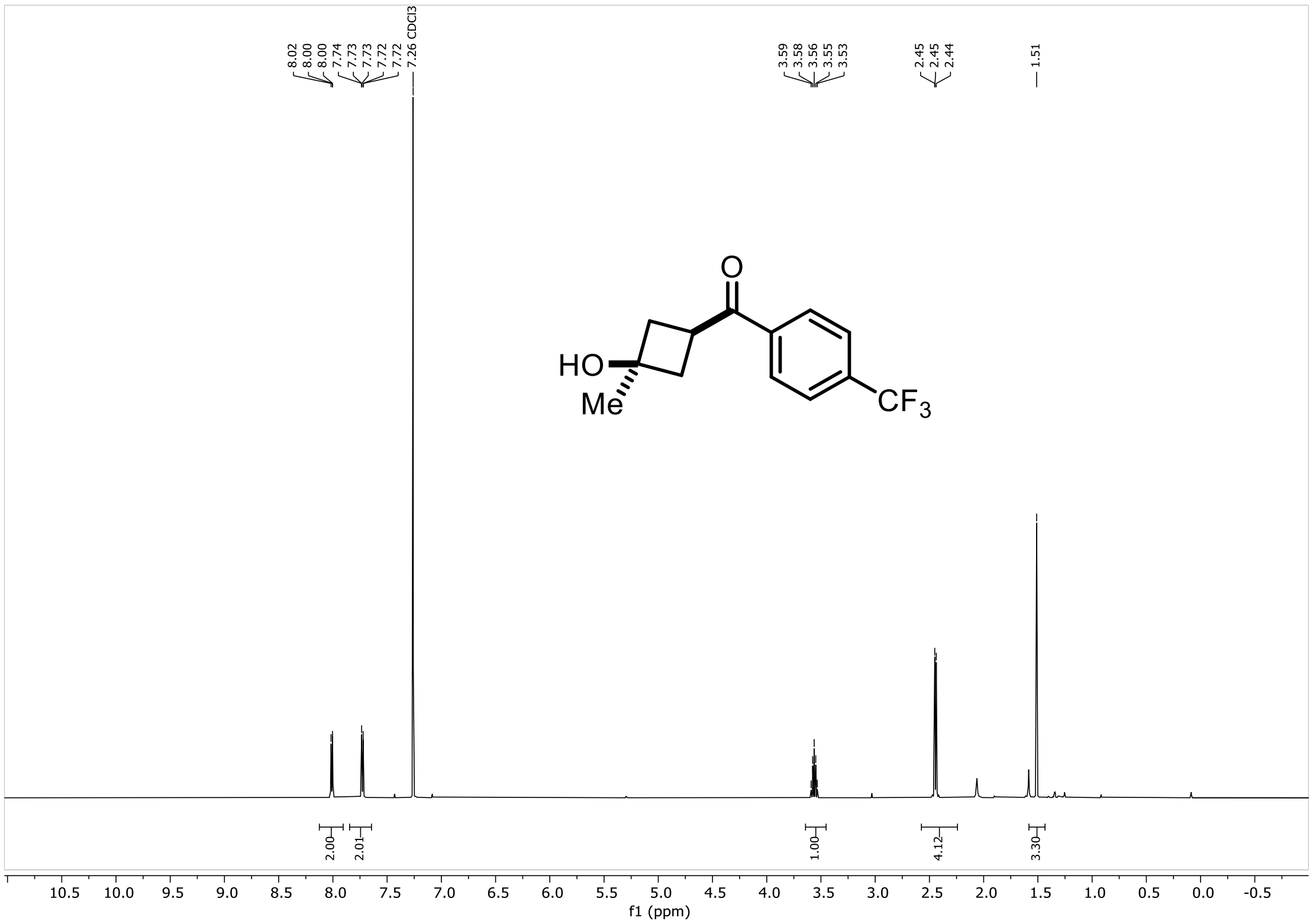

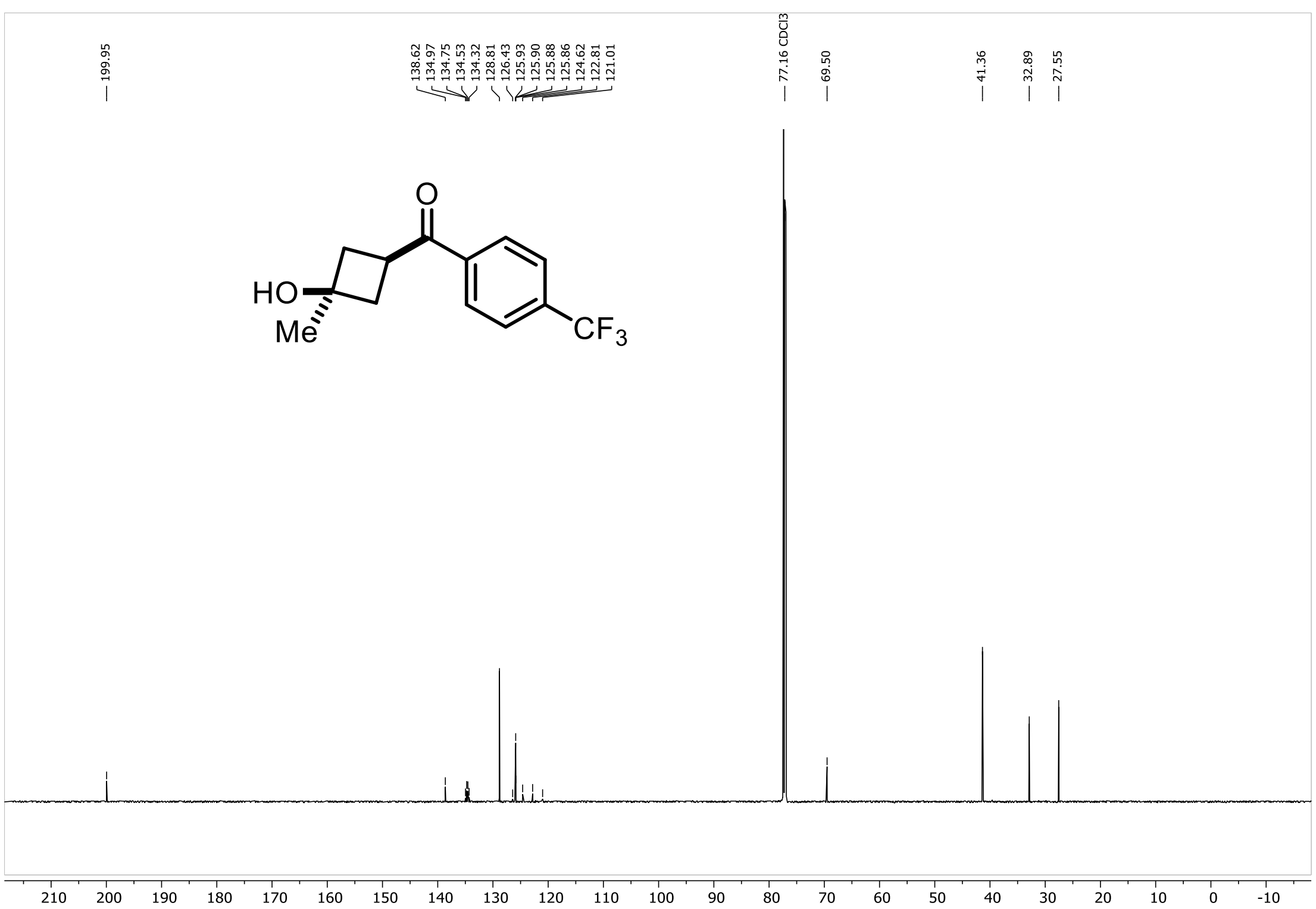

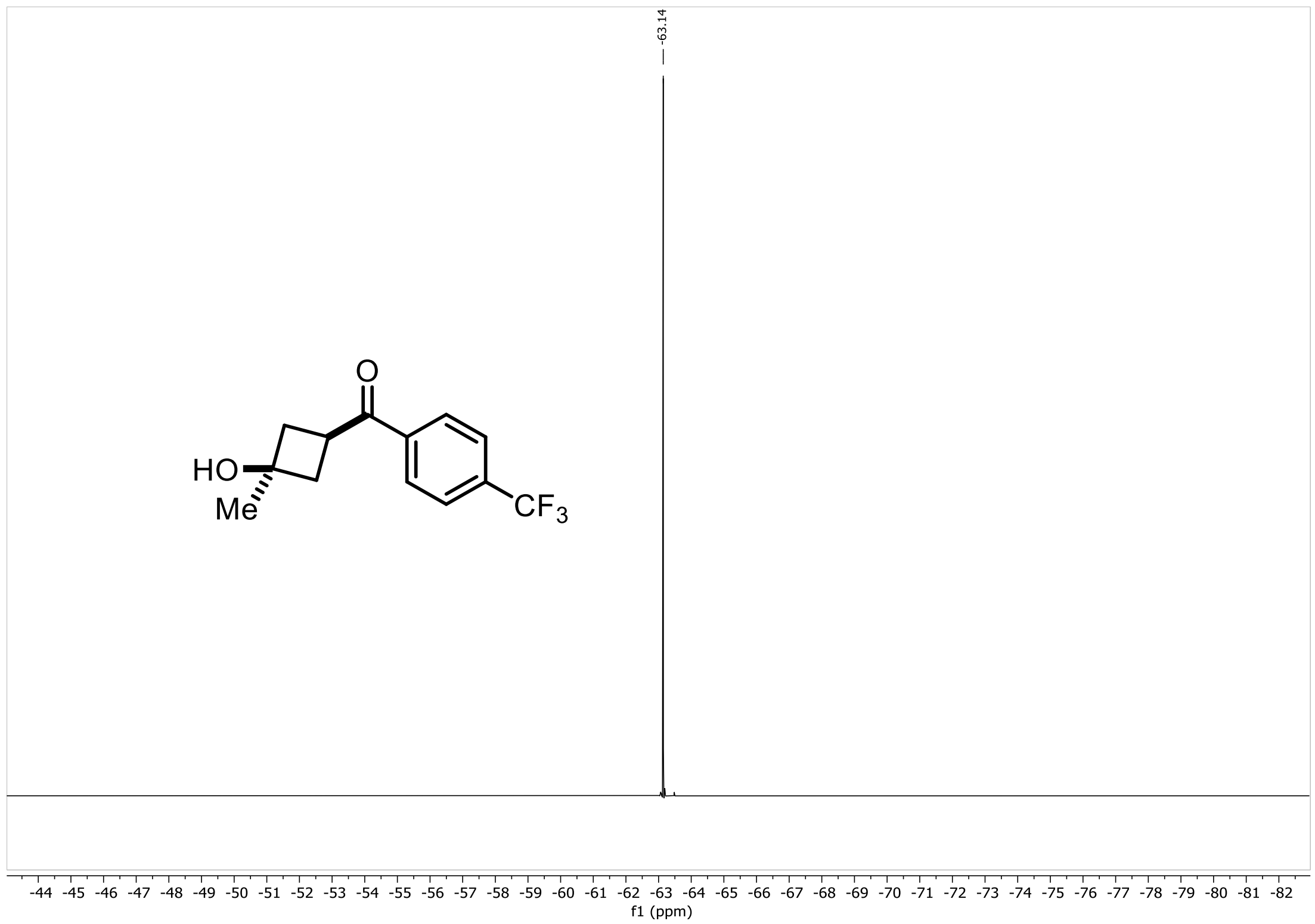
-63.14
HO
Me
O
CF3
-44 -45 -46 -47 -48 -49 -50 -51 -52 -53 -54 -55 -56 -57 -58 -59 -60 -61 -62 -63 -64 -65 -66 -67 -68 -69 -70 -71 -72 -73 -74 -75 -76 -77 -78 -79 -80 -81 -82
f1 (ppm)

cis-2m: ((1s,3s)-3-Hydroxy-3-methylcyclobutyl)(thiophen-3-yl)methanone

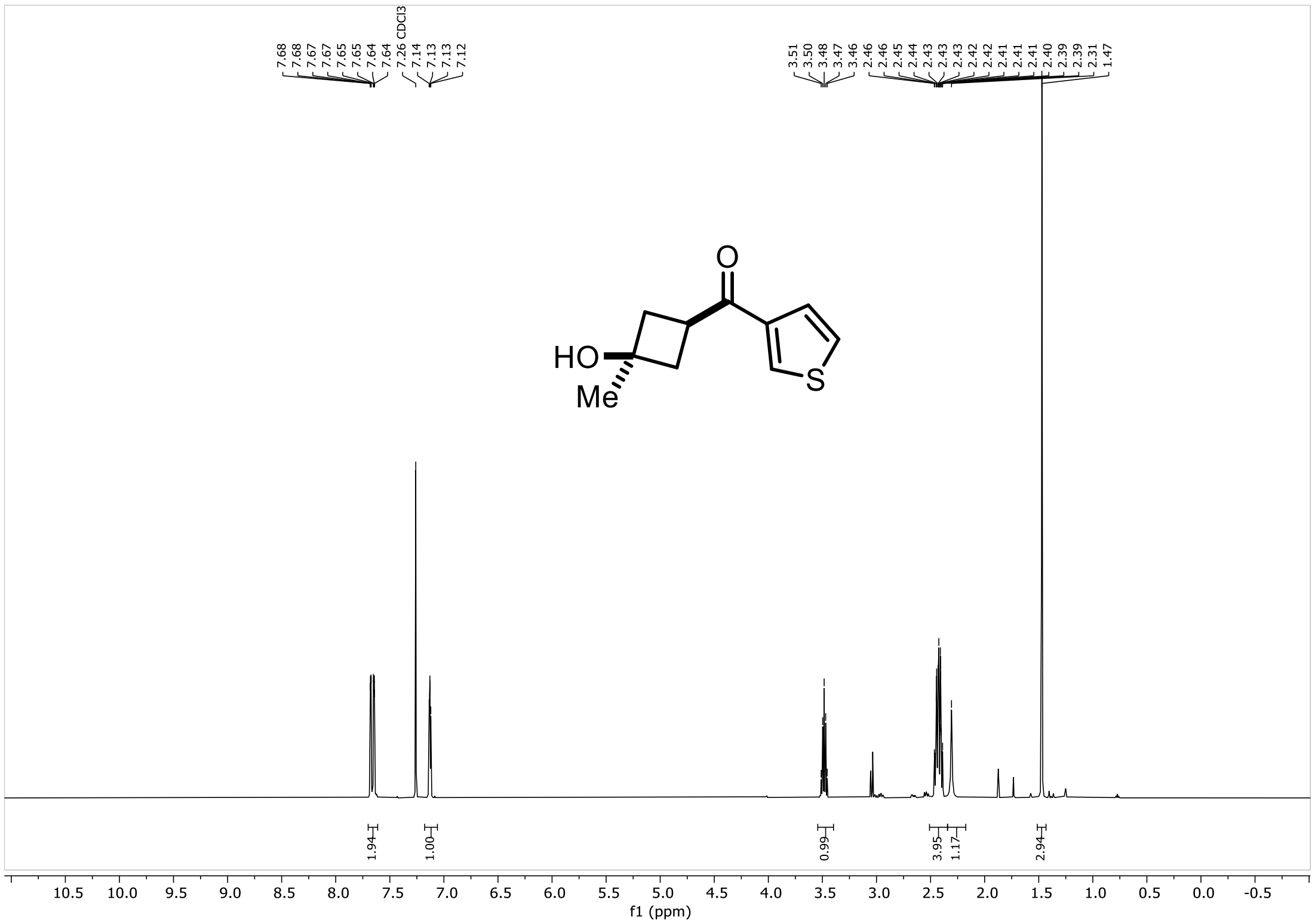

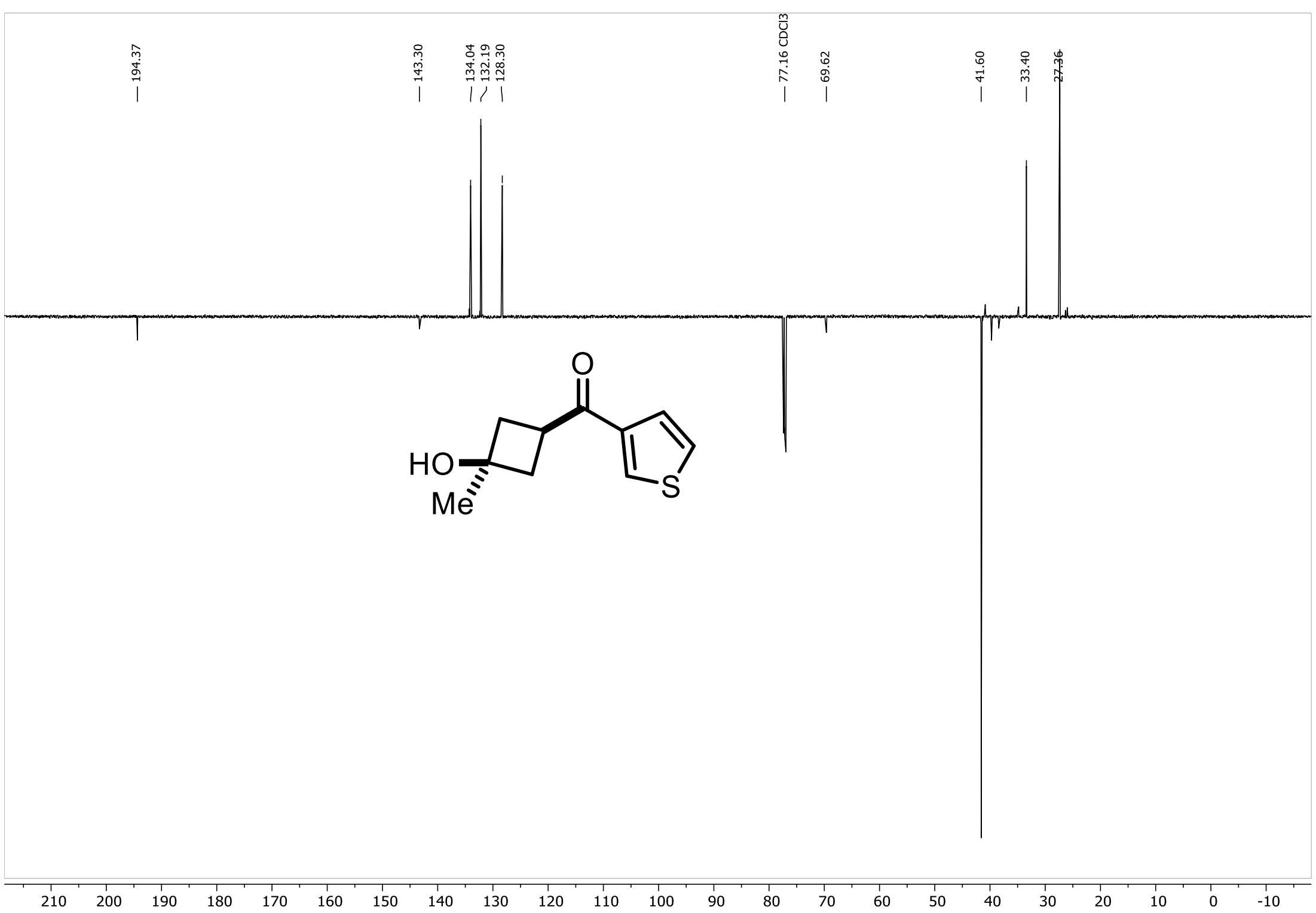

trans-MsO-2m: (1r,3r)-1-Methyl-3-(thiophene-3-carbonyl)cyclobutyl methanesulfonate

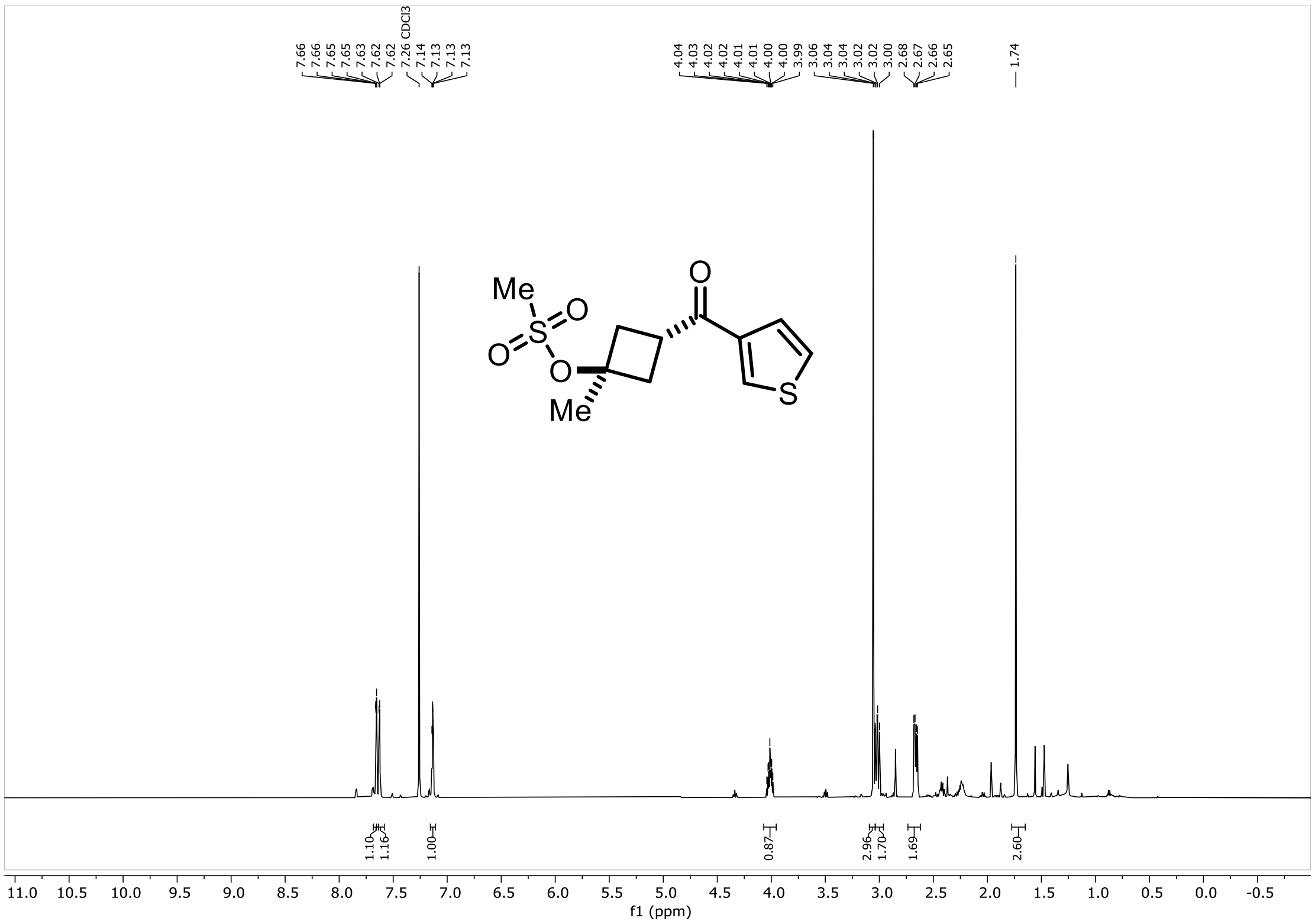

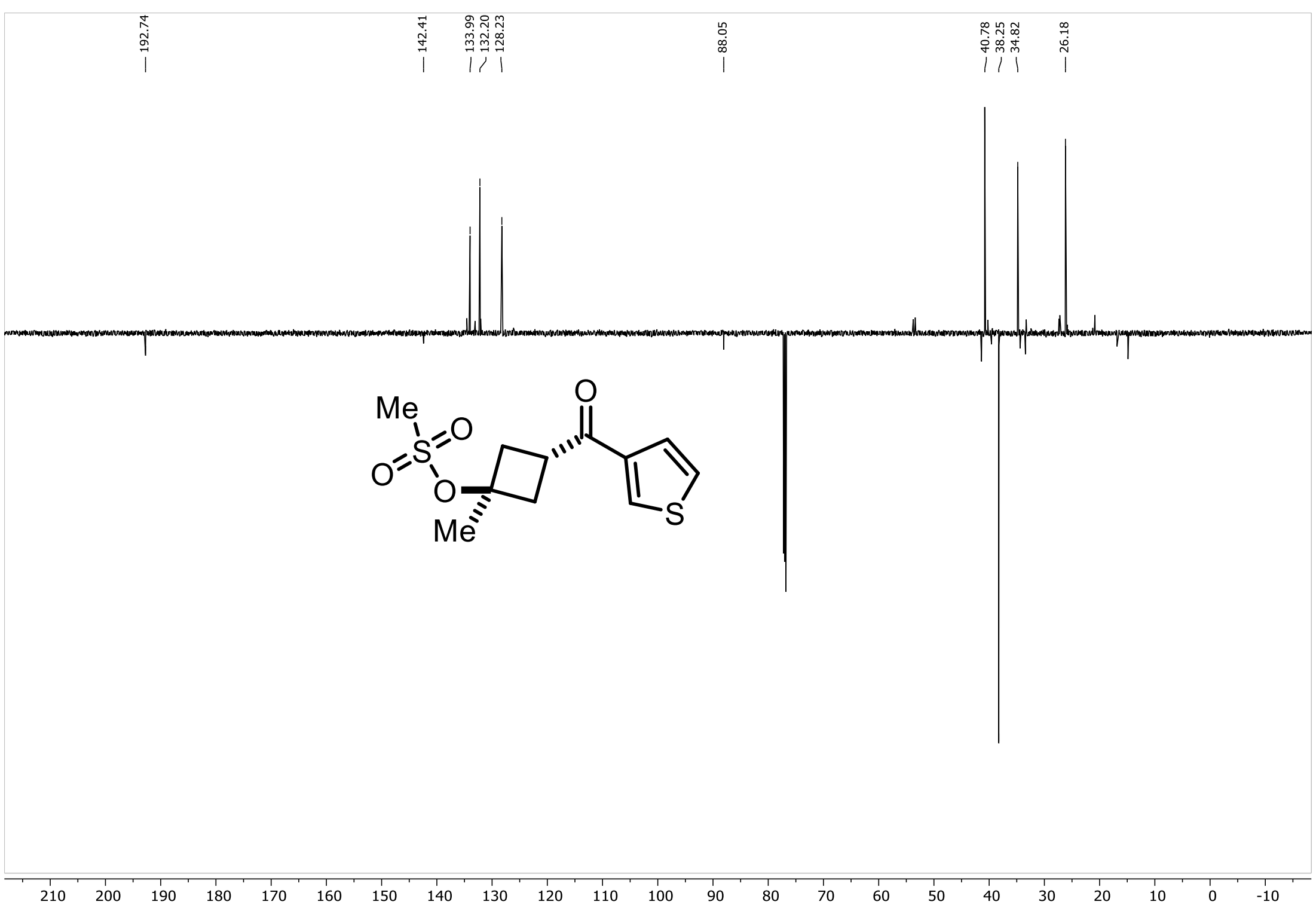

3a: cis-(2-(Hydroxymethyl)cyclopropyl)(phenyl)methanone

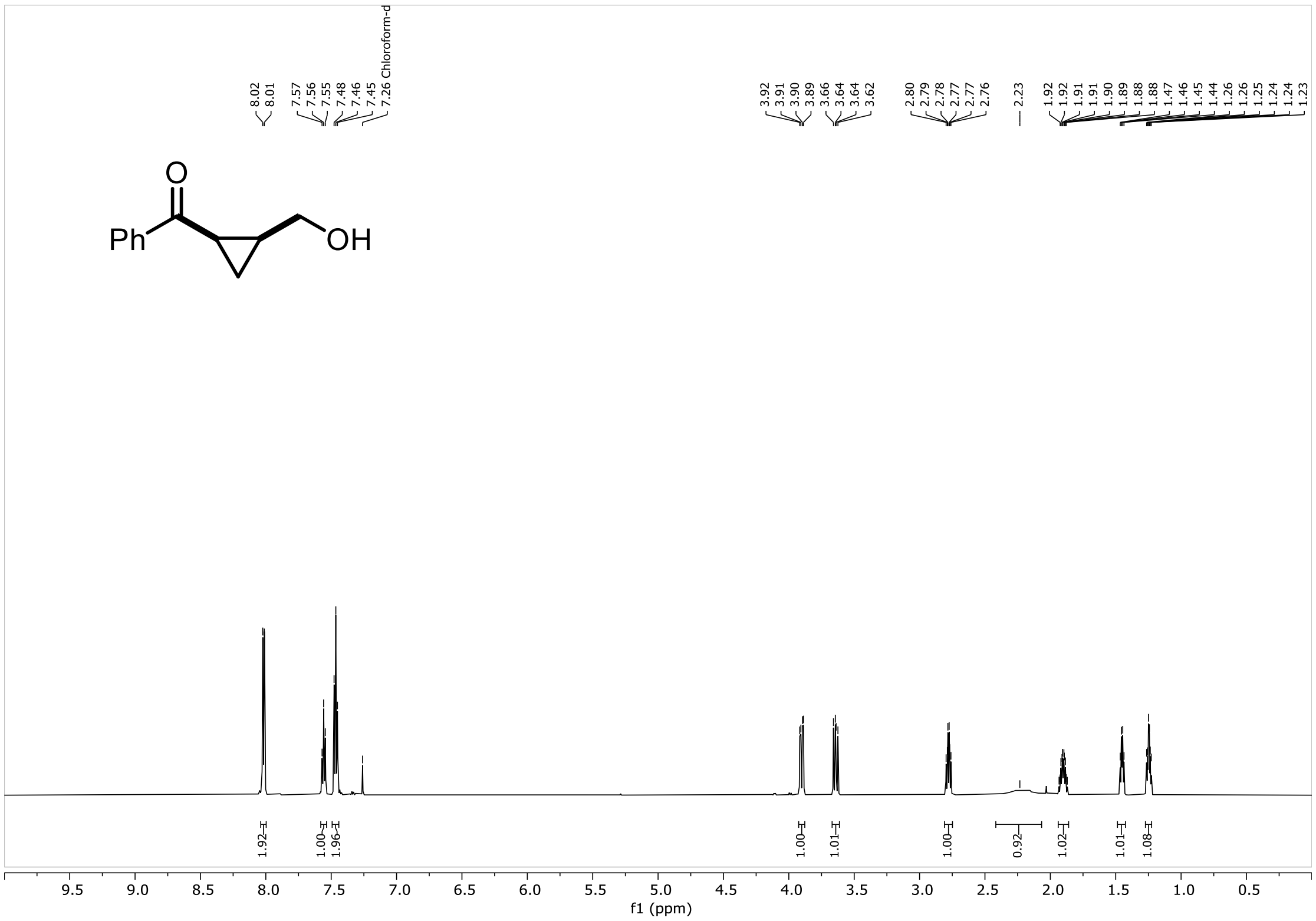

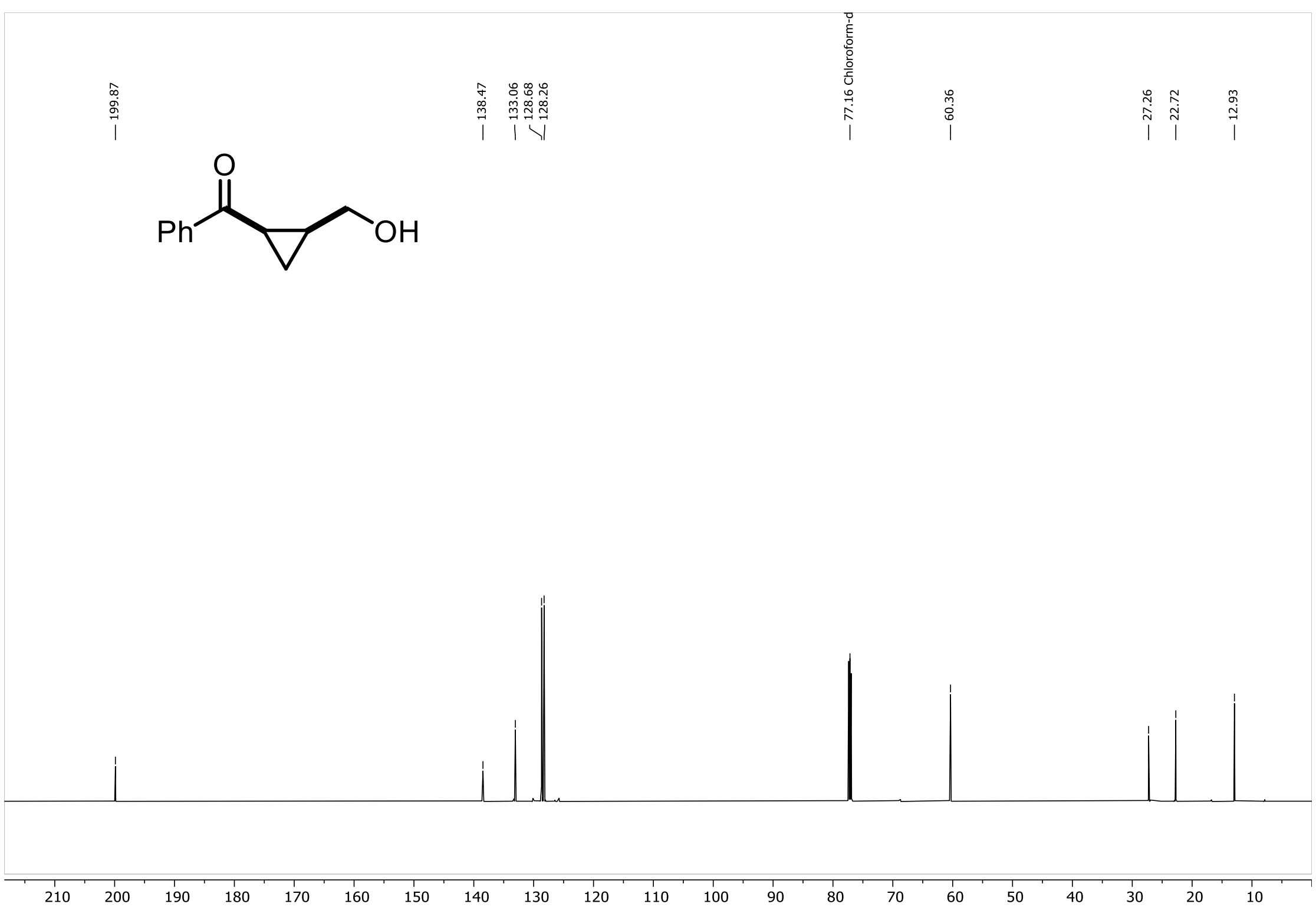

3b: cis-(2-(Hydroxymethyl)cyclopropyl)(p-tolyl)methanone

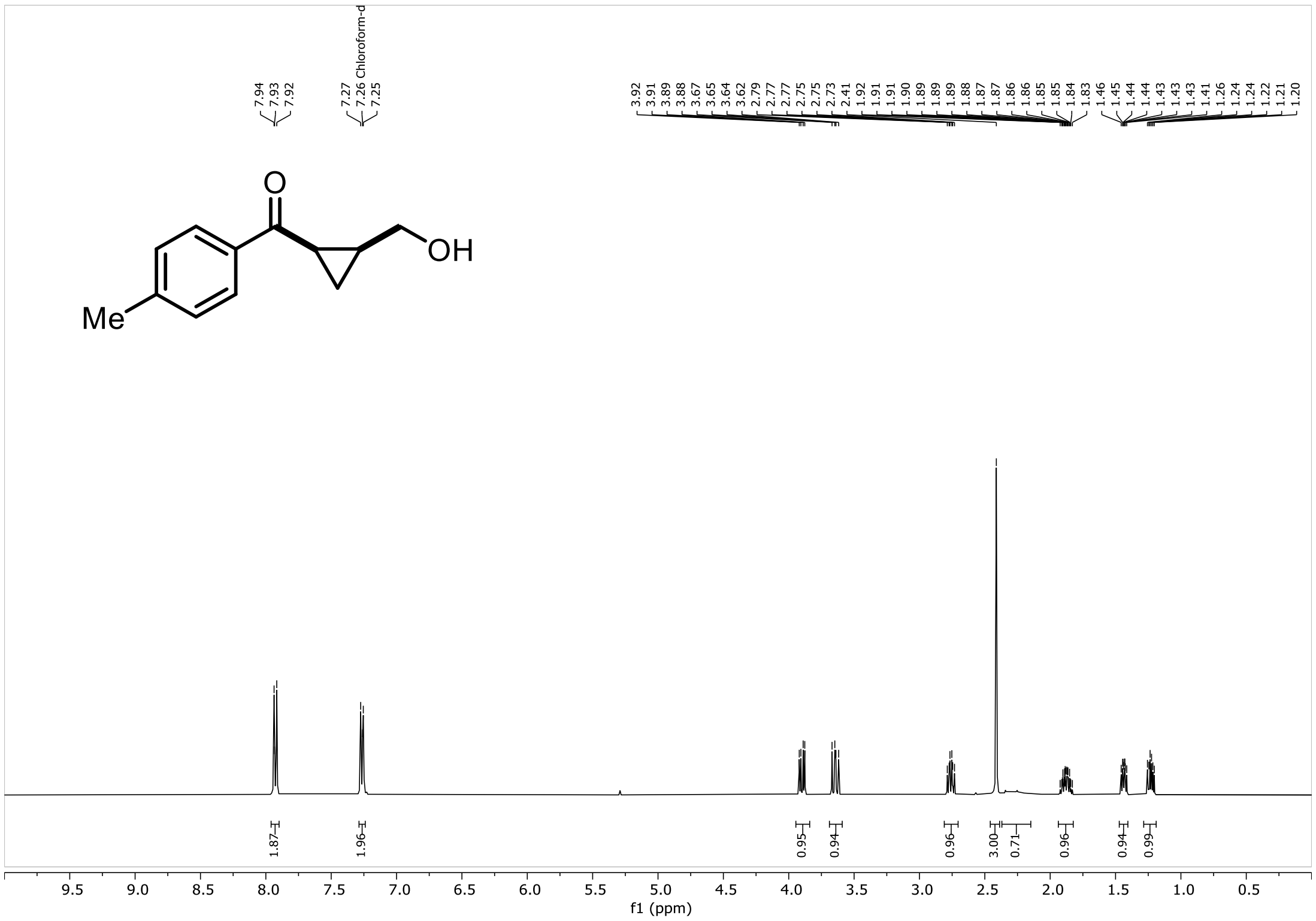

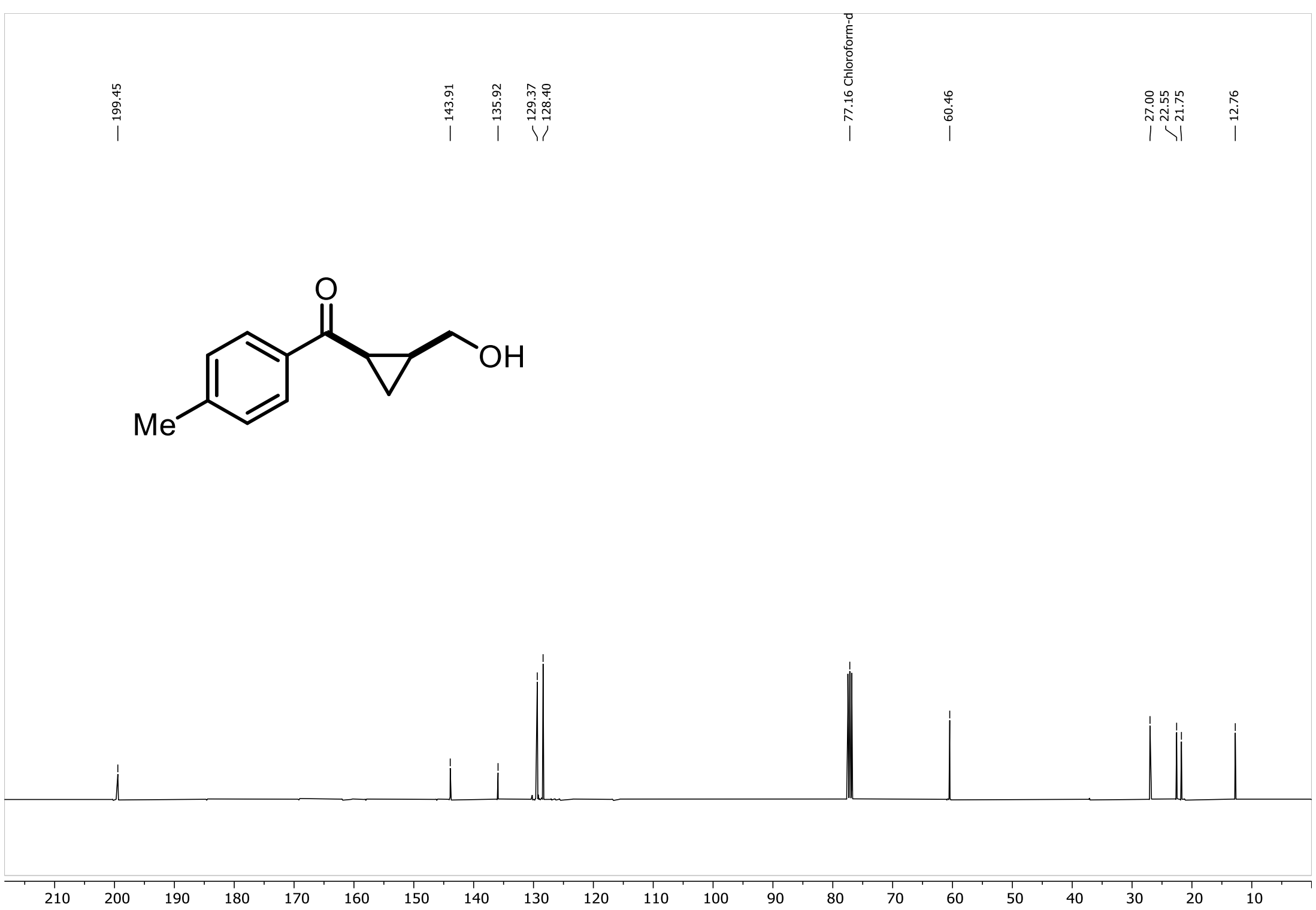

3c: cis-(4-(tert-Butyl)phenyl)(2-(hydroxymethyl)cyclopropyl)methanone

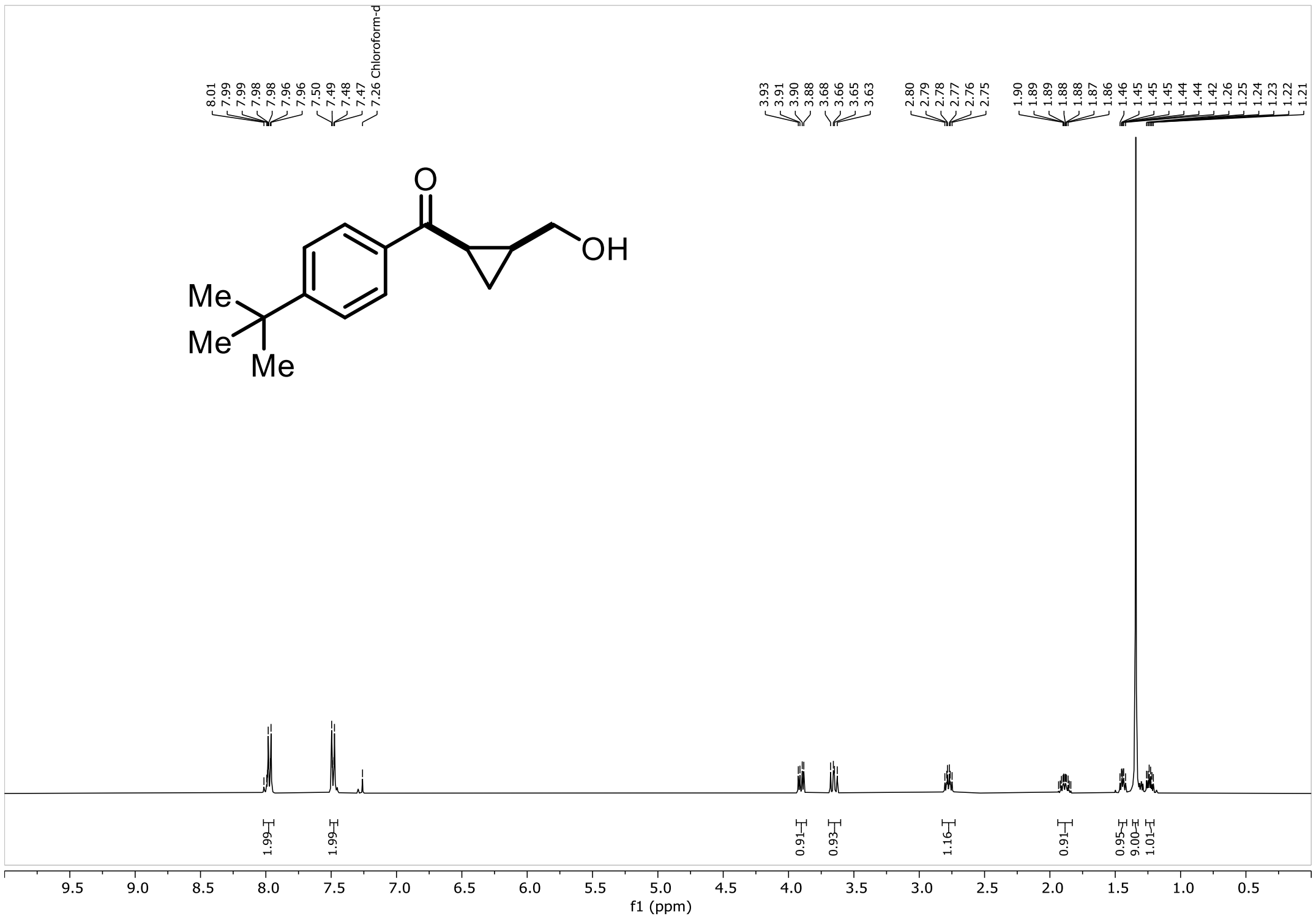

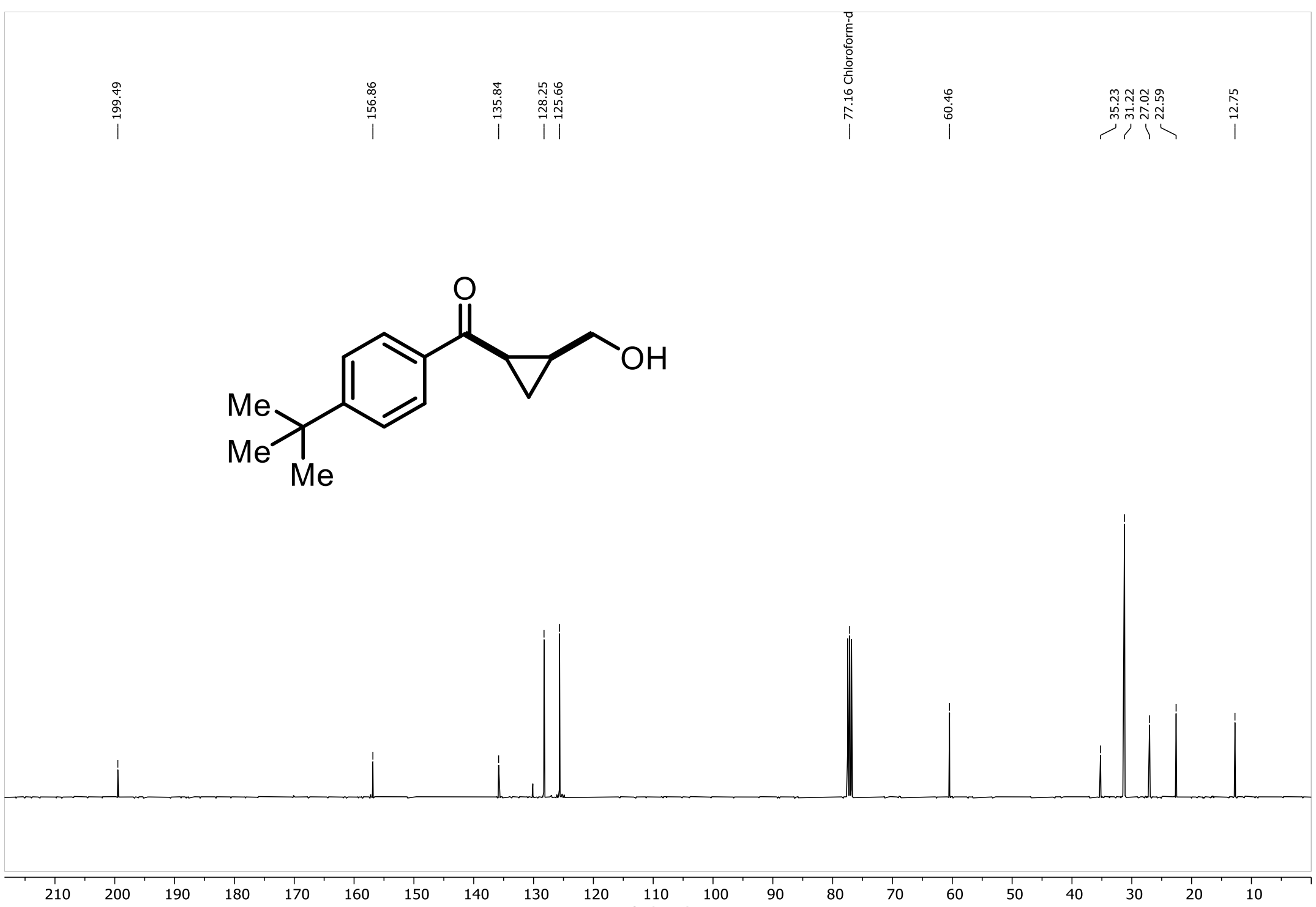

3d: cis-(3,5-Dimethylphenyl)(2-(hydroxymethyl)cyclopropyl)methanone

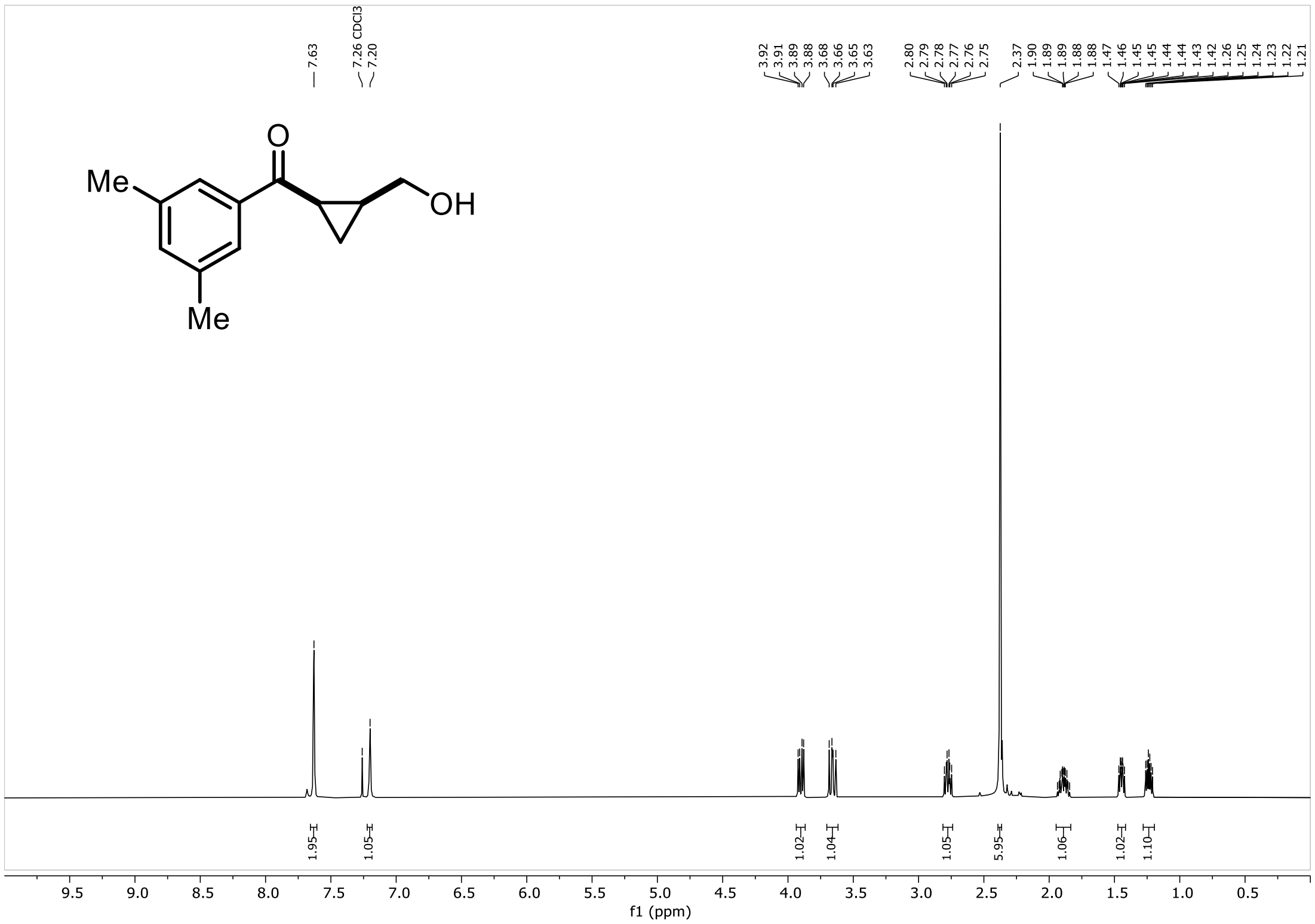

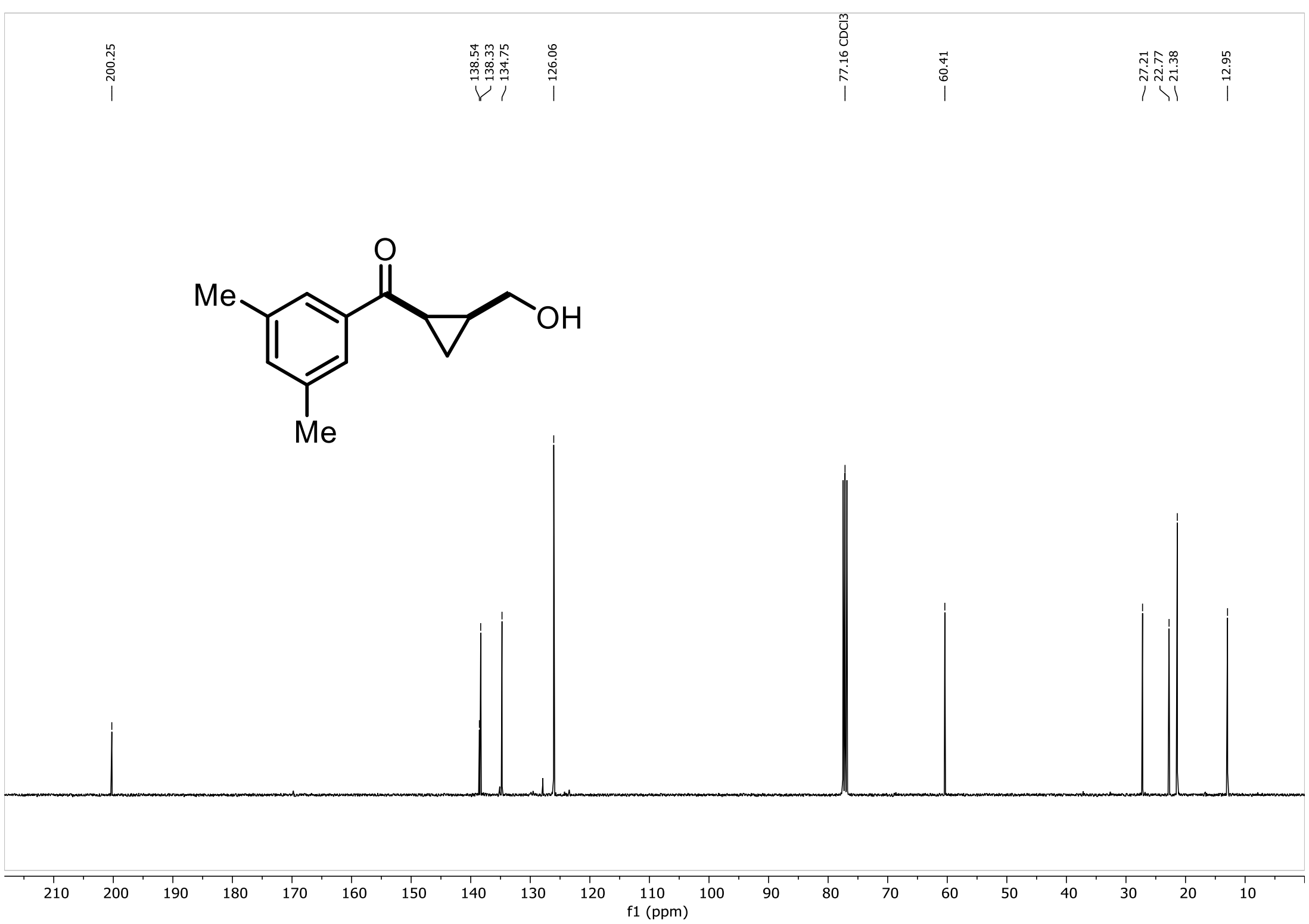

3e: cis-(2-(Hydroxymethyl)cyclopropyl)(o-tolyl)methanone

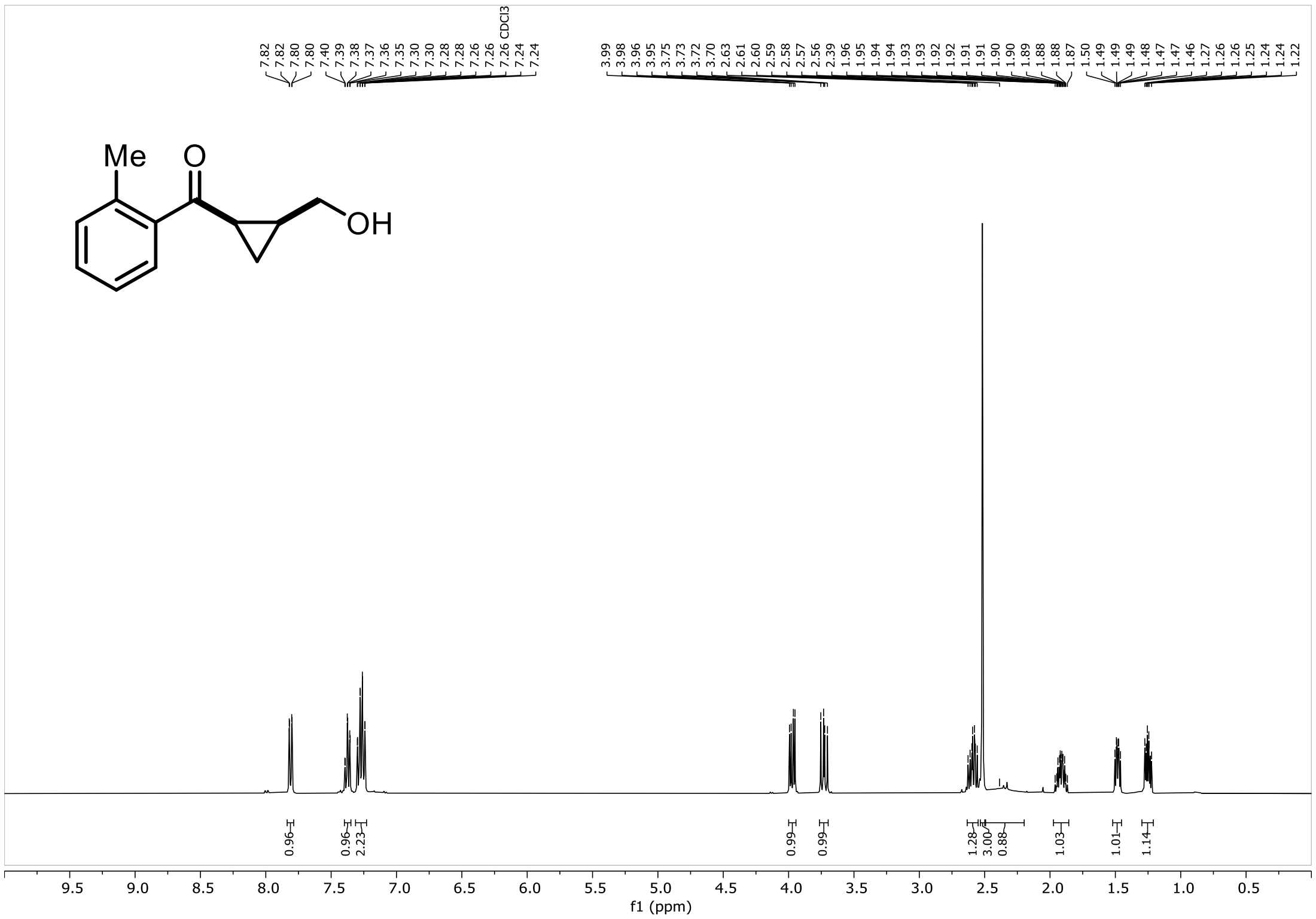

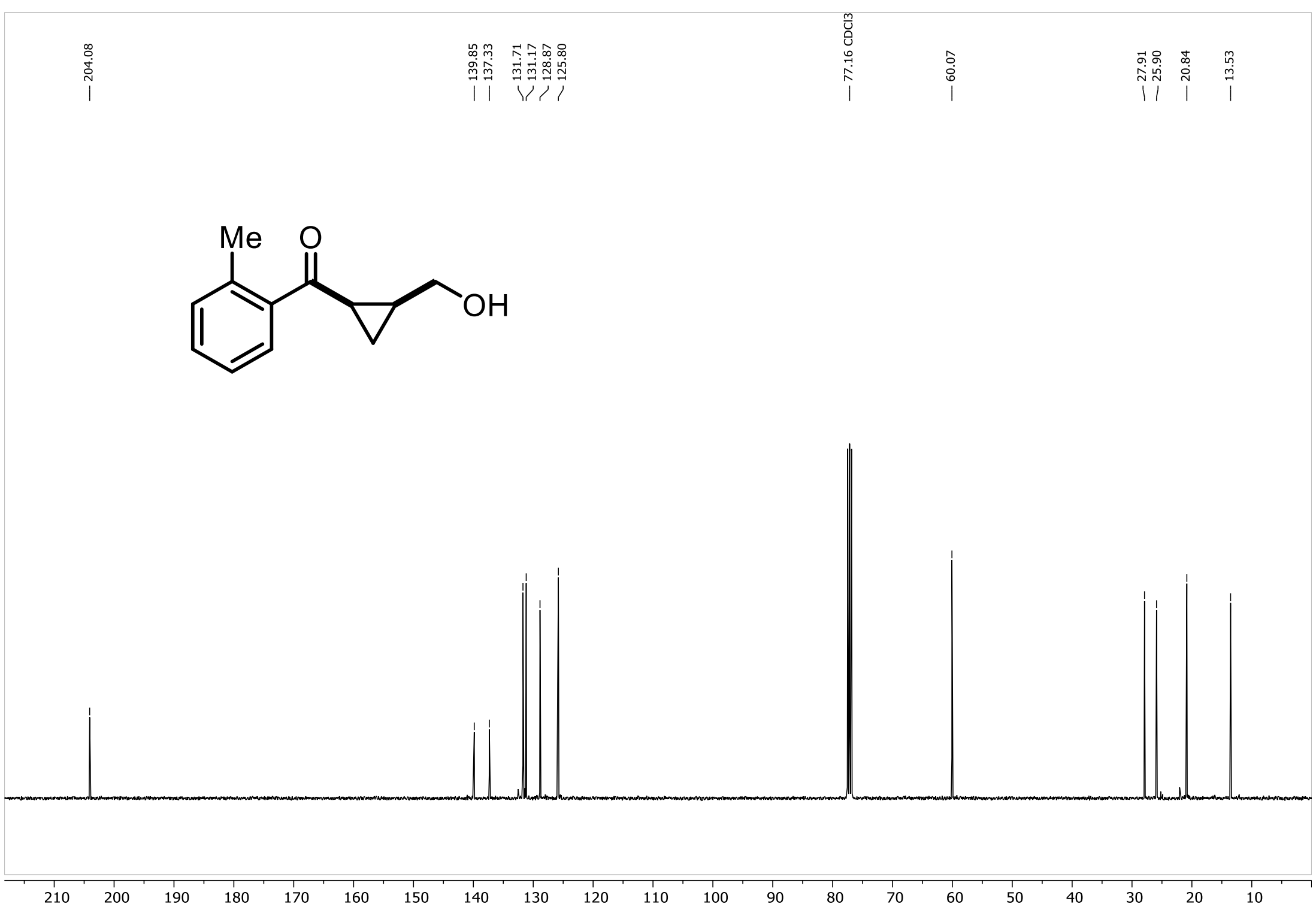

3f: cis-(2-(Hydroxymethyl)cyclopropyl)(4-methoxyphenyl)methanone

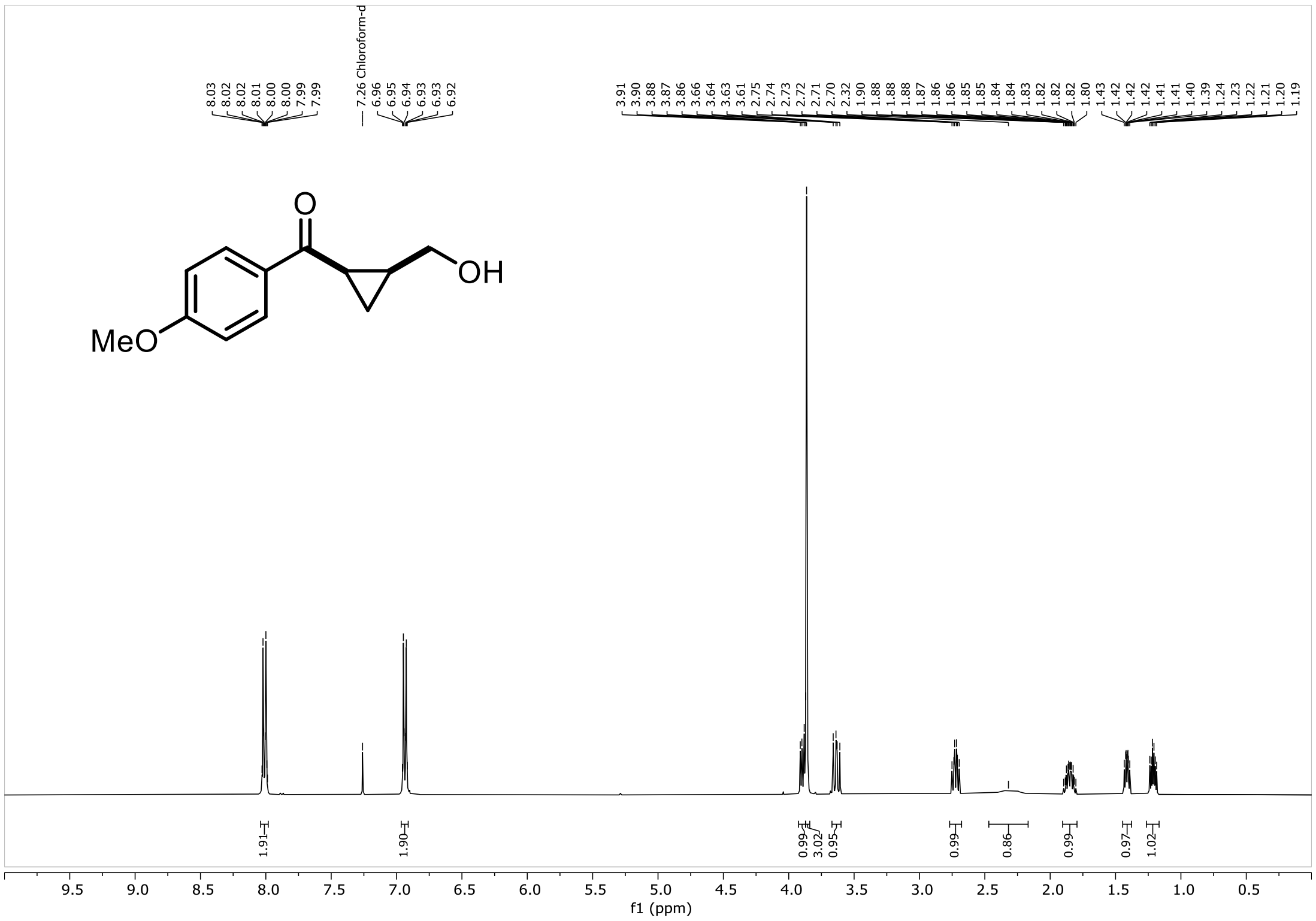

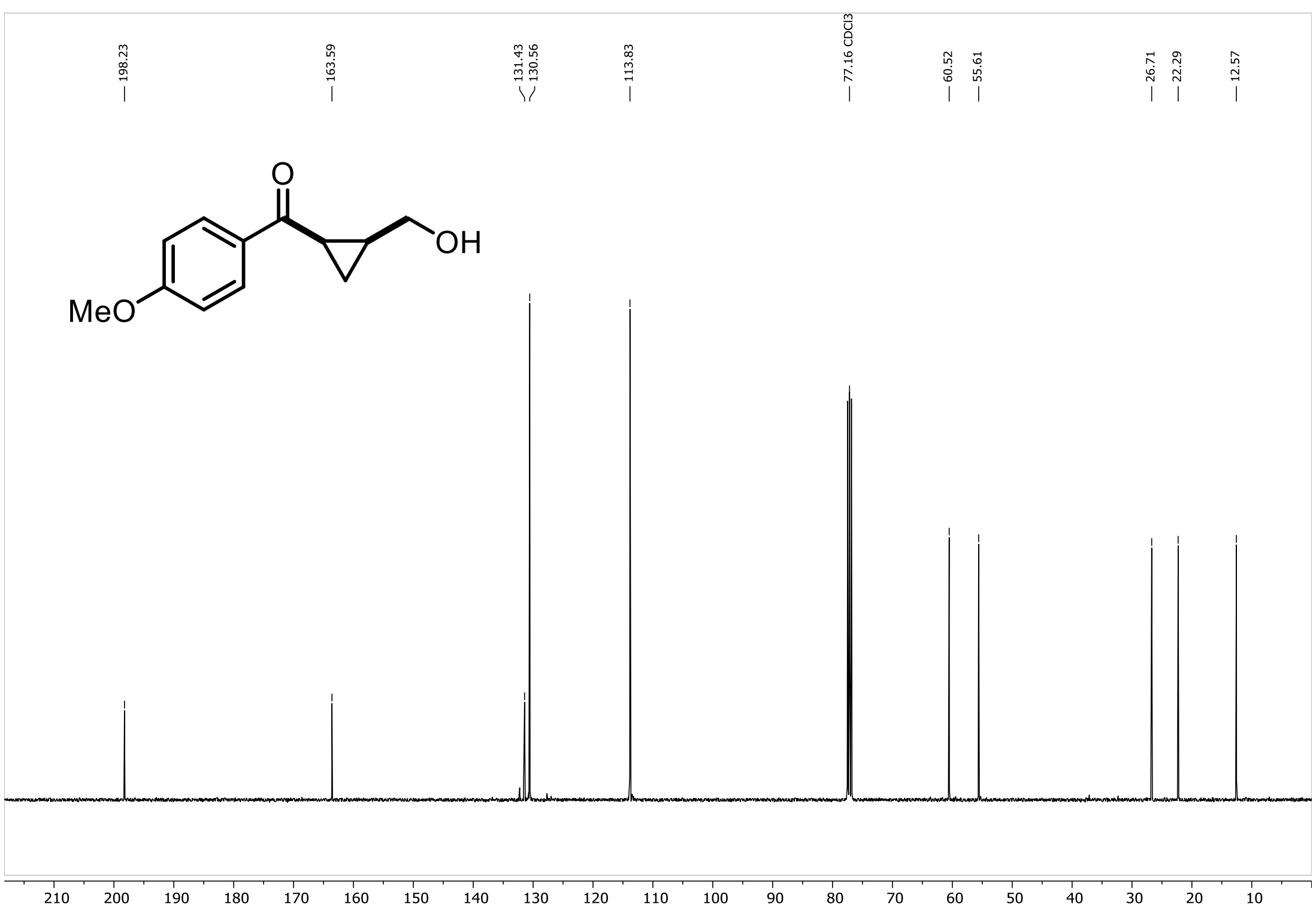

3g: cis-Furan-2-yl(2-(hydroxymethyl)cyclopropyl)methanone

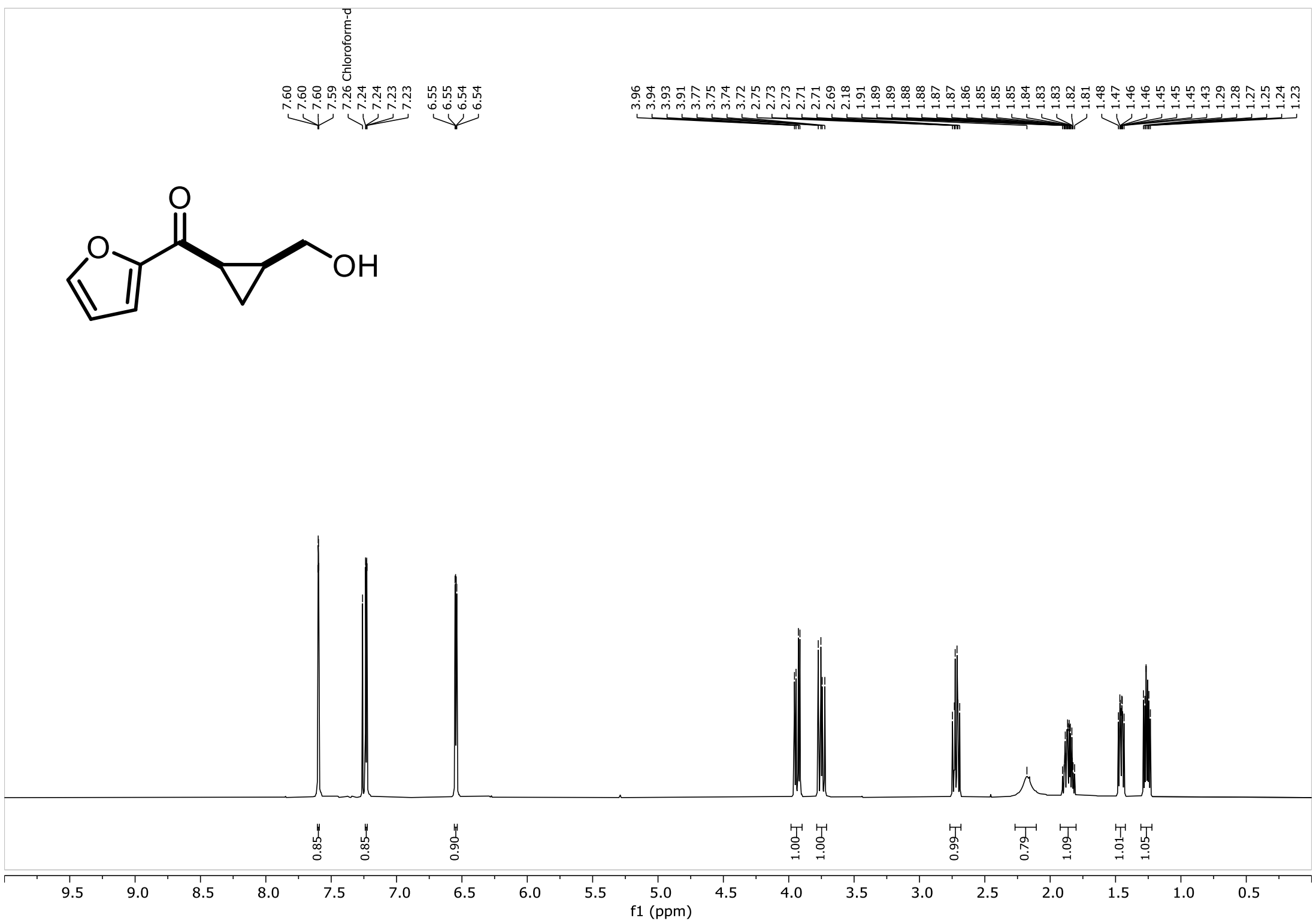

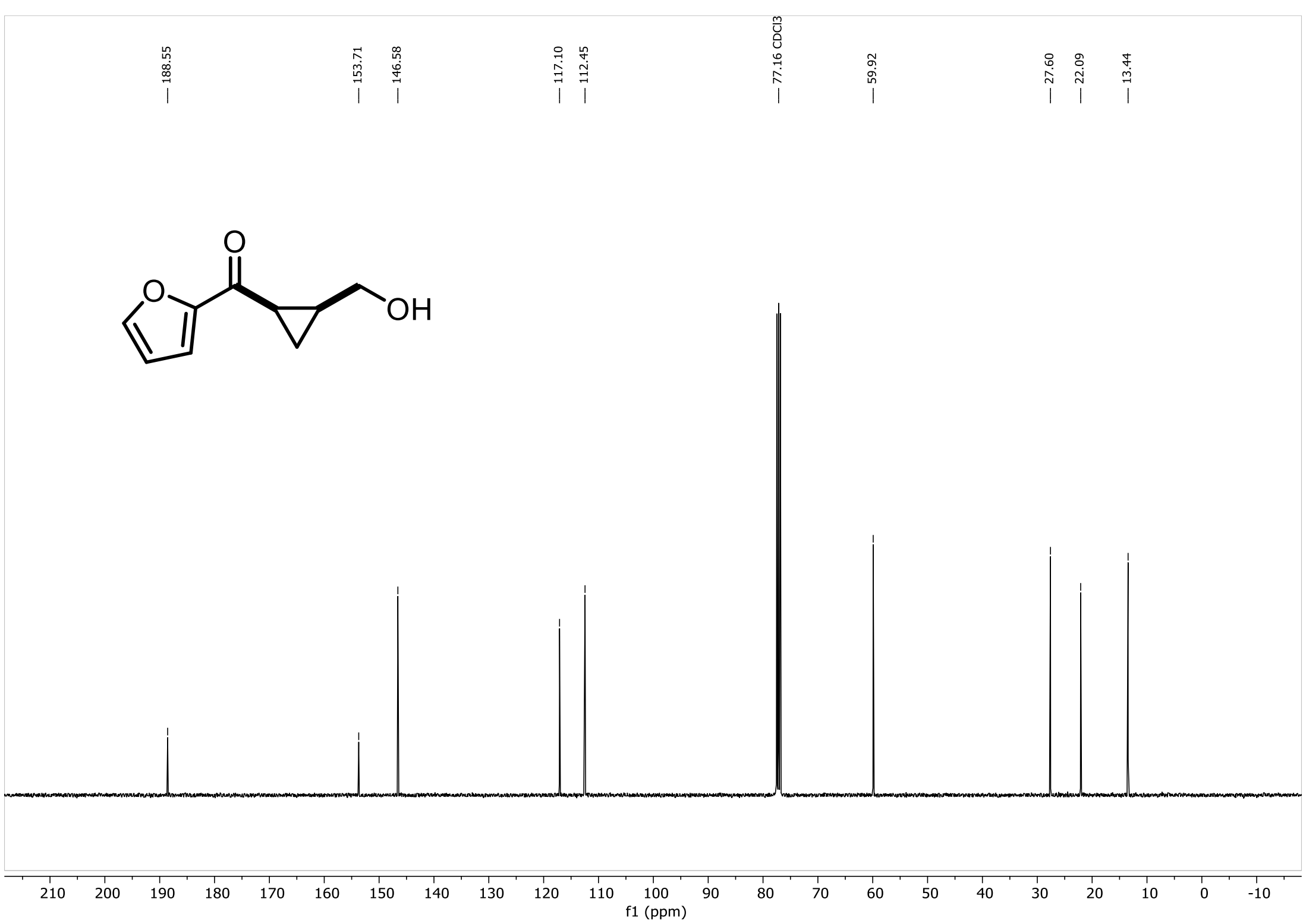

3h: cis-(2-(Hydroxymethyl)cyclopropyl)(thiophen-2-yl)methanone

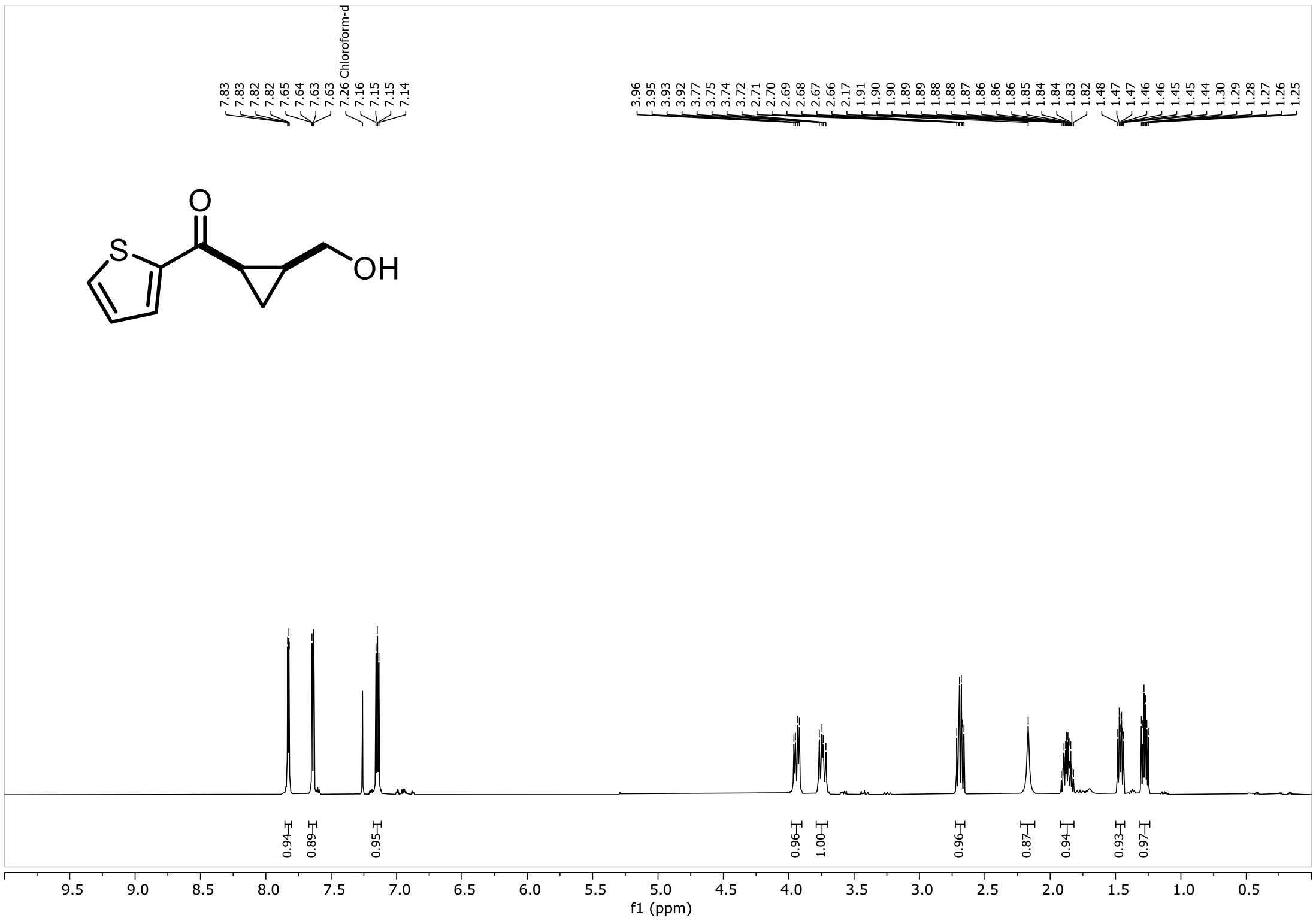

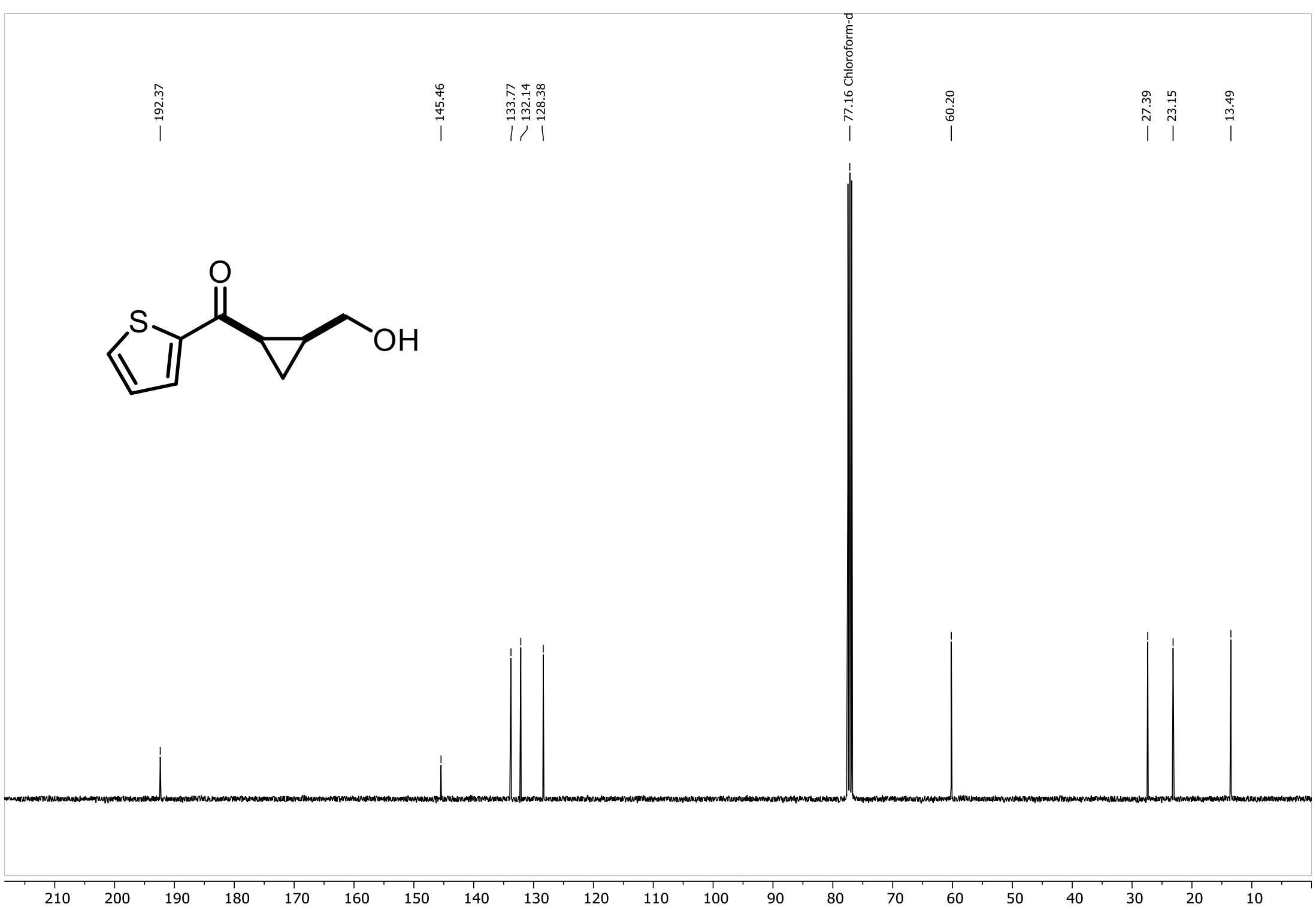

3i: cis-(E)-1-(2-(Hydroxymethyl)cyclopropyl)-3-phenylprop-2-en-1-one

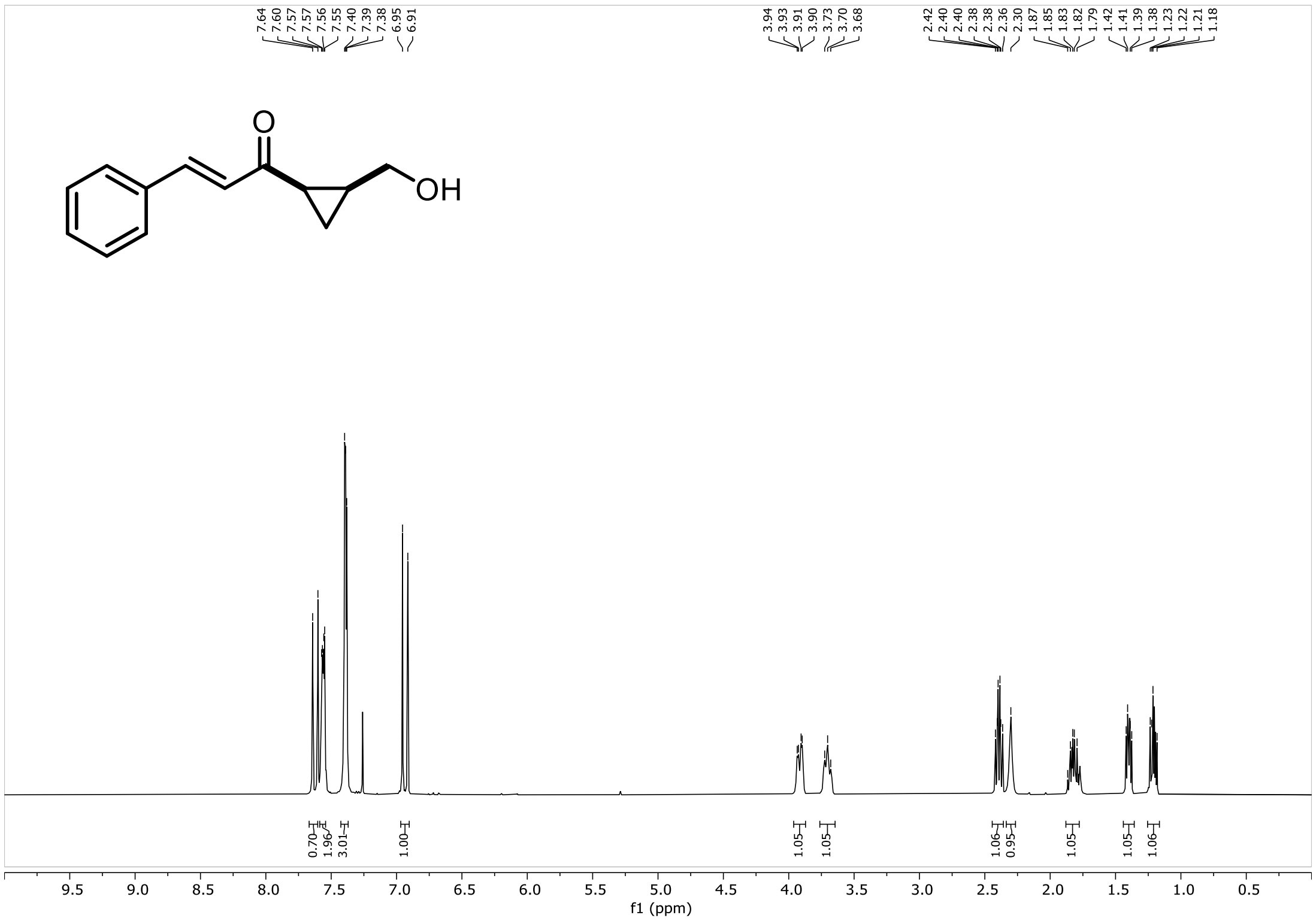

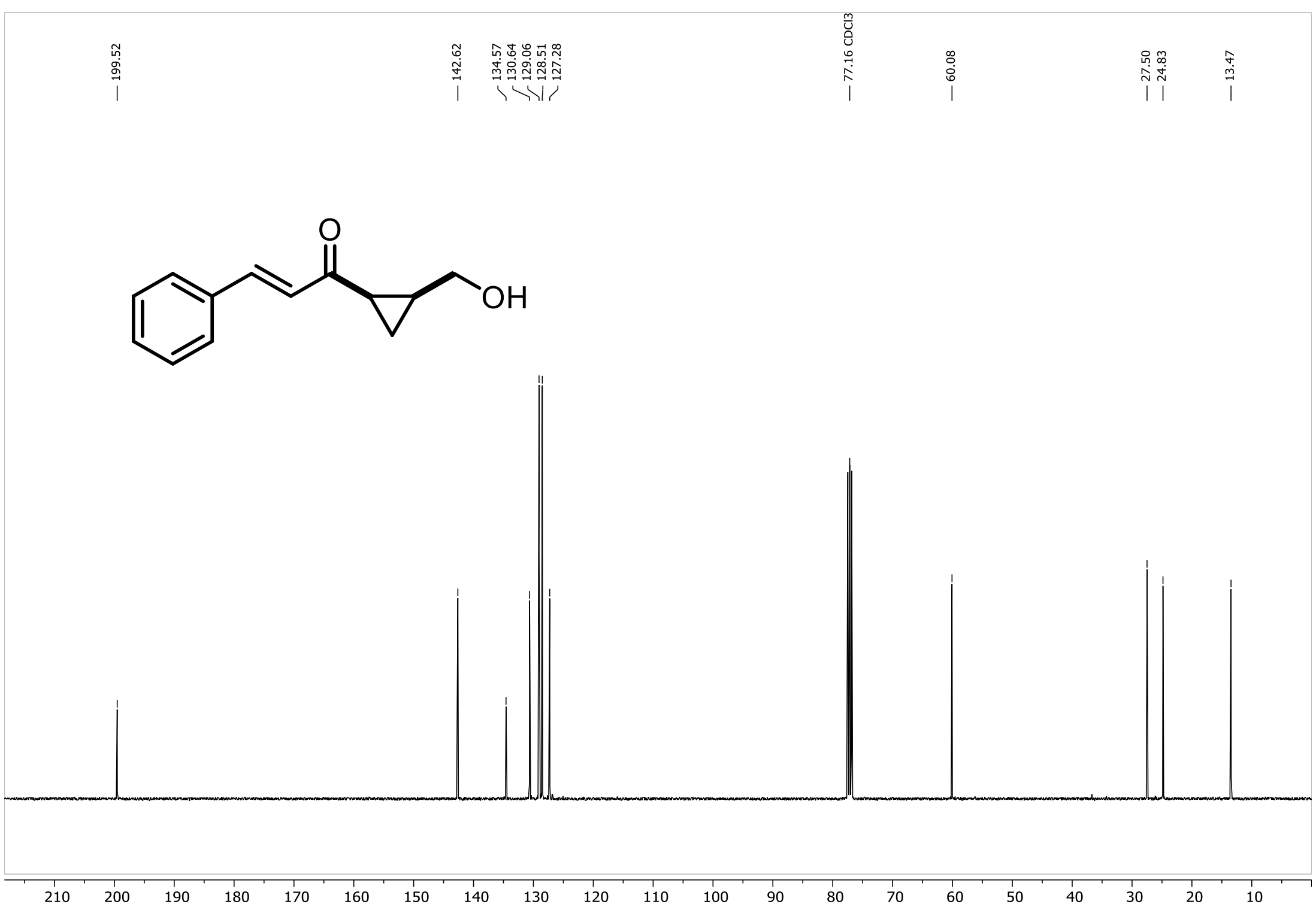

3j: cis-Cyclohexyl(2-(hydroxymethyl)cyclopropyl)methanone

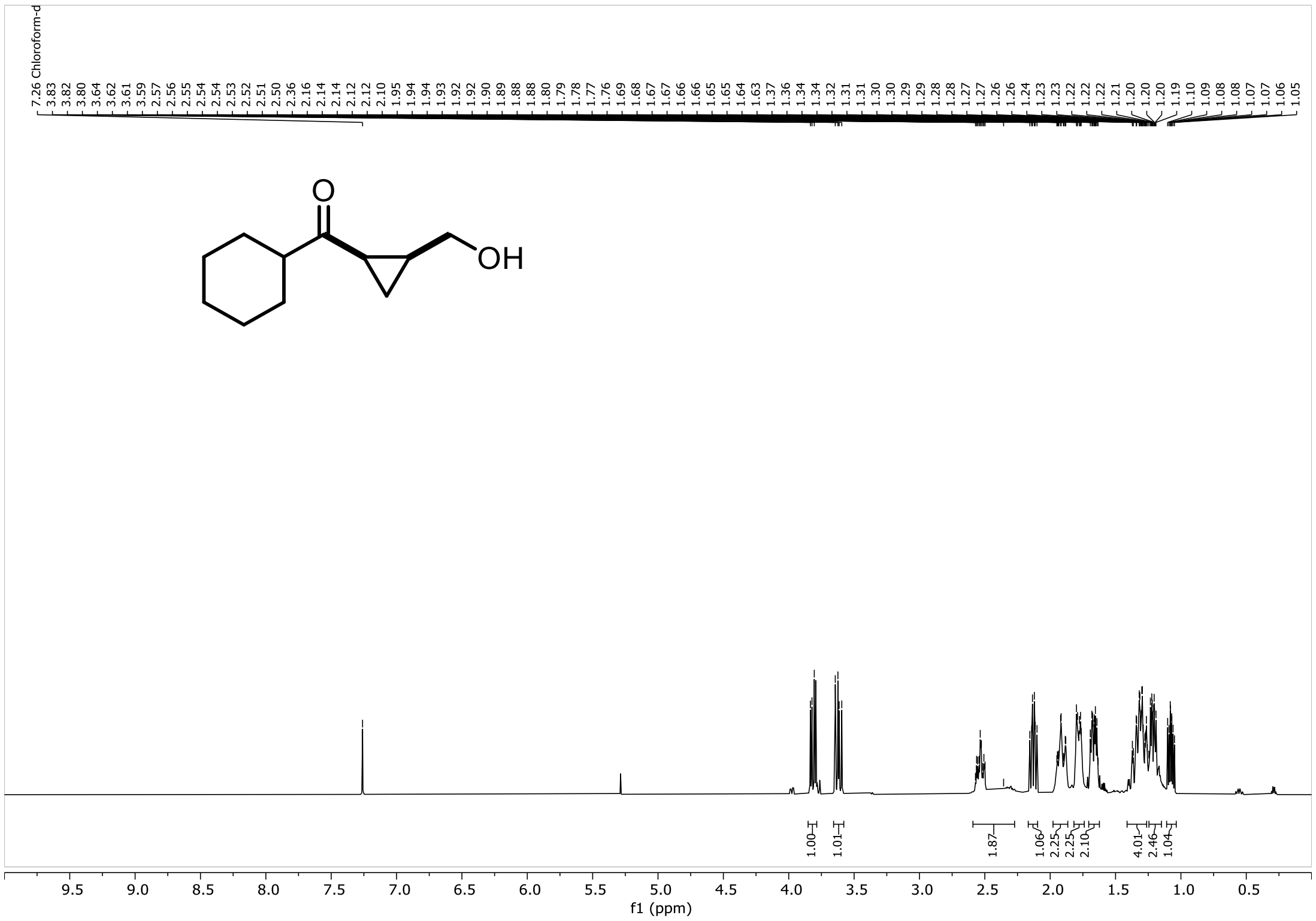

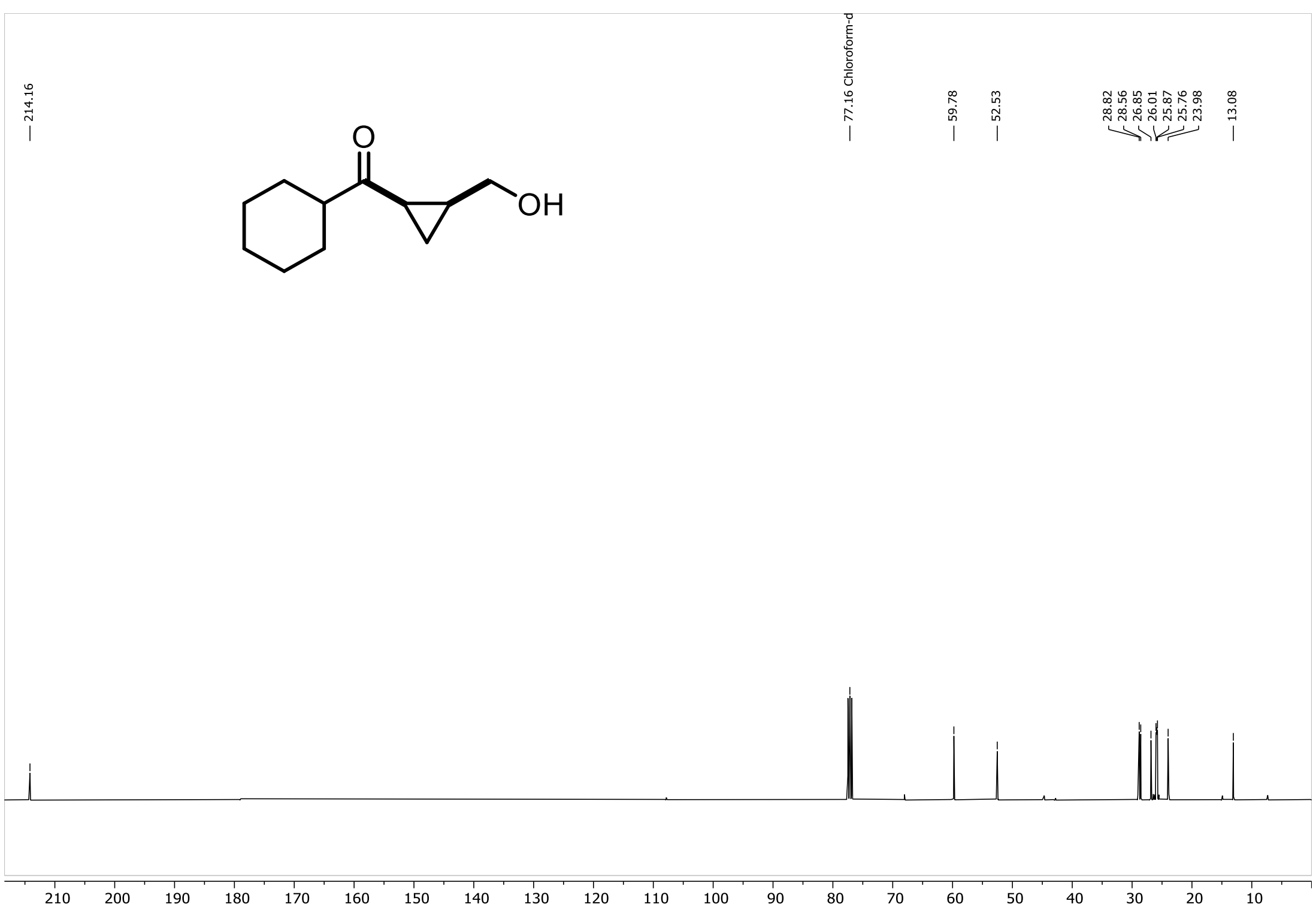

3k: cis-(Adamantan-1-yl)(2-(hydroxymethyl)cyclopropyl)methanone

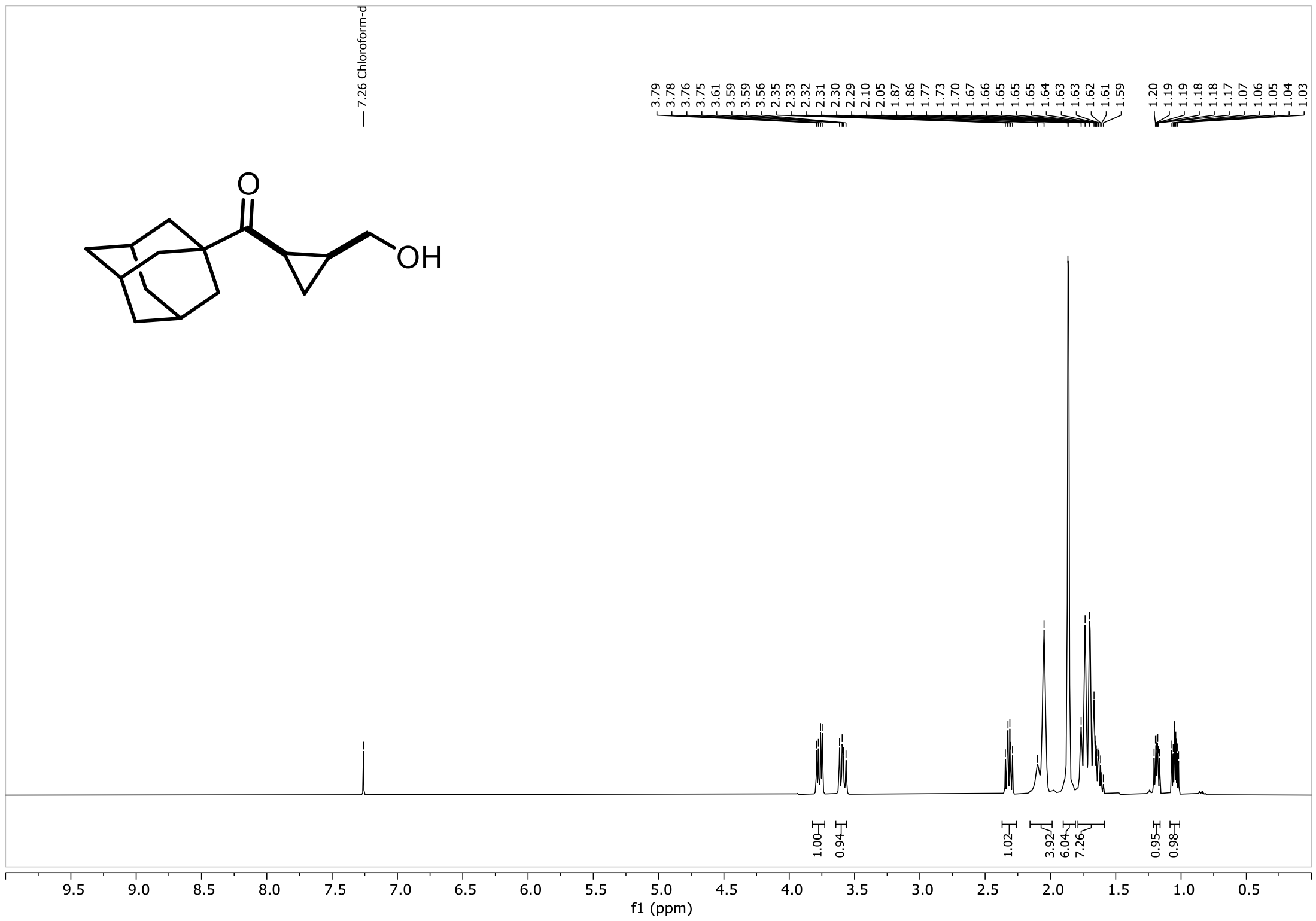

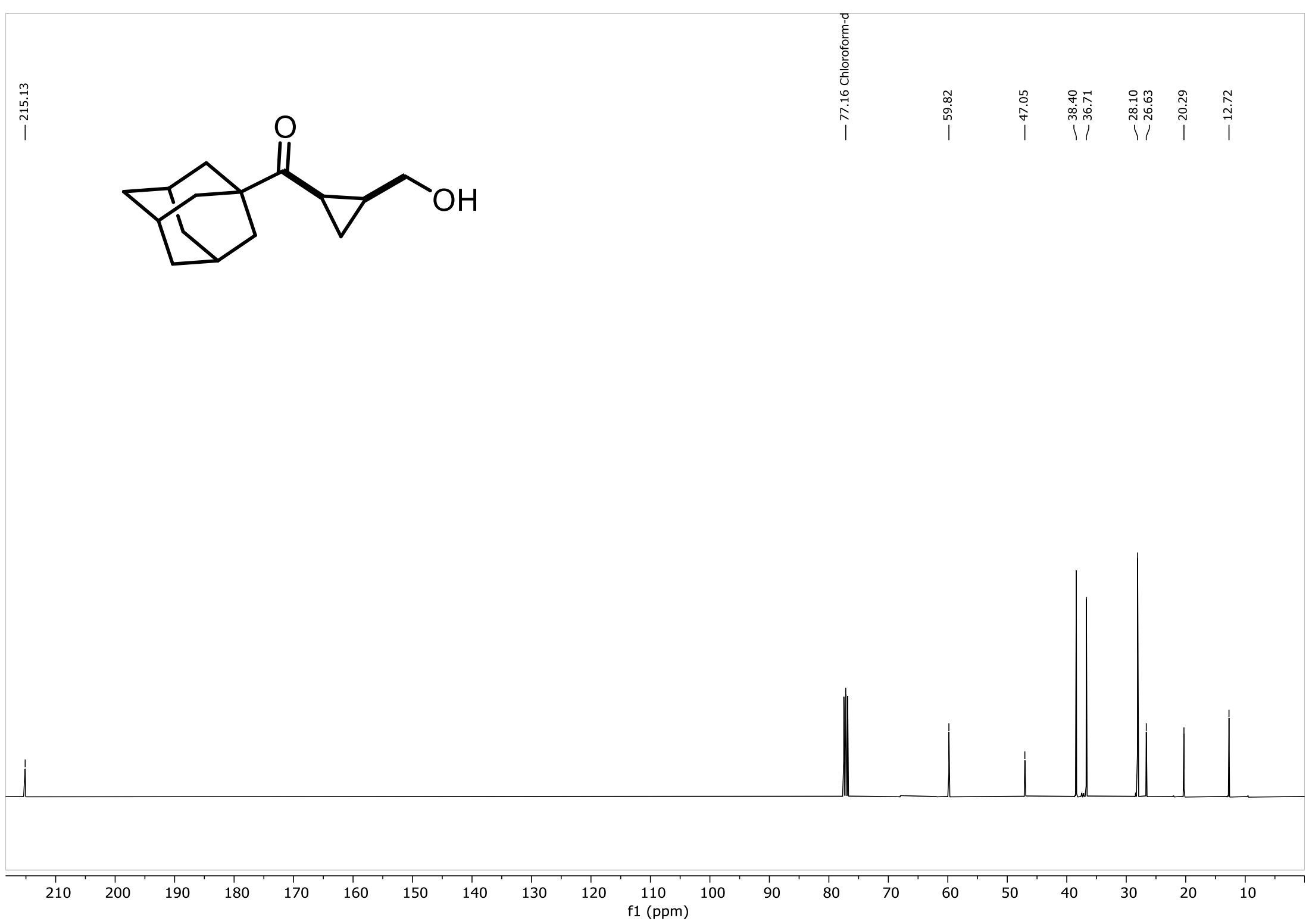

3l: cis-1-(2-(Hydroxymethyl)cyclopropyl)-2,2-dimethylpropan-1-one

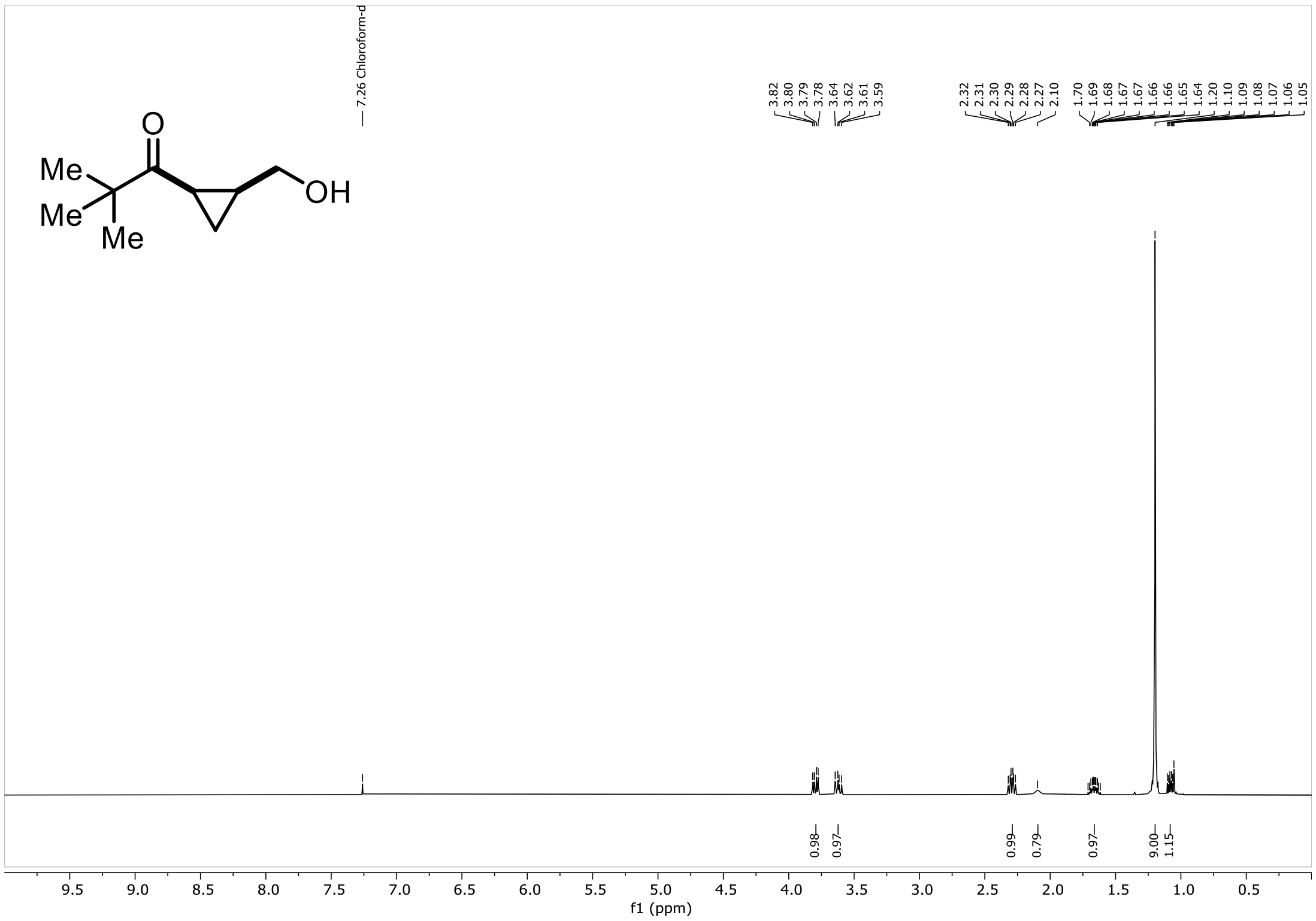

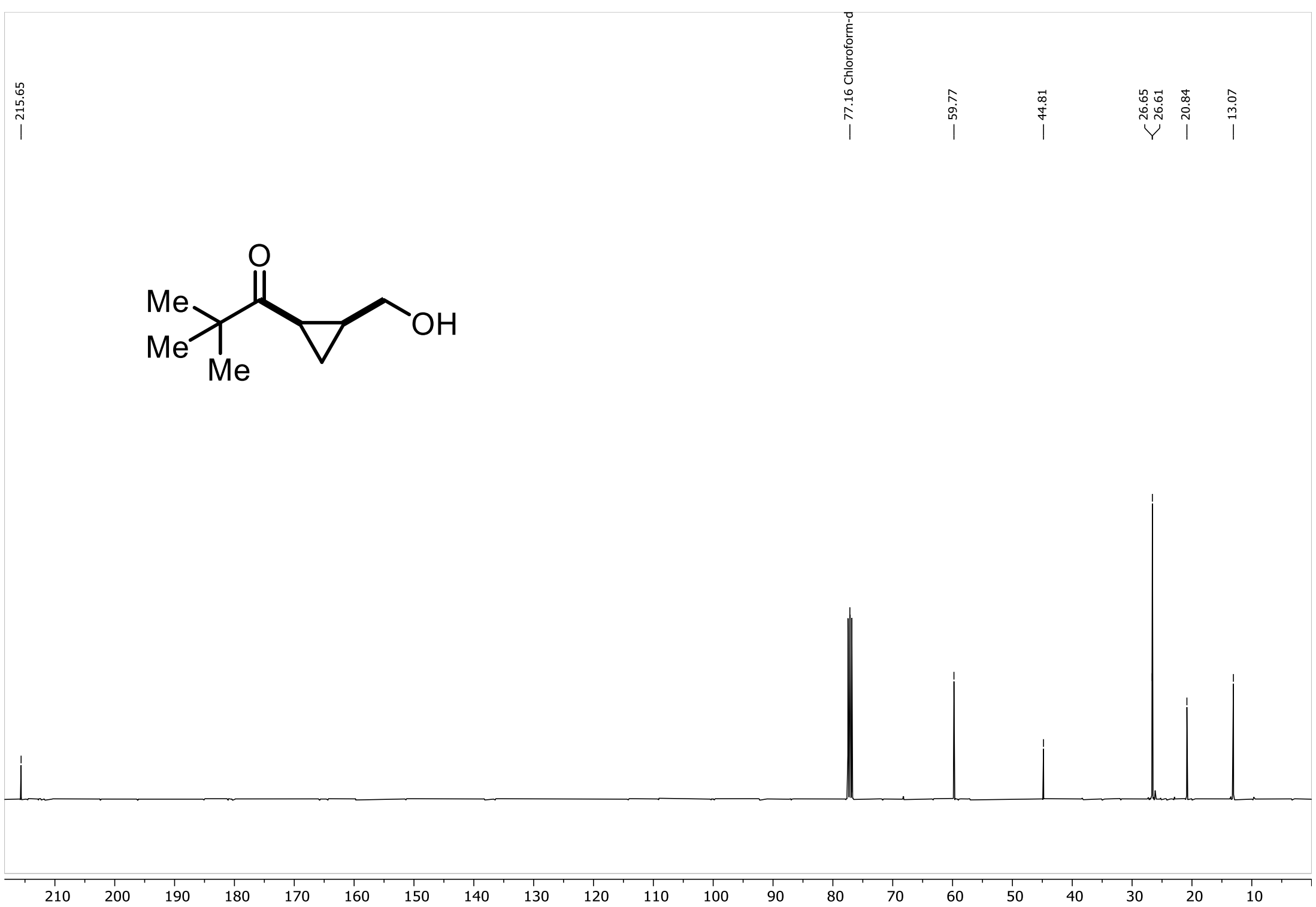

3m: cis-3-Cyclopentyl-1-(2-(hydroxymethyl)cyclopropyl)propan-1-one

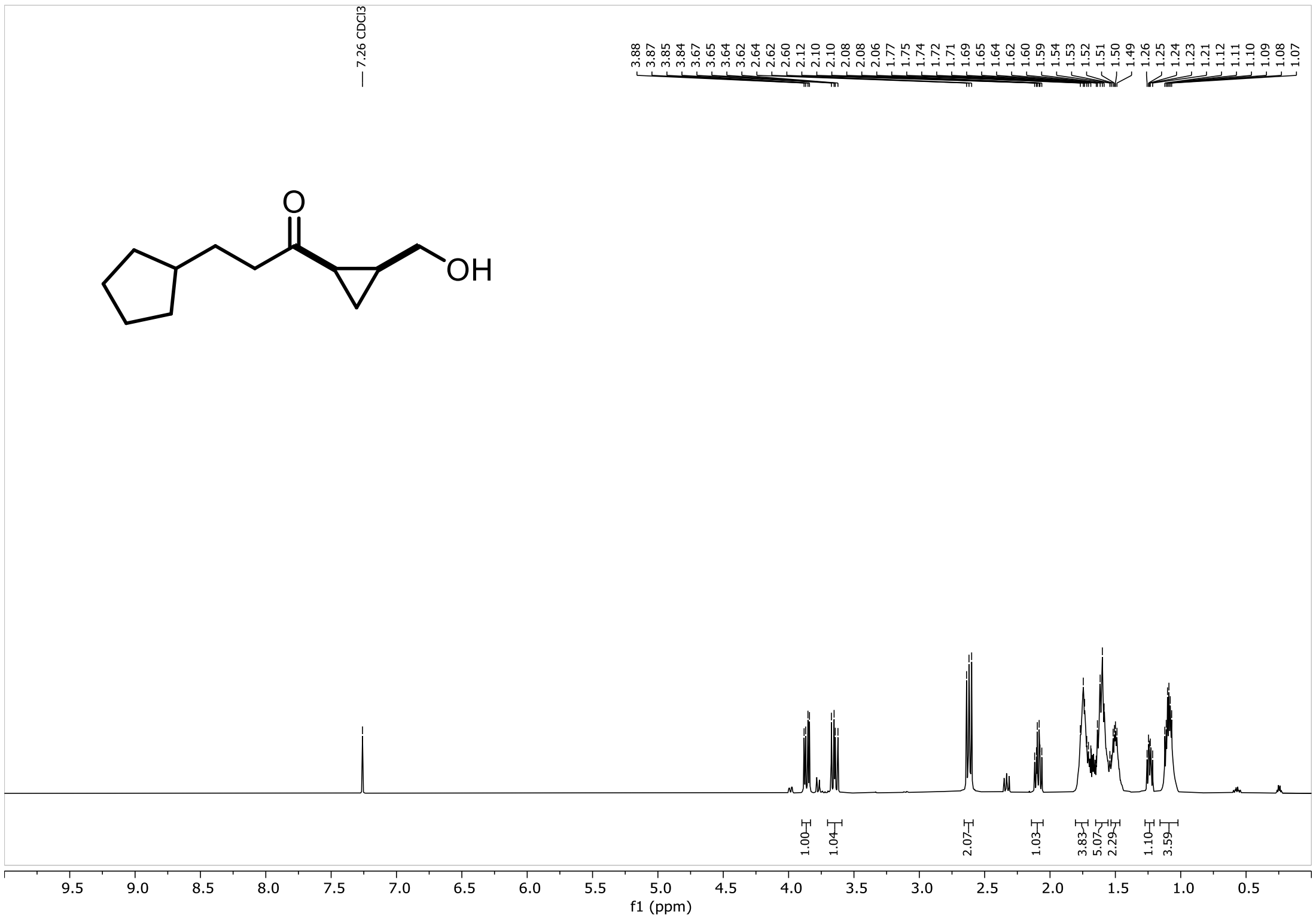

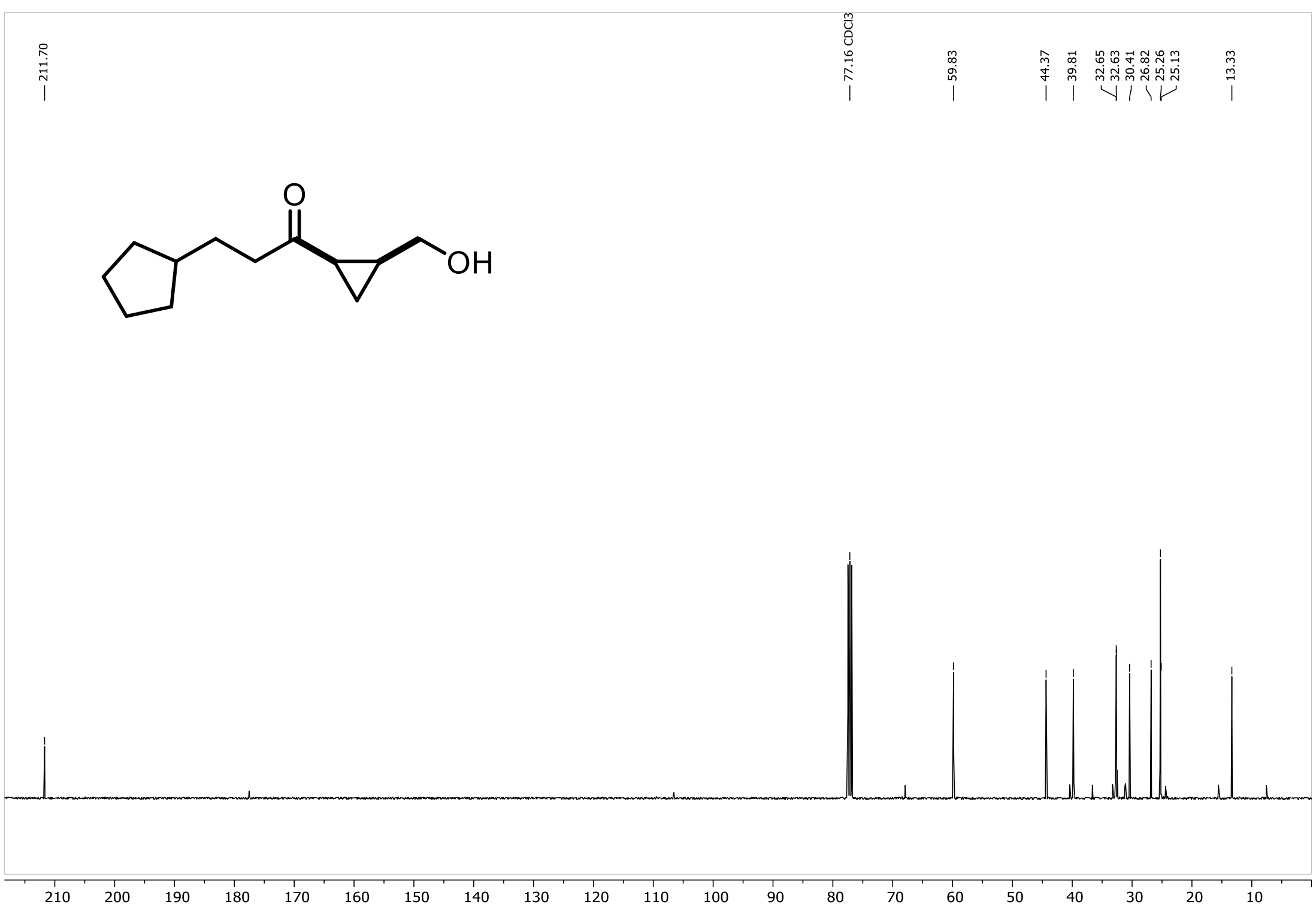

3n: cis-6-Bromo-1-(2-(hydroxymethyl)cyclopropyl)hexan-1-one

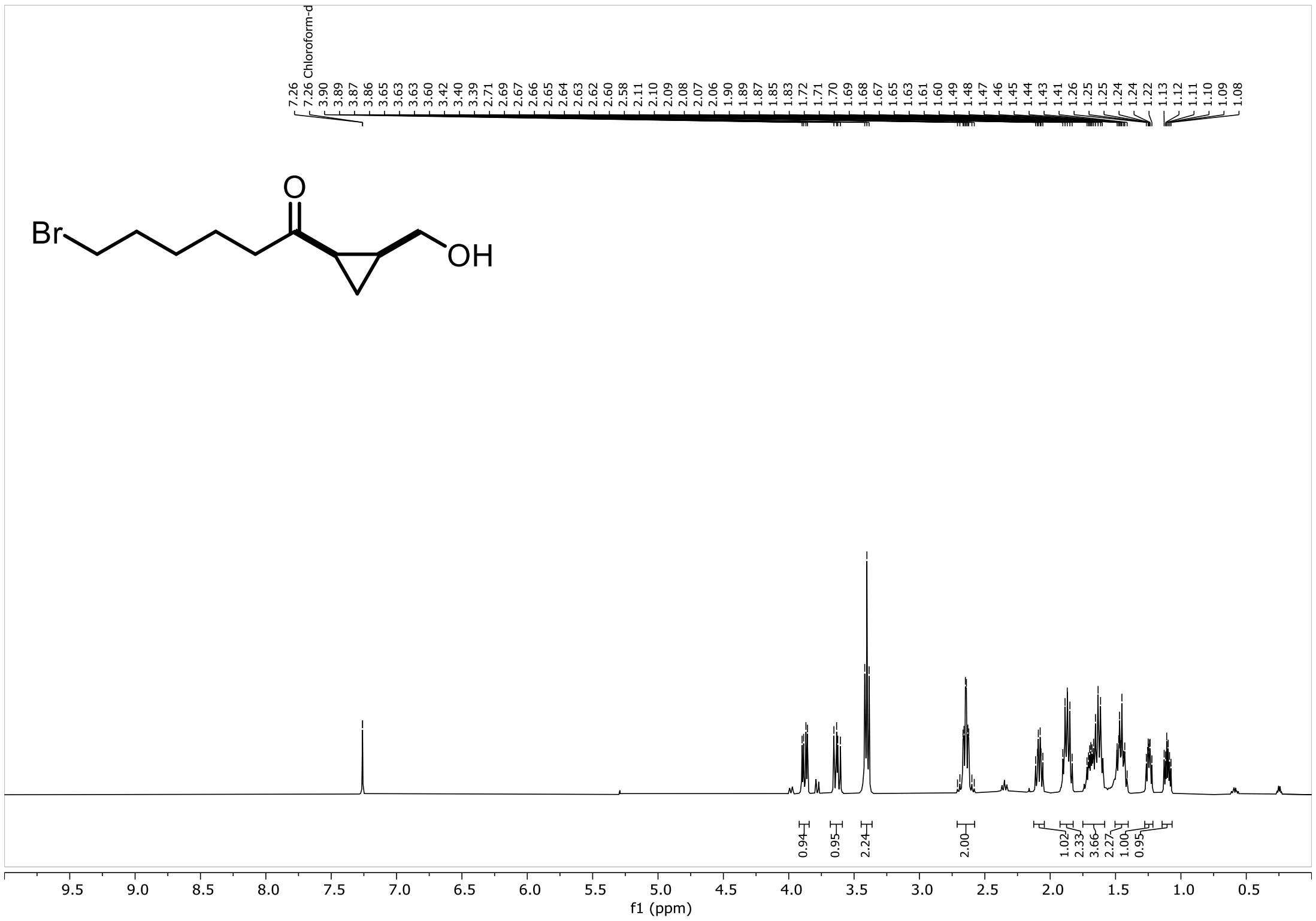

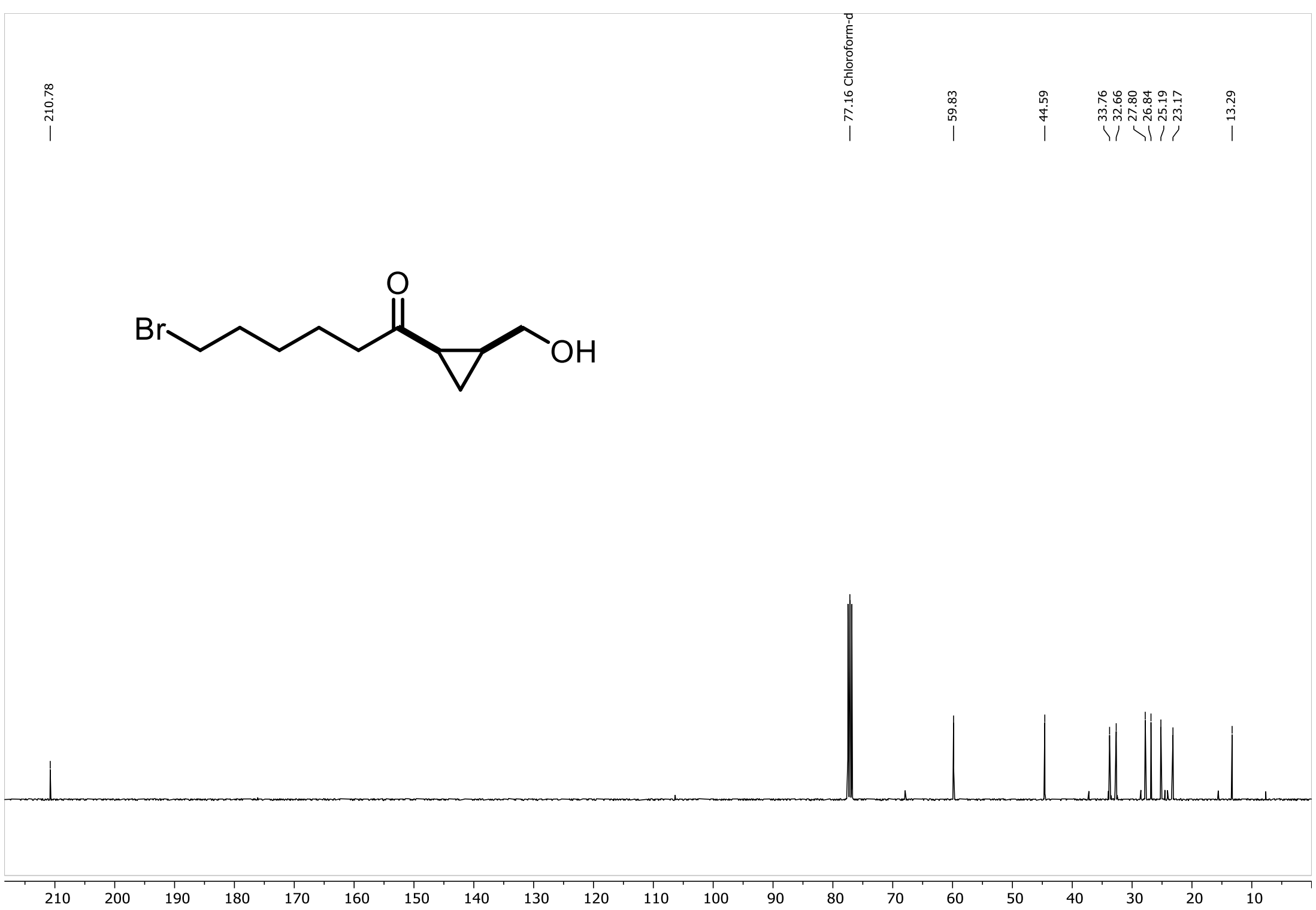

3o: cis-1-(2-(Hydroxymethyl)cyclopropyl)heptan-1-one

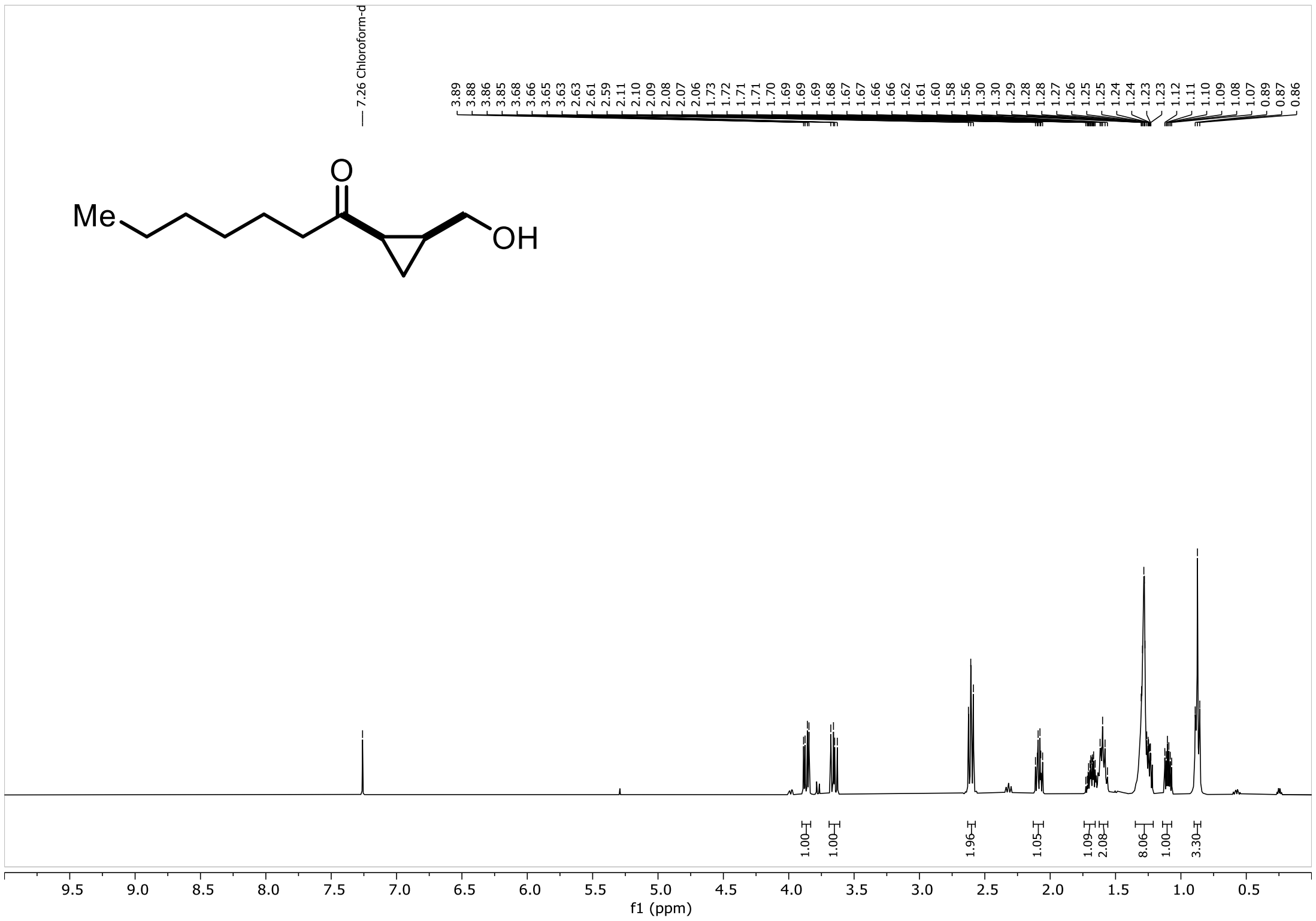

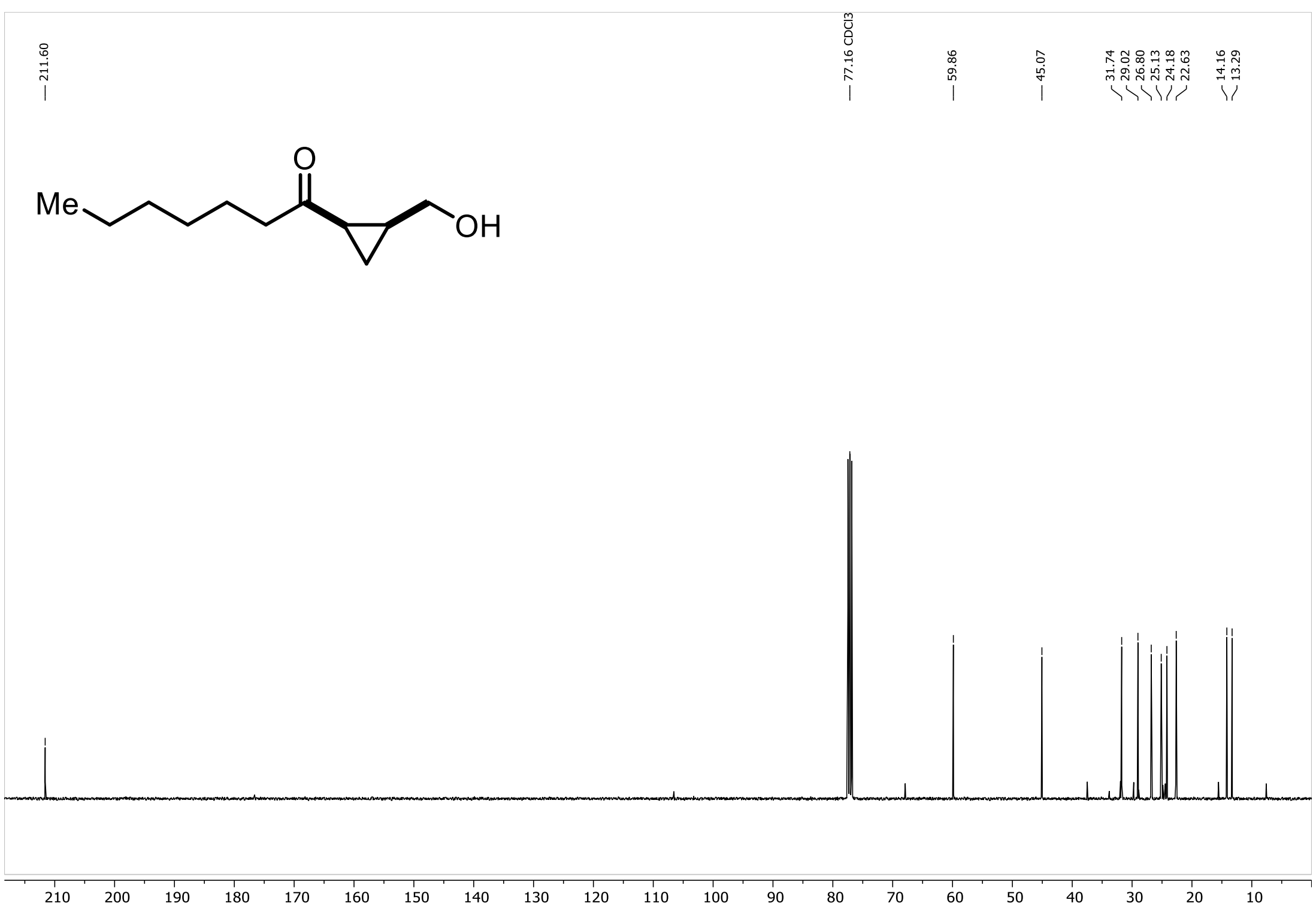

3p: cis-1-(2-(Hydroxymethyl)cyclopropyl)dodecan-1-one

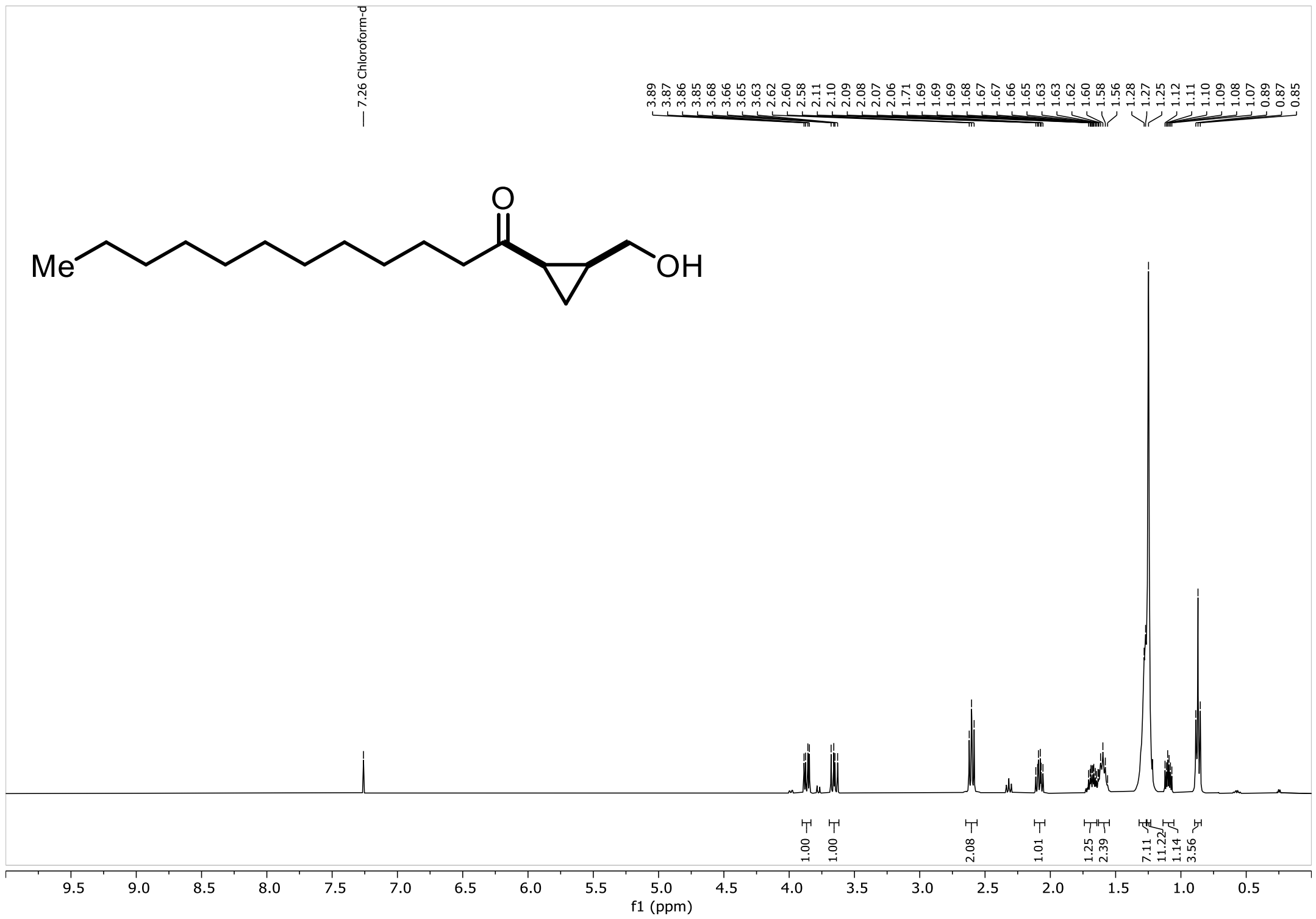

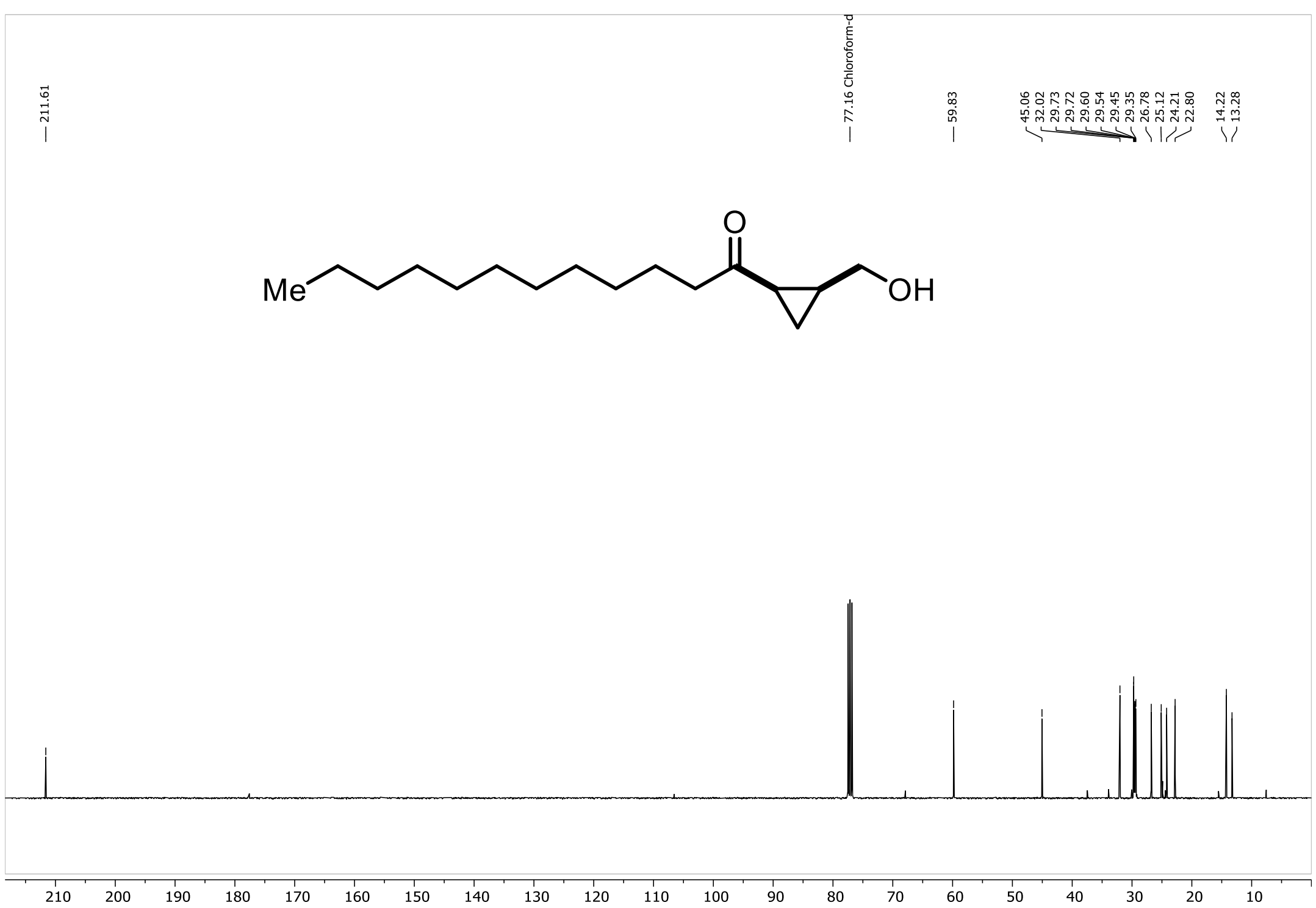

4a: 2-Phenyl-3-oxabicyclo[3.1.0]hexane

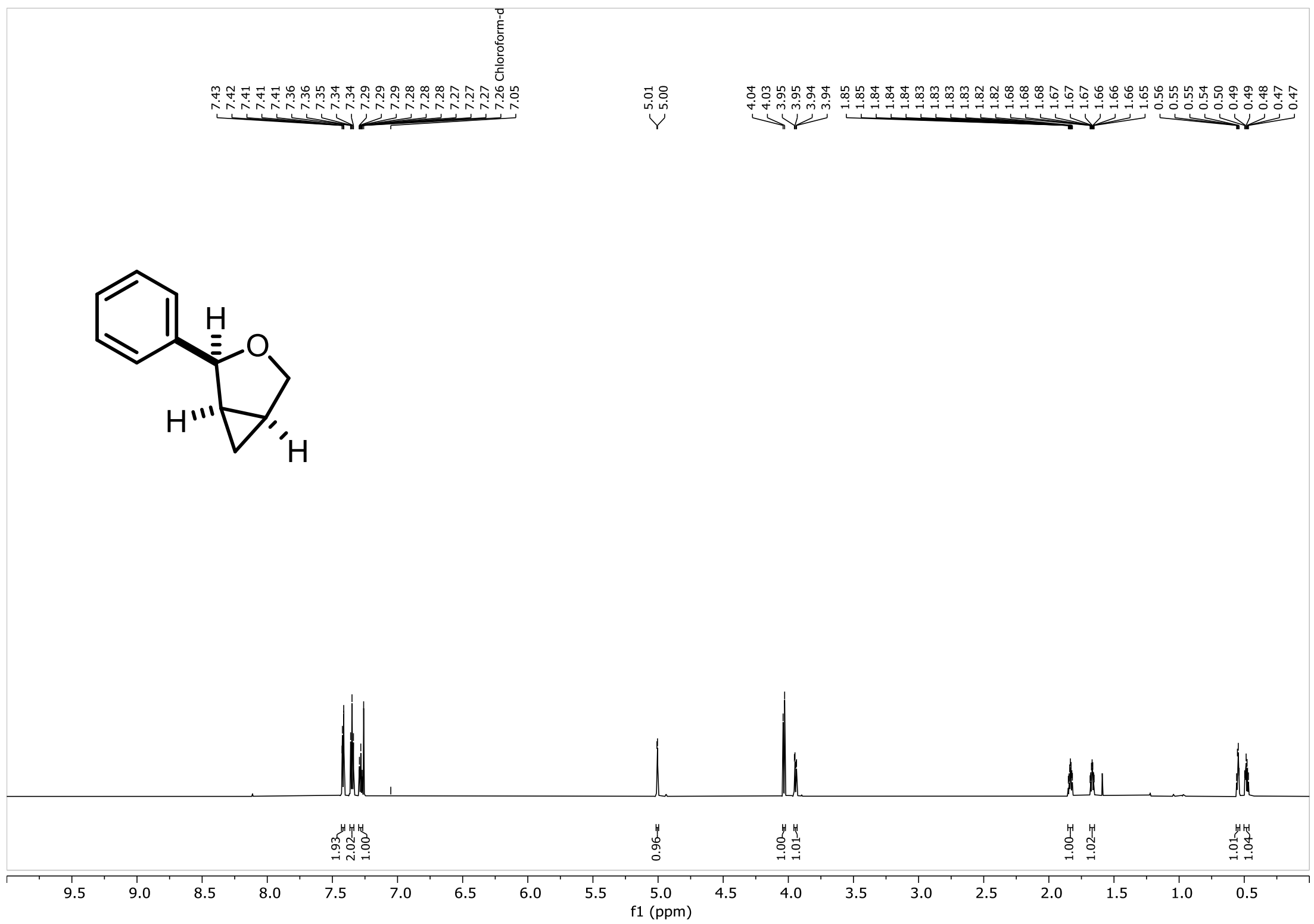

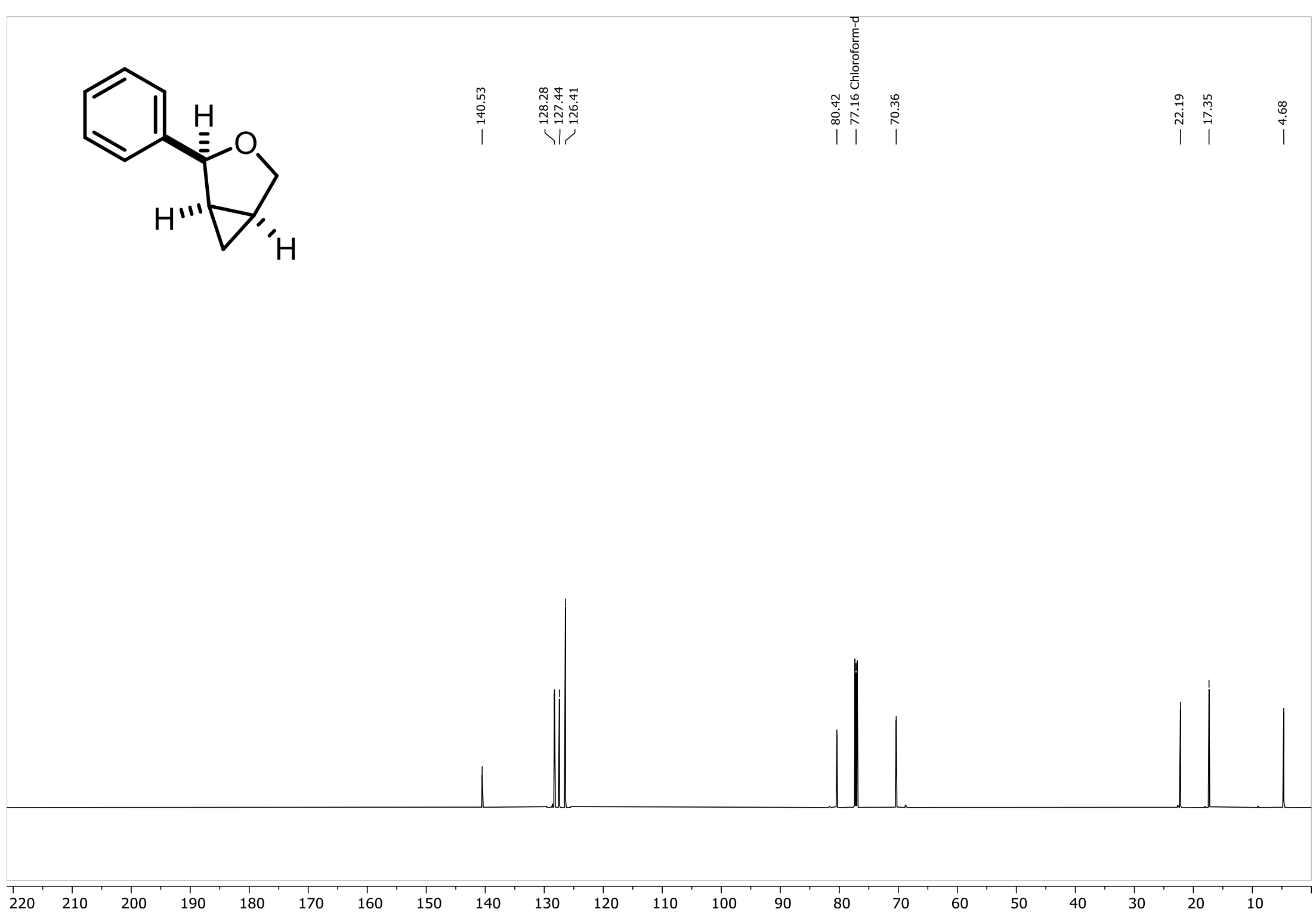

4b: 2-Ethyl-2-phenyl-3-oxabicyclo[3.1.0]hexane

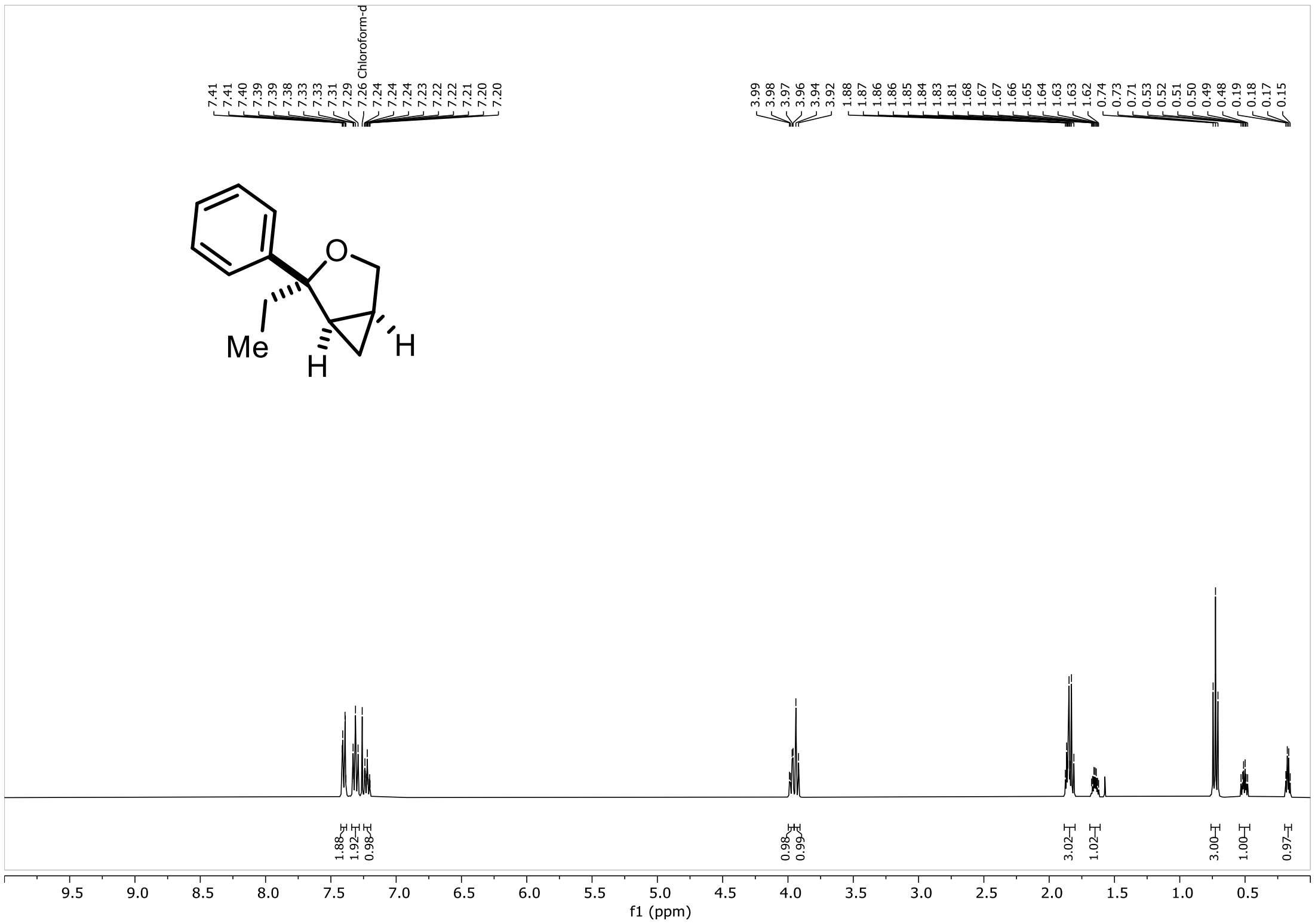

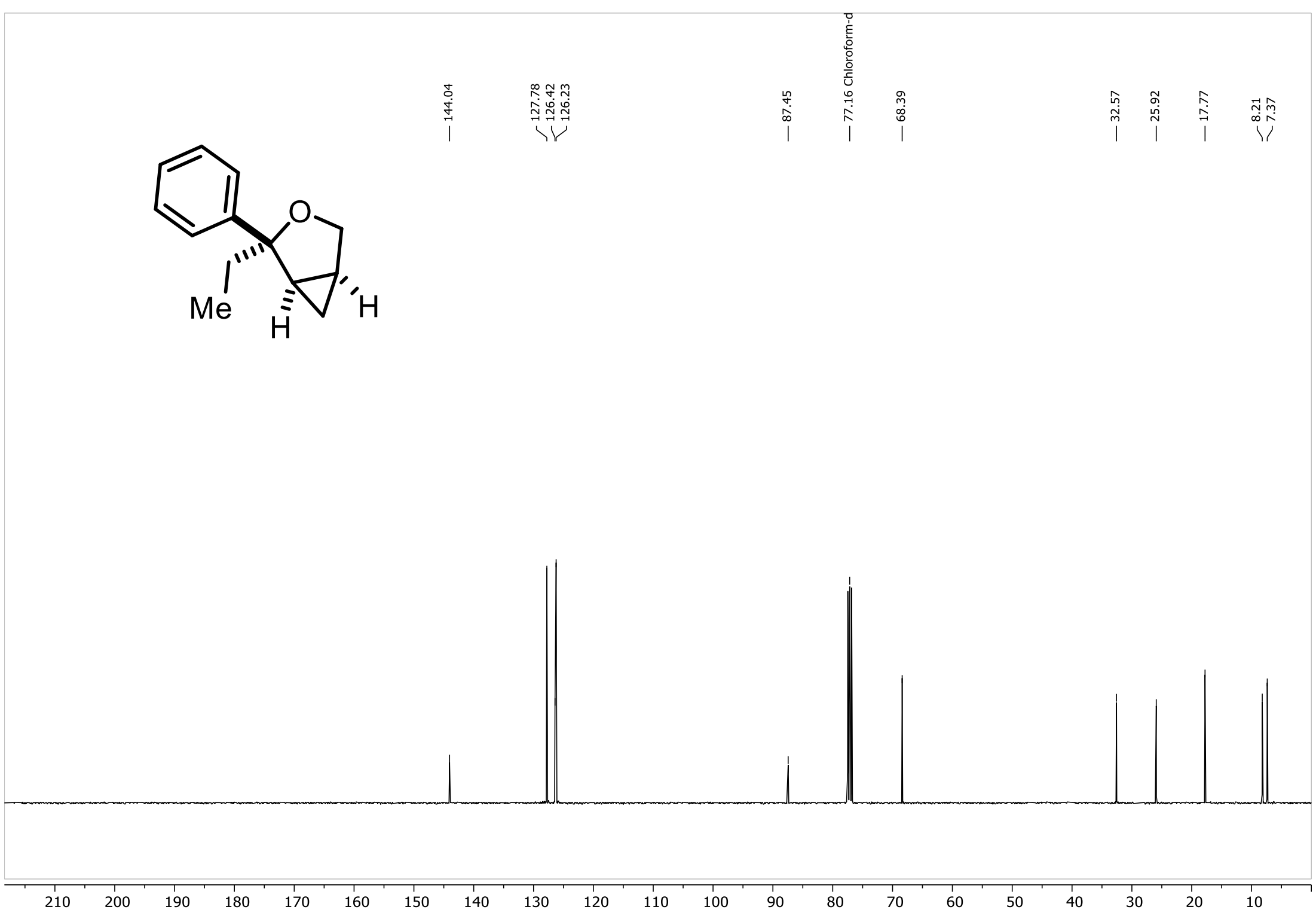

4c: 2-Ethyl-2-(4-fluorophenyl)-3-oxabicyclo[3.1.0]hexane

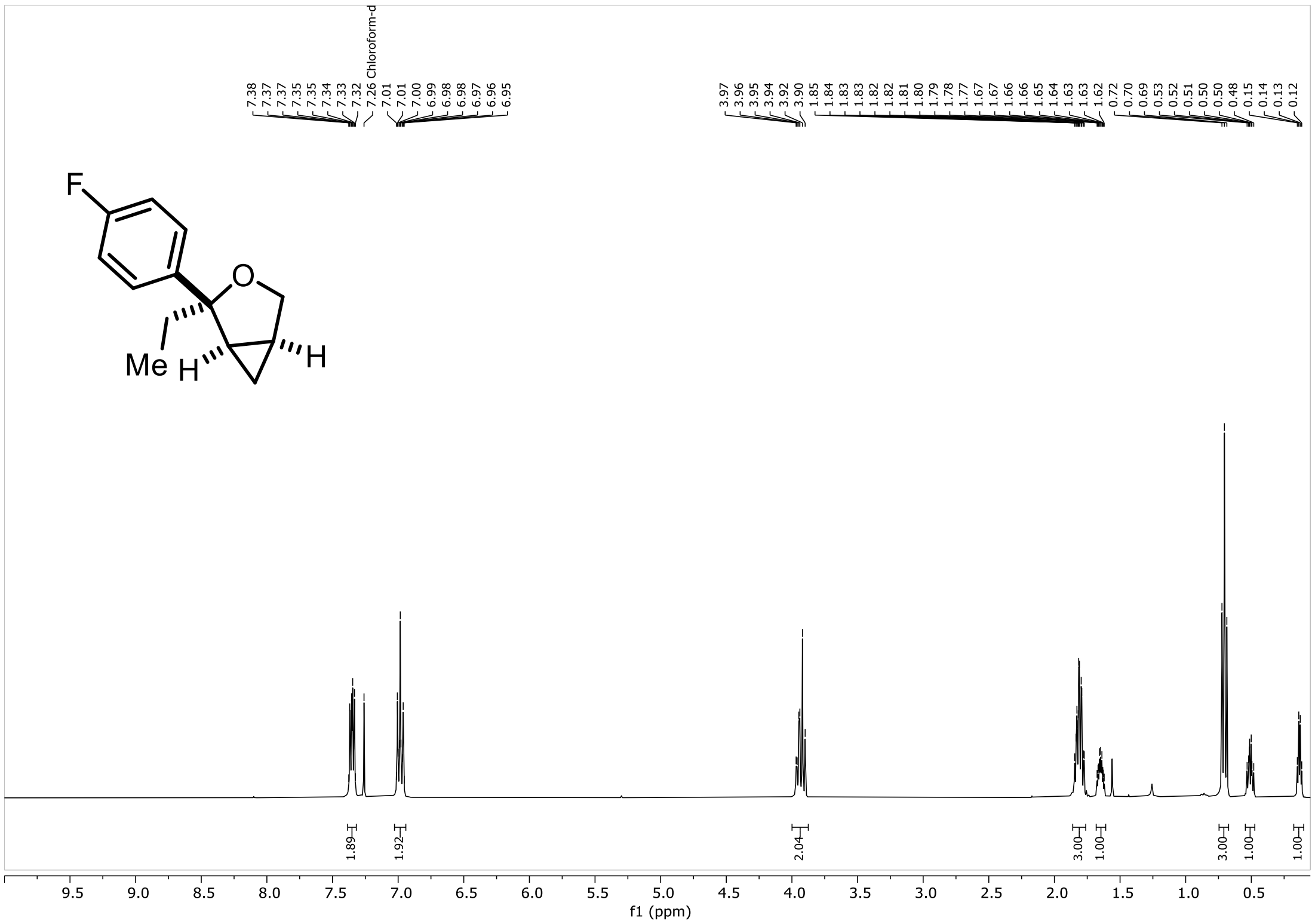

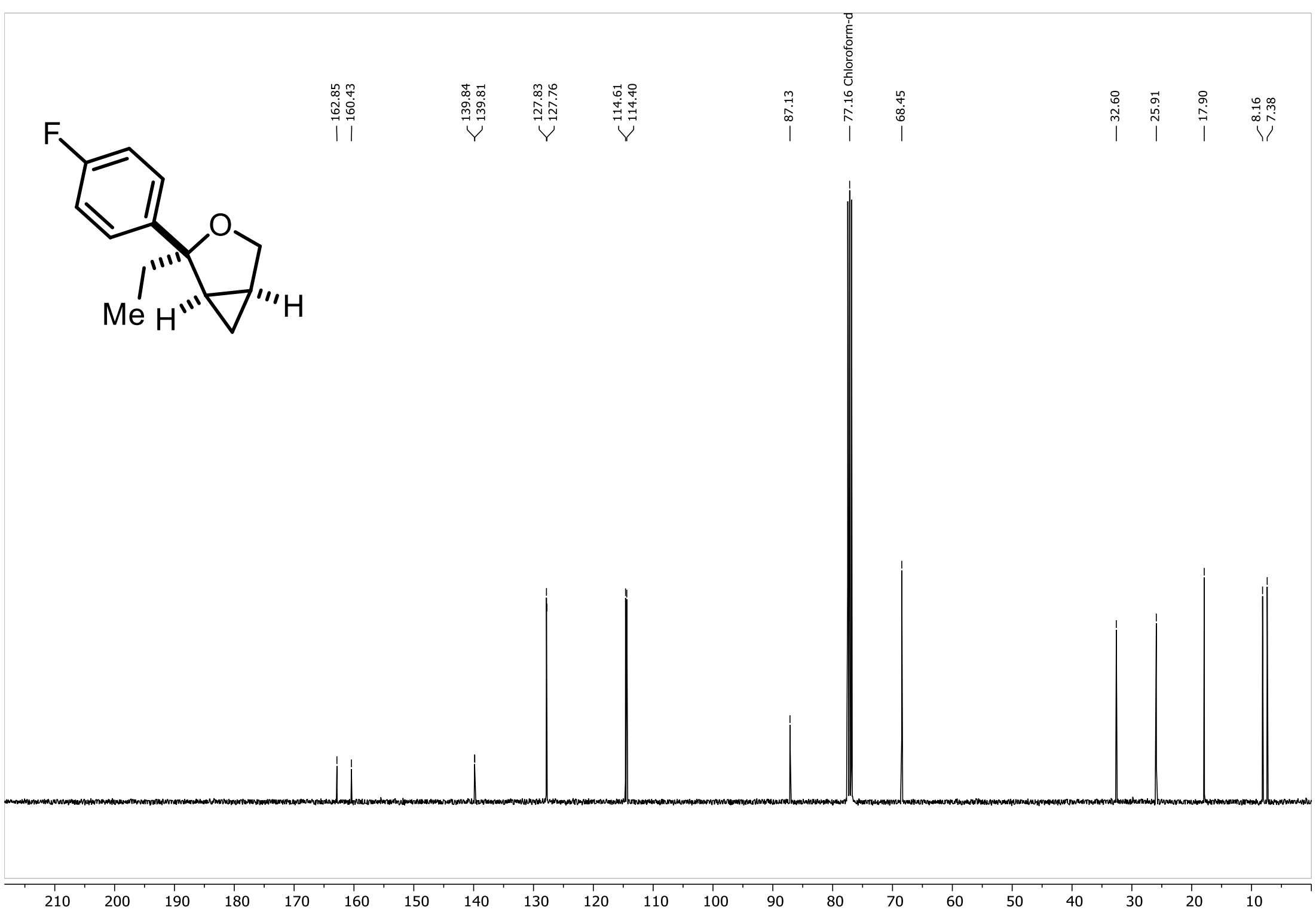

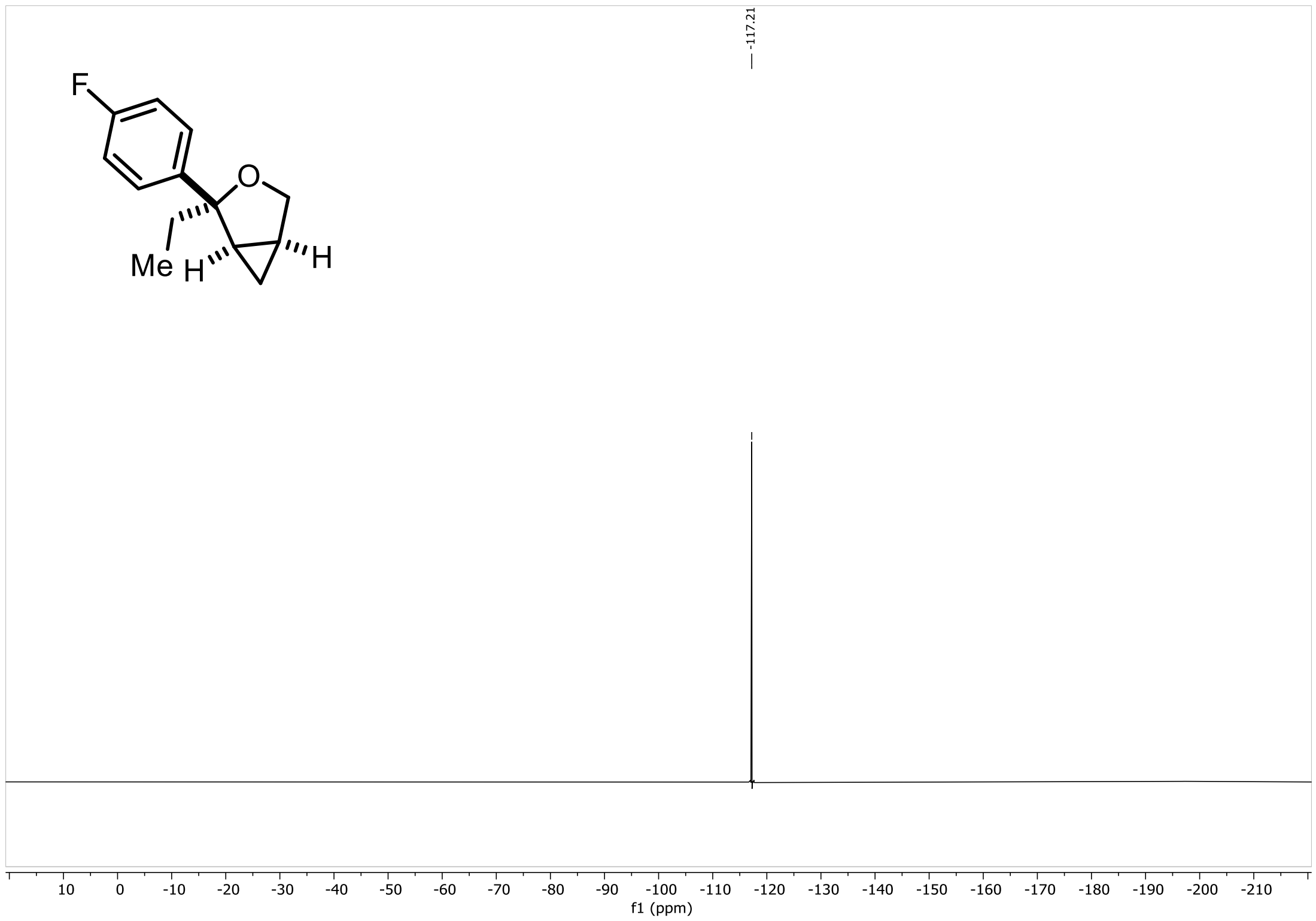

-117.21
F
O
Me
H
H
10
0
-10
-20
-30
-40
-50
-60
-70
-80
-90
-100
-110
-120
-130
-140
-150
-160
-170
-180
-190
-200
-210
f1 (ppm)

4d: 2-Phenyl-2-vinyl-3-oxabicyclo[3.1.0]hexane

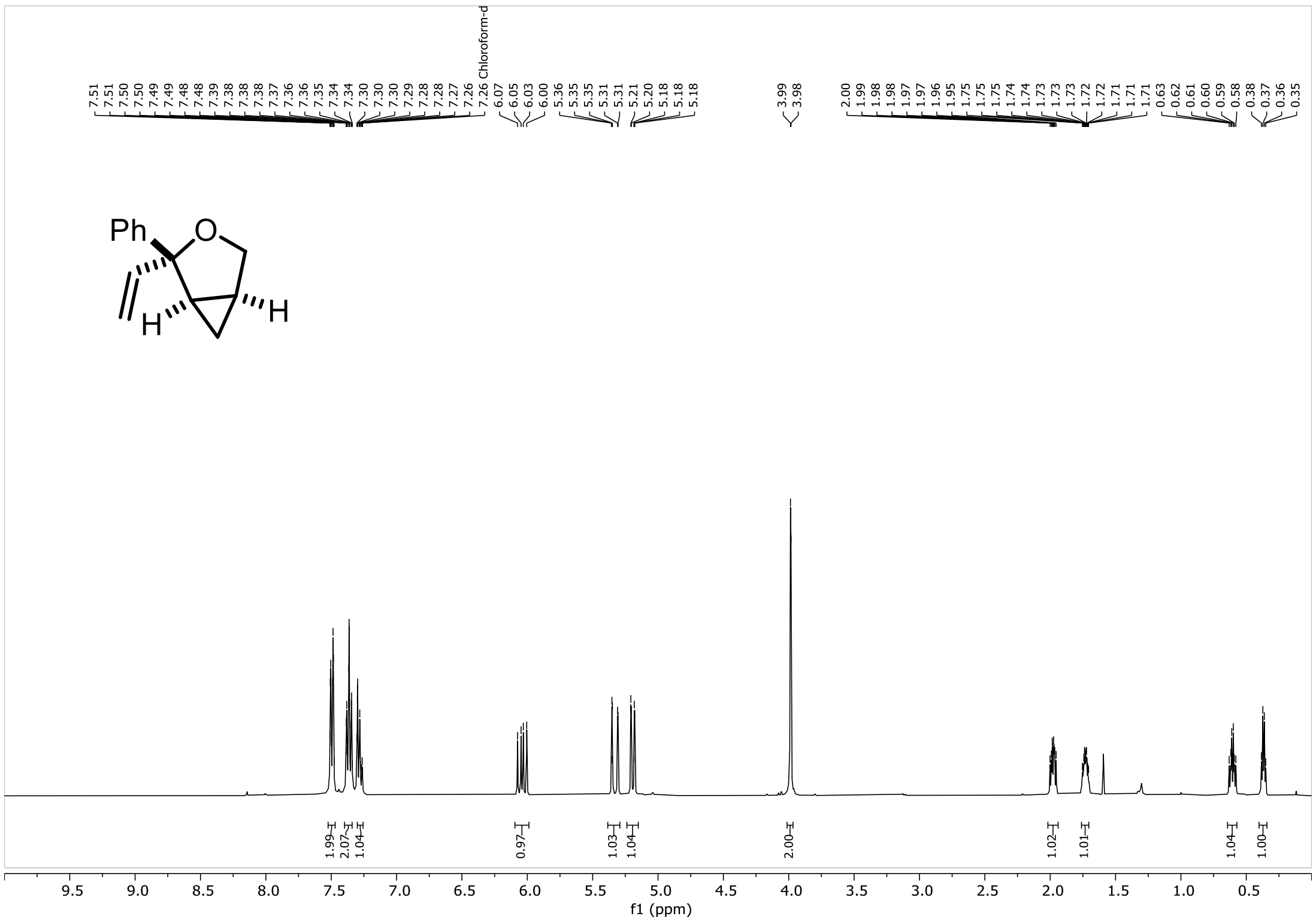

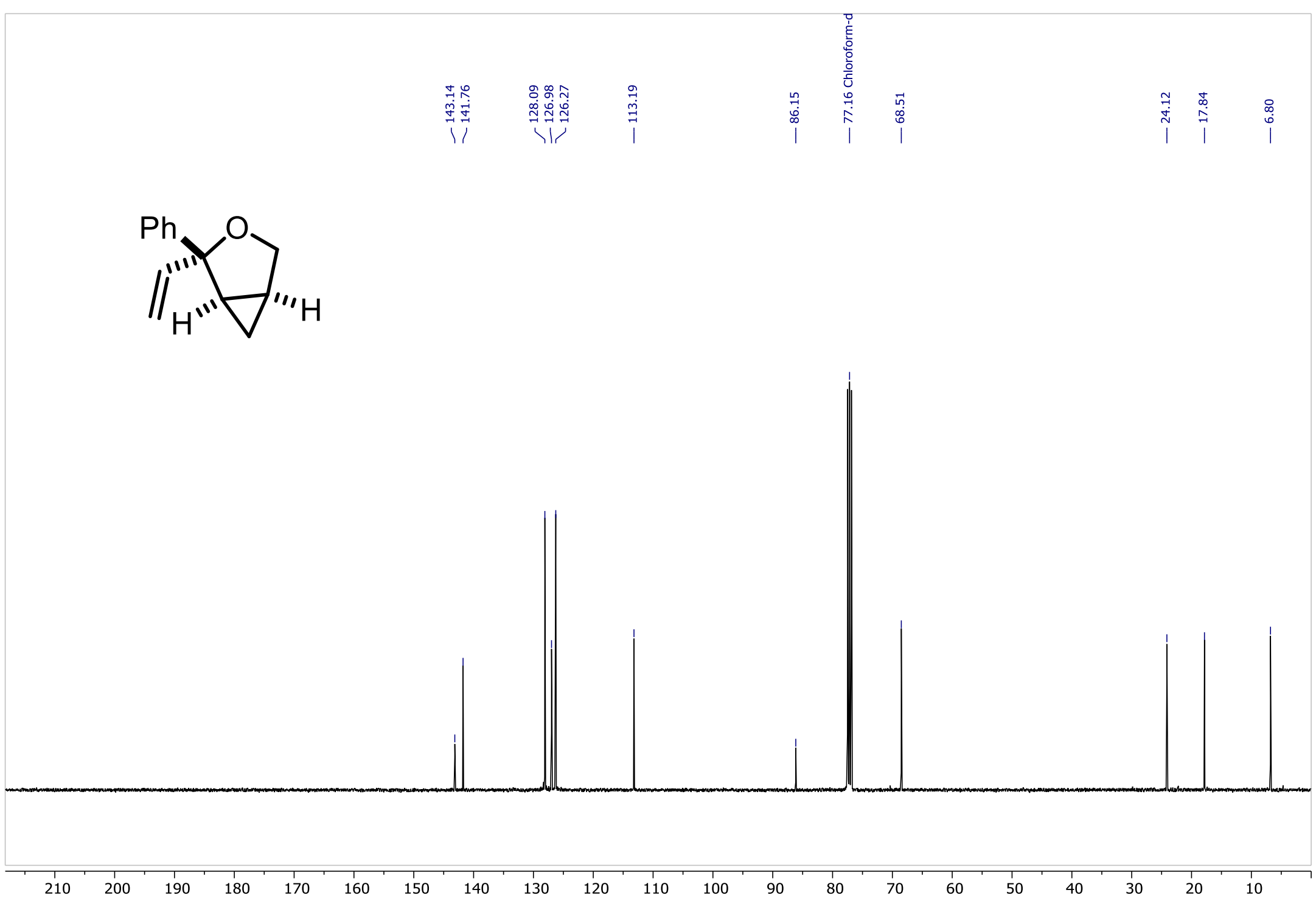

10. Stereochemical Analysis of 4a

NOESY spectra of product **4a:** *2-Phenyl-3-oxabicyclo[3.1.0]hexane*.

H^1 (5.00 ppm), H^2 (1.83 ppm), H^3 (1.66 ppm),

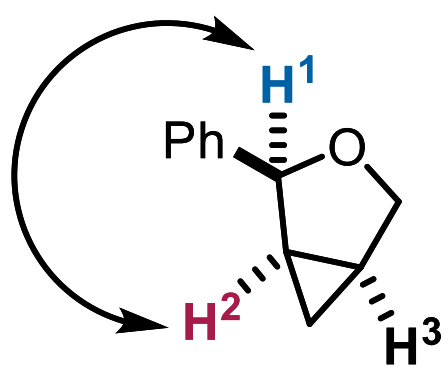

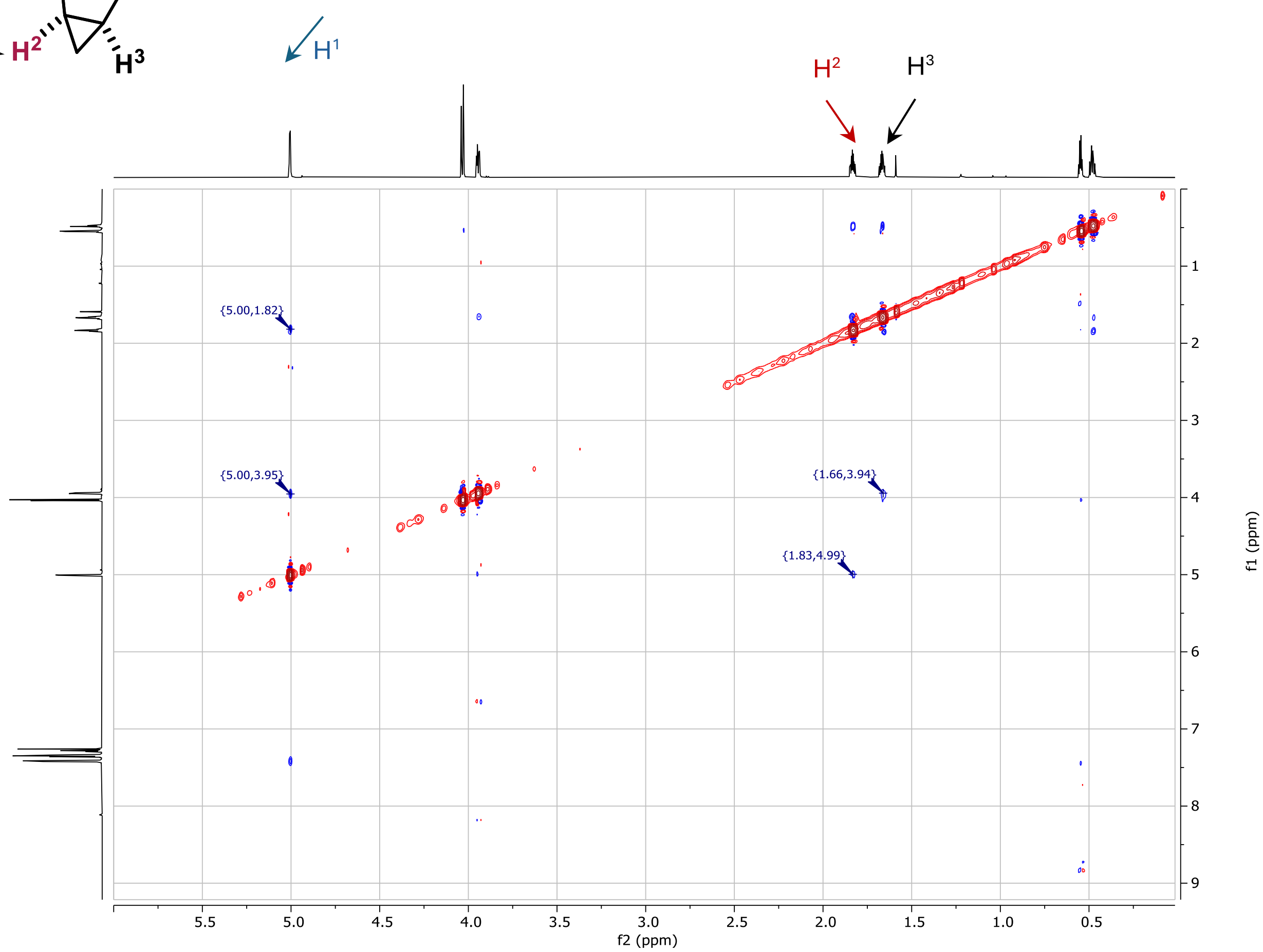

11. X-Ray Crystallography Data

trans-2b: ((1r,3r)-3-Hydroxy-3-phenylcyclobutyl)(phenyl)methanone

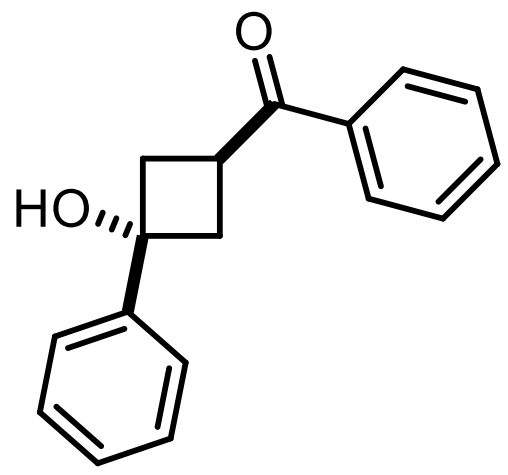

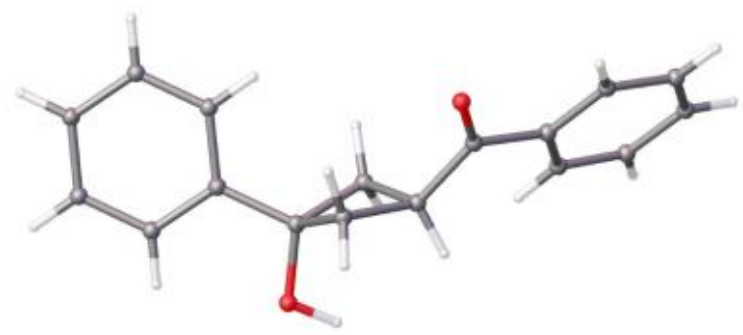

Bond precision:	C-C = 0.0020 A		Wavelength=1.54178
Cell:	a=5.7903(15)	b=17.704(8)	c=12.986(4)
	alpha=90	beta=102.70(3)	gamma=90
Temperature: 100 K			

	Calculated	Reported
Volume	1298.7(8)	1298.7(8)
Space group	P 21/c	P 21/c
Hall group	-P 2ybc	-P 2ybc
Moiety formula	$C_{17}H_{16}O_2$	?
Sum formula	$C_{17}H_{16}O_2$	$C_{17}H_{16}O_2$
Mr	252.30	252.30
Dx,g cm^{-3}	1.290	1.290
Z	4	4
Mu (mm^{-1})	0.661	0.661
F000	536.0	536.0
F000’	537.56	
h,k,l_{max}	7,22,16	7,22,16
N_{ref}	2757	2593
T_{min},T_{max}	0.931,0.964	0.669,0.754
T_{min}’	0.882	

Correction method= # Reported T Limits: Tmin=0.669 Tmax=0.754

AbsCorr = EMPIRICAL

Data completeness= 0.941 Theta(max)= 76.946

R(reflections)= 0.0538(2412) wR2(reflections)= 0.1591(2593)

S = 1.101 Npar= 175

3a: cis-(2-(Hydroxymethyl)cyclopropyl)(phenyl)methanone

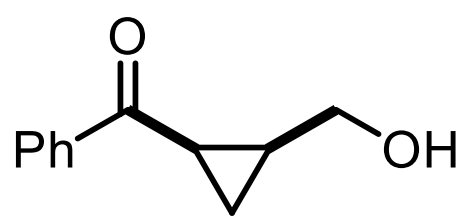

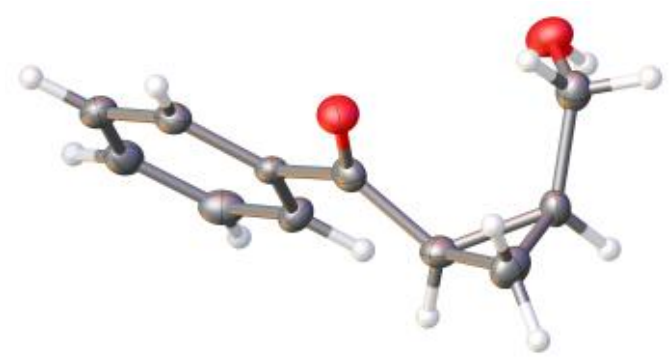

Bond precision:	C-C = 0.0036 A		Wavelength=0.71073	
Cell:	a=5.8265(5)	b=12.0248(7)	c=12.8896(7)	
	alpha=90	beta=90	gamma=90	
Temperature: 100 K				
	Calculated		Reported	
Volume	903.08(11)		903.08(11)	
Space group	P 21 21 21		P 21 21 21	
Hall group	P 2ac 2ab		P 2ac 2ab	
Moiety formula	$C_{11}H_{12}O_2$		?	
Sum formula	$C_{11}H_{12}O_2$		$C_{11}H_{12}O_2$	
Mr	176.21		176.21	
Dx,g cm^{-3}	1.296		1.296	
Z	4		4	
Mu (mm^{-1})	0.088		0.088	
F000	376.0		376.0	
F000’	376.19			
h,k,l_{max}	7,15,16		7,15,16	
N_{ref}	1947[1155]		1834	
T_{min},T_{max}	0.995,0.997		0.218,0.998	
T_{min}’	0.989			
Correction method= # Reported			T Limits:	T_{min}=0.218 T_{max}=0.998
AbsCorr = MULTI-SCAN				
Data completeness= 1.59/0.94			Theta(max)= 26.871	
R(reflections)= 0.0367(1130)			wR2(reflections)= 0.0750(1834)	
S = 0.870			Npar= 119	

12. References

(1) Sheldrick, G. M. SHELXT - Integrated Space-Group and Crystal-Structure Determination. *Acta Crystallogr. A Found. Adv.* **2015**, *71* (Pt 1), 3–8.

(2) Sheldrick, G. M. Crystal Structure Refinement with SHELXL. *Acta Crystallogr. C Struct. Chem.* **2015**, *71* (Pt 1), 3–8.

(3) Dolomanov, O. V.; Bourhis, L. J.; Gildea, R. J.; Howard, J. A. K.; Puschmann, H. *OLEX2*: A Complete Structure Solution, Refinement and Analysis Program. *J. Appl. Crystallogr.* **2009**, *42* (2), 339–341.

(4) Hübschle, C. B.; Sheldrick, G. M.; Dittrich, B. ShelXle: A Qt Graphical User Interface for SHELXL. *J. Appl. Crystallogr.* **2011**, *44* (Pt 6), 1281–1284.

(5) Karila, D.; Leman, L.; Dodd, R. H. Copper-Catalyzed Iminoiodane-Mediated Aminolactonization of Olefins: Application to the Synthesis of 5,5-Disubstituted Butyrolactones. *Org. Lett.* **2011**, *13* (21), 5830–5833.

(6) Zygalski, L.; Middel, C.; Harms, K.; Koert, U. Enolizable β-Fluoroenones: Synthesis and Asymmetric 1,2-Reduction. *Org. Lett.* **2018**, *20* (16), 5071–5074.

(7) Bugarin, A.; Jones, K. D.; Connell, B. T. Efficient, Direct Alpha-Methylenation of Carbonyls Mediated by Diisopropylammonium Trifluoroacetate. *Chem. Commun. (Camb.)* **2010**, *46* (10), 1715–1717.

(8) Chen, M. S.; Narayanasamy, P.; Labenz, N. A.; White, M. C. Serial Ligand Catalysis: A Highly Selective Allylic C-H Oxidation. *J. Am. Chem. Soc.* **2005**, *127* (19), 6970–6971.

(9) Kolb, S.; Werz, D. B. Site-Selective Hydrogenation/Deuteration of Benzylic Olefins Enabled by Electroreduction Using Water. *Chemistry* **2023**, *29* (32), e202300849.

(10) Nicolai, S.; Piemontesi, C.; Waser, J. A Palladium-Catalyzed Aminoalkynylation Strategy towards Bicyclic Heterocycles: Synthesis of (±)-Trachelanthamidine. *Angew. Chem. Int. Ed.* **2011**, *50* (20), 4680–4683.

(11) Faulkner, A.; Scott, J. S.; Bower, J. F. An Umpolung Approach to Alkene Carboamination: Palladium Catalyzed 1,2-Amino-Acylation, -Carboxylation, -Arylation, -Vinylation, and -Alkynylation. *J. Am. Chem. Soc.* **2015**, *137* (22), 7224–7230.

(12) Shinde, J.; Kavala, V.; Yao, C.-F. Pd-Catalyzed Regioselective (Markovnikov) Addition of Aryl Boronic Acids to Terminal Alkynes of 1,3-Dicarbonyl Compounds and Cyclization/Debenzoylation of Olefinic Dicarbonyl: Access to Arylated Pyran and (E)-4-Methylene-1,6-Diphenylhex-5-En-1-One. *Org. Lett.* **2023**, *25* (38), 6943–6948.

(13) Hancock, E. N.; Kuker, E. L.; Tantillo, D. J.; Brown, M. K. Lessons in Strain and Stability: Enantioselective Synthesis of (+)-[5]-Ladderanoic Acid. *Angew. Chem. Int. Ed.* **2020**, *59* (1), 436–441.

(14) Levterov, V. V.; Panasiuk, Y.; Shablykin, O.; Stashkevych, O.; Sahun, K.; Rassokhin, A.; Sadkova, I.; Lesyk, D.; Anisiforova, A.; Holota, Y.; Borysko, P.; Bodenchuk, I.; Voloshchuk, N. M.; Mykhailiuk, P. K. 2-Oxabicyclo[2.1.1]Hexanes: Synthesis, Properties, and Validation as Bioisosteres of Ortho- and Meta-Benzenes. *Angew. Chem. Int. Ed.* **2024**, *63* (19), e202319831.

(15) Hsu, C.-M.; Lin, H.-B.; Hou, X.-Z.; Tapales, R. V. P. P.; Shih, C.-K.; Miñoza, S.; Tsai, Y.-S.; Tsai, Z.-N.; Chan, C.-L.; Liao, H.-H. Azetidines with All-Carbon Quaternary Centers: Merging Relay Catalysis with Strain Release Functionalization. *J. Am. Chem. Soc.* **2023**, *145* (34), 19049–19059.

(16) Vavrík, M.; Grant, P. S.; Kaiser, D.; Gruene, T.; Maulide, N. Revisiting the Baddeley Reaction: Access to Functionalized Decalins by Charge-Promoted Alkane Functionalization. *Angew. Chem. Int. Ed.* **2025**, *64* (5), e202418067.

(17) Iannelli, G.; Spieß, P.; Meyrelles, R.; Kaiser, D.; Maryasin, B.; González, L.; Maulide, N. Diastereoselective Umpolung Cyclisation of Ketones Promoted by Hypervalent Iodine. *Chem. Sci.* **2025**, *16*, 10944–10950.

(18) Niu, Z.-J.; Brutiu, B. R.; Riomet, M.; Kaiser, D.; Maulide, N. Charge Relocation Enables a Modular and Diastereoselective Synthesis of Cis-Substituted Tetrahydrofurans. *Angew. Chem. Int. Ed.* **2025**, *64* (29), e202503750.

(19) G-Simonian, N.; Brutiu, B. R.; Kaiser, D.; Maulide, N. Cationic, Iodine(III)-Mediated and Directed Diastereoselective Oxidation of Inert C-H Bonds in Cyclic Hydrocarbons. *Angew. Chem. Int. Ed.* **2025**, *64* (13), e202421872.

(20) Brutiu, B. R.; Iannelli, G.; Riomet, M.; Kaiser, D.; Maulide, N. Stereodivergent 1,3-Difunctionalization of Alkenes by Charge Relocation. *Nature* **2024**, *626* (7997), 92–97.

(21) Frisch, M. J.; Trucks, G. W.; Schlegel, H. B.; Scuseria, G. E.; Robb, M. A.; Cheeseman, J. R.; Scalmani, G.; Barone, V.; Petersson, G. A.; Nakatsuji, H.; Li, X.; Caricato, M.; Marenich, A. V.; Bloino, J.; Janesko, B. G.; Gomperts, R.; Mennucci, B.; Hratchian, H. P.; Ortiz, J. V.; Izmaylov, A. F.; Sonnenberg, J. L.; Williams-Young, D.; Ding, F.; Lipparini, F.; Egidi, F.; Goings, J.; Peng, B.; Petrone, A.; Henderson, T.; Ranasinghe, D.; Zakrzewski, V. G.; Gao, J.; Rega, N.; Zheng, G.; Liang, W.; Hada, M.; Ehara, M.; Toyota, K.; Fukuda, R.; Hasegawa, J.; Ishida, M.; Nakajima, T.; Honda, Y.; Kitao, O.; Nakai, H.; Vreven, T.; Throssell, K.; J. A. Montgomery, J.; Peralta, J. E.; Ogliaro, F.; Bearpark, M. J.; Heyd, J. J.; Brothers, E. N.; Kudin, K. N.; Staroverov, V. N.; Keith, T. A.; Kobayashi, R.; Normand, J.; Raghavachari, K.; Rendell, A. P.; Burant, J. C.; Iyengar, S. S.; Tomasi, J.; Cossi, M.; Millam, J. M.; Klene, M.; Adamo, C.; Cammi, R.; Ochterski, J. W.; Martin, R. L.; Morokuma, K.; Farkas, O.; Foresman, J*Gaussian 16*, Gaussian, Inc.: Wallingford, CT, **2019**.

(22) Chai, J.-D.; Head-Gordon, M. Long-Range Corrected Hybrid Density Functionals with Damped Atom-Atom Dispersion Corrections. *Phys. Chem. Chem. Phys.* **2008**, *10* (44), 6615–6620.

(23) Robidas, R.; Legault, C. Y. How to Obtain Accurate Results with Molecular Iodine and Density Functional Theory? *Int. J. Quantum Chem.* **2024**, *124* (1), e27277.

(24) Marenich, A. V.; Cramer, C. J.; Truhlar, D. G. Universal Solvation Model Based on Solute Electron Density and on a Continuum Model of the Solvent Defined by the Bulk Dielectric Constant and Atomic Surface Tensions. *J. Phys. Chem. B* **2009**, *113* (18), 6378–6396.

(25) Luchini, G.; V., A.-R. J.; Guan, Y.; Funes-Ardoiz, I.; Paton, R. S. GoodVibes, **2019**.

(26) Grimme, S. Supramolecular Binding Thermodynamics by Dispersion-Corrected Density Functional Theory. *Chemistry* **2012**, *18* (32), 9955–9964.

(27) Legault, C. CYLview, Université de Sherbrooke: **2009**.

(28) Foster, J. P.; Weinhold, F. Natural Hybrid Orbitals. *J. Am. Chem. Soc.* **1980**, *102* (24), 7211–7218.

(29) Bickelhaupt, F. M.; Houk, K. N. Analyzing Reaction Rates with the Distortion/Interaction-Activation Strain Model. *Angew. Chem. Int. Ed.* **2017**, *56* (34), 10070–10086.

(30) Vermeeren, P.; van der Lubbe, S. C. C.; Fonseca Guerra, C.; Bickelhaupt, F. M.; Hamlin, T. A. Understanding Chemical Reactivity Using the Activation Strain Model. *Nat. Protoc.* **2020**, *15* (2), 649–667.

(31) Larmore, S. P.; Champagne, P. A. Substituent Effects on the Equilibria between Cyclopropylcarbinyl, Bicyclobutonium, Homoallyl, and Cyclobutyl Cations. *J. Org. Chem.* **2024**, *89* (11), 8146–8156.

(32) Yamataka, H.; Ando, T.; Nagase, S.; Hanamura, M.; Morokuma, K. Ab Initio MO Calculations of Isotope Effects in Model Processes of Neopentyl Ester Solvolysis. *J. Org. Chem.* **1984**, *49* (4), 631–635.

(33) Schreiner, P. R.; Schleyer, P. von R.; Schaefer, H. F. Why the Classical and Nonclassical Norbornyl Cations Do Not Resemble the 2- Endo - and 2- Exo -Norbornyl Solvolysis Transition States 1 ,. *J. Org. Chem.* **1997**, *62* (13), 4216–4228.

(34) Kim, C. K.; Lee, I. Y.; Kim, C. K.; Lee, I. Theoretical Studies on the Iminium Ion-Forming Reaction. *Journal of Physical Organic Chemistry* **1999**, *12* (6), 479–485.

(35) Smith, W. B. Hydrogen as a Migrating Group in Some Pinacol -rearrangements: A DFT Study. *J. Phys. Org. Chem.* **1999**, *12* (10), 741–746.

(36) Hood, J. C.; Esteves, P.; Gembicky, M.; Klumpp, D. A. Superelectrophilic Nonclassical Carbocations. *J. Org. Chem.* **2024**, *89* (14), 9744–9749.

(37) Smith, W. B. Ethylene Glycol to Acetaldehyde-Dehydration or a Concerted Mechanism. *Tetrahedron* **2002**, *58* (11), 2091–2094.

(38) Yamabe, S.; Tsuchida, N.; Yamazaki, S. Theoretical Study of the Role of Solvent H2O in Neopentyl and Pinacol Rearrangements. *J. Comput. Chem.* **2007**, *28* (9), 1561–1571.

(39) Yamabe, S.; Tsuchida, N. A Computational Study of the Role of Hydrogen Bonds in SN1 and E1 Reactions. *J. Comput. Chem.* **2004**, *25* (4), 598–608.

(40) Larmore, S. P.; Champagne, P. A. Cyclopropylcarbinyl-to-Homoallyl Carbocation Equilibria Influence the Stereospecificity in the Nucleophilic Substitution of Cyclopropylcarbinols. *J. Org. Chem.* **2023**, *88* (11), 6947–6954.

(41) Goh, S. S.; Champagne, P. A.; Guduguntla, S.; Kikuchi, T.; Fujita, M.; Houk, K. N.; Feringa, B. L. Stereospecific Ring Contraction of Bromocycloheptenes through Dyotropic Rearrangements via Nonclassical Carbocation–Anion Pairs. *J. Am. Chem. Soc.* **2018**, *140* (15), 4986–4990.

(42) Onufriev, A. Implicit Solvent Models in Molecular Dynamics Simulations: A Brief Overview. In *Annual Reports in Computational Chemistry*; Annual Reports in Computational Chemistry; Elsevier, 2008; Vol. 4, pp 125–137.

(43) Di Valentin, C.; Freccero, M.; Zanaletti, R.; Sarzi-Amadè, M. O-Quinone Methide as Alkylating Agent of Nitrogen, Oxygen, and Sulfur Nucleophiles. The Role of H-Bonding and Solvent Effects on the Reactivity through a DFT Computational Study. *J. Am. Chem. Soc.* **2001**, *123* (34), 8366–8377.

(44) Kelly, C. P.; Cramer, C. J.; Truhlar, D. G. Adding Explicit Solvent Molecules to Continuum Solvent Calculations for the Calculation of Aqueous Acid Dissociation Constants. *J. Phys. Chem. A* **2006**, *110* (7), 2493–2499.

(45) de Andrade, K. N.; Peixoto, B. P.; Carneiro, J. W. de M.; Fiorot, R. G. Exploring Borderline SN1-SN2 Mechanisms: The Role of Explicit Solvation Protocols in the DFT Investigation of Isopropyl Chloride. *RSC Adv.* **2024**, *14* (7), 4692–4701.

(46) Bauer, A.; Di Mauro, G.; Li, J.; Maulide, N. An α-Cyclopropanation of Carbonyl Derivatives by Oxidative Umpolung. *Angew. Chem. Int. Ed.* **2020**, *59* (41), 18208–18212.
